# Direct Cu-mediated aromatic ^18^F-labeling of highly reactive tetrazines for pretargeted bioorthogonal PET imaging†

**DOI:** 10.1039/d1sc02789a

**Published:** 2021-07-28

**Authors:** Rocío García-Vázquez, Umberto M. Battisti, Jesper T. Jørgensen, Vladimir Shalgunov, Lars Hvass, Daniel L. Stares, Ida N. Petersen, François Crestey, Andreas Löffler, Dennis Svatunek, Jesper L. Kristensen, Hannes Mikula, Andreas Kjaer, Matthias M. Herth

**Affiliations:** Department of Drug Design and Pharmacology, Faculty of Health and Medical Sciences, University of Copenhagen Jagtvej 160 2100 Copenhagen Denmark matthias.herth@sund.ku.dk; Cluster for Molecular Imaging, Department of Biomedical Sciences, University of Copenhagen Blegdamsvej 9 2100 Copenhagen Denmark; Department of Clinical Physiology, Nuclear Medicine & PET Rigshospitalet, Blegdamsvej 9 2100 Copenhagen Denmark; Institute of Applied Synthetic Chemistry, Technische Universität Wien (TU Wien) Getreidemarkt 9 1060 Vienna Austria

## Abstract

Pretargeted imaging can be used to visualize and quantify slow-accumulating targeting vectors with short-lived radionuclides such as fluorine-18 – the most popular clinically applied Positron Emission Tomography (PET) radionuclide. Pretargeting results in higher target-to-background ratios compared to conventional imaging approaches using long-lived radionuclides. Currently, the tetrazine ligation is the most popular bioorthogonal reaction for pretargeted imaging, but a direct ^18^F-labeling strategy for highly reactive tetrazines, which would be highly beneficial if not essential for clinical translation, has thus far not been reported. In this work, a simple, scalable and reliable direct ^18^F-labeling procedure has been developed. We initially studied the applicability of different leaving groups and labeling methods to develop this procedure. The copper-mediated ^18^F-labeling exploiting stannane precursors showed the most promising results. This approach was then successfully applied to a set of tetrazines, including highly reactive H-tetrazines, suitable for pretargeted PET imaging. The labeling succeeded in radiochemical yields (RCYs) of up to approx. 25%. The new procedure was then applied to develop a pretargeting tetrazine-based imaging agent. The tracer was synthesized in a satisfactory RCY of *ca.* 10%, with a molar activity of 134 ± 22 GBq μmol^−1^ and a radiochemical purity of >99%. Further evaluation showed that the tracer displayed favorable characteristics (target-to-background ratios and clearance) that may qualify it for future clinical translation.

## Abstract 2

A simple, scalable and reliable direct ^18^F-labeling procedure has been developed and applied to obtain a pretargeting tetrazine-based imaging agent with favorable *in vivo* characteristics.

## Graphical abstract

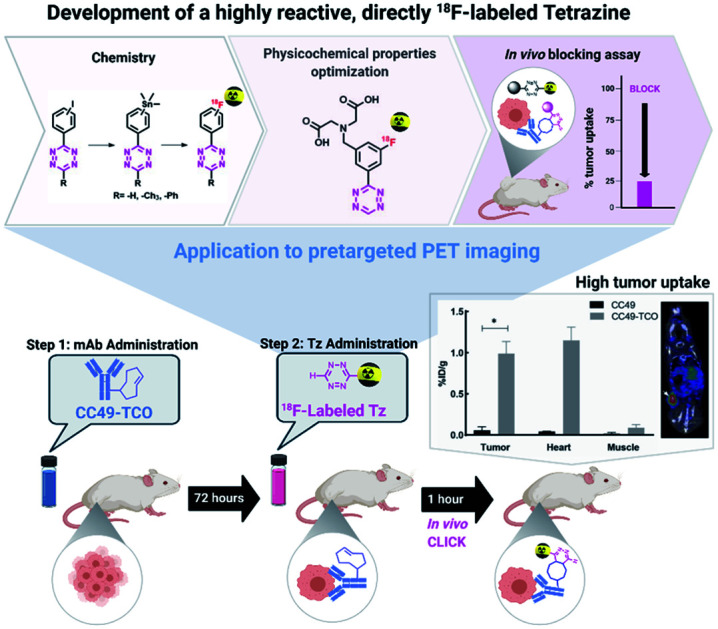

## Introduction

Positron Emission Tomography (PET) is a powerful, non-invasive and routinely used imaging tool in precision medicine or drug development.^1–3^ Its high sensitivity (the level of detection approaches 10^−12^ M of tracer), isotropism and quantitativity are in combination unmatched compared to any other *in vivo* molecular imaging technique.^4,5^ Fluorine-18 (^18^F) is considered as the best suited PET radionuclide for clinical applications as it provides almost ideal physical characteristics for molecular imaging. These include a relatively short positron range (2.4 mm max. range in water), a good branching ratio (96.7% positron decay) and a half-life of approx. 110 min, which enables the distribution of ^18^F-labeled tracers within a several hundred kilometers range.^6–8^ Recently, bioorthogonal chemistry has emerged as a versatile tool for pretargeted nuclear imaging of slow-accumulating targeting vectors such as monoclonal antibodies (mAbs) or other nanomedicines.^9–13^ Improved imaging contrast (up to 100-fold) and lower radiation burden to healthy tissue can be achieved using pretargeting compared to conventional imaging strategies.^14^ These improved imaging characteristics are a result of the temporal separation of the slow targeting process of nanomedicines from the actual imaging step. Consequently, the exceptional target specificity of nanomedicines as well as the optimal pharmacokinetics of small molecules for molecular imaging, *e.g.* selective target accumulation and rapid clearance from blood, can be exploited using pretargeted imaging.^15,16^ So far, the most prominent reaction for pretargeted imaging is the tetrazine (Tz) ligation.^11,17^ Excellent chemoselectivity, metabolic stability and high reactivity make the Tz ligation as exceptional as the biotin–(strept)avidin interaction for pretargeting strategies.^18–21^ The Tz ligation is driven by the Inverse-Electron-Demand Diels–Alder (IEDDA) cycloaddition between an electron-deficient Tz and a strained *trans*-cyclooctene (TCO) derivative, followed by a retro-Diels–Alder elimination of nitrogen.^10,22–24^ Despite efforts focused on TCO-based click imaging agents,^25,26^ the use of radiolabeled Tz has gradually emerged in recent literature.^10^

Throughout the last decade, the labeling of Tzs was mostly limited to chelation of radiometals such as ^64^Cu, ^89^Zr, ^44^Sc or ^68^Ga.^27–31^ In 2013, the first successful attempt to label a Tz moiety with a covalently bound PET radionuclide, *i.e.* with carbon-11, was reported by our group.^32^ Despite significant progress in the field, until recently all reported ^18^F-Tzs had electron-donating alkyl substituents at the Tz ring and thus had low reactivity towards TCOs.^21^ The reason for this is that highly reactive mono- or bis-(hetero)aryl-substituted Tzs decompose under the harsh conditions used for standard nucleophilic ^18^F-fluorination (S_N_2 or S_N_Ar) approaches.^14,21,33^ Only relatively base insensitive and less reactive Tzs could be radiolabeled, *via* an ^18^F-aliphatic substitution (S_N_2) strategy. Radiochemical yields (RCYs) up to 18% were achieved.^21^ More recently, the preparation of a highly reactive ^18^F-labeled glycosylated Tz by Keinänen and co-workers and an [^18^F]AlF-NOTA-labeled Tz radioligand by Meyer and co-workers were reported.^31–33^ The latest strategy added to this portfolio is the synthesis of ^18^F-radiolabeled tetrazines *via* the copper-catalyzed azide–alkyne cycloaddition.^14,21^

**Fig. 1:**
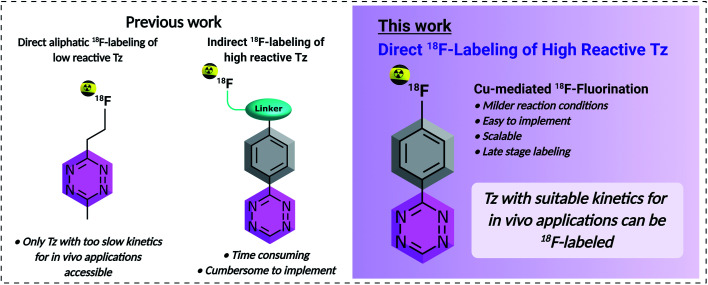
Comparison of previously reported ^18^F-labeling strategies of tetrazines *vs.* the direct aromatic ^18^F-labeling approach developed in this work.

Within this study, we aimed to develop a simple, scalable and reliable direct aromatic radiofluorination procedure that can be applied to access highly reactive ^18^F-labeled Tzs (Fig. 1). Direct aromatic [^18^F]fluorinations are in general fast and efficient and the corresponding fluoroarenes are more stable towards defluorination than their aliphatic counterparts.^34^ For these reasons, the synthesis of ^18^F-fluorinated aryls has found widespread application within the last decade.^8,35–38^ Typically, nucleophilic aromatic substitution (S_N_Ar) is the method of choice to radiolabel fluoroarenes. However, they require relatively strong basic conditions and high temperature, and as such, the S_N_Ar is not ideally suited to ^18^F-label structures containing highly reactive Tz moieties which are known to be base-sensitive.^21,39^ Recently, several mild aromatic ^18^F-labeling strategies have been reported that proceed at lower temperatures and with short reaction time, while using less basic reaction conditions. In particular, Cu-mediated oxidative fluorinations of tin and boronic esters or acids allow fluorination of electron-rich substrates under mild conditions.^40–44^ In this work, several S_N_Ar and oxidative fluorinations were screened in order to label highly reactive Tzs. Cu-mediated fluorinations of stannane precursors succeeded in moderate RCYs (d.c.) of 10–24% at the end of the synthesis (EOS). Based on these results, a new Tz, compound **21**, that possesses the necessary lipophilicity (log *D*_7.4_ < −3) and high rate constant (>50 000 M^−1^ s^−1^) for *in vivo* pretargeting experiments was designed.^14^ [^18^F]**21** was radiolabeled in a RCY (d.c.) of 11 ± 3%, with an *A*_m_ of 134 ± 22 GBq μmol^−1^ (d.c.) and a RCP of ≥99%.^35,37,40^ Pretargeted *in vivo* PET imaging in tumor-bearing mice showed a mean tumor uptake of [^18^F]**21** of 0.99 ± 0.14% ID per g (mean ± S.E.M.) after only 1 hour with a high mean tumor-to-muscle ratio of 10. We believe that the developed tracer shows pharmacokinetic properties warranting in depth preclinical evaluation in the near future and that the developed labeling method will pave the way for developing ^18^F-Tz based pretargeted imaging agents with favorable reaction kinetics, good metabolic stability and a pharmacokinetic profile required for bioorthogonal *in vivo* chemistry.

## Results and discussion

### Preparation of tetrazine precursors

**Fig. 2:**
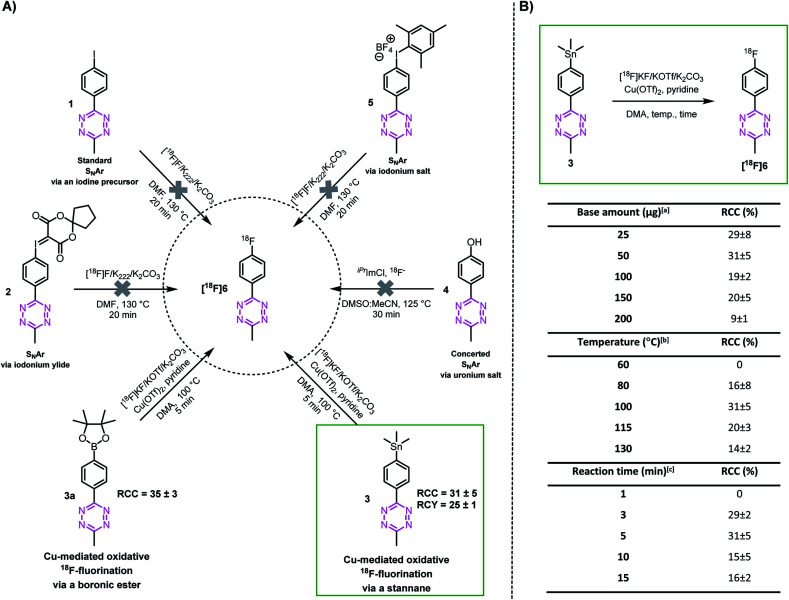
Proof of principle of ^18^F-labeling of a methyl-phenyl-Tz. (A) Radiolabeling strategies using different methyl-phenyl-Tz precursors. (B) Optimization of the Cu-mediated ^18^F-fluorination from stannane precursor **3** to [^18^F]**6**. ^a^Conditions: Cu(OTf)_2_, pyridine, [^18^F]KF, DMA, 100 °C, 5 min. ^b^Conditions: Cu(OTf)_2_, pyridine, [^18^F]KF (50 μg K_2_CO_3_), DMA, 5 min. ^c^Conditions: Cu(OTf)_2_, pyridine, [^18^F]KF (50 μg K_2_CO_3_), DMA, 100 °C. Radiochemical conversion (RCC) was determined by radio-TLC and radio-HPLC (*n* = 3). Radiochemical yield (RCY) was decay corrected to the starting amount of radioactivity received from the cyclotron and the isolated product without a formulation step (*n* = 3).

In order to explore whether highly reactive Tzs can be directly ^18^F-labeled, we investigate different nucleophilic ^18^F-labeling strategies, such as concerted nucleophilic aromatic substitution of uronium or iodonium salts,^36,45–47^ hypervalent iodonium based precursors,^48–50^ minimalistic labeling strategies^35,37^ and Cu-mediated reactions. Tz **6** was initially selected as a simple model as it is readily accessible and displays moderate stability against strong bases. This allows us to first study the suitability of these types of reaction before attempting the most promising strategy with base-sensitive Tz-scaffolds. Precursors **1–5** and reference compound **6** were synthesized similarly to reported procedures (ESI, section S2†).^51,52^ In our hands, ^18^F-labeling strategies including S_N_Ar approaches resulted in decomposition of the product. In contrast, the Cu-mediated ^18^F-fluorination starting from the stannane (**3**) and the boronic ester (**3a**) precursor resulted in the radiolabeling of the model compound [^18^F]**6**. The radiochemical conversion (RCC) was approximately 14% at the first attempt (Fig. 2A).^53^ However, only the stannane precursors of more reactive Tzs could be synthesized. Boronic ester precursors decomposed (ESI, section S2†). Consequently, further optimization of temperature, reaction time and amount of base at the start was only performed with precursor **3** and led to an improvement of approx. 30% RCC (Fig. 2B).

### Synthesis and radiolabeling of tetrazines with increased reaction kinetics

With these encouraging results, we decided to study whether more reactive Tzs could also be labeled using this strategy. Tzs with stepwise increased reactivity were selected to investigate the scope of our radiofluorination method (Table 1). Precursors and reference compounds were synthesized using known procedures (ESI, section S2†)^51,52,54,55^ and radiolabeling was conducted using the best conditions identified labeling our model compound [^18^F]**6**. Moderate RCCs (12–31%) as well as sufficient decay-corrected (d.c.) RCYs (10–24%) were observed at the end of synthesis (EOS) for methyl-, phenyl- and H-Tzs (Table 1). The automated synthesis including [^18^F]fluoride concentration and drying, labeling, high-performance liquid chromatography (HPLC) separation and formulation was carried out within 90 minutes (ESI, section S3†). Radiochemical purity (RCP) was >99% for all prepared ^18^F-fluorinated tetrazines, and the molar activity (*A*_m_) was 190 ± 10 GBq μmol^−1^ (d.c) (*n* = 3) for [^18^F]**6**, which is in line with the results obtained for other tracers on the used module and the same starting activity. The typical activity yield was 2.5–3 GBq starting from ∼12 GBq fluoride-18. Pyridyl structures could not be labeled using this labeling strategy, most likely due to a chelation of the copper ion with the respective pyridyl moieties of the Tz.^56^ As expected, the most reactive Tz resulted in the lowest RCY. However, the observed RCYs are in the range of many clinically applied PET tracers.^41,42,57^

**Table 1:** Product scope for the Cu-mediated ^18^F-fluorination of aryl-tetrazines starting from stannane precursors

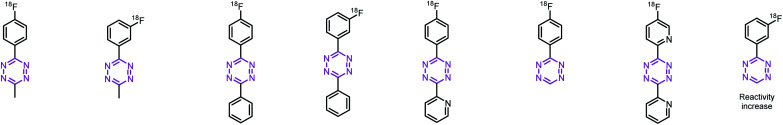								
Compound	[^18^F]**6**	[^18^F]**7**	[^18^F]**8**	[^18^F]**9**	[^18^F]**10**	[^18^F]**11**	[^18^F]**12**	[^18^F]**13**
RCC^a^ [%]	30 ± 5	28 ± 1	30 ± 5	31 ± 2	—d	18 ± 4	—d	12 ± 1
RCY^b^ [%]	23 ± 1	26 ± 2	23 ± 2	24 ± 3	—d	15 ± 3	—d	11 ± 3
Rel. reactivity^c^	1.0	1.4	1.8	3.0	10	70	91	96
RCP^a^ [%]	≥99	≥99	≥99	≥99	—d	99	—d	99

a Radiochemical conversion (RCC) and radiochemical purity (RCP) were determined by radio-HPLC and radio-TLC (*n* = 3).

b Radiochemical yield (RCY) was decay corrected to the starting amount of radioactivity received from the cyclotron and the isolated product without a formulation step (*n* = 3).

c Relative IEDDA reactivity was calculated based on second order rate constants determined by stopped-flow measurements of the respective reference compound (^19^F-Tz) with *trans*-cyclooctene at 25 °C in 1,4-dioxane or acetonitrile (see the ESI).

d No product could be isolated.

### Effect of synthesis and radiolabeling of H-Tz upon substitution in the aryl ring

To study the effect of different substituents at the aryl ring, [^18^F]**13** was selected for further analysis since it displayed the highest relative IEDDA reactivity. The IEDDA reactivity is one of the most crucial factors for pretargeted *in vivo* applications.^14^ Electron-donating and electron-withdrawing substituents were introduced on the phenyl moiety at different positions, and the substitution pattern was correlated with its synthetic accessibility and RCCs (used as a surrogate for RCYs, RCC correlated with RCY in our study) (Table 2). While all 5-substituted stannane precursors were successfully synthesized from respective iodo-Tz intermediates, only the methyl and/or methoxy derivatives among 4- and 6-substituted stannanes could be prepared – most likely due to steric hindrance.^40–42^ During ^18^F-fluorinations, only 3,5-disubstituted stannane precursors provided useful RCCs in the order of 14–31%. No or only minimal product formation could be observed with a different substitution profile (Table 2). Hence, the 3,5-disubstitution pattern was identified to be best suited for Cu-mediated oxidative ^18^F-fluorinations.

**Table 2:** Product scope with respect to different substituted phenyl-Tzs for the Cu-mediated ^18^F-fluorination from stannane precursors

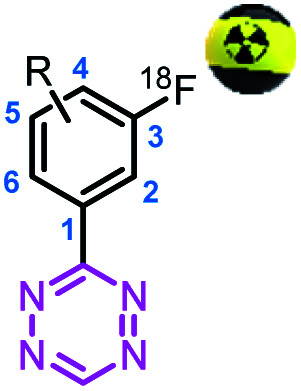				
*R*	Compound	Position		
	(-*p*, -*m*, -*o*)	**4** (-*p*)	**5** (-*m*)	**6** (-*o*)
–CH_3_	[^18^F]**14**	—a	14 ± 3^b^	—c
–OCH_3_	[^18^F]**15**	4 ± 1	17 ± 3^b^	—c
–NHCOCH_3_	[^18^F]**16**	—a	31 ± 3^b^	—d
–CONH_2_	[^18^F]**17**	—a	24 ± 2^b^	—d
–CONHCH_3_	[^18^F]**18**	—a	20 ± 3^b^	—d

a Stannane precursor could not be synthesized.

b RCCs were determined by radio-HPLC and radio-TLC (*n* = 3).

c Decomposed during the Cu-mediated ^18^F-fluorination.

d Iodo-Tz intermediate could not be synthesized.

### Design of the promising tetrazine

**Fig. 3:**
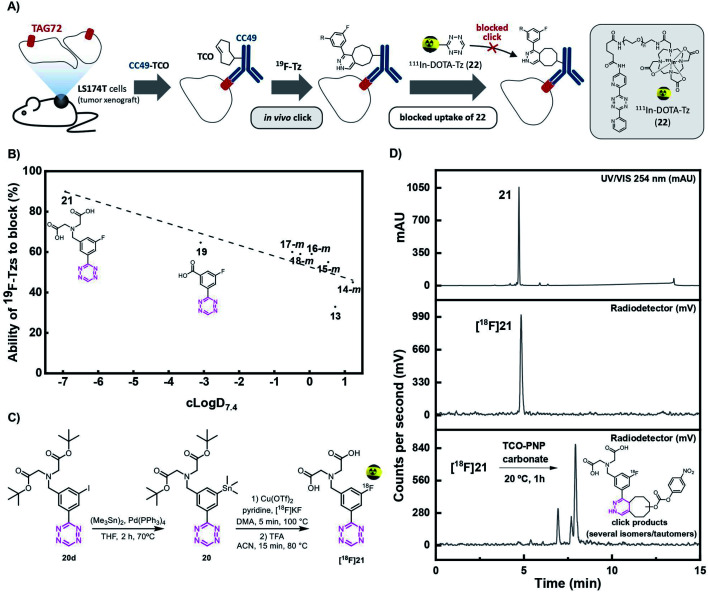
(A) Visualization of the blocking assay. Tumor-bearing mice were first injected with CC49-TCO, 72 h before administration of the non-radioactive Tz. After 1 h, ^111^In-labeled Tz ([^111^In]**DOTA-Tz**, (**22**), was injected and an *ex vivo* biodistribution was carried out 22 h p.i. in order to determine the blocking effect of the non-radioactive Tz. (B) Ability of ^19^F-Tzs (**13**, **14–18-m**, **19**, and **21**) to block ^111^In-DOTA-Tz (**22**) in the *in vivo* screening assay described in (A) (*n* = 3) (ESI, section S5†). (C) Synthesis and radiolabeling of [^18^F]**21**. (D) Analytical-HPLC of reference compound **21** (UV/Vis, 254 nm) (upper panel), and radio-HPLC of the purified [^18^F]**21** (middle panel) and ligation product after click reaction with the TCO-PNP carbonate (**23**), one hour post-injection (lower panel). Analytical HPLC conditions: Luna 5 μm C18(2) 100 Å, 150 mm × 4.6 mm; eluents: A, H_2_O with 0.1% TFA; B, MeCN with 0.1% TFA; gradient from 100% A to 100% B over 12 min, back to 100% A over 3 min, flow rate 2 mL min^−1^.

Recently, our group has demonstrated that the performance of Tz-derivatives and probes for pretargeted *in vivo* ligation strongly depends on the lipophilicity and the IEDDA reactivity of the Tz agent. Low polarity (clog *D*_7.4_ < −3) and rate constants > 50 000 M^−1^ s^−1^ for the click reaction with axially configured TCO tags (Dulbecco's PBS, 37 °C) resulted in the best target-to-background ratios.^14^ In this respect, we designed two highly reactive Tzs, which contained polar groups and allowed for direct ^18^F-labeling. Tz **19** possesses a clog *D*_7.4_ of −3.09 and a rate constant of 91 000 M^−1^ s^−1^, and Tz **21** possesses a clog *D*_7.4_ of −6.93 and a rate constant of 82 000 M^−1^ s^−1^ (ESI, section S5†). Both compounds were synthesized in sufficient yields *via* a Pinner-like synthesis (ESI, section S2†) and evaluated in an *in vivo* assay recently described by our group (Fig. 3A).^14^ This assay, inspired by traditional receptor blocking studies, applies anti-TAG72 mAb CC49 modified with axially configured TCO tags (CC49-TCO) and [^111^In]DOTA-Tz (**22**), which has previously successfully been used for pretargeted imaging in (TAG72 expressing) LS174T tumors.^28^ In short, tumor-bearing mice are injected with a CC49-TCO, 72 h before the non-labeled Tz is to be tested. Subsequently, [^111^In]DOTA-Tz (**22**) is injected after 1 h and a biodistribution is performed 22 h later (ESI, section S5†).^14,28^ The assay evaluates the blocking ability of the non-labeled Tz, and therefore allows estimation of the *in vivo* ligation performance of this compound. Higher blocking capacity is correlated with better *in vivo* performance of the respective Tz.^14^ As expected – based on our previous data – we found a correlation between clog *D*_7.4_ and *in vivo* blocking of the Tzs tested in the assay (Pearson's *r* = 0.89, *p* <0.01) and the most polar Tz **21** (clog *D*_7.4_ = −6.93) resulting in the best blocking effect (90%) (Fig. 3B) was selected for further development.

### Synthesis, radiolabeling and stability of final compound [^18^F]**21**

The shelf stability of Tz **21** was assessed in phosphate-buffered saline (PBS) by analytical-HPLC. Compound **21** did not show degradation in PBS after 12 h at 37 °C at a concentration of 2 nmol mL^−1^ (98%). Consequently, the stannane precursor **20** was synthesized in 4 steps (ESI, section S2†). Radiolabeling succeeded in a one-pot, two-step sequence with a RCY (d.c.) of 11 ± 3% (*n* = 4) and an overall synthesis time of *ca.* 90 minutes including synthesis, separation and formulation. [^18^F]**21** was obtained with an *A*_m_ of 134 ± 22 GBq μmol^−1^ (d.c.), a RCP of ≥99% (*n* = 4) and an activity yield of 600–700 MBq (EOS) starting from ∼12 GBq fluoride-18 (Fig. 3C and D). [^18^F]**21** was stable in PBS at room temperature for minimum 4 h and rapidly reacted with TCO-PNP carbonate (**23**) as confirmed by radio-HPLC (Fig. 3D and ESI, section S3†). Residual amounts of Cu and Sn in the final formulated solution were analyzed by ICP-MS and found to be well below the allowed limits specified in the ICH Guidelines (41–60 and 2.3–3.0 μg L^−1^*vs.* 300 and 600 μg per day, respectively).^41,58–60^

### Pretargeted PET *in vivo* imaging

**Fig. 4:**
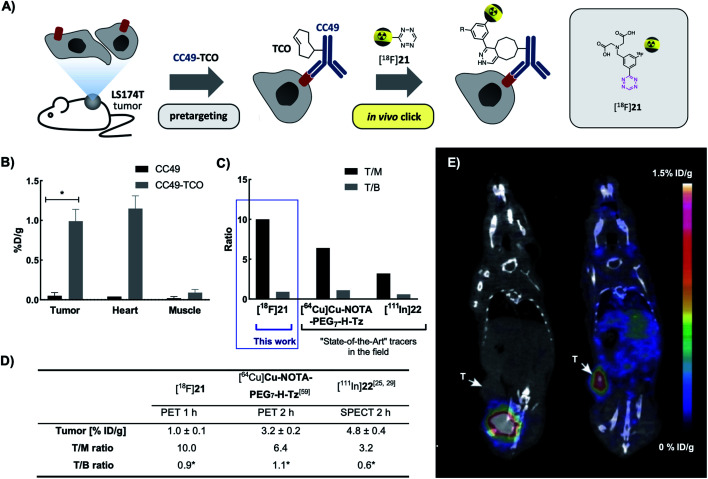
PET/CT scan of CC49-TCO pretargeted [^18^F]**21** in LS174T tumor xenograft bearing mice. (A) General pretargeted imaging approach. (B) PET-image derived mean %ID per g in tumor, heart and muscle tissue 1 h p.i. of [^18^F]**21**. Data are shown as mean ± S.E.M; *n* = 3 per group. *p < 0.05 (Welch's *t*-test). (C and D) Image-derived tumor uptake (mean %ID per g), tumor-to-muscle (T/M) and tumor-to-blood ratio (T/B) of [^18^F]**21** in comparison with the “state-of-the-art” applied Tz imaging agents [^64^Cu]**Cu-NOTA-PEG7-H-Tz** (PET 2 h p.i., *n* = 4) and [^111^In]**22** (SPECT 2 h p.i., *n* = 4). Tumor uptake and ratios of [^64^Cu]**Cu-NOTA-PEG7-H-Tz** and [^111^In]**22** 2 h p.i. in nude BALB/c mice bearing subcutaneous LS174T tumor xenografts pretreated with CC40-TCO (100 μg) have recently been published.^28,60^ Data are shown as mean ± standard error of mean (SEM). *Image-derived uptake in heart from SPECT and PET images used as a surrogate for blood.^28,60^ (E) Representative images from PET/CT-scans 1 h p.i. of [^18^F]**21**. Mice were administered with either non-modified CC49 (left) or CC49-TCO (right), 72 h prior to [^18^F]**21** injection. Arrows indicate LS174T tumor xenografts. Scale bar indicates mean %ID per g.

Next, we evaluated the performance of [^18^F]**21** in pretargeted PET imaging (Fig. 4A). Balb/c nude mice bearing LS174T tumor xenografts (*n* = 3 per group) were injected i.v. with either CC49-TCO (100 μg, 3.9 nmol, ∼7 TCOs per mAb) or non-modified CC49 (control). After 72 h, [^18^F]**21** (2.86 ± 0.99 MBq/100 μL) was administered and the mice were PET/CT scanned after 1 h. Image-derived uptake in tumor, heart (surrogate for blood) and muscle tissue was quantified as percentage injected dose per gram (mean %ID per g) (Fig. 4B–E). After completion of the scan, mice were euthanized and *ex vivo* biodistribution was performed (ESI, section S6†). Mice pretreated with CC49-TCO demonstrated a mean tumor uptake of [^18^F]**21** of 0.99 ± 0.14% ID per g (mean ± S.E.M.). The tracer displayed good target-to-background ratios with muscle uptake < 0.15% ID per g for all animals (Table S9†). This was also evident from PET/CT images, where tumor uptake in the CC49-TCO group was clearly visible (Fig. 4E). The mean tumor-to-blood ratio was 0.9, and thereby the specific uptake is similar to what was previously reported for other pretargeted imaging agents in the same tumor model.^14^

In contrast, a mean tumor-to-muscle ratio of 10 was detected which in fact is significantly higher compared to what has previously been found for the “state-of-the-art” Tz-based imaging agents [^18^F]**22** and [^64^Cu]**Cu-NOTA-PEG7-H-Tz** in a similar pretargeting set-up (LS174T bearing mice, using CC49-TCO 72 h prior to tracer injection, similar imaging timeframes) (Fig. 4C and D).^29,61^ However, [^18^F]**21** showed a 3 to 5-fold lower tumor uptake compared to those imaging agents (Fig. 4E).^29,61^ All tissues including tumors showed low ^18^F-uptake in control animals (CC49) (tumor uptake of 0.05 ± 0.04% ID per g). The findings from the imaging experiment were confirmed by *ex vivo* biodistribution data (Table S10†). Except for the tumor, the only tissue where the tracer uptake was significant was blood. This accumulation is likely caused by the *in vivo* ligation of [^18^F]**21** to CC49-TCO still circulating in the bloodstream, an observation that has been reported before for other pretargeting pairs.^10^ If residual mAbs are removed from the blood pool by *e.g.* a clearing agent, subsequent injection of [^18^F]**21** will likely result in an improved tumor-to-blood ratio.^10^

## Conclusion

In conclusion, this work enabled the first direct ^18^F-labeling of highly reactive Tzs starting from stannane precursors *via* a Cu-mediated approach. Applying this strategy, we have successfully prepared a new ^18^F-Tz, [^18^F]**21**, with highly favorable characteristics for pretargeted *in vivo* imaging. The developed procedure is simple, short, reproducible and scalable. Therefore, it is more suitable for clinical applications than previously used multistep ^18^F-labeling strategies. We are thus convinced that our method for the direct radiofluorination of highly reactive tetrazines will improve and accelerate the clinical translation of pretargeted imaging based *in vivo* click chemistries.

## Data availability

All data needed for this article is published within this article or in its ESI.†

## Author contributions

The organic synthesis was carried out through contributions of RGV, UMB, DLS, INP, and FC. The radiolabeling experiments were performed by RGV, VS, and INP. *In vivo* studies were performed by LH, JTJ, and AK. DS, AL and HM evaluated the Tz reaction kinetics. The study was designed by MMH, AK, RGV and UMB. The manuscript was written through contributions of all authors. All authors have given approval to the final version of the manuscript.

## Conflicts of interest

The authors declare no competing financial interest. All animal experiments in this study were approved by national animal welfare committees in Austria and Denmark, and the experiments were performed in accordance with European guidelines.

## Supplementary Material

SC-012-D1SC02789A-s001

SC-012-D1SC02789A-s002
