# Supplementary material for: Direct Cu-mediated aromatic 18F-labeling of highly reactive tetrazines for pretargeted bioorthogonal PET imaging
Source: Chem Sci. 2021 Jul 28;12(35):11668–75. doi: 10.1039/d1sc02789a (PMC8442695; doi:10.1039/d1sc02789a)
Supplement: SC-012-D1SC02789A-s001 [file SC-012-D1SC02789A-s001.pdf]

Electronic Supplementary Material (ESI) for Chemical Science.
This journal is © The Royal Society of Chemistry 2021

Direct Cu-Mediated Aromatic ^{18}F-Labeling of Highly Reactive Tetrazines for Pretargeted Bioorthogonal PET Imaging

Rocío García-Vázquez[a,c,‡], Umberto M. Battisti[a,‡], Jesper T. Jørgensen[b,c], Vladimir Shalgunov[a,b,c], Lars Hvass[b,c], Daniel L. Stares[a], Ida N. Petersen[b], François Crestey[a], Andreas Löffler[d], Dennis Svatunek[d], Jesper L. Kristensen[a], Hannes Mikula[d], Andreas Kjaer[b,c,*], Matthias M. Herth[a,c,*]

[a]Department of Drug Design and Pharmacology, Faculty of Health and Medical Sciences, University of Copenhagen, Jagtvej 160, 2100 Copenhagen, Denmark; [b]Cluster for Molecular Imaging, Department of Biomedical Sciences, University of Copenhagen, Blegdamsvej 9, 2100 Copenhagen, Denmark; [c]Department of Clinical Physiology, Nuclear Medicine & PET, Rigshospitalet, Blegdamsvej 9, 2100 Copenhagen, Denmark; [d]Institute of Applied Synthetic Chemistry, Technische Universität Wien (TU Wien), Getreidemarkt 9, 1060 Vienna, Austria. [‡]These authors contributed equally to this work. *Corresponding authors

Keywords: *tetrazines • bioorthogonal chemistry • molecular imaging • cancer • pretargeted imaging • fluorine-18 • PET*

Table of Contents

Section S1: General Procedures 3

Section S2: Organic Syntheses 4

General Procedure A. Synthesis of 3,6-disubstituted 1,2,4,5-tetrazines. 16

General Procedure B. Synthesis of 3,6-disubstituted 1,2-dihydro-1,2,4,5-tetrazines. 20

General Procedure C. Synthesis of 3-substituted-6-H-1,2,4,5-tetrazines. 21

Synthesis of ylide tetrazine 37

Synthesis of iodonium salt tetrazine 38

Synthesis of boronic ester tetrazine 39

General Procedure D.1. Synthesis of stannanes tetrazines 39

General Procedure D.2. Synthesis of stannanes tetrazines 44

Compounds for blocking studies and final stannanes precursors 46

Section S3: Radiochemistry 50

Section S4: Antibody Production and Modification 58

Section S5: *In Vitro* Studies 59

Blocking assay[24] 60

Section S6: *In Vivo* Studies 63

Pretargeted PET imaging of TCO-modified mAb (CC49-TCO) with [^{18}F]**21** 63

Section S7: Analytical Radio-HPLC and semi-preparative HPLC chromatograms of formulated [^{18}F]Tzs 65

Section S8: References 77

Section S9: NMR Spectra and Analytical HPLC 79

Section S1: General Procedures

Methods. NMR spectra were acquired on a 600 MHz Bruker Avance III HD (600 MHz for ^{1}H and 151 MHz for ^{13}C), a 400 MHz Bruker Avance II (400 MHz for ^{1}H, 376 MHz for ^{19}F and 101 MHz for ^{13}C) and a 400 MHz Bruker Avance UltraShield (400 MHz for ^{1}H, 376 MHz for ^{19}F and 101 MHz for ^{13}C), using Chloroform-*d*, MeOD or DMSO-d_6 as deuterated solvent and with the residual solvent as the internal reference. For all NMR experiences the deuterated solvent signal was used as the internal lock. Coupling constants (*J* values) are given in Hertz (Hz). Multiplicities of ^{1}H NMR signals are reported as follows: s, singlet; d, doublet; dd, doublet of doublets; ddd, doublet of doublets of doublets; dt, doublet of triplets; t, triplet; q, quartet; m, multiplet; br, broad signal. NMR spectra of all compounds are reprocessed in MestReNova software (version 12.0.22023) from original FID's files. Yields refer to isolated compounds estimated to be >90% pure as determined by ^{1}H NMR (25 °C) and analytical HPLC (SI, Section S2). Analytical HPLC method: Thermo Fisher UltiMate 3000 with a C-18 column (Luna 5 µm C18(2) 100 Å, 150 mm x 4.6 mm). Eluents: A, H_2O with 0.1% TFA; B, MeCN with 0.1% TFA. Gradient from 100% A to 100% B over 12 min, back to 100% A over 3 min, flow rate 2 mL/min. Detection by UV absorption at λ = 254 nm on a UVD 170U detector. High resolution mass spectrometry (HRMS) was performed as matrix-assisted laser desorption/ionization time-of-flight mass spectrometry (MALDI-TOF). Analyses were performed in positive ion mode with ionization on a ThermoQExactive Orbitrap mass spectrometer (Thermo Scientific) equipped with an AP-SMALDI 10 ion source (TransmitMIT) and operated with mass resolving power 140,000 at *m/z* 200 and lock-mass for internal mass calibration. Samples were dissolved in a matrix consisting of 2,5-dihydrooxybenzoic acid, 20 mg/mL, (positive mode). Thin-layer chromatography (TLC) was carried out on silica gel 60 F_{254} plates from Merck (Germany). Visualization was accomplished by UV lamp (254 nm). Preparative HPLC was carried out on an UltiMate HPLC system (Thermo Scientific) consisting of an LPG-3200BX pump (10 mL/min), a Rheodyne 9725i injector, a 10 mL loop, a MWD-3000SD detector (254 nm), and an AFC-3000SD automated fraction collector, using a Gemini-NX C18 column (21.2 x 250 mm, 5 µm, 110Å) (Phenomenex) equipped with a guard. Purifications were performed using linear gradients of 0.1% TFA in MiliQ-H_2O (A) and 0.1% TFA, 10% MiliQ-H_2O in MeCN (B). Data was acquired and processed using Chromeleon Software v. 6.80. Semi-preparative HPLC was performed on the same system using a Luna 5µ C18 column (250 x 10 mm) with a flow rate of 3 mL/min. Automated Flash Column Chromatography was performed on a CombiFlash NextGen 300+ system supplied by TeleDyne ISCO, equipped with RediSep silica packed columns. Detection of the compounds was carried out by means of a UV-Vis variable wavelength detector operating from 200 to 800 nm and by Evaporative Light Scattering Detector (ELSD). Solvent systems for separation were particular for each compound but consisted of various mixtures of heptane, EtOAc, DCM and MeOH. Microwave-assisted synthesis was carried out in a Biotage Initiator apparatus operating in single mode; the microwave cavity producing controlled irradiation at 2.45 GHz (Biotage AB, Uppsala, Sweden). The reactions were run in sealed vessels. These experiments were performed by employing magnetic stirring and a fixed hold time using variable power to reach (during 1–2 min) and then maintain the desired temperature in the vessel for the programmed time period. The temperature was monitored by an IR sensor focused on a point on the reactor vial glass. The IR sensor was calibrated to internal solution reaction temperature by the manufacturer. Mass spectra analysis was completed using MS-Acquity-A: Waters Acquity UPLC with QDa-detector.

Materials. All reagents and solvents were purchased from commercial suppliers and used without further purification. Anhydrous tetrahydrofuran (THF) was obtained from a SG Water solvent purification system (Pure Process Technology). Anhydrous dimethyl sulfoxide (DMSO), *N*,*N*-dimethylacetamide (DMA), MeCN and pyridine were purchased from commercially suppliers and stored under argon. Reactions requiring anhydrous conditions were carried out under inert atmosphere (nitrogen or argon) and using oven-dried glassware (152 °C). Syringes used to transfer anhydrous solvents or reagents were purged with argon prior to use. Other solvents were analytical or HPLC grade and were used as received.

The following abbreviations are used: MW: microwave; DCM: dichloromethane; DMSO: dimethyl sulfoxide; MeOH: methanol; EtOAc: ethyl acetate; PIDA: phenyliodine(III) diacetate; DMF: dimethylformamide; DEAD: diethyl azodicarboxylate; DIPEA: N,N-diisopropylethylamine; TEA: triethylamine; THF: tetrahydrofuran; EtOH: ethanol; Et_2O: diethyl ether; *m*CPBA: meta-chloroperoxybenzoic acid; MeCN MeCN; DMA: *N*,*N*-dimethylacetamide; NBS: N-Bromosuccinimide; AIBN: azobisisobutyronitrile; r.t.: room temperature; CDI: 1,1′-carbonyldiimidazole; TFA: trifluoroacetic acid; ^{me}CgPPh: 1,3,5,7-tetramethyl-6-phenyl-2,4,8-trioxa-6-phosphaadamantane.

Section S2: Organic Syntheses

Schematic overview to synthesize precursors, reference compounds and radiolabeled ^{18}F-compounds

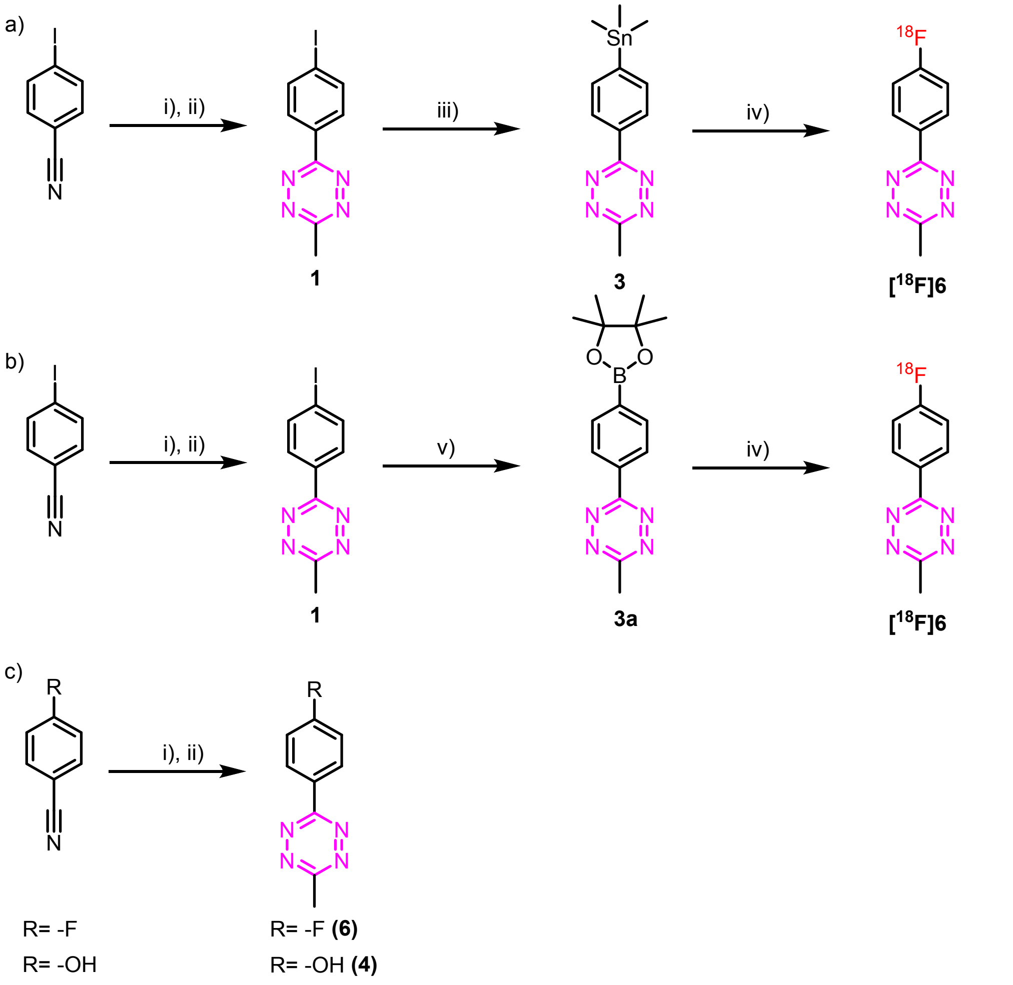

Scheme S1. *Synthesis of Tzs **3**(a), **3a**(b), and [^{18}F]**6** (a), **6** and **4** (c). i) MeCN, Zn(OTf)$_2$, $NH_2NH_2 \cdot H_2O$, EtOH, 60 ºC, 24 h; ii) $NaNO_2$, AcOH, 0 ºC, 20 min, 39% (**1**), 20% (**4**), 30% (**6**); iii) $(Me_3Sn)_2$, Pd(OAc)$_2$, ^{me}CgPPh, THF, 70 ºC, MW, 30 min, 76%; iv) B2pin2, Pd2dba3, XPhos, KOAc, 1,4-Dioxane, 24 h, 110ºC, 63%; v) Cu(OTf)$_2$, pyridine, [^{18}F]KF, DMA, 5 min, 100 °C, 30% RCC (a), 35% RCC (b).*

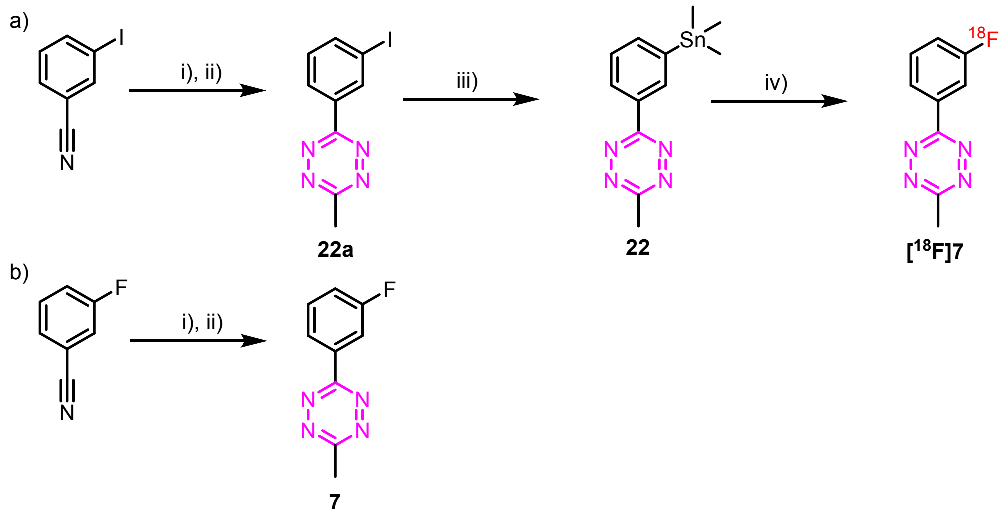

Scheme S2. *Synthesis of Tzs [^{18}F]**7** (a) and **7** (b). i) MeCN , Zn(OTf)$_2$, $NH_2NH_2 \cdot H_2O$, EtOH, 60 ºC, 24 h; ii) $NaNO_2$, AcOH, 0 ºC, 20 min, 29% (**22a**), 42% (**7**); iii) $(Me_3Sn)_2$, Pd(OAc)$_2$, ^{me}CgPPh, THF, 70 ºC, MW, 30 min, 65%; iv) Cu(OTf)$_2$, pyridine, [^{18}F]KF, DMA, 5 min, 100 °C, 28% RCC.*

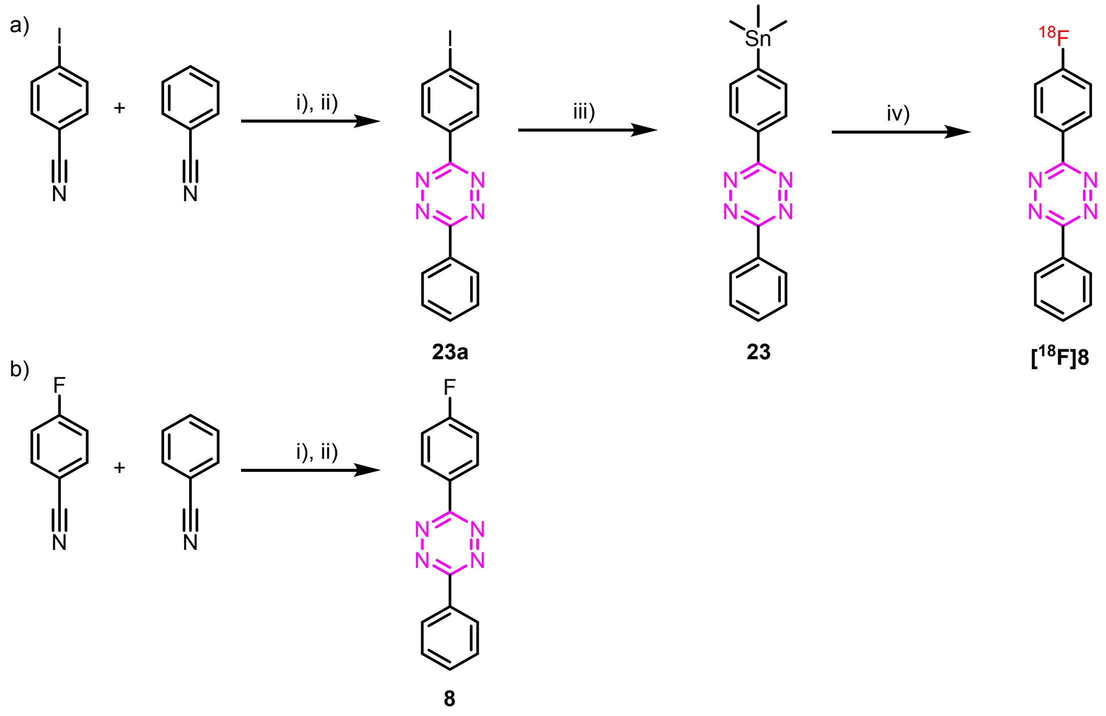

Scheme S3. *Synthesis of Tzs [^{18}F]**8** (a) and **8** (b). i) Zn(OTf)$_2$, $NH_2NH_2 \cdot H_2O$, EtOH, 60 ºC, 24 h; ii) $NaNO_2$, AcOH, 0 ºC, 20 min, 10% (**23a**), 8% (**8**); iii) $(Me_3Sn)_2$, Pd(OAc)$_2$, ^{me}CgPPh, THF, 70 ºC, MW, 30 min, 95%; iv) CU Cu(OTf)$_2$, pyridine, [^{18}F]KF, DMA, 5 min, 100 °C, 30% RCC.*

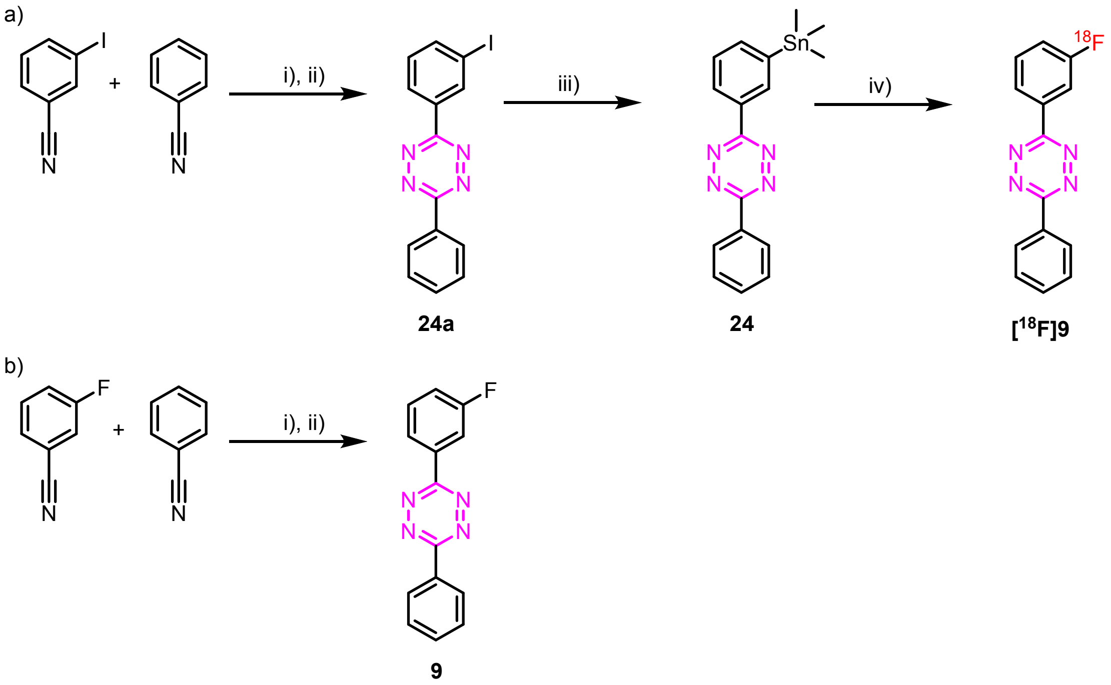

Scheme S4. *Synthesis of Tzs [^{18}F]**9** (a) and **9** (b). i) Zn(OTf)$_2$, NH$_2$NH$_2$ · H$_2$O, EtOH, 60 ºC, 24 h; ii) NaNO$_2$, AcOH, 0 ºC, 20 min, 14% (**24a**), 9% (**9**); iii) (Me$_3$Sn)$_2$, Pd(OAc)$_2$, ^{me}CgPPh, THF, 70 ºC, MW, 30 min, 85%; iv) Cu(OTf)$_2$, pyridine, [^{18}F]KF, DMA, 5 min, 100 °C, 31% RCC.*

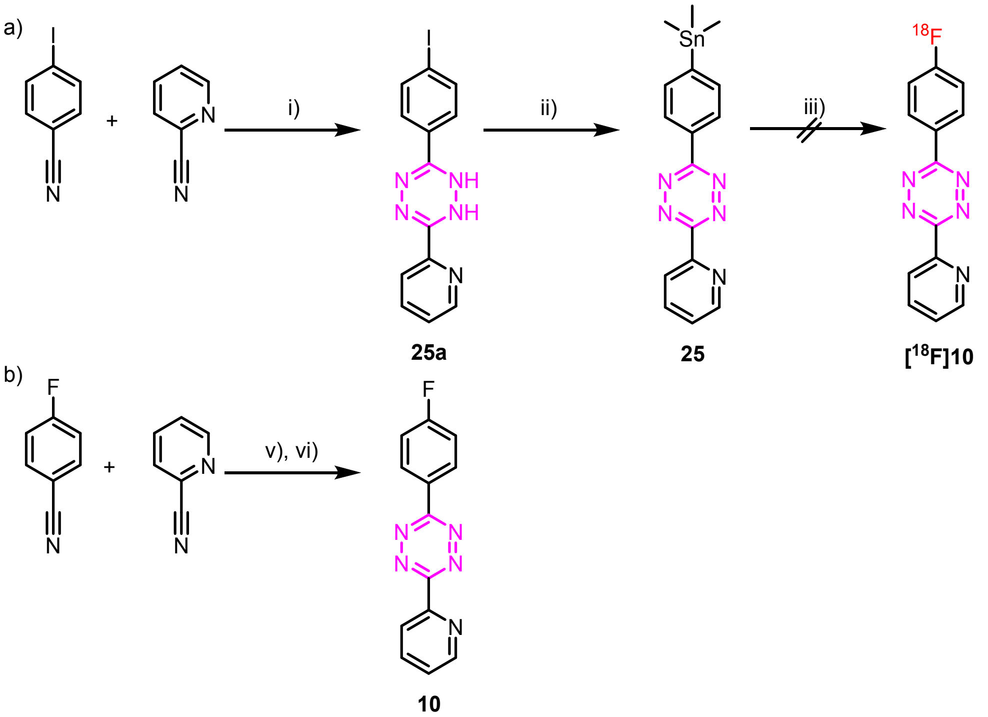

Scheme S5. *Synthesis of Tzs [^{18}F]**10** (a) and **10** (b). i) S$_8$, NH$_2$NH$_2$ · H$_2$O, EtOH, 50 ºC, 24 h, 19% (**25a**); ii) (Me$_3$Sn)$_2$, Pd(OAc)$_2$, ^{me}CgPPh, THF, 70 ºC, MW, 30 min, 49%; iii) Cu(OTf)$_2$, pyridine, [^{18}F]KF, DMA, 5 min, 100 °C, 0% RCC; v) Zn(OTf)$_2$, NH$_2$NH$_2$ · H$_2$O, EtOH, 60 ºC, 24 h; vi) NaNO$_2$, AcOH, 0 ºC, 20 min, 16% (**10**).*

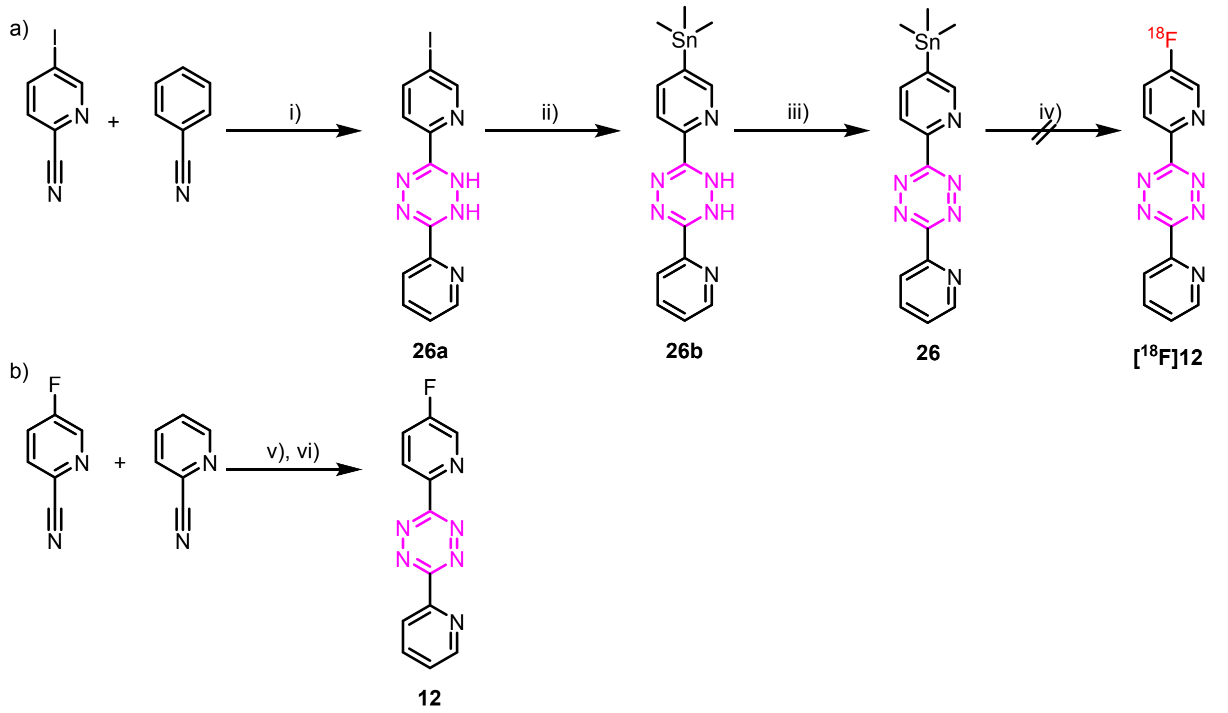

Scheme S6. *Synthesis of Tzs [*^{18}*F]**12** (a) and **12** (b). i) *S_8*, *$NH_2NH_2 \cdot H_2O$*, EtOH, 50 ºC, 24 h, 15% (**26a**); ii) *$(Me_3Sn)_2$*, *$Pd(OAc)_2$*, *^{me}*CgPPh, THF, 70 ºC, MW, 30 min, 85%; iii) PIDA, DCM, 0 ºC, 80%; iv) *$Cu(OTf)_2$*, pyridine, [*^{18}*F]KF, DMA, 5 min, 100 °C, 0% RCC; v) *$Zn(OTf)_2$*, *$NH_2NH_2 \cdot H_2O$*, EtOH, 60 ºC, 24 h; vi) *$NaNO_2$*, AcOH, 0 ºC, 20 min, 18% (**12**).*

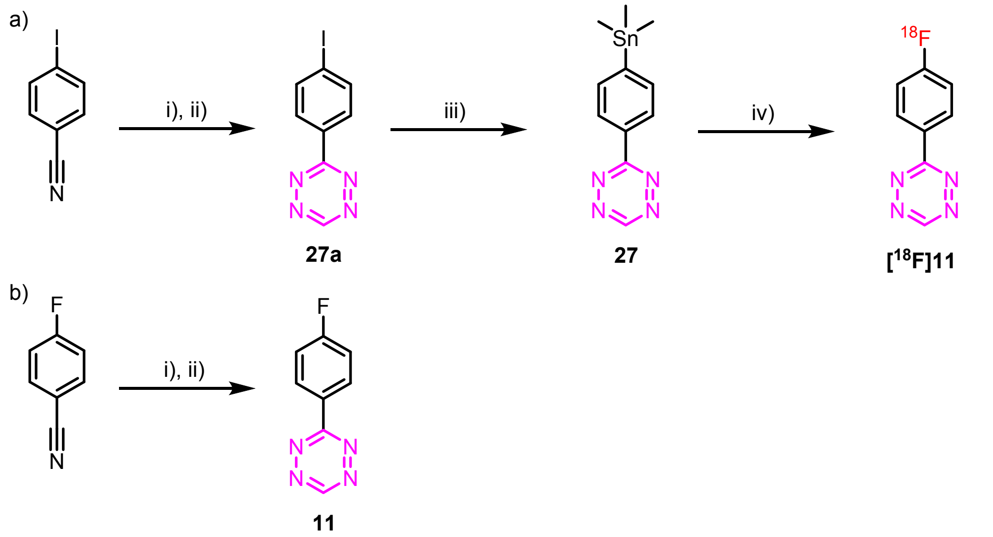

Scheme S7. *Synthesis of Tzs [*^{18}*F]**11** (a) and **11** (b). i) DCM, *S_8*, *$NH_2NH_2 \cdot H_2O$*, EtOH, 50 ºC, 24 h; ii) *$NaNO_2$*, AcOH, 0 ºC, 20 min, 27% (**27a**), 34% (**11**); iii) *$(Me_3Sn)_2$*, *$Pd(OAc)_2$*, *^{me}*CgPPh, THF, 70 ºC, MW, 30 min., 60%; iv) *$Cu(OTf)_2$*, pyridine, [*^{18}*F]KF, DMA, 5 min, 100 °C, 18% RCC.*

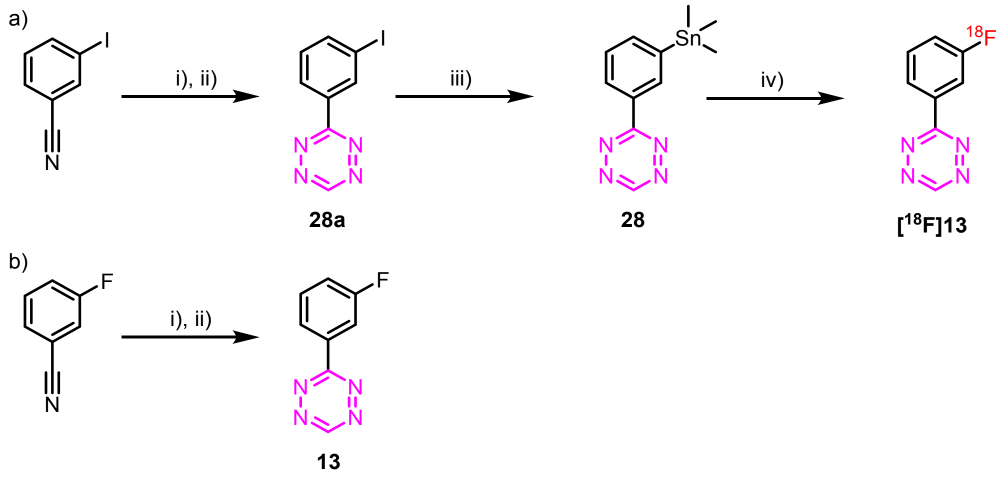

Scheme S8. *Synthesis of Tzs [^{18}F]**13** (a) and **13** (b). i) DCM, S_8, $NH_2NH_2 \cdot H_2O$, EtOH, 50 ºC, 24 h; ii) $NaNO_2$, AcOH, 0 ºC, 20 min, 33% (**28a**), 34% (**13**); iii) $(Me_3Sn)_2$, $Pd(OAc)_2$, ^{me}CgPPh, THF, 70 ºC, MW, 30 min., 58%; iv) $Cu(OTf)_2$, pyridine, [^{18}F]KF, DMA, 5 min, 100 °C, 12% RCC.*

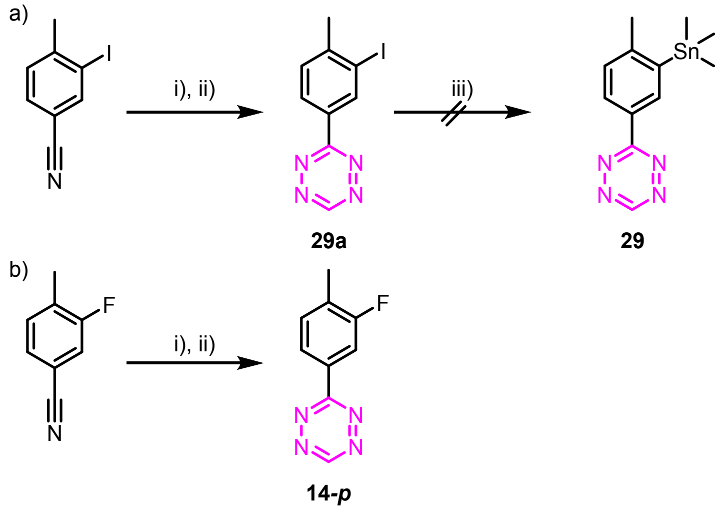

Scheme S9. *Synthesis of Tzs [^{18}F]**14-p** (a) and **14-p** (b). i) DCM, S_8, $NH_2NH_2 \cdot H_2O$, EtOH, 50 ºC, 24 h; ii) $NaNO_2$, AcOH, 0 ºC, 20 min, 18% (**29a**), 29% (**14**); iii) $(Me_3Sn)_2$, $Pd(OAc)_2$, ^{me}CgPPh, THF, 70 ºC, MW, 30 min., 0%.*

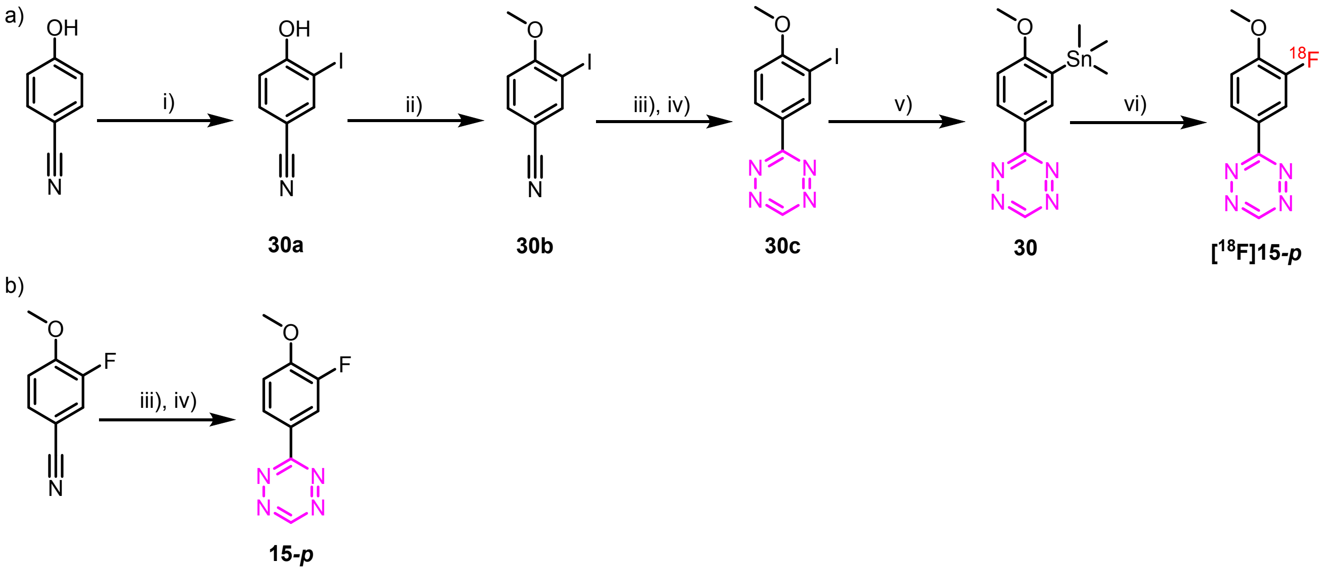

Scheme S10. *Synthesis of Tzs [*^{18}*F]**15-p** (a) and **15-p** (b). i) KI, I_2, NH_4OH, r.t., 20 h, 79%; ii) K_2CO_3, $(CH_3)_2SO_4$, acetone, 70 °C, 90 min., 88%; iii) DCM, S_8, $NH_2NH_2 \cdot H_2O$, EtOH, 50 ºC, 24 h; iv) $NaNO_2$, AcOH, 0 ºC, 20 min, 20% (**30c**), 29% (**15-p**); v) $(Me_3Sn)_2$, $Pd(OAc)_2$, ^{me}CgPPh, THF, 70 ºC, MW, 30 min., 36%; vi) $Cu(OTf)_2$, pyridine, [^{18}F]KF, DMA, 5 min, 100 °C, 4% RCC.*

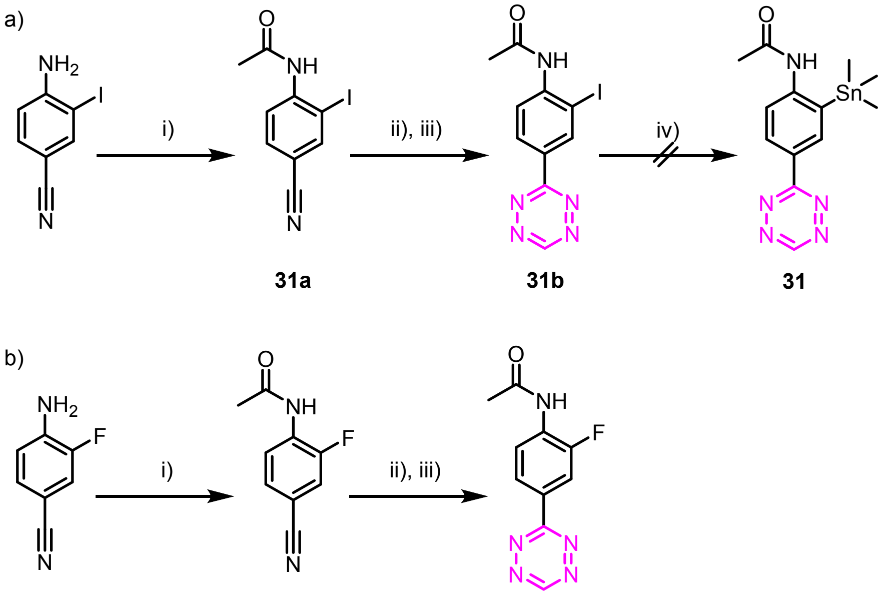

Scheme S11. *Synthesis of Tzs [*^{18}*F]**16-p** (a) and **16-p** (b). i) Acetic anhydride, DCM, r.t., 12 h, 52% (**31a**), 55% (**16a-p**); ii) DCM, S_8, $NH_2NH_2 \cdot H_2O$, EtOH, 50 ºC, 24 h; iii) $NaNO_2$, AcOH, 0 ºC, 20 min, 21% (**31b**), 40% (**16-p**); iv) $(Me_3Sn)_2$, $Pd(OAc)_2$, ^{me}CgPPh, THF, 70 ºC, MW, 30 min, 0%.*

a)

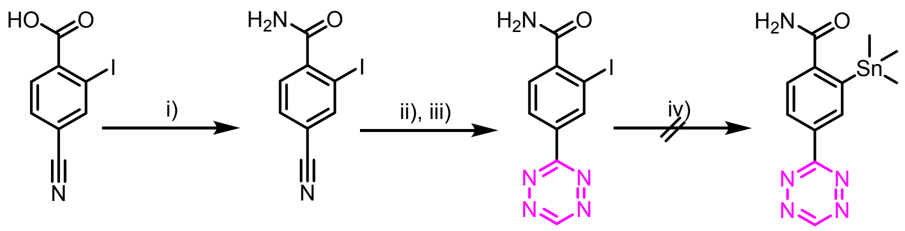

32a **32b** **32**

b)

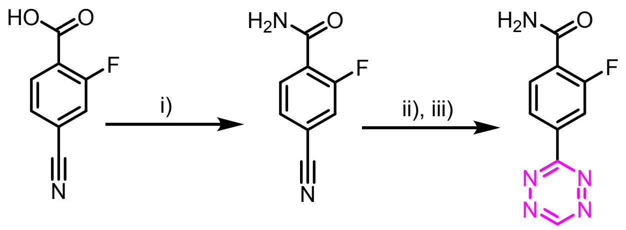

17a-*p* **17-*p***

Scheme S12. *Synthesis of Tzs [^{18}F]**17-p** (a) and **17-p** (b). i) CDI, NH_4OH, MeCN, 1 h, 79% (**32a**), 89% (**16a-p**); ii) DCM, S_8, $NH_2NH_2 \cdot H_2O$, EtOH, 50 ºC, 24 h; iii) $NaNO_2$, AcOH, 0 ºC, 20 min, 41% (**32b**), 20% (**16-p**); v) $(Me_3Sn)_2$, $Pd(OAc)_2$, ^{me}CgPPh, THF, 70 ºC, MW, 30 min., 0%.*

a)

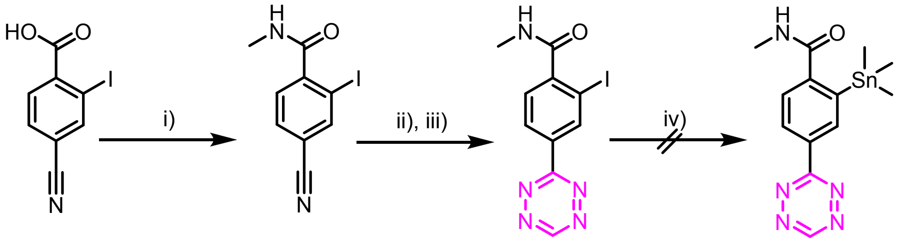

33a **33b** **33**

b)

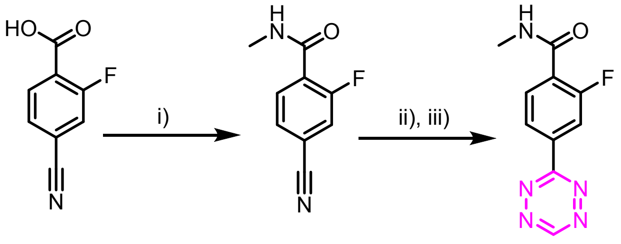

18a-*p* **18-*p***

Scheme S13. *Synthesis of Tzs [^{18}F]**18-p** (a) and **18-p** (b). i) CDI, NH_4CH_3, MeCN, r.t., 1 h, 81% (**33a**), 80% (**17a-p**); ii) DCM, S_8, $NH_2NH_2 \cdot H_2O$, EtOH, 50 ºC, 24 h; iii) $NaNO_2$, AcOH, 0 ºC, 20 min, 35% (**33b**), 36% (**17-p**); v) $(Me_3Sn)_2$, $Pd(OAc)_2$, ^{me}CgPPh, THF, 70 ºC, MW, 30 min., 0%.*

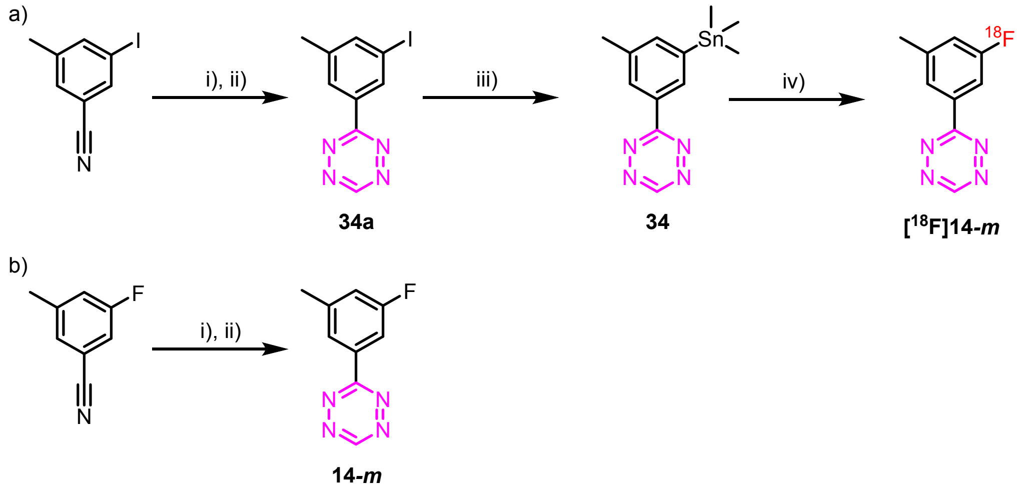

Scheme S14. *Synthesis of Tzs [^{18}F]**14-m** (a) and **14-m** (b). i) DCM, S_8, $NH_2NH_2 \cdot H_2O$, EtOH, 50 ºC, 24 h; ii) $NaNO_2$, AcOH, 0 ºC, 20 min, 22% (**34a**), 34% (**14-m**); iii) $(Me_3Sn)_2$, $Pd(PPh_3)_4$, THF, 65 ºC, MW, 3 h, 27%; iv) $Cu(OTf)_2$, pyridine, [^{18}F]KF, DMA, 5 min, 100 °C, 14% RCC.*

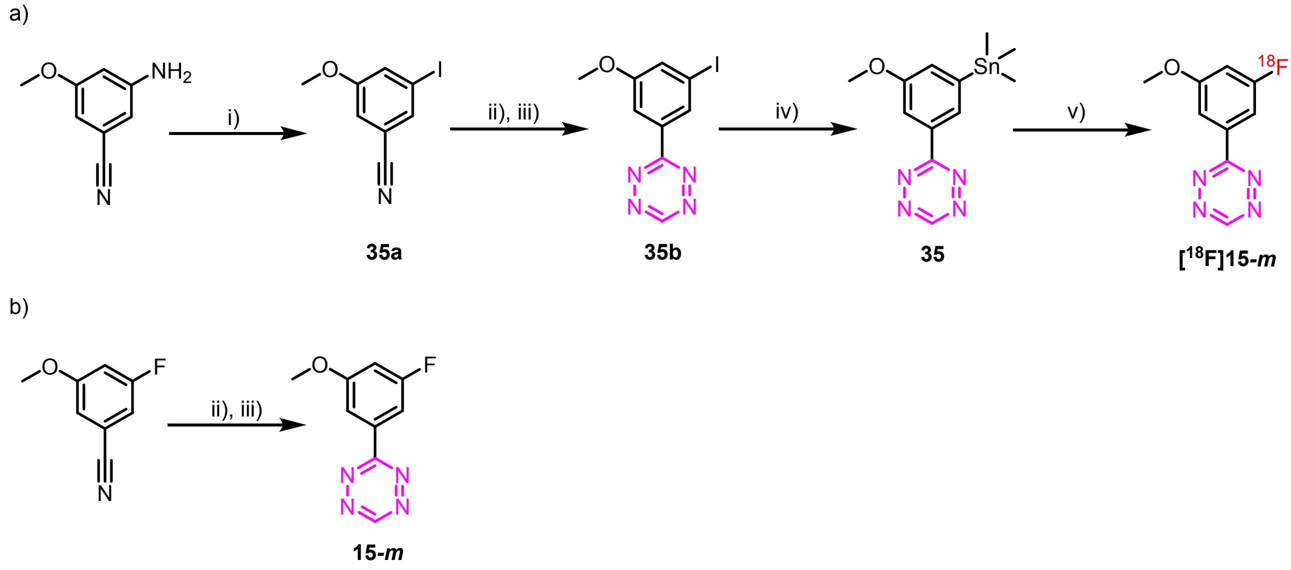

Scheme S15. *Synthesis of Tzs [^{18}F]**15-m** (a) and **15-m** (b). i) HCl, $NaNO_2$; KI, 0 °C to reflux, 4 h, 34%; ii) DCM, S_8, $NH_2NH_2 \cdot H_2O$, EtOH, 50 ºC, 24 h; iii) $NaNO_2$, AcOH, 0 ºC, 20 min, 30% (**35b**), 26% (**15-m**); iv) $(Me_3Sn)_2$, $Pd(PPh_3)_4$, THF, 65 ºC, MW, 3 h, 65%; v) $Cu(OTf)_2$, pyridine, [^{18}F]KF, DMA, 5 min, 100 °C, 17% RCC.*

a)

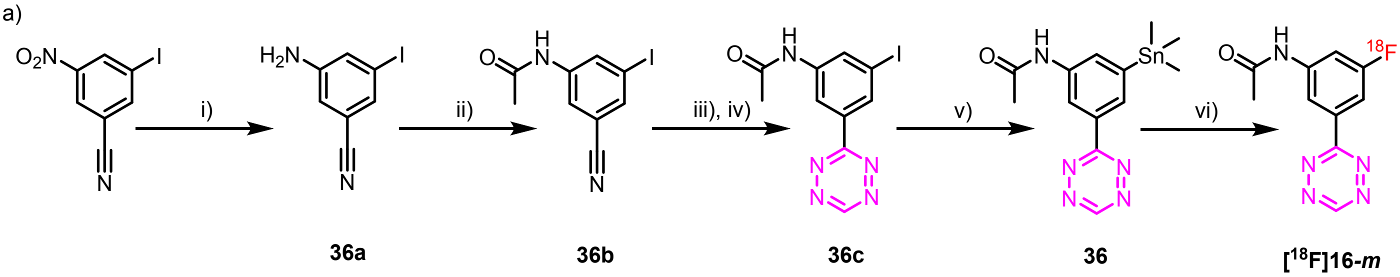

b)

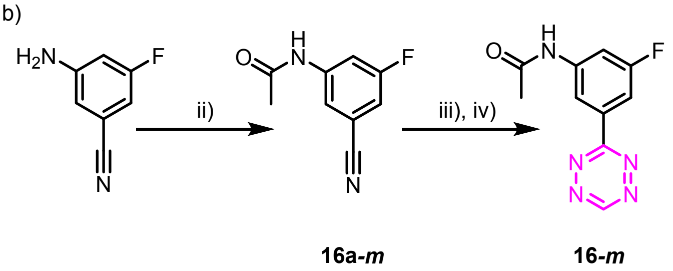

Scheme S16. *Synthesis of Tzs [^{18}F]**16-m** (a) and **16-m** (b). i) Zn, AcOH, MeOH, r.t., 2h, 56%; ii) Acetic anhydride, DCM, r.t., 12 h, 90% (**36b**), 55% (**16a-m**); iii) DCM, S_8, $NH_2NH_2 \cdot H_2O$, EtOH, 50 ºC, 24 h; iv) $NaNO_2$, AcOH, 0 ºC, 20 min, 26% (**36c**), 36% (**16-m**); v) $(Me_3Sn)_2$, $Pd(PPh_3)_4$, THF, 65 ºC, MW, 3 h, 41%; vi) $Cu(OTf)_2$, pyridine, [^{18}F]KF, DMA, 5 min, 100 °C, 31% RCC.*

a)

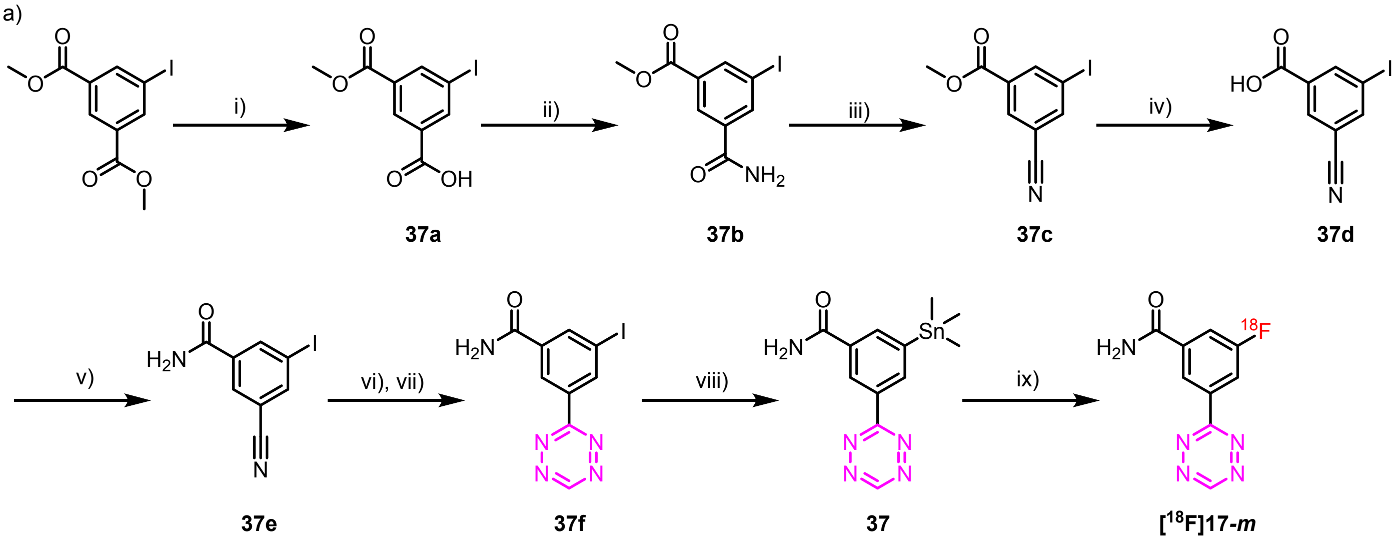

b)

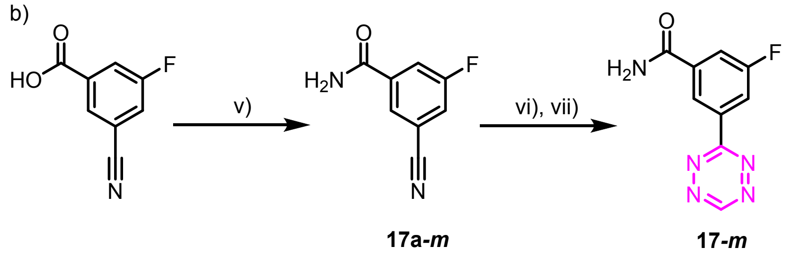

Scheme S17. *Synthesis of Tzs [^{18}F]**17-m** (a) and **17-m** (b). i) NaOH, MeOH, H_2O, r.t., 48 h, 84%; ii) $SOCl_2$, reflux, 2 h; NH_4OH, MeCN, 0 ºC, 1h, 75%; iii) DIPEA, TfOH, DCM, 0 ºC to reflux, 30 min., 91%; iv) LiOH, MeOH, H_2O, reflux, 2 h, 94%; v) CDI, NH_4OH, MeCN, r.t., 1 h,*

*88% (**37e**), 78% (**17a-m**); vi) DCM, S_8, $NH_2NH_2 \cdot H_2O$, EtOH, 50 ºC, 24 h; vii) $NaNO_2$, AcOH, 0 ºC, 20 min, 28% (**37f**), 36% (**17-m**); viii) $(Me_3Sn)_2$, $Pd(PPh_3)_4$, THF, 65 ºC, MW, 3h, 41%; ix) $Cu(OTf)_2$, pyridine, [^{18}F]KF, DMA, 5 min, 100 °C, 24% RCC.*

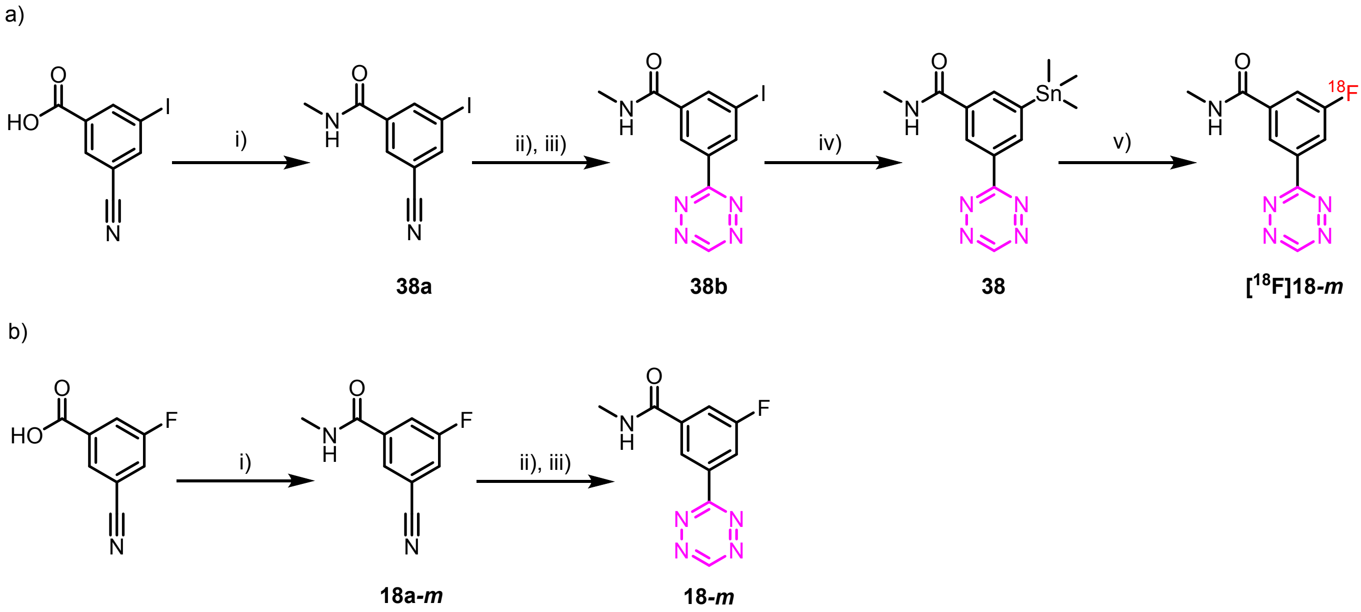

***Scheme S18.** Synthesis of Tzs [^{18}F]**18-m** (a) and **18-m** (b). i) CDI, NH_2CH_3, MeCN, 1h, 98% (**38a**), 76% (**18a-m**); ii) DCM, S_8, $NH_2NH_2 \cdot H_2O$, EtOH, 50 ºC, 24 h; iii) $NaNO_2$, AcOH, 0 ºC, 20 min, 24% (**38b**), 38% (**18-m**); iv) $(Me_3Sn)_2$, $Pd(PPh_3)_4$, THF, 65 ºC, MW, 3 h, 63%; v) $Cu(OTf)_2$, pyridine, [^{18}F]KF, DMA, 5 min, 100 °C, 20% RCC.*

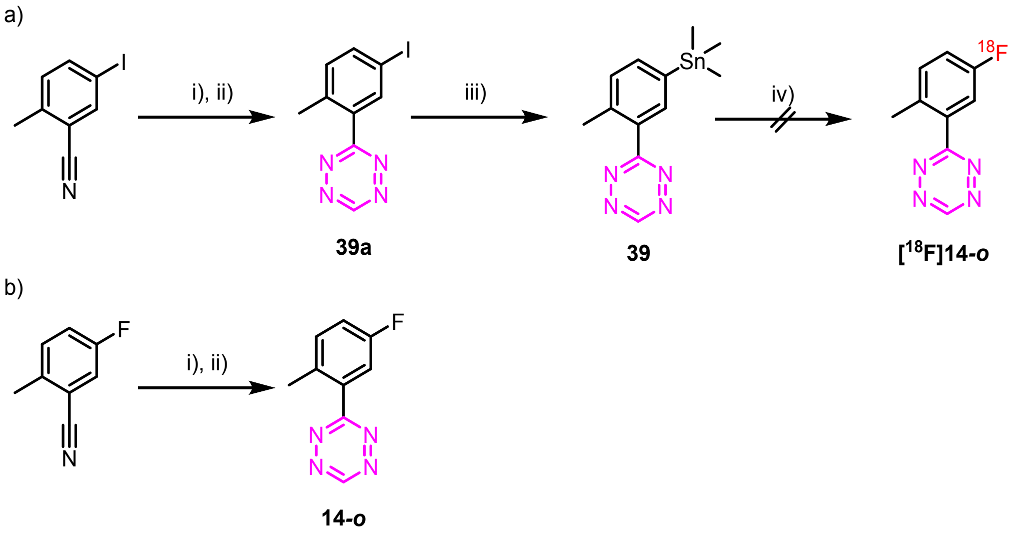

***Scheme S19.** Synthesis of Tzs [^{18}F]**14-o** (a) and **14-o** (b). i) DCM, S_8, $NH_2NH_2 \cdot H_2O$, EtOH, 50 ºC, 24 h; ii) $NaNO_2$, AcOH, 0 ºC, 20 min, 17% (**39a**), 18% (**14-o**); iii) $(Me_3Sn)_2$, $Pd(PPh_3)_4$, THF, 65 ºC, MW, 3 h, 18%; iv) $Cu(OTf)_2$, pyridine, [^{18}F]KF, DMA, 5 min, 100 °C, 0% RCC.*

a)

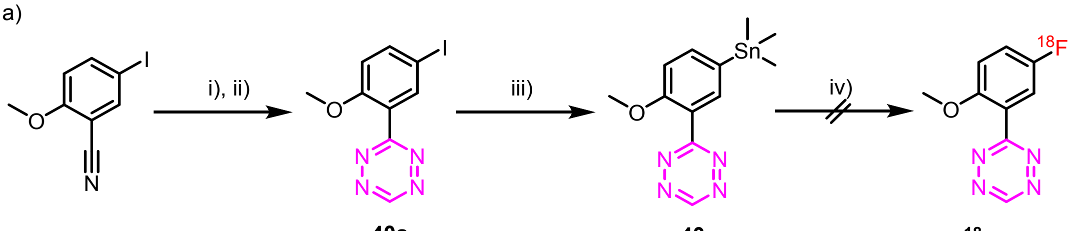

40a **40** **[^{18}F]15-*o***

b)

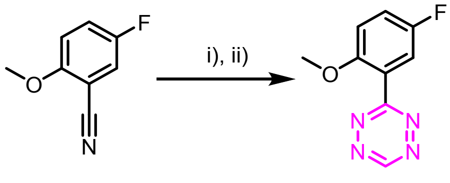

15-*o*

Scheme S20. *Synthesis of Tzs [^{18}F]**15-o** (a) and **15-o** (b). i) DCM, S_8, $NH_2NH_2 \cdot H_2O$, EtOH, 50 ºC, 24 h; ii) $NaNO_2$, AcOH, 0 ºC, 20 min, 15% (**40a**), 16% (**15-o**); iii) $(Me_3Sn)_2$, $Pd(OAc)_2$, ^{me}CgPPh, THF, 70 ºC, MW, 30 min., 40%; iv) $Cu(OTf)_2$, pyridine, [^{18}F]KF, DMA, 5 min., 100 °C, 0% RCC.*

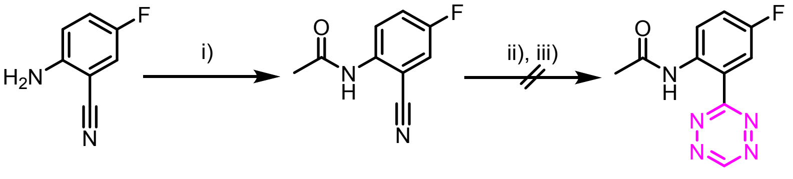

16a-*o* **16-*o***

Scheme S21. *Synthesis of Tz **16-o**. i) Acetic anhydride, DCM, r.t., 12 h, 76%; ii) DCM, S_8, $NH_2NH_2 \cdot H_2O$, EtOH, 50 ºC, 24 h; iii) $NaNO_2$, AcOH, 0 ºC, 20 min., 0%.*

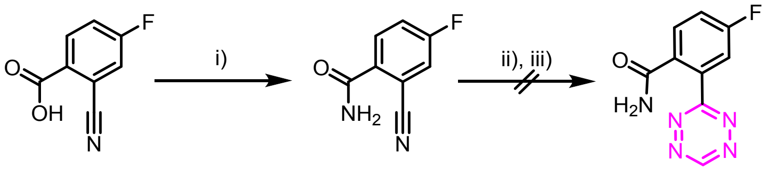

17a-*o* **17-*o***

Scheme S22. *Synthesis of Tz **17-o**. i) CDI, NH_4OH, MeCN, 1h, 71%; ii) DCM, S_8, $NH_2NH_2 \cdot H_2O$, EtOH, 50 ºC, 24 h; iii) $NaNO_2$, AcOH, 0 ºC, 20 min., 0%.*

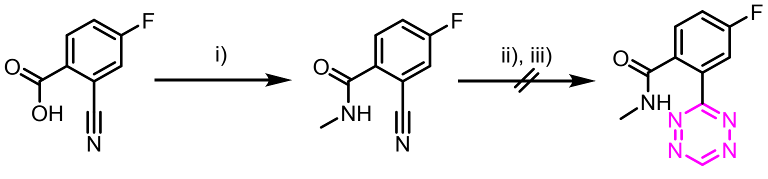

18a-*o* **18-*o***

Scheme S23. *Synthesis of Tz **18-o**. i) CDI, NH_4CH_3, MeCN, 1h, 69%; ii) DCM, S_8, $NH_2NH_2 \cdot H_2O$, EtOH, 50 ºC, 24 h; iii) $NaNO_2$, AcOH, 0 ºC, 20 min., 0%.*

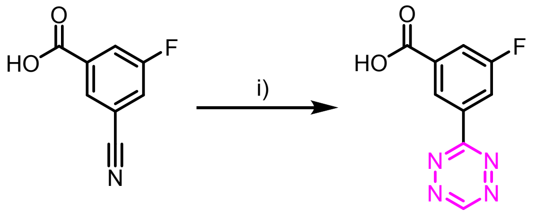

Scheme S24. *Synthesis of Tz **19**. i) DCM, S_8, $NH_2NH_2 \cdot H_2O$, EtOH, 50 ºC, 24 h, 24%.*

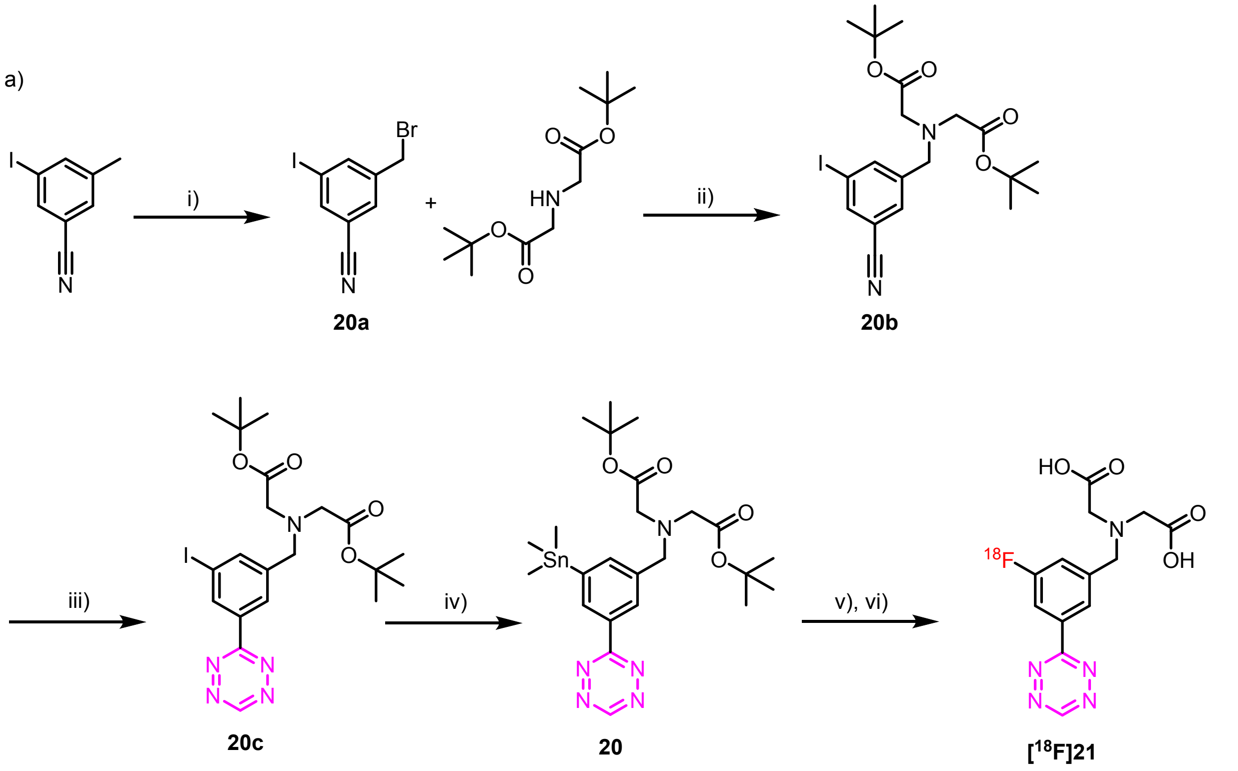

b)

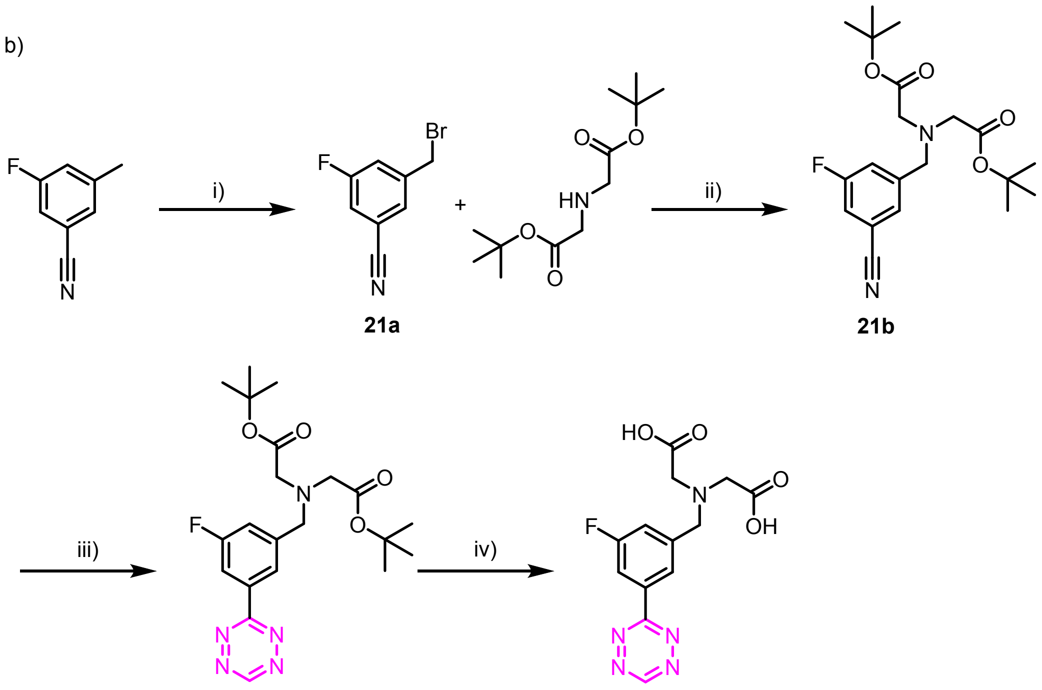

21c 21

Scheme S25. *Synthesis of Tzs [^{18}F]**21** (a) and **21** (b).* ***(a)*** *i) NBS, AIBN, $CHCl_3$, 65 ºC, 24 h; 49%; ii) K_2CO_3, MeCN, 25 ºC, 24 h, 99 %; iii) DCM, S_8, $NH_2NH_2 \cdot H_2O$, EtOH, 50 ºC, 24 h, 15–24%; iv) $(Me_3Sn)_2$, $Pd(PPh_3)_4$, THF, 65 ºC, MW, 3 h, 62%; v) $Cu(OTf)_2$, pyridine, [^{18}F]KF, DMA, 5 min, 100 °C; vi) TFA, MeCN, 100 ºC, 15 min, 11% RCC;* ***(b)*** *i) NBS, AIBN, $CHCl_3$, 65 ºC, 12 h, 52%; ii) K_2CO_3, MeCN, r.t., 24 h, 89%; iii) DCM, S_8, $NH_2NH_2 \cdot H_2O$, EtOH, 50 ºC, 24 h, 17%; iv) TFA, DCM, 25 ºC, 2 h, 51%.*

<u>General Procedure A</u>. Synthesis of 3,6-disubstituted 1,2,4,5-tetrazines.

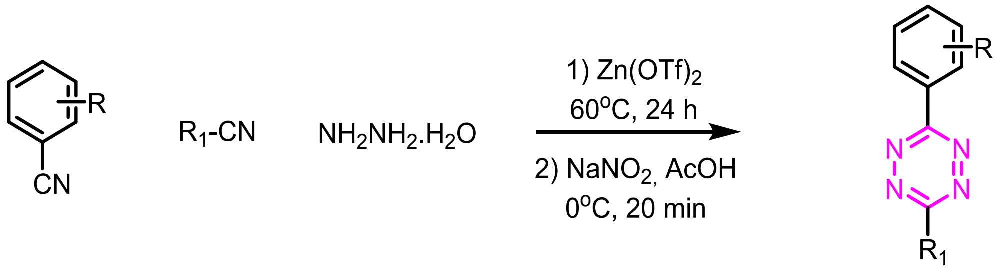

The preparation of these intermediates, were performed using a previously described method.[1] The selected aromatic halogenated nitrile (1 mmol, 1 equiv.), $Zn(OTf)_2$ (182 mg, 0.50 mmol, 0.5 equiv.) and hydrazine monohydrate (2.43 mL, 50 mmol, 50 equiv.), along with the appropriate second nitrile (5 mmol, 5 equiv.) were added to a microwave vial equipped with a stir bar and sealed. The reaction was allowed to stir at 60 °C for 24 hours before being allowed to cool to room temperature and unsealed. $NaNO_2$ (1.35 g, 20 mmol, 20 equiv.) dissolved in water (30 mL) was added to the now yellow mixture followed by dropwise addition of acetic acid (14 mL) producing a red mixture. The reaction mixture was then extracted with EtOAc, washed with brine, dried over $MgSO_4$, filtered and concentrated under reduced pressure. The tetrazine was then purified via automatic flash chromatography utilising various mixtures as the eluent.

3-(4-Fluorophenyl)-6-methyl-1,2,4,5-tetrazine (6)

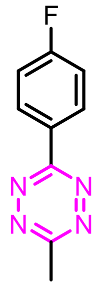

The final compound was obtained from 4-fluorobenzonitrile (121 mg, 1.00 mmol) and MeCN (261 µL, 5.00 mmol) following the *General Procedure A*. The crude was purified using flash chromatography (90/10 n-heptane/EtOAc) to yield 57 mg (30%) of a pink solid. *Rf* = 0.31 (n-heptane:10%EtOAc); ^{1}H NMR (600 MHz, Chloroform-*d*) δ 8.67 – 8.56 (m, 2H), 7.30 – 7.26 (m, 2H), 3.10 (s, 3H).; ^{13}C NMR (151 MHz, Chloroform-*d*) δ 167.4, 165.9 (d, *J* = 254.0 Hz), 163.5, 130.4 (d, *J* = 9.0 Hz), 128.2 (d, *J* = 3.1 Hz), 116.7 (d, *J* = 22.0 Hz), 21.3.; HPLC-MS [M+H]$^{+}$ m/z calc. for $[C_9H_8FN_4]^{+}$: 191.07; Found: 191.20.

3-(4-Iodophenyl)-6-methyl-1,2,4,5-tetrazine (1)

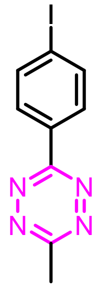

The final compound was obtained from 4-iodobenzonitrile (229 mg, 1.00 mmol) and MeCN (261 µL, 5.00 mmol) following the *General Procedure A*. The crude was purified using flash chromatography (90/10 n-heptane/EtOAc) to yield 85 mg (39%) of a red solid. *Rf* = 0.25 (n-heptane:10%EtOAc); ^{1}H NMR (400 MHz, Chloroform-*d*) δ 8.35 – 8.27 (m, 2H), 8.00 – 7.90 (m, 2H), 3.09 (s, 3H).; ^{13}C NMR (101 MHz, Chloroform-*d*) δ 167.7, 163.9, 138.7, 131.4, 129.4, 100.3, 21.4.

4-(1,2,4,5-tetrazin-3-yl)phenol (4)

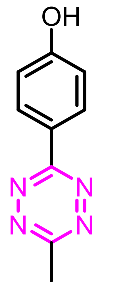

The final compound was obtained from 4-hydroxybenzonitrile (120 mg, 1.00 mmol) and MeCN (261 µL, 5.00 mmol) following the *General Procedure A*. The crude was purified using flash chromatography (90/10 n-heptane/EtOAc) to yield 32 mg (20%) of a red solid. *Rf* = 0.35 (n-heptane:10%EtOAc); ^{1}H NMR (400 MHz, Methanol-d_4) δ 8.42 (d, *J* = 8.8 Hz, 2H), 7.01 (d, *J* = 8.8 Hz, 2H), 3.02 (s, 3H); ^{13}C NMR (101 MHz, MeOD) δ 167.8, 165.2, 163.1, 130.7, 124.3, 117.1, 20.9; HPLC-MS [M+H]$^{+}$ m/z calc. for $[C_9H_9ON_4]^{+}$: 189.08; Found: 189.40.

3-(3-fluorophenyl)-6-methyl-1,2,4,5-tetrazine (7)

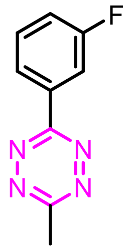

The final compound was obtained from 3-fluorobenzonitrile (120 mg, 1.00 mmol) and MeCN (261 µL, 5.00 mmol) following the *General Procedure A*. The crude was purified using flash chromatography (90/10 n-heptane/EtOAc) to yield 80 mg (42%) of a purple solid. *Rf* = 0.28 (n-heptane:10%EtOAc); ^{1}H NMR (400 MHz, Chloroform-*d*) δ 8.49 – 8.35 (m, 1H), 8.35 – 8.22 (m, 1H), 7.67 – 7.49 (m, 1H), 7.43 – 7.28 (m, 1H), 3.11 (s, 3H); ^{13}C NMR (101 MHz, Chloroform-*d*) δ 167.79, 163.5 (d, *J* = 3.3 Hz), 163.5 (d, *J* = 247.1 Hz), 134.2 (d, *J* = 8.3 Hz), 131.1 (d, *J* = 8.0 Hz), 123.8 (d, *J* = 3.1 Hz), 119.7 (d, *J* = 21.3 Hz), 114.9 (d, *J* = 24.0 Hz), 21.3; HPLC-MS [M+H]$^{+}$ m/z calc. for $[C_9H_8FN_4]^{+}$: 191.07; Found: 191.47.

3-(3-iodophenyl)-6-methyl-1,2,4,5-tetrazine (22a)

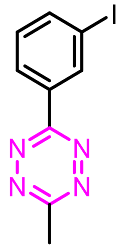

The final compound was obtained from 3-iodobenzonitrile (229 mg, 1.00 mmol) and MeCN (261 µL, 5.00 mmol) following the *General Procedure A*. The crude was purified using flash chromatography (90/10 n-heptane/EtOAc) to yield 88 mg (29%) of a purple solid. *Rf* = 0.26 (n n-heptane:20%EtOAc); ^{1}H NMR (400 MHz, Chloroform-*d*) δ 8.96 (t, *J* = 1.7 Hz, 1H), 8.57 (dt, *J* = 7.9, 1.4 Hz, 1H), 7.96 (ddd, *J* = 7.9, 1.8, 1.1 Hz, 1H), 7.33 (t, *J* = 7.9 Hz, 1H), 3.11 (s, 3H); ^{13}C NMR (101 MHz, Chloroform-*d*) δ 167.8, 163.1, 141.5, 136.8, 133.9, 130.9, 127.2, 94.9, 21.4.

3-(4-fluorophenyl)-6-phenyl-1,2,4,5-tetrazine (8)

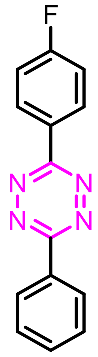

The final compound was obtained from 4-fluorobenzonitrile (121 mg, 1.00 mmol) and benzonitrile (477 µL, 5.00 mmol) following the *General Procedure A*. The final compound was isolated by preparative TLC (60/30 Toluene/n-heptane) to yield a pink solid. *Rf* = 0.65 (Toluene:10%n-heptane); ^{1}H NMR (600 MHz, Chloroform-*d*) δ 8.72 – 8.67 (m, 2H), 8.67 – 8.62 (m, 2H), 7.69 – 7.59 (m, 3H), 7.31 (t, *J* = 8.6 Hz, 2H); ^{13}C NMR (151 MHz, Chloroform-*d*) δ 166.8, 164.3 (d, *J* = 269.7 Hz), 164.1, 132.9, 131.9, 130.5 (d, *J* = 9.0 Hz), 129.5, 128.2, 128.1, 116.8 (d, *J* = 22.0 Hz); HPLC-MS [M+H]$^{+}$ m/z calc. for $[C_{14}H_9FN_4]^{+}$: 253.09; Found: 253.0.

3-(4-iodophenyl)-6-phenyl-1,2,4,5-tetrazine (23a)

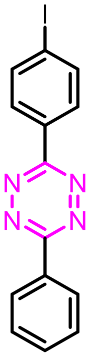

The final compound was obtained from 4-iodobenzonitrile (229 mg, 1.00 mmol) and benzonitrile (477 µL, 5.00 mmol) following the *General Procedure A*. The crude was purified using flash chromatography (90/10 Toluene/n-heptane) to yield 35 mg (10%) of a pink solid. *Rf* = 0.5 (Toluene:10%n-heptane); ^{1}H NMR (600 MHz, Chloroform-*d*) δ 8.68 – 8.63 (m, 2H), 8.40 – 8.35 (m, 2H), 8.00 – 7.95 (m, 2H), 7.68 – 7.59 (m, 3H); ^{13}C NMR (151 MHz, Chloroform-*d*) δ 164.3, 163.8, 138.8, 133.0, 131.8, 131.5, 129.5, 129.4, 128.2, 100.5.

3-(3-fluorophenyl)-6-phenyl-1,2,4,5-tetrazine (9)

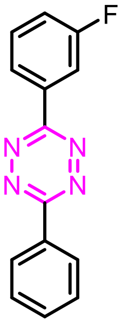

The final compound was obtained from 3-fluorobenzonitrile (107 uL, 1.00 mmol) and benzonitrile (477 µL, 5.00 mmol) following the *General Procedure A*. The final compound was isolated by preparative TLC (60/30 Toluene/n-heptane) to yield a pink solid. *Rf* = 0.65 (Toluene:10%n-heptane); ^{1}H NMR (400 MHz, Chloroform-*d*) δ 8.64 – 8.58 (m, 2H), 8.40 (dt, *J* = 7.8, 1.3 Hz, 1H), 8.33 – 8.26 (m, 1H), 7.62 – 7.50 (m, 4H), 7.28 (tdd, *J* = 8.3, 2.7, 1.0 Hz, 1H); ^{13}C NMR (151 MHz, Chloroform-*d*) δ 164.4, 163.5 (d, *J* = 247.2 Hz), 163.4 (d, *J* = 3.1 Hz), 134.2 (d, *J* = 8.3 Hz), 133.1, 131.8, 131.2 (d, *J* = 8.0 Hz), 129.5, 128.3, 123.8, 119.8 (d, *J* = 21.4 Hz), 115.0 (d, *J* = 24.0 Hz); HPLC-MS $[M+H]^+$ m/z calc. for $[C_{14}H_9FN_4]^+$: 253.09; Found: 253.0.

3-(3-iodophenyl)-6-phenyl-1,2,4,5-tetrazine (24a)

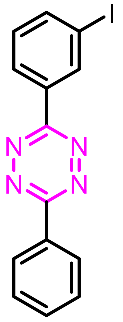

The final compound was obtained from 3-iodobenzonitrile (229 mg, 1.00 mmol) and benzonitrile (477 µL, 5.00 mmol) following the *General Procedure A*. The crude was purified using flash chromatography (90/10 n-heptane/EtOAc) to yield 50 mg (14%) of a pink solid. *Rf* = 0.5 (Toluene:10%n-heptane); ^{1}H NMR (400 MHz, Chloroform-*d*) δ 9.02 (t, *J* = 1.8 Hz, 1H), 8.65 (dd, *J* = 8.0, 1.6 Hz, 2H), 8.62 (d, *J* = 8.0 Hz, 1H), 7.97 (d, *J* = 7.9 Hz, 1H), 7.66 – 7.57 (m, 3H), 7.35 (t, *J* = 7.9 Hz, 1H).; ^{13}C NMR (151 MHz, Chloroform-*d*) δ 164.4, 163.0, 141.6, 136.9, 133.9, 133.1, 131.7, 131.0, 129.5, 128.3, 127.2, 95.0.

3-(4-fluorophenyl)-6-(pyridin-2-yl)-1,2,4,5-tetrazine (10)

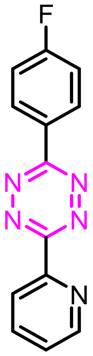

The final compound was obtained from 4-flourbenzonitrile (121 mg, 1.00 mmol) and 2-cyanopyridine (520 mg, 5.00 mmol) following the *General Procedure A*. The crude was purified using flash chromatography (50/50 n-heptane/EtOAc) to yield 40 mg (16%) of a pink solid. *Rf* = 0.5 (n-heptane:50%EtOAC); ^{1}H NMR (600 MHz, Chloroform-*d*) δ 8.92 (ddd, *J* = 4.8, 1.8, 0.9 Hz, 1H), 8.69 – 8.63 (m, 2H), 8.63 (dt, *J* = 7.8, 1.1 Hz, 1H), 7.95 (td, *J* = 7.7, 1.8 Hz, 1H), 7.52 (ddd, *J* = 7.6, 4.8, 1.2 Hz, 1H), 7.28 – 7.19 (m, 2H); ^{13}C NMR (151 MHz, Chloroform-*d*) δ 167.1, 164.6 (d, *J* = 243.4 Hz), 163.5, 151.0, 150.3, 137.7, 131.0 (d, *J* = 9.2 Hz), 127.9 (d, *J* = 3.0 Hz), 126.6, 124.1, 116.8 (d, *J* = 22.1 Hz); HPLC-MS [M+H]$^{+}$ m/z calc. for $[C_{13}H_{8}FN_{5}]^{+}$: 254.08; Found: 254.0.

3-(5-fluoropyridin-2-yl)-6-(pyridin-2-yl)-1,2,4,5-tetrazine (12)

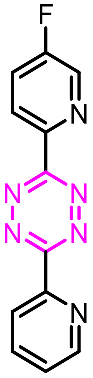

The final compound was obtained from 5-fluoropicolinonitrile (122 mg, 1.00 mmol) and 2-cyanopyridine (520 µL, 5.00 mmol) following the *General Procedure A*. The crude was purified using flash chromatography (50/50 n-heptane/EtOAc) to yield 45 mg (18%) of a pink solid. *Rf* = 0.5 (n-heptane:50%EtOAC); ^{1}H NMR (600 MHz, Chloroform-*d*) δ 9.00 (ddt, *J* = 4.8, 2.1, 1.0 Hz, 1H), 8.85 – 8.83 (m, 1H), 8.83 – 8.80 (m, 1H), 8.76 (dq, *J* = 7.9, 1.1 Hz, 1H), 8.02 (tt, *J* = 7.8, 1.5 Hz, 1H), 7.72 (tdd, *J* = 8.8, 2.9, 1.1 Hz, 1H), 7.59 (ddt, *J* = 7.3, 4.7, 1.2 Hz, 1H).; ^{13}C NMR (151 MHz, Chloroform-*d*) δ 164.0, 163.2, 161.4 (d, *J* = 264.3 Hz), 151.3, 150.2, 146.4 (d, *J* = 4.1 Hz), 140.1 (d, *J* = 24.8 Hz), 137.7, 126.8, 126.2 (d, *J* = 5.5 Hz), 124.7, 124.3 (d, *J* = 18.7 Hz).; HPLC-MS [M+H]$^{+}$ m/z calc. for $[C_{12}H_{8}FN_{6}]^{+}$: 255.08; Found: 255.0.

<u>General Procedure B</u>. *Synthesis of 3,6-disubstituted 1,2-dihydro-1,2,4,5-tetrazines.*

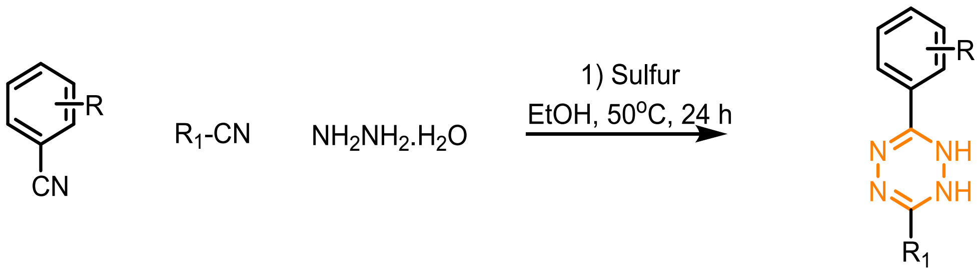

The preparation of this intermediates, was performed using a method described previously.[2] The selected aromatic halogenated nitrile (1 mmol, 1 equiv.), sulfur (513 mg, 2.00 mmol, 2 equiv.), hydrazine monohydrate (804 µL, 16.5 mmol, 16.5 equiv.) and ethanol

(2.0 mL), along with the appropriate second nitrile (4.5 mmol, 4.5 equiv.), were added to a microwave vial equipped with a stir bar and sealed. The reaction mixture was heated to 125 °C for 2 hours before being allowed to cool to room temperature, unsealed and dry under vacuum. The mixture was suspended in 10 mL water and extracted with DCM (2x10 mL), washed with brine, dried over $MgSO_4$, filtered andconcentrated under reduced pressure. The tetrazine was then purified via automatic flash chromatography utilising in various mixtures as the eluent.

3-(4-iodophenyl)-6-(pyridin-2-yl)-1,2-dihydro-1,2,4,5-tetrazine (25a)

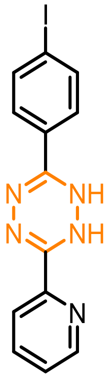

The final compound was obtained from 4-iodobenzonitrile (229 mg, 1.00 mmol) and 2-cyanopyridine (433 µL, 4.5 mmol) following the *General Procedure B*. The crude was purified using flash chromatography (90/10 Toluene/EtOAc) to yield 71 mg (19%) of an orange solid. *Rf* = 0.38 (Toluene/10%EtOAc); ^{1}H NMR (400 MHz, DMSO-d_6) δ 9.31 (s, 1H), 8.74 (s, 1H), 8.63 (d, *J* = 4.9 Hz, 1H), 8.00 – 7.86 (m, 2H), 7.85 – 7.75 (m, 2H), 7.61 (d, *J* = 8.4 Hz, 2H), 7.53 (ddd, *J* = 6.9, 4.8, 1.6 Hz, 1H).; ^{13}C NMR (151 MHz, Chloroform-*d*) δ 148.4, 147.1, 147.1, 138.2, 137.2, 129.8, 127.6, 125.3, 121.5, 97.0.

3-(5-iodopyridin-2-yl)-6-(pyridin-2-yl)-1,2-dihydro-1,2,4,5-tetrazine (26a)[3]

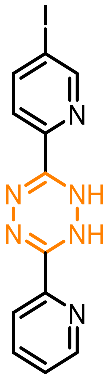

The final compound was obtained from 2-Cyano-5-iodopyridine (231 mg, 1.00 mmol) and 2-Cyanopyridine (433 µL, 4.5 mmol) following the *General Procedure B*. The crude was purified using flash chromatography (95/5 n-heptane/EtOAc) to yield 53 mg (15%) of an orange solid. *Rf* = 0.44 (n-heptane:5%EtOAC); ^{1}H NMR (600 MHz, Chloroform-*d*) δ 8.79 (dd, *J* = 2.1, 0.8 Hz, 1H), 8.61 – 8.54 (m, 2H), 8.40 (s, 1H), 8.08 – 8.02 (m, 2H), 7.83 (dd, *J* = 8.3, 0.9 Hz, 1H), 7.75 (td, *J* = 7.7, 1.7 Hz, 1H), 7.35 (ddd, *J* = 7.5, 4.9, 1.2 Hz, 1H).; ^{13}C NMR (151 MHz, Chloroform-*d*) δ 154.6, 148.5, 147.5, 146.7, 146.6, 146.2, 145.2, 136.9, 125.1, 122.9, 121.5, 95.0.

General Procedure C. Synthesis of 3-substituted-6-H-1,2,4,5-tetrazines.

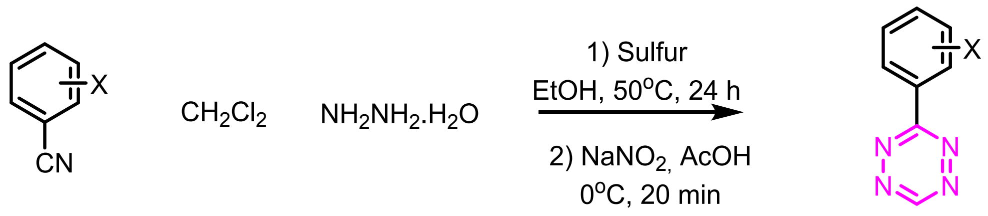

The preparation of this intermediates, was performed using a method described previously.[2] DCM (0.256 mL, 4.00 mmol, 1 equiv.), sulfur (0.257 g, 1.00 mmol, 0.25 equiv.), hydrazine monohydrate (1.6 mL, 32.00 mmol, 8 equiv.) and ethanol (4.0 mL) along with the appropriate nitrile (4 mmol, 1 equiv.) were added to a microwave vial equipped with a stir bar. The vessel was sealed, and the reaction mixture was heated to 50 °C for 24 hours, before being allowed to cool to room temperature and unsealed. Then 3 mL of DCM and $NaNO_2$ (2.8 g, 40.00 mmol, 10 equiv.) in water (40 mL) were added to the now yellow mixture followed by dropwise addition of acetic acid (14 mL), producing a mixture red in colour. The reaction mixture was extracted with DCM, washed with brine, dried with $MgSO_4$ and filtered before concentrating *in vacuo*. The tetrazine was then purified via flash chromatography utilising n-heptane and EtOAc in various mixtures as the eluent and recrystallized in n-heptane.

3-(4-fluorophenyl)-1,2,4,5-tetrazine (11)

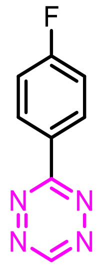

The final compound was obtained from 4-fluorobenzonitrile (242 mg, 4 mmol) following *General Procedure C*. The crude was purified using flash chromatography (90/10 n-heptane/EtOAc) to yield 0.12 g (34%) of a red solid. *Rf* = 0.33 (n-heptane:10%EtOAC); ^{1}H NMR (400 MHz, Chloroform-*d*) δ 10.21 (s, 1H), 8.70 – 8.61 (m, 2H), 7.29 (t, *J* = 8.7 Hz, 2H); ^{13}C NMR (101 MHz, Chloroform-*d*) δ 167.5 (d, *J* = 255.2 Hz), 165.0, 161.8 (d, *J* = 798.2 Hz), 130.9 (d, *J* = 9.2 Hz), 127.9 (d, *J* = 3.2 Hz), 116.8 (d, *J* = 22.1 Hz); HPLC-MS [M+H]$^+$ m/z calc. for $[C_8H_6FN_4]^+$: 177.06; Found: 177.34.

3-(4-iodophenyl)-1,2,4,5-tetrazine (27a)

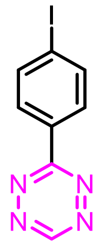

The final compound was obtained from 4-iodobenzonitrile (458 mg, 4 mmol) following *General Procedure C*. The crude was purified using flash chromatography (90/10 n-heptane/EtOAc) to yield 0.16 mg (27%) of a pink solid. *Rf* = 0.37 (n-heptane:10%EtOAC); ^{1}H NMR (400 MHz, Chloroform-*d*) δ 10.24 (s, 1H), 8.39 – 8.32 (m, 2H), 8.02 – 7.95 (m, 2H).; ^{13}C NMR (101 MHz, Chloroform-*d*) δ 166.4, 158.1, 138.9, 131.2, 129.7, 101.2.

3-(3-fluorophenyl)-1,2,4,5-tetrazine (13)

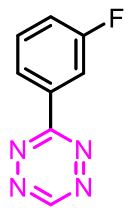

The final compound was obtained from 3-fluorobenzonitrile (242 mg, 4 mmol) following *General Procedure C*. The crude was purified using flash chromatography (90/10 n-heptane/EtOAc) to yield 0.12 g (34%) of a red solid. *Rf* = 0.34 (n-heptane:10%EtOAC); ^{1}H NMR (400 MHz, Chloroform-*d*) δ 10.25 (s, 1H), 8.44 (dt, *J* = 7.8, 1.3 Hz, 1H), 8.33 (ddd, *J* = 9.7, 2.7, 1.6 Hz, 1H), 7.60 (td, *J* = 8.1, 5.7 Hz, 1H), 7.36 (tdd, *J* = 8.3, 2.7, 1.0 Hz, 1H); ^{13}C NMR (101 MHz, Chloroform-*d*) δ 165.9 (d, *J* = 3.3 Hz), 163.5 (d, *J* = 247.6 Hz), 158.2, 133.9 (d, *J* = 8.2 Hz), 131.2 (d, *J* = 8.0 Hz), 124.2 (d, *J* = 3.2 Hz), 120.4 (d, *J* = 21.3 Hz), 115.3 (d, *J* = 24.1 Hz); HPLC-MS [M+H]$^+$ m/z calc. for $[C_8H_6FN_4]^+$: 177.05; Found: 177.54.

3-(3-iodophenyl)-1,2,4,5-tetrazine (28a)

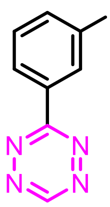

The final compound was obtained from 3-iodobenzonitrile (458 mg, 4 mmol) following *General Procedure C*. The crude was purified using flash chromatography (n-heptane:10%EtOAc) to yield 0.19 g (33%) of a pink solid. *Rf* = 0.36 (n-heptane:10%EtOAC); ^{1}H NMR (400 MHz, Chloroform-*d*) δ 10.25 (s, 1H), 9.00 (t, *J* = 1.7 Hz, 1H), 8.60 (ddd, *J* = 7.9, 1.7, 1.1 Hz, 1H), 7.99 (ddd, *J* = 7.9, 1.8, 1.0 Hz, 1H), 7.35 (t, *J* = 7.9 Hz, 1H); ^{13}C NMR (101 MHz, Chloroform-*d*) δ 165.5, 158.2, 142.1, 137.2, 133.6, 131.1, 127.5, 95.0.

3-(3-fluoro-4-methylphenyl)-1,2,4,5-tetrazine (14-*p*)

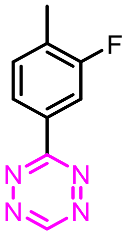

The final compound was obtained from 3-fluoro-4-methylbenzonitrile (0.54 g, 4.00 mmol) following *General Procedure C*. The crude was purified using flash chromatography (95/5 n-heptane/EtOAc) to yield after crystallization with n-heptane 0.21 g (28%) of a red solid. *Rf* = 0.4 (n-heptane:20%EtOAc); ^{1}H NMR (400 MHz, Chloroform-*d*) δ 10.21 (s, 1H), 8.33 (dd, *J* = 8.0, 1.7 Hz, 1H), 8.27 (dd, *J* = 10.5, 1.7 Hz, 1H), 7.43 (t, *J* = 7.8 Hz, 1H), 2.41 (s, 3H); ^{13}C NMR (101 MHz, Chloroform-*d*) δ 165.8, 161.8 (d, *J* = 246.1 Hz), 157.8, 132.5 (d, *J* = 8.3 Hz), 131.1 (d, *J* = 8.3 Hz), 130.9 (d, *J* = 17.4 Hz), 123.8 (d, *J* = 3.5 Hz), 114.7 (d, *J* = 25.1 Hz), 31.9; HPLC-MS [M+H]$^{+}$ m/z calc. for $[C_9H_8FN_4]^+$: 191.07; found 191.11

3-(3-iodo-4-methylphenyl)-1,2,4,5-tetrazine (29a)

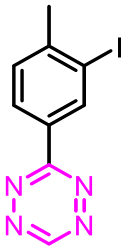

The final compound was obtained from 3-iodo-4-methylbenzonitrile (972 mg, 4.00 mmol) following *General Procedure C*. The crude was purified using flash chromatography (95/5 n-heptane/EtOAc) followed by recrystallization from n-heptane afforded 0.21 g (18%) of a red solid. *Rf* = 0.42 (n-heptane:10%EtOAc); ^{1}H NMR (600 MHz, Chloroform-*d*) δ 10.21 (s, 1H), 9.08 (s, 1H), 8.50 (d, *J* = 9.7 Hz, 1H), 7.46 (d, *J* = 8.0 Hz, 1H), 2.56 (s, 3H); ^{13}C NMR (151 MHz, Chloroform-*d*) δ 165.2, 157.9, 147.2, 138.6, 130.7, 130.5, 127.8, 101.7, 28.5.

3-(3-fluoro-4-methoxyphenyl)-1,2,4,5-tetrazine (15-*p*)

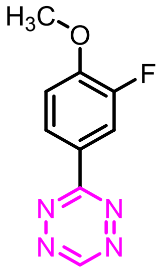

The final compound was obtained from 3-Fluoro-4-methoxylbenzonitrile (0.60 g, 4.00 mmol) following *General Procedure C*. The crude was purified using flash chromatography (95/5 n-heptane/EtOAc) followed by recrystallization from n-heptane afforded 0.24 g (29%) of a red solid. *Rf* = 0.39 (n-heptane:20%EtOAC); ^{1}H NMR (400 MHz, Chloroform-*d*) δ 10.18 (s, 1H), 8.45 (ddd, *J* = 8.6, 2.1, 1.3 Hz, 1H), 8.38 (dd, *J* = 12.1, 2.2 Hz, 1H), 7.18 (t, *J* = 8.5 Hz, 1H), 4.04 (s, 3H); ^{13}C NMR (101 MHz, Chloroform-*d*) δ 165.5, 157.5, 152.7 (d, *J* = 247.7 Hz), 152.1 (d, *J* = 10.7 Hz), 125.3 (d, *J* = 3.6 Hz), 124.4 (d, *J* = 7.2 Hz), 115.8 (d, *J* = 20.8 Hz), 113.5 (d, *J* = 2.2 Hz), 56.4; HPLC-MS $[M+H]^+$ m/z calc. for $[C_9H_8FN_4O]^+$: 207.06; found 207.08.

4-hydroxy-3-iodobenzonitrile (30a)

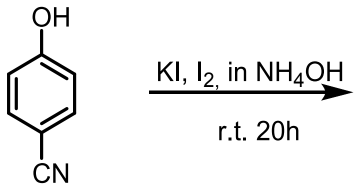

The preparation of this intermediates, was performed using a previously described method.[4] To a solution of 4-hydroxybenzonitrile (4 g, 33.6 mmol) in 25% NH_4OH (180 mL) was added a mixture of KI (27.31 g, 167.9 mmol), I_2 (9.38 g, 36.9 mmol) in H_2O (40 mL). The reaction was allowed to stir at r.t. for 20 hours, in which time the mixture colour gradually turned from black to a white thick suspension. The precipitate formed was filtered off and the filtrate concentrated. The residue was then dissolved in DCM and washed with H_2O, saturated aqueous $Na_2S_2O_3$ solution, and brine. The organic layer was dried over anhydrous $MgSO_4$, filtered and concentratedunder reduced pressure. The crude was purified using flash chromatography (90/10 n-heptane/EtOAc) to yield the product (6.52 g, 79%) as a brown solid. ^{1}H NMR (400 Hz, Chloroform-*d*) δ 7.97 (d, *J* = 1.9 Hz, 1H), 7.55 (dd, *J* = 8.5, 1.95 Hz, 1H), 7.05 (d, *J* = 8.5 Hz, 1H), 5.87 (s, 1H); ^{13}C NMR (600 Hz, Chloroform-*d*) δ 158.7, 142.2, 134.3, 117.3, 116.3, 106.0, 85.5.

3-iodo-4-methoxybenzonitrile (30b)

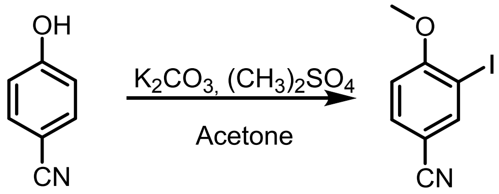

The preparation of this intermediate was performed using a previously described method.[5] 4-Hydroxy-3-iodobenzonitrile (6.52 g, 26.6 mmol) and K_2CO_3 (11.03 g, 79.8 mmol) were suspended in acetone (130 mL) before $(CH_3)_2SO_4$ (5.03 g, 79.8 mmol) was added. Then the flask is fitted with a reflux condenser and the mixture was heated to 70 °C for 90 minutes after which the mixture became a pale yellow thick suspension. After cooling to room temperature, the mixture was filtered, and the filter cake was washed with additional acetone. The solvent was removed on a rotary evaporator and the residue was suspended in water (250 mL) for 90 minutes. The precipitate was filtered off and dried overnight. The crude was purified using flash chromatography (90/10 n-heptane/EtOAc) to yield the product (6.06 g, 88%) as a white solid . ^{1}H NMR (400 Hz, Chloroform-*d*) δ 8.05 (d, *J* = 2.0 Hz, 1H), 7.64 (dd, *J* = 8.6, 2.0 Hz, 1H), 6.85 (d, *J* = 8.6 Hz,1H), 3.95 (s, 3H); ^{13}C NMR (600 Hz, Chloroform-*d*) δ 161.6, 142.8, 134.1, 117.6, 110.7, 105.9, 86.0, 56.7.

3-(3-iodo-4-methoxyphenyl)-1,2,4,5-tetrazine (30c)

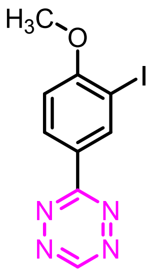

The final compound was obtained from 3-iodo-4-methoxylbenzonitrile (1.03 g, 4.00 mmol) following *General Procedure C*. The crude was purified using flash chromatography (95/5 n-heptane/EtOAc) to yield after recrystallization with n-heptane 0.26 g (20%) as a red

solid. *Rf* = 0.30 (n-heptane:20%EtOAc); ^{1}H NMR (600 MHz, Chloroform-*d*) δ 10.16 (s, 1H), 9.07 (s, 1H), 8.62 (dd, *J* = 8.7, 2.1 Hz, 1H), 7.01 (d, *J* = 8.7 Hz, 1H), 4.01 (s, 3H); ^{13}C NMR (151 MHz, Chloroform-*d*) δ 165.2, 162.2, 157.7, 139.7, 130.3, 125.8, 111.2, 86.9, 56.9.

N-(4-cyano-2-fluorophenyl)acetamide (16a-*p*)

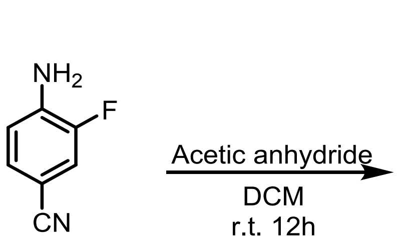

The preparation of this intermediate was performed using a previously described method.[6] To a solution of 4-amino-3-fluorobenzonitrile (0.82 g, 6.00 mmol) in DCM (30.0 mL) was added acetic anhydride (0.80 mL, 8.40 mmol). The mixture was stirred at room temperature for 12 hours. The suspension was filtered, and the solvent removed under reduced pressure. Purification by flash chromatography (70/30 n-heptane/EtOAc) afforded 0.90 g (92%) of N-(4-cyano-2-fluorophenyl)acetamide as a white solid. *Rf* = 0.27 (n-heptane:40%EtOAC); ^{1}H NMR (400 MHz, DMSO-d_6) δ 10.12 (s, 1H), 8.28 (t, *J* = 8.2 Hz, 1H), 7.88 (dd, *J* = 11.1, 1.9 Hz, 1H), 7.65 (dt, *J* = 8.5, 1.3 Hz, 1H), 2.15 (s, 3H); ^{13}C NMR (101 MHz, DMSO-d_6) δ 169.9, 152.1 (d, *J* = 247.2 Hz), 132.1 (d, *J* = 11.2 Hz), 129.8 (d, *J* = 3.6 Hz), 123.3 (d, *J* = 2.9 Hz), 119.8 (d, *J* = 23.4 Hz), 118.4 (d, *J* = 2.7 Hz), 106.2 (d, *J* = 9.4 Hz), 24.3.

N-(2-Fluoro-4-(1,2,4,5-tetrazin-3-yl)phenyl)acetamide (16-*p*)

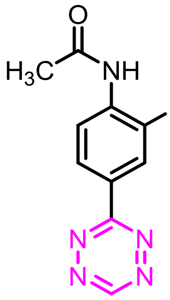

The final compound was obtained from N-(4-cyano-2-fluorophenyl)acetamide (0.71 g, 4.00 mmol) following *General Procedure C*. The crude was purified using flash chromatography (60/40 n-heptane/EtOAc) to yield 0.37 g (40%) of **16-*p*** as a red solid. *Rf* = 0.25 (n-heptane:40%EtOAC); ^{1}H NMR (400 MHz, DMSO-d_6) δ 10.58 (s, 1H), 10.09 (s, 1H), 8.45 – 8.21 (m, 3H), 2.18 (s, 3H); ^{13}C NMR (101 MHz, DMSO-d_6) δ 169.8, 164.9 (d, *J* = 3.0 Hz), 158.4, 153.2 (d, *J* = 246.0 Hz), 131.4 (d, *J* = 11.3 Hz), 128.0 (d, *J* = 7.9 Hz), 124.7 (d, *J* = 3.3 Hz), 123.7, 114.8 (d, *J* = 22.1 Hz), 24.3; HPLC-MS $[M+H]^+$ m/z calc. for $[C_{10}H_9FN_5O]^+$: 234.08; found 234.10

N-(4-cyano-2-iodophenyl)acetamide (31a)

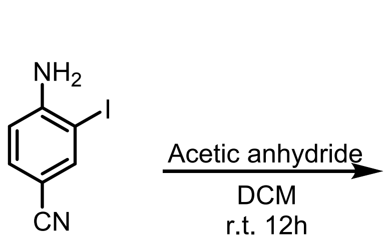

The preparation of this intermediate, was performed using a method described previously.[6] To a solution of the corresponding aniline (1.5 g, 6.00 mmol) in DCM (30.0 mL) was added acetic anhydride (0.85 mL, 9 mmol). The mixture was stirred at room temperature for 12 hours. The suspension was filtered, and the solvent removed under vacuum. Purification by flash chromatography (70/30 n-heptane/EtOAc) afforded 0.90 g (52%) of **31a** as a white solid. *Rf* = 0.5 (n-heptane:40%EtOAc); ^{1}H NMR (600 MHz, DMSO-d_6) δ 9.50 (s, 1H), 8.36 (d, *J* = 1.9 Hz, 1H), 7.82 (dd, *J* = 8.4, 1.9 Hz, 1H), 7.73 (d, *J* = 8.4 Hz, 1H), 2.12 (s, 3H).; ^{13}C NMR (151 MHz, DMSO) δ 168.7, 144.1, 142.4, 132.4, 125.8, 117.3, 108.9, 94.4, 23.5.

N-(2-iodo-4-(1,2,4,5-tetrazin-3-yl)phenyl)acetamide (31b)

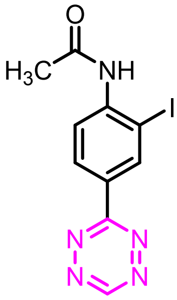

The final compound was obtained from N-(4-cyano-2-iodophenyl)acetamide (1.14 g, 4.00 mmol) following *General Procedure C*. The crude was purified using flash chromatography (60/40 n-heptane/EtOAc) to yield 0.29 g (21%) of **31b** as a pink solid. *Rf* = 0.29 (n-Heptane:40%EtOAc); ^{1}H NMR (600 MHz, Chloroform-*d*) δ 10.20 (s, 1H), 9.07 (s, 1H), 8.62 (d, *J* = 8.7 Hz, 1H), 8.58 (d, *J* = 8.6 Hz, 1H), 7.71 (s, 1H), 2.32 (s, 2H).; ^{13}C NMR (151 MHz, Chloroform-*d*) δ 168.6, 164.9, 157.9, 142.6, 138.8, 129.6, 128.5, 121.2, 25.3, 1.2; HRMS (MALDI-TOF) calculated for $C_{10}H_9N_5O_1I$ $[M+H]^-$: 341.9846, found: 341.9832.

4-Cyano-2-fluorobenzamide (17a-*p*)

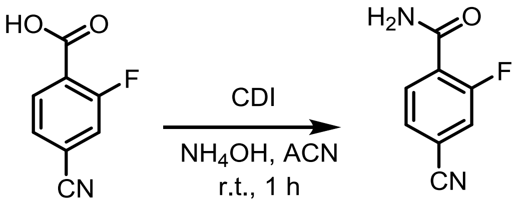

To a solution of 4-cyano-2-fluorobenzoic acid (0.99 g, 6.0 mmol) in MeCN (20 mL) was added CDI (1.46 g, 9.0 mmol). The mixture was stirred at room temperature for 45 minutes, before addition of a 35% aqueous ammonium hydroxide solution (20 mL). The reaction mixture was stirred for 45 minutes and ice-cold water (15 mL) was added. The precipitate was collected by filtration and dried to give the 0.78 g (79%) of **17a-*p*** as a white solid. *Rf* = 0.25 (n-heptane:60%EtOAC); ^{1}H NMR (400 MHz, DMSO-d_6) δ 8.02 – 7.91 (m, 2H), 7.86 (s, 1H), 7.80 – 7.74 (m, 2H); ^{13}C NMR (101 MHz, DMSO-d_6) δ 164.6, 158.9 (d, *J* = 251.4 Hz), 131.6 (d, *J* = 4.0 Hz), 129.6 (d, *J* = 15.7 Hz), 129.2 (d, *J* = 4.0 Hz), 120.8 (d, *J* = 26.7 Hz), 117.7 (d, *J* = 2.8 Hz), 114.6 (d, *J* = 10.0 Hz).

2-Fluoro-4-(1,2,4,5-tetrazin-3-yl)benzamide (17-*p*)

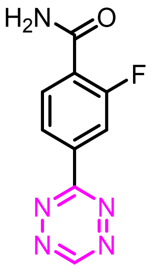

The final compound was obtained from 4-cyano-2-fluorobenzamide (0.78 g, 4.75 mmol) following *General Procedure C*. The crude was purified using flash chromatography (98/2 DCM/MeOH) to yield 0.21 g (20%) of **17-*p*** as a red solid. *Rf* = 0.30 (n-heptane:60%EtOAC); ^{1}H NMR (400 MHz, DMSO-d_6) δ 10.68 (s, 1H), 8.39 (dd, *J* = 8.0, 1.6 Hz, 1H), 8.30 (dd, *J* = 11.1, 1.6 Hz, 1H), 7.97 (s, 1H), 7.93 (t, *J* = 7.7 Hz, 1H), 7.83 (s, 1H); ^{13}C NMR (101 MHz, DMSO-d_6) δ 165.1, 164.8 (d, *J* = 2.9 Hz), 159.9 (d, *J* = 250.1 Hz), 158.8, 136.2 (d, *J* = 8.5 Hz), 131.8 (d, *J* = 3.4 Hz), 128.3 (d, *J* = 15.2 Hz), 124.2 (d, *J* = 3.4 Hz), 115.6 (d, *J* = 25.4 Hz); HPLC-MS $[M+H]^+$ m/z calc. for $[C_9H_7FN_5O]^+$: 220.06; found 226.07.

4-Cyano-2-iodobenzamide (32a)

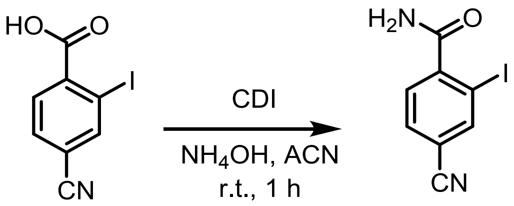

To a solution of 4-cyano-2-iodobenzoic acid (1.00 g, 3.66 mmol) in MeCN (20 mL) was added CDI (0.89 g, 5.49 mmol). The mixture was stirred at room temperature for 45 minutes, before addition of aqueous ammonium hydroxide solution (35%, 20 mL). The reaction mixture was stirred for 45 minutes and ice-cold water (15 mL) was added. The precipitate was collected by filtration and dried to give the 0.89 g (89%) of **32a** as a white solid. *Rf* = 0.23 (n-heptane:60%EtOAC); ^{1}H NMR (400 MHz, DMSO-d_6) δ 8.37 (d, *J* = 1.5 Hz, 1H), 7.98 (s, 1H), 7.91 (dd, *J* = 7.9, 1.6 Hz, 1H), 7.72 (s, 1H), 7.48 (d, *J* = 7.8 Hz, 1H); ^{13}C NMR (101 MHz, DMSO-d_6) δ 170.1, 148.1, 142.5, 132.3, 128.4, 117.4, 113.4, 94.1.

2-Iodo-4-(1,2,4,5-tetrazin-3-yl)benzamide (32b)

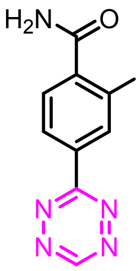

The final compound was obtained from 4-cyano-2-iodobenzamide (0.50 g, 1.83 mmol) following *General Procedure C*. The crude was purified using flash chromatography (98/2 DCM/MeOH) to yield 0.25 g (41%) of **32b** as a red solid. *Rf* = 0.32 (n-heptane:60%EtOAC); ^{1}H NMR (400 MHz, DMSO-d_6) δ 10.66 (s, 1H), 8.89 (d, *J* = 1.6 Hz, 1H), 8.52 (dd, *J* = 8.0, 1.7 Hz, 1H), 8.02 (s, 1H), 7.71 (s, 1H), 7.63 (d, *J* = 8.0 Hz, 1H); ^{13}C NMR (101 MHz, DMSO-d_6) δ 170.6, 164.6, 158.8, 147.2, 138.5, 134.2, 129.0, 127.7, 94.4; HRMS (MALDI-TOF) calculated for $C_9H_7N_5OI$ $[M+H]^-$: 327.9690, found: 327.9676.

4-Cyano-2-fluoro-N-methylbenzamide (18a-*p*)

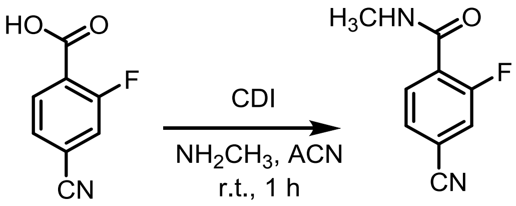

To a solution of 4-cyano-2-fluorobenzoic acid (0.99 g, 6.0 mmol) in MeCN (20 mL) was added 1,1'-carbonyldiimidazole (1.46 g, 9.0 mmol). The mixture was stirred at room temperature for 45 minutes, before addition of aqueous methylamine solution (40%, 20 mL).The reaction mixture was stirred for 45 minutes and ice cold water (15 mL) was added. The precipitate was collected by filtration and dried to give the 0.86 g (80%) of **18a-*p*** as a white solid. *Rf* = 0.29 (n-heptane:60%EtOAC); ^{1}H NMR (400 MHz, DMSO-d_6) δ 8.50 (d, *J* = 6.3 Hz, 1H), 8.02 – 7.92 (m, 1H), 7.81 – 7.71 (m, 2H), 2.79 (d, *J* = 4.6 Hz, 3H); ^{13}C NMR (101 MHz, DMSO-d_6) δ 163.3, 158.8 (d, *J* = 251.2 Hz), 131.6 (d, *J* = 3.8 Hz), 129.5 (d, *J* = 15.6 Hz), 129.2 (d, *J* = 3.9 Hz), 120.8 (d, *J* = 26.6 Hz), 117.7 (d, *J* = 2.9 Hz), 114.6 (d, *J* = 10.1 Hz), 26.7.

2-Fluoro-N-methyl-4-(1,2,4,5-tetrazin-3-yl)benzamide (18-*p*)

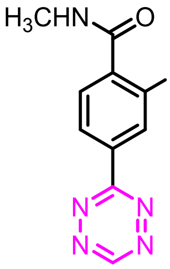

The final compound was obtained from 4-cyano-2-fluoro-N-methylbenzamide (0.77 g, 4.32 mmol) following *General Procedure C*. The crude was purified using flash chromatography (98/2 DCM/MeOH) to yield 0.36 g (36%) of **18-*p*** as a red solid. *Rf* = 0.35 (n-heptane:60%EtOAC); ^{1}H NMR (400 MHz, DMSO-d_6) δ 10.68 (s, 1H), 8.50 (d, *J* = 5.3 Hz, 1H), 8.40 (dd, *J* = 8.1, 1.6 Hz, 1H), 8.30 (dd, *J* = 11.1, 1.6 Hz, 1H), 7.91 (t, *J* = 7.7 Hz, 1H), 2.83 (d, *J* = 4.6 Hz, 3H); ^{13}C NMR (101 MHz, DMSO-d_6) δ 164.8 (d, *J* = 2.9 Hz), 163.8, 159.8 (d, *J* = 249.8 Hz), 158.8, 136.1 (d, *J* = 8.5 Hz), 131.8 (d, *J* = 3.5 Hz), 128.3 (d, *J* = 15.4 Hz), 124.2 (d, *J* = 3.3 Hz), 115.6 (d, *J* = 25.4 Hz), 26.8; HPLC-MS [M+H]$^+$ m/z calc. for $[C_{10}H_9FN_5O]^+$: 234.08; found 234.10; HRMS (MALDI-TOF) calculated for $C_{10}H_9N_5OF$ [M+H]$^-$: 234.0785, found: 234.0779.

4-Cyano-2-iodo-N-methylbenzamide (33a)

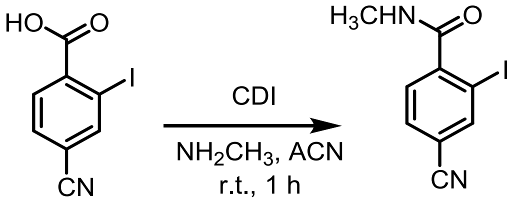

To a solution of 4-cyano-2-iodobenzoic acid (1.00 g, 3.66 mmol) in MeCN (20 mL) was added CDI (0.89 g, 5.49 mmol). The mixture was stirred at room temperature for 45 minutes, before addition of a 40% aqueous methylamine solution (20 mL). The reaction mixture was stirred for 45 minutes and ice-cold water (15 mL) was added. The precipitate was collected by filtration and dried to give the 0.85 g (81%) of **33a** as a white solid. *Rf* = 0.31 (n-heptane:60%EtOAC); ^{1}H NMR (400 MHz, DMSO-d_6) δ 8.44 (d, *J* = 4.8 Hz, 1H), 8.38 (d, *J* = 1.5 Hz, 1H), 7.91 (dd, *J* = 7.8, 1.6 Hz, 1H), 7.47 (d, *J* = 7.8 Hz, 1H), 2.76 (d, *J* = 4.6 Hz, 3H); ^{13}C NMR (101 MHz, DMSO-d_6) δ 168.8, 148.1, 142.4, 132.4, 128.7, 117.4, 113.5, 94.6, 26.4; HRMS (MALDI-TOF) calculated for $C_9H_8N_2OI$ [M+H]$^-$: 286.9676, found: 286.9675.

2-Iodo-N-methyl-4-(1,2,4,5-tetrazin-3-yl)benzamide (33b)

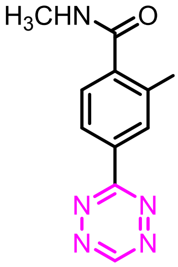

The final compound was obtained from 4-cyano-2-iodo-N-methylbenzamide (0.60 g, 2.09 mmol) following *General Procedure C*. The crude was purified using flash chromatography (98/2 DCM/MeOH) to yield 0.25 g (35%) of **33b** as a red solid. *Rf* = 0.20 (n-heptane:60%EtOAC); ^{1}H NMR (400 MHz, DMSO-d_6) δ 10.66 (s, 1H), 8.89 (d, *J* = 1.6 Hz, 1H), 8.57 – 8.44 (m, 2H), 7.60 (d, *J* = 8.0 Hz, 1H), 2.81 (d, *J* = 4.6 Hz, 3H); ^{13}C NMR (101 MHz, DMSO-d_6) δ 169.2, 164.5, 158.8, 147.2, 138.3, 134.3, 129.2, 127.7, 94.9, 26.5; HRMS (MALDI-TOF) calculated for $C_{10}H_9N_5OI$ $[M+H]^-$: 341.9846, found: 341.9846.

3-(3-fluoro-5-methylphenyl)-1,2,4,5-tetrazine (14-*m*)

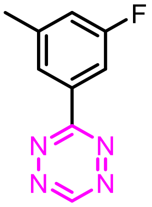

The final compound was obtained from 3-Fluoro-5-methylbenzonitrile (0.54 g, 4.00 mmol) following *General Procedure C*. The crude was purified using flash chromatography (95/5 n-heptane/EtOAc) to yield 0.26 g (34%) of a red oil. *Rf* = 0.39 (n-heptane:20%EtOAc); ^{1}H NMR (400 MHz, Chloroform-*d*) δ 10.16 (s, 1H), 8.19 (d, *J* = 1.4 Hz, 1H), 8.05 (d, *J* = 9.4 Hz, 1H), 7.10 (d, *J* = 9.2 Hz, 1H), 2.43 (s, 3H); ^{13}C NMR (101 MHz, Chloroform-*d*) δ 165.8 (d, *J* = 3.4 Hz), 163.3 (d, *J* = 246.8 Hz), 157.9, 141.8 (d, *J* = 7.7 Hz), 133.3 (d, *J* = 8.9 Hz), 124.7 (d, *J* = 2.7 Hz), 120.8 (d, *J* = 21.2 Hz), 112.3 (d, *J* = 24.3 Hz), 21.5 (d, *J* = 1.8 Hz); HPLC-MS $[M+H]^+$ m/z calc. for $[C_9H_8FN_4]^+$: 191.07; found 191.08.

3-(3-iodo-5-methylphenyl)-1,2,4,5-tetrazine (34a)

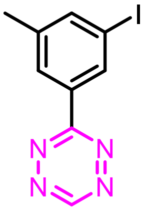

The final compound was obtained from 3-iodo-5-methylbenzonitrile (0.97 g, 4.00 mmol) following *General Procedure C*. The crude was purified using flash chromatography (95/5 n-heptane/EtOAc) to yield after recrystallization with n-heptane 0.27 g (22%) as a red solid. *Rf* = 0.45 (n-heptane:20%EtOAc); ^{1}H NMR (400 MHz, Chloroform-*d*) δ 10.22 (s, 1H), 8.83 – 8.67 (m, 1H), 8.44 – 8.28 (m, 1H), 7.87 – 7.74 (m, 1H), 2.43 (s, 2H); ^{13}C NMR (151 MHz, Chloroform-*d*) δ 165.5, 158.1, 142.7, 141.4, 134.3, 133.3, 128.2, 95.0, 21.2.

3-(3-fluoro-5-methoxyphenyl)-1,2,4,5-tetrazine (15-*m*)

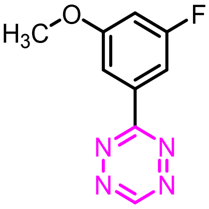

The final compound was obtained from 3-fluoro-5-methoxylbenzonitrile (0.60 g, 4.00 mmol) following *General Procedure C*. The crude was purified using flash chromatography (85/15 n-heptane/EtOAc) and recrystallized from n-heptane to 0.21 g (26%) of **15-*m*** as a red solid. *Rf* = 0.41 (n-heptane:20%EtOAc); ^{1}H NMR (400 MHz, Chloroform-*d*) δ 10.17 (s, 1H), 7.91 (d, *J* = 2.0 Hz, 1H), 7.87 (ddd, *J* = 9.1, 2.4, 1.4 Hz, 1H), 6.83 (dd, *J* = 10.1, 2.4 Hz, 1H), 3.86 (s, 3H); ^{13}C NMR (101 MHz, Chloroform-*d*) δ 165.6 (d, *J* = 3.9 Hz), 164.1 (d, *J* = 246.5 Hz), 161.8 (d, *J* = 11.4 Hz), 158.0, 133.9 (d, *J* = 10.7 Hz), 109.0 (d, *J* = 2.8 Hz), 107.7 (d, *J* = 24.7 Hz), 107.0 (d, *J* = 24.9 Hz), 56.0; HPLC-MS $[M+H]^+$ m/z calc. for $[C_9H_8FN_4O]^+$: 207.07; found 207.05.

3-Iodo-5-methoxybenzonitrile (35a)

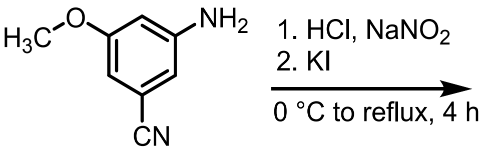

Concentrated HCl (3 mL) was added to a solution of aniline (1.00 g, 6.75 mmol) in water (3 mL) at 0 °C. To this was added a chilled solution of sodium nitrite (0.84 g, 12.15 mmol) in water (4 mL), dropwise, with vigorous mechanical stirring. Stirring was continued at 0 °C for 15 min. after the addition was complete, and then a solution of potassium iodide (2.24 g, 13.50 mmol) in water (4 mL) was added carefully. The cooling bath was removed, and the reaction heated to reflux. When the production of purple vapor ceased, the mixture was cooled to rt and extracted with DCM (3 x 20 mL). The combined organic extracts were dried ($MgSO_4$), filtered, and concentrated under reduced pressure. Purification by flash chromatography (90/10 Heptane/EtOAc) afforded 0.60 g (34%) of **35a** as a white solid. *Rf* = 0.34 (n-heptane:20%EtOAc); ^{1}H NMR (400 MHz, Chloroform-*d*) δ 7.48 (t, *J* = 1.4 Hz, 1H), 7.40 (dd, *J* = 2.4, 1.4 Hz, 1H), 7.04 (dd, *J* = 2.4, 1.4 Hz, 1H), 3.75 (s, 3H); ^{13}C NMR (101 MHz, Chloroform-*d*) δ 159.9, 132.7, 128.2, 116.9, 114.6, 94.1, 55.8.

3-(3-Iodo-5-methoxyphenyl)-1,2,4,5-tetrazine (35b)

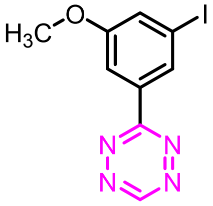

The final compound was obtained from 3-iodo-5-methoxylbenzonitrile (0.52 g, 2.00 mmol) following *General Procedure C*. The crude was purified using flash chromatography (85/15 n-heptane/EtOAc) and recrystallized from n-heptane 0.19 g (30%) of **35b** as a red solid. *Rf* = 0.25 (n-heptane:20%EtOAc); ^{1}H NMR (400 MHz, Chloroform-*d*) δ 10.17 (s, 1H), 8.49 (t, *J* = 1.4 Hz, 1H), 8.03 (dd, *J* = 2.4, 1.4 Hz, 1H), 7.44 (dd, *J* = 2.5, 1.5 Hz, 1H), 3.83 (s, 3H); ^{13}C NMR (101 MHz, Chloroform-*d*) δ 165.1, 160.7, 158.0, 134.1, 129.5, 128.4, 112.5, 95.0, 55.8; HRMS (MALDI-TOF) calculated for $C_9H_8N_4OI$ [M+H]$^-$: 314.9737, found: no ionization detected.

N-(5-Cyano-3-fluorophenyl)acetamide (16a-*m*)

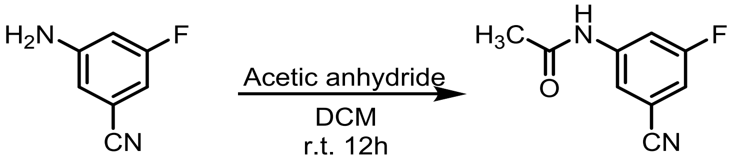

To a solution of 3-amino-5-fluorobenzonitrile (0.82 g, 6.00 mmol) in DCM (30.0 mL) was added acetic anhydride (0.80 mL, 8.40 mmol). The mixture was stirred at room temperature for 12 h. The suspension was filtered, and the solvent removed under vacuum. Purification by flash chromatography (70/30 n-heptane/EtOAc) afforded 0.92 g of **16a-*m*** as a white solid. *Rf* = 0.31 (n-heptane:40%EtOAc); ^{1}H NMR (400 MHz, DMSO-d_6) δ 10.45 (s, 1H), 7.86 – 7.70 (m, 2H), 7.57 – 7.37 (m, 1H), 2.09 (s, 3H); ^{13}C NMR (101 MHz, DMSO-d_6) δ 169.7, 162.2 (d, *J* = 244.3 Hz), 142.4 (d, *J* = 11.8 Hz), 118.7, 118.1 (d, *J* = 3.6 Hz), 113.7 (d, *J* = 25.5 Hz), 113.3 (d, *J* = 12.1 Hz), 110.95 (d, *J* = 26.2 Hz), 24.5.

N-(3-Fluoro-5-(1,2,4,5-tetrazin-3-yl)phenyl)acetamide (16-*m*)

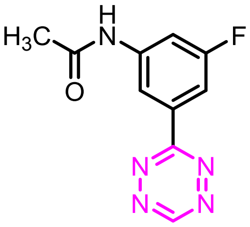

The final compound was obtained from N-(5-cyano-3-fluorophenyl)acetamide (0.58 g, 3.25 mmol) following *General Procedure C*. The crude was purified using flash chromatography (60/40 n-heptane/EtOAc) to yield 0.19 g (25%) of a red solid. *Rf* = 0.25 (n-heptane:40%EtOAc); ^{1}H NMR (400 MHz, DMSO-d_6) δ 10.64 (s, 1H), 10.48 (s, 1H), 8.52 (t, *J* = 1.7 Hz, 1H), 7.98 – 7.81 (m, 2H), 2.12 (s, 3H); ^{13}C NMR (101 MHz, DMSO-d_6) δ 169.5, 165.0 (d, *J* = 3.8 Hz), 163.1 (d, *J* = 242.2 Hz), 158.9, 142.5 (d, *J* = 11.5 Hz), 134.6 (d, *J* = 10.1 Hz), 114.4 (d, *J* = 2.6 Hz), 109.9 (d, *J* = 26.6 Hz), 108.7 (d, *J* = 24.4 Hz), 24.6; HPLC-MS [M+H]$^+$ m/z calc. for $[C_{10}H_9FN_5O]^+$: 234.08; found 234.10.

3-Amino-5-iodobenzonitrile (36a)

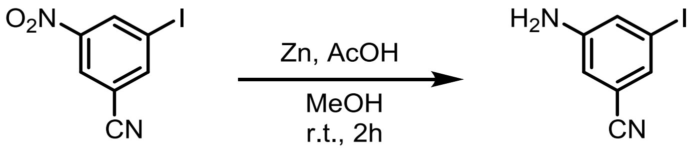

To a solution of 3-iodo-5-nitrobenzonitrile (0.500 g, 1.82 mmol) and Zn (0.58 g, 8.87 mmol) in MeOH (20 mL) was added dropwise 1 mL of acetic acid. The reaction was stirred at room temperature for 2 h and then concentrated under reduced pressure. Purification by flash chromatography afforded 0.250 g (56%) of **36a** as a white solid. *Rf* = 0.22 (n-heptane:20%EtOAc); ^{1}H NMR (400 MHz, Chloroform-*d*) δ 7.24 (d, *J* = 1.4 Hz, 1H), 7.15 (t, *J* = 1.9 Hz, 1H), 6.81 – 6.73 (m, 1H), 3.81 (s, 2H); ^{13}C NMR (101 MHz, Chloroform-*d*) δ 147.7, 129.9, 127.6, 117.4, 116.8, 114.5, 94.5.

N-(5-Cyano-3-iodophenyl)acetamide (36b)

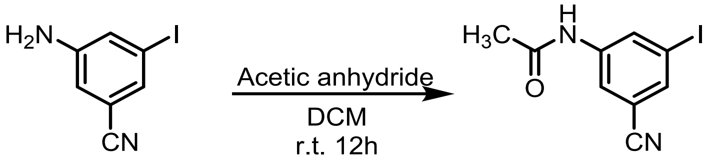

To a solution of 3-amino-5-iodobenzonitrile (0.20 g, 0.81 mmol) in DCM (10.0 mL) was added acetic anhydride (0.1 mL, 1.15 mmol). The mixture was stirred at room temperature for 12 h. The suspension was filtered, and the solvent removed under vacuum. Purification by flash chromatography (70/30 n-heptane/EtOAc) afforded 0.21 (90%) of **36b** as a white solid. *Rf* = 0.29 (n-heptane:40%EtOAc); ^{1}H NMR (400 MHz, Methanol-d_4) δ 8.13 (t, *J* = 1.8 Hz, 1H), 7.87 (t, *J* = 1.7 Hz, 1H), 7.66 (t, *J* = 1.5 Hz, 1H), 2.04 (s, 3H); ^{13}C NMR (101 MHz, Methanol-d_4) δ 170.5, 140.5, 134.8, 132.2, 121.4, 116.5, 113.8, 93.2, 22.5; HRMS (MALDI-TOF) calculated for $C_9H_8N_2OI$ [M+H]$^-$: 286.9676, found: 286.9675.

N-(3-Iodo-5-(1,2,4,5-tetrazin-3-yl)phenyl)acetamide (36c)

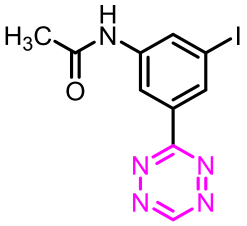

The final compound was obtained from N-(5-cyano-3-iodophenyl)acetamide (0.18 g, 0.63 mmol) following *General Procedure C*. The crude was purified using flash chromatography (60/40 n-heptane/EtOAc) to yield 0.055 g (26%) of **36c** as a red solid. *Rf* = 0.21 (n-heptane:40%EtOAc); ^{1}H NMR (600 MHz, DMSO-d_6) δ 10.63 (s, 1H), 10.35 (s, 1H), 8.73 (t, *J* = 1.7 Hz, 1H), 8.44 (t, *J* = 1.5 Hz, 1H), 8.38 (t, *J* = 1.8 Hz, 1H), 2.11 (s, 3H); ^{13}C NMR (151 MHz, DMSO-d_6) δ 169.4, 164.7, 158.8, 141.9, 134.7, 131.0, 130.6, 117.6, 95.9, 24.6; HRMS (MALDI-TOF) calculated for $C_{10}H_9N_5OI$ [M+H]$^-$: 341.9846, found: 341.9848.

3-Cyano-5-fluorobenzamide (17a-*m*)

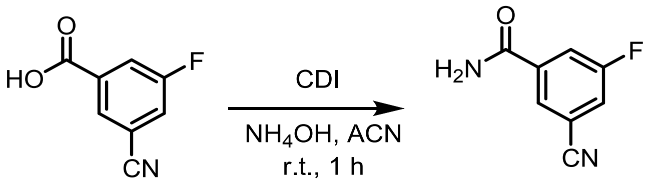

To a solution of 5-cyano-3-fluorobenzoic acid (0.99 g, 6.0 mmol) in MeCN (20 mL) was added 1,1'-carbonyldiimidazole (1.46 g, 9.0 mmol). The mixture was stirred at room temperature for 45 min, before addition of a 35% aqueous ammonium hydroxide solution (20 mL). The reaction mixture was stirred for 45 min and ice-cold water (15 mL) was added. The precipitate was collected by filtration and dried to give the title compound 0.77 g (78%) of **17a-*m*** as a white solid. *Rf* = 0.41 (n-heptane:40%EtOAc); ^{1}H NMR (400 MHz, DMSO-d_6) δ 8.21 (s, 1H), 8.16 (d, *J* = 1.5 Hz, 1H), 8.05 (ddd, *J* = 8.4, 2.6, 1.3 Hz, 1H), 8.01 (ddd, *J* = 9.6, 2.5, 1.4 Hz, 1H), 7.78 (s, 1H); ^{13}C NMR (101 MHz, DMSO-d_6) δ 165.1 (d, *J* = 2.4 Hz), 162.0 (d, *J* = 247.5 Hz), 138.4 (d, *J* = 7.3 Hz), 128.1 (d, *J* = 3.1 Hz), 122.4 (d, *J* = 25.7 Hz), 120.2 (d, *J* = 22.9 Hz), 117.7 (d, *J* = 3.1 Hz), 113.52 (d, *J* = 9.9 Hz).

3-Fluoro-5-(1,2,4,5-tetrazin-3-yl)benzamide (17-*m*)

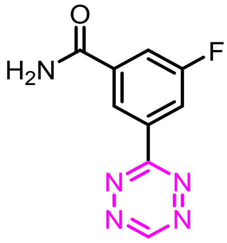

The final compound was obtained from 3-cyano-5-fluorobenzamide (0.75 g, 4.57 mmol) following *General Procedure C*. The crude was purified using flash chromatography (98/2 DCM/MeOH) to yield o yield 0.36 g (36%) of **17-*m*** as a pink solid. *Rf* = 0.31 (n-heptane:60%EtOAC); ^{1}H NMR (400 MHz, DMSO-d_6) δ 10.69 (s, 1H), 8.88 (s, 1H), 8.48 – 8.20 (m, 2H), 8.16 – 7.92 (m, 1H), 7.71 (s, 1H); ^{13}C NMR (101 MHz, DMSO-d_6) δ 166.1 (d, *J* = 2.3 Hz), 164.9 (d, *J* = 3.3 Hz), 162.9 (d, *J* = 245.6 Hz), 158.9, 138.3 (d, *J* = 6.9 Hz), 134.9 (d, *J* = 8.2 Hz), 123.6 (d, *J* = 2.9 Hz), 118.9 (d, *J* = 23.0 Hz), 117.3 (d, *J* = 24.1 Hz); HPLC-MS [M+H]$^+$ m/z calc. for $[C_9H_7FN_5O]^+$: 220.06; found 220.09.

3-Iodo-5-(methoxycarbonyl)benzoic acid (37a)

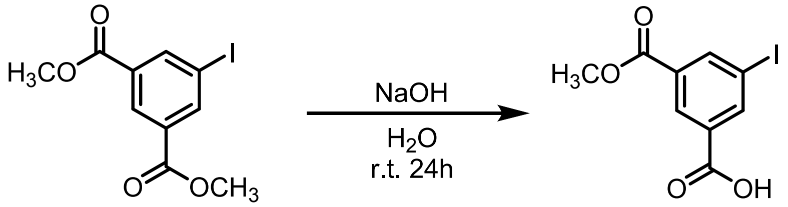

The preparation of this intermediate, was performed using a previously described method.[7] To a solution of dimethyl 5-iodoisophthalate (12.8 g, 40 mmol), methanol (80 mL), and DCM (40 mL) was added NaOH (1.68 g, 42 mmol). The mixture was allowed to stir at room temperature for 24 hours. The solvents were removed under reduced pressure. Lots of white precipitate formed when water (9 mL), dichloromethane (10 mL), and ethyl acetate (10 mL) were added while stirring, which was collected by filtration, well washed with a mixture of dichloromethane (10 mL) and ethyl acetate (10 mL), and then with water (10 mL). After transferring the solid (mono sodium salt) to a separatory funnel, ethyl acetate (80 mL) and concentrated HCl (3 mL) diluted with water (20 mL) were successively added. The mixture was vigorously shaken until the solid was disappeared. Then the organic layer was separated, and the aqueous layer was extracted by ethyl acetate (25 mL). The organic layers were combined and washed by brine (20 mL), dried over MgSO4, filtered, and concentrated. The solid obtained was washed recrystallized from MeOH to give 10.3 g (84%) of **37a** as a white solid. *Rf* = 0.33 (DCM:5%MeOH:0.1%AcOH); ^{1}H NMR (400 MHz, DMSO-d_6) δ 13.57 (s, 1H), 8.49 – 8.27 (m, 3H), 3.89 (s, 3H); ^{13}C NMR (101 MHz, DMSO-d_6) δ 165.6, 164.7, 142.2, 141.6, 133.7, 132.3, 129.7, 95.4, 53.6.

Methyl 3-carbamoyl-5-iodobenzoate (37b)

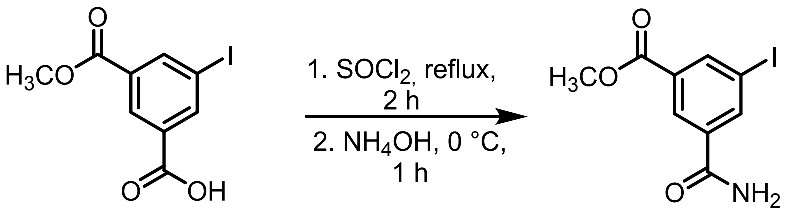

A solution of 3-methoxycarbony-5-iodobenzoic acid (10.3 g, 33.65 mmol) in thionyl chloride (30.0 mL) was heated for 2 hours at 60 °C The reaction mixture was cooled and concentrated under reduced pressure. The intermediate acid chloride was then diluted with tetrahydrofuran (40 mL) and cooled to 0 °C. The mixture was then treated with a solution of 2M ammonia (60 mL, 120 mmol, methanol) and the reaction stirred for 1 hour at 0°C. The mixture was then filtered, and the solvent removed under reduced pressure. Recrystallization from methanol afforded 7.70 g (75%) of **37b** as a white solid. *Rf* = 0.25 (n-heptane:40%EtOAc); ^{1}H NMR (400 MHz, DMSO-d_6) δ 8.47 (t, *J* = 1.6 Hz, 1H), 8.44 (d, *J* = 1.6 Hz, 1H), 8.35 (t, *J* = 1.6 Hz, 1H), 8.24 (s, 1H), 7.61 (s, 1H), 3.89 (s, 3H); ^{13}C NMR (101 MHz, DMSO-d_6) δ 165.7, 165.0, 140.7, 140.2, 137.0, 132.1, 128.1, 95.2, 53.1.

Methyl 3-cyano-5-iodobenzoate (37c)

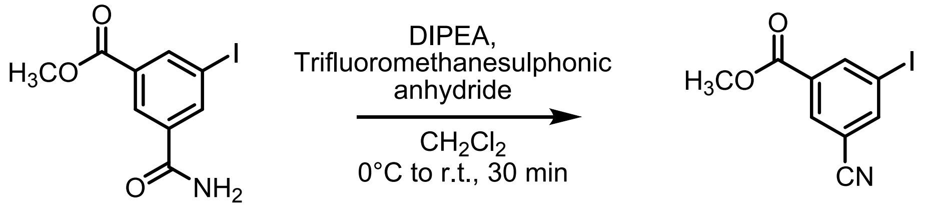

At a temperature of about 0 °C, a solution of 2.8 mL (16.5 mmol) of trifluoromethanesulphonic anhydride in 50 mL of dichloromethane was added dropwise to a solution of 2.80 g (9.18 mmol) of methyl 3-carbamoyl-5-iodobenzoate and 8 mL (45.9 mmol) of N,N-diisopropylethylamine in 150 mL of dichloromethane. After a reaction time of 30 min at 0 °C, 50 mL of saturated aqueous sodium bicarbonate solution were added, and the mixture was stirred vigorously at room temperature for 10 minutes. The organic phase was separated off, dried over anhydrous $MgSO_4$, filtered and freed from the solvent on a rotary evaporator. Purification by flash chromatography (80/20 Heptane/EtOAc) afforded 2.4 g (91%) of **37c** as a white solid. *Rf* = 0.5 (n-heptane:20%EtOAc); ^{1}H NMR (400 MHz, Chloroform-*d*) δ 8.58 (s, 1H), 8.27 (s, 1H), 8.14 (s, 1H), 3.96 (s, 3H); ^{13}C NMR (101 MHz, Chloroform-*d*) δ 163.7, 144.1, 142.6, 132.7, 132.3, 116.3, 114.6, 93.7, 53.0.

3-Cyano-5-iodobenzoic acid (37d)

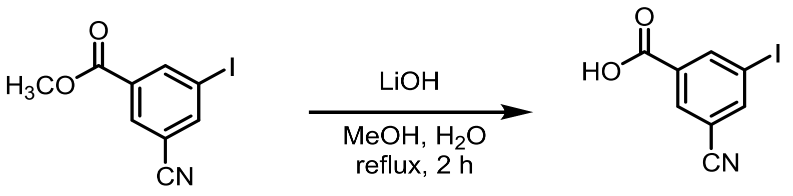

A solution of methyl 3-cyano-5-iodobenzoate (2.36 g, 8.22 mmol) in THF (25 mL) was treated with 0.5M LiOH (20 mL, 9.86 mmol) and methanol. The reaction mixture was heated at reflux for 1 hour. The solvent was concentrated in vacuo and the mixture treated with 1 N HCl. The resulting white precipitate was filtered, and the filtrate was extracted with DCM. The residue and the extracted filtrate were combined and concentrated in vacuo to afford 2.1 g (94%) of **37d** as a white solid. *Rf* = 0.33 (n-heptane:40%EtOAc); ^{1}H NMR (400 MHz, Chloroform-*d*) δ 10.73 (s, 1H), 8.65 (s, 1H), 8.35 (s, 1H), 8.22 (s, 1H); ^{13}C NMR (101 MHz, Chloroform-*d*) δ 168.4, 145.0, 143.1, 132.8, 131.7, 116.0, 114.8, 93.8.

3-Cyano-5-iodobenzamide (37e)

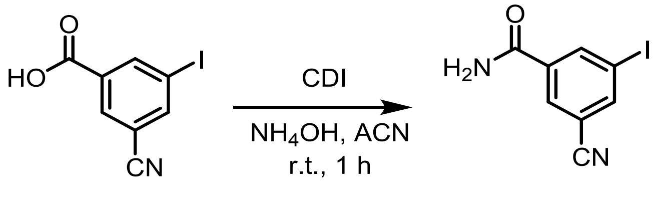

To a solution of 5-cyano-3-iodobenzoic acid (0.40 g, 1.46 mmol) in MeCN (10 mL) was added 1,1'-carbonyldiimidazole (0.36 g, 2.20 mmol). The mixture was stirred at room temperature for 45 min, before addition of a 28% ammonium hydroxide solution (5 mL). The reaction mixture was stirred for 45 min and ice-cold water (15 mL) was added. The precipitate was collected by filtration and dried to give the title compound 0.35 g (88%) of **37e** as a white solid. *Rf* = 0.44 (n-heptane:40%EtOAc); ^{1}H NMR (400 MHz, Methanol-d_4) δ 8.41 (s, 1H), 8.18 (s, 1H), 8.11 (s, 1H); ^{13}C NMR (101 MHz, Methanol-d_4) δ 166.9, 142.9, 140.8, 136.4, 130.2, 116.2, 114.0, 93.36.

3-Iodo-5-(1,2,4,5-tetrazin-3-yl)benzamide (37f)

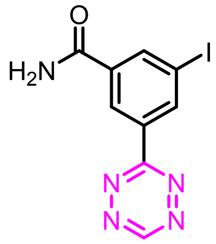

The final compound was obtained from 3-cyano-5-iodobenzamide (0.21 g, 0.77 mmol) following *General Procedure C*. The crude was purified using flash chromatography (98/2 DCM/MeOH) to yield 0.07 g (28%) of **37f** as a pink solid. *Rf* = 0.41 (n-heptane:60%EtOAC); ^{1}H NMR (400 MHz, DMSO-d_6) δ 10.68 (s, 1H), 8.99 (d, *J* = 1.7 Hz, 1H), 8.89 (d, *J* = 1.7 Hz, 1H), 8.55 (d, *J* = 1.7 Hz, 1H), 8.34 (s, 1H), 7.66 (s, 1H); ^{13}C NMR (101 MHz, DMSO-d_6) δ 166.0, 164.6, 158.8, 140.1, 138.8, 137.6, 134.6, 126.8, 96.0; HRMS (MALDI-TOF) calculated for $C_9H_7N_5OI$ [M+H]$^-$: 327.9690, found: 327.9691.

5-Cyano-3-fluoro-N-methylbenzamide (18a-*m*)

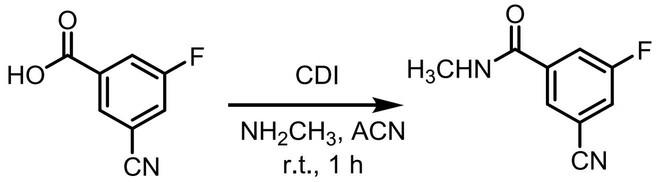

To a solution of 5-cyano-3-fluorobenzoic acid (0.99 g, 6.0 mmol) in MeCN (20 mL) was added CDI (1.46 g, 9.0 mmol). The mixture was stirred at room temperature for 45 min, before addition of a 40% aqueous methylamine solution (20 mL). The reaction mixture was stirred for 45 min and ice-cold water (15 mL) was added. The precipitate was collected by filtration and dried to give the title compound 0.81 g (76%) of **18a-*m*** as a white solid. *Rf* = 0.32 (n-heptane:60%EtOAc); ^{1}H NMR (400 MHz, DMSO-d_6) δ 8.72 (d, *J* = 5.8 Hz, 1H), 8.11 (t, *J* = 1.5 Hz, 1H), 8.05 (ddd, *J* = 8.1, 2.7, 1.4 Hz, 1H), 7.97 (ddd, *J* = 9.5, 2.7, 1.5 Hz, 1H), 2.81 (d, *J* = 4.6 Hz, 3H); ^{13}C NMR (101 MHz, DMSO-d_6) δ 164.0 (d, *J* = 2.5 Hz), 162.0 (d, *J* = 247.5 Hz), 138.5 (d, *J* = 7.4 Hz), 127.8 (d, *J* = 3.3 Hz), 122.2 (d, *J* = 25.6 Hz), 119.9 (d, *J* = 23.1 Hz), 117.7 (d, *J* = 3.4 Hz), 113.6 (d, *J* = 10.0 Hz), 26.8; HRMS (MALDI-TOF) calculated for $C_9H_8N_2OF$ [M+H]$^-$: 179.0615, found: 179.0613.

3-Fluoro-N-methyl-5-(1,2,4,5-tetrazin-3-yl)benzamide (18-*m*)

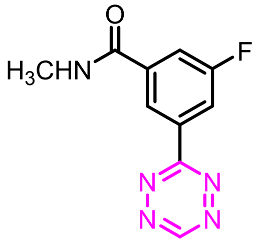

The final compound was obtained from 5-cyano-3-fluoro-N-methylbenzamide (0.62 g, 3.48 mmol) following *General Procedure C*. The crude was purified using flash chromatography (98/2 DCM/MeOH) to afford 0.28 g (34%) of **18-*m*** as a pink solid. *Rf* = 0.31 (n-heptane:60%EtOAC); ^{1}H NMR (400 MHz, DMSO-d_6) δ 10.70 (s, 1H), 8.87 (s, 2H), 8.39 (dd, *J* = 9.3, 1.9 Hz, 1H), 7.99 (dt, *J* = 9.5, 2.0 Hz, 1H), 2.85 (d, *J* = 4.5 Hz, 3H); ^{13}C NMR (101 MHz, DMSO-d_6) δ 164.9 (d, *J* = 11.1 Hz), 162.9 (d, *J* = 245.8 Hz), 158.9, 138.4 (d, *J* = 7.5 Hz),

135.0 (d, J = 8.2 Hz), 133.1, 123.1, 118.6 (d, J = 22.8 Hz), 117.2 (d, J = 24.0 Hz), 27.0; HPLC-MS $[M+H]^+$ m/z calc. for $[C_{10}H_9FN_5O]^+$: 234.08; found 234.06; HRMS (MALDI-TOF) calculated for $C_{10}H_9N_5OF$ $[M+H]^-$: 234.0785, found: 234.0785.

5-Cyano-3-iodo-N-methylbenzamide (38a)

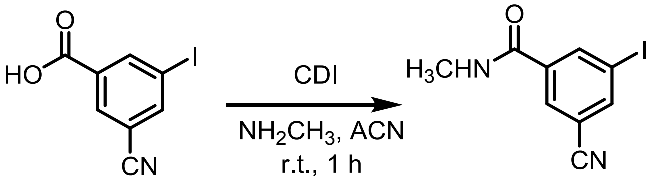

To a solution of 5-cyano-3-iodobenzoic acid (0.40 g, 1.46 mmol) in MeCN (10 mL) was added CDI (0.36 g, 2.20 mmol). The mixture was stirred at room temperature for 45 min, before addition of aqueous Methylamine solution (80%, 5 mL). The reaction mixture was stirred for 45 min and ice-cold water (15 mL) was added. The precipitate was collected by filtration and dried to give the title compound 0.41 g (98%) of **38a** as a white solid. *Rf* = 0.55 (n-heptane:40%EtOAc); ^{1}H NMR (400 MHz, Methanol-d_4) δ 8.45 (d, J = 1.7 Hz, 1H), 8.27 (d, J = 1.9 Hz, 1H), 8.15 (s, 1H), 2.93 (s, 3H); ^{13}C NMR (101 MHz, Methanol-d_4) δ 165.4, 142.6, 140.4, 136.8, 129.8, 116.2, 114.0, 93.4, 25.7; HRMS (MALDI-TOF) calculated for $C_9H_8N_2OI$ $[M+H]^-$: 286.9676, found: 286.9675.

3-Iodo-N-methyl-5-(1,2,4,5-tetrazin-3-yl)benzamide (38b)

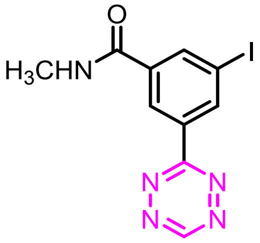

The final compound was obtained from 5-cyano-3-iodo-N-methylbenzamide (0.38 g, 1.32 mmol) following *General Procedure C*. The crude was purified using flash chromatography (98/2 DCM/MeOH) to yield 0.11 g (24%) of **38b** as a pink solid. *Rf* = 0.45 (n-heptane:60%EtOAC); ^{1}H NMR (600 MHz, DMSO-d_6) δ 10.68 (s, 1H), 8.97 (s, 1H), 8.89 (s, 1H), 8.84 (q, J = 4.5 Hz, 1H), 8.51 (s, 1H), 2.83 (d, J = 4.5 Hz, 3H); ^{13}C NMR (151 MHz, DMSO-d_6) δ 164.7, 164.6, 158.9, 139.8, 138.6, 137.6, 134.6, 126.3, 96.0, 26.9; HRMS (MALDI-TOF) calculated for $C_{10}H_8N_5OI$ $[M+H]^-$: 341.9846, found: 341.9846.

3-(3-fluoro-6-methylphenyl)-1,2,4,5-tetrazine (14-*o*)

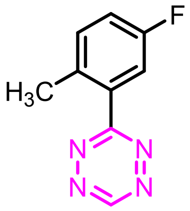

The final compound was obtained from 5-fluoro-2-methylbenzonitrile (0.54 g, 4.00 mmol) following *General Procedure C*. The crude was purified using flash chromatography (95/5 n-heptane/EtOAc) to yield after crystallization with n-heptane 0.12 g (16%) of a red solid. *Rf* = 0.37 (n-heptane:10%EtOAc); ^{1}H NMR (600 MHz, Chloroform-d) δ 10.23 (s, 1H), 7.72 (dd, J = 8.5, 3.2 Hz, 1H), 7.32 – 7.27 (m, 1H), 7.08 (dd, J = 9.1, 4.2 Hz, 1H), 3.90 (s, 3H); ^{13}C NMR (151 MHz, Chloroform-d) δ 167.8 (d, J = 2.2 Hz), 157.1 (d, J = 240.5 Hz), 157.0, 155.0 (d, J = 2.1 Hz), 122.9 (d, J = 7.8 Hz), 120.1 (d, J = 22.9 Hz), 118.5 (d, J = 25.3 Hz), 113.9 (d, J = 7.9 Hz), 57.0; HPLC-MS $[M+H]^+$ m/z calc. for $[C_9H_7FN_4]^+$: 191.07; Found 191.09.

3-(5-iodo-2-methylphenyl)-1,2,4,5-tetrazine (39a)

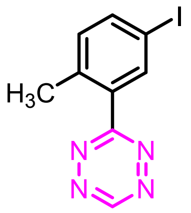

The final compound was obtained from 3-iodo-5-methylbenzonitrile (0.97 g, 4.00 mmol) following *General Procedure C*. The crude was purified using flash chromatography (90/10 n-heptane/EtOAc) to yield after crystallization with n-heptane 0.21 g (17%) of a red solid. *Rf* = 0.42 (n n-heptane:10%EtOAc).; ^{1}H NMR (600 MHz, Chloroform-*d*) δ 10.26 (s, 1H), 8.43 (s, 1H), 7.81 (d, J = 8.1 Hz, 1H), 7.15 (d, J = 8.1 Hz, 1H), 2.59 (s, 3H); ^{13}C NMR (151 MHz, Chloroform-*d*) δ 168.6, 157.1, 140.7, 139.6, 138.6, 133.9, 133.5, 90.9, 21.3.

3-(3-fluoro-6-methoxyphenyl)-1,2,4,5-tetrazine (15-*o*)

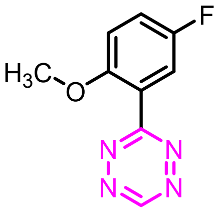

The final compound was obtained from 5-fluoro-2-methoxylbenzonitrile (0.60 g, 4.00 mmol) following *General Procedure C*. The crude was purified using flash chromatography (95/5 n-heptane/EtOAc) to yield after crystallization with n-heptane 0.13 g (16%) as a red solid. *Rf* = 0.18 (n-heptane:10%EtOAc); ^{1}H NMR (600 MHz, Chloroform-*d*) δ 10.23 (s, 1H), 8.26 (s, 1H), 8.12 (d, *J* = 9.4 Hz, 1H), 7.17 (d, *J* = 9.1 Hz, 1H), 2.50 (s, 3H); ^{13}C NMR (151 MHz, Chloroform-*d*) δ 166.0 (d, *J* = 3.4 Hz), 163.5 (d, *J* = 246.8 Hz), 158.1, 142.0 (d, *J* = 7.8 Hz), 133.5 (d, *J* = 8.9 Hz), 124.8, 121.0 (d, *J* = 21.2 Hz), 112.4 (d, *J* = 24.2 Hz), 21.6; HPLC-MS [M+H]$^{+}$ m/z calc. for $[C_9H_7FN_4O]^{+}$: 207.07; Found: 207.9.

3-(5-iodo-2-methoxyphenyl)-1,2,4,5-tetrazine (39a)

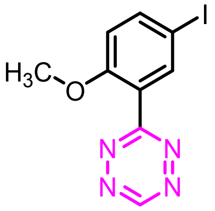

The final compound was obtained from 3-iodo-5-methoxybenzonitrile (1.03 g, 4.00 mmol) following *General Procedure C*. The crude was purified using flash chromatography (95/5 n-heptane/EtOAc) to yield after crystallization with n-heptane 0.19 g (15%) as a red solid. *Rf* = 0.21 (n-heptane:20%EtOAc); ^{1}H NMR (600 MHz, Chloroform-*d*) δ 10.21 (s, 1H), 8.21 (s, 1H), 7.82 (dd, *J* = 8.8, 2.3 Hz, 1H), 6.88 (d, *J* = 8.8 Hz, 1H), 3.87 (s, 3H); ^{13}C NMR (151 MHz, Chloroform-*d*) δ 167.3, 158.4, 156.9, 142.1, 140.1, 124.1, 114.6, 82.7, 56.4; HRMS (MALDI-TOF) calculated for $C_9H_8N_2I$ [M+H]$^{-}$: 298.9788, found: 298.9790.

N-(2-cyano-4-fluorophenyl)acetamide (16a-*o*)

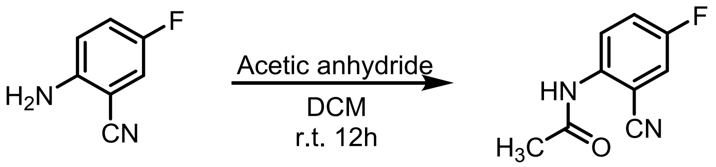

To a solution of the 2-amino-5-fluorobenzonitrile (0.82 g, 6.00 mmol) in DCM (30.0 mL) was added acetic anhydride (0.80 mL, 8.40 mmol). The mixture was stirred at room temperature for 12 hours. The suspension was filtered, and the solvent removed under vacuum. Purification by flash chromatography (70/30 Heptane/EtOAc) afforded 0.81 g (76%) of **16a-*o*** as a white solid. *Rf* = 0.41 (n-heptane:40%EtOAc); ^{1}H NMR (400 MHz, DMSO-d_6) δ 10.16 (s, 1H), 7.81 (dd, *J* = 8.4, 1.8 Hz, 1H), 7.59 (d, *J* = 1.7 Hz, 1H), 7.57 (d, *J* = 1.7 Hz, 1H), 2.09 (s, 3H); ^{13}C NMR (101 MHz, DMSO-d_6) δ 169.2, 158.9 (d, *J* = 244.3 Hz), 137.6 (d, *J* = 3.0 Hz), 128.4 (d, *J* = 8.7 Hz), 121.7 (d, *J* = 22.4 Hz), 120.0 (d, *J* = 26.0 Hz), 116.2 (d, *J* = 2.7 Hz), 109.3, 23.5.

2-cyano-4-fluorobenzamide (17a-*o*)

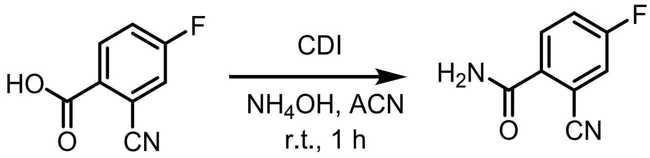

To a solution of 2-cyano-4-fluorobenzoic acid (0.99 g, 6.0 mmol) in MeCN (20 mL) was added 1,1'-carbonyldiimidazole (1.46 g, 9.0 mmol). The mixture was stirred at room temperature for 45 min, before addition of aqueous ammonium hydroxide solution (35%, 20 mL). The reaction mixture was stirred for 45 min and ice-cold water (20 mL) was added. The precipitate was collected by filtration and dried to give 0.71 g (71%) of the desired compound as a white solid. *Rf* = 0.18 (Heptane: 60%EtOAc); ^{1}H NMR (400 MHz, DMSO-d_6) δ 10.47 (s, 1H), 9.67 (s, 1H), 8.79 – 7.87 (m, 2H), 7.50 (s, 1H); ^{13}C NMR (101 MHz, DMSO-d_6) δ 172.3 (d, *J* = 20.8 Hz), 165.4 (d, *J* = 249.8 Hz), 163.4 (d, *J* = 9.7 Hz), 136.6, 129.4, 125.3 (d, *J* = 9.8 Hz), 119.7 (d, *J* = 23.8 Hz), 110.0 (d, *J* = 25.5 Hz).

2-Cyano-4-fluoro-N-methylbenzamide (18a-*o*)

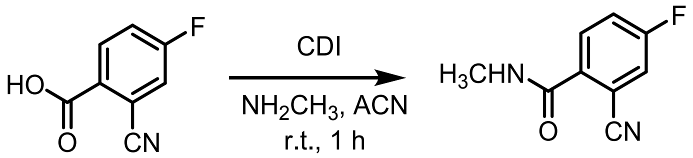

The compound was obtained from 2-cyano-4-fluorobenzoic acid (0.99 g, 6.0 mmol) and aqueous methylamine solution (40%, 20 mL) as reported above for to give 0.74 g (69%) of the desired compound as a white solid. Rf = 0.24 (Heptane/EtOAc 40/60); ^{1}H NMR (400 MHz, DMSO-d_6) δ 10.11 (s, 0H), 9.45 (s, 0H), 8.03 (dd, *J* = 8.4, 2.3 Hz, 0H), 7.82 (dd, *J* = 8.3, 4.8 Hz, 0H), 7.72 – 7.54 (m, 0H), 7.72 – 7.16 (m, 0H), 3.12 (s, 1H); ^{13}C NMR (101 MHz, DMSO-d_6) δ 167.0, 165.59 (d, *J* = 250.2 Hz), 158.6, 134.5 (d, *J* = 10.3 Hz), 127.4 (d, *J* = 2.5 Hz), 125.4 (d, *J* = 9.7 Hz), 119.6 (d, *J* = 23.9 Hz), 110.5 (d, *J* = 25.8 Hz), 24.9; HRMS (MALDI-TOF) calculated for $C_9H_8N_2OF$ $[M+H]^-$: 179.0615, found: 179.0614.

Synthesis of ylide tetrazine

6,10-dioxaspiro[4.5]decane-7,9-dione (2a)[8]

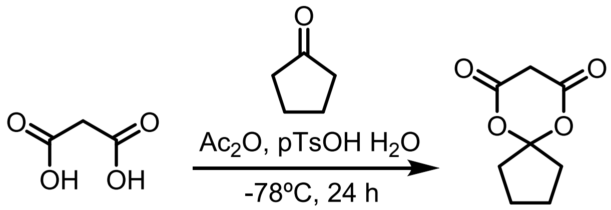

2a

Malonic acid (2 g) and pTsOH·H_2O (73 mg) were added to a flask and cooled to -75 °C. Ac_2O (2.7 mL) was dded dropwise and then cyclopentanone (1.7 mL) under stirring. The reaction was allowed to heat to room temperature overnight. Water was added tot he reaction (10 mL) and it was cooled down, until the formation of a white precipitate was observed. The precipitate was then filreted and washed with water and cold EtOH. The white crystal powder was collected to yield 165 mg. 1H-NMR (400 MHz, DMSO-d_6): δ 8.14 (dd, 2H) 8.06 (dd, 2H), 2.90 (s, 4H), 2.01 (m, 4H), 1.70 (m, 4H); 13C-NMR (101 MHz, DMSO-d_6): δ 170.1, 163.5, 161.1, 133.1, 132.0, 126.3, 123.7, 112.3, 58.9, 36.8, 25.5, 22.8.

8-((4-(6-methyl-1,2,4,5-tetrazin-3-yl)phenyl)-l3-iodaneylidene)-6,10-dioxaspiro[4.5]decane-7,9-dione (2)

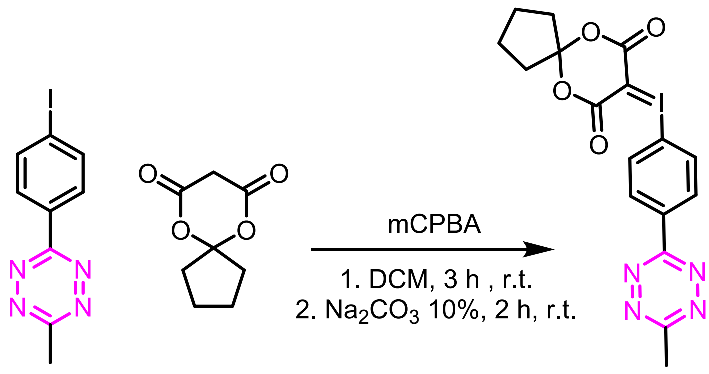

1 **2a** **2**

3-(4-iodophenyl)-6-methyl-1,2,4,5-tetrazine (15 mg, 0.05 mmol, 1 equiv) is dissolved DCM (1 mL/1mmol) in a sealed tube before adding mCPBA (13.8 mg, 0.06 mmol, 1.2 equiv), the mixture is sealed and allowed to stir at room temperature for 3 hours. A solution of 6,10-dioxaspiro[4.5]decane-7,9-dione (9.4 mg, 0.05 mmol,1.1 equiv) in Na_2CO_3 10% (2.86 mL/mmol) is prepared and then added dropwise to the mixture in the sealed tube. The mixture was stirred at room temperature for additional 2 hours. To the reaction mixture 5 mL of water is added and is extracted by DCM, dried over $MgSO_4$, filtered and concentrated *in vacuo*. The crude was submited to combi flash from 100% DCM to DCM/10% EtOH. All fractions containing compound were concentrated, disolved in warm methanol and left to crystalized at 4 °C, which afforded pink crystals (5.4 mg, 15%). ^{1}H NMR (400 MHz, Methanol-d_4) δ 8.65 (d, J = 8.6 Hz, 2H), 8.19 (d, J = 8.6 Hz, 2H), 3.11 (s, 3H), 2.16 (t, J = 7.4 Hz, 4H), 1.84 (t, J = 7.5 Hz, 4H); ^{13}C NMR (151 MHz, Chloroform-d) δ 168.3, 164.4, 163.0, 138.7, 135.9, 133.9, 131.2, 117.8, 114.6, 37.6, 23.6, 21.5.

Synthesis of iodonium salt tetrazine

mesityl(4-(6-methyl-1,2,4,5-tetrazin-3-yl)phenyl)iodonium (5)[9]

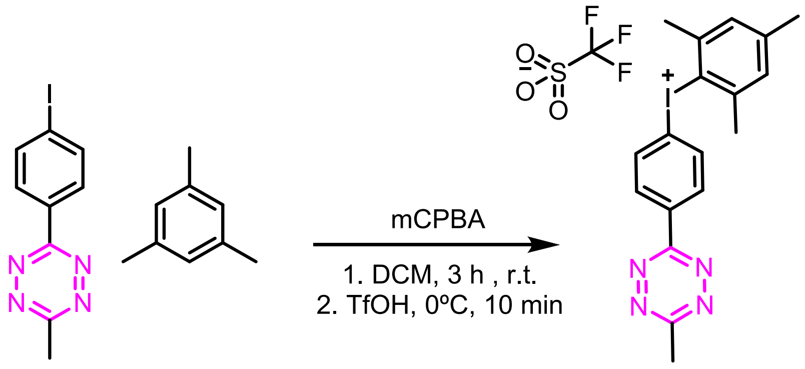

1 **5**

In a sealed tube m-Chloroperbenzoic acid (11.3 mg, 0.05 mmol) and 3-(4-iodophenyl)-6-methyl-1,2,4,5-tetrazine (**1**) (10 mg, 0.03 mmol) were dissolved in DCM (1 mL/0.23 mmol) and stirred at r.t. during 3 hours. Mesitulene (5.1 µL, 0.04 mmol) is added and the mixture is cooled to 0ºC followed by dropwise addition of TfOH (8.9µL, 0.10mmol). The reaction mixture was stirred at r.t during 10 minutes. The crude reaction was concentrated under vacuum. Diethyl ether was added and the mixture was stirred at r.t. during 20 minutes and then stored in the freezer during 1 hour for ensure complete precipitation, before filtered and washed with diethyl ether. The resulting solid was collected with methanol and dried under vacuum (12mg, 71%). ^{1}H NMR (600 MHz, MeOD) δ 8.57 (d, J = 8.7 Hz, 2H), 8.07 (d, J = 8.7 Hz, 2H), 3.00 (s, 3H), 2.64 (s, 6H), 2.31 (s, 3H).

Synthesis of boronic ester tetrazine

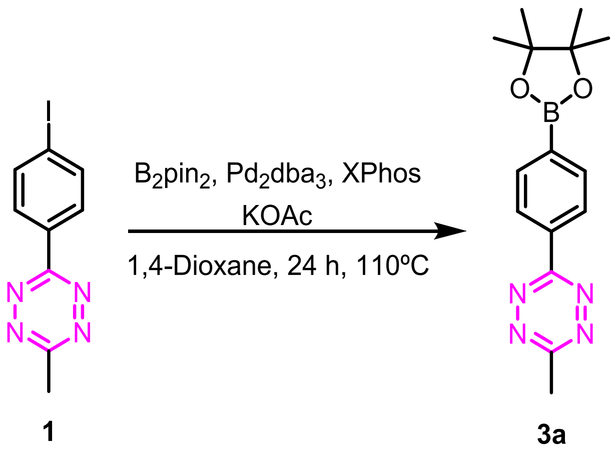

Compound **3a** was synthesized according a previously reported method with minor modifications.[10] An oven-dried MW vial was charged with Pd_2dba_3 (6.7 mg, 0.007 mmol), XPhos (6.9 mg, 0.01 mmol), 3-(4-iodophenyl)-6-methyl-1,2,4,5-tetrazine (**1**) (50 mg, 0.242 mmol), bis(pinacolato)diboron (123 mg, 0.484 mmol) and KOAc (72 mg, 0.726 mmol). The MW vial was sealed with a septum and then evacuated and backfilled with argon (this sequence was carried out two times). 1,4-Dioxane (0.50 mL) was added via syringe, through the septum. The reaction mixture was refluxed at 110°C for 24 h, then NaNO2 and AcOH was added to oxidise back the tetrazine core. The solution was extracted with DCM washed with brine, dried over $MgSO_4$, filtered and concentrated under reduced pressure. The tetrazine was then purified via automatic flash chromatography utilising n-heptane:EtOAc (80/20), resulting in 45 mg of a pink solid (63%). ^{1}H NMR (600 MHz, $CDCl_3$) δ 8.58 (d, *J* = 8.3 Hz, 2H), 8.02 (d, *J* = 8.2 Hz, 2H), 3.10 (s, 3H), 1.38 (s, 12H). ^{13}C NMR (151 MHz, $CDCl_3$) δ 167.47, 164.34, 135.62, 134.18, 127.12, 84.36, 25.05, 21.32. HPLC-MS $[M+H]^+$ m/z calc. for $[C_{15}H_{20}N_4O_2B]^+$: 299.19; Found: 299.32.

<u>General Procedure D.1.</u> *Synthesis of stannanes tetrazines*

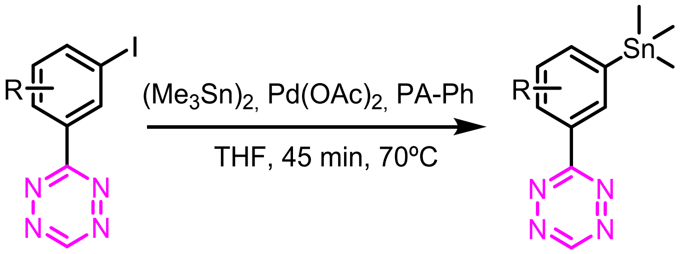

The preparation of these intermediates, was performed using a method described previously with minor modifications.[11] Palladium acetate (4.5 mg, 12%) and 1,3,5,7-tetramethyl-2,4,8-trioxa-(2,4-dimethoxyphenyl)-6-phosphaadamantane (PA-Ph) (9.8 mg, 20%) dry THF (1.5 mL) and hexamethylditin (75 µL, 137 mg, 0.42 mmol, 2.5 equiv.) were successively added to a microwave vial equipped with a stir bar which was then sealed and purged with N_2. A solution of the appropriate iodo-phenyl-1,2,4,5-tetrazine (0.17 mmol) in dry THF (1 mL) was added via a syringe and the reaction allowed to stir at 70 ºC in a microwave for 45 minutes. The reaction was allowed to cool to room temperature and unsealed before being quenched with saturated aqueous KF (1 mL). The solution was extracted with DCM washed with brine, dried over $MgSO_4$, filtered and concentrated under reduced pressure. The tetrazine was then purified via automatic flash chromatography utilising n-heptane and EtOAc as the eluent.

3-(4-trimethyltin)-6-methyl-1,2,4,5-tetrazine (3)

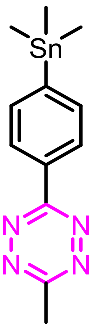

The final compound was obtained from 50 mg (0.17 mmol) of the starting material, following the *General Procedure D.1.* The crude was purified using flash chromatography (90/10 n-heptane/EtOAc) to yield 46 mg (76%) of a pink solid. *Rf* = 0.39 (n-heptane:10%EtOAc); ^{1}H NMR (400 MHz, Chloroform-*d*) δ 8.52 (d, J = 8.2 Hz, 2H), 7.73 (d, J = 8.2 Hz, 2H), 3.09 (s, 3H), 0.36 (s, 9H).; ^{13}C NMR (101 MHz, Chloroform-*d*) δ 167.4, 164.6, 149.3, 136.8, 131.6, 126.8, 21.3, -9.4.; HPLC-MS [M+H]$^{+}$ m/z calc. for $[C_{12}H_{17}SnN_4]^{+}$: 337.04; Found: 337.45.

3-(3-trimethyltin)-6-methyl-1,2,4,5-tetrazine (22)

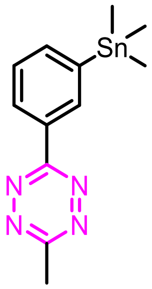

The final compound was obtained from 50 mg (0.17 mmol) of the starting material, following the *General Procedure D.1.* The crude was purified using flash chromatography (90/10 n-heptane/EtOAc) to yield 32 mg (65%) of a pink solid. *Rf* = 0.38 (n-heptane:10%EtOAc); ^{1}H NMR (400 MHz, Chloroform-*d*) δ 8.78 – 8.62 (m, 1H), 8.51 (ddd, *J* = 7.9, 2.0, 1.4 Hz, 1H), 7.74 (dt, *J* = 7.2, 1.2 Hz, 1H), 7.55 (ddd, *J* = 7.8, 7.1, 0.6 Hz, 1H), 3.10 (s, 3H), 0.37 (s, 9H).; ^{13}C NMR (101 MHz, Chloroform-*d*) δ 167.3, 164.6, 144.1, 140.2, 135.3, 131.3, 128.8, 128.0, 21.3, -9.3.; HPLC-MS [M+H]$^{+}$ m/z calc. for $[C_{12}H_{17}SnN_4]^{+}$: 337.04; Found: 337.38.

3-(4-trimethyltin)-6-methyl-1,2,4,5-tetrazine (27)

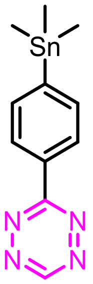

The final compound was obtained from 50 mg (0.17 mmol) of the starting material, following the *General Procedure D.1.* The crude was purified using flash chromatography (90/10 n-heptane/EtOAc) to yield 27 mg (61%) of a pink solid. *Rf* = 0.43 (n-heptane:20%EtOAc); ^{1}H NMR (400 MHz, Chloroform-*d*) δ 10.20 (s, 1H), 8.58 – 8.54 (m, 2H), 7.84 – 7.67 (m, 2H), 0.37 (s, 9H).; ^{13}C NMR (101 MHz, Chloroform-*d*) δ 167.0, 158.0, 150.3, 136.9, 130.3, 127.4, -9.3.; HPLC-MS [M+H]$^{+}$ m/z calc. for $[C_{11}H_{15}SnN_4]^{+}$: 323.04; Found: 323.38.

3-(3-trimethyltin)-1,2,4,5-tetrazine (28)

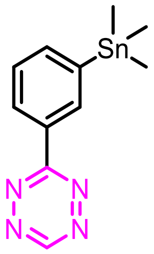

The final compound was obtained from 50 mg (0.17 mmol) of the starting material, following the *General Procedure D.1.* The crude was purified using flash chromatography (90/10 n-heptane/EtOAc) to yield 28 mg (58%) of a pink solid. *Rf* = 0.30 (n-heptane:10%EtOAc); ^{1}H NMR (400 MHz, Chloroform-*d*) δ 10.21 (s, 1H), 8.74 (d, *J* = 1.8 Hz, 1H), 8.55 (dt, *J* = 7.9, 1.6 Hz, 1H), 7.78 (d, *J* = 7.2 Hz, 1H), 7.57 (t, *J* = 7.5 Hz, 1H), 0.37 (s, 7H).; ^{13}C NMR (101 MHz, Chloroform-*d*) δ 167.0, 157.9, 144.3, 140.8, 135.6, 131.1, 128.9, 128.3, -9.3.; HPLC-MS [M+H]$^{+}$ m/z calc. for $[C_{11}H_{15}SnN_4]^{+}$: 323.04; Found: 323.38.

3-(4-methoxy-3-(trimethylstannyl)phenyl)-1,2,4,5-tetrazine (30)

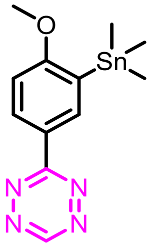

The final compound was obtained from 50 mg (0.17 mmol) of the starting material, following the *General Procedure D.1.* The crude was purified using flash chromatography (90/10 n-heptane/EtOAc) to yield 20 mg (36%) of a pink solid. *Rf* = 0.28 (n-heptane:10%EtOAc); ^{1}H NMR (600 MHz, Chloroform-*d*) δ 10.11 (s, 1H), 8.65 – 8.52 (m, 2H), 7.00 (d, *J* = 8.5 Hz, 1H), 3.91 (s, 3H), 0.34 (s, 9H); ^{13}C NMR (151 MHz, Chloroform-*d*) δ 168.2, 166.6, 157.4, 136.9, 132.4, 131.4, 124.3, 109.7, 55.8, -8.9; HPLC-MS [M+H]$^{+}$ m/z calc. for $[C_{12}H_{16}N_4OSn]^{+}$: 353.04; Found: 353.1.

3-(2-methoxy-5-(trimethylstannyl)phenyl)-1,2,4,5-tetrazine (40)

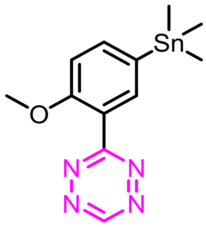

The final compound was obtained from 50 mg (0.17 mmol) of the starting material, following the *General Procedure D.1.* The crude was purified using flash chromatography (90/10 n-heptane/EtOAc) to yield 23 mg (40%) of a pink solid. *Rf* = 0.16 (n-heptane:10%EtOAc); ^{1}H NMR (600 MHz, Chloroform-*d*) δ 10.21 (s, 1H), 8.11 – 7.92 (m, 1H), 7.75 – 7.58 (m, 1H), 7.19 – 7.05 (m, 1H), 3.90 (s, 3H), 0.32 (s, 9H); ^{13}C NMR (151 MHz, Chloroform-*d*) δ 168.9, 158.9, 156.9, 141.1, 139.2, 133.7, 122.1, 112.2, 56.1, -9.2; HPLC-MS [M+H]$^{+}$ m/z calc. for $[C_{12}H_{16}N_4OSn]^{+}$: 353.04; Found: 353.1.

3-phenyl-6-(4-(trimethylstannyl)phenyl)-1,2,4,5-tetrazine (23)

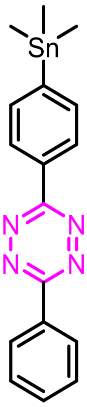

The final compound was obtained from 50 mg (0.17 mmol) of the starting material, following the *General Procedure D.1.* The crude was purified using flash chromatography (90/10 n-heptane/EtOAc) to yield 52 mg (95%) of a pink solid. *Rf* = 0.48 (n-heptane:10%EtOAc). ^{1}H NMR (400 MHz, Chloroform-*d*) δ 8.70 – 8.63 (m, 2H), 8.63 – 8.53 (m, 2H), 7.85 – 7.68 (m, 2H), 7.68 – 7.57 (m, 3H), 0.37 (s, 9H).; ^{13}C NMR (151 MHz, Chloroform-*d*) δ 164.5, 164.1, 149.7, 136.9, 132.8, 132.0, 131.6, 129.5, 128.1, 127.1, -9.3.; HPLC-MS $[M+H]^+$ m/z calc. for $[C_{17}H_{18}N_4Sn]^+$: 399.06; Found: 399.1.

3-phenyl-6-(3-(trimethylstannyl)phenyl)-1,2,4,5-tetrazine (24)

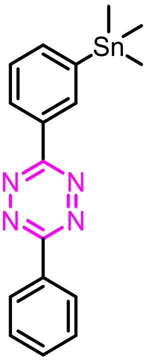

The final compound was obtained from 50 mg (0.17 mmol) of the starting material, following the *General Procedure D.1.* The crude was purified using flash chromatography (90/10 n-heptane/EtOAc) to yield 53 mg (95%) of a pink solid. *Rf* = 0.48 (n-heptane:10%EtOAc); ^{1}H NMR (600 MHz, Chloroform-*d*) δ 8.78 (dt, *J* = 1.7, 0.7 Hz, 1H), 8.69 – 8.65 (m, 2H), 8.59 (ddd, *J* = 7.9, 2.0, 1.3 Hz, 1H), 7.77 (dt, *J* = 7.2, 1.2 Hz, 1H), 7.65 – 7.61 (m, 3H), 7.58 (ddd, *J* = 7.8, 7.2, 0.6 Hz, 1H), 0.38 (s, 9H).; ^{13}C NMR (151 MHz, Chloroform-*d*) δ 164.4, 164.1, 144.2, 140.3, 135.3, 132.8, 132.0, 131.3, 129.5, 128.9, 128.1, 128.0, -9.2; HPLC-MS $[M+H]^+$ m/z calc. for $[C_{17}H_{18}N_4Sn]^+$: 399.06; Found: 399.1.

3-(pyridin-2-yl)-6-(5-(trimethylstannyl)pyridin-2-yl)-1,2-dihydro-1,2,4,5-tetrazine (26b)

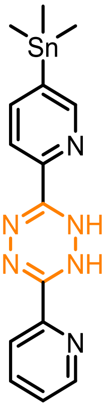

The final compound was obtained from 50 mg (0.17 mmol) of the starting material, following the *General Procedure D.1.* The crude was purified using flash chromatography (70/30 n-heptane/EtOAc) to yield 47 mg (85%) of an orange solid. *Rf* = 0.50 (n-heptane:10%EtOAc); ^{1}H NMR (400 MHz, Chloroform-*d*) δ 8.62 – 8.51 (m, 4H), 8.05 (dt, *J* = 8.0, 1.1 Hz, 1H), 7.97 (dd, *J* = 7.7, 1.1 Hz, 1H), 7.84 (dd, *J* = 7.7, 1.5 Hz, 1H), 7.75 (td, *J* = 7.7, 1.7 Hz, 1H), 7.34 (ddd, *J* = 7.5, 4.9, 1.2 Hz, 1H), 0.36 (s, 9H).; ^{13}C NMR (101 MHz, Chloroform-d) δ 154.2, 148.5, 147.7, 147.2, 147.1, 146.8, 144.2, 139.8, 136.8, 125.0, 121.4, 121.0, -9.4.

3-(pyridin-2-yl)-6-(5-(trimethylstannyl)pyridin-2-yl)-1,2,4,5-tetrazine (26)

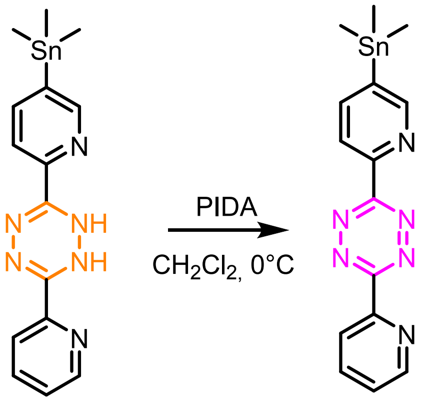

The 1,2-dihydro-1,2,4,5-tetrazine stannane was dissolved in dry DCM and cooled to 0 °C, followed by the portion wise addition of (Diacetoxyiodo)benzene (1.2 equiv.). The reaction was allowed to warm to r.t. and stirred for 3h. The crude was purified using flash chromatography. *Rf* = 0.29 (EtOAc:50%n-heptane); ^{1}H NMR (400 MHz, Chloroform-*d*) δ 8.99 (dq, *J* = 4.7, 1.0 Hz, 1H), 8.75 (dt, *J* = 7.9, 1.0 Hz, 1H), 8.66 (dd, *J* = 7.6, 1.0 Hz, 1H), 8.11 (dd, *J* = 7.6, 1.5 Hz, 1H), 8.01 (td, *J* = 7.8, 1.8 Hz, 1H), 7.58 (ddd, *J* = 7.7, 4.8, 1.1 Hz, 1H).; ^{13}C NMR (101 MHz, Chloroform-*d*) δ 164.5, 164.0, 156.8, 151.2, 150.3, 149.7, 145.2, 142.8, 137.6, 126.7, 124.6, 124.1, 77.5, -9.3; HPLC-MS $[M+H]^{+}$ m/z calc. for $[C_{15}H_{16}N_{6}Sn]^{+}$: 401.05; Found: 401.1.

3-(pyridin-2-yl)-6-(4-(trimethylstannyl)phenyl)-1,2,4,5-tetrazine (25)

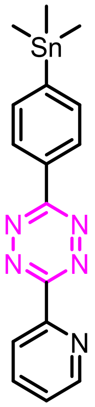

The final compound was obtained from 50 mg (0.17 mmol) of the starting material, following the *General Procedure D.1.* The final compound autooxidised under reaction conditions. The crude was purified using flash chromatography (60/40 n-heptane/EtOAc) to yield 14 mg (49%) of a pink solid. *Rf* = 0.31 (EtOAc:50%n-heptane); ^{1}H NMR (600 MHz, Chloroform-*d*) δ 9.00 – 8.94 (m, 1H), 8.69 (dt, *J* = 7.9, 1.0 Hz, 1H), 8.64 – 8.60 (m, 2H), 8.00 (td, *J* = 7.8, 1.8 Hz, 1H), 7.78 – 7.74 (m, 2H), 7.59 – 7.52 (m, 1H), 0.37 (s, 9H); ^{13}C NMR (151 MHz, Chloroform-*d*) δ 164.9, 163.6, 151.1, 150.5, 150.3, 137.6, 136.9, 131.4, 127.5, 126.4, 124.0, -9.3; HPLC-MS $[M+H]^{+}$ m/z calc. for $[C_{16}H_{17}N_{5}Sn]^{+}$: 400.06; Found: 400.1.

General Procedure D.2. *Synthesis of stannanes tetrazines*

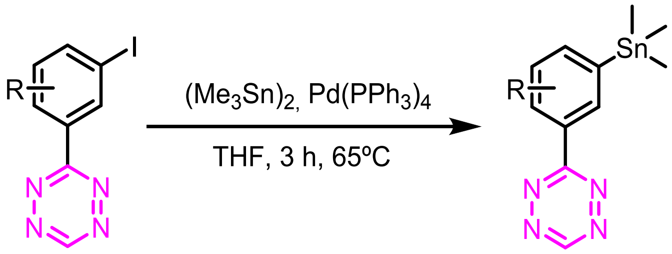

The preparation of these intermediates, was performed using a method described previously with minor modifications.[11] $Pd(PPh_3)_4$ (19.4 mg, 10%) and Hexamethylditin (87 µL, 0.42 mmol, 2.5 equiv.) were successively added to a microwave vial equipped with a stir bar which was then sealed and purged with N_2. A solution of the appropriate *iodo-phenyl-1,2,4,5-tetrazine* (0.17 mmol) in dry THF (2.5 mL) was added via a syringe and the reaction allowed to stir at 65 ºC in a microwave for 3 hours. The reaction was allowed to cool to room temperature and unsealed before being quenched with saturated aqueous KF (1 mL). The solution was extracted with DCM washed with brine, dried over $MgSO_4$, filtered and concentrated under reduced pressure. The tetrazine was then purified via automatic flash chromatography utilising n-heptane and EtOAc as eluent.

3-(3-methyl-5-(trimethylstannyl)phenyl)-1,2,4,5-tetrazine (34)

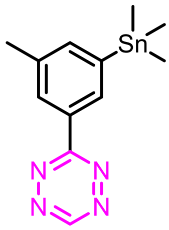

The final compound was obtained from 50 mg (0.17 mmol) of the starting material, following the *General Procedure D.2.* The crude was purified using flash chromatography (90/10 n-heptane/EtOAc) to yield 25 mg (27%) of a pink solid. *Rf* = 0.34 (n-heptane:20%EtOAc); ^{1}H NMR (400 MHz, Chloroform-*d*) δ 10.20 (s, 1H), 8.65 – 8.50 (m, 1H), 8.48 – 8.33 (m, 1H), 7.73 – 7.43 (m, 1H), 2.48 (s, 3H), 0.36 (s, 9H); ^{13}C NMR (151 MHz, Chloroform-*d*) δ 167.1, 157.9, 144.1, 141.6, 138.6, 132.8, 131.0, 128.9, 21.6, -9.3; HPLC-MS $[M+H]^+$ m/z calc. for $[C_{12}H_{16}N_4Sn]^+$: 337.05; Found: 337.1.

3-(2-methyl-5-(trimethylstannyl)phenyl)-1,2,4,5-tetrazine (39)

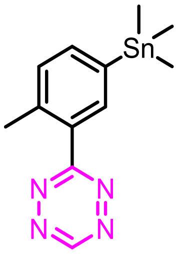

The final compound was obtained from 50 mg (0.17 mmol) of the starting material, following the *General Procedure D.2.* The crude was purified using flash chromatography (90/10 n-heptane/EtOAc) to yield 10 mg (18%) of a pink solid. *Rf* = 0.32 (n-heptane:20%EtOAc); ^{1}H NMR (600 MHz, Chloroform-*d*) δ 10.23 (s, 1H), 8.15 (s, 1H), 7.63 (d, *J* = 7.4 Hz, 1H), 7.38 (d, *J* = 7.4 Hz, 1H), 2.62 (s, 3H), 0.33 (s, 9H); ^{13}C NMR (151 MHz, Chloroform-*d*) δ 170.2, 156.9, 140.5, 139.5, 138.7, 138.5, 131.6, 131.5, 21.4, -9.3; HPLC-MS $[M+H]^+$ m/z calc. for $[C_{12}H_{16}N_4Sn]^+$: 337.05; Found: 337.1; HRMS (MALDI-TOF) calculated for (reduced in situ) $C_{12}H_{21}N_4Sn$ $[M+H]^-$: 341.0783, found: 341.0778.

3-(3-methoxy-5-(trimethylstannyl)phenyl)-1,2,4,5-tetrazine (35)

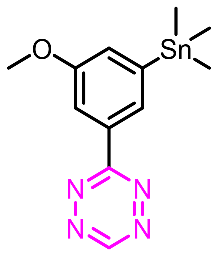

The final compound was obtained from 55 mg (0.17 mmol) of the starting material, following the *General Procedure D.2.* The crude was purified using flash chromatography (90/10 n-heptane/EtOAc) to yield 40 mg (65%) of **35** as a purple solid. *Rf* = 0.41 (n-heptane:20%EtOAc); ^{1}H NMR (400 MHz, Chloroform-*d*) δ 10.21 (s, 1H), 8.35 (d, *J* = 1.5 Hz, 1H), 8.07 (dd, *J* = 2.7, 1.6 Hz, 1H), 7.32 (d, *J* = 2.7 Hz, 1H), 3.94 (s, 3H), 0.37 (s, 9H); ^{13}C NMR (101 MHz, Chloroform-*d*) δ 166.6, 157.8, 145.6, 132.1, 127.9, 127.6, 111.7, 55.5, -9.4; HPLC-MS [M+H]$^+$ m/z calc. for $[C_{12}H_{17}N_4SnO]^+$: 353.04; Found: 353.07; HRMS (MALDI-TOF) calculated for (reduced in situ) $C_{12}H_{21}N_4OSn$ [M+H]$^-$: 357.0732, found: 357.0723.

N-(3-(1,2,4,5-tetrazin-3-yl)-5-(trimethylstannyl)phenyl)acetamide (36)

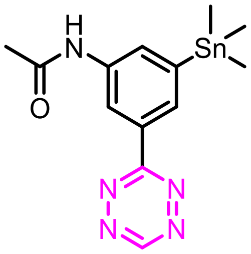

The final compound was obtained from 55 mg (0.17 mmol) of the starting material, following the *General Procedure D.2.* The crude was purified using flash chromatography (70/30 n-heptane/EtOAc) to yield 25 mg (41%) of **36** as a purple oil. *Rf* = 0.35 (n-heptane:50%EtOAc); ^{1}H NMR (400 MHz, Chloroform-*d*) δ 10.20 (s, 1H), 8.56 (t, *J* = 2.1 Hz, 1H), 8.44 (s, 1H), 8.03 (d, *J* = 2.2 Hz, 1H), 7.63 (s, 1H), 2.24 (s, 3H), 0.36 (s, 9H); ^{13}C NMR (101 MHz, Chloroform-*d*) δ 168.7, 166.5, 157.8, 145.5, 138.4, 131.7, 131.5, 131.2, 119.4, 24.6, -9.3; HPLC-MS [M+H]$^+$ m/z calc. for $[C_{13}H_{18}N_5SnO]^+$: 380.05; Found: 380.09; HRMS (MALDI-TOF) calculated for (reduced in situ) $C_{13}H_{22}N_5OSn$ [M+H]$^-$: 384.0841, found: 384.0835.

3-(1,2,4,5-tetrazin-3-yl)-5-(trimethylstannyl)benzamide (37)

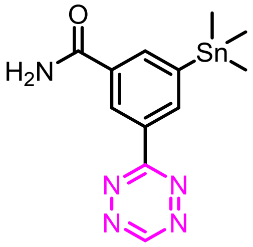

The final compound was obtained from 55 mg (0.17 mmol) of the starting material, following the *General Procedure D.2.* The crude was purified using flash chromatography (40/60 n-heptane/EtOAc) to yield 25 mg (41%) of **37** as a purple oil. *Rf* = 0.42 (n-heptane:60%EtOAc); ^{1}H NMR (600 MHz, Methanol-*d*$_4$) δ 10.28 (s, 1H), 8.76 (dd, *J* = 1.8, 0.9 Hz, 1H), 8.20 (dd, *J* = 1.9, 0.8 Hz, 1H), 0.31 (s, 9H); ^{13}C NMR (151 MHz, Methanol-*d*$_4$) δ 170.5, 166.3, 158.0, 144.8, 138.5, 137.7, 134.0, 131.7, 126.8, -11.1; HPLC-MS [M+H]$^+$ m/z calc. for $[C_{12}H_{16}N_5SnO]^+$: 366.04; Found: 366.08; HRMS (MALDI-TOF) calculated for (reduced in situ) $C_{12}H_{20}N_4OSn$ [M+H]$^-$: 370.0684, found: 370.0678.

N-methyl-3-(1,2,4,5-tetrazin-3-yl)-5-(trimethylstannyl)benzamide (38)

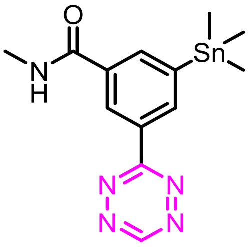

The final compound was obtained from 50 mg (0.14 mmol) of the starting material, following the *General Procedure D.2.* The crude was purified using flash chromatography (60/40 n-heptane/EtOAc) to yield 35 mg (63%) of **38** as a purple oil. *Rf* = 0.46 (n-heptane:60%EtOAC); ^{1}H NMR (400 MHz, Chloroform-*d*) δ 10.25 (s, 1H), 8.89 – 8.80 (m, 2H), 8.26 (dd, *J* = 1.9, 0.9 Hz, 1H), 6.37 (s, 1H), 3.08 (d, *J* = 4.8 Hz, 3H), 0.39 (s, 9H); ^{13}C NMR (101 MHz, Chloroform-*d*) δ 167.7, 166.2, 158.0, 145.6, 139.4, 138.0, 134.9, 130.9, 125.6, 27.0, -9.2; HPLC-MS [M+H]$^{+}$ m/z calc. for $[C_{13}H_{18}N_5SnO]^+$: 380.05; Found: 380.02; HRMS (MALDI-TOF) calculated for (reduced in situ) $C_{13}H_{22}N_4OSn$ [M+H]$^{-}$: 384.0841, found: 384.0836.

Compounds for blocking studies and final stannanes precursors

3-Fluoro-5-(1,2,4,5-tetrazin-3-yl)benzoic acid (19)

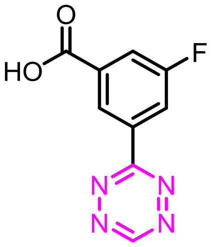

The final compound was obtained from 3-Fluoro-5-cyanobenzoic acid (0.66 g, 4.00 mmol) following *General Procedure C*. The crude was purified using flash chromatography (98/2 DCM/MeOH) to yield 210 mg (24%) of **19** as a red solid. *Rf* = 0.31 (n-heptane:60%EtOAC); ^{1}H NMR (400 MHz, Methanol-*d*$_4$) δ 10.32 (s, 1H), 8.95 (t, *J* = 1.5 Hz, 1H), 8.42 (ddd, *J* = 9.2, 2.6, 1.5 Hz, 1H), 7.90 (ddd, *J* = 8.8, 2.7, 1.4 Hz, 1H); ^{13}C NMR (101 MHz, Methanol-*d*$_4$) δ 166.0, 165.1, 163.1 (d, *J* = 247.1 Hz), 158.3, 134.9 (d, *J* = 8.2 Hz), 134.5 (d, *J* = 7.3 Hz), 124.6 (d, *J* = 3.1 Hz), 119.9 (d, *J* = 23.2 Hz), 118.3 (d, *J* = 24.7 Hz); HPLC-MS [M-H]$^{-}$ m/z calc. for $[C_9H_4FN_4O_2]^+$: 219.03; Found: 219.07.

3-(Bromomethyl)-5-fluorobenzonitrile (21a)

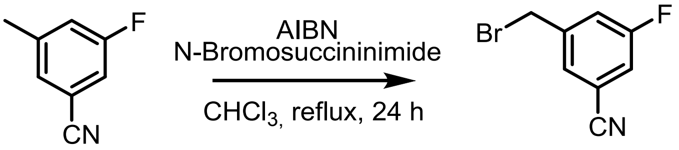

To a solution of 3-fluoro-5-methylbenzonitrile (2.61 g, 19.24 mmol) and NBS (5.13 g, 28.86 mmol) in $CHCl_3$ was added AIBN (1.26 g, 7.69 mmol). The reaction was refluxed for 24 h. The solvent was removed under vacuum and the crude purified by flash chromatography (heptane/EtOAc 95/5) to give 2.10 g (51%) of **21a** as a colorless oil. *Rf* = 0.32 (n-heptane:5%EtOAc); ^{1}H NMR (400 MHz, Chloroform-*d*) δ 7.50 (t, *J* = 1.5 Hz, 1H), 7.38 (dt, *J* = 8.9, 2.0 Hz, 1H), 7.30 (ddd, *J* = 7.9, 2.5, 1.3 Hz, 1H), 4.46 (s, 2H); ^{13}C NMR

(101 MHz, Chloroform-*d*) δ 162.1 (d, *J* = 251.3 Hz), 141.9 (d, *J* = 8.0 Hz), 128.5 (d, *J* = 3.4 Hz), 121.1 (d, *J* = 22.0 Hz), 119.0 (d, *J* = 24.7 Hz), 117.1 (d, *J* = 3.3 Hz), 114.3 (d, *J* = 9.9 Hz), 30.3 (d, *J* = 1.9 Hz).

Di-tert-butyl 2,2'-((3-cyano-5-fluorobenzyl)azanediyl)diacetate (21b)

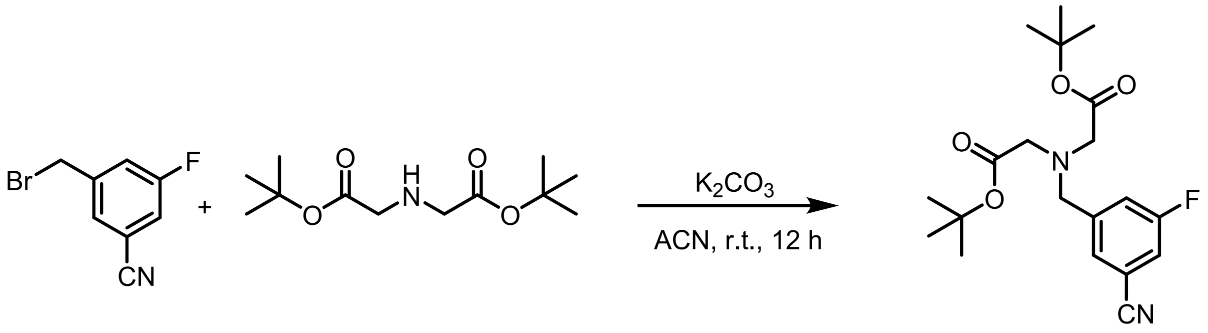

To a solution of 3-fluoro-5-bromomethylbenzonitrile (1.09 g, 5.10 mmol) in MeCN (30 mL) was added K_2CO_3 (1.06 g, 7.65 mmol) and di-tert-butyl iminodiacetate (1.50 g, 6.12 mmol). The reaction mixture was stirred at room temperature overnight. The solvent was removed in vacuo, and the resulting mixture was diluted with water (20 mL), extracted with EtOAc (2 x 25 mL), washed with brine (30 mL), dried over $MgSO_4$, filtered and concentrated under reduced pressure. Purification by flash chromatography (90/10 Heptane/EtOAc) afforded 1.72 g (89%) of **21b** as a white solid. *Rf* = 0.24 (n-heptane:10%EtOAc); ^{1}H NMR (400 MHz, Chloroform-*d*) δ 7.53 (s, 1H), 7.51 – 7.42 (m, 1H), 7.23 (ddd, *J* = 7.8, 2.5, 1.4 Hz, 1H), 3.93 (s, 2H), 3.39 (s, 4H), 1.46 (s, 18H); ^{13}C NMR (101 MHz, Chloroform-*d*) δ 170.1, 162.4 (d, *J* = 250.0 Hz), 144.0 (d, *J* = 7.5 Hz), 128.1 (d, *J* = 3.1 Hz), 121.0 (d, *J* = 21.5 Hz), 117.8 (d, *J* = 24.9 Hz), 117.6 (d, *J* = 3.4 Hz), 113.6 (d, *J* = 9.6 Hz), 81.4, 56.4 (d, *J* = 1.9 Hz), 55.3, 28.1; HRMS (MALDI-TOF) calculated for $C_{20}H_{28}N_2O_4F$ $[M+H]^-$: 379.2027, found: 379.2025.

Di-tert-butyl 2,2'-((3-fluoro-5-(1,2,4,5-tetrazin-3-yl)benzyl)azanediyl)diacetate (21c)

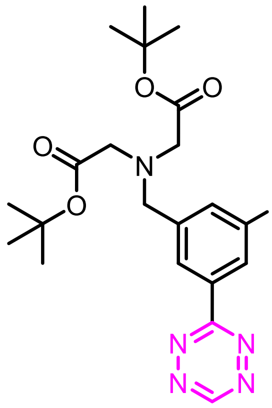

The compound was obtained from di-tert-butyl 2,2'-((3-cyano-5-fluorobenzyl)azanediyl)diacetate (1.70 g, 4.49 mmol) following *General Procedure C*. The crude was purified using flash chromatography (heptane/EtOAc 95/5) to yield 110 mg (24%) of **21c** as a red solid. *Rf* = 0.39 (n-heptane:20%EtOAC); ^{1}H NMR (400 MHz, Chloroform-*d*) δ 10.17 (s, 1H), 8.34 (d, *J* = 1.4 Hz, 1H), 8.14 (ddd, *J* = 9.2, 2.5, 1.5 Hz, 1H), 7.55 – 7.45 (m, 1H), 3.98 (s, 2H), 3.40 (s, 4H), 1.41 (s, 18H); ^{13}C NMR (101 MHz, Chloroform-*d*) δ 170.3, 165.8 (d, *J* = 3.2 Hz), 163.6 (d, *J* = 247.5 Hz), 158.0, 143.3 (d, *J* = 7.1 Hz), 133.5 (d, *J* = 8.7 Hz), 124.2 (d, *J* = 2.7 Hz), 120.6 (d, *J* = 21.8 Hz), 114.2 (d, *J* = 24.5 Hz), 81.3, 57.0 (d, *J* = 1.8 Hz), 55.4, 28.2; HRMS (MALDI-TOF) calculated for $C_{21}H_{29}N_5O_4F$ $[M+H]^-$: 434.2198, found: 434.2195.

1-Carboxy-N-(carboxymethyl)-N-(3-fluoro-5-(1,2,4,5-tetrazin-3-yl)benzyl)methanaminium 2,2,2-trifluoroacetate (21)

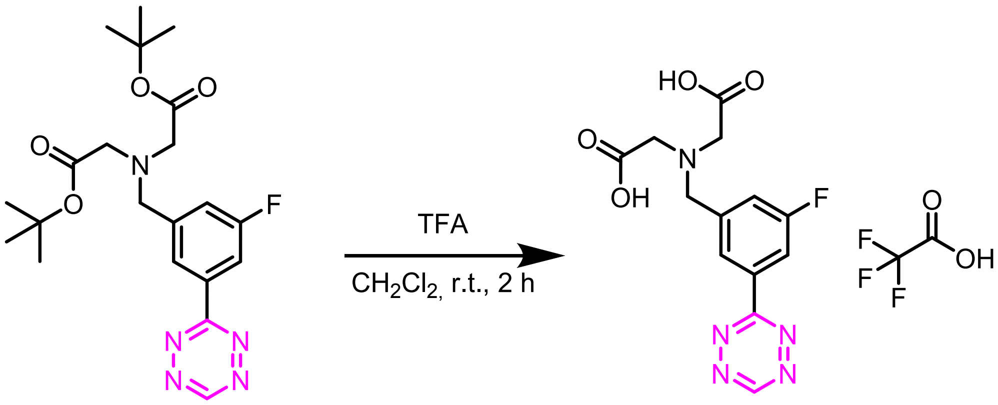

To a solution of di-tert-butyl 2,2'-((3-fluoro-5-(1,2,4,5-tetrazin-3-yl)benzyl)azanediyl)diacetate (0.15 g, 0.36 mmol) in DCM (5 mL) was added trifluoroacetic acid (5 mL). The reaction was stirred at room temperature for 2 hours. The solvent was then removed under reduced pressure to obtain a pik solid. NMR of the crude shows full conversion. Purification by preparative HPLC afforded 80 mg (51%) of **21** as a pink solid. ^{1}H NMR (400 MHz, Methanol-d_4) δ 10.42 (s, 1H), 8.60 (d, *J* = 1.4 Hz, 1H), 8.41 – 8.32 (m, 1H), 7.73 – 7.64 (m, 1H), 5.11 (s, 7H), 4.59 (s, 2H), 4.11 (s, 4H); ^{13}C NMR (101 MHz, Methanol-d_4) δ 168.7, 165.1 (d, *J* = 3.2 Hz), 163.3 (d, *J* = 247.6 Hz), 158.2, 135.4 (d, *J* = 7.7 Hz), 135.1 (d, *J* = 8.6 Hz), 126.1 (d, *J* = 3.0 Hz), 121.7 (d, *J* = 22.7 Hz), 115.5 (d, *J* = 24.4 Hz), 57.9, 53.6; HPLC-MS [M+H]$^+$ m/z calc. for $[C_{13}H_{13}FN_5O_4]^+$: 322.27; Found: 322.27; HRMS (MALDI-TOF) calculated for $C_{13}H_{13}N_5O_4F$ [M+H]$^-$: 322.0946, found: 322.0945.

3-(Bromomethyl)-5-iodobenzonitrile (20a)

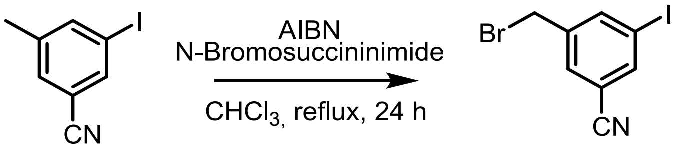

To a solution of 3-iodo-5-methylbenzonitrile (2.50 g, 10.28 mmol) and N-bromosuccinimide (2.28 g, 12.86 mmol) in $CHCl_3$ (40 mL) was added AIBN (0.67 g, 4.11 mmol). The reaction was refluxed for 24 h. The solvent was removed under vacuum and the crude purified by flash chromatography (heptane/EtOAc 95/5) to give 1.61 g (49%) of **20a** as a white solid. *Rf* = 0.28 (n-heptane:5%EtOAc); ^{1}H NMR (400 MHz, Chloroform-*d*) δ 7.96 (d, *J* = 1.6 Hz, 1H), 7.89 (d, *J* = 1.6 Hz, 1H), 7.64 (t, *J* = 1.6 Hz, 1H), 4.38 (s, 2H) ^{13}C NMR (101 MHz, Chloroform-*d*) δ 142.2, 140.9, 140.1, 131.7, 116.6, 114.6, 94.1, 29.9; HRMS (MALDI-TOF) calculated for C_8H_4NIBr [M+H]$^-$: 321.8728, found: no ionization detected.

Di-tert-butyl 2,2'-((3-cyano-5-iodobenzyl)azanediyl)diacetate (20b)

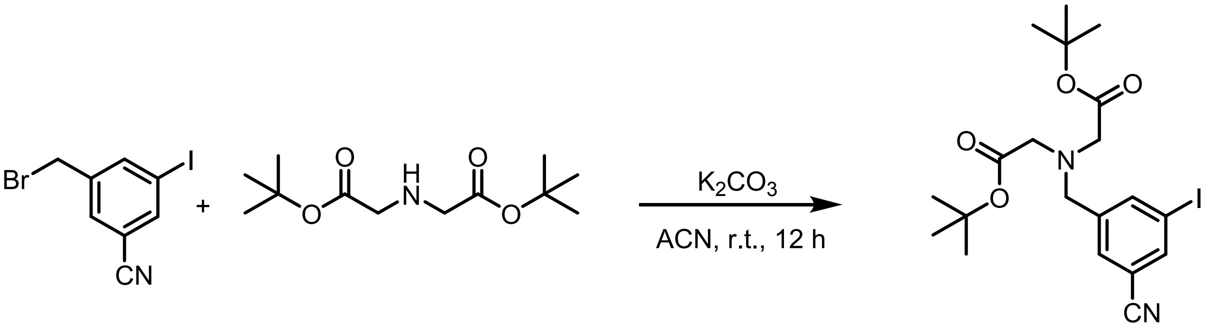

To a solution of 3-(bromomethyl)-5-iodobenzonitrile (1.00 g, 3.10 mmol) in MeCN (30 mL) was added K_2CO_3 (0.64 g, 7.65 mmol) and the corresponding amine (0.91 g, 3.72 mmol). The reaction mixture was stirred at room temperature overnight. The solvent was removed in vacuo, and the resulting mixture was diluted with water (20 mL), extracted with EtOAc (2 x 25 mL), washed with brine (30 mL), dried over $MgSO_4$, filtered and concentrated in vacuo. Purification by flash chromatography (90/10 Heptane/EtOAc) afforded 1.35 g (89%) of **20b** as a colorless oil. *Rf* = 0.31 (n-heptane:5%EtOAc); ^{1}H NMR (400 MHz, Chloroform-*d*) δ 7.96 (s, 1H), 7.80 (s, 1H),

7.65 (s, 1H), 3.83 (s, 2H), 3.33 (s, 4H), 1.40 (s, 18H); ^{13}C NMR (101 MHz, Chloroform-*d*) δ 167.0, 142.5, 142.1, 139.3, 131.6, 117.1, 114.1, 93.9, 81.5, 56.2, 55.2, 28.2; HRMS (MALDI-TOF) calculated for $C_{20}H_{28}N_2O_4I$ [M+H]$^-$: 487.1088, found: 487.1084.

Di-tert-butyl 2,2'-((3-iodo-5-(1,2,4,5-tetrazin-3-yl)benzyl)azanediyl)diacetate (20c)

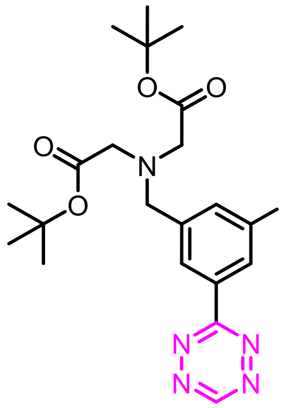

The compound was obtained from di-tert-butyl 2,2'-((3-cyano-5-iodobenzyl)azanediyl)diacetate (1.30 g, 2.67 mmol) following *General Procedure C*. The crude was purified using flash chromatography (heptane/EtOAc 95/5) to 0.37 g (26%) of **20c** as red oil. *Rf* = 0.39 (n-heptane:20%EtOAC); ^{1}H NMR (600 MHz, Chloroform-*d*) δ 10.22 (s, 1H), 8.84 (s, 1H), 8.55 (s, 1H), 8.10 (s, 1H), 3.99 (s, 2H), 3.44 (s, 4H), 1.46 (s, 18H); ^{13}C NMR (151 MHz, Chloroform-*d*) δ 170.1, 165.3, 158.0, 142.4, 136.1, 133.4, 129.0, 95.1, 81.4, 56.7, 55.3, 28.2; ; HRMS (MALDI-TOF) calculated for $C_{21}H_{29}N_5O_4I$ [M+H]$^-$: 542.1258, found: 542.1262.

Di-tert-butyl 2,2'-((3-(1,2,4,5-tetrazin-3-yl)-5-(trimethylstannyl)benzyl)azanediyl)diacetate (20)

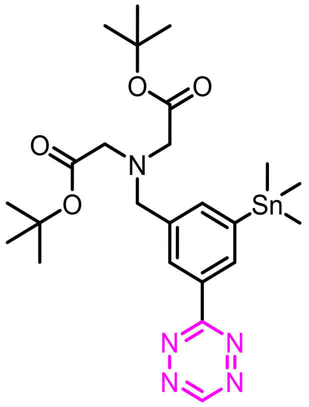

The final compound was obtained from 50 mg (0.09 mmol) of the starting material, following the *General Procedure D.2.* The crude was purified using flash chromatography (95/5 n-heptane/EtOAc) to yield 0.025 g (47%) of **20** as a purple oil. *Rf* = 0.37 (n-heptane:20%EtOAc); ^{1}H NMR (400 MHz, Chloroform-*d*) δ 10.20 (s, 1H), 8.63 (d, *J* = 1.8 Hz, 1H), 8.55 (s, 1H), 7.97 – 7.73 (m, 1H), 4.08 (s, 2H), 3.50 (s, 4H), 1.47 (s, 18H), 0.37 (s, 9H); ^{13}C NMR (101 MHz, Chloroform-*d*) δ 170.1, 166.8, 157.7, 144.3, 141.4, 134.6, 131.1, 129.0, 81.3, 57.4, 55.1, 28.2, -9.3; HPLC-MS [M+H]$^+$ m/z calc. for $[C_{24}H_{38}N_5SnO_4]^+$: 580.19; Found: 580.22; HRMS (MALDI-TOF) calculated for (reduced in situ) $C_{24}H_{38}N_5O_4Sn$ [M+H]$^-$: 580.1940, found: 580.1953.

Section S3: Radiochemistry

General information. Radiochemistry was performed at Department of Clinical Physiology, Nuclear Medicine & PET, Rigshospitalet, Denmark. [^{18}F]Fluoride was produced via the (p,n)-reaction in a cyclotron (60 mikroA CTI Siemens or 40 mikroA Scanditronix) by irradiating [^{18}O]H_2O with a 11 MeV (CTI siemens) or 16 MeV (Scanditronix) proton beam. Automated syntheses were performed on a Scansys Laboratorieteknik synthesis module housed in a hot cell. Analytical HPLC was performed on a Dionex system connected to a P680A pump, a UVD 170U detector and a Scansys radiodetector. The system was controlled by Chromeleon software. Semi-preparative HPLC was performed on the built-in HPLC system in the synthesis module and the flow rate was set to 4 mL/min at all times.

Radiochemical conversion (RCC) of all radiolabeled compounds was determined by analysing a labeled aliquot of the reaction mixture by radio-HPLC and analyzed by integrating the radioactive peaks from the reaction solution.[12] The products were characterized by comparing the radio-HPLC trace of the reaction mixtures with the HPLC UV traces of the authentic ^{19}F-reference samples, respectively. The radiochemical yield (RCY) was determined using the activity of [^{18}F]fluoride received from the cyclotron at the beginning of the synthesis and that of the formulated product at the end of the synthesis, the decomposition was corrected and have been decay corrected (d.c.). The molar activity (A_m) was determined by integrating the area of the UV absorbance peak corresponding to the radiolabeled product on the HPLC chromatogram. This area was converted into a molar mass by comparison with an average of integrated areas (triplet) of a known concentration for the corresponding reference compounds. The values for radiochemical yield (RCY), radiochemical purity (RCP) and molar activity (A_m) are given as mean values. This applies for all radiolabeled compounds described below.

Tetrazine labeling

*Labeling procedure for labelling [^{18}F]**6** starting from precursors **1, 2** and **5***

General procedure for the preparation of anhydrous [^{18}F]fluoride for radiolabeling. An anion exchange resin (Sep-Pak Light Waters Accell Plus QMA cartridge) was washed with EtOH (20 mL) and water (20 mL) and dried with air. Then the aqueous [^{18}F]fluoride solution was passed through this exchange resin and the resin eluted with 0.6 mL of a Kryptofix$_{222}$/K_2CO_3 solution: Kryptofix$_{222}$ (4,7,13,16,21,24-hexaoxa-1,10-diazabicyclo[8.8.8]hexa-cosane) (10 mg), K_2CO_3 (1.2 mg) in H2O (0.1 mL) and MeOH (0.5 mL). The resulting mixture was then gently concentrated to dryness at 100 °C by acetropic drying with 2 x 0.6 mL MeCN under a nitrogen stream for 20 min to give nocarrier-added K[^{18}F]F–K222 complex as a white semi-solid residue. All radioligands were produced from a starting activity of around 50–100 MBq.

Manual labeling of tetrazine [^{18}F]6 starting from precursor 1.[13, 14] The appropriate precursor (**1**) (2.4 mg, 0.008 mmol) was dissolved in DMF (0.3 mL) just before addition to the K [^{18}F]F–K222 complex. This mixture was stirred for 20 min at 130 °C before cooling to room temperature and quenching with H_2O (1 mL). The reaction mixture was analyzed by radio-HPLC. No radiolabeled compound [^{18}F]**6** could be detected.

Manual labeling of tetrazine [^{18}F]6 starting from precursor 5.[15, 16] The appropriate precursor (**2**) (3.6 mg, 0.008 mmol) was dissolved in DMF (0.3 mL) just before addition to the K [^{18}F]F–K222 complex. This mixture was stirred for 20 min at 130 °C before cooling to room temperature and quenching with H_2O (1 mL). The reaction mixture was analyzed by radio-HPLC. No radiolabeled compound [^{18}F]**6** could be detected.

Manual labeling of tetrazine [^{18}F]6 starting from precursor 2.[17] The appropriate precursor (**5**) (3.9 mg, 0.008 mmol) and TEMPO (1 mg) were dissolved in DMF (0.3 mL) just before addition to the K [^{18}F]F–K222 complex. This mixture was stirred for 20 min at 130 °C before cooling to room temperature and quenching with H_2O (1 mL). The reaction mixture was analyzed by radio-HPLC. No radiolabeled compound [^{18}F]**6** could be detected.

*Labeling procedure for labelling [^{18}F]**6** starting from precursor **4***

General procedure for the preparation of [^{18}F]fluoride for radiolabeling. Target water from the cyclotron containing ^{18}F-fluoride was loaded with a syringe into a QMA anion exchange cartridge (Chromafix 30-PS-HCO3) and the radioactivity of the trapped ^{18}F-fluoride was measured. The cartridge was washed with MeCN (1.0 mL). The cartridge was inverted and fitted with a female × female Luer adapter.

Manual labeling of tetrazine [^{18}F]6 starting from precursor 4.[18] The appropriate precursor (**4**) (1.64 mg, 8.7 µmol), imidazolium chloride (12 mg, 26 µmol, 3.0 eq.) and 150 µL of MeCN were added to a vial, and the resulting solution was drawn into a 1.0 mL polypropylene syringe. With the syringe, which contained the corresponding solution, the ^{18}F-fluoride was eluted into a 1 dram (4 mL) borosilicate vial. The cartridge was washed with DMSO (150 µL), followed by DMSO:MeCN (50 µL, 1:1 (v/v)) and the radioactivity

of the eluted solution was measured. The reaction vial, which contained 400 µL of the reaction mixture was heated at 125 °C for 30 min. The vial, which contained the reaction mixture was removed from the heat and was allowed to stand for 3 min at 23 °C. The reaction mixture was analyzed by radio-HPLC. No radiolabeled compound [^{18}F]**6** could be detected.

*Labeling procedure for labelling [^{18}F]**6** starting from stannane precursor **3** and **3a***

General procedure for the preparation of anhydrous [^{18}F]fluoride for radiolabeling. All QMA anion exchange cartridges (Sep-Pak Accell Plus QMA Plus Light, chloride form, Waters) were washed with EtOH (10 mL), 90 mg/mL KOTf (aq) (10 mL), water (10 mL) and dried with air before use. All C18 cartridges (Sep-Pak C18 Plus Short types) were washed with EtOH (10 mL), water (10 mL) and dried with air before use. Irradiated [^{18}O]water containing [^{18}F]F^- was passed through an anion exchange resin cartridge (Sep-Pak Accell Plus QMA Plus Light, chloride form). [^{18}F]Fluoride trapped on the QMA was then eluted with a mixture of KOTf (10 mg) and K_2CO_3 (50 ug) (optimized base amount, Table S1) in 550 µL water. The resulting mixture was then gently concentrated to dryness at 100 °C by azeotropic drying with 2xMeCN (0.6 mL), under a nitrogen stream for 20 min, to give no-carrier-added K[^{18}F]F complex as a white semi-solid residue.

Manual labeling of tetrazine [^{18}F]6 starting from precursor 3a. Labeling of compound [^{18}F]**6**, was performed using a method described previously, with minor modifications.[19] The boronic ester precursor **3a** (0.01 mmol) was dissolved in 0.8 mL DMA and added 0.1 mL of stock solutions of $Cu(OTf)_2$ (7.2 mg, 0.02 mmol in 0.1 mL DMA) and pyridine (12 µL, 0.15 mmol in 0.1 mL DMA). This mixture was added to the dried [^{18}F]FK and heated to 100 °C for 5 min (optimized conditions, Table S1). The mixture was cooled down for 2 min at r.t. before quenched with 1 mL of H_2O/0.1%TFA. Samples were analysed via radio-HPLC to determine the radiochemical conversion (RCC) of [^{18}F]**6**. All radio ligands were produced from a starting activity of around 100-50 MBq. This afforded [^{18}F]**6** in 35±3% radiochemical conversion (RCC) within 5 min (entry 3, Table S1).

Manual labeling of tetrazine [^{18}F]6 starting from precursor 3. Labeling of compound [^{18}F]**6**, was performed using a method described previously, with minor modifications.[19] The organotin precursor **3** (0.01 mmol) was dissolved in 0.8 mL DMA and added 0.1 mL of stock solutions of $Cu(OTf)_2$ (7.2 mg, 0.02 mmol in 0.1 mL DMA) and pyridine (12 µL, 0.15 mmol in 0.1 mL DMA). This mixture was added to the dried [^{18}F]FK and heated to 100 °C for 5 min (optimized conditions, Table S1). The mixture was cooled down for 2 min at r.t. before quenched with 1 mL of H_2O/0.1%TFA. Samples were analysed via radio-HPLC to determine the radiochemical conversion (RCC) of [^{18}F]**6**. All radio ligands were produced from a starting activity of around 100-50 MBq. The optimal radiofluorination conditions for **3** were as followed: 2 equiv. of $Cu(OTf)_2$, 15 equiv. of pyridine, 0.01 mmol of **3**, in 1 mL DMA at 100 °C. This afforded [^{18}F]**6** in 31±5% radiochemical conversion (RCC) within 5 min (entry 3, Table S1).

Table S1. *Radiolabeling optimization of Cu-mediated ^{18}F-fluorination reaction from stannane precursor **3** to [^{18}F]**6**. [a] Conditions: $Cu(OTf)_2$, pyridine, [^{18}F]KF (base, K_2CO_3), DMA, 100 ℃, 5 min. [b] Conditions: $Cu(OTf)_2$, pyridine, [^{18}F]KF (50 ug K_2CO_3), DMA, 5 min, temperature. [c] Conditions: $Cu(OTf)_2$, pyridine, [^{18}F]KF (50 ug K_2CO_3), DMA, time, 100 ℃. Radiochemical conversion (RCC) was determined by radio-HPLC (n=3).*

Base amount K_2CO_3 screen

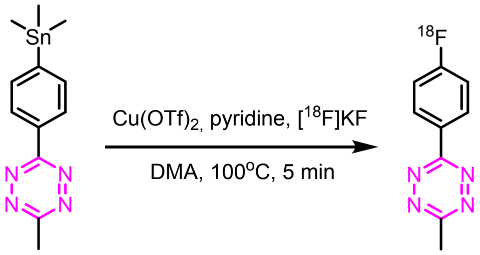

Entry	*Base amount (μg)* [a]	*RCC (%)*
1	25	29±8
2	50	31±5
3	100	19±2
4	150	20±5
5	200	9±1

Temperature screen

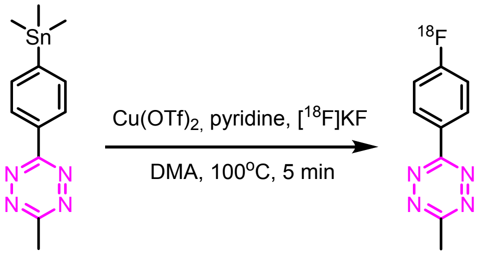

Entry	*Temperature (℃)* [b]	*RCC (%)*
1	60	0
2	80	16±8
3	100	31±5
4	115	20±3
5	140	14±2

Time screen

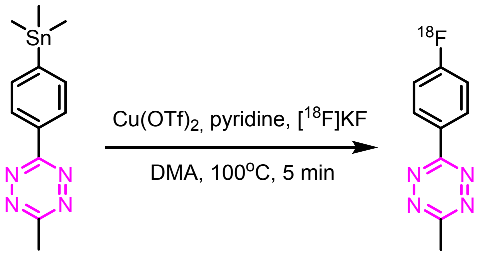

Entry	*Reaction time (min)* [c]	*RCC (%)*
1	1	0
2	3	29±2
3	5	31±5
4	10	15±5
5	15	16±2

Automated synthesis of [^{18}F]6. Automated synthesis was performed on a Scansys Laboratorieteknik synthesis module. The same procedure was used as in the optimization described above with minor differences. A solution of the organotin precursor **3** (0.01 mmol), $Cu(OTf)_2$ (7.2 mg, 0.02mmol), and pyridine (12 µL, 0.15 mmol) in 1 mL DMA was added to a reaction vial containing the dried [^{18}F]FK and the reaction allowed to proceed at 100 °C for 5 minutes. The solution was then cooled to 40 °C with compressed air before quenched with 2 mL of H_2O/0.1% TFA. The crude reaction was then purified via semi-preparative HPLC (Thermo Fisher UltiMate 3000) with a C-18 column (Luna 5 µm C18(2) 100 Å, 250 mm x 10 mm) using an isocratic method based on H_2O/MeCN mixture (40/60 v/v) and a flowrate of 4 mL/min. Fraction was collected in 60 mL H_2O and applied to a Sep-Pak C18 Plus and subsequently formulated by eluting with 1 mL EtOH into 0.1 M phosphate buffer (pH 7.4) for formulation to a final concentration of 100-70 MBq/mL. The automated synthesis including purification and concentration[^{18}F]fluoride, labeling, HPLC separation and formulation was carried out within 90 minutes. Tz [^{18}F]**6** was afforded in 25% RCY d.c., a RCP of 99% and an A_m: 190 ± 10 GBq/µmol (d.c) (n=3) (Table 2). UV detection at 254 nm, rt (radio): 7.57 min, semi-preparative HPLC r.t.: 675 seconds. A typical activity yield was 2.5–3 GBq starting from ~12 GBq fluoride-18. Radiochemical purity (RCP), radiochemical yield (RCY) and molar activity (A_m) of [^{18}F]**6** were determined according to the *General information* described above. Radio-HPLC and UV HPLC analytical traces of the formulated product can be found in *Section S7: Analytical Radio-HPLC and semi-preparative HPLC chromatograms of formulated [^{18}F]Tzs.*

*Labeling procedure for labelling [^{18}F]**6–13** Tzs starting from the appropriate stannane precursor.*

Manual labeling of [^{18}F]6–13 Tzs. The synthesis was performed according to the *Labeling procedure for labelling [^{18}F]**6** starting from stannane precursor **3*** described above. Radiochemical conversions (RCCs) were determined according to the *General information* described above (Table S2).

Automated synthesis of [^{18}F]6–13 Tzs. The synthesis was performed according to the *Labeling procedure for labelling [^{18}F]**6** starting from stannane precursor **3*** described above. Radiochemical purity (RCP) and radiochemical yield (RCY) were determined according to the *General information* described above (Table S2). Different H_2O/MeCN (v/v) solvent mixtures were used for the semi-preparative HPLC purification of each radiolabeled ^{18}F-Tz (Table S2). Radio-HPLC and UV HPLC analytical traces of the formulated product can be found in *Section S7: Analytical Radio-HPLC and semi-preparative HPLC chromatograms of formulated [^{18}F]Tzs.*

Table S2. *Radiolabeling with Cu-mediated fluorination reaction of higher reactive tetrazines. [a] Radiochemical conversion (RCC) and Radiochemical purity (RCP) were determined by radio-HPLC (n=3) (Section S7: Analytical Radio-HPLC and semi-preparative HPLC chromatograms of formulated [^{18}F]Tzs) . [b] Radiochemical yield (RCY) was decay corrected to the starting amount of radioactivity received from the cyclotron and the isolated product with formulation step (n=3). [c] No product formed or could not be isolated.*

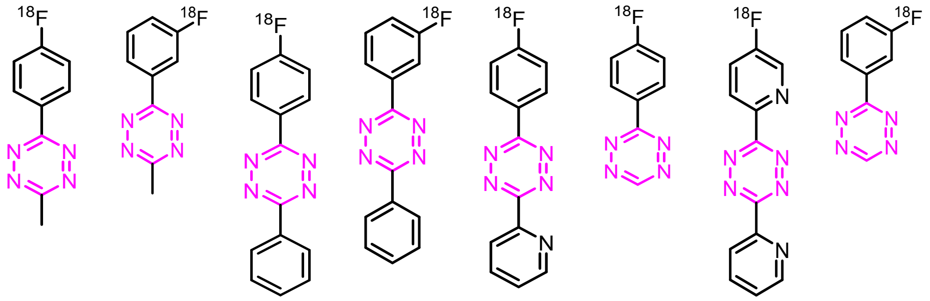

Compound	[^{18}F]**6**	[^{18}F]**7**	[^{18}F]**8**	[^{18}F]**9**	[^{18}F]**10**	[^{18}F]**11**	[^{18}F]**12**	[^{18}F]**13**
RCC (%) Radio-HPLC (n=3) [a]	31±5	28±1	30±5	31±2	-[c]r	18±4	-[c]	12±1
RCY (%) EOS (d.c.) (n=3) [b]	25±1	26±2	23±2	24±3	-[c]	15±3	-[c]	11±3
RCP (%) radio-HPLC (n=3) [a]	≥99%	≥99%	≥99%	≥99%	-[c]	≥99%	-[c]	≥99%
R.t. Analytical HPLC (minutes)	7.57	7.67	9.63	9.70	-[c]	6.97	-[c]	6.87
Solvent Semi-prep HPLC H_2O/ MeCN (v/v)	40/60	40/60	30/70	30/70	-[c]	50/50	-[c]	50/50
R.t. Semi-prep HPLC (seconds)	675	580	980	790	-[c]	1050	-[c]	1150

*Labeling procedure for labelling [^{18}F]**14–18** Tzs starting from the appropiate stannane precursor.*

General procedure for the manual labeling of [^{18}F]14–18 *Tzs*. The synthesis was performed according to the *Labeling procedure for labelling [^{18}F]**6** starting from stannane precursor **3*** described above. Radiochemical conversion (RCC), of all radiolabeled compounds were determined according to the *General information* described above (Table S3).

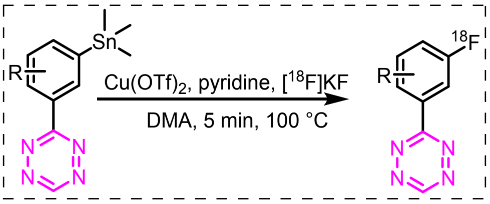

Table S3. *Study of the RCCs when including in the aromatic ring different linkers. [a] Stannane precursor could not be synthesized. [b] No tetrazine formation detected. [c] Decomposition during Cu-mediated ^{18}F-fluorination reaction. [d] RCCs were determined by radio-HPLC (n = 3).*

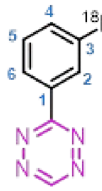

Linkers	***Compound***	***Position***		
		4 (*-p*)	5 (*-m*)	6 (*-o*)
-CH$_3$	[^{18}F]**14**	-[a]	14±3	-[c]
-OCH$_3$	[^{18}F]**15**	4±1	17±3	-[c]
-NHCOCH$_3$	[^{18}F]**16**	-[a]	30±4	-[b]
-CONH$_2$	[^{18}F]**17**	-[a]	24±2	-[b]
-CONHCH$_3$	[^{18}F]**18**	-[a]	20±3	-[b]

General procedure for the automated synthesis of *[^{18}F]14–18* Tzs. The synthesis was performed according to the *Labeling procedure for labelling [^{18}F]**6** starting from stannane precursor **3*** described above, for those tetrazines that could be detected with sufficient radiochemical conversion (RCC) in the manual synthesis experiments (Table S4). Radiochemical conversion (RCC), radiochemical purity (RCP), radiochemical yield (RCY) and molar activity (A_m) of all radiolabeled compounds were determined according to the *General information* described above. Radio-HPLC and UV HPLC analytical traces of the formulated product can be found in *Section S7: Analytical Radio-HPLC and semi-preparative HPLC chromatograms of formulated [^{18}F]Tzs.*

Table S4. *Radiolabeling with Cu-mediated fluorination reaction. Study of the RCYs when including in the aromatic ring different linkers. [a] Radiochemical conversion (RCC) and Radiochemical purity (RCP) were determined by radio-HPLC (n=3) (*Section S7: Analytical Radio-HPLC and semi-preparative HPLC chromatograms of formulated [^{18}F]Tzs*). [b] Radiochemical yield (RCY) was decay corrected to the starting amount of radioactivity received from the cyclotron and the isolated product without formulation step (n=3).*

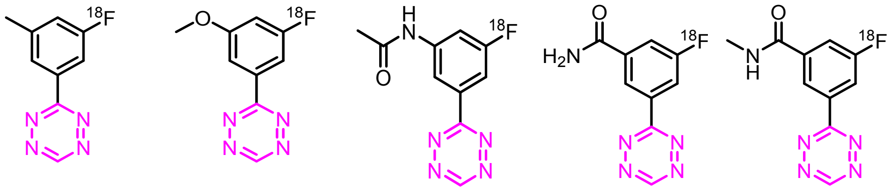

Compound	[^{18}F]**14-*m***	[^{18}F]**15-*m***	[^{18}F]**16-*m***	[^{18}F]**17-*m***	[^{18}F]**18-*m***
RCC (%) Radio-HPLC (n=3) [a]	14±3	17±3	30±4	24±2	20±3
RCY (%) EOS (d.c.) (n=3) [b]	11±6	15±4	15±1	15±3	13±3
RCP (%) radio-HPLC (n=3) [a]	≥99%	≥99%	≥99%	≥99%	≥99%
A_m (GBq/umol) (d.c.)	108±15	145±10	142±20	102±17	114±22
R.t. Analytical HPLC (minutes)	7.35	7.67	6.23	5.51	5.93
Solvent Semi-prep HPLC H_2O/MeCN (v/v)	40/60	40/60	65/35	77/23	75/25
R.t. Semi-prep HPLC (seconds)	720	750	1150	790	990

*Labeling procedure for labelling [^{18}F]**21** Tz starting from stannane precursors **20***

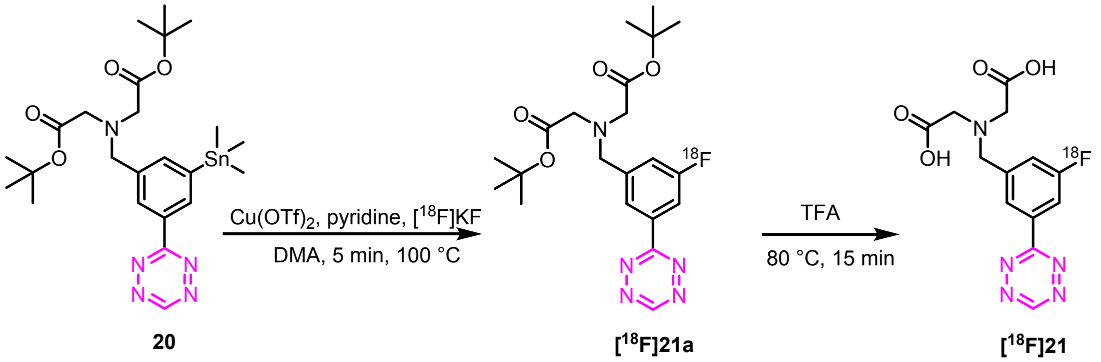

Scheme S26. *Labeling of [^{18}F]**21** Tz. Cu(OTf)$_2$, pyridine, [^{18}F]KF, DMA, 5 min, 100 °C; TFA, MeCN, 100 ºC, 15 min, 11% RCC.*

Manual labeling of [^{18}F]21 Tz. The same procedure was used as describe above, with minor modifications. The appropriate precursor **20** (0.01 mmol) was dissolved in 0.8 mL DMA and added 0.1 mL of stock solutions of Cu(OTf)$_2$ (7.2 mg, 0.02 mmol in 0.1 mL DMA) and pyridine (12 µL, 0.15 mmol in 0.1 mL DMA). This mixture was added to the dried [^{18}F]FK and heated to 100 °C for 5 min. The mixture was cooled down before quenched with 1 mL of H_2O/0.1% TFA. The reaction mixture was diluted with 20 mL of H_2O and put through a Sep-Pak Plus 18 cartridge (SPE) preconditioned by flushing with 10 mL of EtOH followed by 10 mL of H_2O. The SPE was eluted with 3 mL of MeCN into a vial containing 1 mL TFA. The mixture containing the protected product was heated during 15 minutes at 80 °C for fully deprotection. The mixture was cooled down and the sample was analysed via radio-HPLC to detect the radiolabeled product [^{18}F]**21.**

General procedure for the automated synthesis of [^{18}F]21. Automated synthesis was performed on a Scansys Laboratorieteknik synthesis module. The same procedure was used as in manual synthesis with minor differences. A solution of the organotin precursors **20** (0.01 mmol), Cu(OTf)$_2$ (7.2 mg, 0.02 mmol), and pyridine (12 µL, 0.15 mmol) in 1 mL DMA was added to a reaction vial containing the dried fluoride and the reaction allowed to proceed at 100 °C for 5 minutes. The solution was then cooled to 40 °C with compressed air before quenched with 2 mL of H_2O/0.1% TFA. The reaction mixture was put through a Sep-Pak Plus 18 cartridge (SPE) preconditioned by flushing with 10 mL of EtOH followed by 10 mL of H_2O. The SPE was washed with 10 mL water and dried with air before eluted with 3 mL of MeCN into a vial containing 1 mL TFA. The mixture containing the protected radiolabeled product was heated during 10 minutes at 80 °C for fully deprotection. The resulting mixture was then gently concentrated at 100 °C under a nitrogen stream for 20 min. The solution was then cooled to 40 °C with compressed air before the addition of 3 mL of water to resubilize the reaction mixture. The crude reaction was then purified via semi-preparative HPLC (Thermo Fisher UltiMate 3000) with a C-18 column (Luna 5 µm C18(2) 100 Å, 250 mm x 10 mm) used an isocratic method (15% EtOH in water 0.1% TFA, flowrate 4 mL/min).

The isolated product collected from the HPLC, 600–700 MBq starting from ~12 GBq fluoride-18, was formulated with 0.1 M phosphate buffer (pH 7.4) for formulation to a final activity concentration of 100-70 MBq/mL for injection. The automated synthesis including purification and concentration of [^{18}F]fluoride, labeling, HPLC separation and formulation was carried out within 90 minutes. Tz [^{18}F]**21** was afforded in 11±3% RCY (d.c.) over 2 steps, a RCP of 99% and an A_m: 134±22 GBq/µmol GBq/µmol (d.c) (n=4) (Table 2). UV detection at 254 nm, r.t. (radio): 4.86 min, semi-preparative HPLC r.t.: 580 seconds. Radiochemical purity (RCP), radiochemical yield (RCY) and molar activity (A_m) of [^{18}F]**21** were determined according to the *General information* described above. Radio-HPLC and UV HPLC analytical traces of the formulated product can be found in *Section S7: Analytical Radio-HPLC and semi-preparative HPLC chromatograms of formulated [^{18}F]Tzs.*

[^{18}F]21 stability and TCO click ability.

The stability of the formulated tracer [^{18}F]**21** was tested in PBS at room temperature for 4 h post-injection (n=4). The Tz-scaffold was analyzed by analytical-HPLC by comparing the Tz-region peak (Table S5). The intact Tz was also analyze by the TCO click ability, as follow. To 50 uL of ((1R,8S,9r,E)-bicyclo[6.1.0]non-4-en-9-yl)methanol (**s-TCO**) (1 mg/mL) were added 500 uL of PBS and 100 uL of the formulated [^{18}F]**21**. In given time intervals 2 µL of the solution was withdrawn and quenched with 20 µL of a 3 mg/mL solution of 3,6-di(pyridin-2-yl)-1,2,4,5-tetrazine. The TCO click ability of [^{18}F]**21** was determined by analytical-HPLC (Figure S1). The fraction of [^{18}F]**21** that reacted with the TCO was determined by integrating the radio-HPLC peaks from the chromatogram. The percentage of intact Tz-scaffold was determined by comparing the Tz-region of the radio-HPLC before and after addition of TCO agent. [^{18}F]**21** reacted immediately with the s-TCO.

Table S5. *Stability studies of formulated [^{18}F]**21** in PBS at r.t. during 4 h p.i. and s-TCO click ability of [^{18}F]**21** to verify the intact Tz-scaffold.*

*[^{18}F]**21***

Time	*Stability*	*TCO click ability*
15 min	≥ 99%	100%
10 min	≥ 99%	100%
30 min	≥ 99%	100%
1 h	≥ 99%	100%
2 h	99%	100%
3 h	98%	100%
4 h	98%	100%

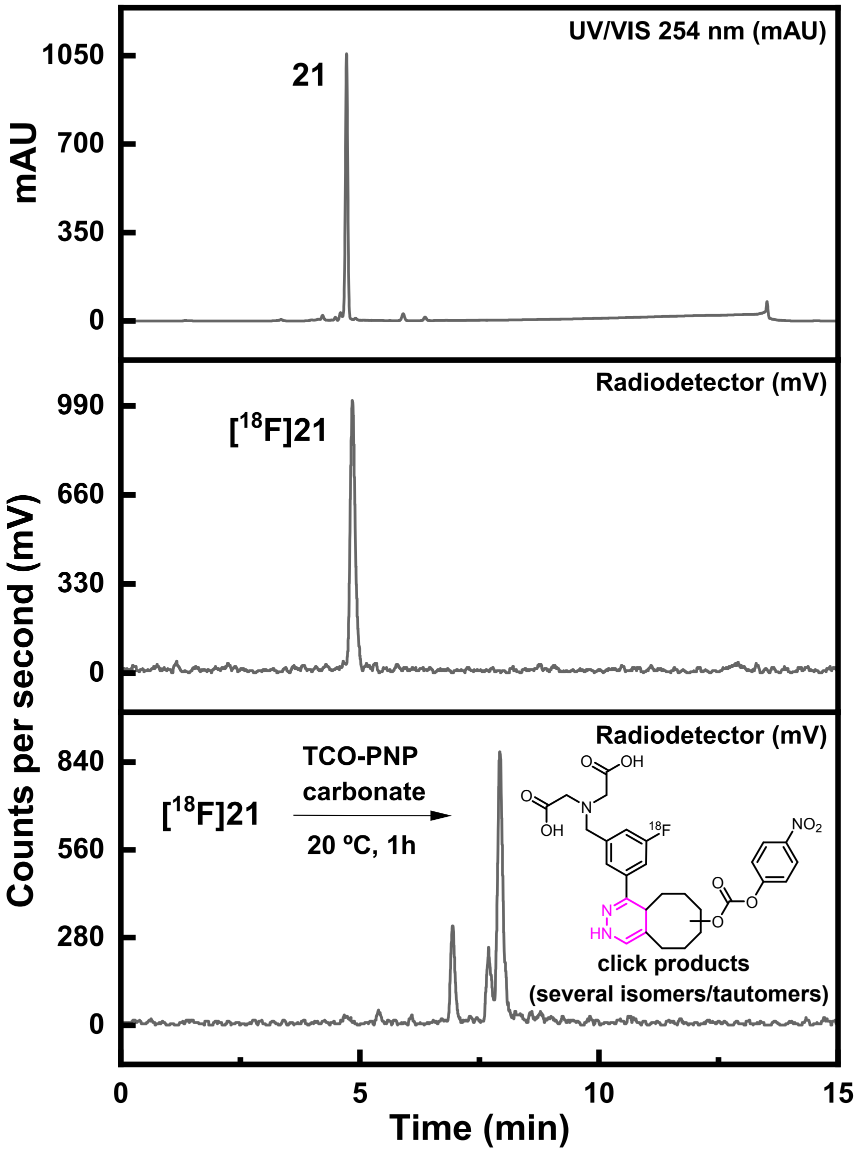

Figure S1. *Analytical-HPLC of reference compound **21** (UV/Vis, 254 nm), radio-HPLC of purified [^{18}F]**21** and ligation product after click reaction with s-TCO, 1 h p.i. HPLC conditions: Luna 5 µm C18(2) 100 Å, 150 mm x 4.6 mm, H_2O/MeCN gradient elution.*

Determination of Sn and Cu-Content.

The contents of tin and copper were determined by PerkinElmer Elan 6100DRC ICP-MS. Solutions of the formulated tracer **[^{18}F]21** in PBS were measured. The sample was analyzed with quantitative method of Cu and Sn. Standard: multielement standard Merck VI and Sn standard for ICP-MS. Software for instrument control, data collection, calibration and quantification: PerkinElmer Elan version 3.3.

The residual amounts of Cu and Sn in the final solution were well below the allowed limits specified in the ICH Guidelines[20] (41-60 and 2.3-3.0 µg/L vs. 300 and 600 µg/day, respectively) (n=4).

Section S4: Antibody Production and Modification

The antibody and the modified antibody were kindly provided by Tagworks and prepared according to the literature.[21-23]

Section S5: *In Vitro* Studies

Reaction kinetics

Reactivities of the fluoro-Tz **6**-**13** (reference compounds for the radiolabeled analogs) in the IEDDA reaction with TCO were determined by pseudo-first order measurements in 1,4-dioxane and/or acetonitrile (depending on stability, solubility and availability) at 25 °C and in Dulbecco's phosphate buffered saline (DPBS) at 37 °C by stopped flow spectrophotometry.

Solutions of TCO[24] in anhydrous 1,4-dioxane or acetonitrile and axTCO-PEG_4[25] (**S1**) in DPBS (10 mM) were prepared at an approximate concentration above 2 mM. ***Note***: The axially configured axTCO is also used as TCO tag on modified CC49.[23] The exact concentration was determined by absorbance titration with 3,6-dimethyltetrazine[26] (**S2**) (extinction coefficient 510 $M^{-1}cm^{-1}$ at 520 nm), quantifying the decrease in tetrazine absorbance upon reaction with TCO or **S1**. These initial stock solutions were diluted before stopped-flow analysis to reach a final TCO concentration of 2 mM.

Stock solutions of tetrazines were prepared in DMSO at a concentration of 10 mM. Serial dilution into 1,4-dioxane or acetonitrile (TCO) or DPBS (**S1**) was used to prepare solutions for stopped-flow analysis at a Tz concentration of 100 µM.

Stopped-flow measurements were performed using an SX20-LED stopped-flow spectrophotometer (Applied Photophysics) equipped with a 535nm LED (optical pathlength 10mm, full width half-maximum 34nm) to monitor the characteristic tetrazine visible light absorbance (520-540 nm). The reagent syringes were loaded with solutions of the Tz and TCO or **S1** and the instrument was primed. Subsequent data were collected in triplicate to sextuplicate for each tetrazine. Reactions were conducted at 25 °C (1,4-dioxane or acetonitrile) or 37 °C (DPBS) and recorded automatically at the time of acquisition.

Data sets were analyzed by fitting an exponential decay using Prism 6 (GraphPad) to calculate the observed pseudo-first order rate constants that were converted into second order rate constants by dividing through the concentration of excess TCO compound.

Table S6. *Second order rate constants (k_2, mean values, SD<7%, n>3) determined for the reaction of Tz **6**-**13** with TCO in 1,4-dioxane and/or acetonitrile (25 °C).*

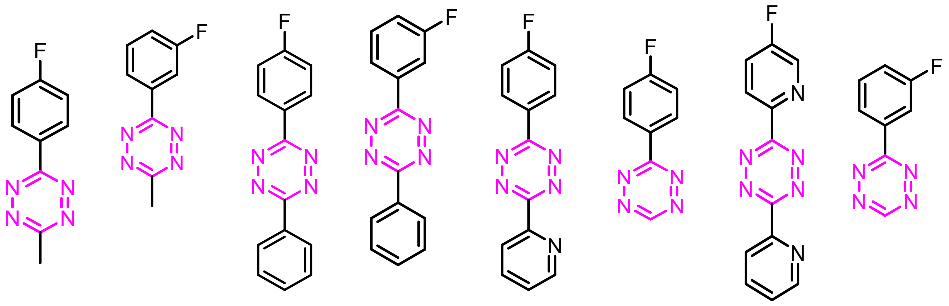

Compound	**6**	**7**	**8**	**9**	**10**	**11**	**12**	**13**
k_2 in dioxane [$M^{-1}s^{-1}$]	2.3	3.2	*n.d.*	*n.d.*	22	160	210	220
k_2 in MeCN [$M^{-1}s^{-1}$]	*n.d.*	14	18	30	110	*n.d.*	*n.d.*	*n.d.*
Relative reactivity	1	1.4[a]	1.8[b]	3.0[b]	10[a]<br>11[b]	70[a]	91[a]	96[a]

[a] calculated based on k_2 (dioxane) for **6**

[b] calculated based on k_2 (acetonitrile) and a relative reactivity of 1.4 for Tz **7**

Table S7. *Second order rate constants (k_2, mean values, SD<7%, n>3) for the reaction of substituted 3-fluorophenyl-Tz with TCO in acetonitrile (25 °C) and with axTCO-PEG$_4$ (**S1**) in DPBS (37 °C).*

Tetrazine		*Compound*			*Rate constant (k_2) [$M^{-1}s^{-1}$] in DPBS (37 °C)*			*Rate constant (k_2) [$M^{-1}s^{-1}$] in MeCN (25 °C)*		
Structure	Substituent (R)	Position			Position			Position		
		4	5	6	4	5	6	4	5	6
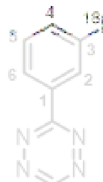	-CH_3	14-*p*	14-*m*	14-*o*	n.d.	79,000	n.d.	n.d.	1060	n.d.
	-OCH_3	15-*p*	15-*m*	15-*o*	n.d.	88,000	n.d.	n.d.	1290	n.d.
	-$CONH_2$	16-*p*	16-*m*	16-*o*	n.d.	93,000	n.d.	n.d.	1920	n.d.
	-$NHCOCH_3$	17-*p*	17-*m*	17-*o*	n.d.	99,000	n.d.	n.d.	1380	n.d.
	-$CONHCH_3$	18-*p*	18-*m*	18-*o*	n.d.	88,000	n.d.	n.d.	1850	n.d.
	-COOH	-	19	-	-	91,000	-	-	1980	-
	-$CH_2N(CH_2COOH)_2$	-	21	-	-	82,000	-	-	*n.d.*	-

[a] Tz could not be prepared.

Blocking assay[25]

Establishing tumor xenografts in mice. All animal experiments were performed under a protocol approved by the Animal Research Committee of the Danish Ministry of Environment and Food (license nr: 2016-15-0201-00920), and the Animal Ethics Committee of the University of Copenhagen, and in compliance with the guidelines in Directive 2010/63/EU of the European Parliament on the protection of animals used for scientific purposes. Five weeks old female nude BALB/c mice (Charles River, Sulzfeld, Germany) were upon arrival allowed to acclimatize for one week at the animal facility At all times the animals had access to water and chow ad libitum. Human colon cancer cell line (LS174T; obtained from ATCC) was cultured in minimum essential medium (MEM) supplemented with 10% fetal bovine serum, 1% *L*-glutamine, 1% sodium pyruvate, 1% non-essential amino acids, and 1% penicillin-streptomycin at 37 °C and 5% CO_2. At a confluence of 70–90% the cells were harvested by trypsinization, washed and resuspended in PBS. Subcutaneous tumors were established in the flank of the nude BALB/c mice by inoculation of ~ 5 × 10^6 LS174T cells in 100 µL PBS. The tumors were allowed to grow for 7–10 days, and the size was monitored using caliper measurements (tumor volume = ½(length x width^2).

Blocking experiments. The tumor-bearing mice were matched into groups based on their tumor (tumor volumes of ~ 100–300 mm^3, *n* = 3 in each group), and injected (i.v.) with CC49-TCO (100 µg/100 µL, ~7 TCO/mAb). Three days later, the animals were first administered (i.v.) with non-radioactive Tz (39 nmol/100 µL), and 1 h later they received a second injection (i.v.) with [^{111}In]**DOTA-Tz** (~13 MBq/100 µL, 3.9 nmol) via the tail vein. Tz [^{111}In]**DOTA-Tz** was radiolabeled as previously described. [1] Additionally, a group of mice injected exclusively with [^{111}In]**DOTA-Tz** without any previous administration of Tzs, was used as a reference. The change in uptake of [^{111}In]**DOTA-Tz** induced by the different Tzs where normalized to this group of animals. In addition, **41**, the non-radioactive precursor of [^{111}In]**DOTA-Tz,** was also included as a positive control.The mice were euthanized after 22 h after [^{111}In]**DOTA-Tz** administration and different tissues were resected, weighted, and the radioactivity measured using a gamma counter (Wizard2, Perkin Elmer). Data was corrected for decay, tissue weight and injected amount of radioactivity.

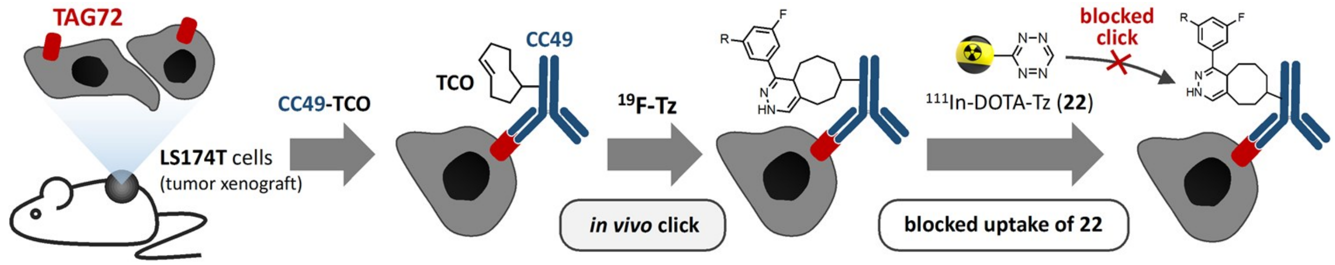

Figure S2. *Schematic illustration of the blocking assay. Tumor-bearing mice were first injected with CC49-TCO, 72 h before administration of the non-radioactive Tz. After 1 h, the* ^{111}*In-labeled Tz ([*^{111}*In]**DOTA-Tz**, (**22**) was injected and ex vivo biodistribution was carried out 22 p.i. in order to determine the blocking effect caused by the non-radioactive Tz.*

Ex vivo biodistribution data for all evaluated Tzs (**13**, **14–18-*m***, **19**, **21**) are displayed below - Ability of ^{19}F-tetrazines to block ^{111}In-DOTA-tetrazine from the tetrazine ligation. The relationship between cLogD$_{7.4}$ and blocking effect was analysed using Persson's correlation. Results were considered significant when $p < 0.05$.

Table S8. *Indirect evaluation of the ability of cold* ^{19}*F-tetrazines to block the participation of [*^{111}*In]**DOTA-Tz** in the tetrazine ligation reaction, in an in vivo tumour blocking assay using pretargeted imaging. [a] Calculated distribution coefficient at physiological pH (7.4) in Chemicalize software. [b] Second order rate constants (*k_2*, mean values, SD<7%, n>3) for the reaction of substituted 3-fluorophenyl-Tz with TCO in acetonitrile (25 °C) and with axTCO-PEG*$_4$ *(**S1**) in DPBS (37 °C).*

Compound	*Tetrazine structure*	*clogD7.4* [a]	*TPSA*	*Rate constant [*M^{-1} s^{-1}*]* [b]	*Normalized blocking effect*	*Ability of Tzs to block (%)*
13	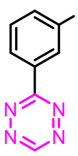	0.73	51.56	67,000	67	33
14-m	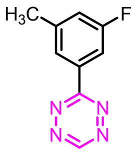	1.23	51.56	79,000	55	45
15-m	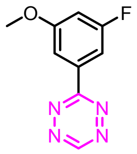	0.53	60.79	88,000	45	55
16-m	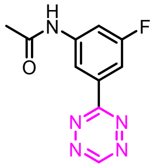	0.06	80.66	93,000	41	59

17-m	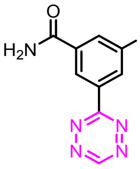	-0.49	94.65	99,000	40	60
18-m	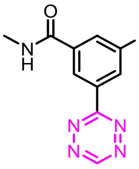	-0.26	80.66	88,000	41	59
19	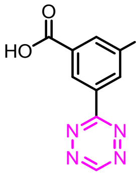	-3.09	88.86	91,000	35	65
21	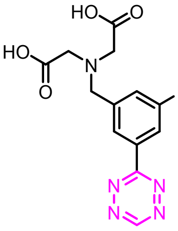	-6.93	129.40	82,000	10	90
DOTA-Tz	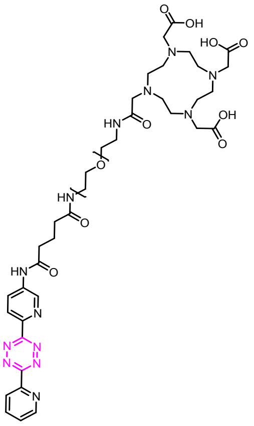	-4.13	362	73,000	1	99

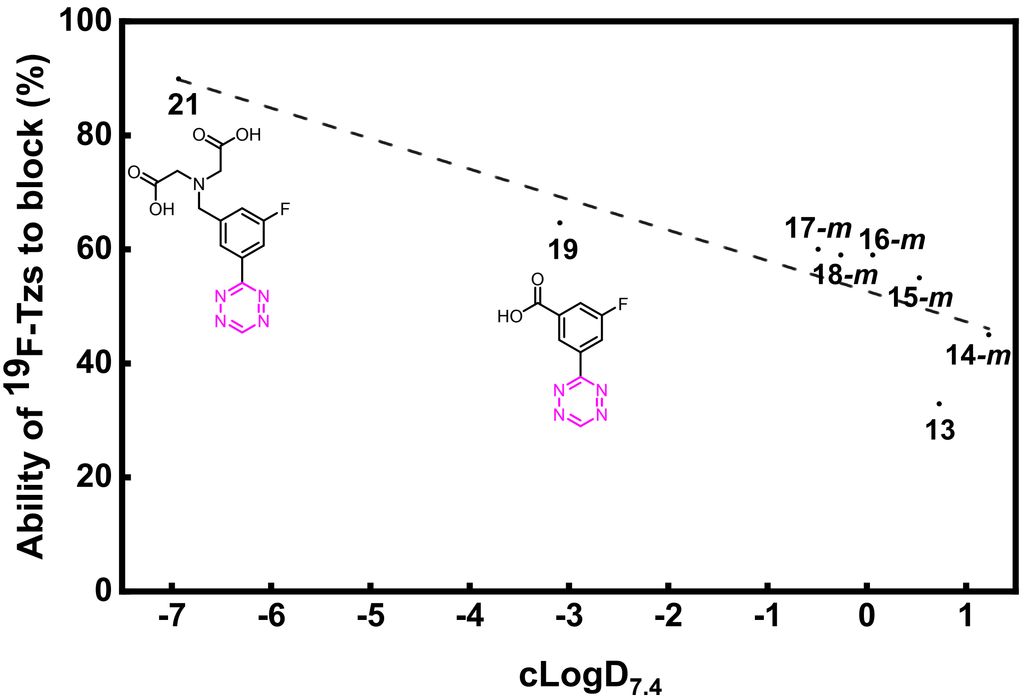

Graph S1. *Ability of* [19]*F-Tzs (**13, 14–18-m, 19, 21**) to block [*[111]*In]**DOTA-Tz** from participating in the tetrazine ligation reaction, in an in vivo pretargeted model-screening assay (n=3).*

Section S6: *In Vivo* Studies

Pretargeted PET imaging of TCO-modified mAb (CC49-TCO) with [^{18}*F]**21***

LS174T tumor xenografts in mice were established using the same procedure as for the blocking assay and ex vivo studies (see previous section). Tumor-bearing animals were matched into 2 groups based on their tumor volume (tumor volumes of ~ 60–180 mm^3, n = 3-4 in each group) and were administered either with CC49-TCO (100 µg/100 µL, ~7 TCO/mAb) or CC49 (100 µg/100 µL). After 72 h, the animals were injected via the tail vein with [^{18}F]**21** (2.86 ± 0.99 MBq /100 µL). The tracer was allowed to distribute and the mice were PET/CT scanned (Inveon, Siemens Medical Solutions) after 1 h (PET acquisition: 5 min, energy window of 350–650 KeV and a time resolution of 6 ns; CT scan: 360 projections, 65 kV, 500 µA and 400 ms). During scans the animals were placed on a heating pad to avoid temperature chenges and anaesthetized by breathing sevoflurane (3%). After the scan animals were euthanized and ex vivo biodistribution performed as described previously for the ex vivo blocking assay (see previous section).

Signograms from PET scans were reconstructed using a 3-dimensional maximum a posteriori algorithm with scatter correction and CT-based attenuation correction. PET and CT images were co-registered and tissue uptake analyzed using Inveon software (Siemens). The mean percentage of injected dose per grams (%ID/g) was extracted by manually creating regions of interest (ROI) on fussed PET/CT images. Differences in tumor uptake between mice pretreated with CC49-TCO and CC49 was analysed using Welch's t-test. Results were considered significant when $p < 0.05$.

Table S9. Image derived uptake values of **[^{18}F]21** in pretargeted experiments

	CC49-TCO (mean %ID/g) *n* = 3	***CC49*** (mean %ID/g) *n* = 3
Tumor	0.99 ± 0.15	0.05 ± 0.04
Heart	1.15 ± 0.16	0.04 ± 0.00
Muscle	0.09 ± 0.04	0.02 ± 0.02

Table S10*. Wellcounter data of ex vivo uptake values of* ***[^{18}F]21*** *in pretargeted experiments.*

	CC49-TCO (mean %ID/g) *n* = 3	***CC49*** (mean %ID/g) n = 3
Tumor	1.85 ± 0.21	0.06 ± 0.02
Blood	1.25 ± 0.02	0.06 ± 0.00
Heart	0.27 ± 0.01	0.02 ± 0.00
Lung	0.51 ± 0.03	0.06 ± 0.00
Liver	0.49 ± 0.02	0.10 ± 0.01
Spleen	0.32 ± 0.02	0.02 ± 0.00
Kidney	0.47 ± 0.02	0.26 ± 0.01
Muscle	0.12 ± 0.03	0.03 ± 0.02

Section S7: Analytical Radio-HPLC and semi-preparative HPLC chromatograms of formulated [^{18}F]Tzs

Radio-HPLC tracer of the formulated [^{18}F]Tzs synthesized following the general procedure for the automated synthesis of the tetrazines describe in Section S3, with authentic UV ^{19}F-references overlaid are shown below. All samples were run on an analytical-HPLC method: Thermo Fisher UltiMate 3000 with a C-18 column (Luna 5 µm C18(2) 100 Å, 150 mm x 4.6 mm). Eluents: A, H_2O with 0.1% TFA; B, MeCN with 0.1% TFA. Gradient from 100% A to 100% B over 12 minutes, back to 100% A over 3 min, flow rate 2 mL/min. Detection by UV absorption at λ = 254 nm on a UVD 170U detector. The solid red line indicates the radio-HPLC [^{18}F]trace and the solid black line indicates the UV trace for the cold reference compound. Semi-preparative HPLC was performed on a Luna 5µ C18(2) (100Å 250 x 10 mm) column using H_2O/MeCN (v/v) mixtures as eluent (SI, Section S3), flow rate 4 mL/min in the Scansys module.

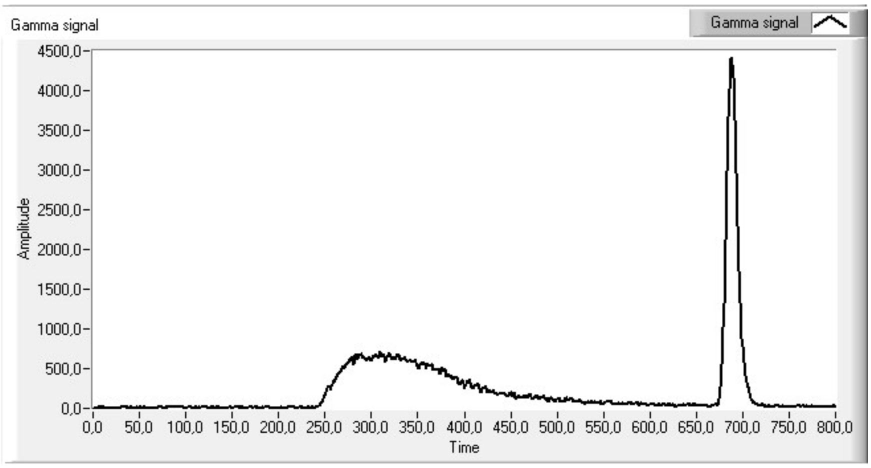

Figure S3. *Semi-preparative HPLC chromatogram for* ***[^{18}F]6*** *(R_t = 675 sec).*

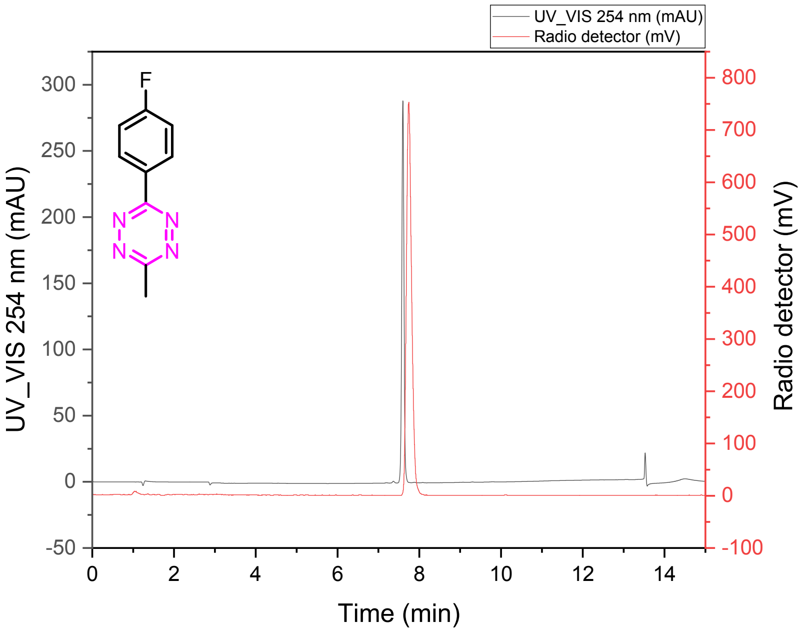

Figure S4. *Analytical-HPLC chromatogram of reference compound* ***6*** *(UV/Vis, 254 nm) and radio-HPLC chromatogram of formulated* ***[^{18}F]6*** *(Rt = 7.57 minutes).*

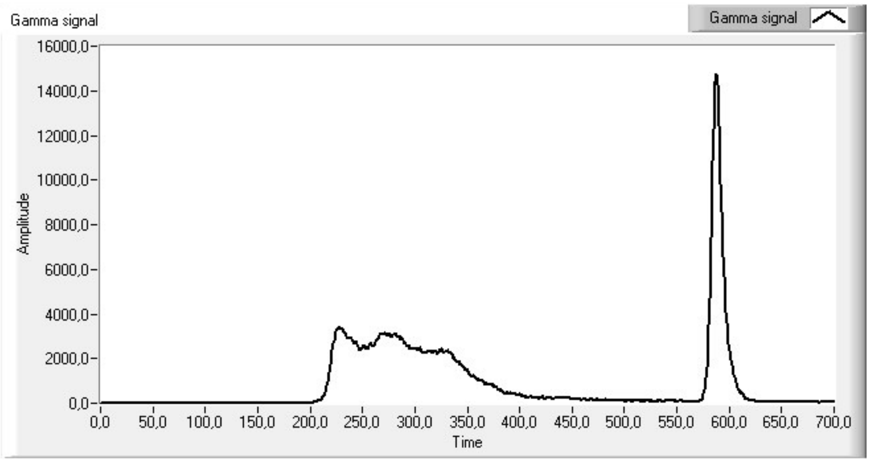

Figure S5. *Semi-preparative HPLC chromatogram for **[18F]7** (R_t = 580 sec).*

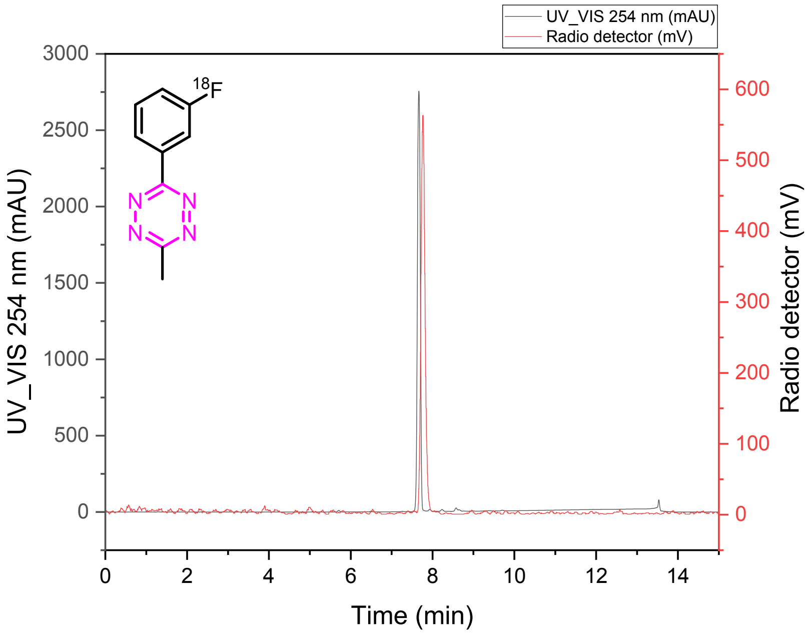

Figure S6. *Analytical-HPLC chromatogram of reference compound **7** (UV/Vis, 254 nm) and radio-HPLC chromatogram of formulated **[18F]7** (Rt = 7.67 minutes).*

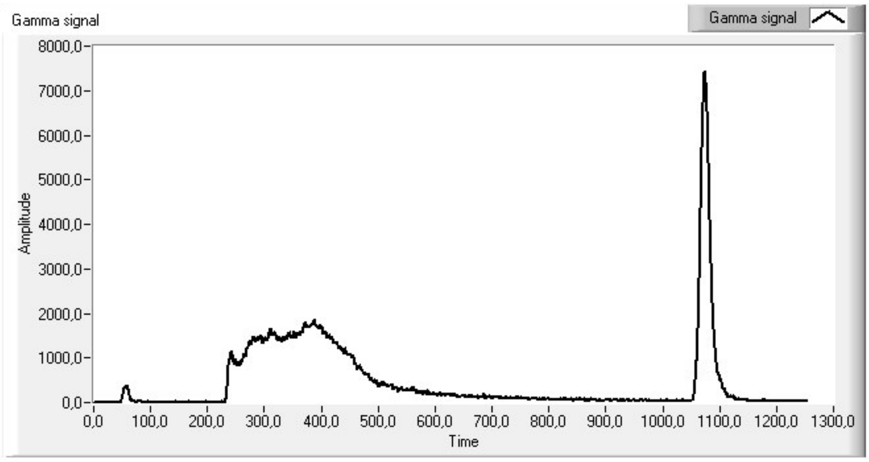

Figure S7. *Semi-preparative HPLC chromatogram for **[^{18}F]8** (R_t = 1050 sec).*

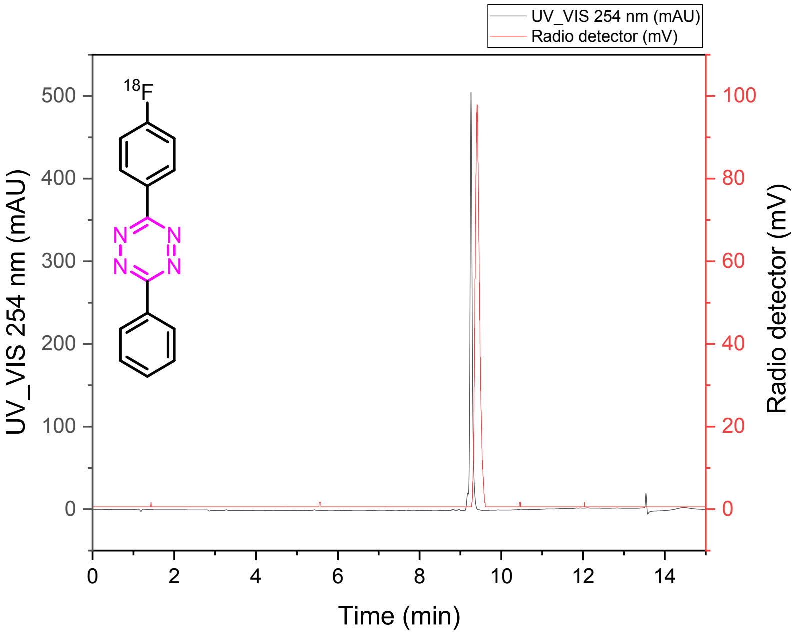

Figure S8. *Analytical-HPLC chromatogram of reference compound **8** (UV/Vis, 254 nm) and radio-HPLC chromatogram of formulated **[^{18}F]8** (Rt = 9.63 minutes).*

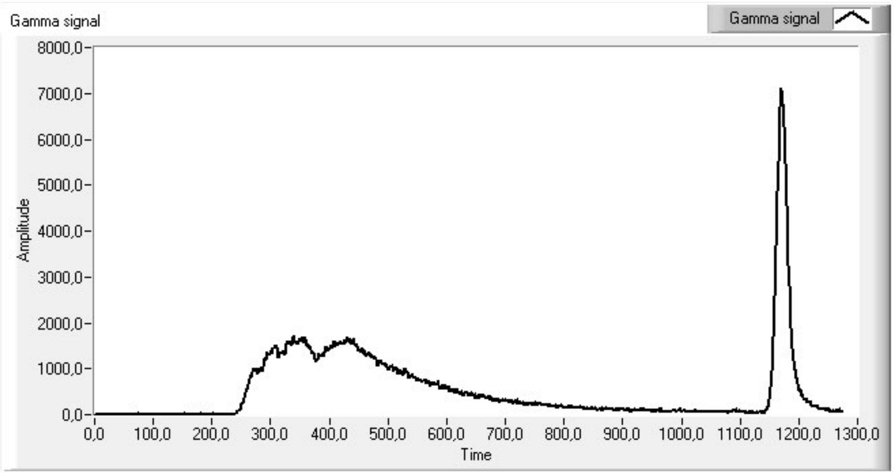

Figure S9. *Semi-preparative HPLC chromatogram for* ***[¹⁸F]9*** *(R_t = 1050 sec).*

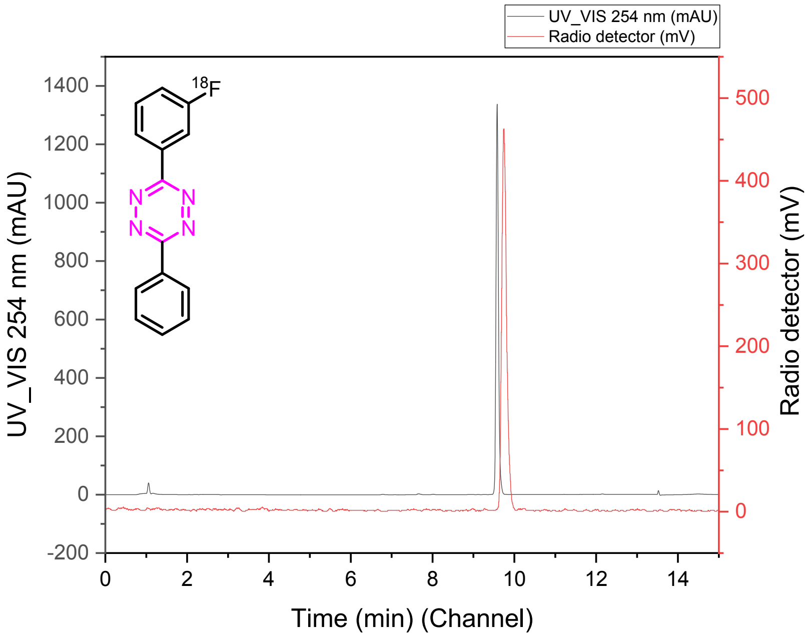

Figure S10. *Analytical-HPLC chromatogram of reference compound* ***9*** *(UV/Vis, 254 nm) and radio-HPLC chromatogram of formulated* ***[¹⁸F]9*** *(Rt = 9.70 minutes).*

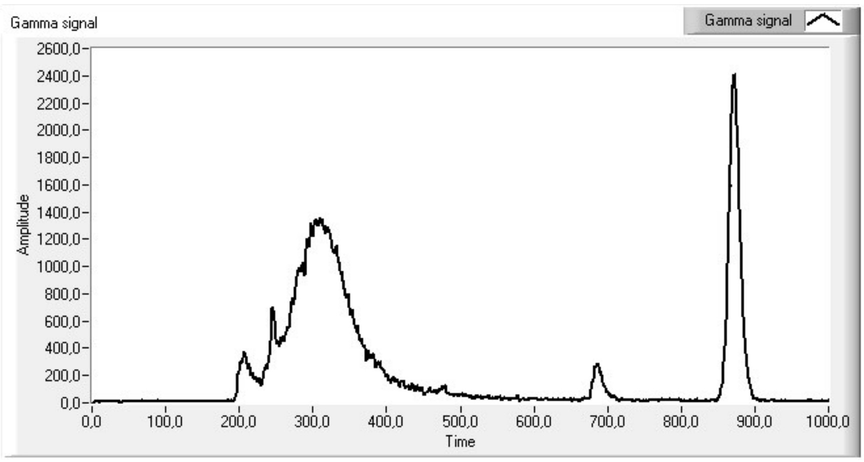

Figure S11. *Semi-preparative HPLC chromatogram for **[^{18}F]11** (R_t = 980 sec).*

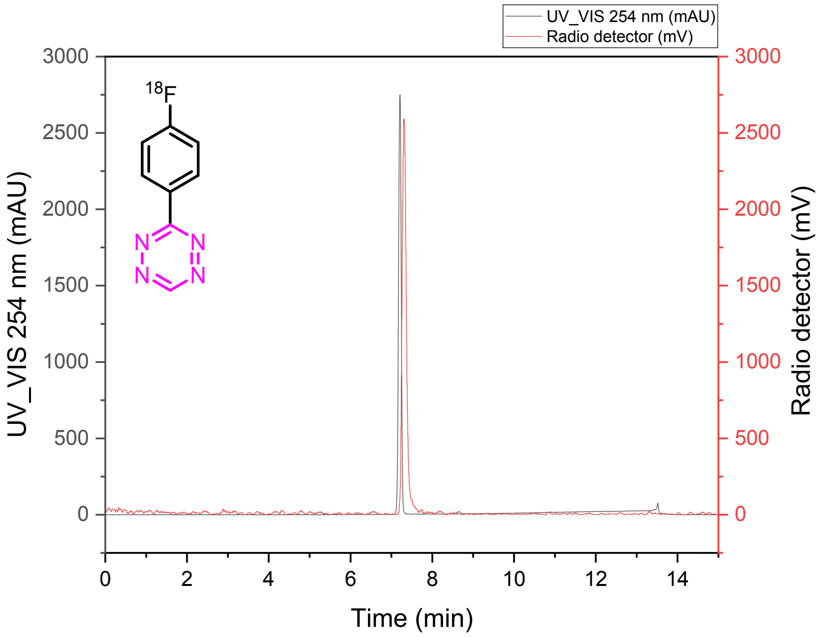

Figure S12. *Analytical-HPLC chromatogram of reference compound **11** (UV/Vis, 254 nm) and radio-HPLC chromatogram of formulated **[^{18}F]11** (Rt = 6.97 minutes).*

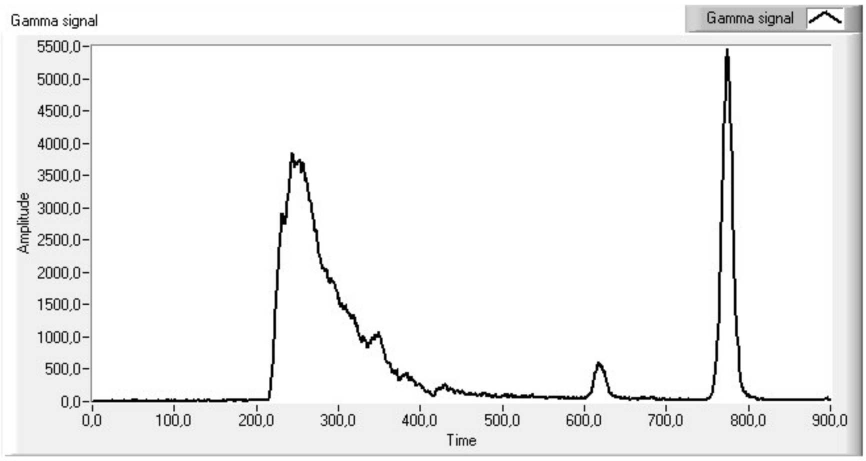

Figure S13. *Semi-preparative HPLC chromatogram for **[¹⁸F]13** (R_t = 790 sec).*

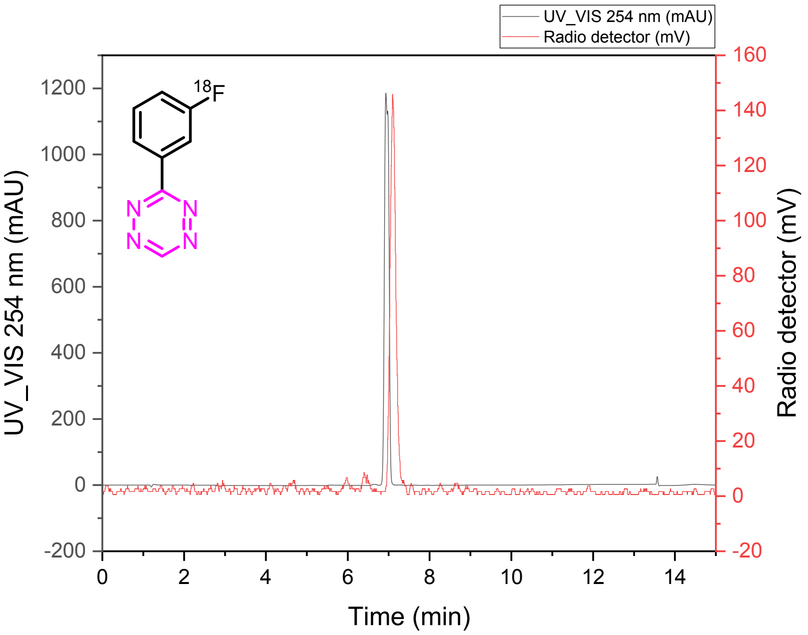

Figure S14. *Analytical-HPLC chromatogram of reference compound **13** (UV/Vis, 254 nm) and radio-HPLC chromatogram of formulated **[¹⁸F]13** (Rt = 6.87 minutes).*

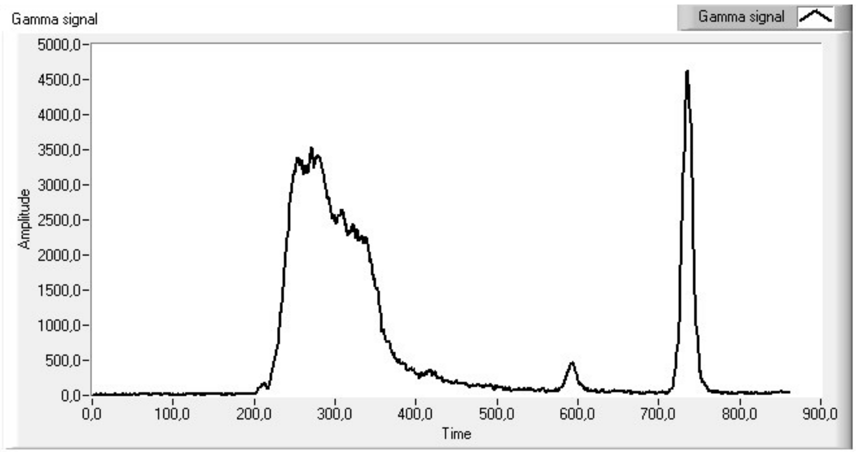

Figure S15. *Semi-preparative HPLC chromatogram for **[^18F]14-m** (R_t = 720 sec).*

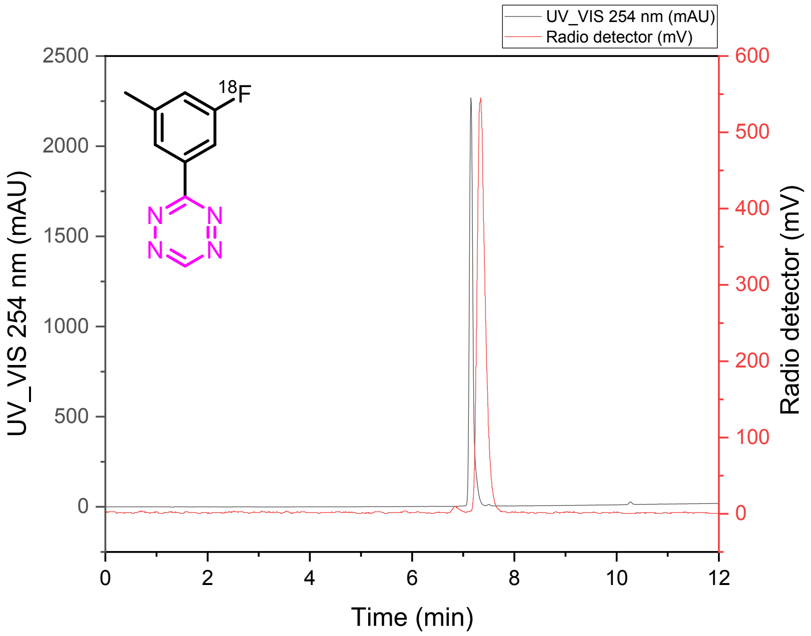

Figure S16. *Analytical-HPLC chromatogram of reference compound **14-m** (UV/Vis, 254 nm) and radio-HPLC chromatogram of formulated **[^18F]14** (Rt = 7.35 minutes).*

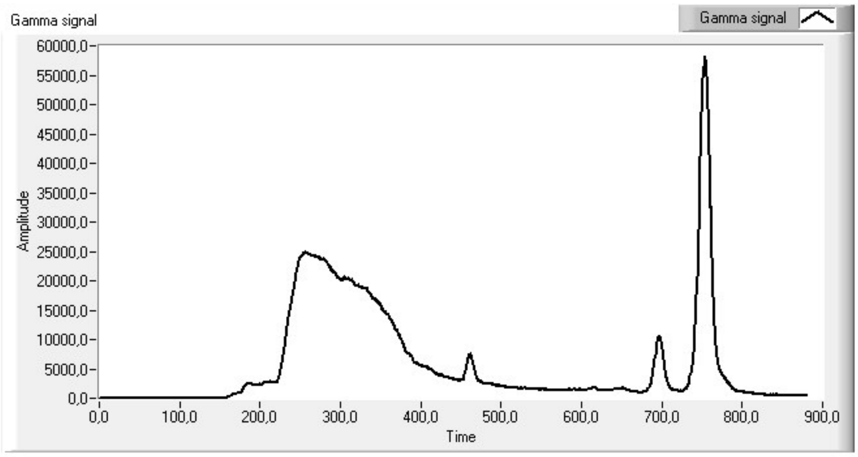

Figure S17. *Semi-preparative HPLC chromatogram for **[**

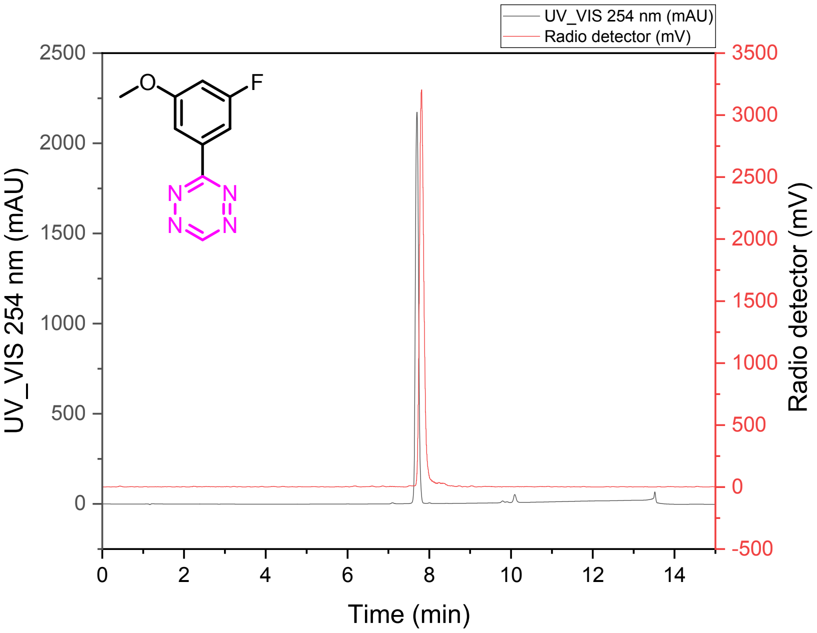

Figure S18. *Analytical-HPLC chromatogram of reference compound **15-m** (UV/Vis, 254 nm) and radio-HPLC chromatogram of formulated **[^{18}F]15-m** (Rt = 7.67 minutes).*

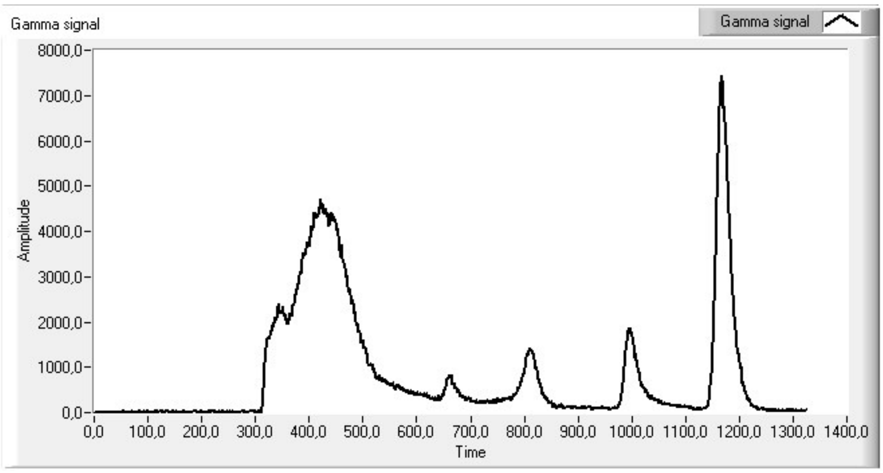

Figure S19. *Semi-preparative HPLC chromatogram for **[^{18}F]16-m** (R_t = 1150 sec).*

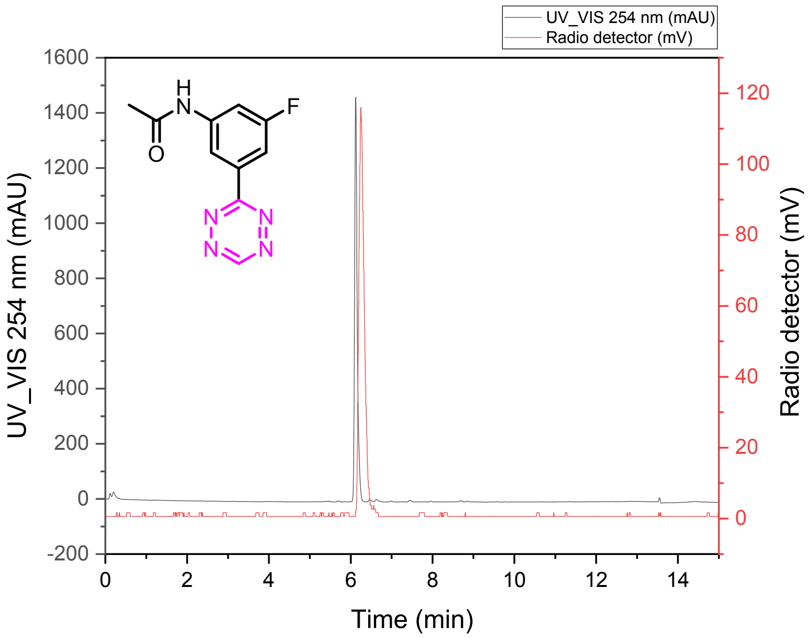

Figure S20. *Analytical-HPLC chromatogram of reference compound **16-m** (UV/Vis, 254 nm) and radio-HPLC chromatogram of formulated **[^{18}F]16-m** (Rt = 6.23 minutes).*

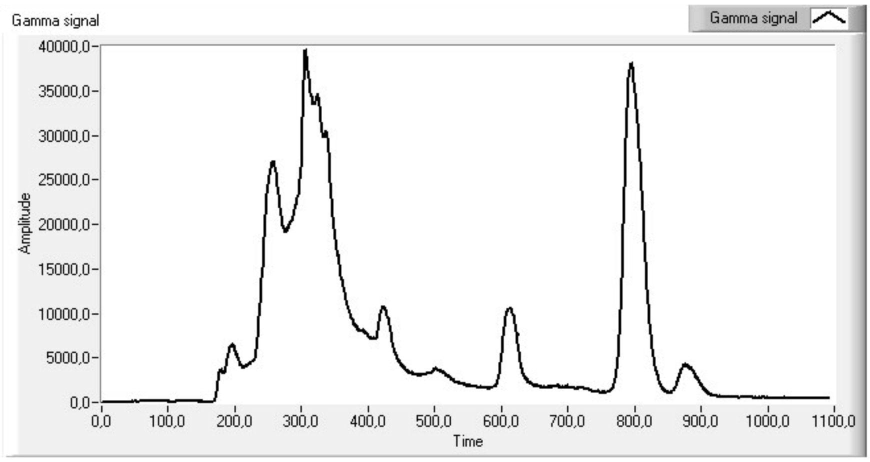

Figure S21. *Semi-preparative HPLC chromatogram for **[^{18}F]17-m** (R_t = 790 sec).*

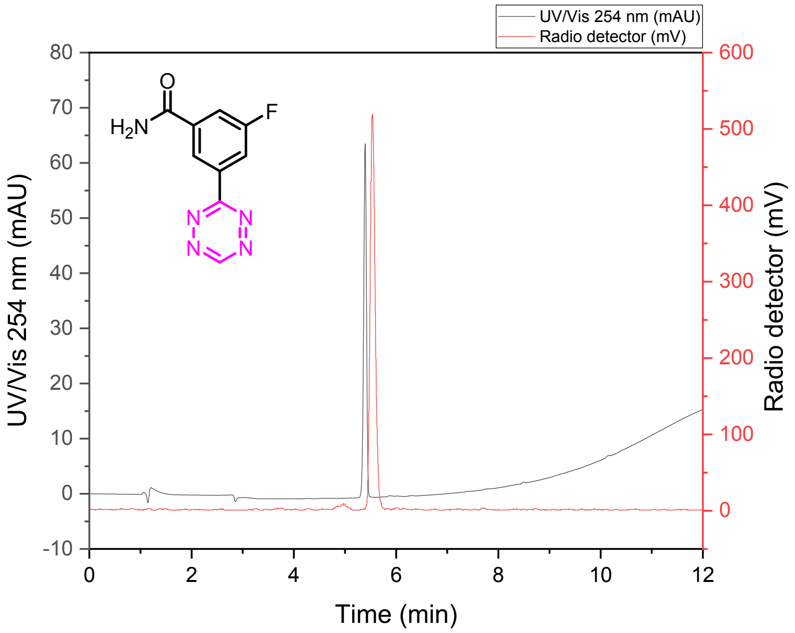

Figure S22. *Analytical-HPLC chromatogram of reference compound **17-m** (UV/Vis, 254 nm) and radio-HPLC chromatogram of formulated **[^{18}F]17-m** (Rt = 5.51 minutes).*

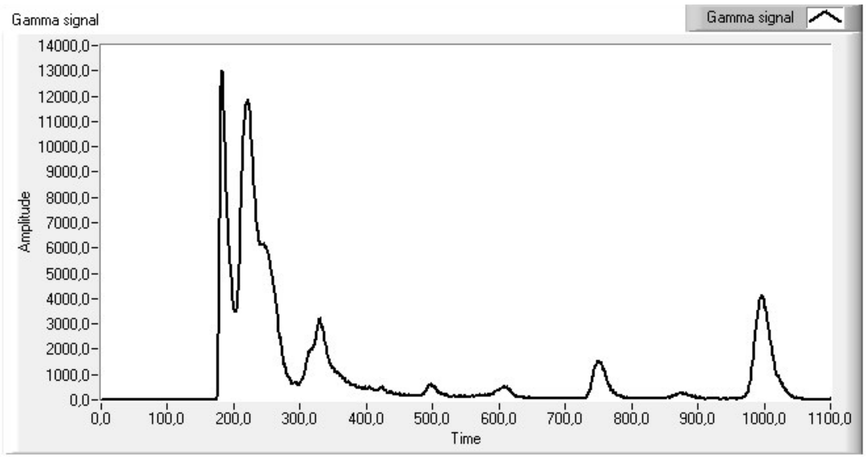

Figure S23. *Semi-preparative HPLC chromatogram for* ***[18F]18-m*** *(R_t = 990 sec).*

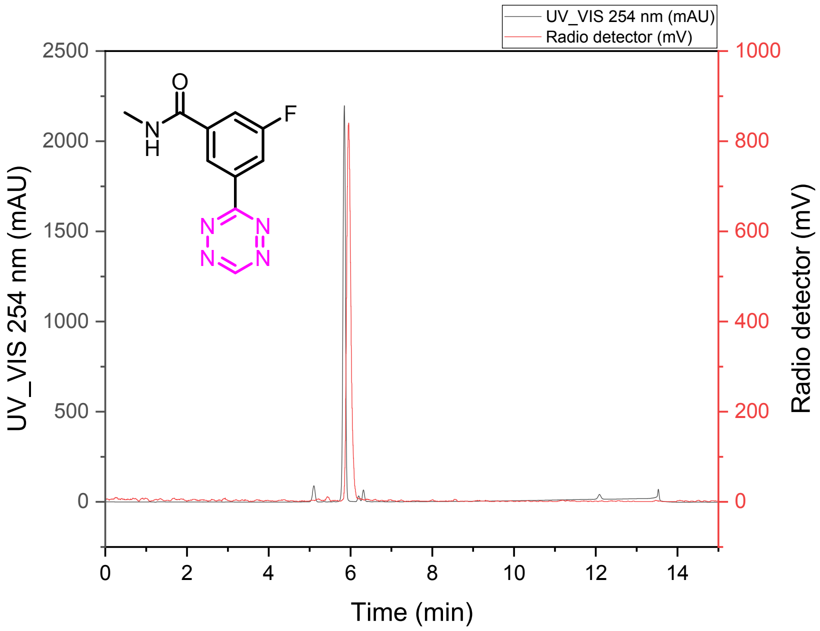

Figure S24. *Analytical-HPLC chromatogram of reference compound* ***18-m*** *(UV/Vis, 254 nm) and radio-HPLC chromatogram of formulated* ***[18F]18-m*** *(Rt = 5.93 minutes).*

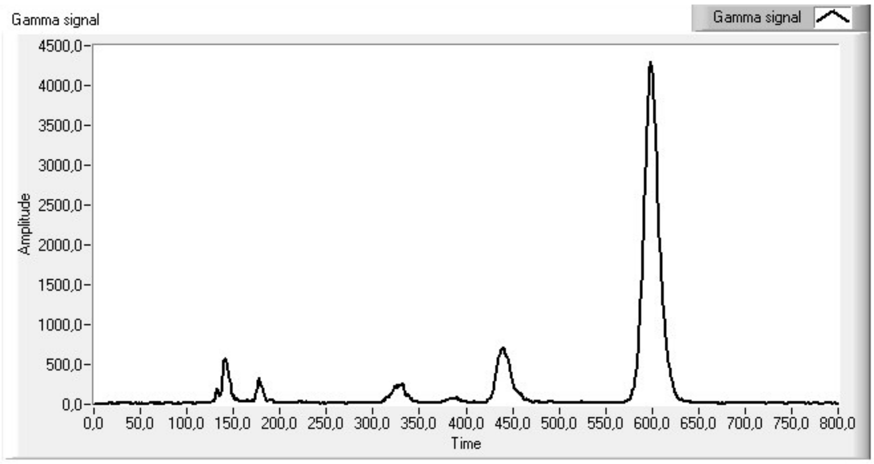

Figure S25. *Semi-preparative HPLC chromatogram for **[^{18}F]21** (R_t = 580 sec).*

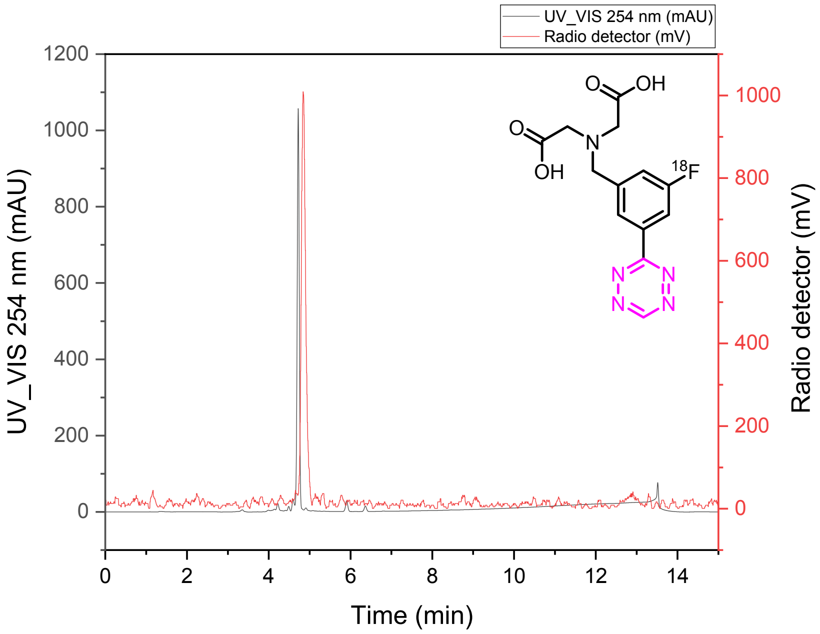

Figure S26. *Analytical-HPLC chromatogram of reference compound **21** (UV/Vis, 254 nm) and radio-HPLC chromatogram of formulated **[^{18}F]21** (Rt = 4.86 minutes).*

Section S8: References

1. J. Yang, M. R. Karver, W. Li, S. Sahu and N. K. Devaraj, *Angew Chem Int Ed Engl*, 2012, **51**, 5222-5225.
2. Y. Qu, F. X. Sauvage, G. Clavier, F. Miomandre and P. Audebert, *Angew Chem Int Ed Engl*, 2018, **57**, 12057-12061.
3. S. A. Albu, S. A. Al-Karmi, A. Vito, J. P. Dzandzi, A. Zlitni, D. Beckford-Vera, M. Blacker, N. Janzen, R. M. Patel, A. Capretta and J. F. Valliant, *Bioconjug Chem*, 2016, **27**, 207-216.
4. M. Sun, C. Zhao, G. A. Gfesser, C. Thiffault, T. R. Miller, K. Marsh, J. Wetter, M. Curtis, R. Faghih, T. A. Esbenshade, A. A. Hancock and M. Cowart, *J Med Chem*, 2005, **48**, 6482-6490.
5. I. K. Khanna, R. M. Weier, Y. Yu, X. D. Xu, F. J. Koszyk, P. W. Collins, C. M. Koboldt, A. W. Veenhuizen, W. E. Perkins, J. J. Casler, J. L. Masferrer, Y. Y. Zhang, S. A. Gregory, K. Seibert and P. C. Isakson, *J Med Chem*, 1997, **40**, 1634-1647.
6. M. Esfahanizadeh, K. Omidi, J. Kauffman, A. Gudarzi, S. Shahraki Zahedani, S. Amidi and F. Kobarfard, *Iran J Pharm Res*, 2014, **13**, 115-126.
7. Z. Zhu, J. Wang, A. I. Lopez, F. Yu, Y. Huang, A. Kumar, S. Li, L. Zhang and C. Cai, *Biomater Sci*, 2015, **3**, 842-851.
8. I. Nymann Petersen, J. Madsen, C. Bernard Matthijs Poulie, A. Kjær and M. Manfred Herth, *Molecules*, 2019, **24**.
9. M. E. Kieffer, K. V. Chuang and S. E. Reisman, *Chem Sci*, 2012, **3**, 3170-3174.
10. E. Kozma, G. Estrada Girona, G. Paci, E. A. Lemke and P. Kele, *Chem Commun (Camb)*, 2017, **53**, 6696-6699.
11. J. W. McIntee, C. Sundararajan, A. C. Donovan, M. S. Kovacs, A. Capretta and J. F. Valliant, *J Org Chem*, 2008, **73**, 8236-8243.
12. M. M. Herth, S. Ametamey, D. Antuganov, A. Bauman, M. Berndt, A. F. Brooks, G. Bormans, Y. S. Choe, N. Gillings, U. O. Häfeli, M. L. James, K. Kopka, V. Kramer, R. Krasikova, J. Madsen, L. Mu, B. Neumaier, M. Piel, F. Rösch, T. Ross, R. Schibli, P. J. H. Scott, V. Shalgunov, N. Vasdev, W. Wadsak and B. M. Zeglis, *Nucl Med Biol*, 2021, **93**, 19-21.
13. C. N. Neumann, J. M. Hooker and T. Ritter, *Nature*, 2016, **534**, 369-373.
14. M. Tredwell and V. Gouverneur, *Angewandte Chemie International Edition*, 2012, **51**, 11426-11437.
15. T. L. Ross, J. Ermert, C. Hocke and H. H. Coenen, *J Am Chem Soc*, 2007, **129**, 8018-8025.
16. M. Pauton, C. Aubert, G. Bluet, F. Gruss-Leleu, S. b. Roy and C. c. Perrio, *Organic Process Research & Development*, 2019, **23**, 900-911.
17. I. N. Petersen, J. Villadsen, H. D. Hansen, J. Madsen, A. A. Jensen, N. Gillings, S. Lehel, M. M. Herth, G. M. Knudsen and J. L. Kristensen, *Org Biomol Chem*, 2017, **15**, 4351-4358.
18. M. H. Beyzavi, D. Mandal, M. G. Strebl, C. N. Neumann, E. M. D'Amato, J. Chen, J. M. Hooker and T. Ritter, *ACS Cent Sci*, 2017, **3**, 944-948.
19. K. J. Makaravage, A. F. Brooks, A. V. Mossine, M. S. Sanford and P. J. H. Scott, *Org Lett*, 2016, **18**, 5440-5443.
20. P. Borman and D. Elder, *Journal*, 2018, 39-40.
21. R. Rossin, P. Renart Verkerk, S. M. van den Bosch, R. C. Vulders, I. Verel, J. Lub and M. S. Robillard, *Angewandte Chemie*, 2010, **122**, 3447-3450.

22. R. Rossin, S. M. van den Bosch, W. Ten Hoeve, M. Carvelli, R. M. Versteegen, J. Lub and M. S. Robillard, *Bioconjug Chem*, 2013, **24**, 1210-1217.

23. R. Rossin, S. M. van Duijnhoven, T. Läppchen, S. M. van den Bosch and M. S. Robillard, *Mol Pharm*, 2014, **11**, 3090-3096.

24. D. Svatunek, C. Denk, V. Rosecker, B. Sohr, C. Hametner, G. Allmaier, J. Fröhlich and H. Mikula, *Monatsh Chem*, 2016, **147**, 579-585.

25. J. Stéen, J. T. Jørgensen, D. Christoph, U. M. Battisti, K. Nørregaard, P. Edem, K. Bratteby, V. Shalgunov, W. Martin and D. Svatunek, 2020.

26. R. M. Versteegen, R. Rossin, W. ten Hoeve, H. M. Janssen and M. S. Robillard, *Angew Chem Int Ed Engl*, 2013, **52**, 14112-14116.
