# Supplementary material for: Direct Cu-mediated aromatic 18F-labeling of highly reactive tetrazines for pretargeted bioorthogonal PET imaging
Source: Chem Sci. 2021 Jul 28;12(35):11668–75. doi: 10.1039/d1sc02789a (PMC8442695; doi:10.1039/d1sc02789a)
Supplement: SC-012-D1SC02789A-s002 [file SC-012-D1SC02789A-s002.pdf]

This journal is © The Royal Society of Chemistry 2021

Section S9: NMR spectra and Analytical HPLC

^{1}H NMR spectrum of 3-(4-fluorophenyl)-6-methyl-1,2,4,5-tetrazine (**6**) (600 MHz, $CDCl_3$)

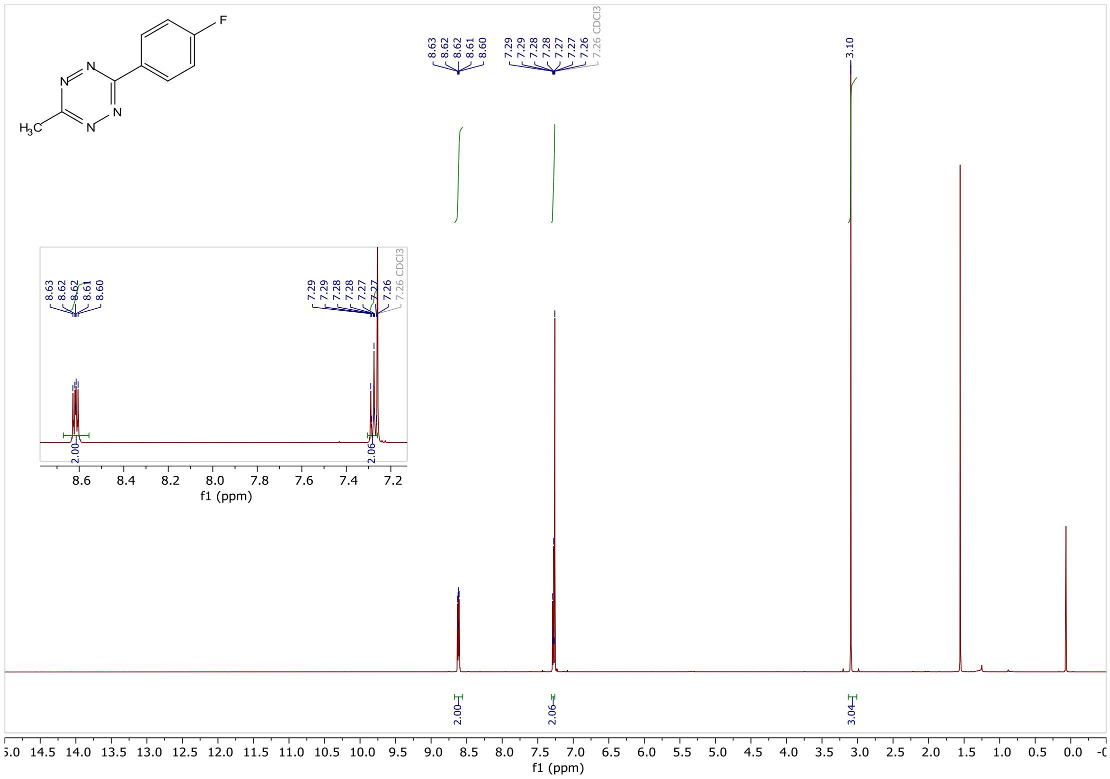

^{13}C NMR spectrum of 3-(4-fluorophenyl)-6-methyl-1,2,4,5-tetrazine (**6**) (151 MHz, CDCl3)

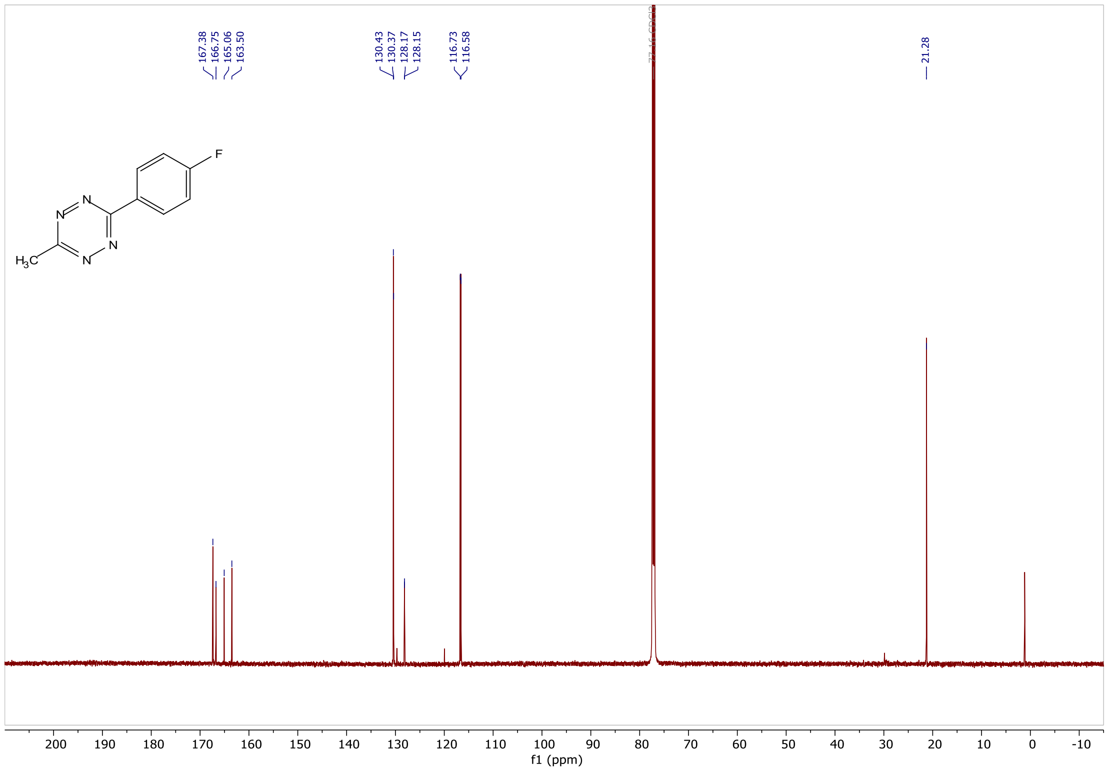

Analytical HPLC of 3-(4-fluorophenyl)-6-methyl-1,2,4,5-tetrazine (**6**)

Chromatogram

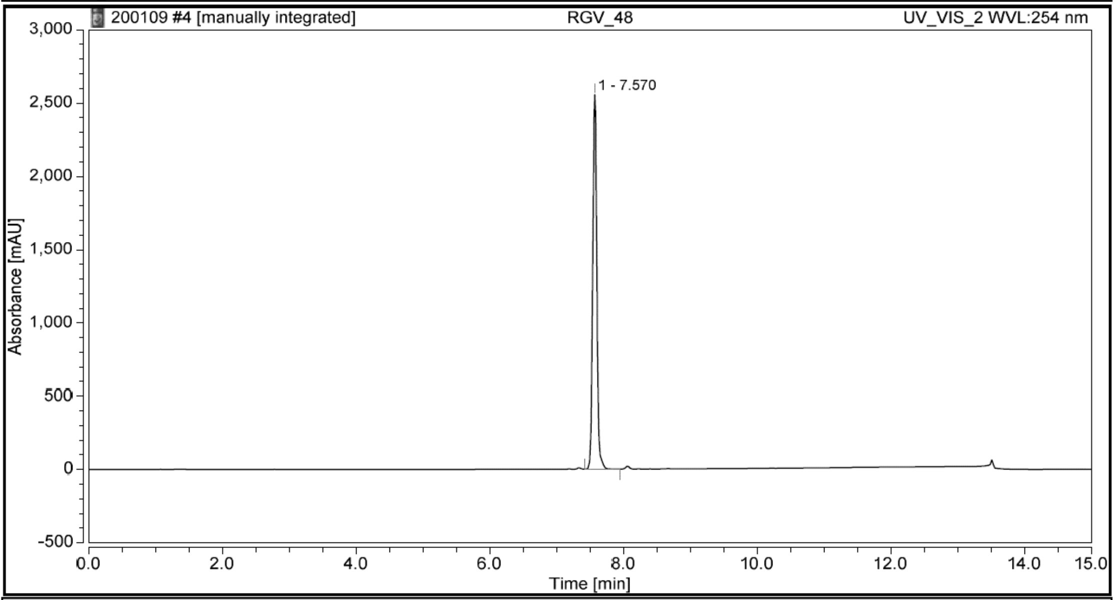

Integration Results

No.	Peak Name	Retention Time min	Area mAU*min	Height mAU	Relative Area %	Relative Height %	Amount
n.a.	Tosylate	n.a.	n.a.	n.a.	n.a.	n.a.	n.a.
n.a.	cold fallyprode	n.a.	n.a.	n.a.	n.a.	n.a.	n.a.
n.a.	18F-fallypride	n.a.	n.a.	n.a.	n.a.	n.a.	n.a.
1		7.570	180.963	2556.418	100.00	100.00	n.a.
**Total:**			**180.963**	**2556.418**	**100.00**	**100.00**	

^{1}H NMR spectrum of 3-(4-iodophenyl)-6-methyl-1,2,4,5-tetrazine (**1**) (400 MHz, $CDCl_3$)

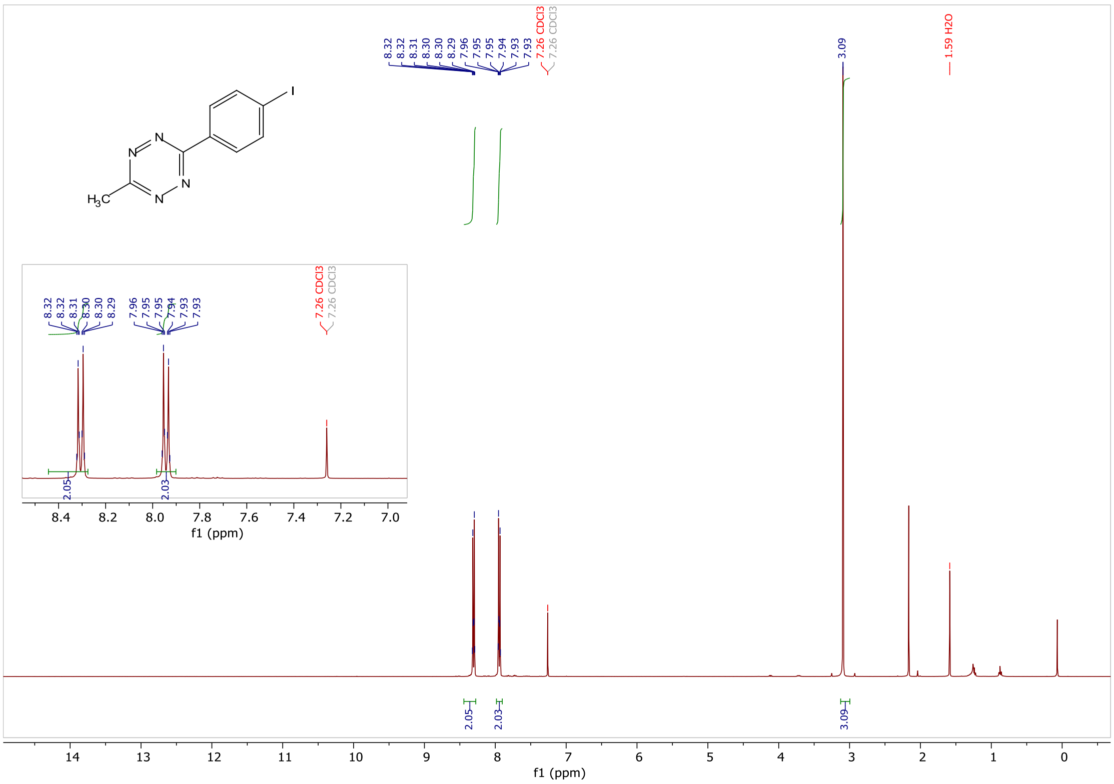

^{13}C NMR spectrum of 3-(4-iodophenyl)-6-methyl-1,2,4,5-tetrazine (**1**) (101 MHz, CDCl3)

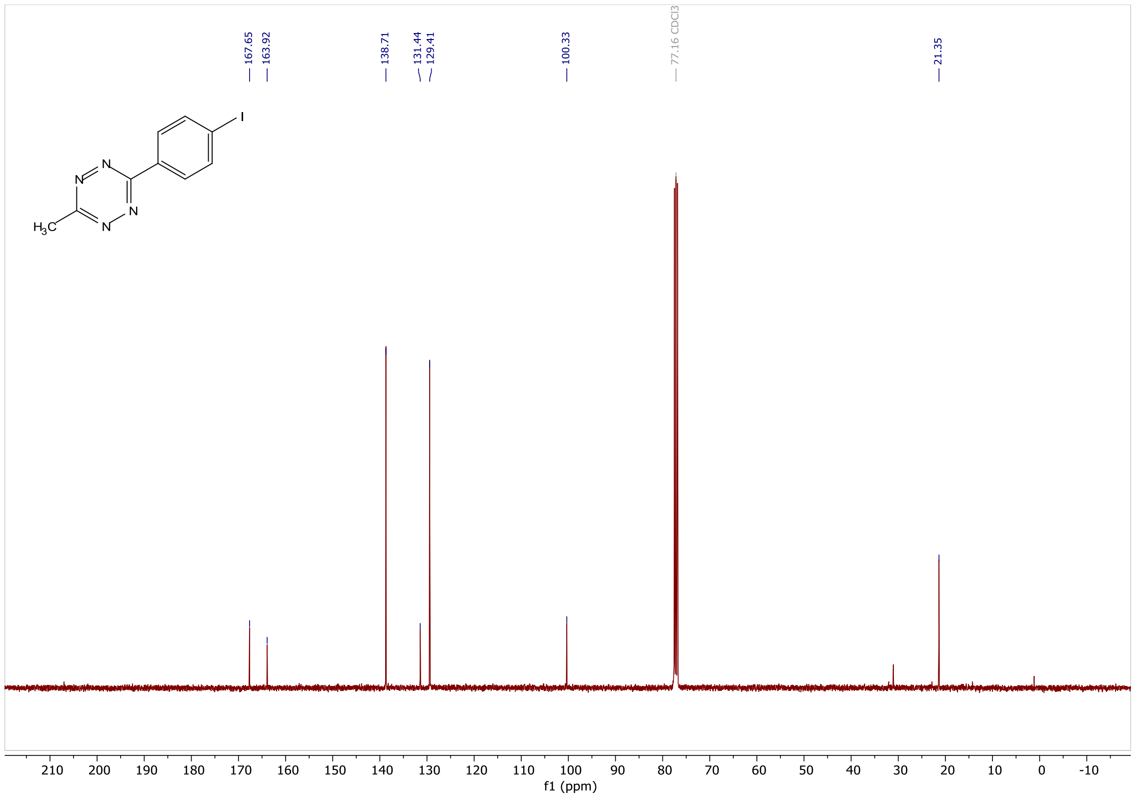

^{1}H NMR spectrum of 4-(1,2,4,5-tetrazin-3-yl)phenol (**4**) (400 MHz, MeOD)

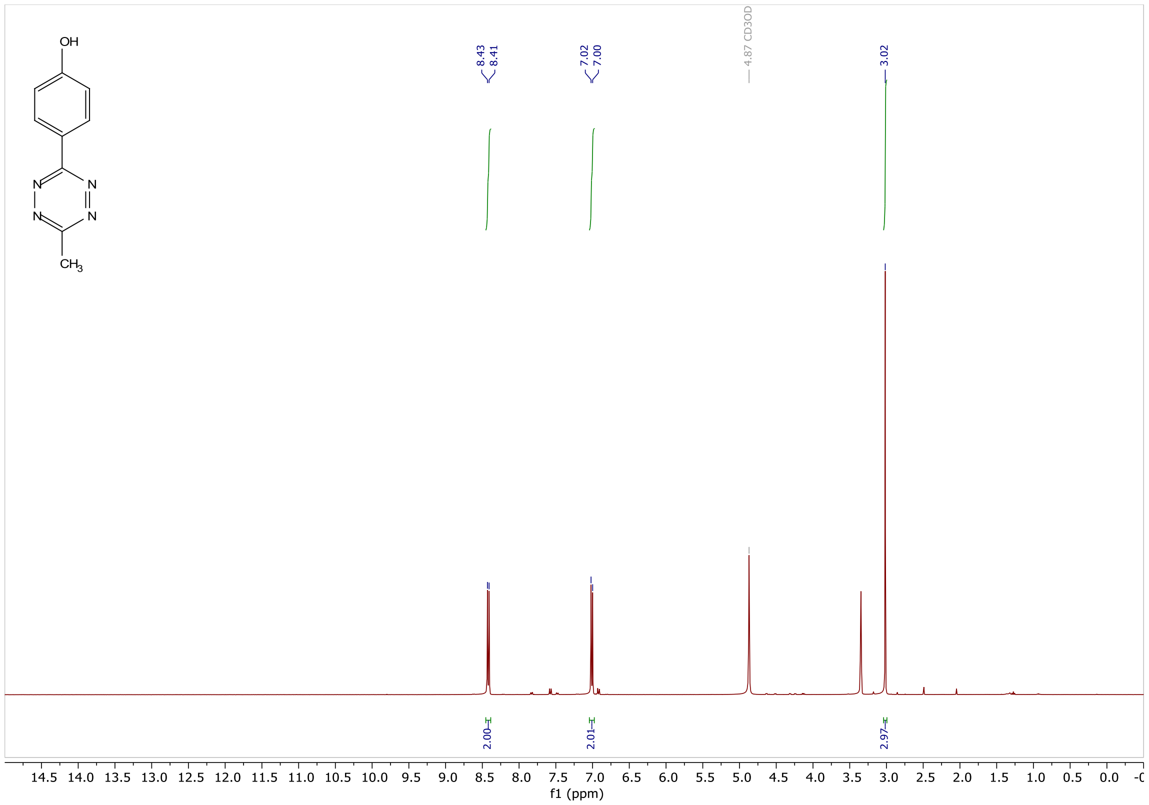

^{13}C NMR spectrum of 4-(1,2,4,5-tetrazin-3-yl)phenol (**4**) (101 MHz, MeOD)

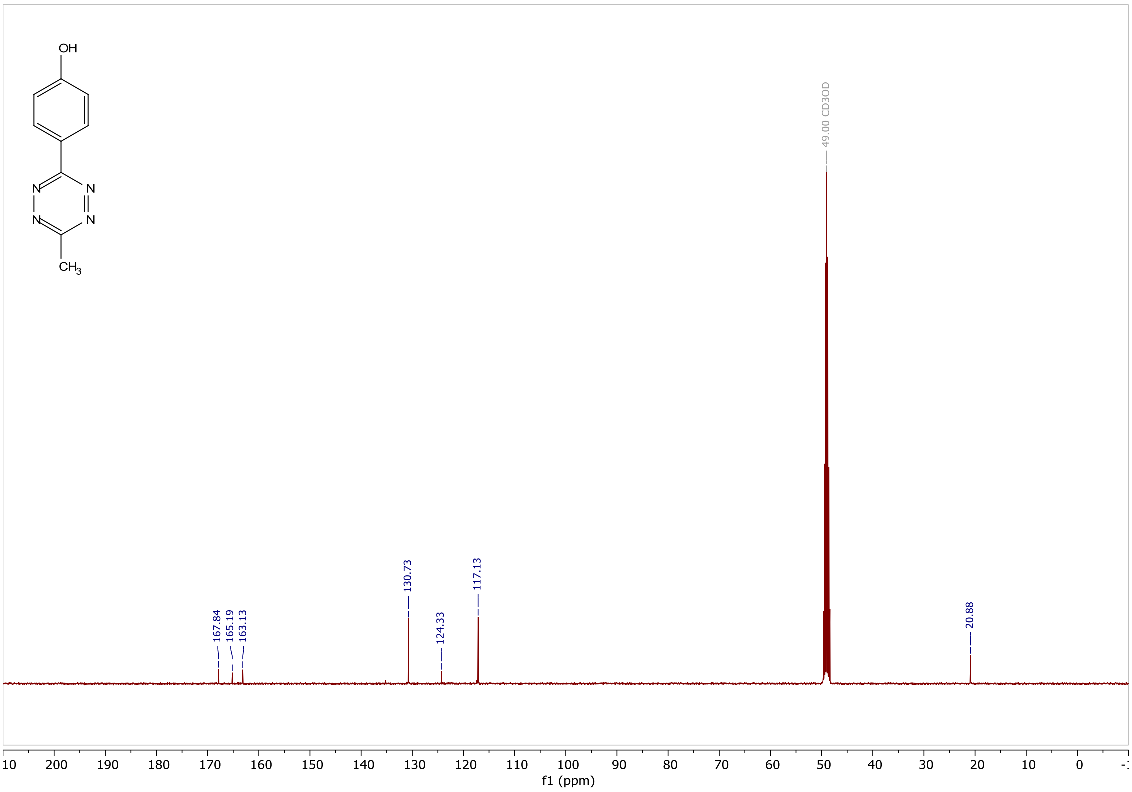

1H NMR spectrum of 3-(3-fluorophenyl)-6-methyl-1,2,4,5-tetrazine (**7**) (400 MHz, $CDCl_3$)

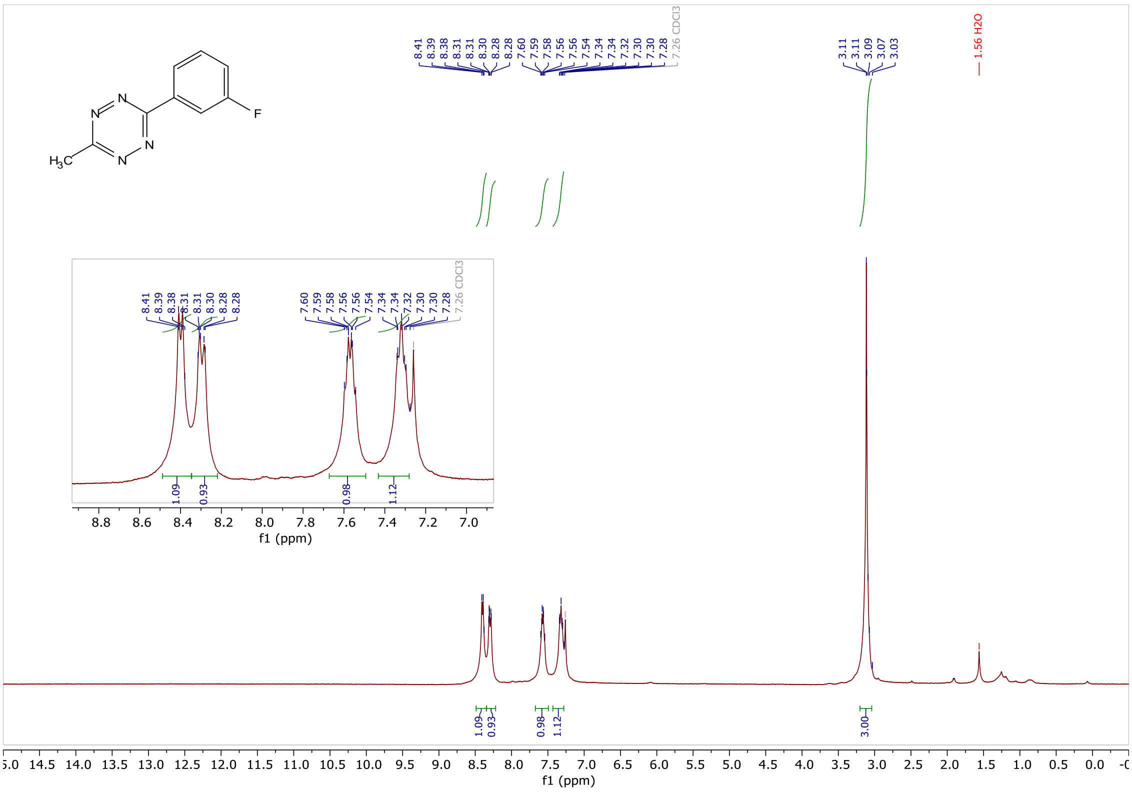

^{13}C NMR spectrum of 3-(3-fluorophenyl)-6-methyl-1,2,4,5-tetrazine (**7**) (101 MHz, CDCl3)

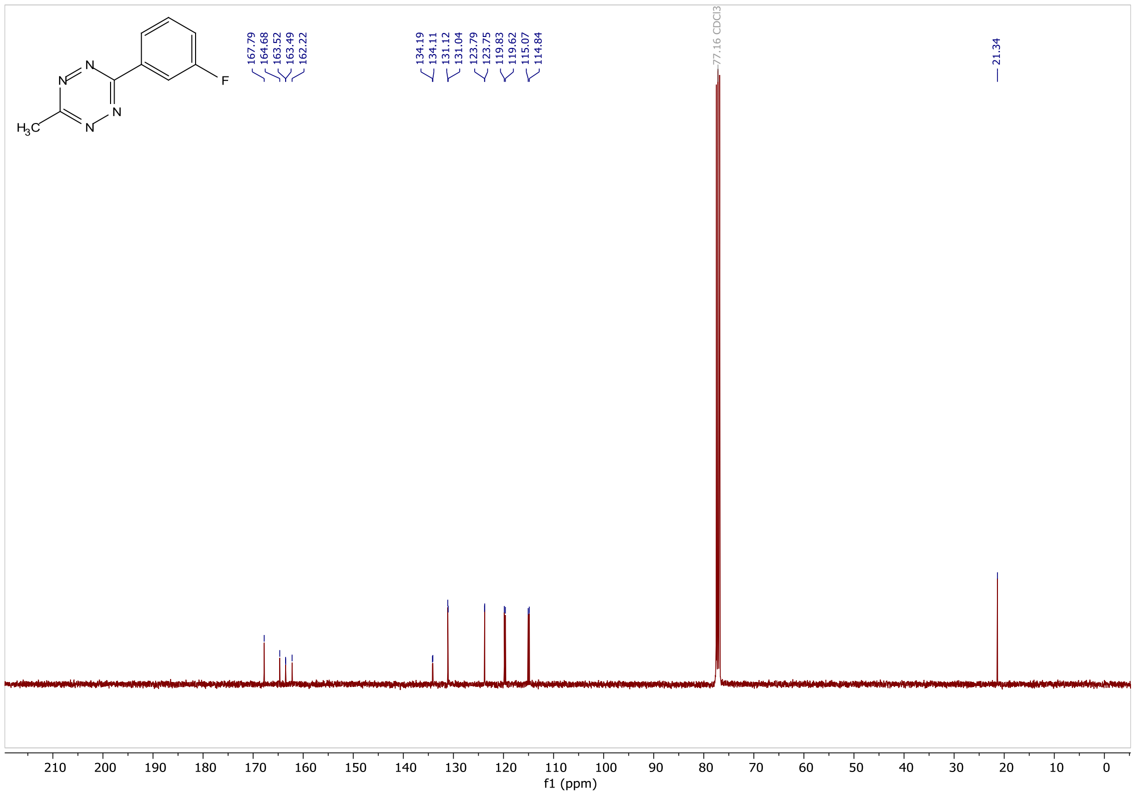

Analytical HPLC of 3-(3-fluorophenyl)-6-methyl-1,2,4,5-tetrazine (**7**)

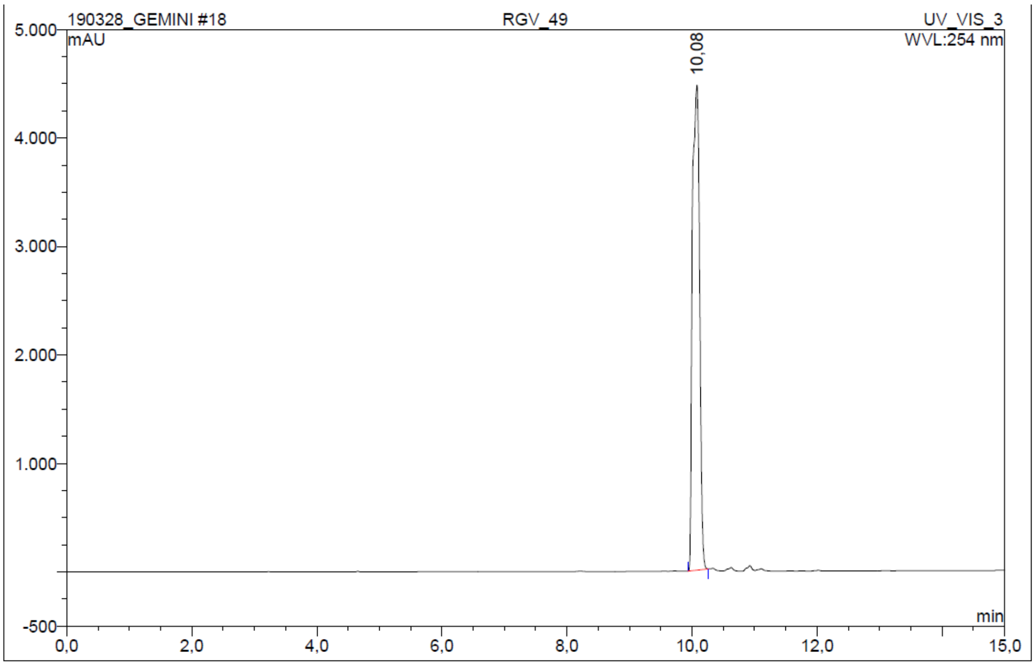

No.	Ret.Time min	Peak Name	Height mAU	Area mAU*min	Rel.Area %	Amount	Resolution(EP)
1	10,08	n.a.	4471,813	589,147	100,00	n.a.	n.a.
Total:			4471,813	589,147	100,00	0,000	

^{1}H NMR spectrum of 3-(3-iodophenyl)-6-methyl-1,2,4,5-tetrazine (**22a**) (400 MHz, $CDCl_3$)

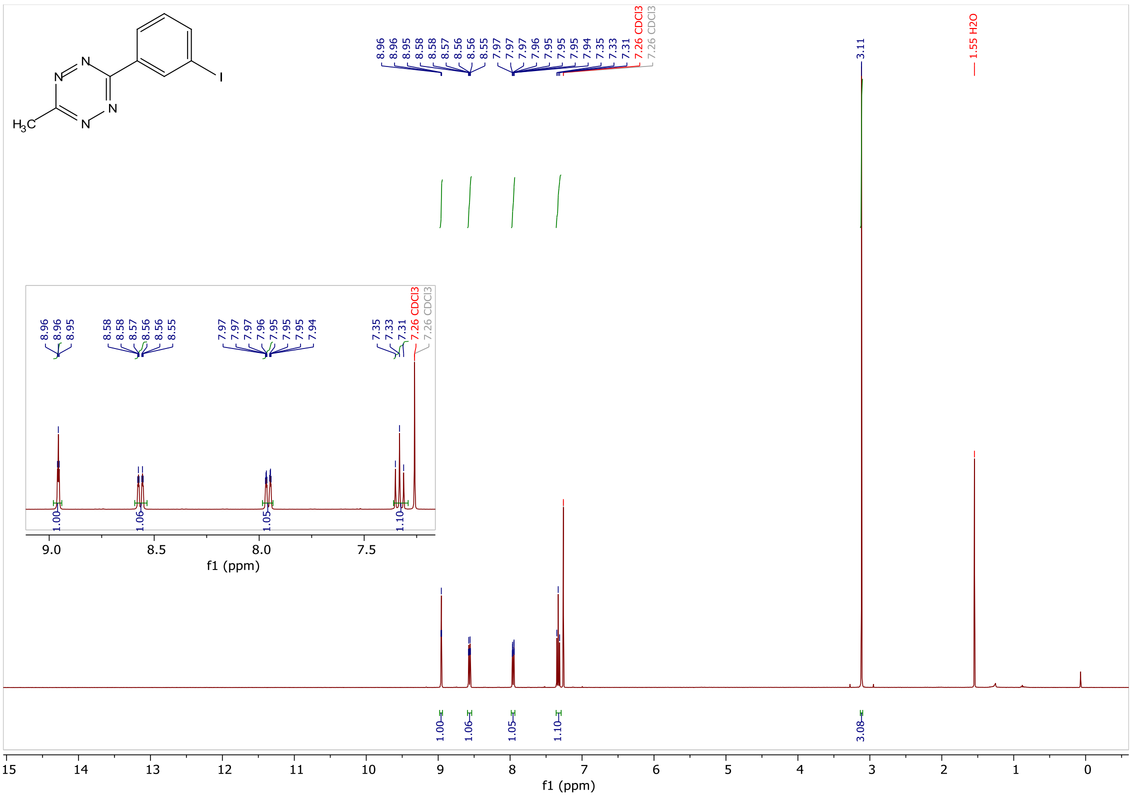

^{13}C NMR spectrum of 3-(3-iodophenyl)-6-methyl-1,2,4,5-tetrazine (**22a**) (101 MHz, CDCl3)

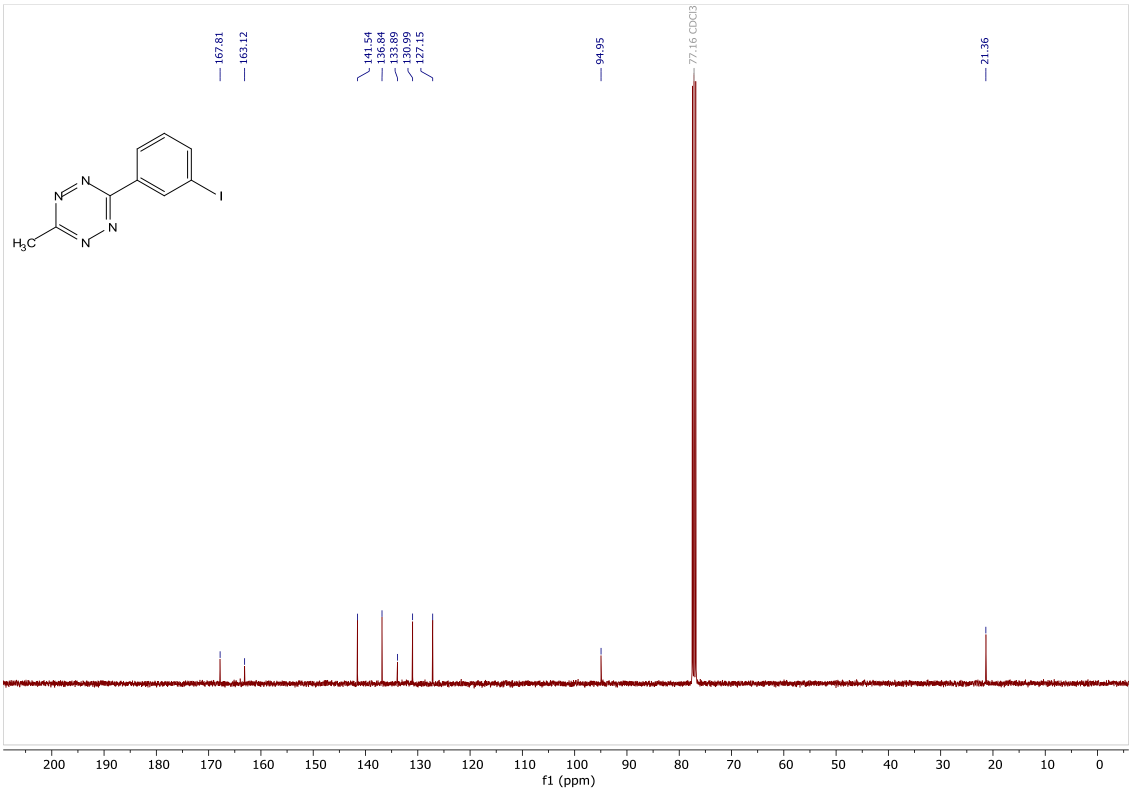

^{1}H NMR spectrum of 3-(4-fluorophenyl)-6-phenyl-1,2,4,5-tetrazine (**8**) (600 MHz, $CDCl_3$)

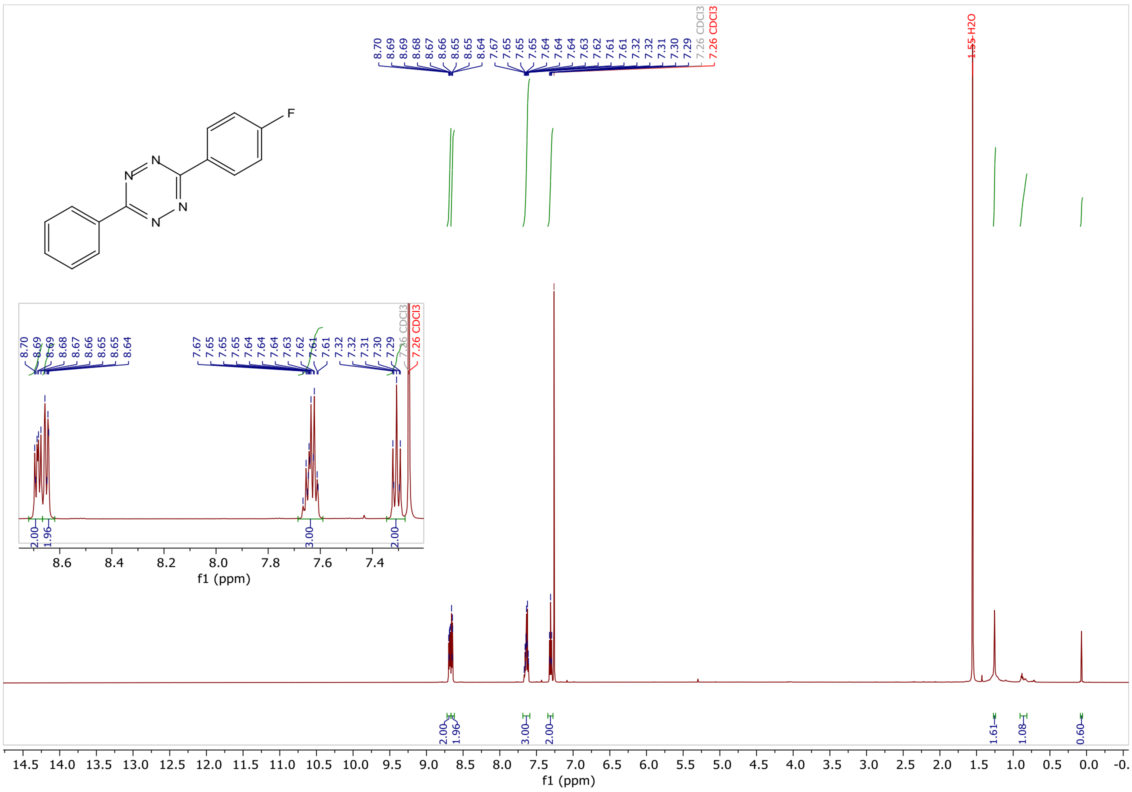

^{13}C NMR spectrum of 3-(4-fluorophenyl)-6-phenyl-1,2,4,5-tetrazine (**8**) (151 MHz, CDCl3)

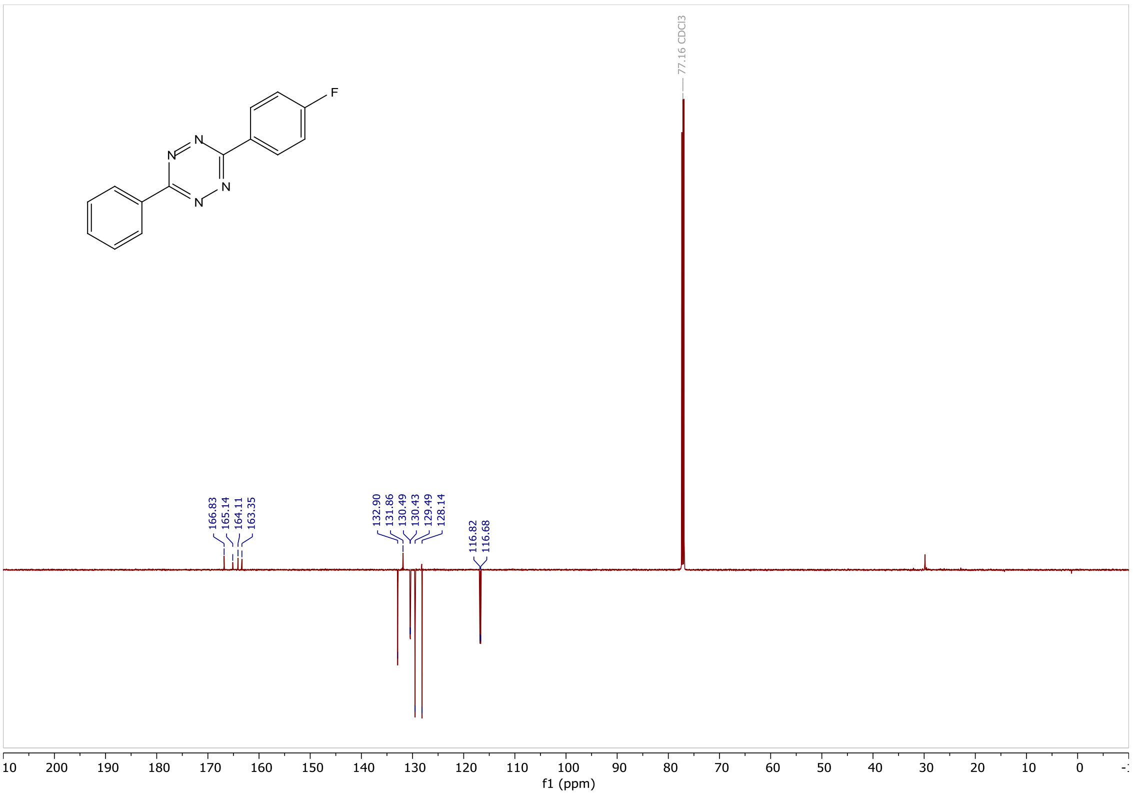

Analytical HPLC of 3-(4-fluorophenyl)-6-phenyl-1,2,4,5-tetrazine (**8**)

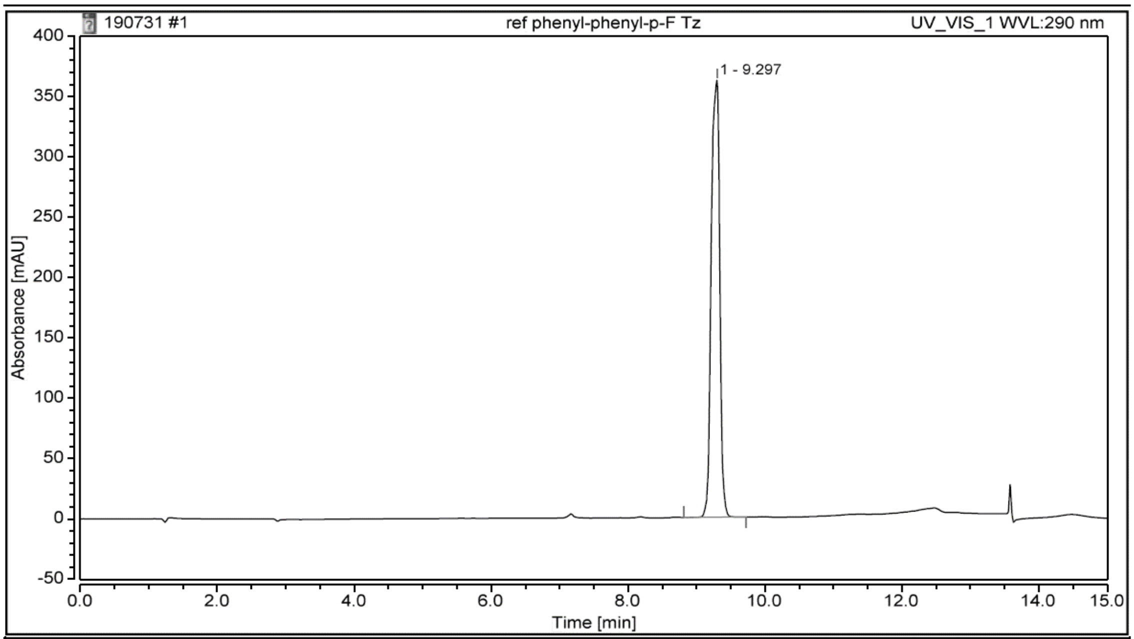

Integration Results

No.	Peak Name	Retention Time min	Area mAU*min	Height mAU	Relative Area %	Relative Height %
1		9.297	51.978	362.384	100.00	100.00
**Total:**			**51.978**	**362.384**	**100.00**	**100.00**

^{1}H NMR spectrum of 3-(4-iodophenyl)-6-phenyl-1,2,4,5-tetrazine (**23a**) (600 MHz, $CDCl_3$)

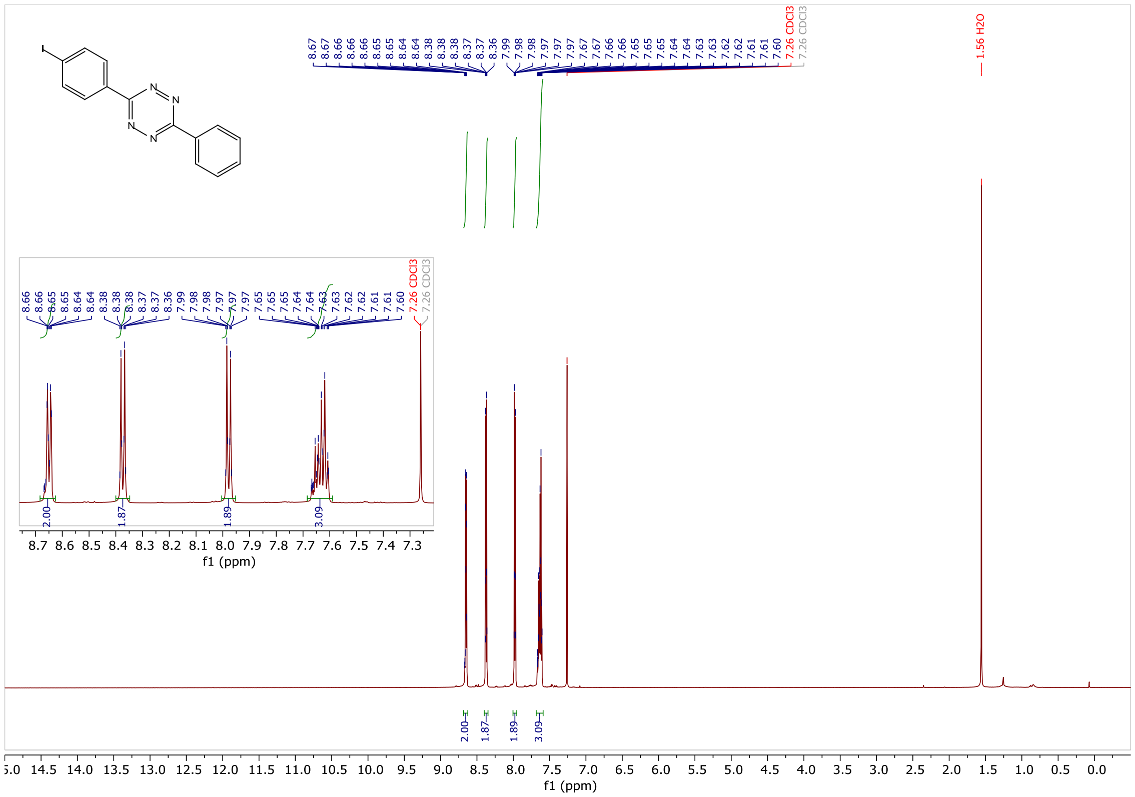

¹³C NMR spectrum of 3-(4-iodophenyl)-6-phenyl-1,2,4,5-tetrazine (**23a**) (151 MHz, CDCl3)

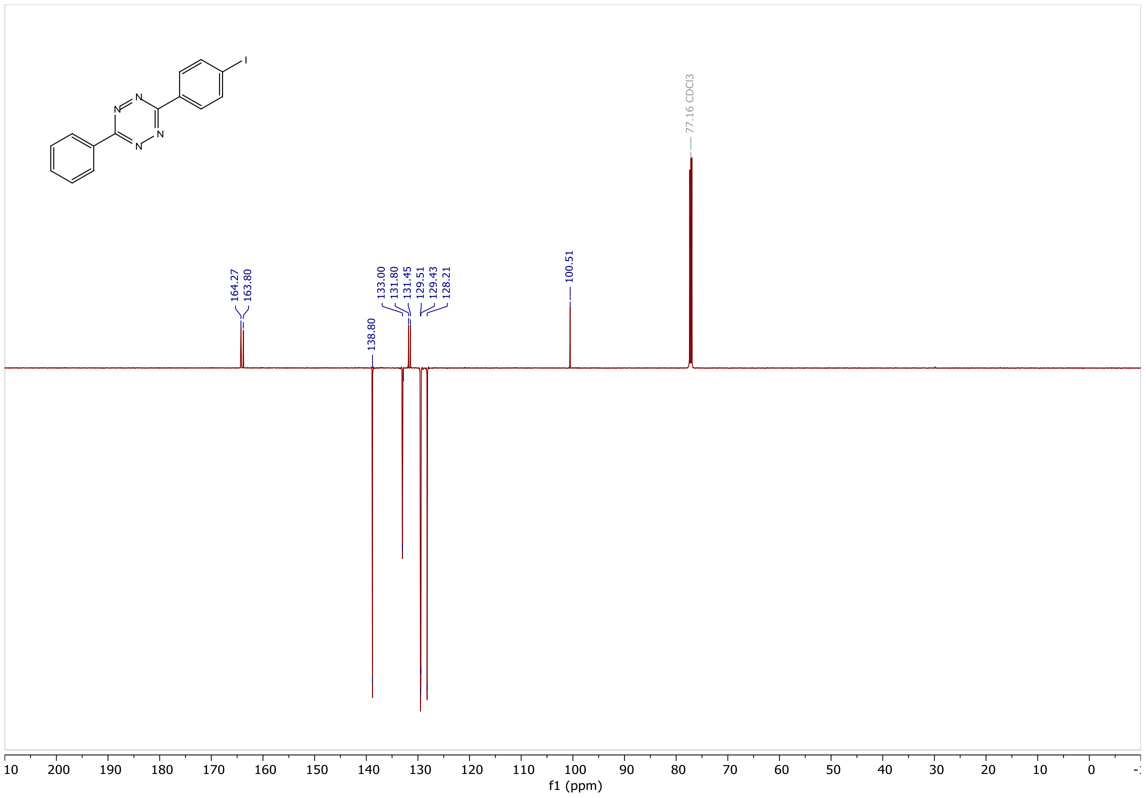

^{1}H NMR spectrum of 3-(3-fluorophenyl)-6-phenyl-1,2,4,5-tetrazine (**9**) (400 MHz, $CDCl_3$)

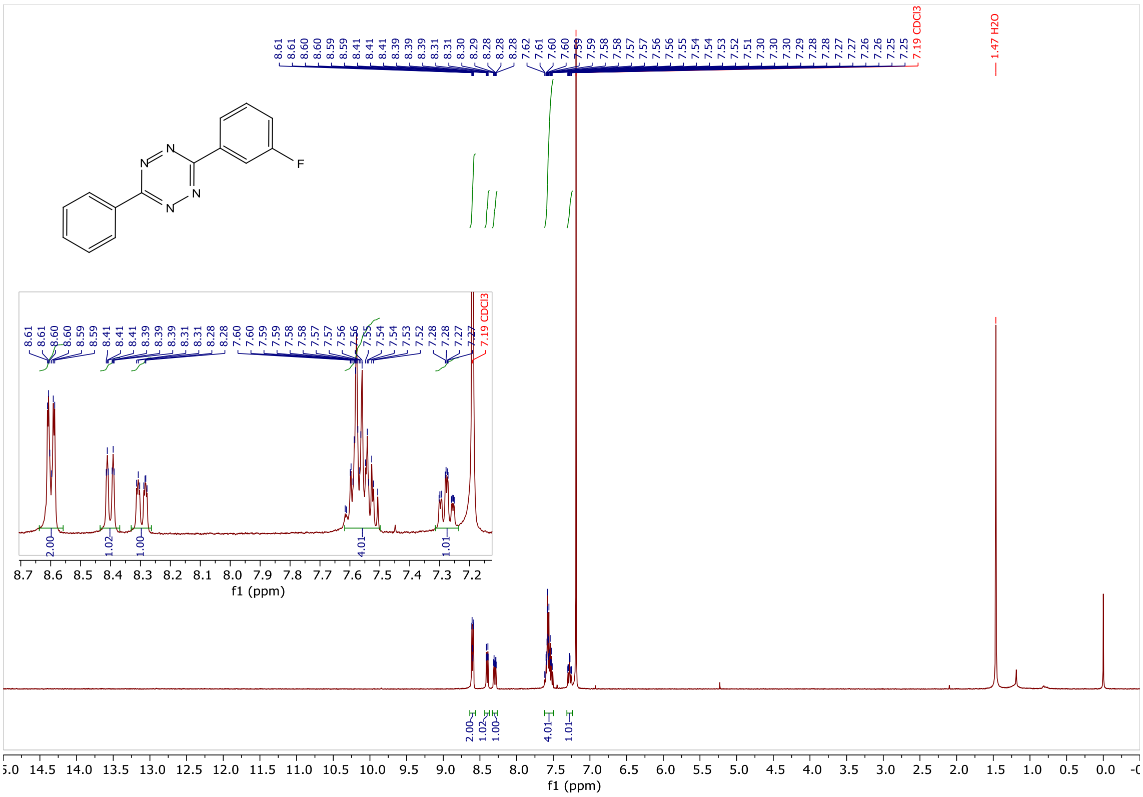

^{13}C NMR spectrum of 3-(3-fluorophenyl)-6-phenyl-1,2,4,5-tetrazine (**9**) (151 MHz, CDCl3)

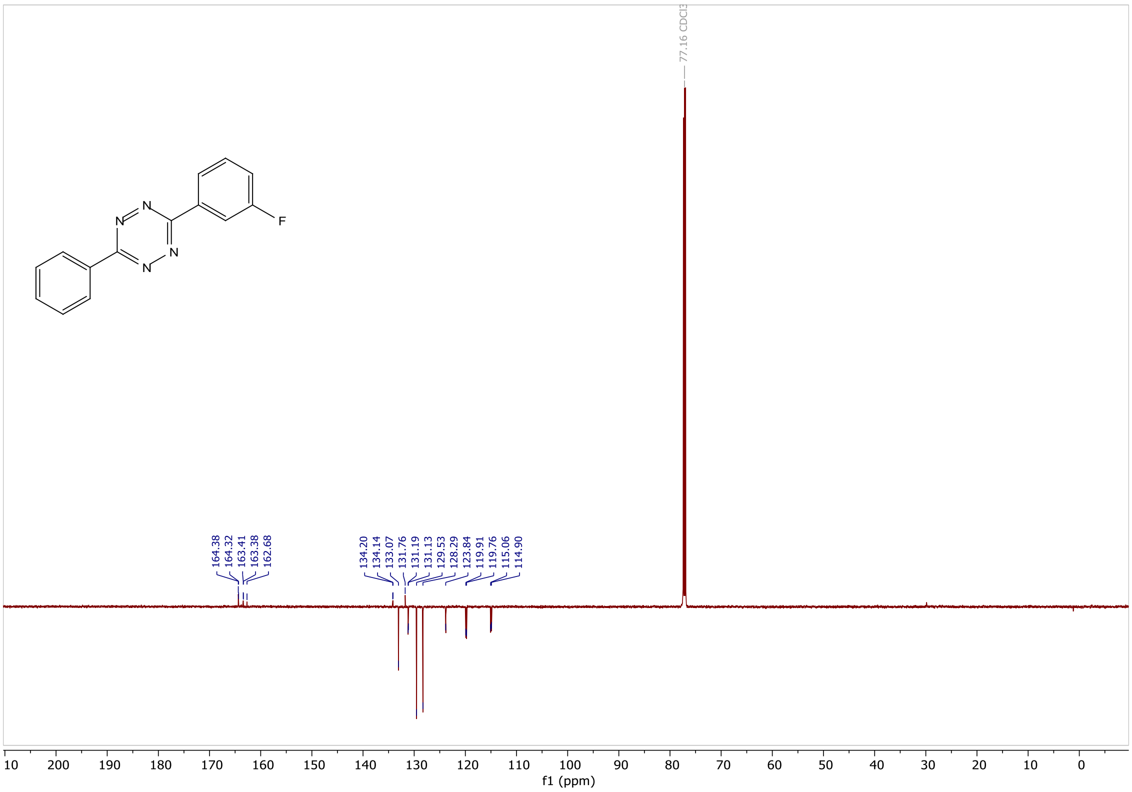

Analytical HPLC of 3-(3-fluorophenyl)-6-phenyl-1,2,4,5-tetrazine (**9**)

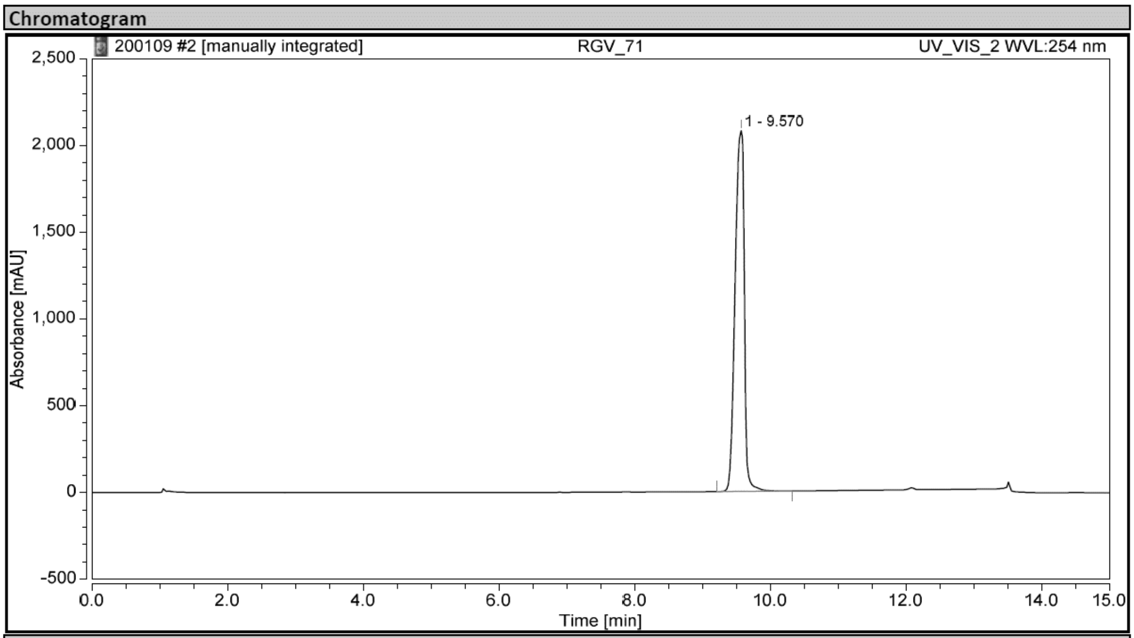

Integration Results

No.	Peak Name	Retention Time min	Area mAU*min	Height mAU	Relative Area %	Relative Height %	Amount
n.a.	Tosylate	n.a.	n.a.	n.a.	n.a.	n.a.	n.a.
n.a.	cold fallyprode	n.a.	n.a.	n.a.	n.a.	n.a.	n.a.
n.a.	18F-fallypride	n.a.	n.a.	n.a.	n.a.	n.a.	n.a.
1		9.570	325.754	2079.320	100.00	100.00	n.a.
**Total:**			**325.754**	**2079.320**	**100.00**	**100.00**	

^{1}H NMR spectrum of 3-(3-iodophenyl)-6-phenyl-1,2,4,5-tetrazine (**24a**) (400 MHz, $CDCl_3$)

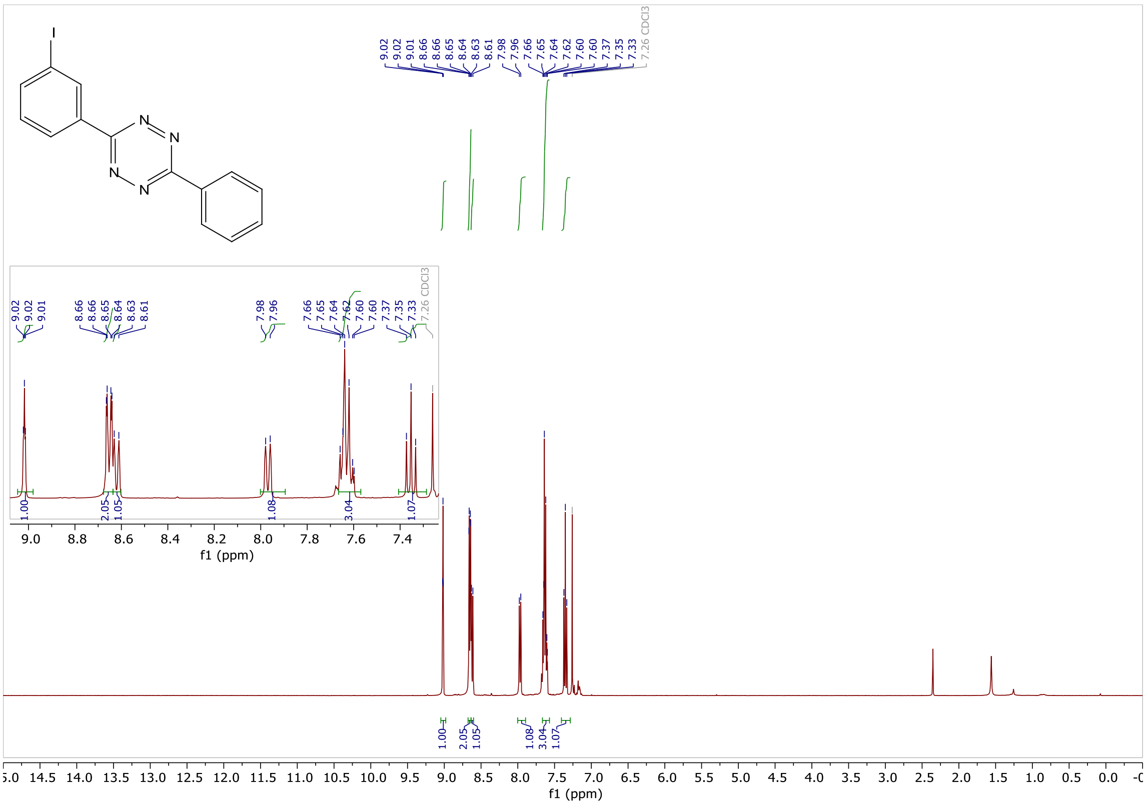

^{13}C NMR spectrum of 3-(3-iodophenyl)-6-phenyl-1,2,4,5-tetrazine (**24a**) (151 MHz, CDCl3)

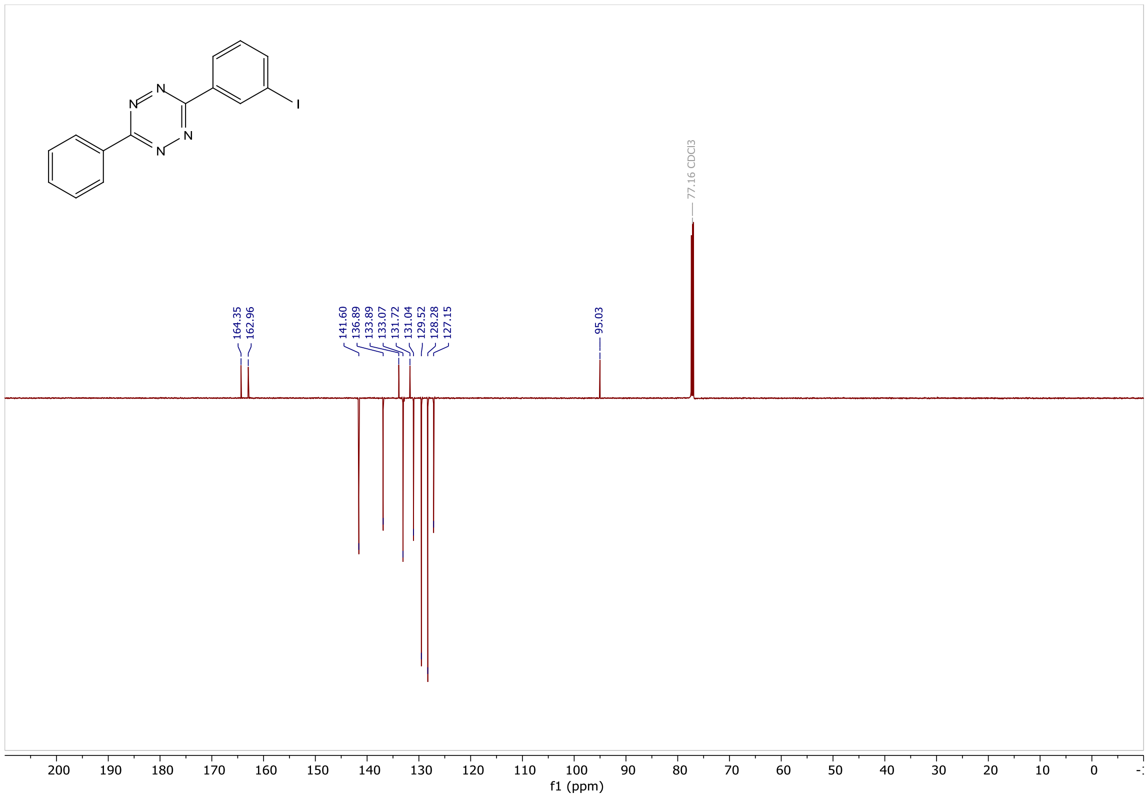

^{1}H NMR spectrum of 3-(4-fluorophenyl)-6-(pyridin-2-yl)-1,2,4,5-tetrazine (**10**) (600 MHz, $CDCl_3$)

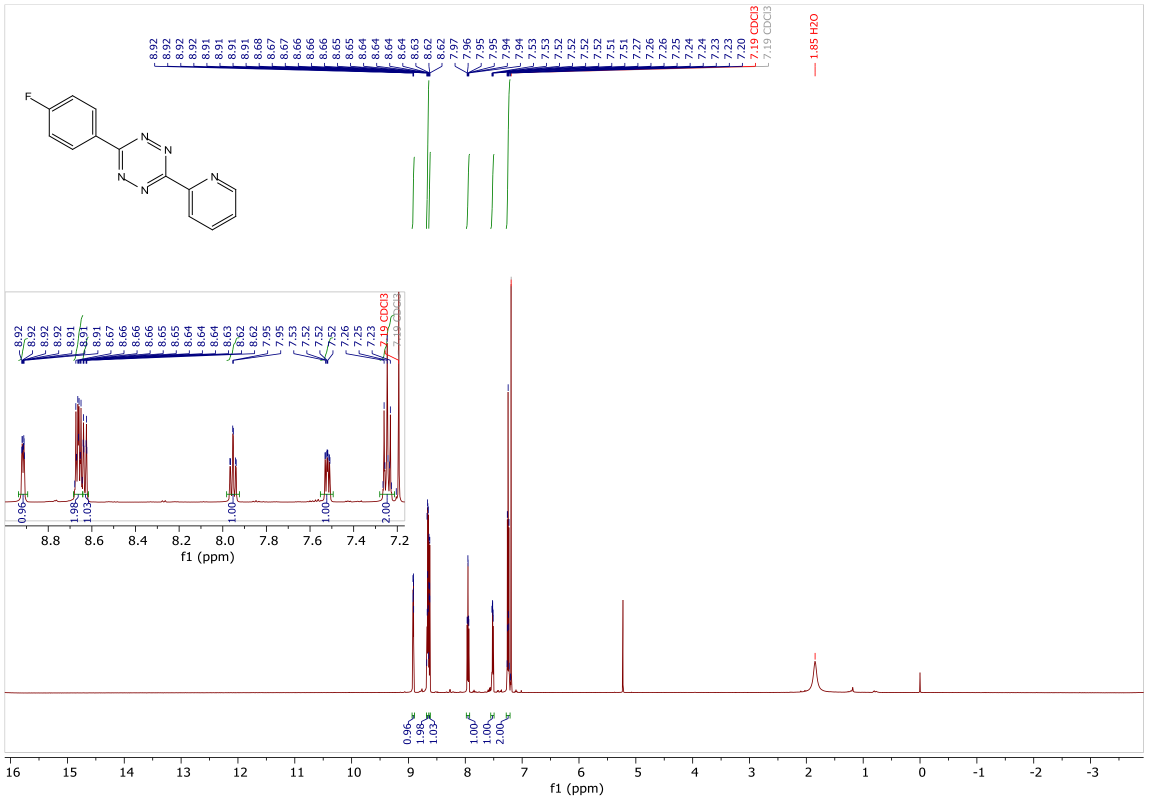

^{13}C NMR spectrum of 3-(4-fluorophenyl)-6-(pyridin-2-yl)-1,2,4,5-tetrazine (**10**) (151 MHz, CDCl3)

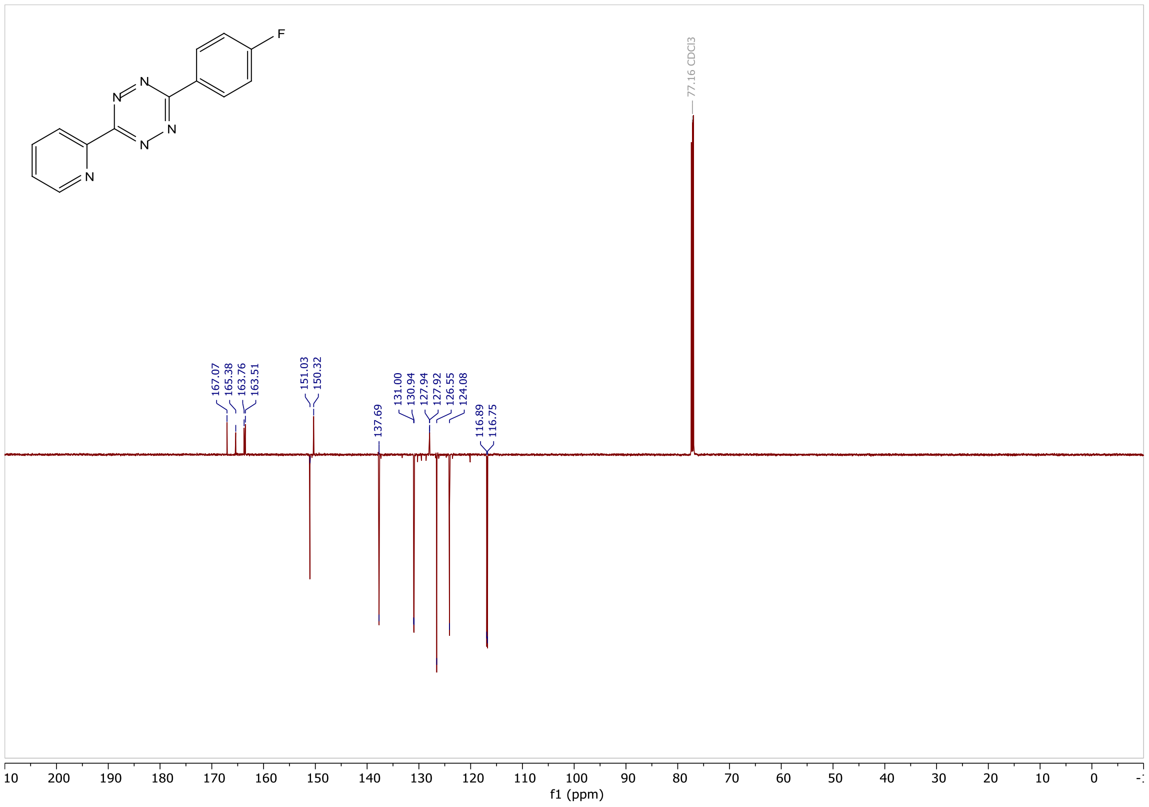

Analytical HPLC of 3-(4-fluorophenyl)-6-methyl-1,2,4,5-tetrazine (**10**)

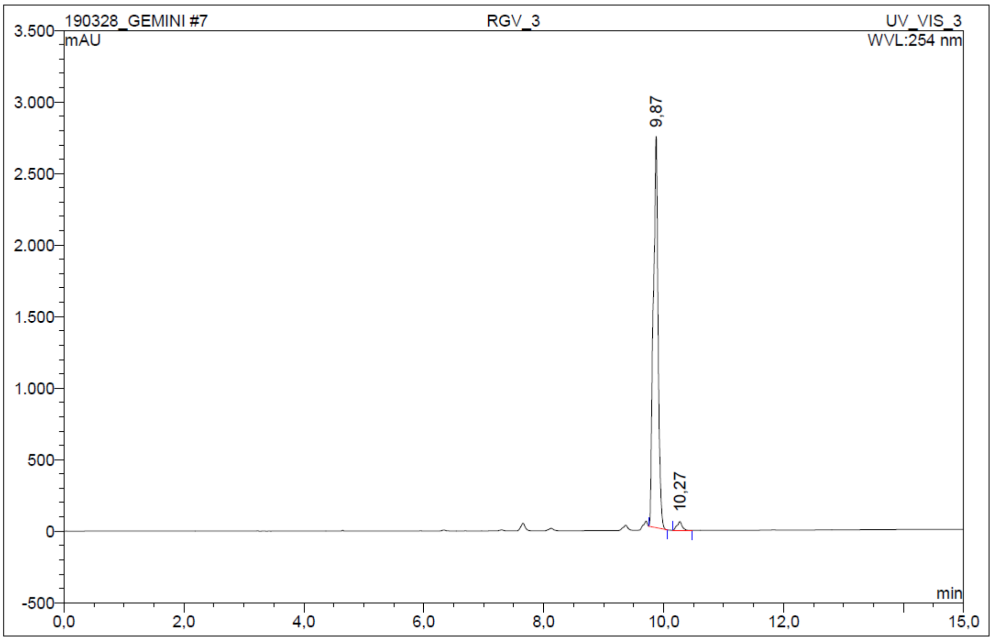

No.	Ret.Time min	Peak Name	Height mAU	Area mAU*min	Rel.Area %	Amount	Resolution(EP)
1	9,87	n.a.	2735,269	251,499	97,71	n.a.	2,58
2	10,27	n.a.	60,697	5,898	2,29	n.a.	n.a.
Total:			2795,966	257,397	100,00	0,000	

^{1}H NMR spectrum of 3-(5-fluoropyridin-2-yl)-6-(pyridin-2-yl)-1,2,4,5-tetrazine (**12**) (600 MHz, $CDCl_3$)

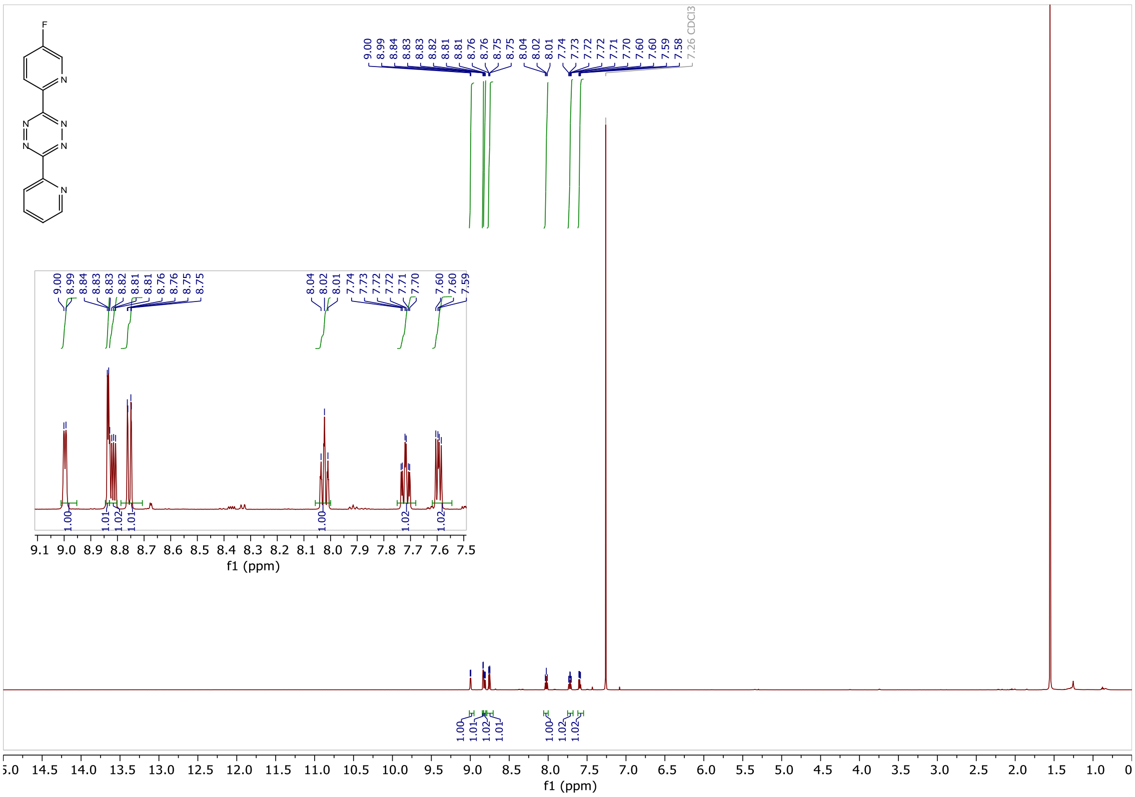

^{13}C NMR spectrum of 3-(5-fluoropyridin-2-yl)-6-(pyridin-2-yl)-1,2,4,5-tetrazine zine (**12**) (151 MHz, CDCl3)

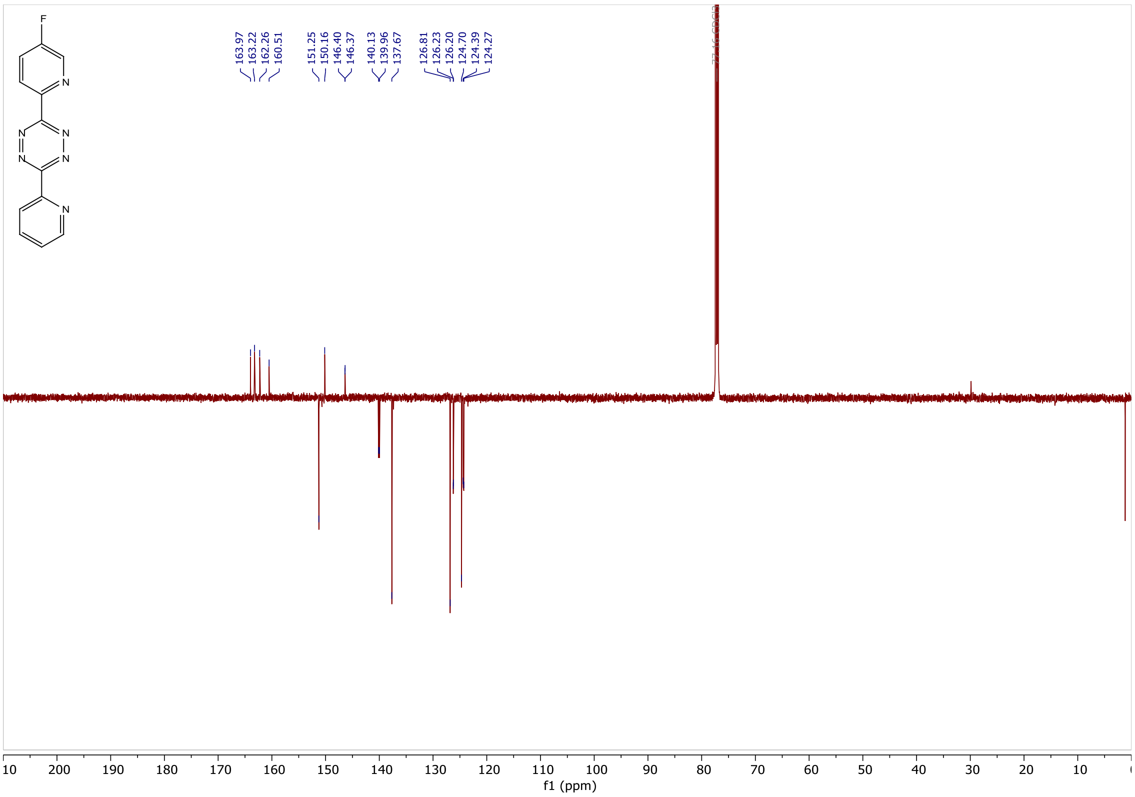

Analytical HPLC of 3-(5-fluoropyridin-2-yl)-6-(pyridin-2-yl)-1,2,4,5-tetrazine (**12**)

Chromatogram

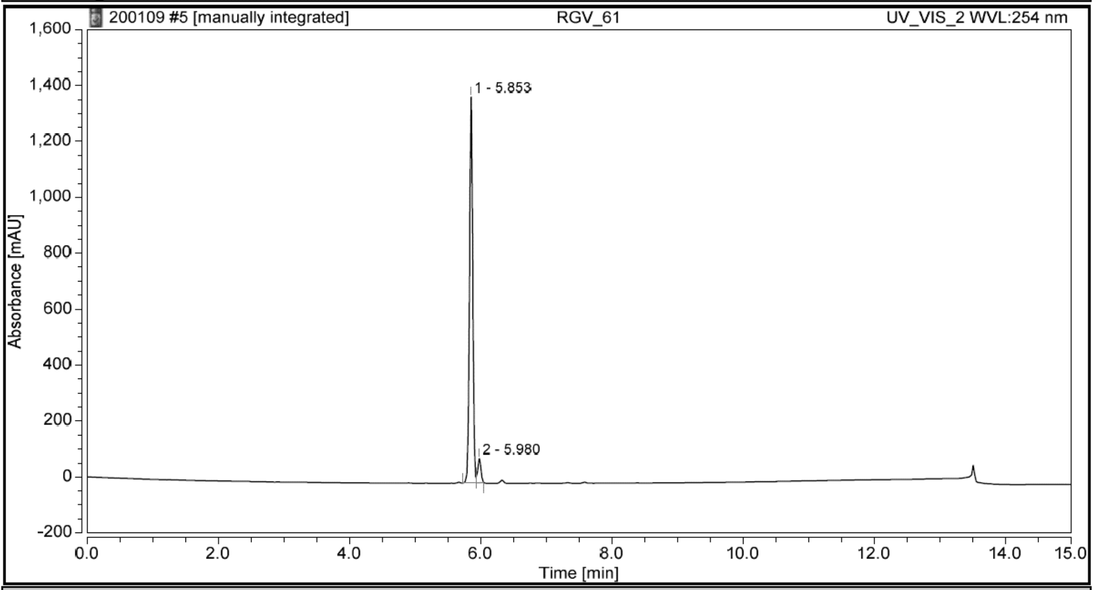

Integration Results

No.	Peak Name	Retention Time min	Area mAU*min	Height mAU	Relative Area %	Relative Height %	Amount
n.a.	Tosylate	n.a.	n.a.	n.a.	n.a.	n.a.	n.a.
n.a.	cold fallyprode	n.a.	n.a.	n.a.	n.a.	n.a.	n.a.
n.a.	18F-fallypride	n.a.	n.a.	n.a.	n.a.	n.a.	n.a.
1		5.853	79.090	1380.486	94.20	94.12	n.a.
2		5.980	4.870	86.220	5.80	5.88	n.a.
**Total:**			**83.960**	**1466.706**	**100.00**	**100.00**	

^{1}H NMR spectrum of 3-(4-iodophenyl)-6-(pyridin-2-yl)-1,2-dihydro-1,2,4,5-tetrazine (**25a**) (400 MHz, $CDCl_3$)

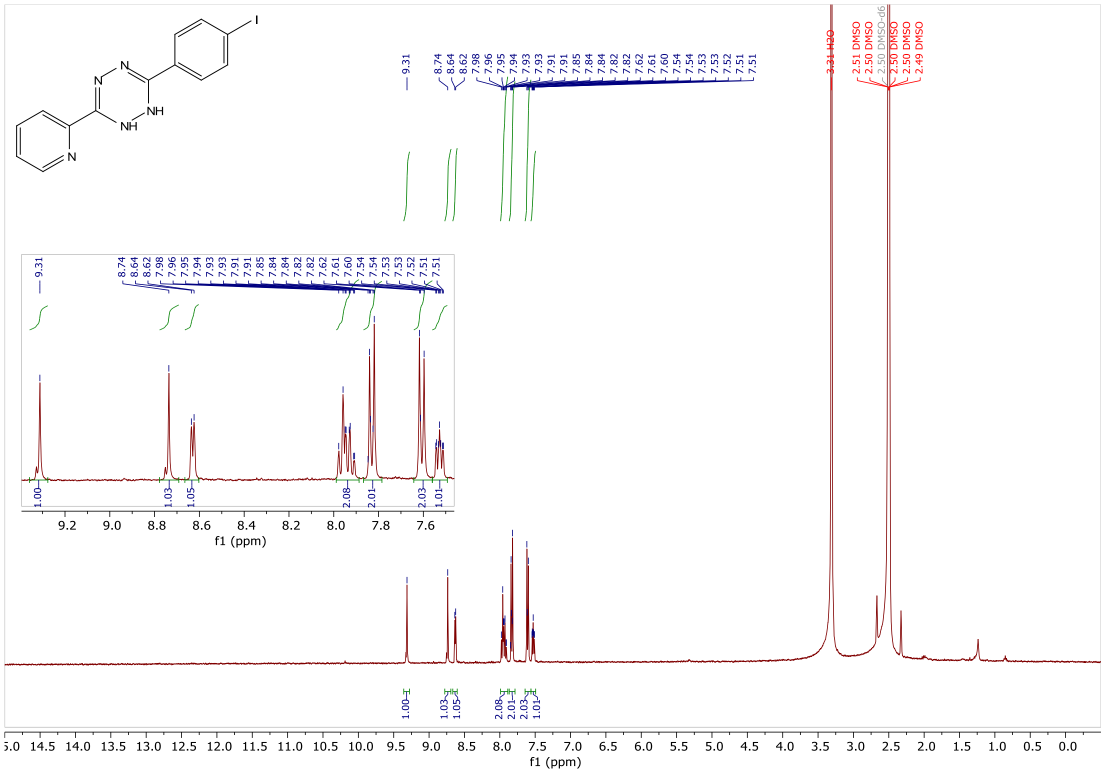

^{13}C NMR spectrum of 3-(4-iodophenyl)-6-(pyridin-2-yl)-1,2-dihydro-1,2,4,5-tetrazine (**25a**) (101 MHz, CDCl3)

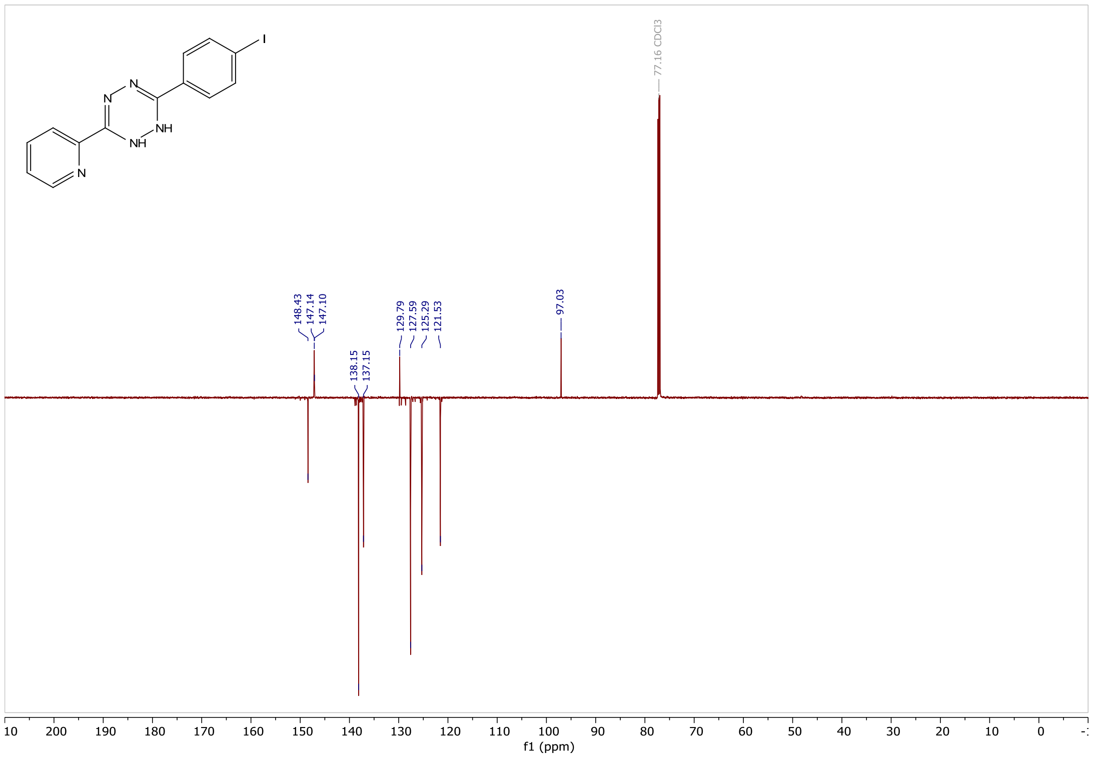

^{1}H NMR spectrum of 3-(5-iodopyridin-2-yl)-6-(pyridin-2-yl)-1,2-dihydro-1,2,4,5-tetrazine (**26a**) (600 MHz, $CDCl_3$)

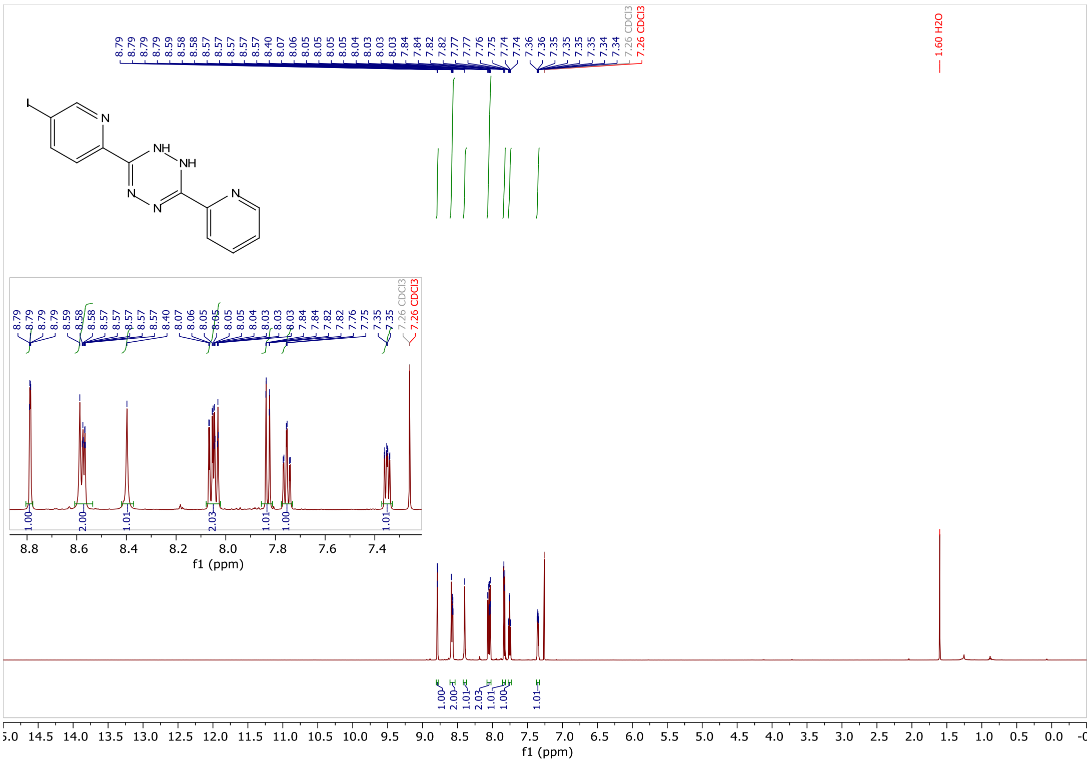

^{13}C NMR spectrum of 3-(5-iodopyridin-2-yl)-6-(pyridin-2-yl)-1,2-dihydro-1,2,4,5-tetrazine (**26a**) (151 MHz, $CDCl_3$)

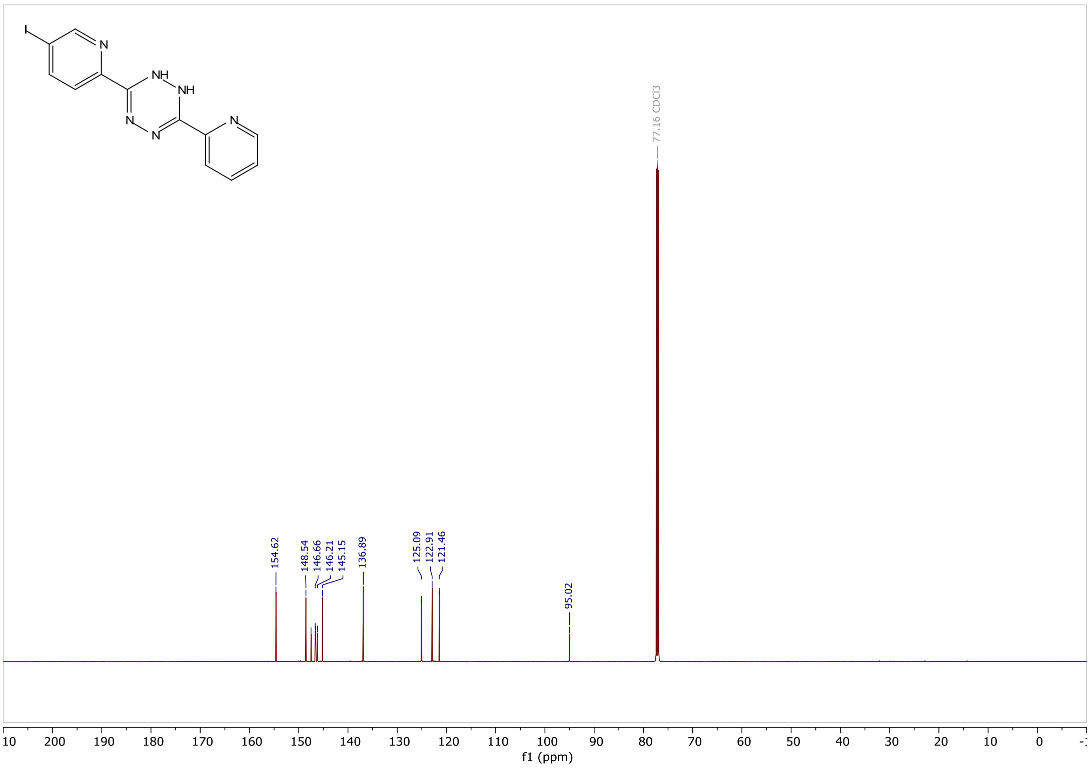

^{1}H NMR spectrum of 3-(4-fluorophenyl)-1,2,4,5-tetrazine (**11**) (400 MHz, $CDCl_3$)

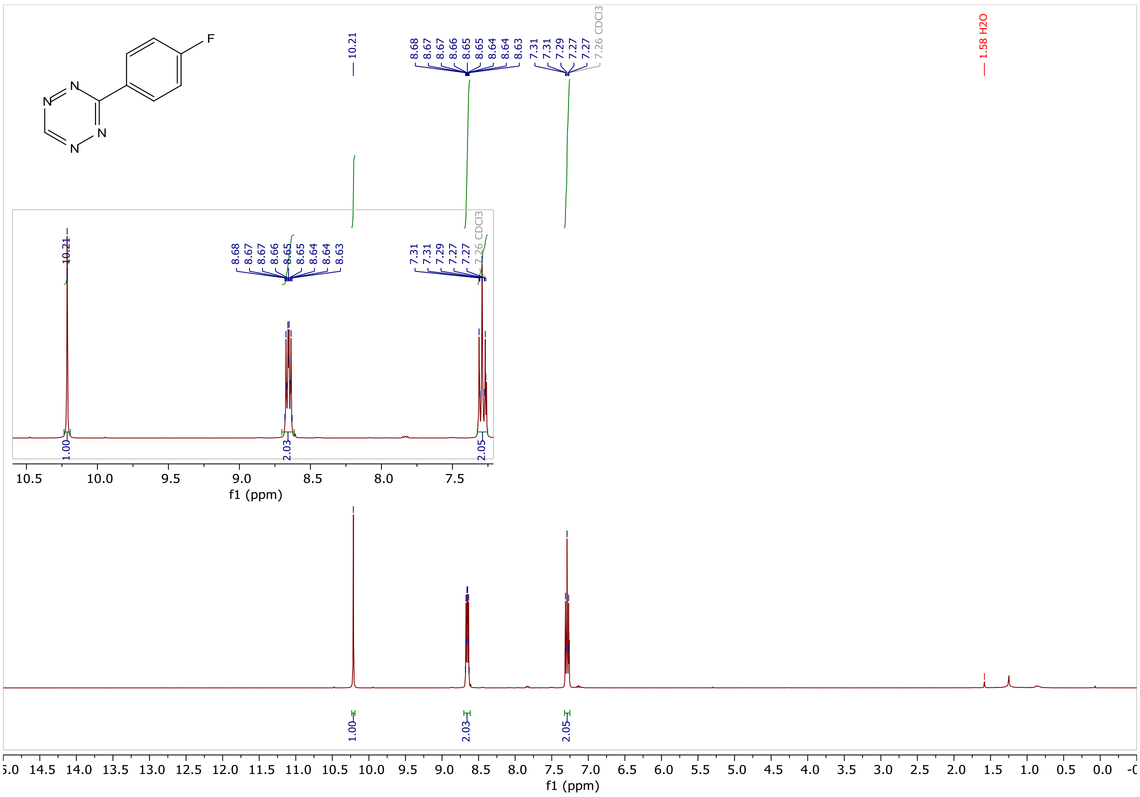

^{13}C NMR spectrum of 3-(4-fluorophenyl)-1,2,4,5-tetrazine (**11**) (101 MHz, CDCl3)

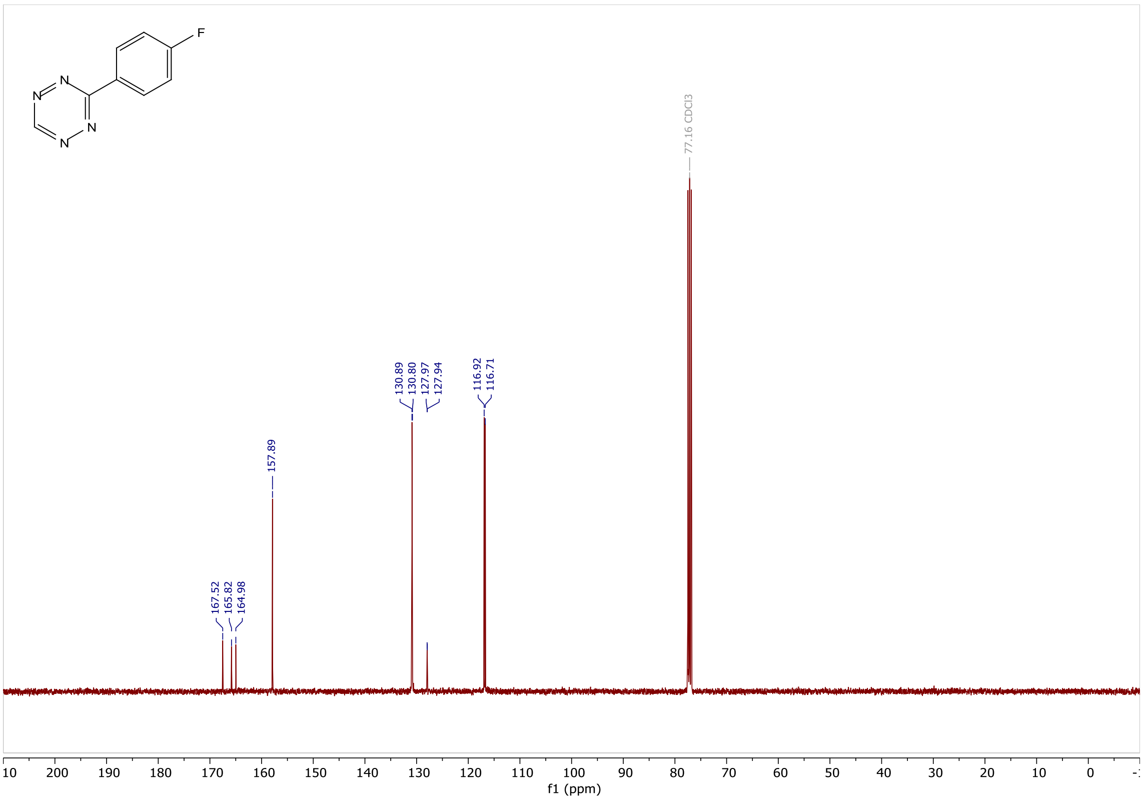

Analytical HPLC of 3-(4-fluorophenyl)-1,2,4,5-tetrazine (**11**)

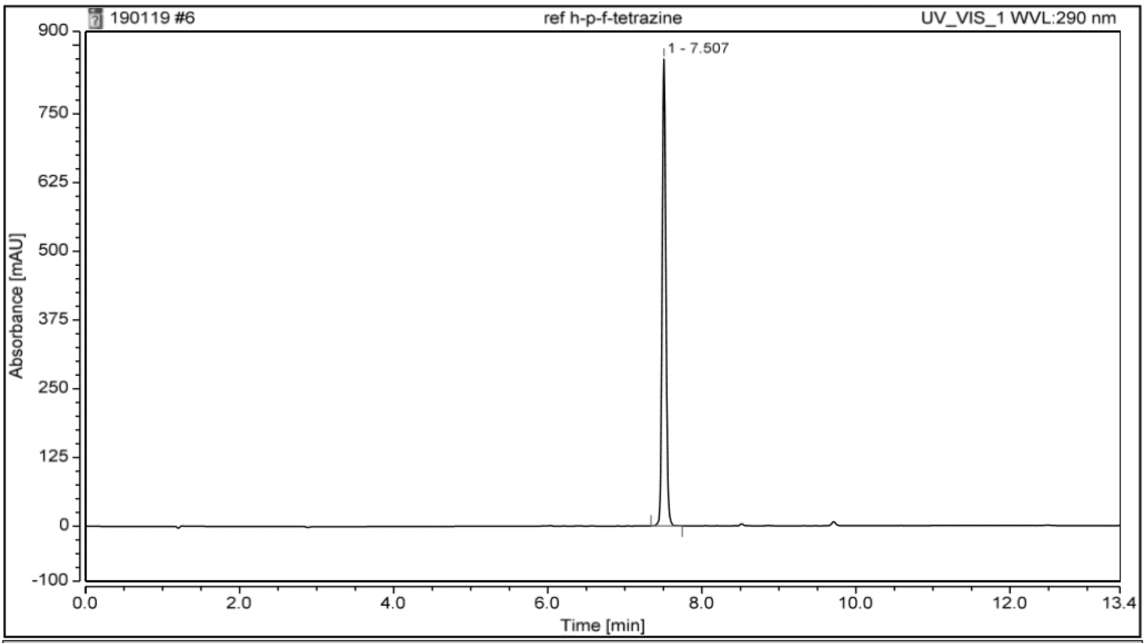

Integration Results

No.	Peak Name	Retention Time min	Area mAU*min	Height mAU	Relative Area %	Relative Height %
1		7.507	47.322	849.159	100.00	100.00
**Total:**			**47.322**	**849.159**	**100.00**	**100.00**

^{1}H NMR spectrum of 3-(4-iodophenyl)-1,2,4,5-tetrazine (**27a**) (400 MHz, $CDCl_3$)

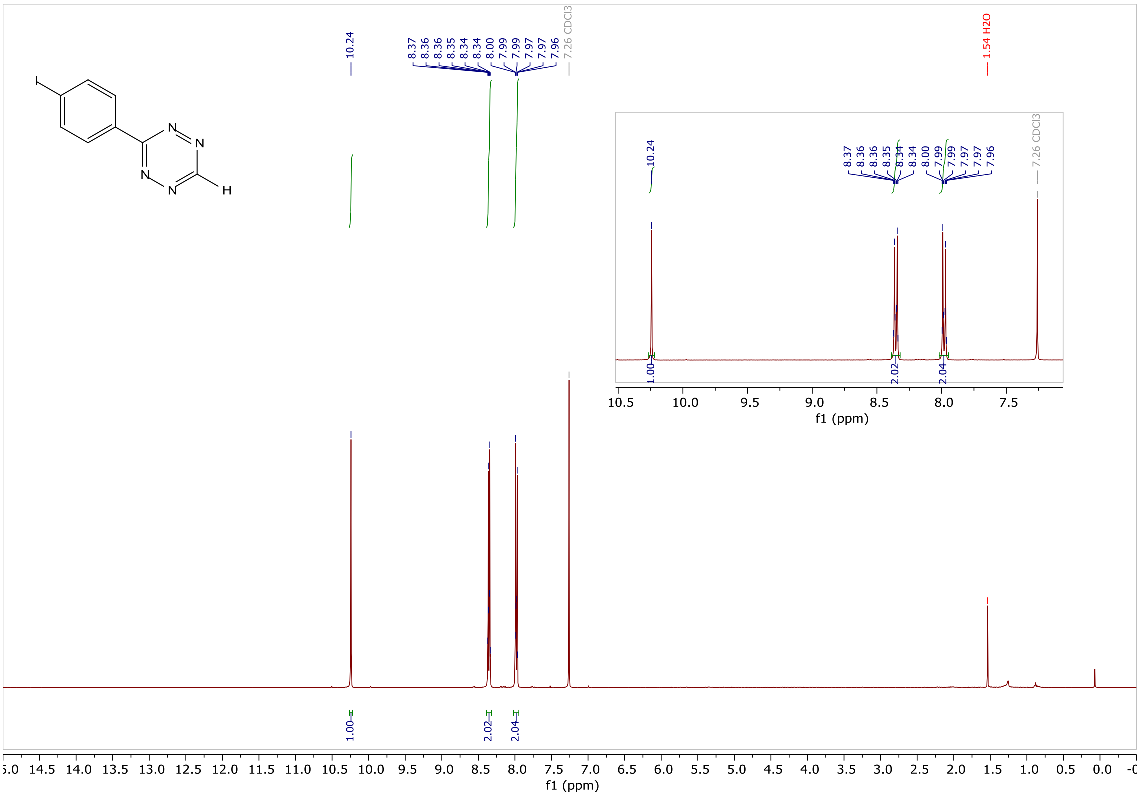

^{13}C NMR spectrum of 3-(4-iodophenyl)-1,2,4,5-tetrazine (**27a**) (101 MHz, CDCl3)

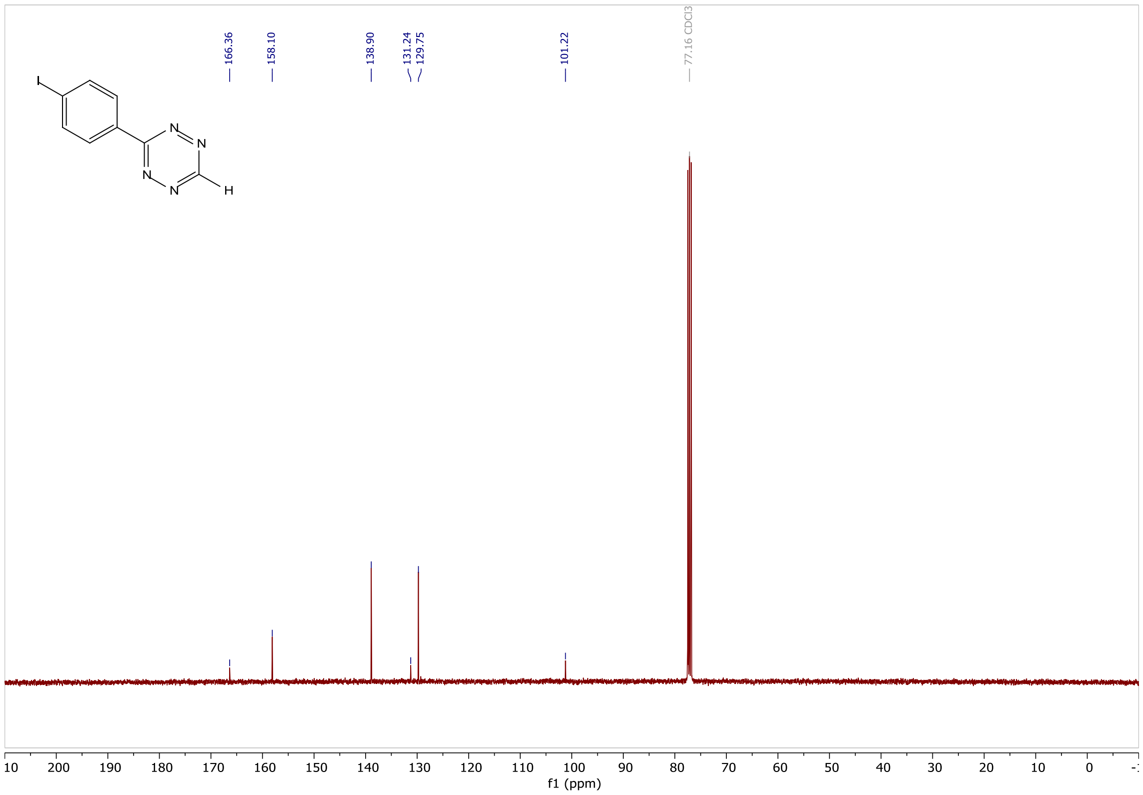

^{1}H NMR spectrum of 3-(3-fluorophenyl)-1,2,4,5-tetrazine (**13**) (400 MHz, CDCl3)

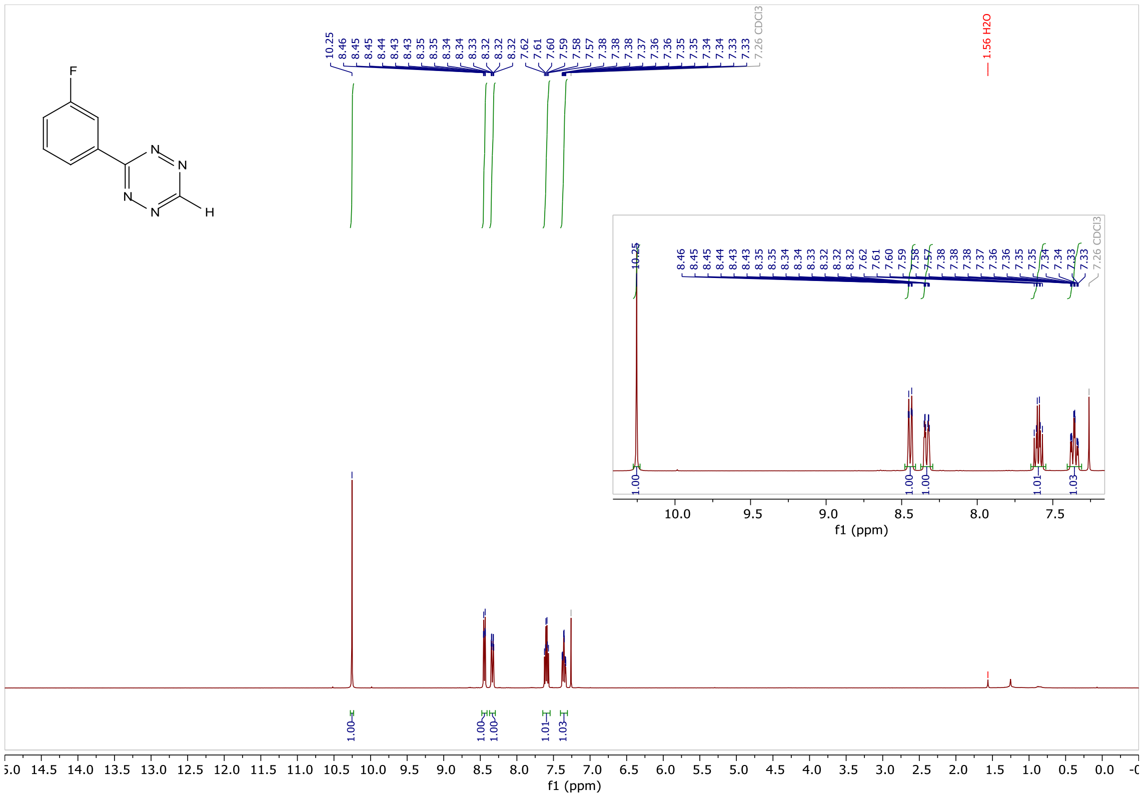

^{13}C NMR spectrum of 3-(3-fluorophenyl)-1,2,4,5-tetrazine (**13**) (101 MHz, CDCl3)

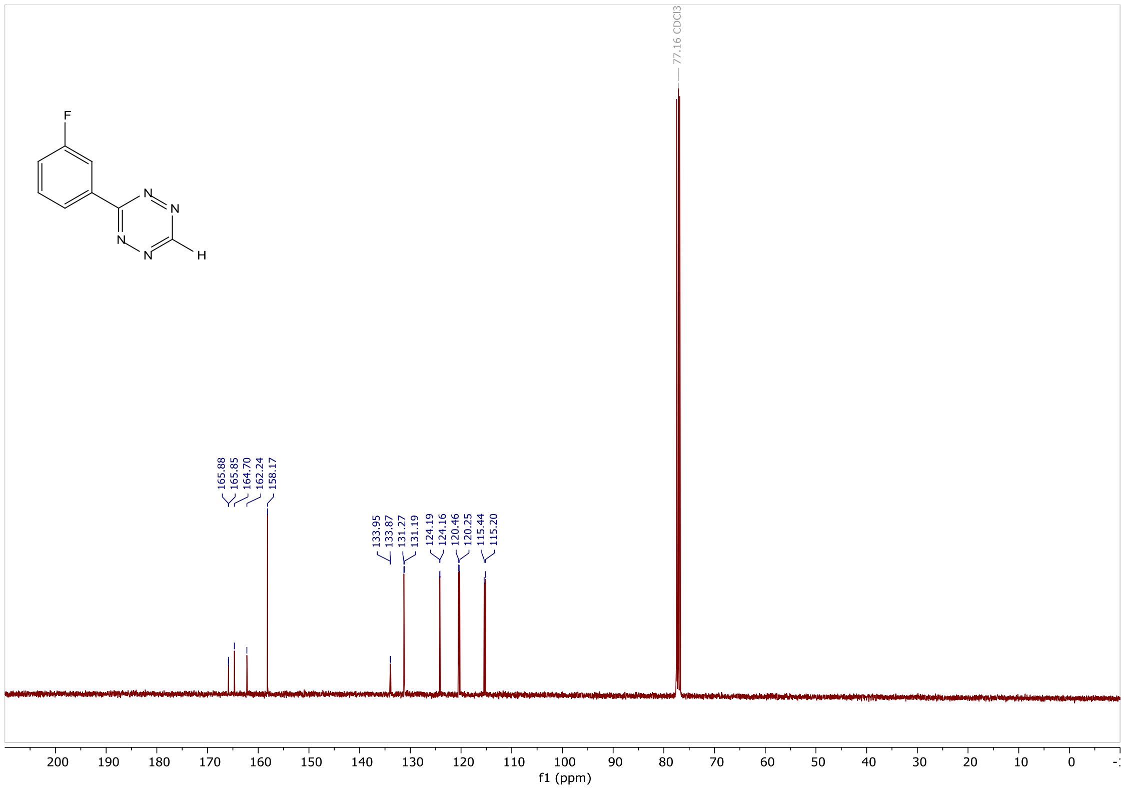

Analytical HPLC of 3-(3-fluorophenyl)-1,2,4,5-tetrazine (**13**)

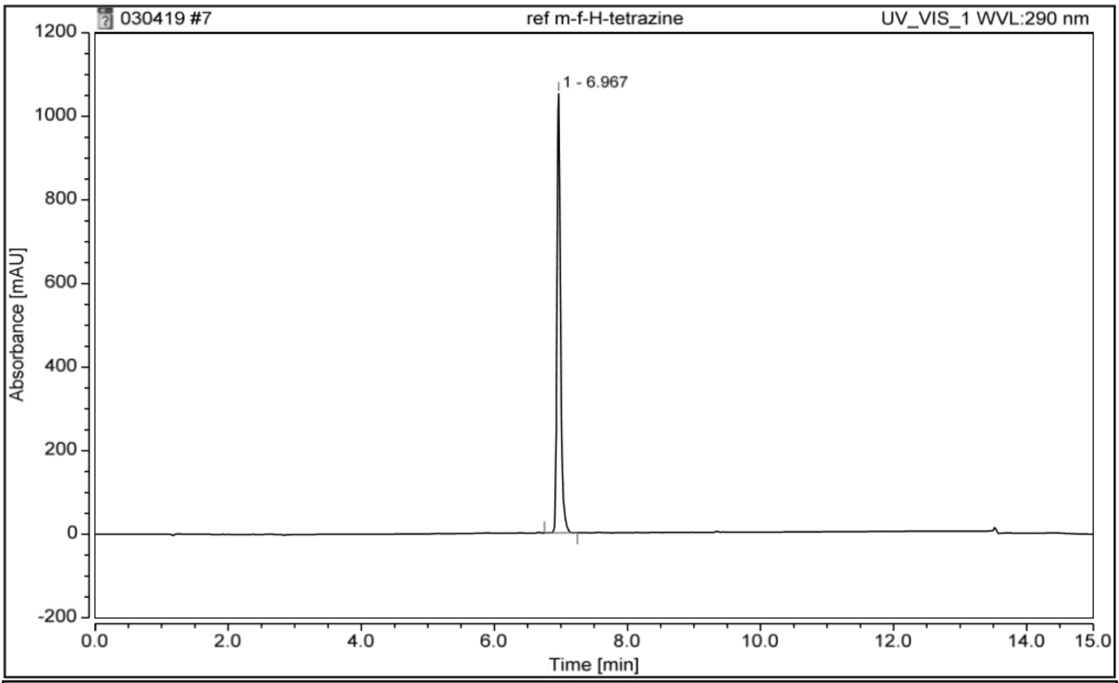

Integration Results

No.	Peak Name	Retention Time min	Area mAU*min	Height mAU	Relative Area %	Relative Height %
1		6.967	65.196	1050.848	100.00	100.00
**Total:**			**65.196**	**1050.848**	**100.00**	**100.00**

^{1}H NMR spectrum of 3-(3-iodophenyl)-1,2,4,5-tetrazine (**28a**) (400 MHz, $CDCl_3$)

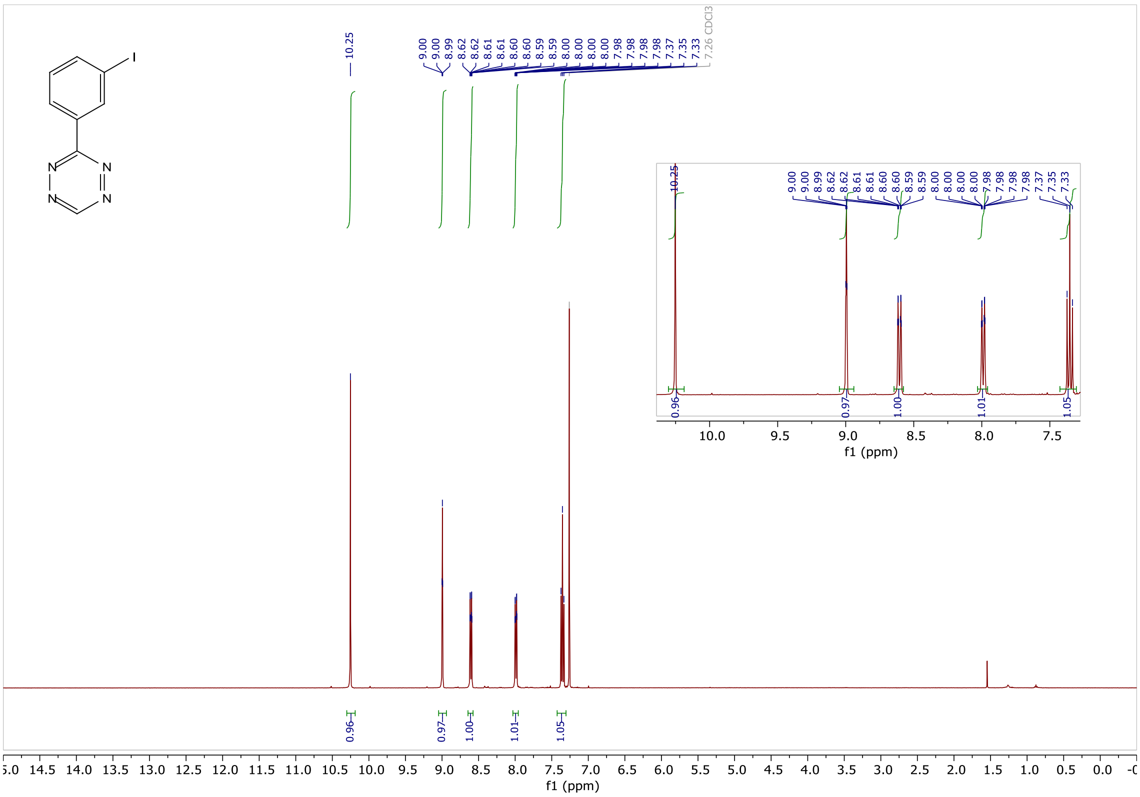

^{13}C NMR spectrum of 3-(3-iodophenyl)-1,2,4,5-tetrazine (**28a**) (101 MHz, CDCl3)

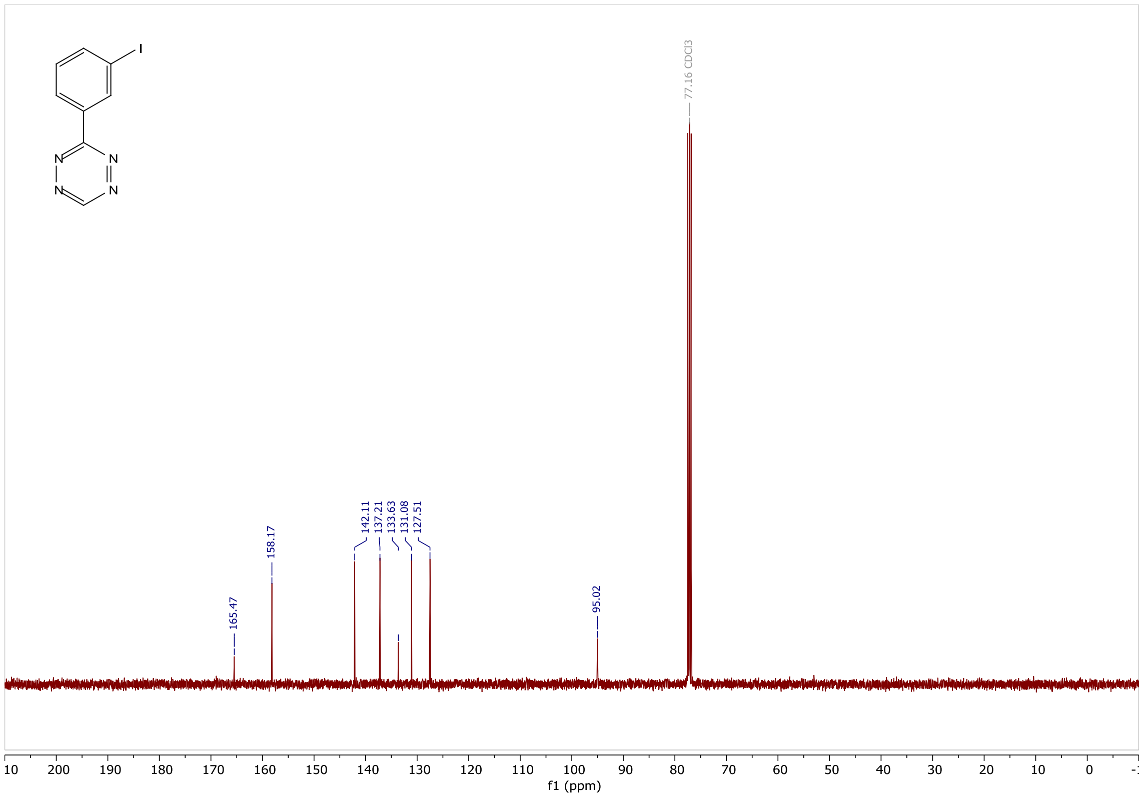

^{1}H NMR spectrum of 3-(3-fluoro-4-methylphenyl)-1,2,4,5-tetrazine (**14-*p***) (400 MHz, $CDCl_3$)

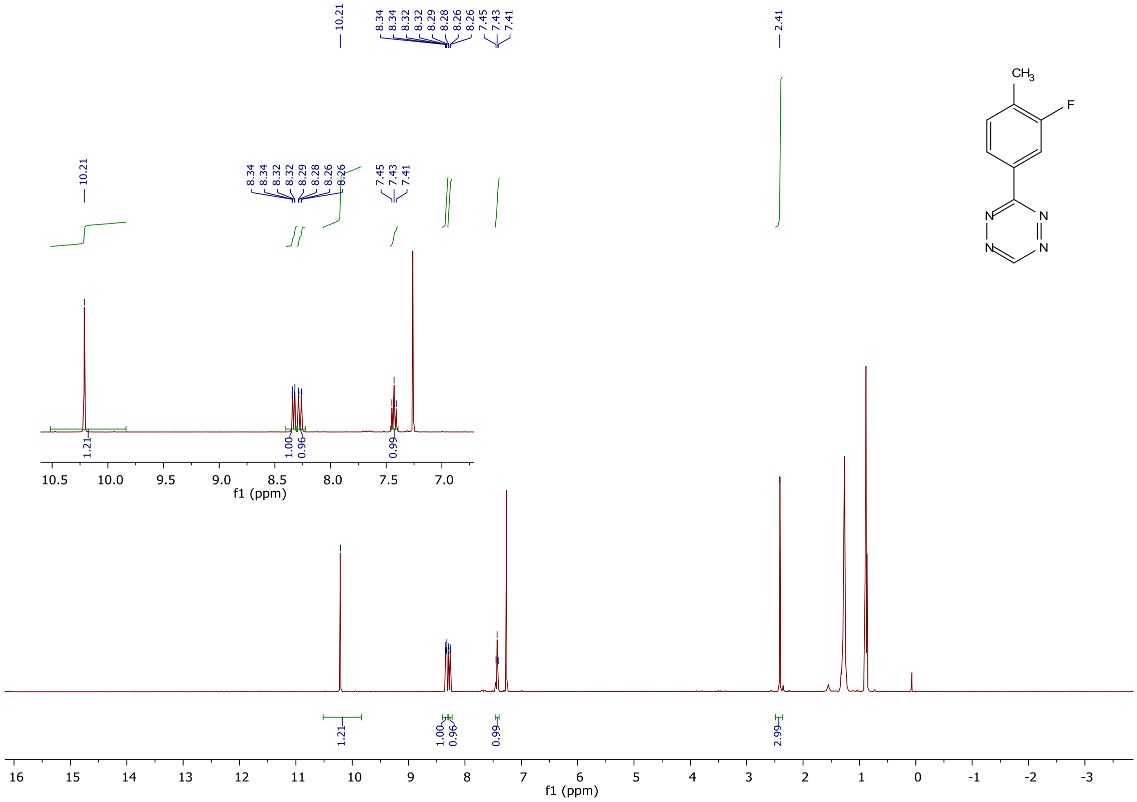

^{13}C NMR spectrum of 3-(3-fluoro-4-methylphenyl)-1,2,4,5-tetrazine (**14-*p***) (101 MHz, CDCl3)

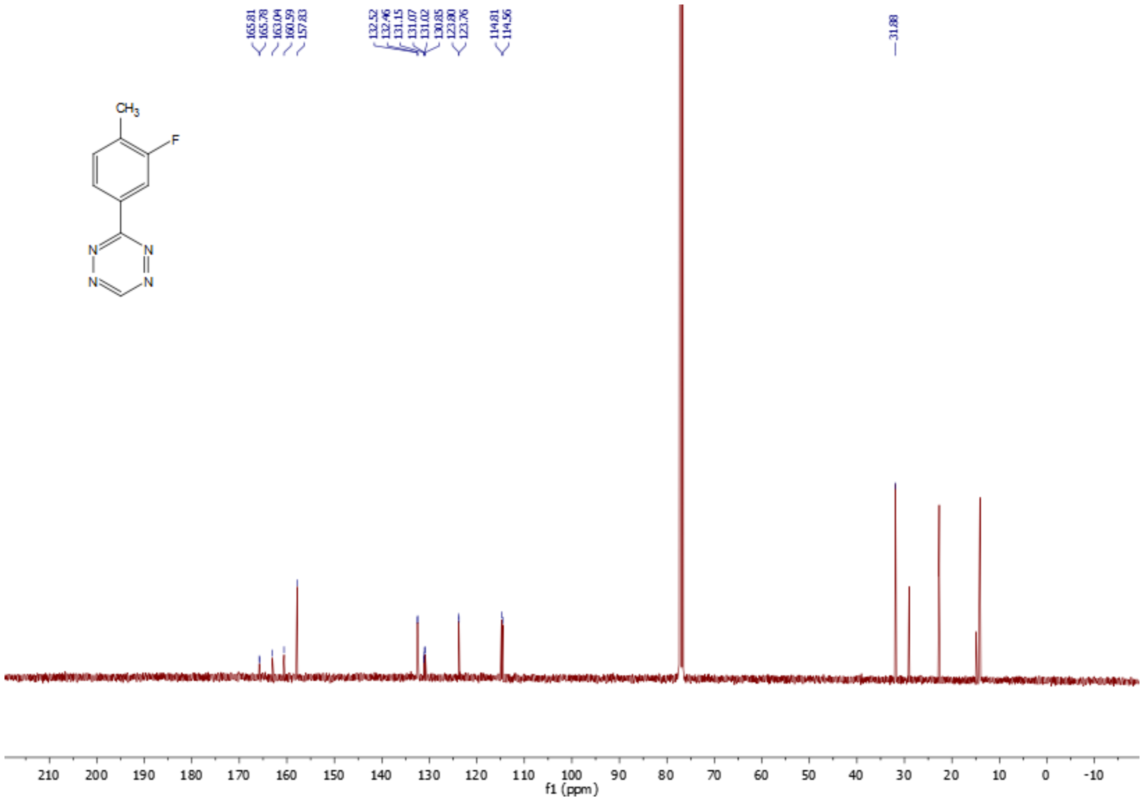

Analytical HPLC of 3-(4-fluorophenyl)-6-methyl-1,2,4,5-tetrazine (**14-*p***)

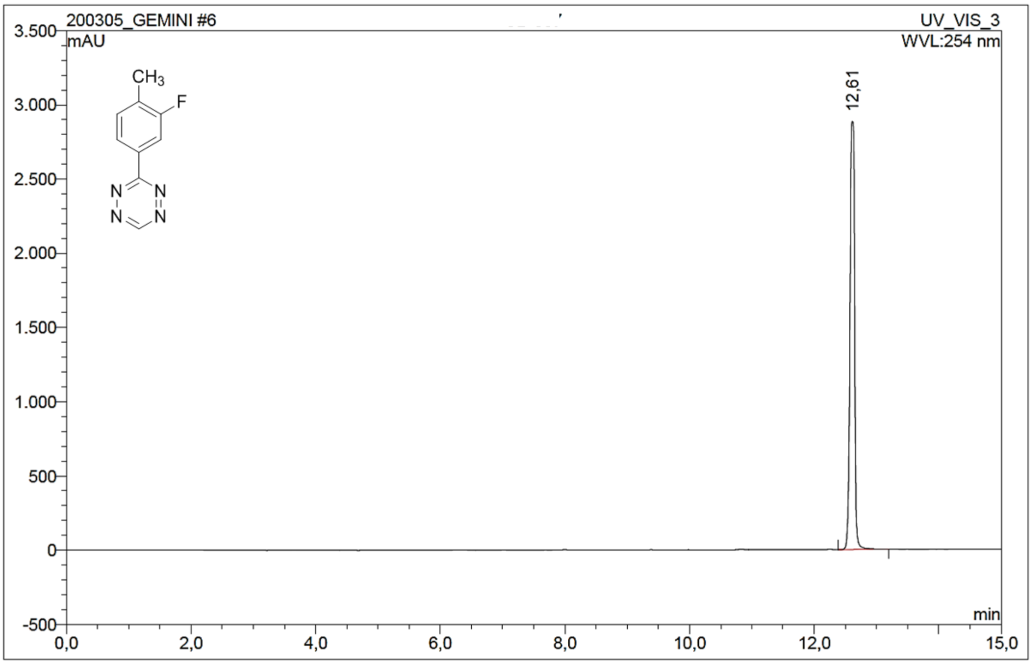

No.	Ret.Time min	Peak Name	Height mAU	Area mAU*min	Rel.Area %	Amount	Resolution(EP)
1	12,61	n.a.	2884,966	257,366	100,00	n.a.	n.a.
Total:			2884,966	257,366	100,00	0,000	

^{1}H NMR spectrum of 3-(3-iodo-4-methylphenyl)-1,2,4,5-tetrazine (**29a**) (600 MHz, $CDCl_3$)

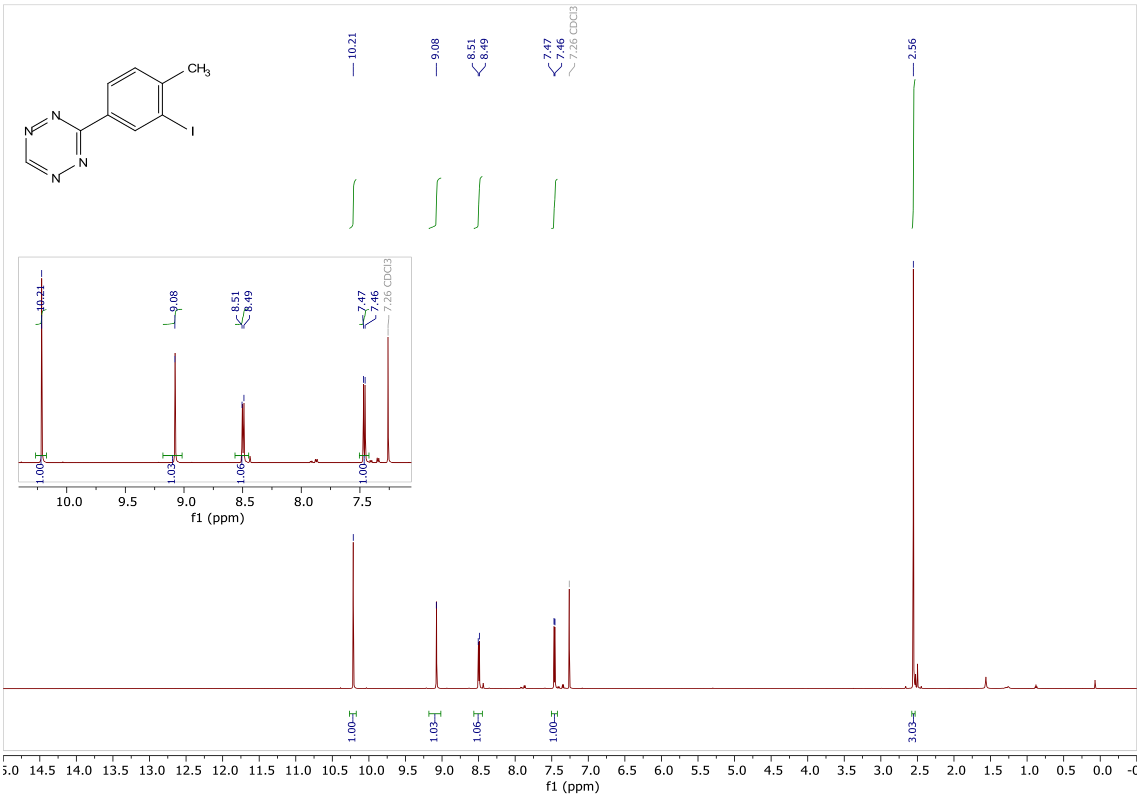

^{13}C NMR spectrum of 3-(3-iodo-4-methylphenyl)-1,2,4,5-tetrazine (**29a**) (101 MHz, CDCl3)

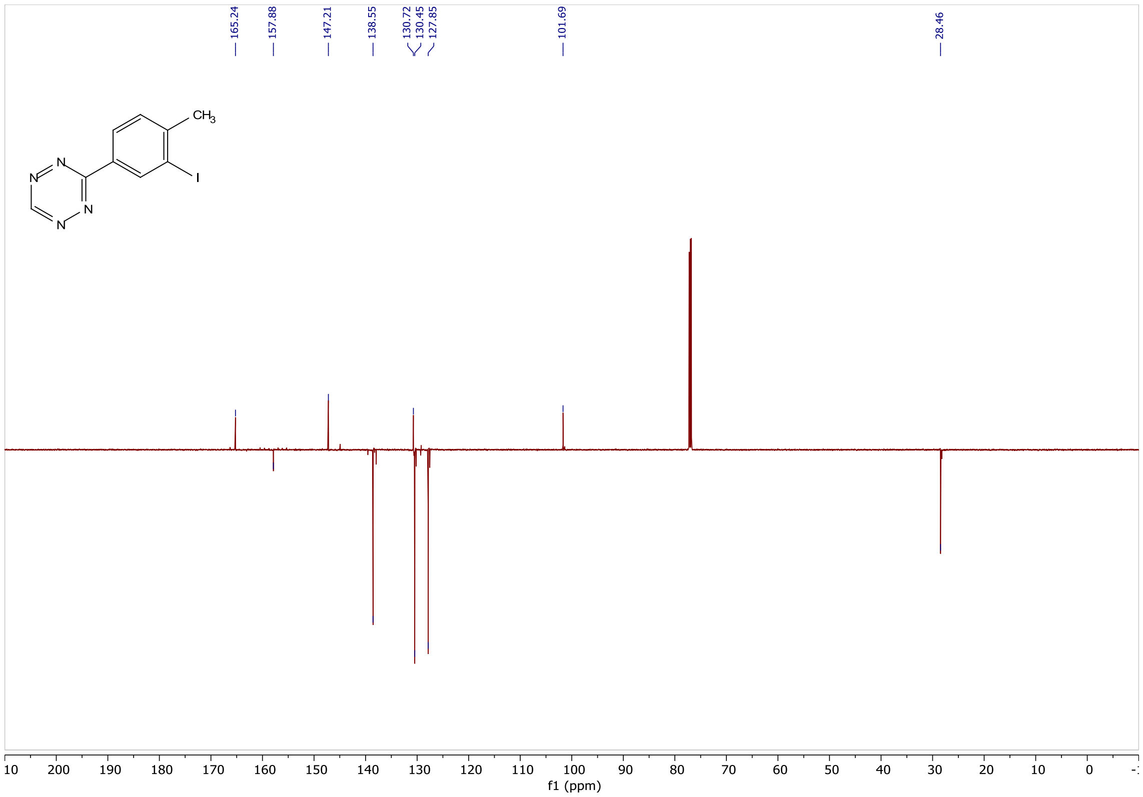

¹H NMR spectrum of 3-(3-fluoro-4-methoxyphenyl)-1,2,4,5-tetrazine (**15-*p***) (400 MHz, $CDCl_3$)

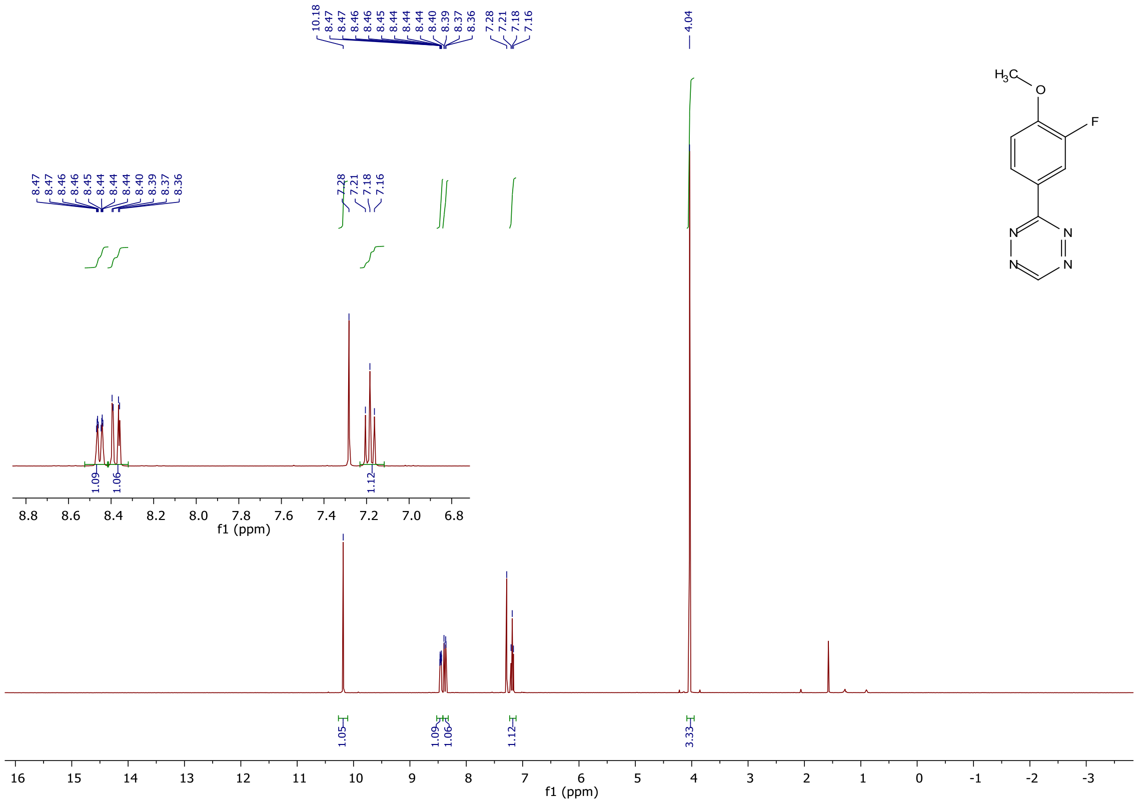

^{13}C NMR spectrum of 3-(3-fluoro-4-methoxyphenyl)-1,2,4,5-tetrazine (**15-*p***) (101 MHz, CDCl3)

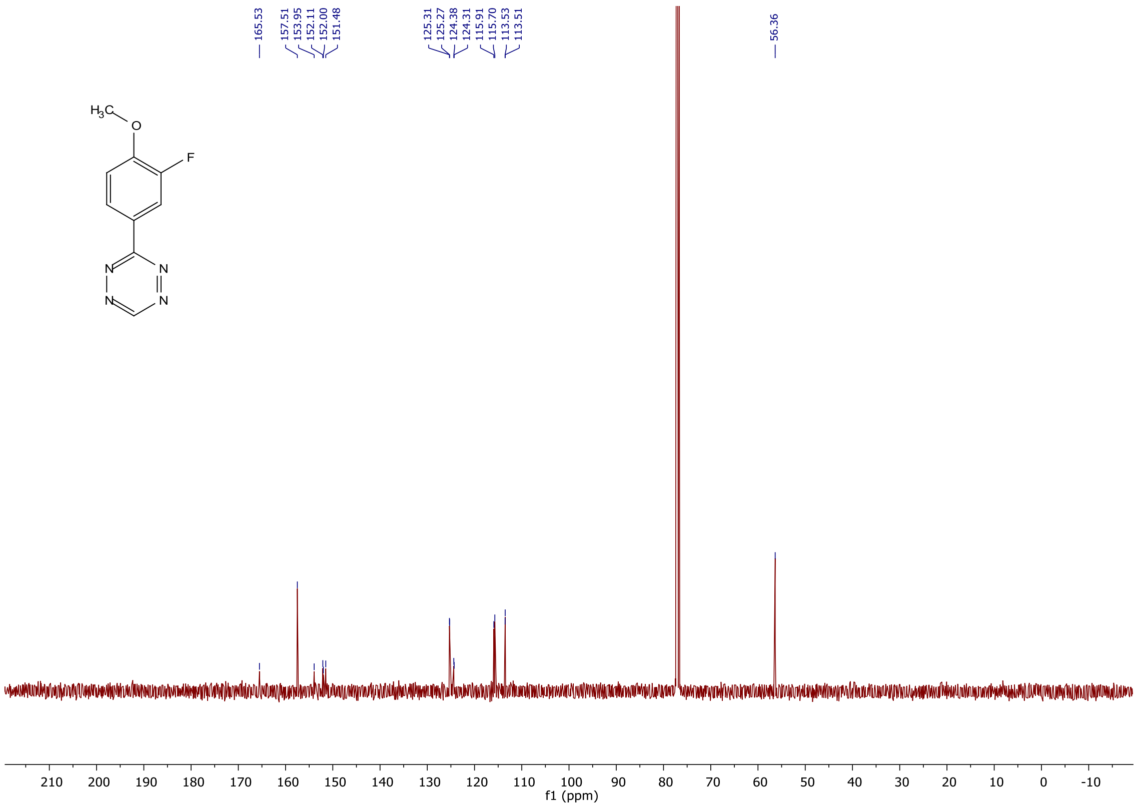

Analytical HPLC of 3-(3-fluoro-4-methoxyphenyl)-1,2,4,5-tetrazine (**15-*p***)

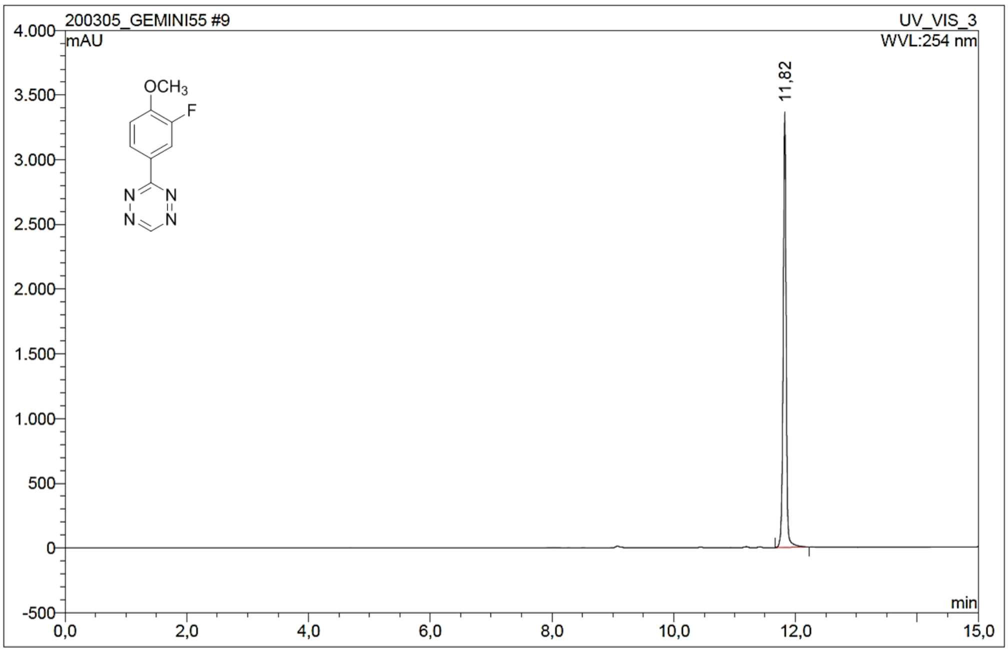

No.	Ret.Time min	Peak Name	Height mAU	Area mAU*min	Rel.Area %	Amount	Resolution(EP)
1	11,82	n.a.	3364,798	196,865	100,00	n.a.	n.a.
**Total:**			3364,798	196,865	100,00	0,000	

^{1}H NMR spectrum of 3-(3-iodo-4-methoxyphenyl)-1,2,4,5-tetrazine (**15-*p***) (400 MHz, $CDCl_3$)

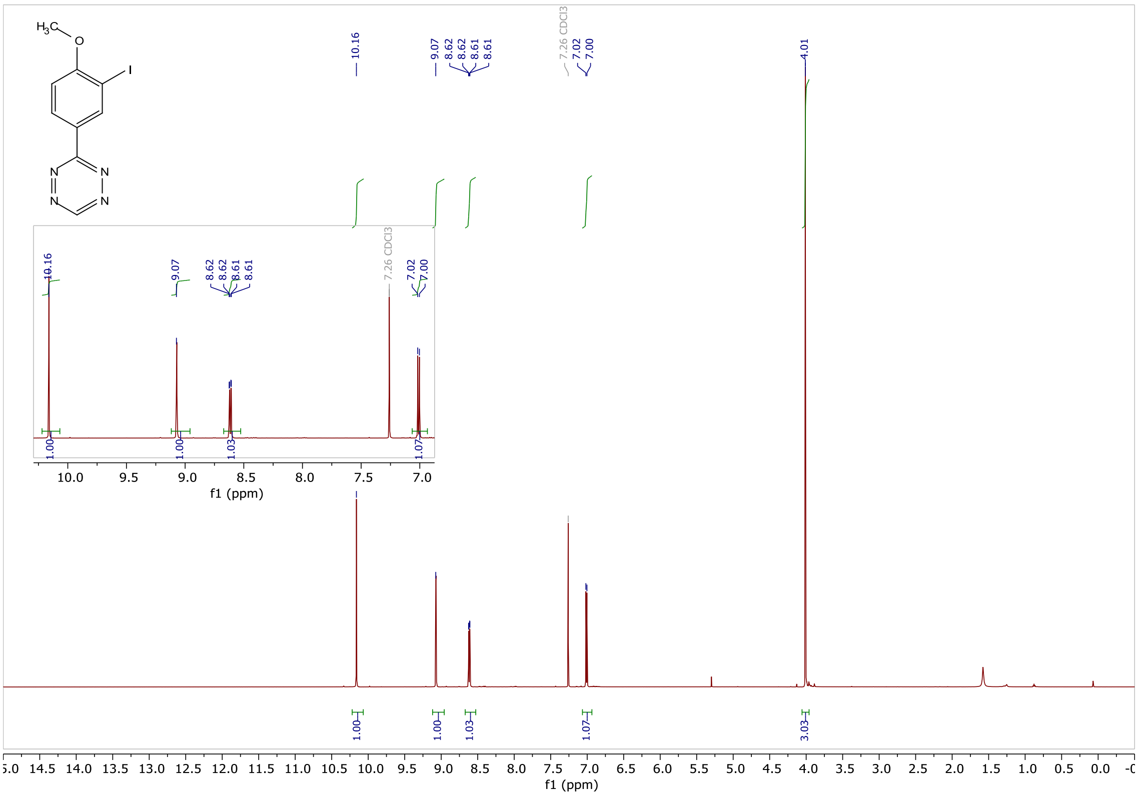

^{13}C NMR spectrum of 3-(3-iodo-4-methoxyphenyl)-1,2,4,5-tetrazine (**30c**) (101 MHz, CDCl3)

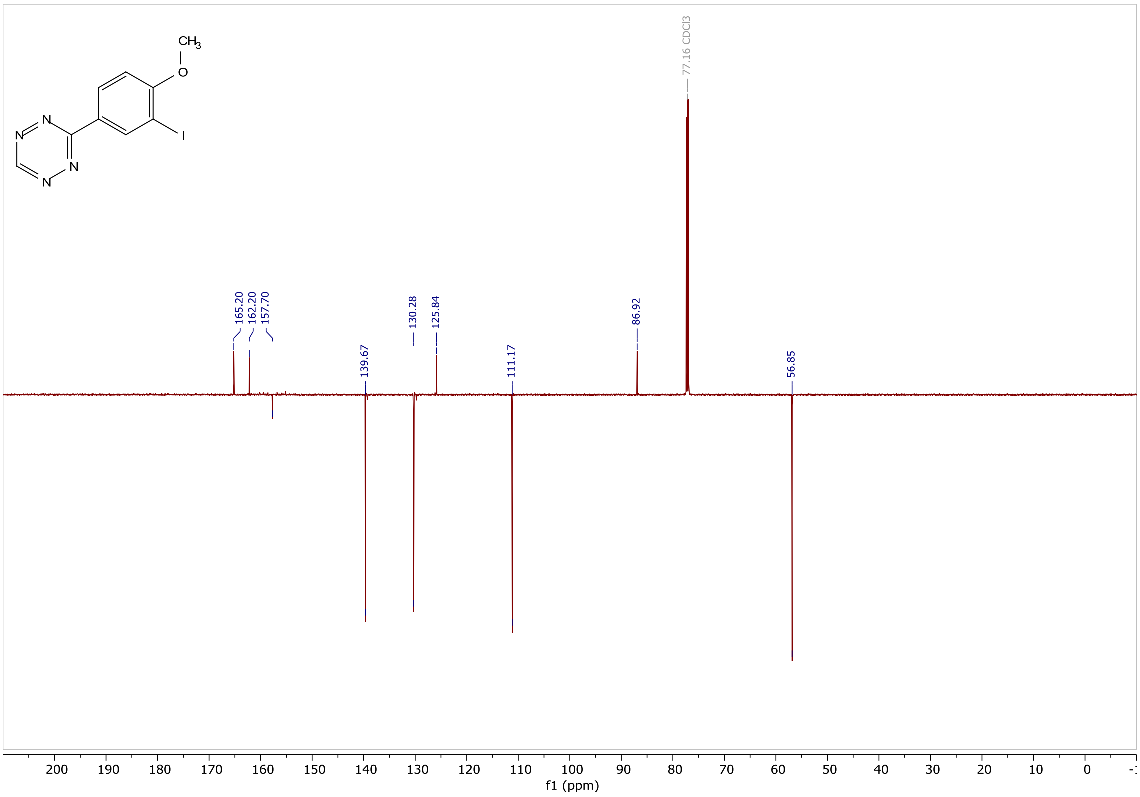

^{1}H NMR spectrum of N-(4-cyano-2-fluorophenyl)acetamide (**16a-*p***) (400 MHz, DMSO-d_6)

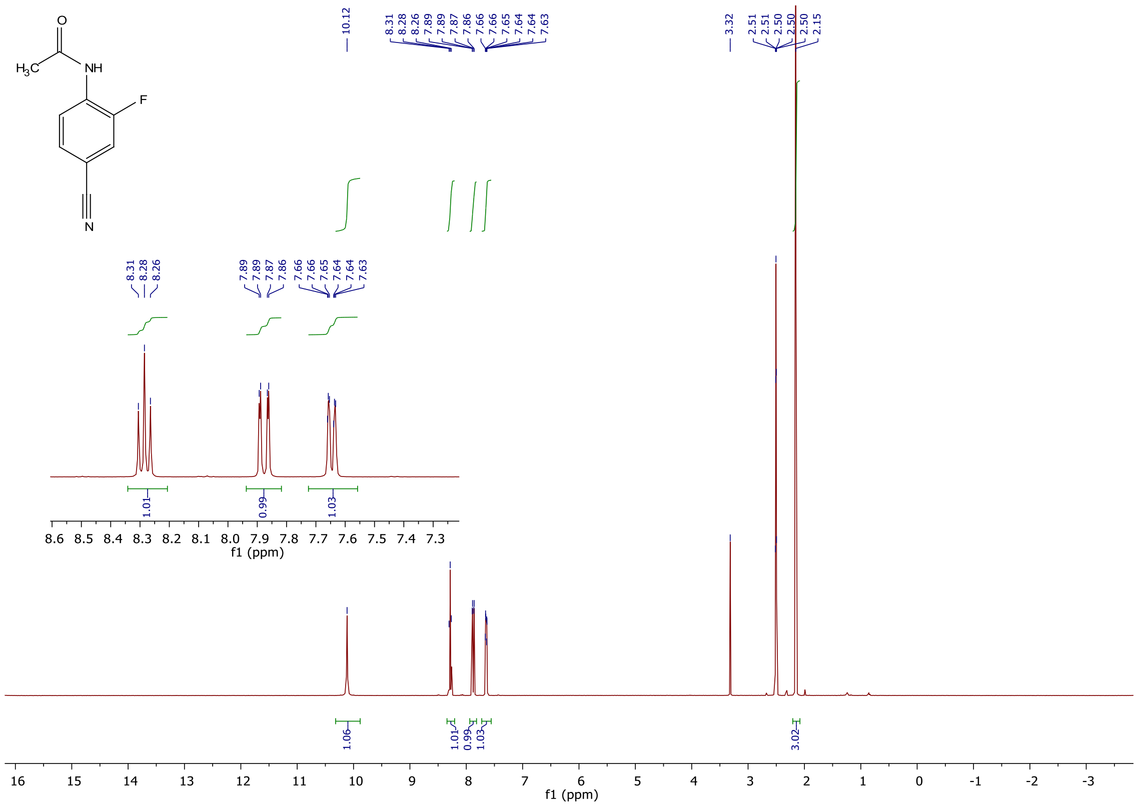

^{13}C NMR spectrum of of N-(4-cyano-2-fluorophenyl)acetamide (**16a-*p***) (101 MHz, DMSO-d_6)

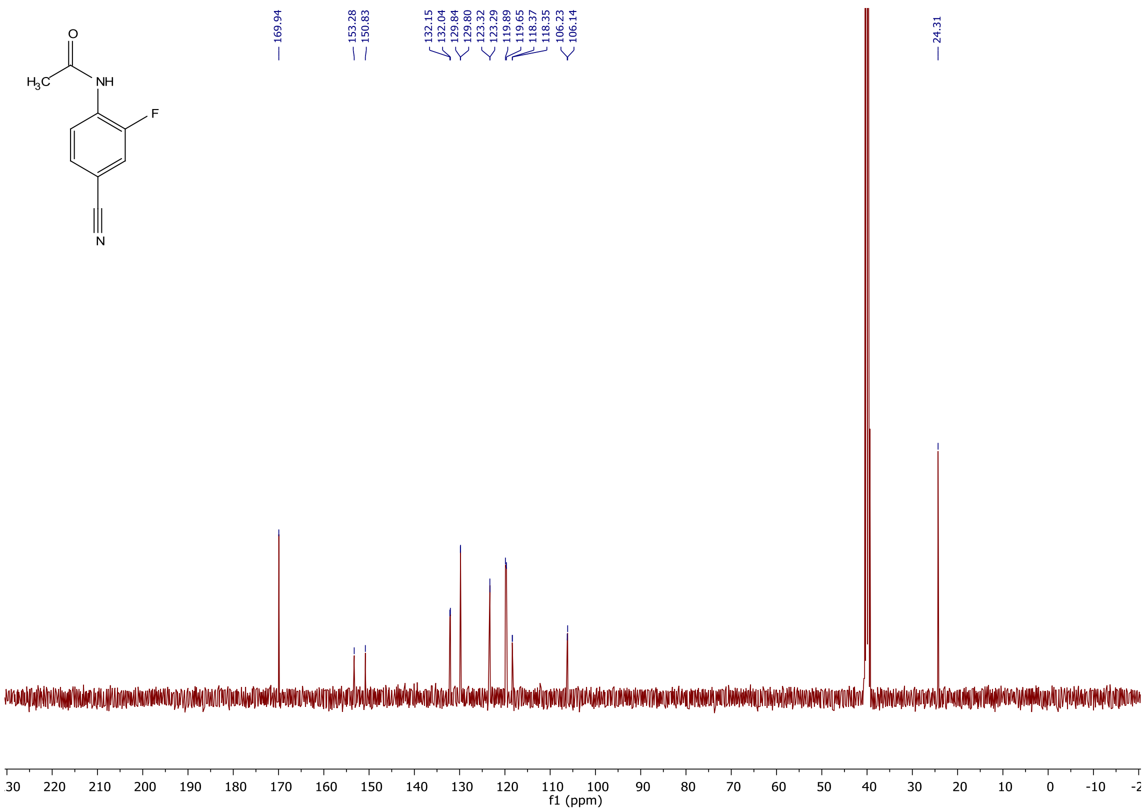

^{1}H NMR spectrum of N-(2-Fluoro-4-(1,2,4,5-tetrazin-3-yl)phenyl)acetamide (**16-*p***) (400 MHz, DMSO-d$_6$)

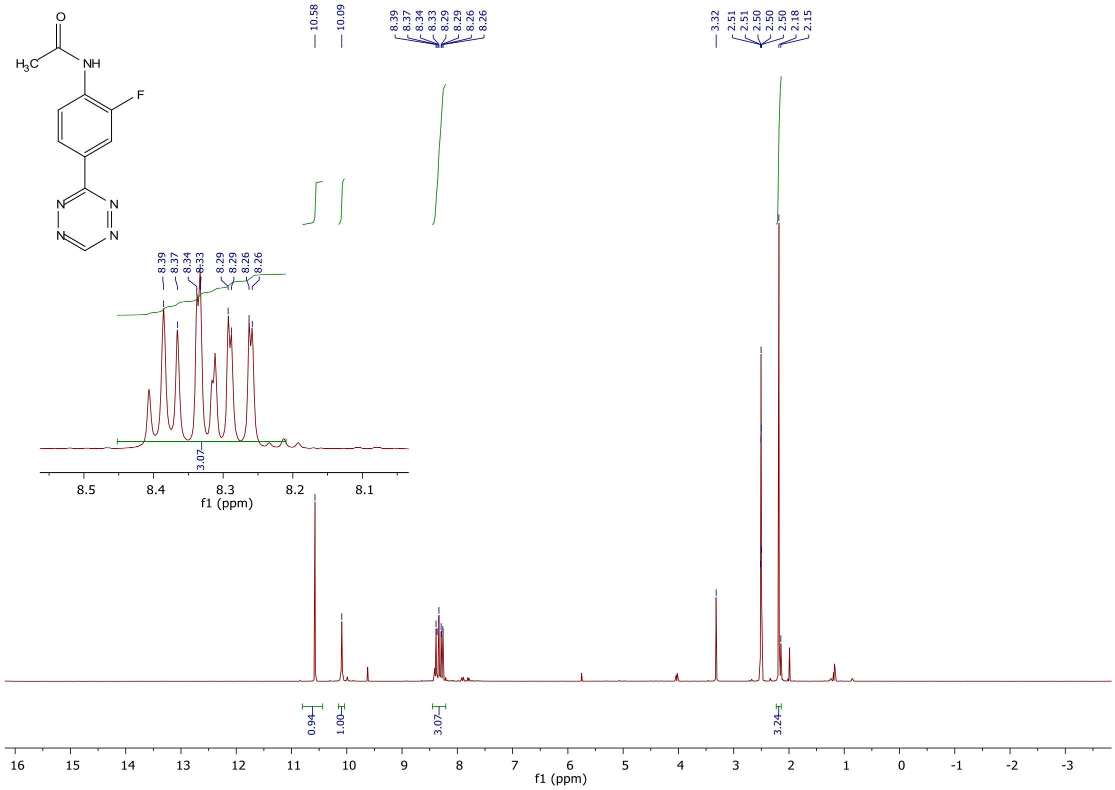

^{13}C NMR spectrum of N-(2-Fluoro-4-(1,2,4,5-tetrazin-3-yl)phenyl)acetamide (**16-*p***) (101 MHz, DMSO-d$_6$)

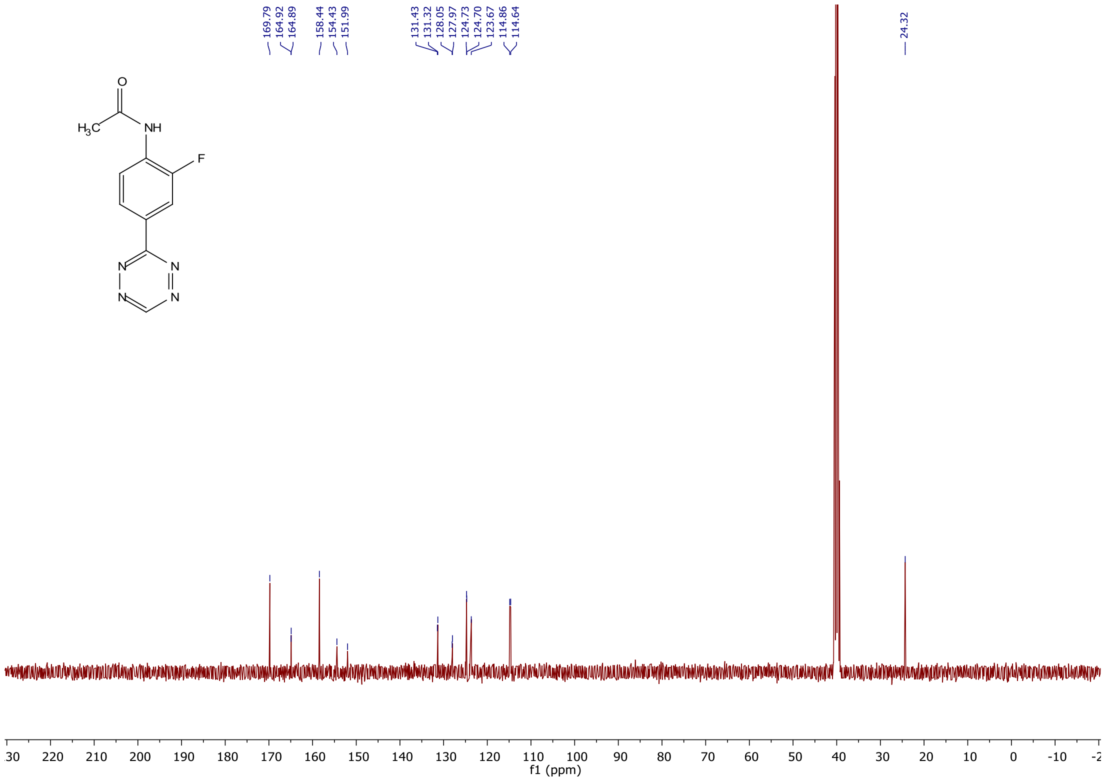

Analytical HPLC of N-(2-Fluoro-4-(1,2,4,5-tetrazin-3-yl)phenyl)acetamide (**16-*p***)

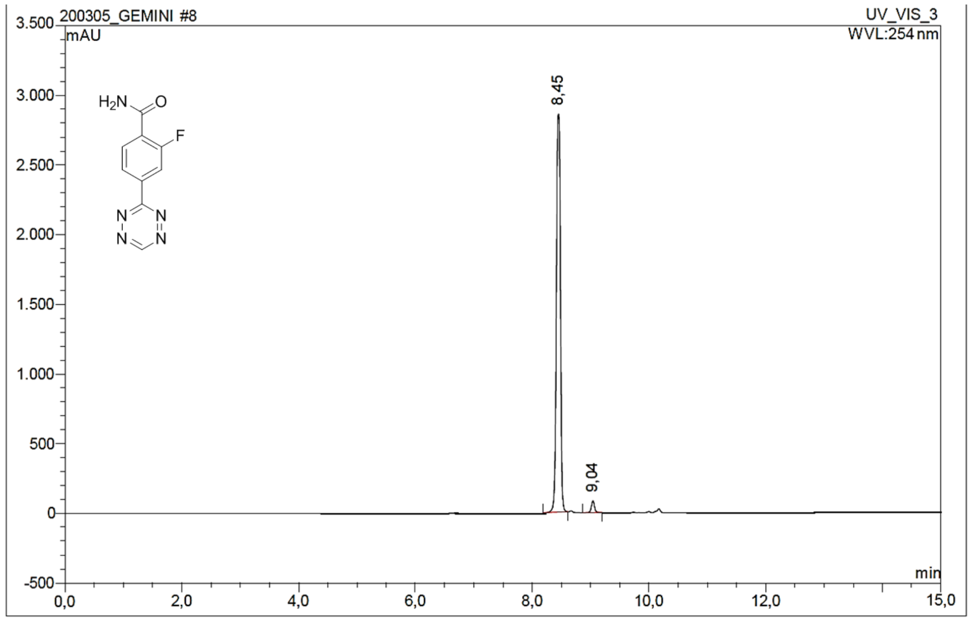

No.	Ret.Time min	Peak Name	Height mAU	Area mAU*min	Rel.Area %	Amount	Resolution(EP)
1	8,45	n.a.	2860,425	232,535	97,71	n.a.	5,21
2	9,04	n.a.	85,917	5,459	2,29	n.a.	n.a.
Total:			2946,342	237,994	100,00	0,000	

^{1}H NMR spectrum of N-(4-cyano-2-iodophenyl)acetamide (**31a**) (600 MHz, $CDCl_3$)

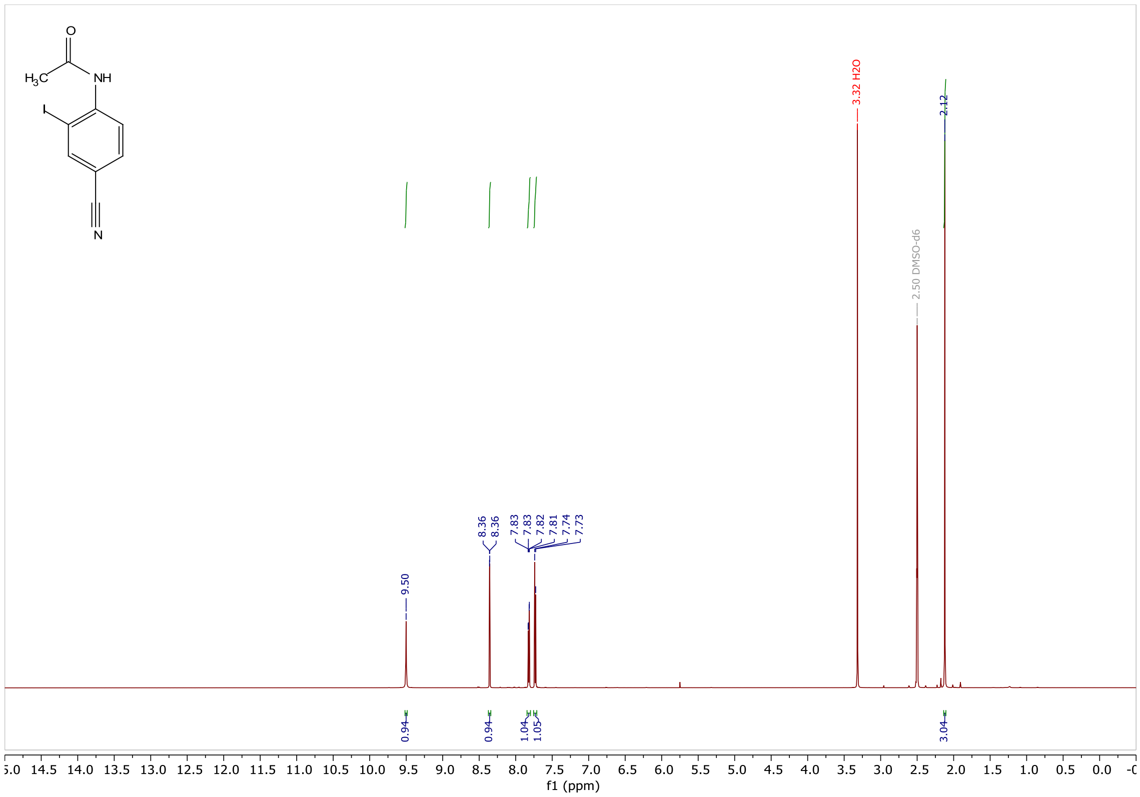

^{13}C NMR spectrum of N-(4-cyano-2-iodophenyl)acetamide (**31a**) (151 MHz, $CDCl_3$)

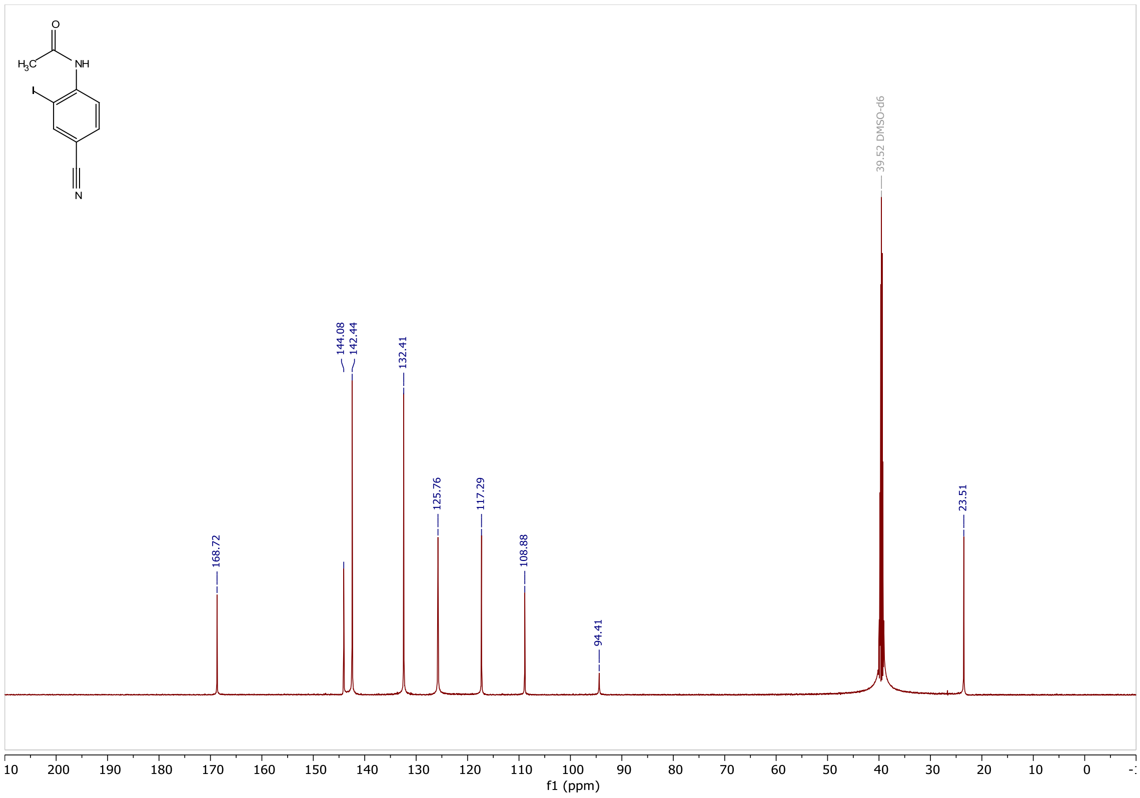

^{1}H NMR spectrum of N-(2-iodo-4-(1,2,4,5-tetrazin-3-yl)phenyl)acetamide (**31b**) (600 MHz, $CDCl_3$)

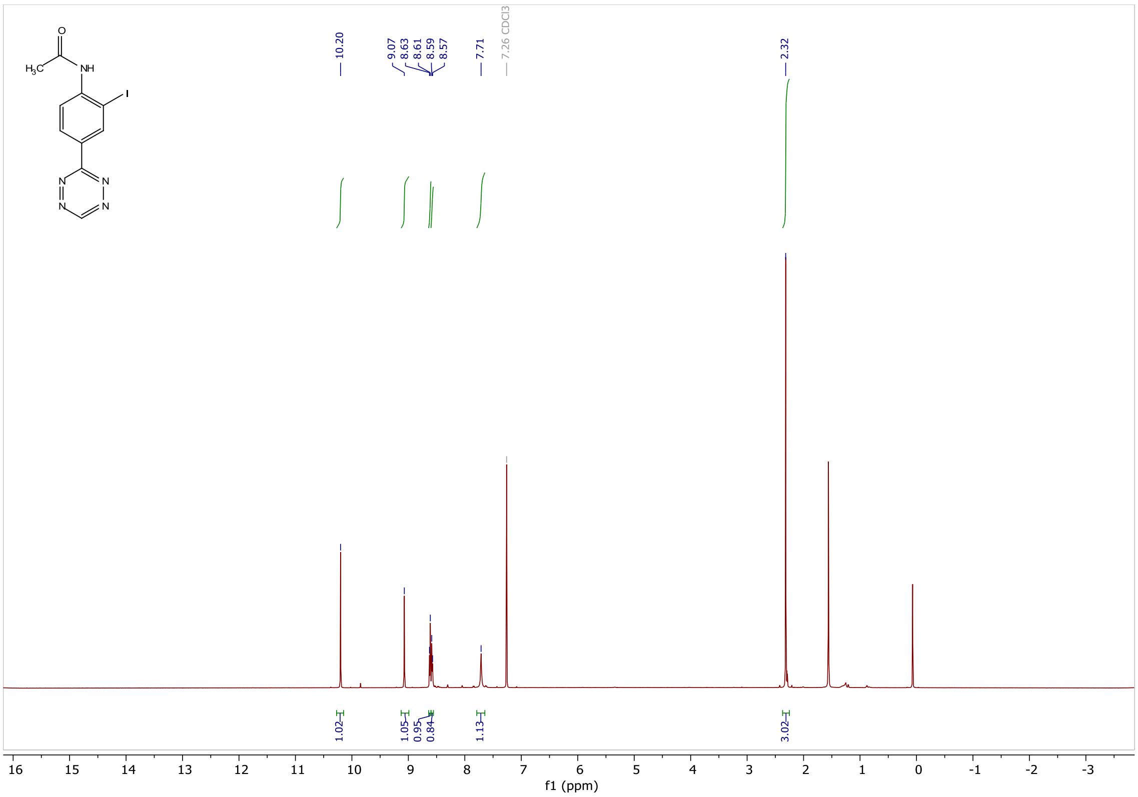

^{13}C NMR spectrum of N-(2-iodo-4-(1,2,4,5-tetrazin-3-yl)phenyl)acetamide (**31b**) (151 MHz, CDCl3)

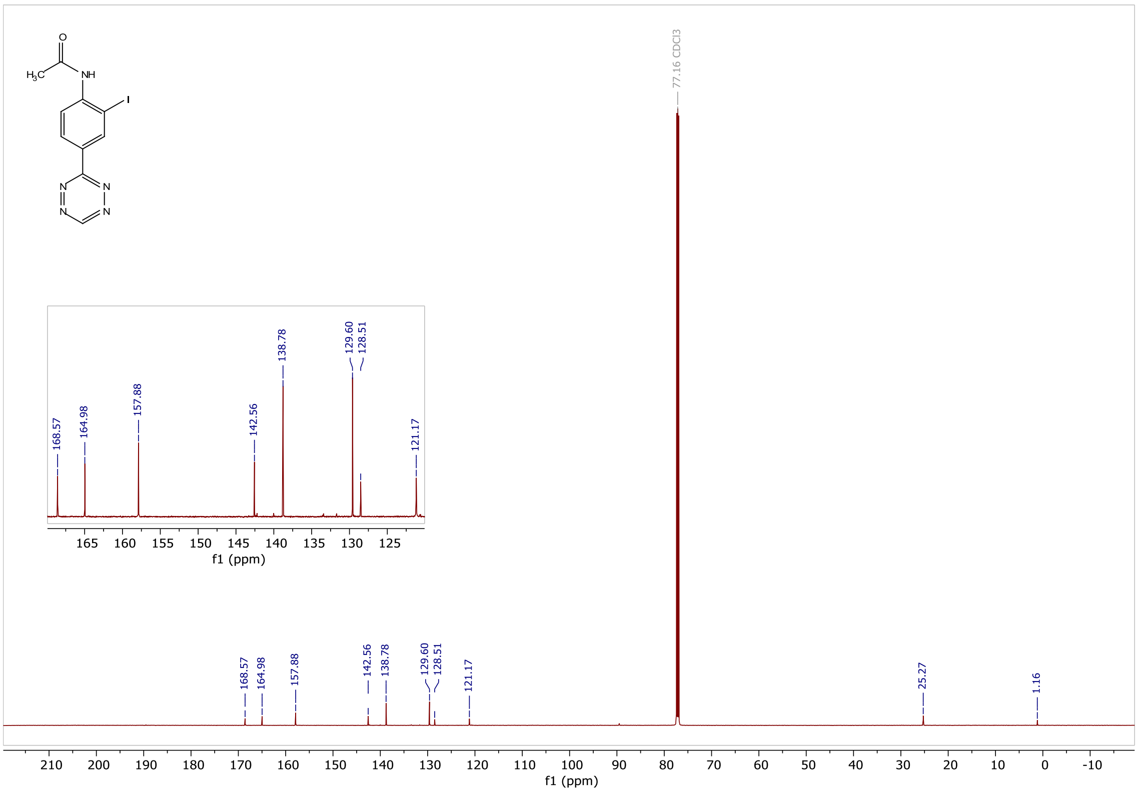

^{1}H NMR spectrum of 4-cyano-2-fluorobenzamide (**17a-*p***) (400 MHz, DMSO-d$_6$)

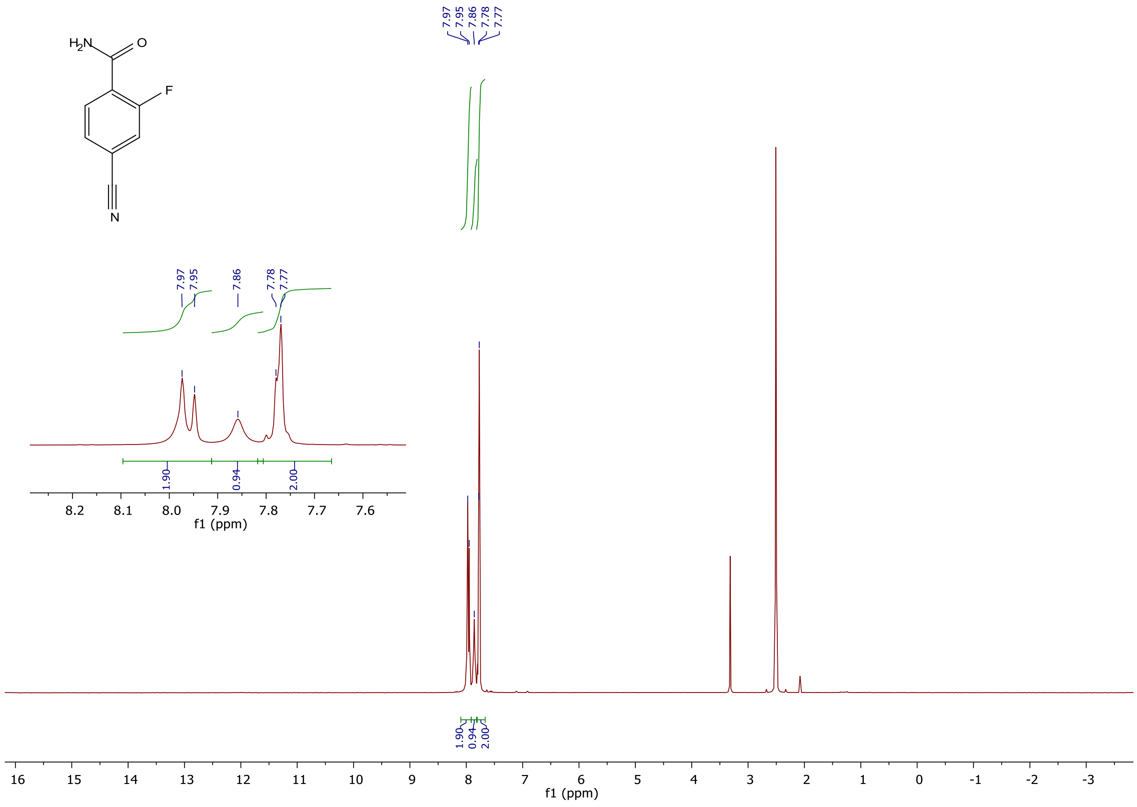

^{13}C NMR spectrum of 4-cyano-2-fluorobenzamide (**17a-*p***) (101 MHz, DMSO-d$_6$)

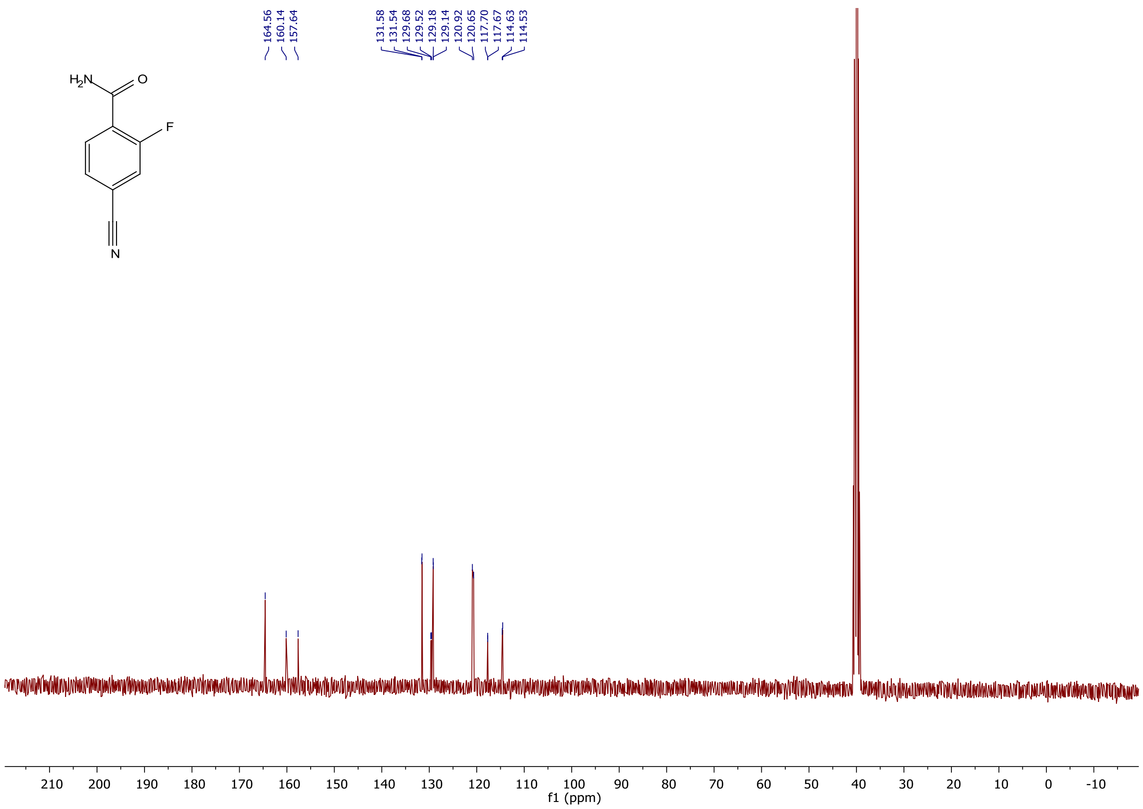

^{1}H NMR spectrum of 2-fluoro-4-(1,2,4,5-tetrazin-3-yl)benzamide (**17-*p***) (400 MHz, DMSO-d$_6$)

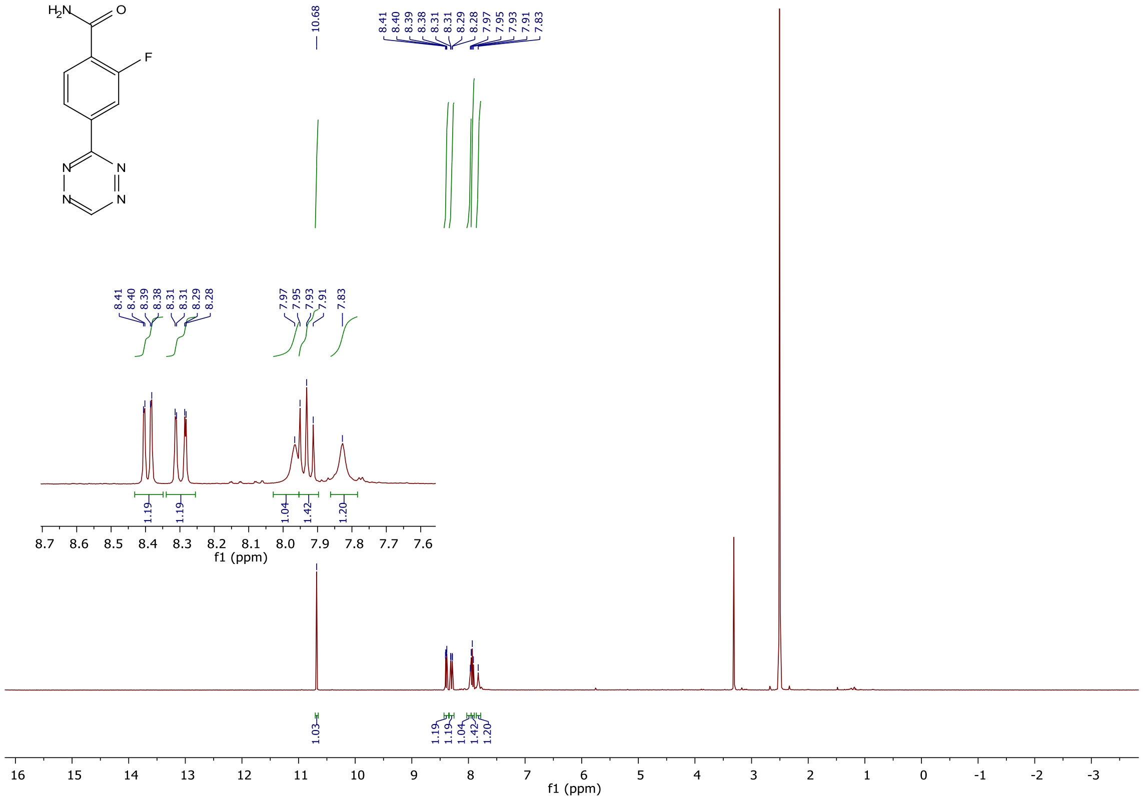

^{13}C NMR spectrum of 2-fluoro-4-(1,2,4,5-tetrazin-3-yl)benzamide (**17-*p***) (101 MHz, DMSO-d$_6$)

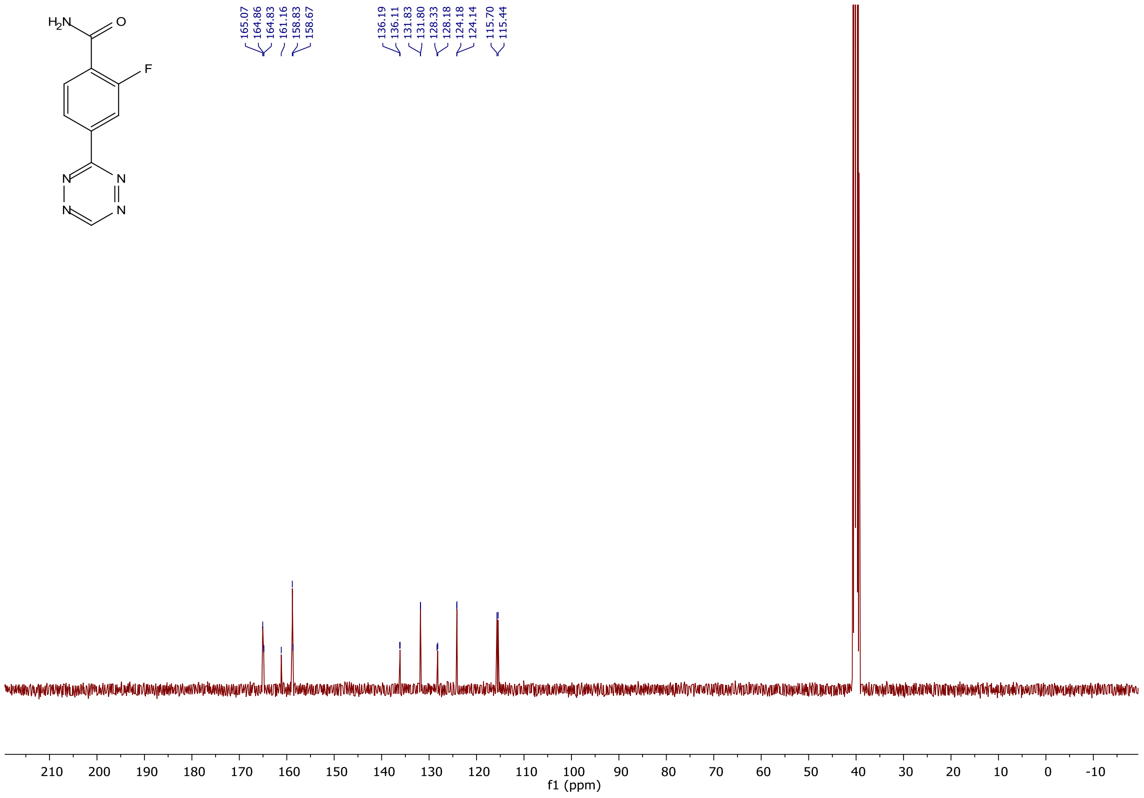

Analytical HPLC of 2-fluoro-4-(1,2,4,5-tetrazin-3-yl)benzamide (**17-*p***)

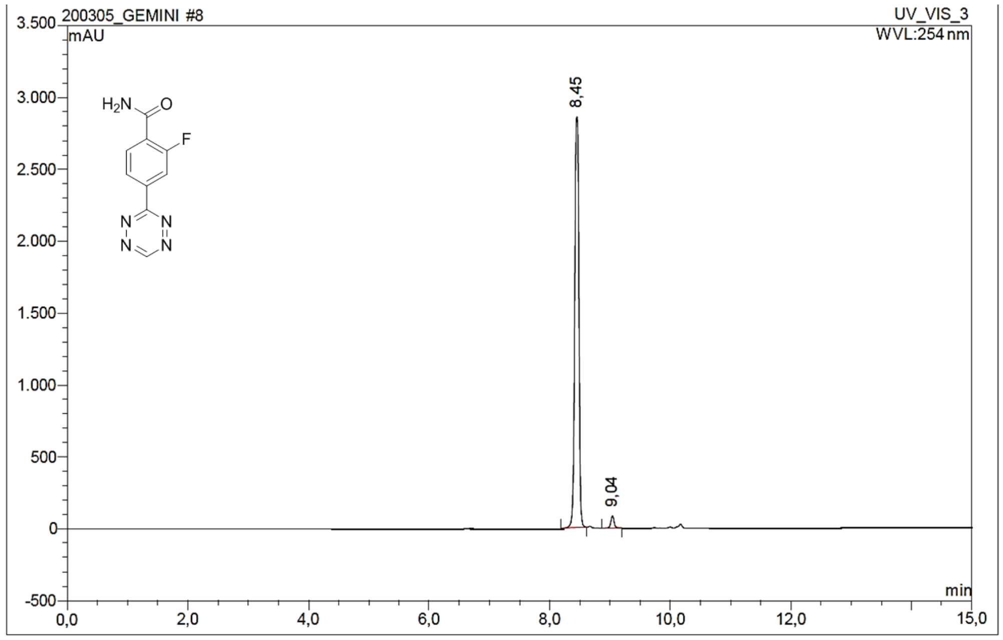

No.	Ret.Time min	Peak Name	Height mAU	Area mAU*min	Rel.Area %	Amount	Resolution(EP)
1	8,45	n.a.	2860,425	232,535	97,71	n.a.	5,21
2	9,04	n.a.	85,917	5,459	2,29	n.a.	n.a.
**Total:**			2946,342	237,994	100,00	0,000	

^{1}H NMR spectrum of 4-cyano-2-iodobenzamide (**32a**) (400 MHz, DMSO-d_6)

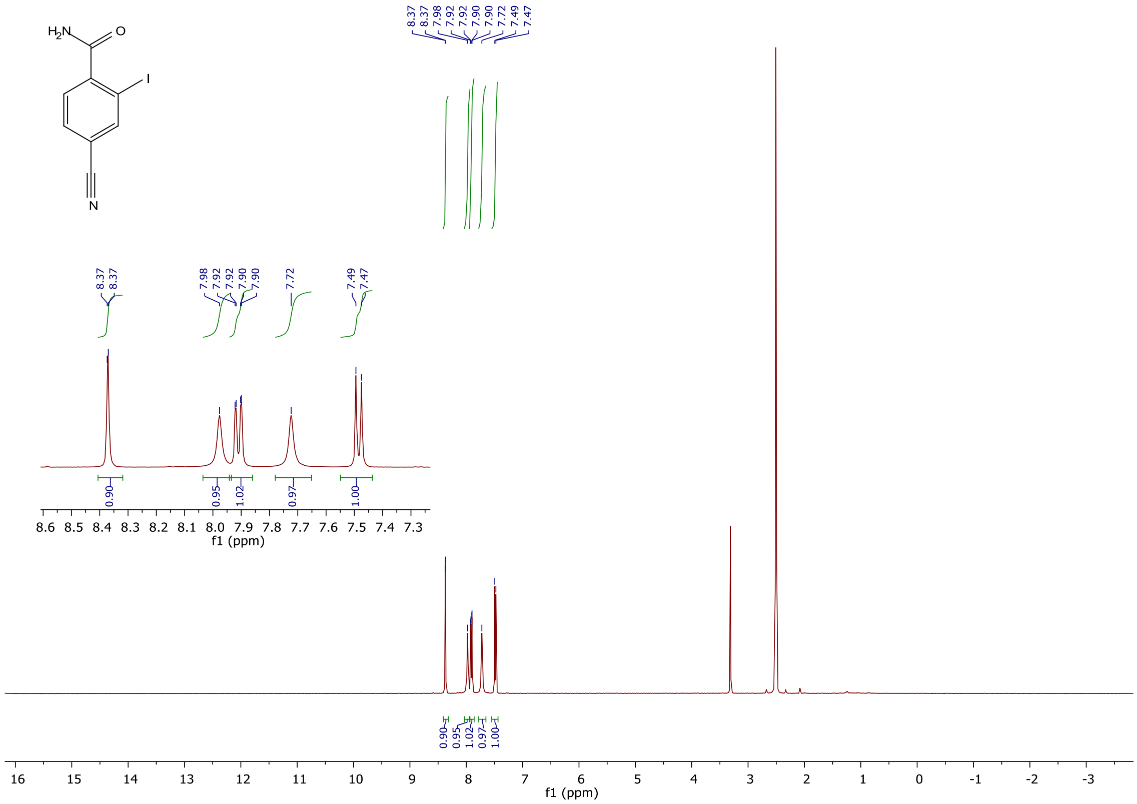

^{13}C NMR spectrum of 4-cyano-2-iodobenzamide (**32a**) (101 MHz, DMSO-d$_6$)

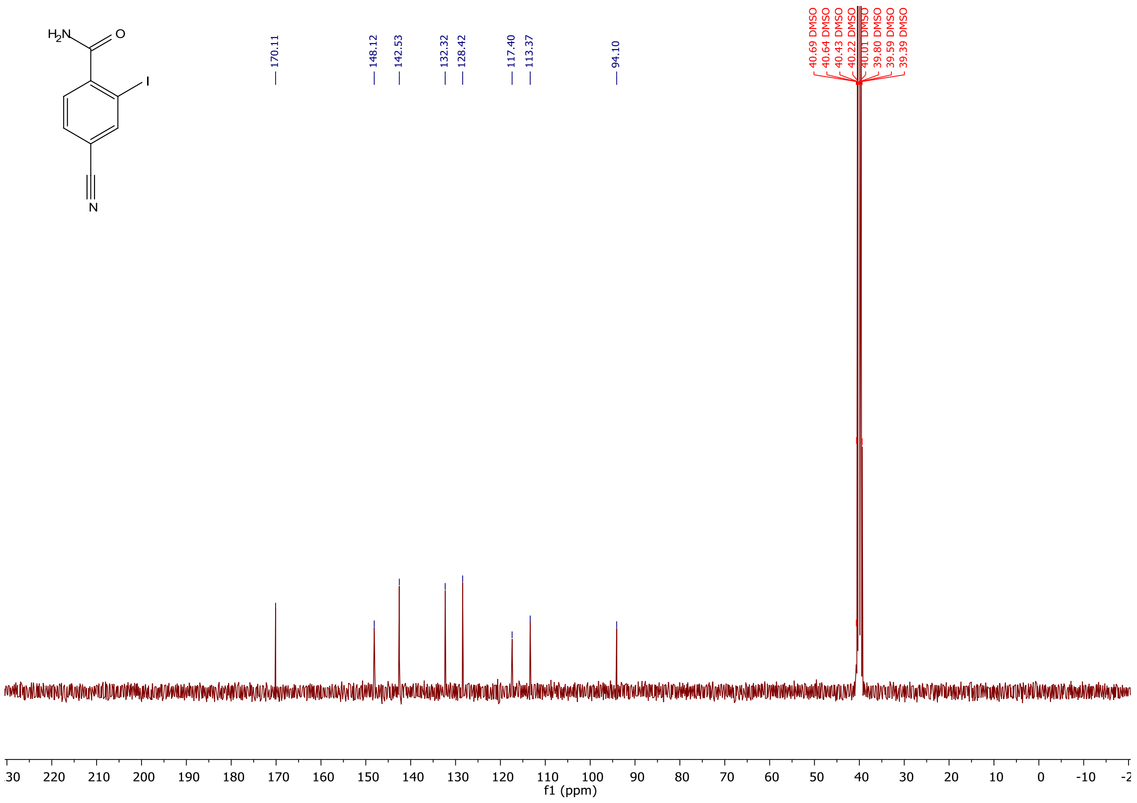

^{1}H NMR spectrum of 2-iodo-4-(1,2,4,5-tetrazin-3-yl)benzamide (**32b**) (400 MHz, DMSO-d_6)

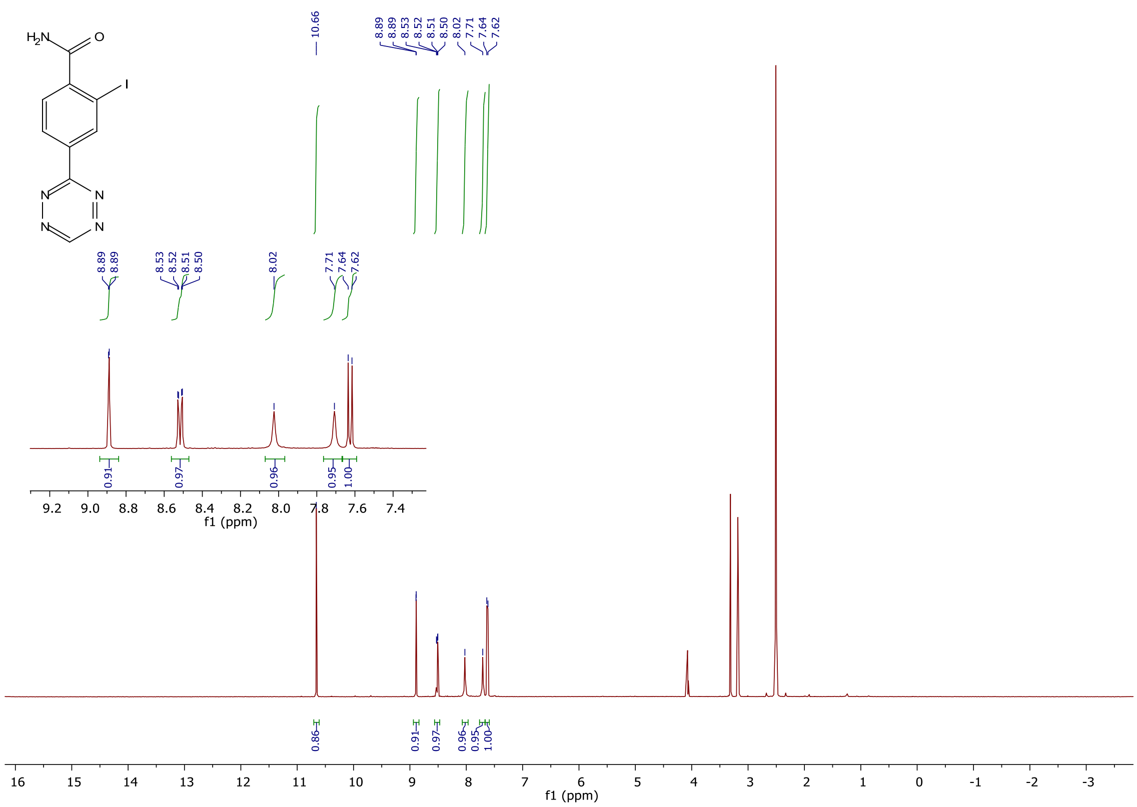

^{13}C NMR spectrum of 2-iodo-4-(1,2,4,5-tetrazin-3-yl)benzamide (**32b**) (101 MHz, DMSO-d_6)

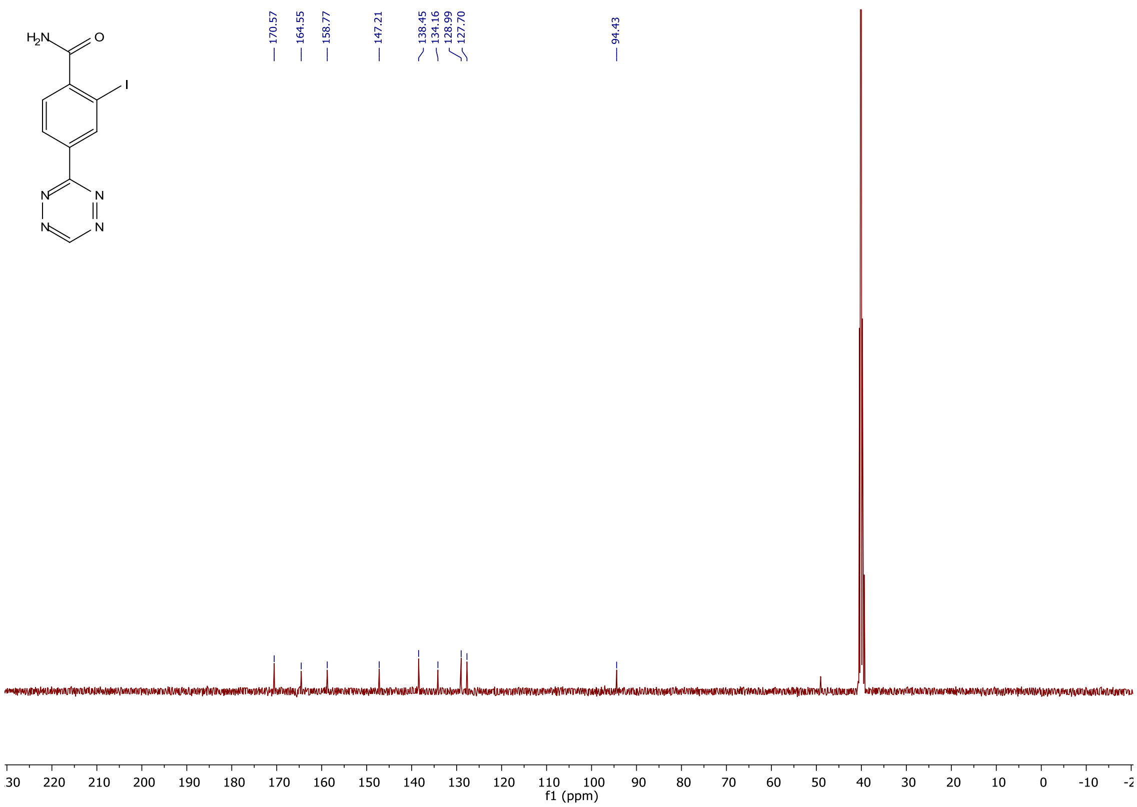

^{1}H NMR spectrum of 4-cyano-2-fluoro-N-methylbenzamide (**18a-*p***) (400 MHz, DMSO-d_6)

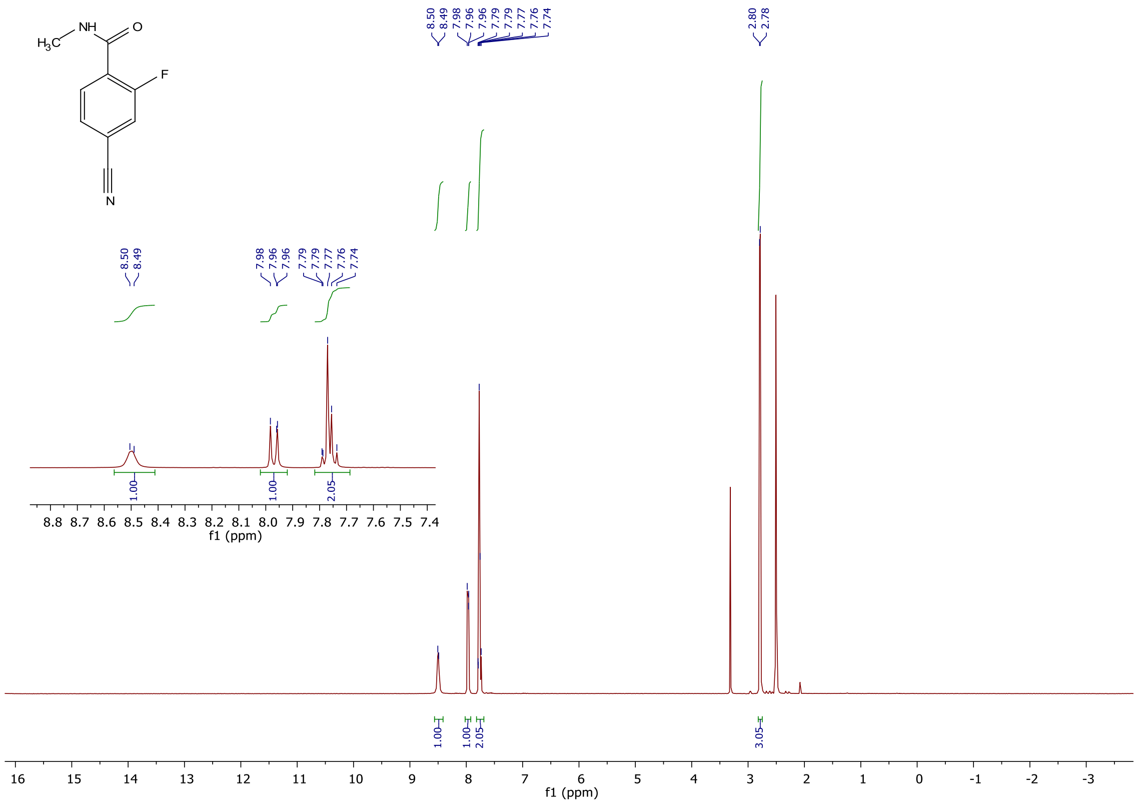

^{13}C NMR spectrum of 4-cyano-2-fluoro-N-methylbenzamide (**18a-*p***) (101 MHz, DMSO-d_6)

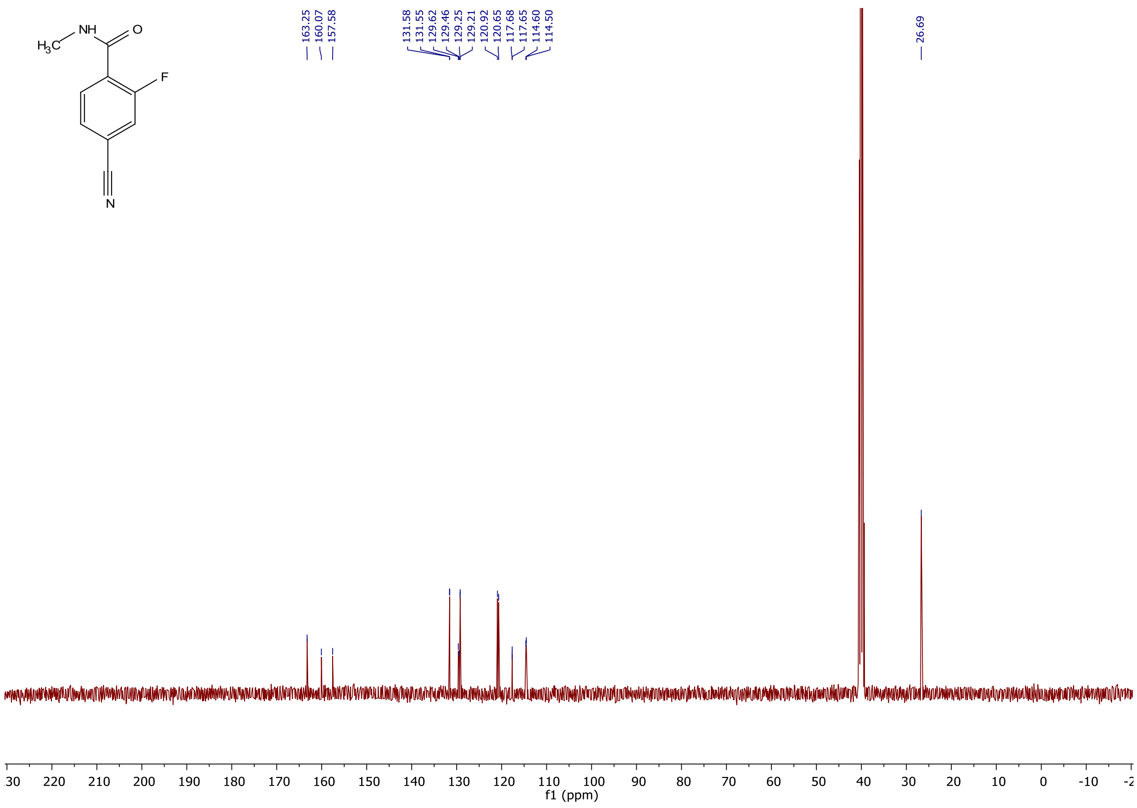

^{1}H NMR spectrum of 2-fluoro-N-methyl-4-(1,2,4,5-tetrazin-3-yl)benzamide (**18-*p***) (400 MHz, DMSO-d_6)

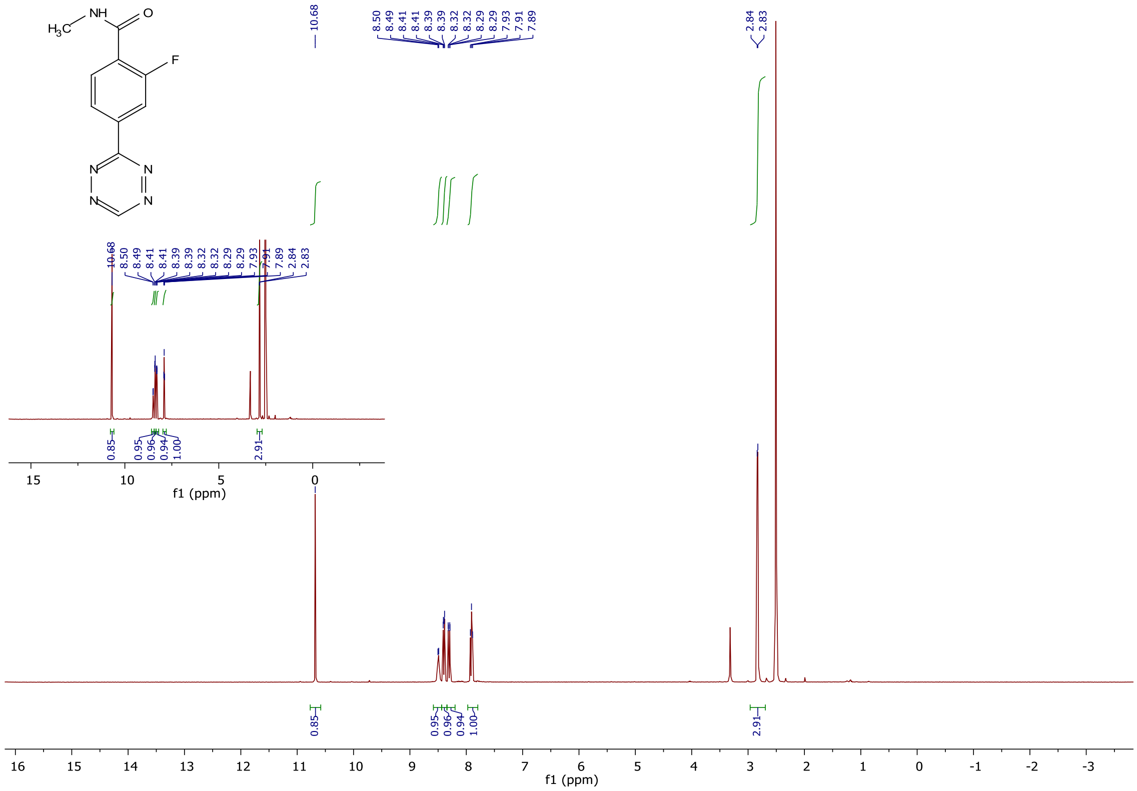

^{13}C NMR spectrum of 2-fluoro-N-methyl-4-(1,2,4,5-tetrazin-3-yl)benzamide (**18-*p***) (101 MHz, DMSO-d$_6$)

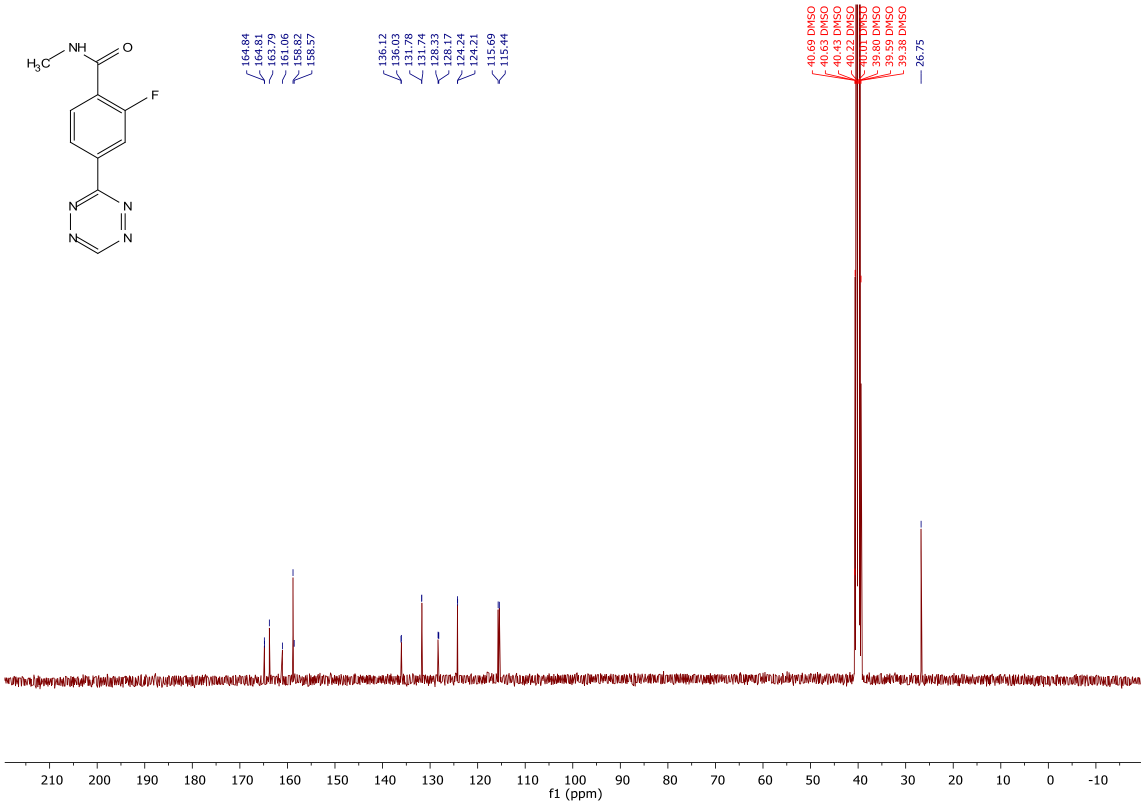

Analytical HPLC of 2-fluoro-N-methyl-4-(1,2,4,5-tetrazin-3-yl)benzamide (**18-*p***)

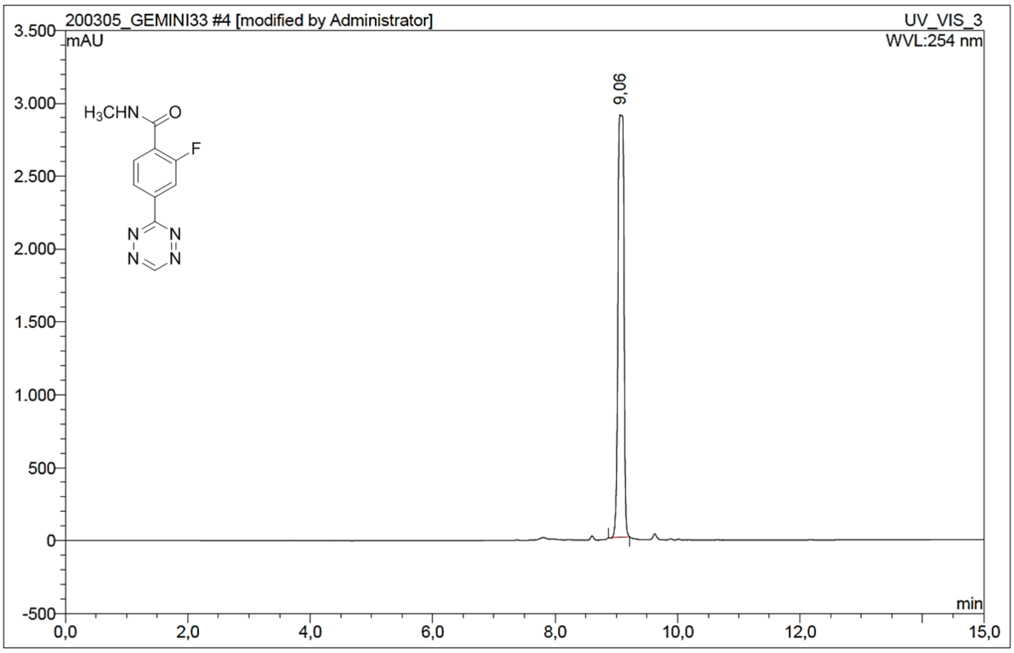

No.	Ret.Time min	Peak Name	Height mAU	Area mAU*min	Rel.Area %	Amount	Resolution(EP)
1	9,06	n.a.	2899,156	318,914	100,00	n.a.	n.a.
Total:			2899,156	318,914	100,00	0,000	

^{1}H NMR spectrum of 4-cyano-2-iodo-N-methylbenzamide (**33a**) (400 MHz, DMSO-d$_6$)

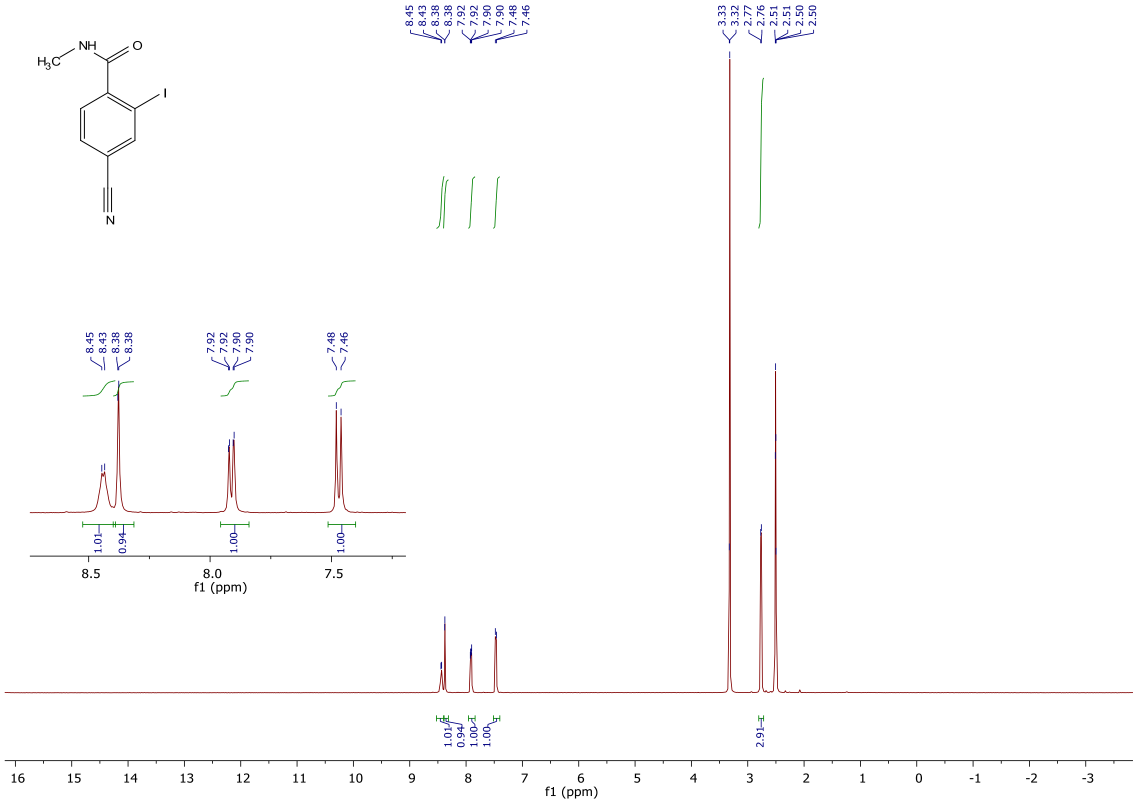

^{13}C NMR spectrum of 4-cyano-2-iodo-N-methylbenzamide (**33a**) (101 MHz, DMSO-d$_6$)

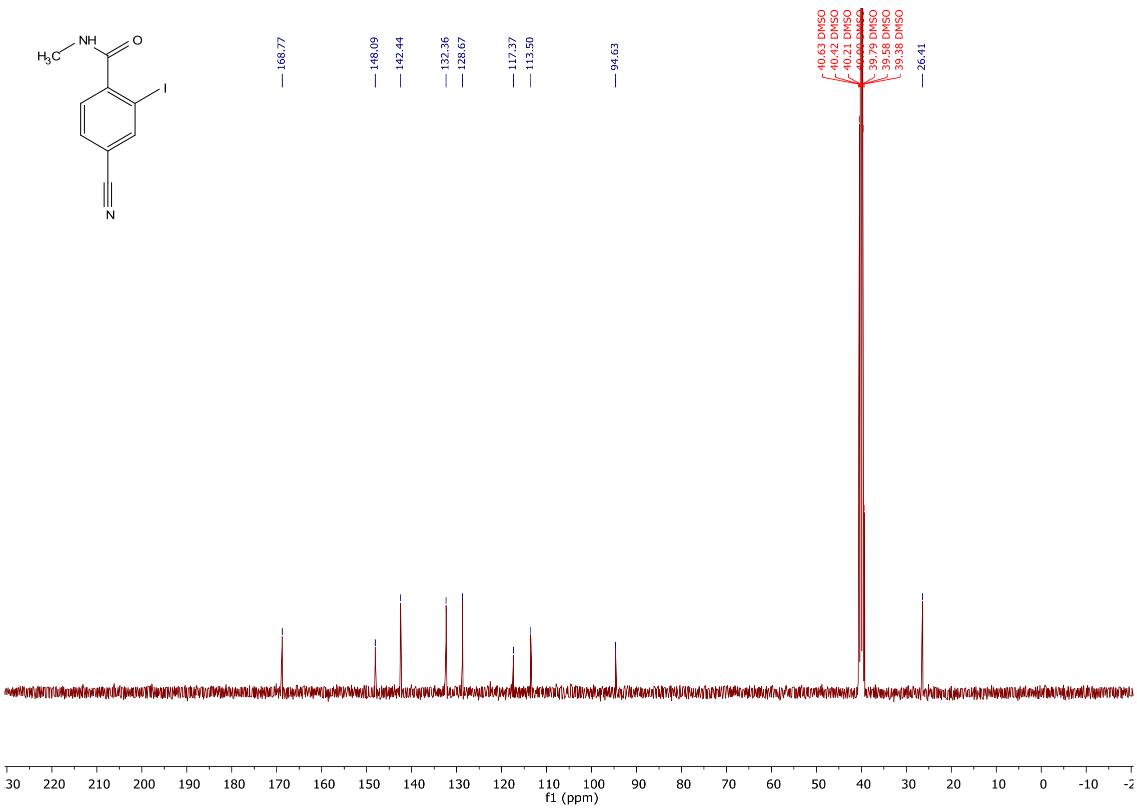

^{1}H NMR spectrum of 2-iodo-N-methyl-4-(1,2,4,5-tetrazin-3-yl)benzamide (**33b**) (400 MHz, DMSO-d$_6$)

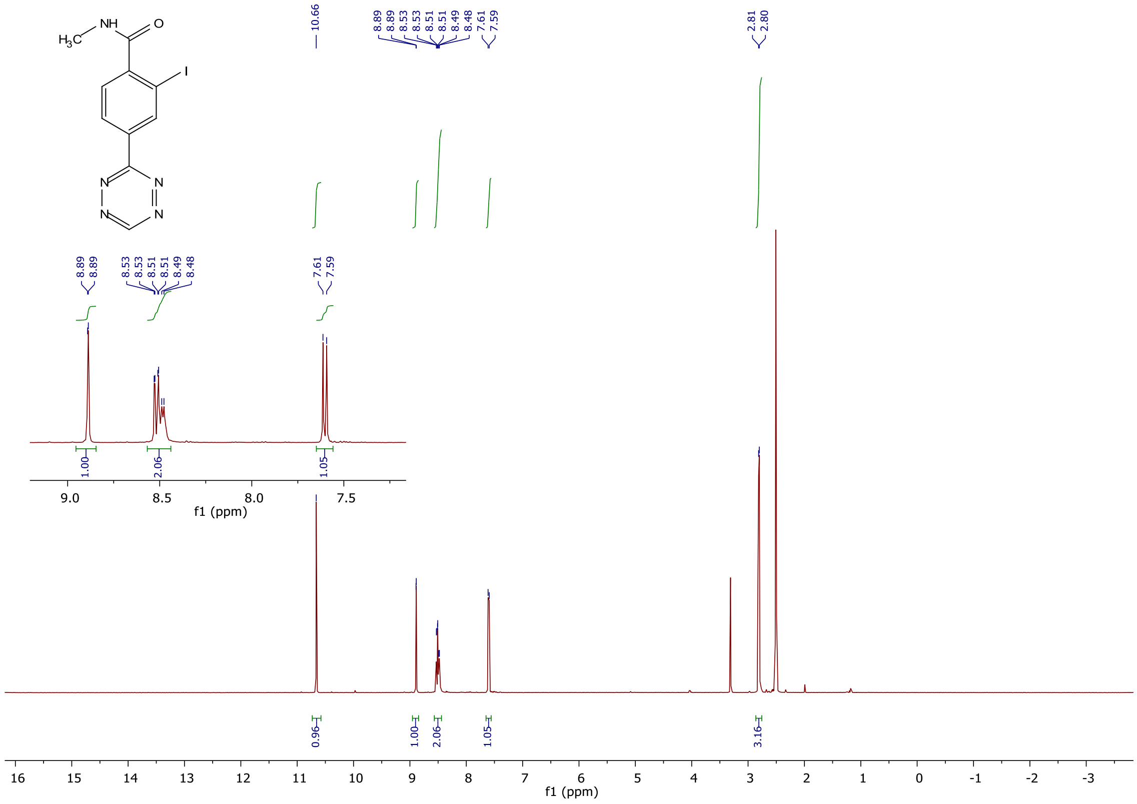

^{13}C NMR spectrum of 2-iodo-N-methyl-4-(1,2,4,5-tetrazin-3-yl)benzamide (**33b**) (101 MHz, DMSO-d_6)

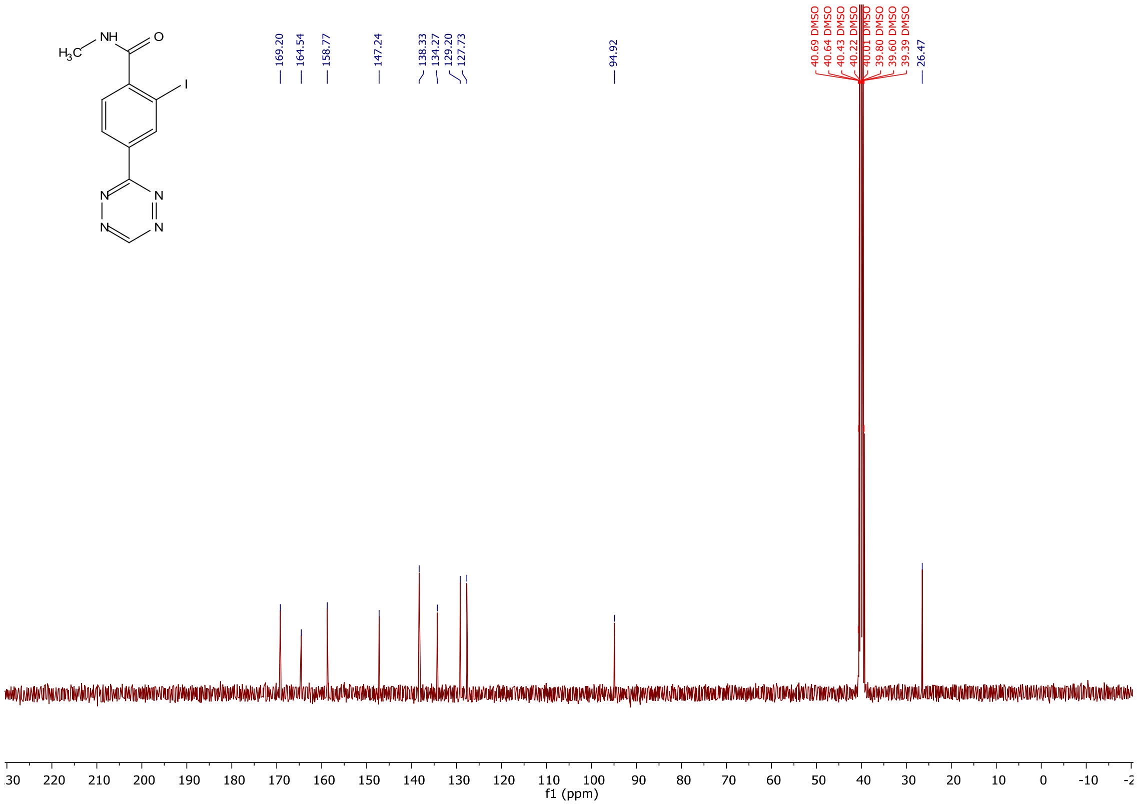

^{1}H NMR spectrum of 3-(3-fluoro-5-methylphenyl)-1,2,4,5-tetrazine (**14-*m***) (400 MHz, $CDCl_3$)

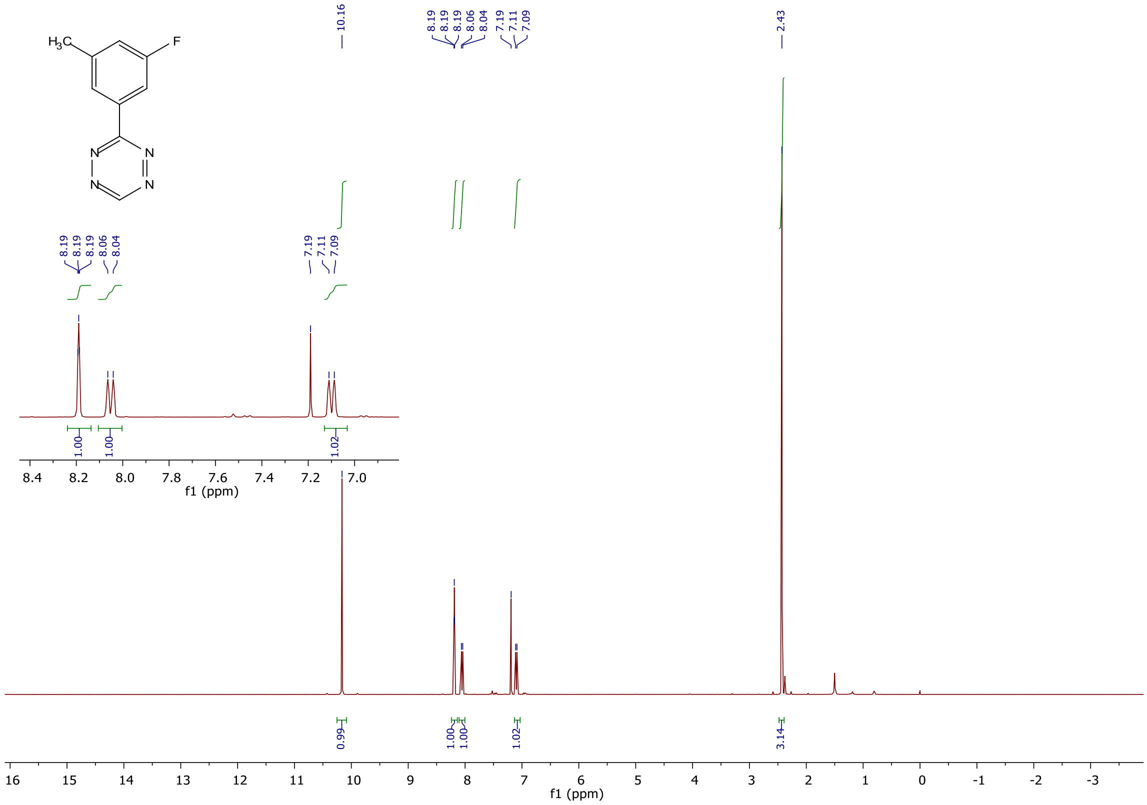

^{13}C NMR spectrum of 3-(3-fluoro-5-methylphenyl)-1,2,4,5-tetrazine (**14-*m***) (101 MHz, CDCl3)

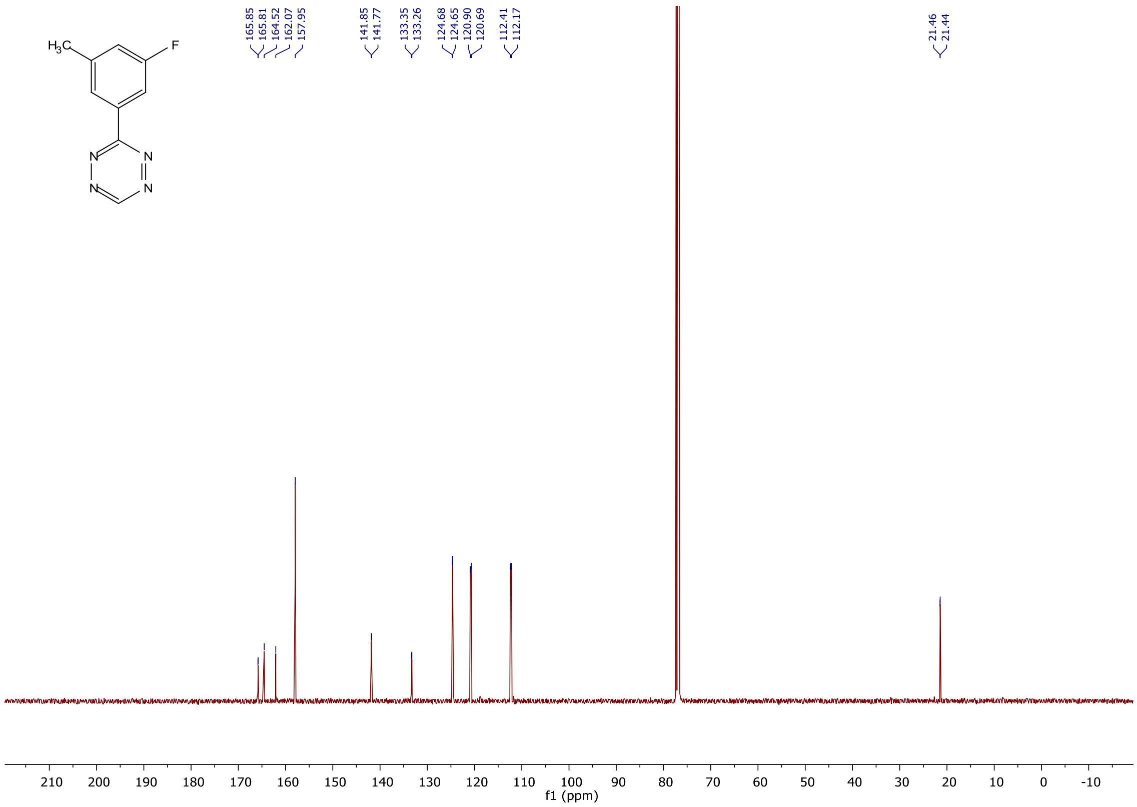

Analytical HPLC of 3-(3-fluoro-5-methylphenyl)-1,2,4,5-tetrazine (**14-*m***)

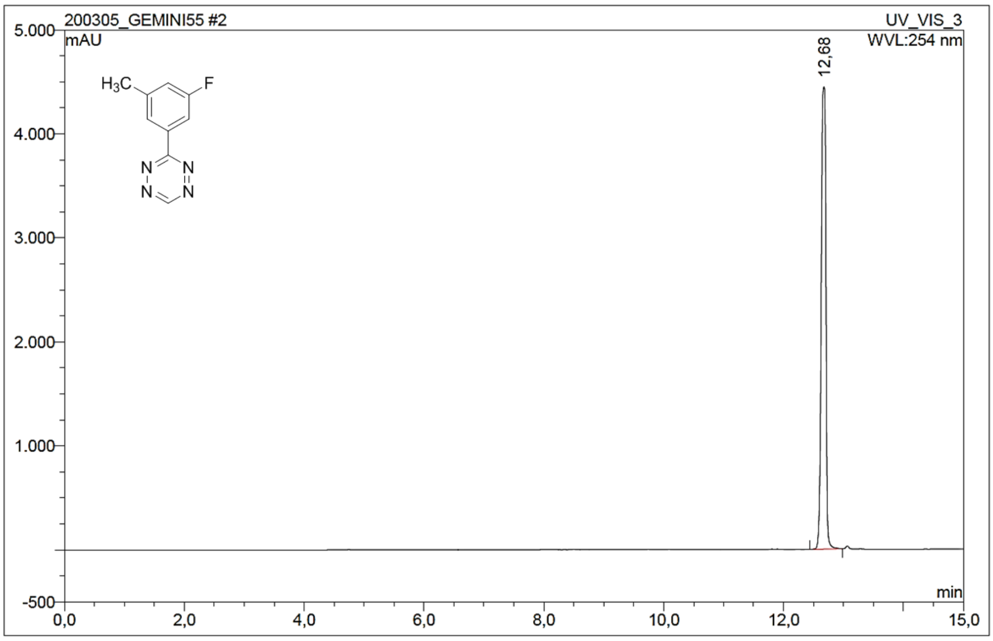

No.	Ret.Time min	Peak Name	Height mAU	Area mAU*min	Rel.Area %	Amount	Resolution(EP)
1	12,68	n.a.	4447,213	389,900	100,00	n.a.	n.a.
Total:			4447,213	389,900	100,00	0,000	

^{1}H NMR spectrum of 3-(3-iodo-5-methylphenyl)-1,2,4,5-tetrazine (**34a**) (400 MHz, $CDCl_3$)

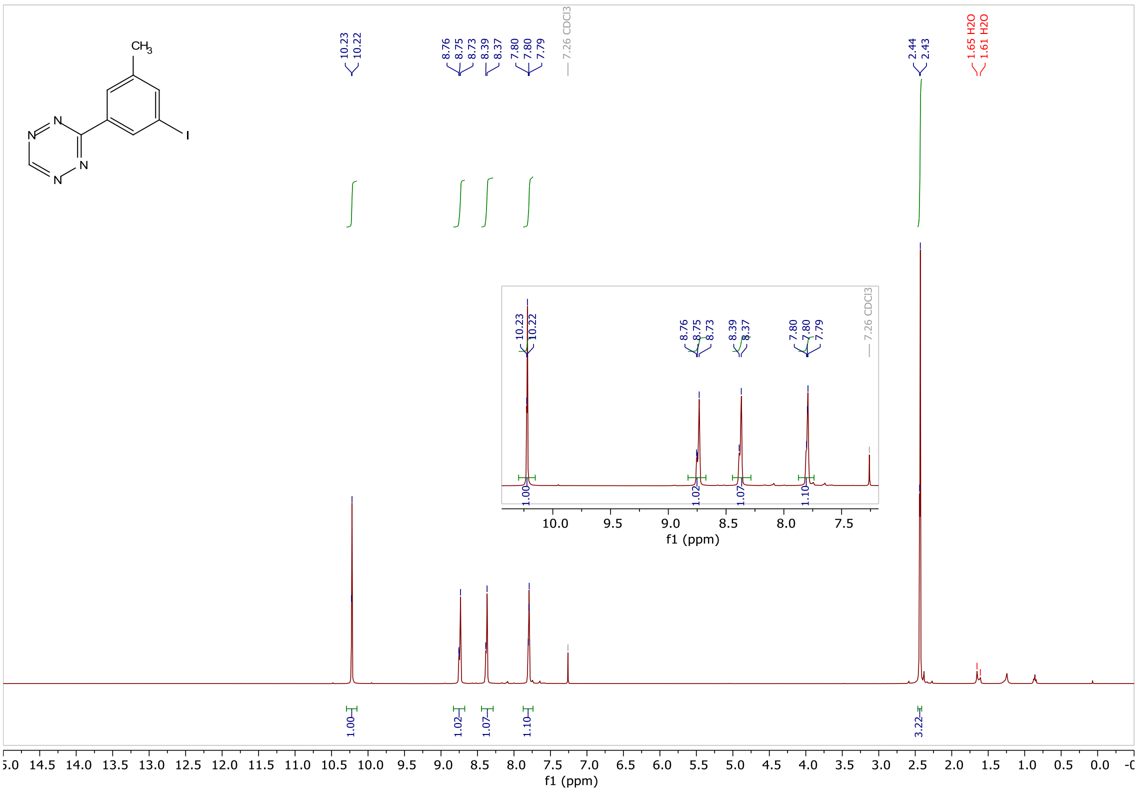

^{13}C NMR spectrum of 3-(3-iodo-5-methylphenyl)-1,2,4,5-tetrazine (**34a**) (101 MHz, CDCl3)

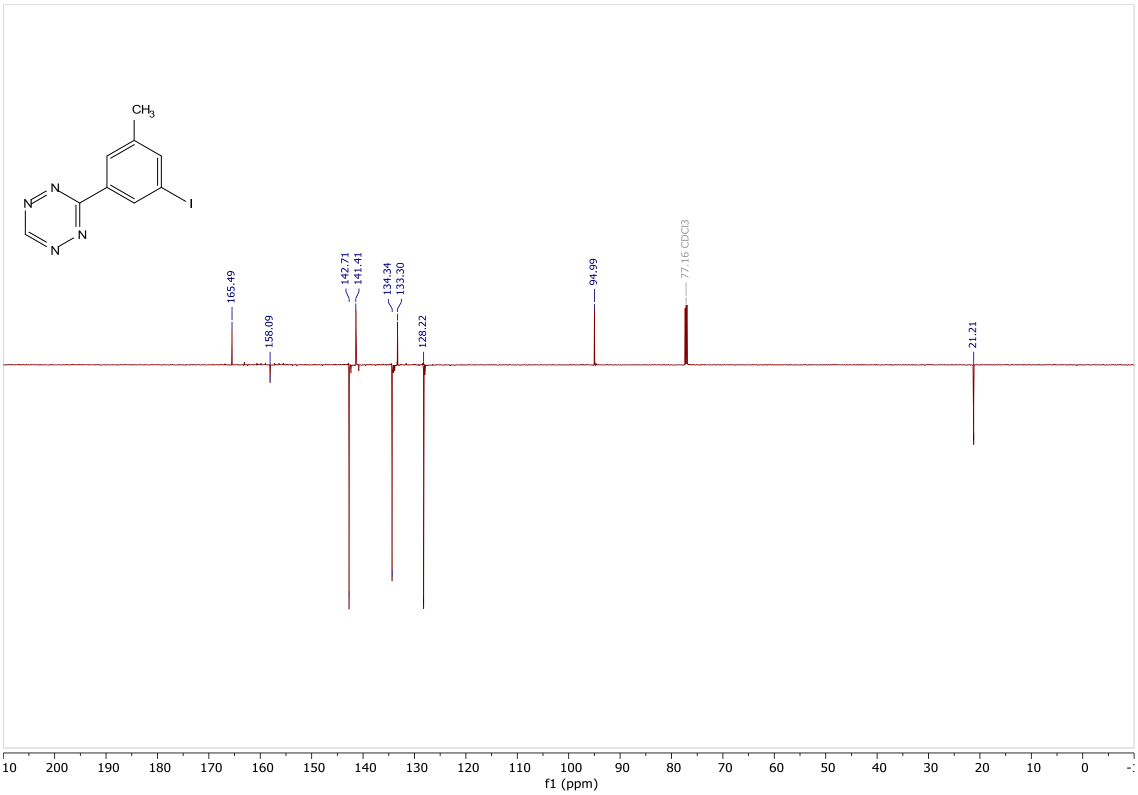

^{1}H NMR spectrum of 3-(3-fluoro-5-methoxyphenyl)-1,2,4,5-tetrazine (**15-*m***) (400 MHz, CDCl3)

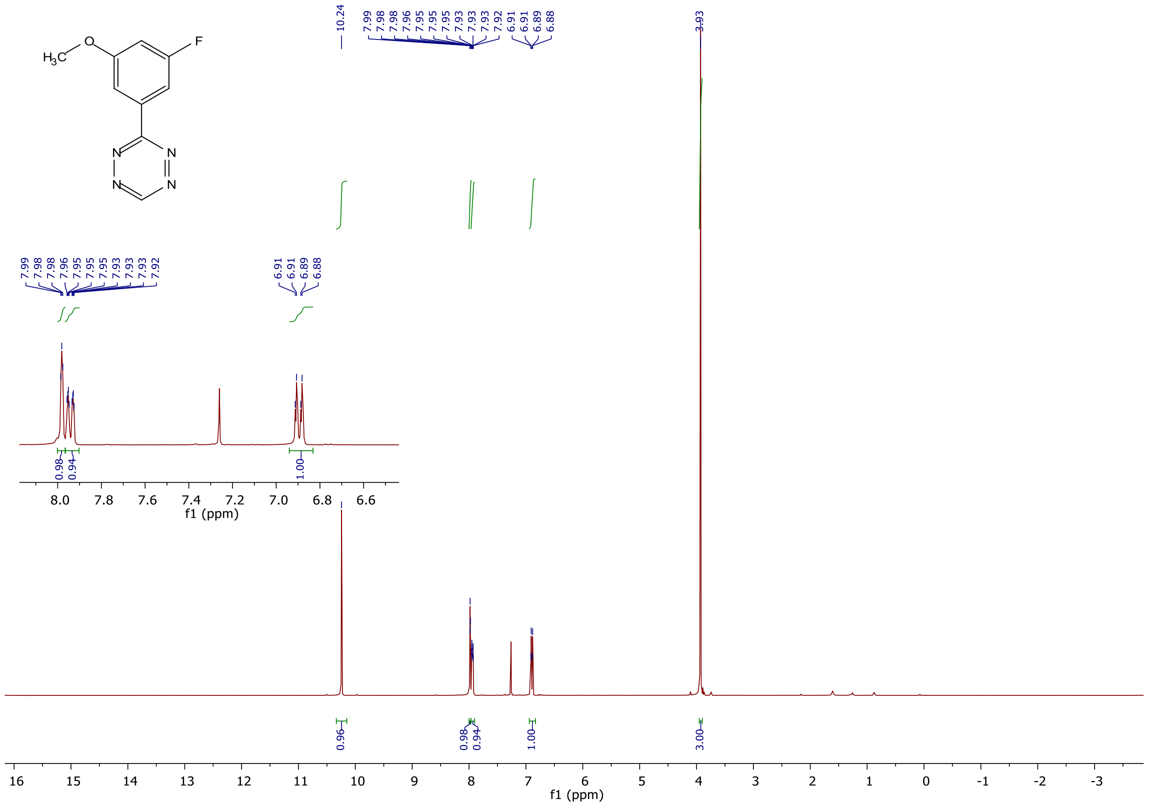

^{13}C NMR spectrum of 3-(3-fluoro-5-methoxyphenyl)-1,2,4,5-tetrazine (**15-*m***) (101 MHz, CDCl3)

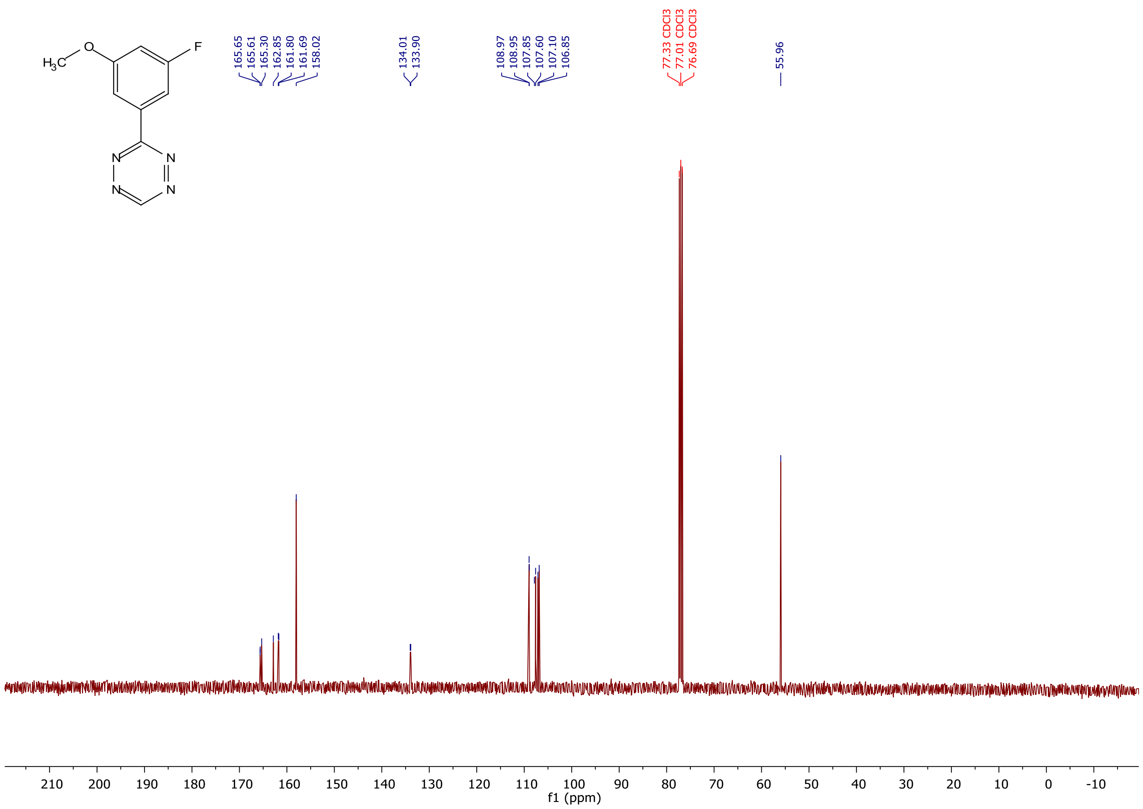

Analytical HPLC of 3-(3-fluoro-5-methoxyphenyl)-1,2,4,5-tetrazine (**15-*m***)

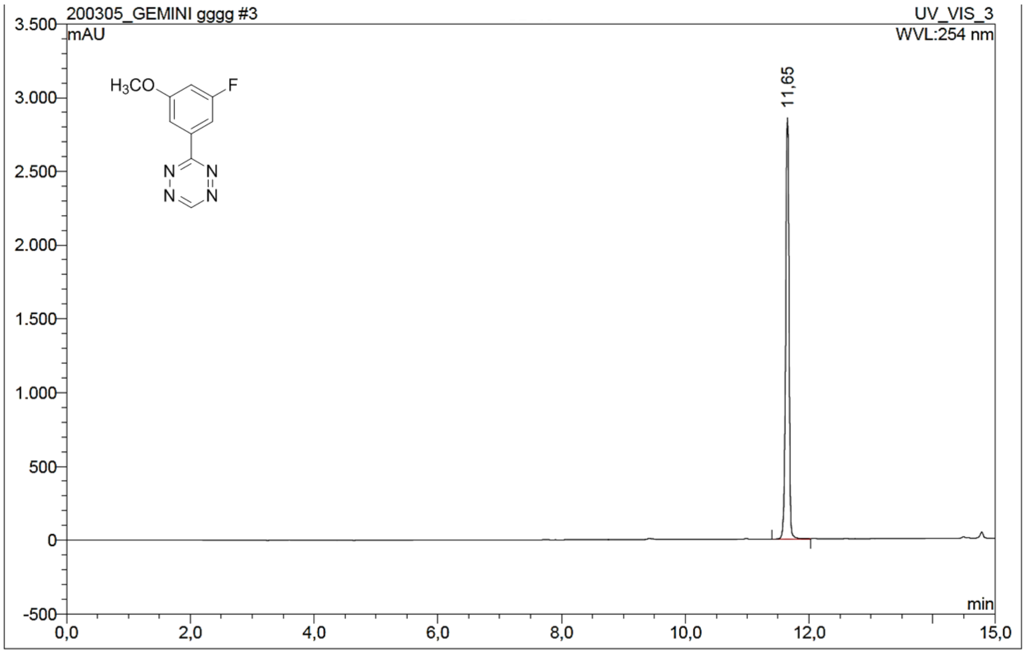

No.	Ret.Time min	Peak Name	Height mAU	Area mAU*min	Rel.Area %	Amount	Resolution(EP)
1	11,65	n.a.	2855,952	185,883	100,00	n.a.	n.a.
**Total:**			2855,952	185,883	100,00	0,000	

^{1}H NMR spectrum of 3-iodo-5-methoxybenzonitrile (**35a**) (400 MHz, CDCl3)

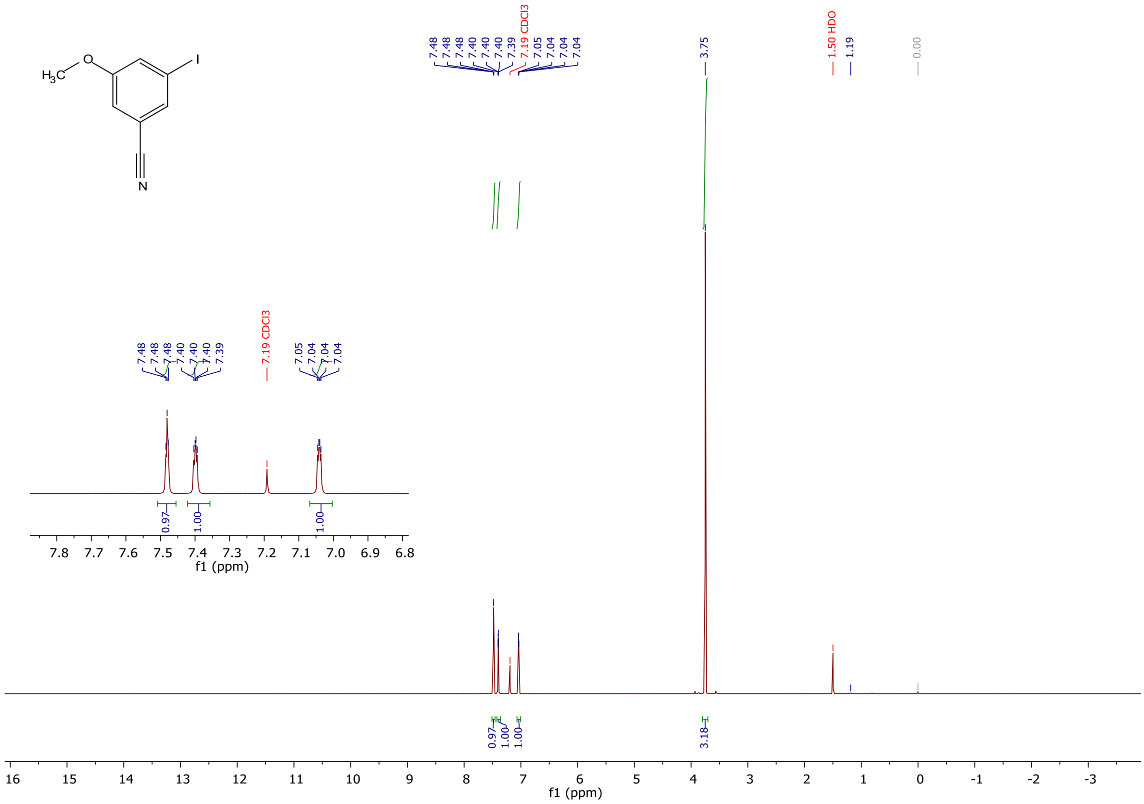

^{13}C NMR spectrum of 3-iodo-5-methoxybenzonitrile (**35a**) (101 MHz, CDCl3)

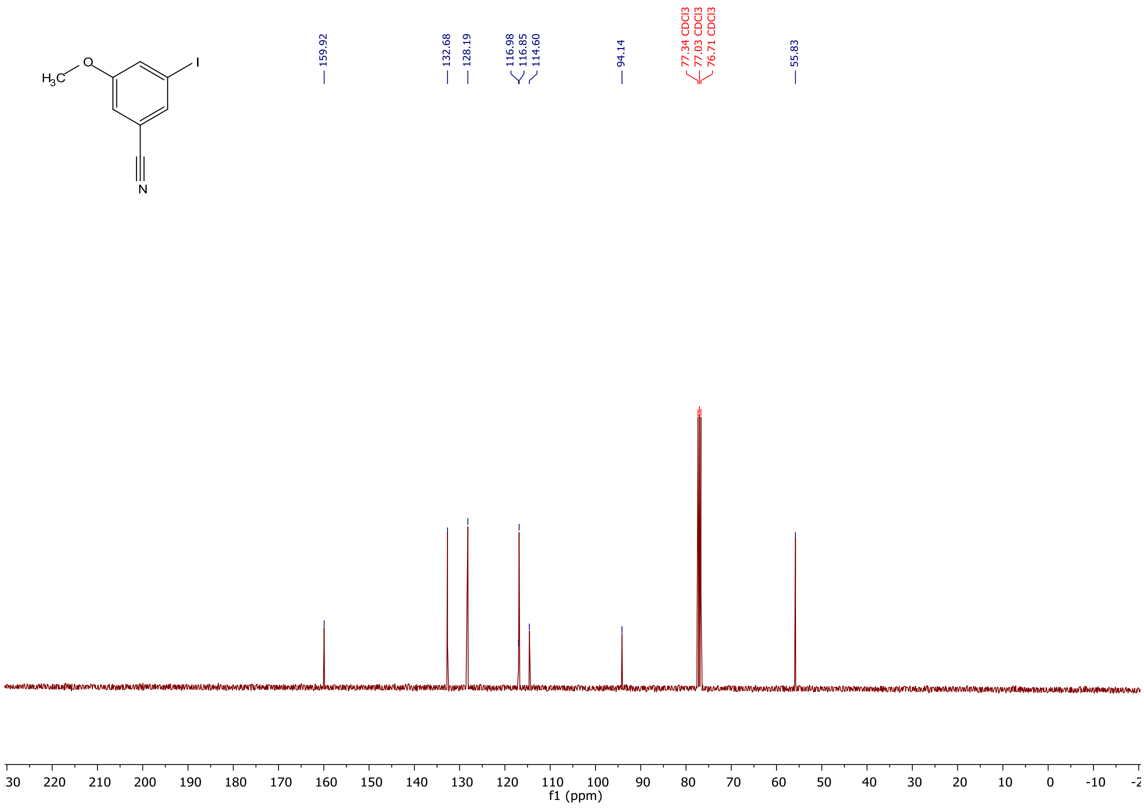

^{1}H NMR spectrum of 3-(3-iodo-5-methoxyphenyl)-1,2,4,5-tetrazine (**35b**) (400 MHz, CDCl3)

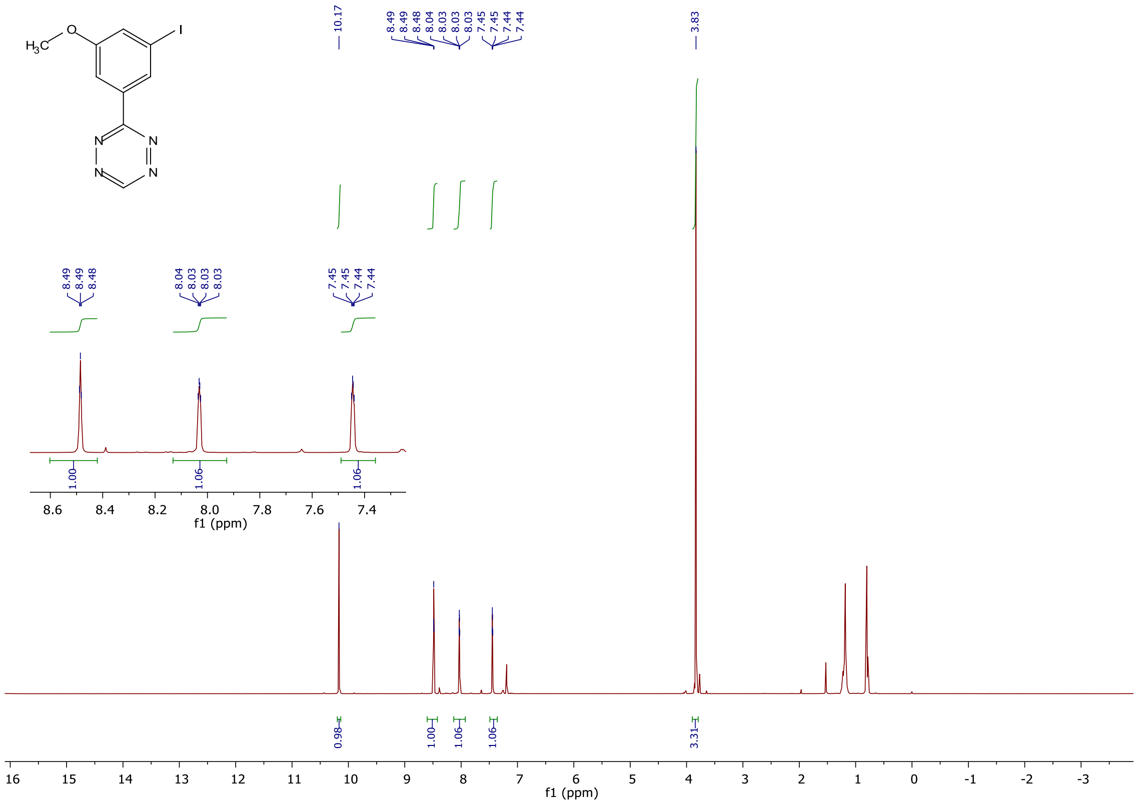

^{13}C NMR spectrum of 3-(3-iodo-5-methoxyphenyl)-1,2,4,5-tetrazine (**35b**) (101 MHz, CDCl3)

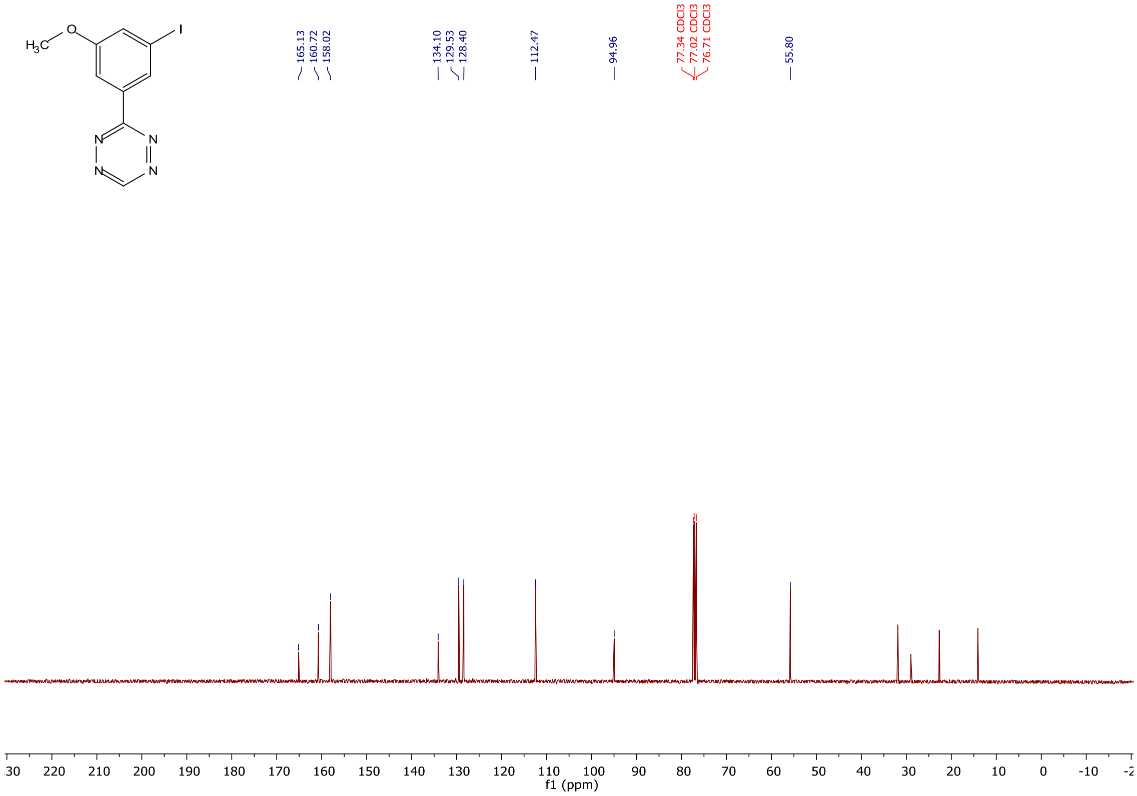

^{1}H NMR spectrum of N-(5-cyano-3-fluorophenyl)acetamide (**16a-*m***) (400 MHz, DMSO-d6)

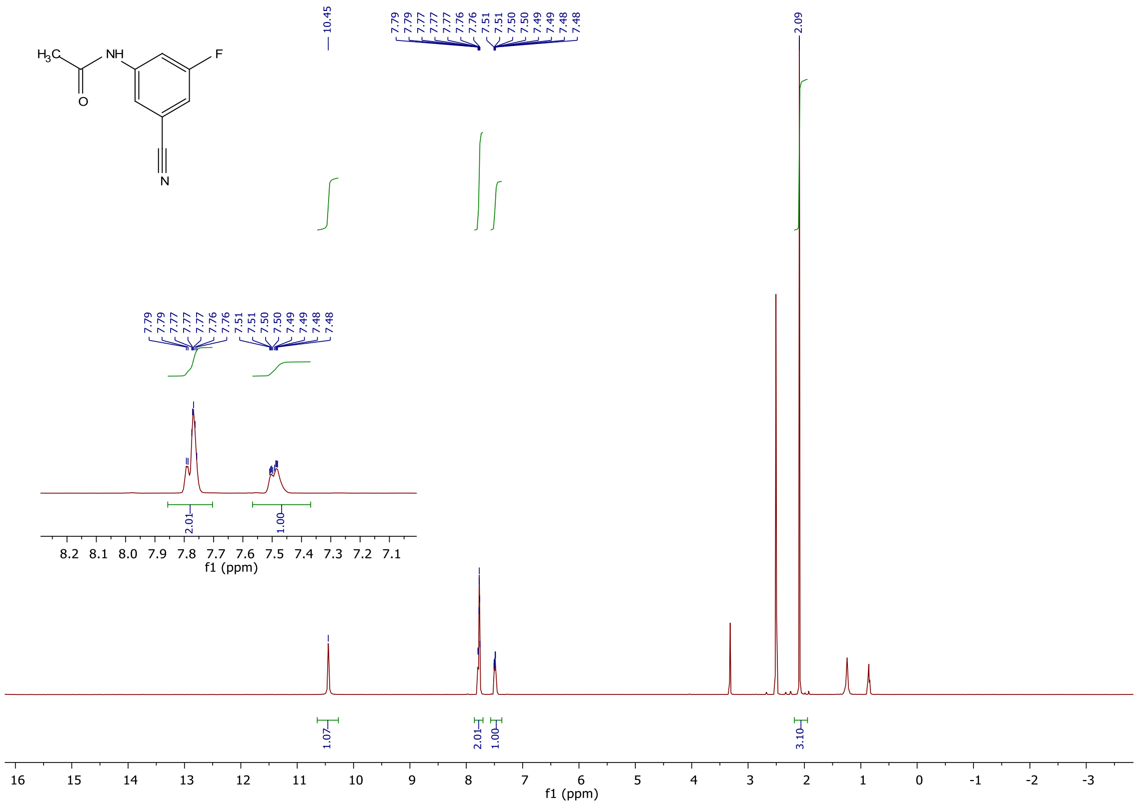

^{13}C NMR spectrum of N-(5-cyano-3-fluorophenyl)acetamide (**16a-*m***) (101 MHz, DMSO-d_6)

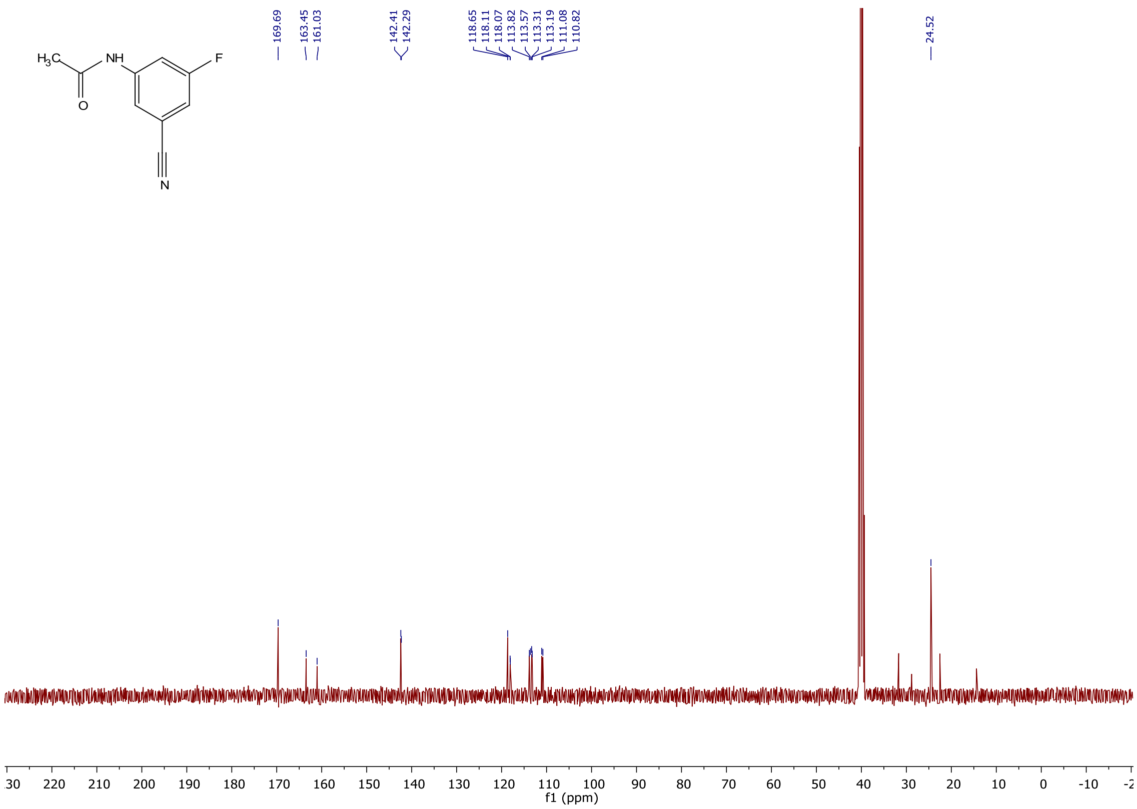

^{1}H NMR spectrum of N-(3-Fluoro-5-(1,2,4,5-tetrazin-3-yl)phenyl)acetamide (**16-*m***) (400 MHz, DMSO-d6)

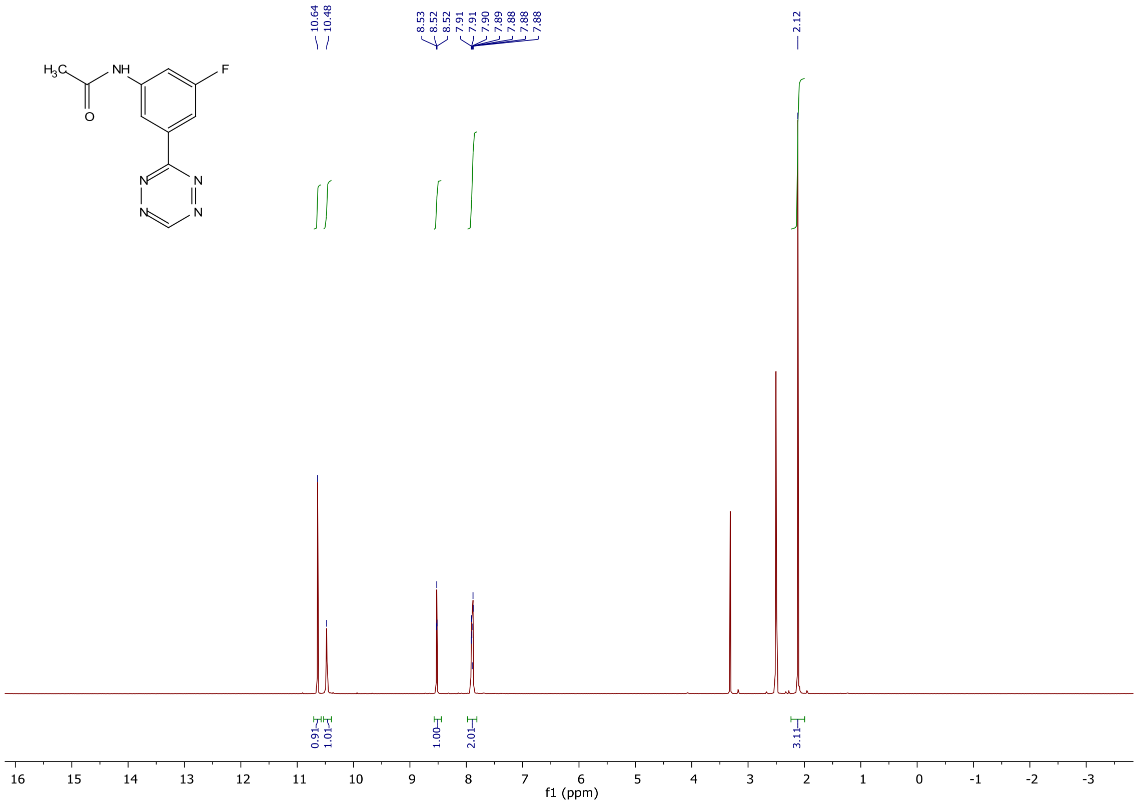

^{13}C NMR spectrum of N-(3-Fluoro-5-(1,2,4,5-tetrazin-3-yl)phenyl)acetamide (**16-*m***) (101 MHz, DMSO-d_6)

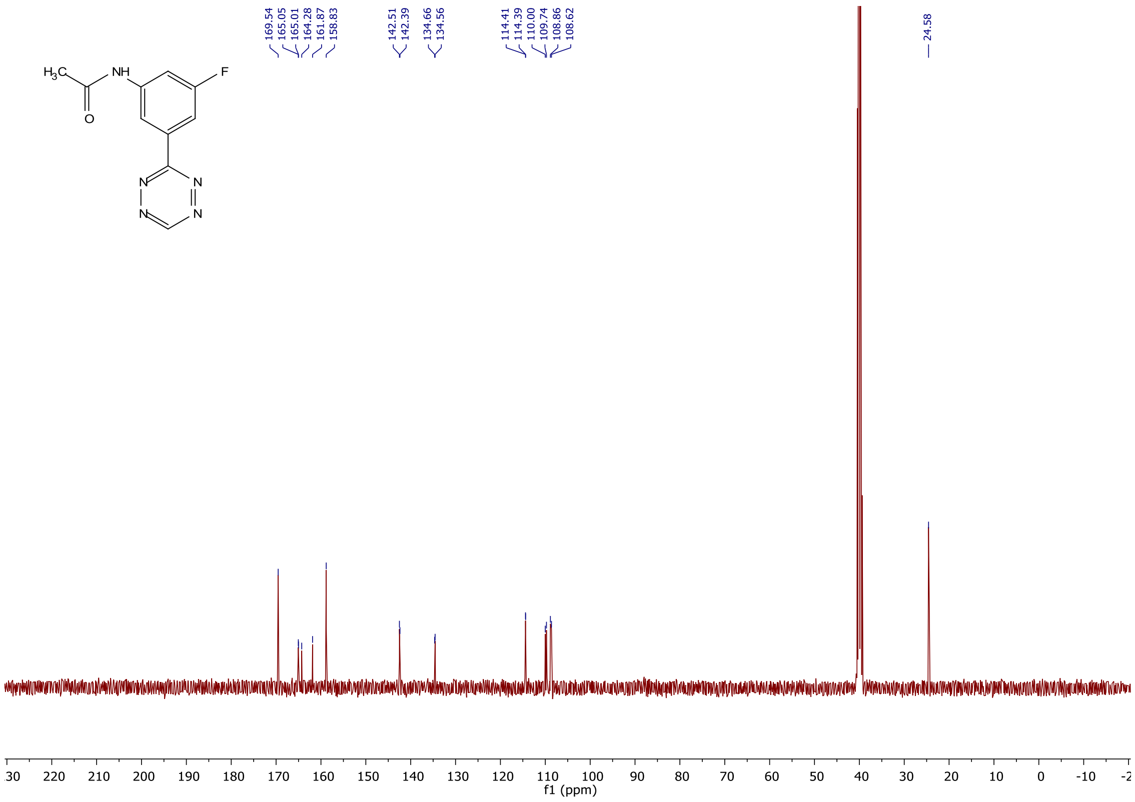

Analytical HPLC of N-(3-Fluoro-5-(1,2,4,5-tetrazin-3-yl)phenyl)acetamide (**16-*m***)

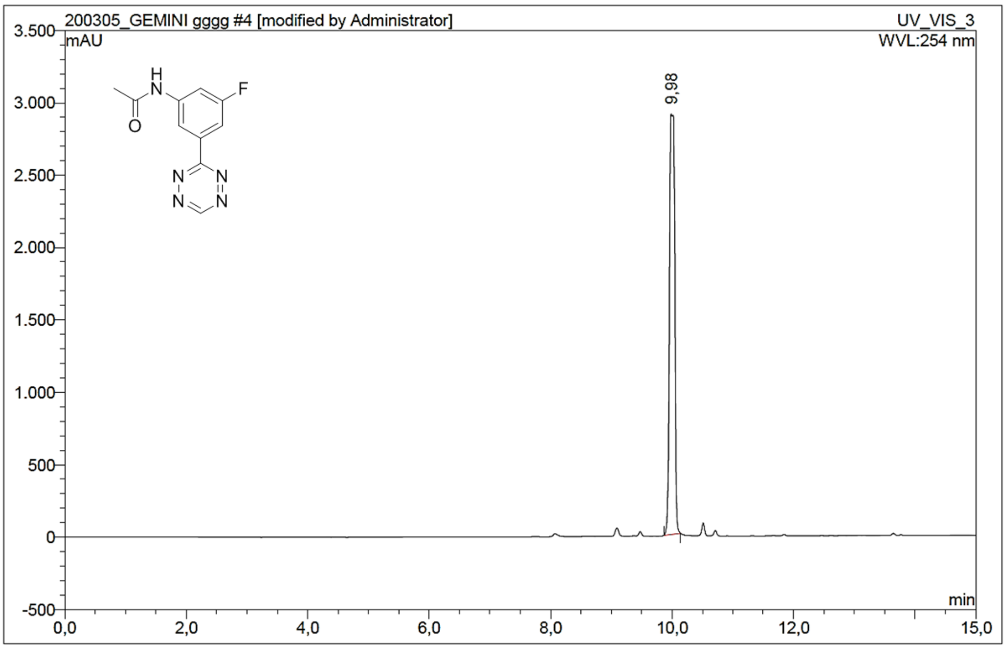

No.	Ret.Time min	Peak Name	Height mAU	Area mAU*min	Rel.Area %	Amount	Resolution(EP)
1	9,98	n.a.	2906,144	287,377	100,00	n.a.	n.a.
Total:			2906,144	287,377	100,00	0,000	

^{1}H NMR spectrum of 3-amino-5-iodobenzonitrile (**36a**) (400 MHz, CDCl3)

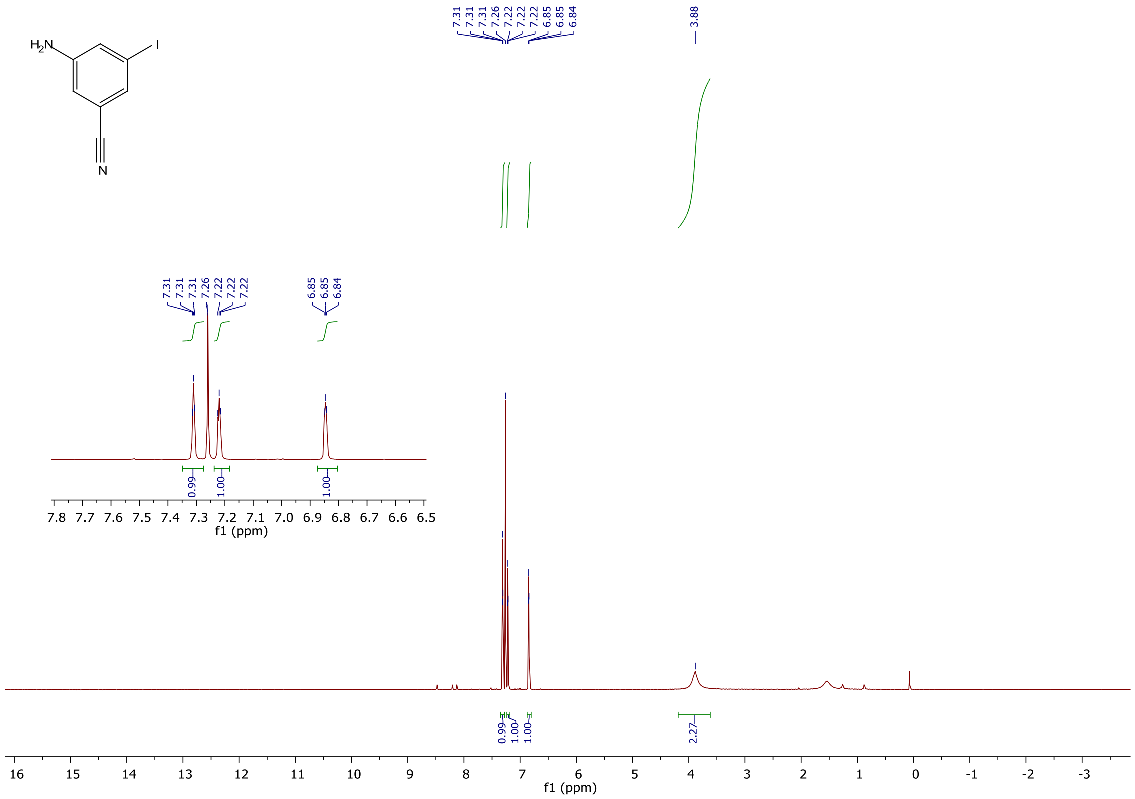

^{13}C NMR spectrum of 3-amino-5-iodobenzonitrile (**36a**) (101 MHz, CDCl3)

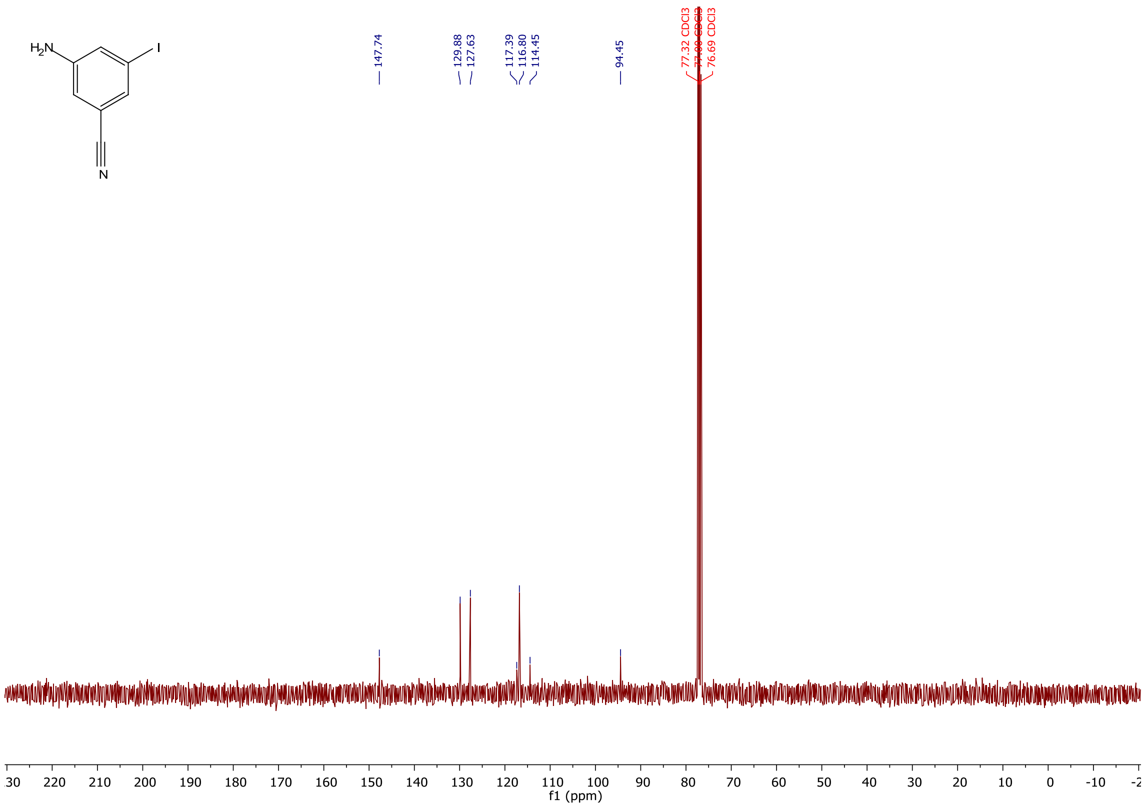

^{1}H NMR spectrum of N-(5-cyano-3-iodophenyl)acetamide (**36b**) (400 MHz, CD_3OD)

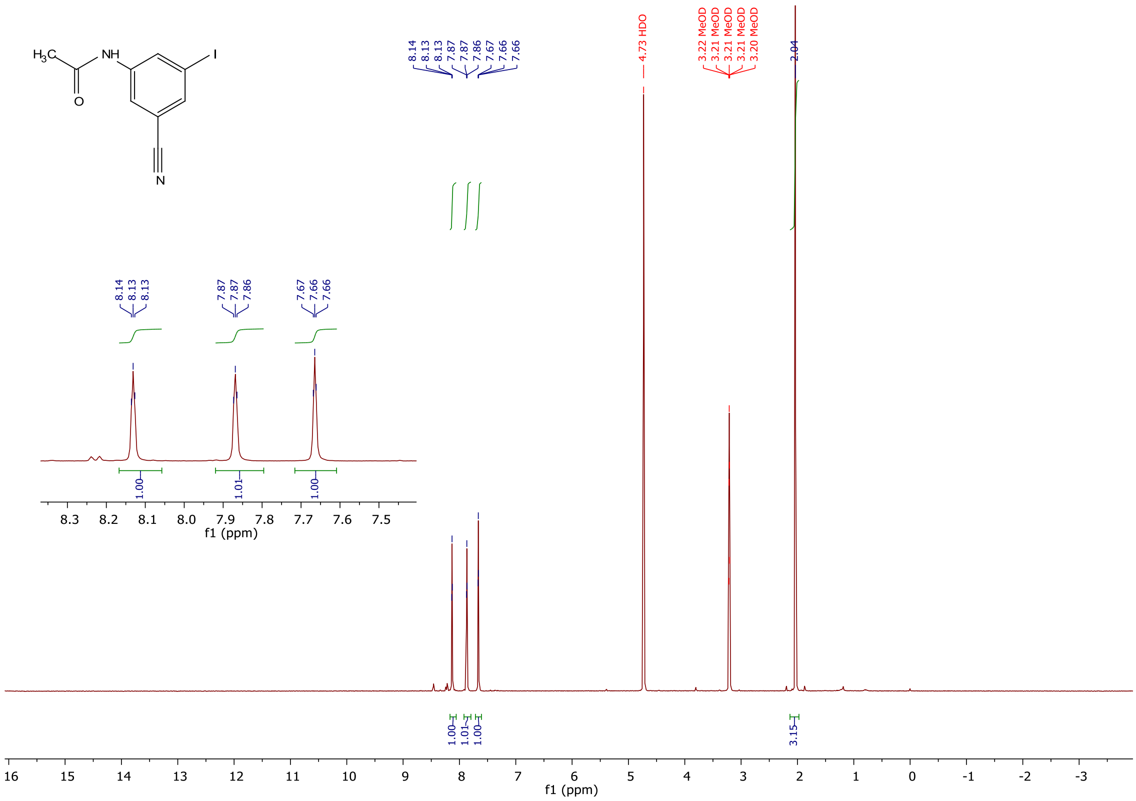

^{13}C NMR spectrum of N-(5-cyano-3-iodophenyl)acetamide (**36b**) (101 MHz, CD_3OD)

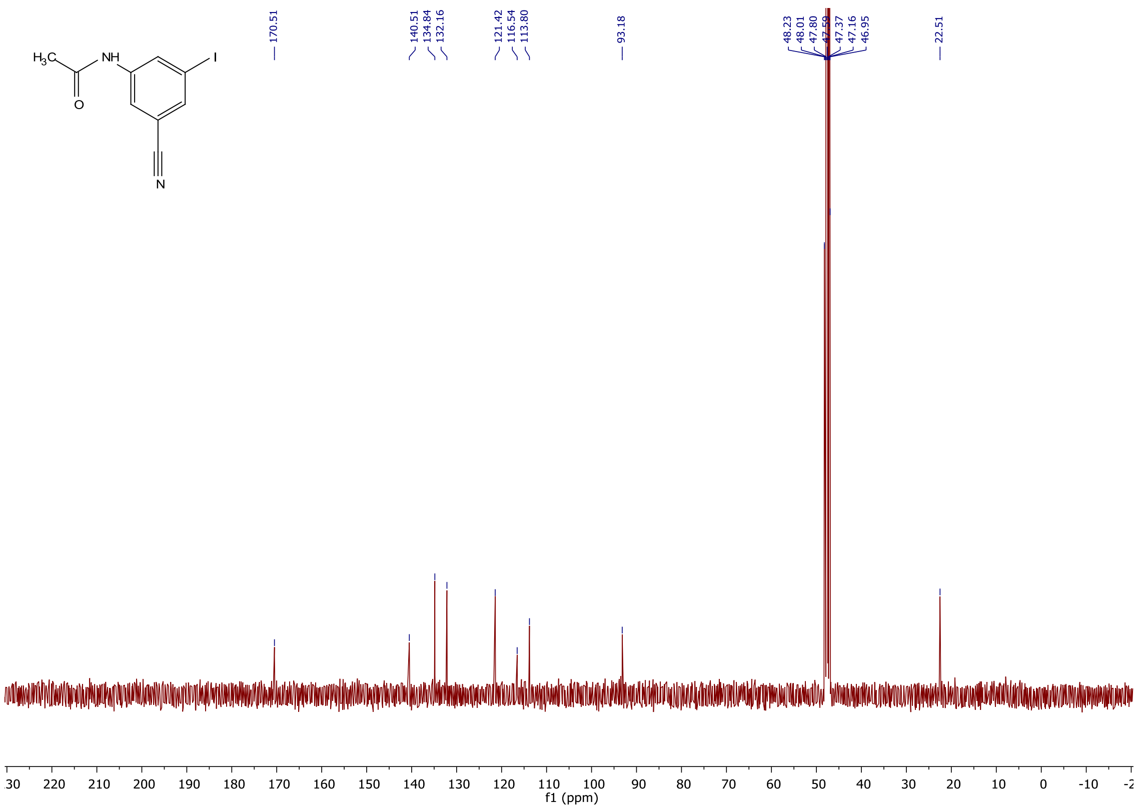

^{1}H NMR spectrum of N-(3-iodo-5-(1,2,4,5-tetrazin-3-yl)phenyl)acetamide (**36c**) (600 MHz, DMSO-d_6)

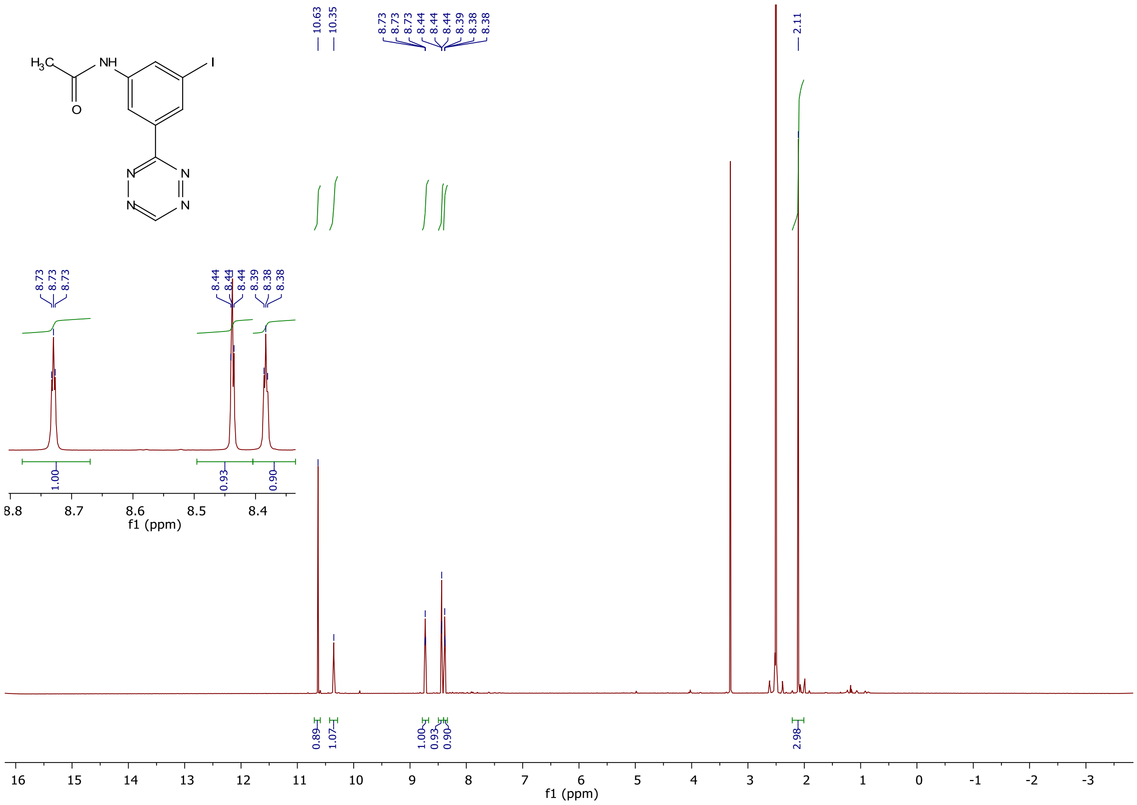

^{13}C NMR spectrum of N-(3-iodo-5-(1,2,4,5-tetrazin-3-yl)phenyl)acetamide (**36c**) (151 MHz, DMSO-d_6)

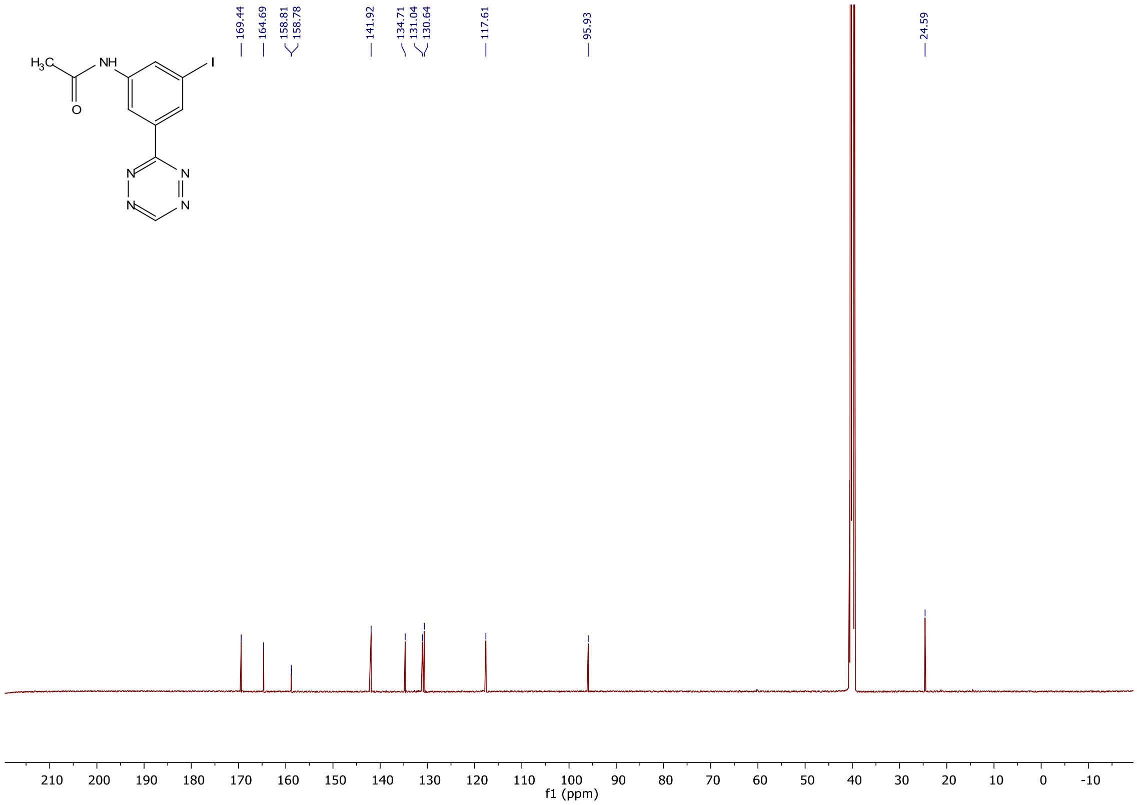

^{1}H NMR spectrum of 3-cyano-5-fluorobenzamide (**17a-*m***) (400 MHz, DMSO-d_6)

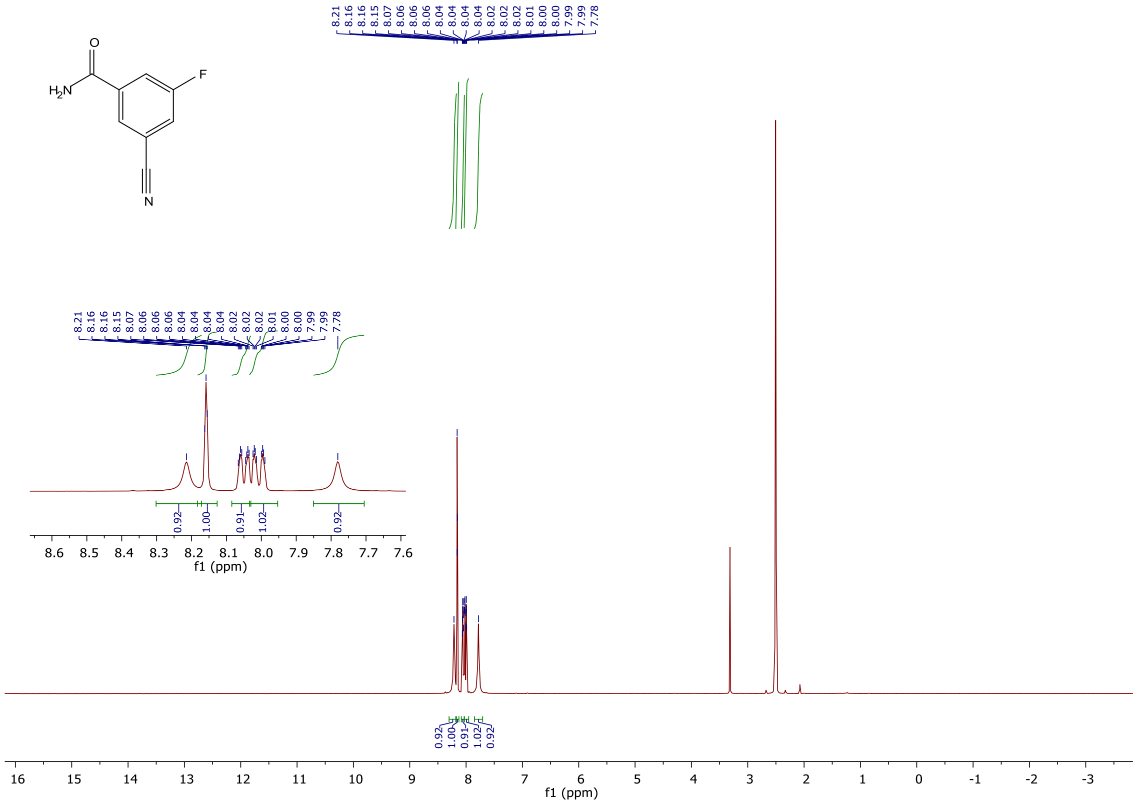

^{13}C NMR spectrum of 3-cyano-5-fluorobenzamide (**17a-*m***) (101 MHz, DMSO-d_6)

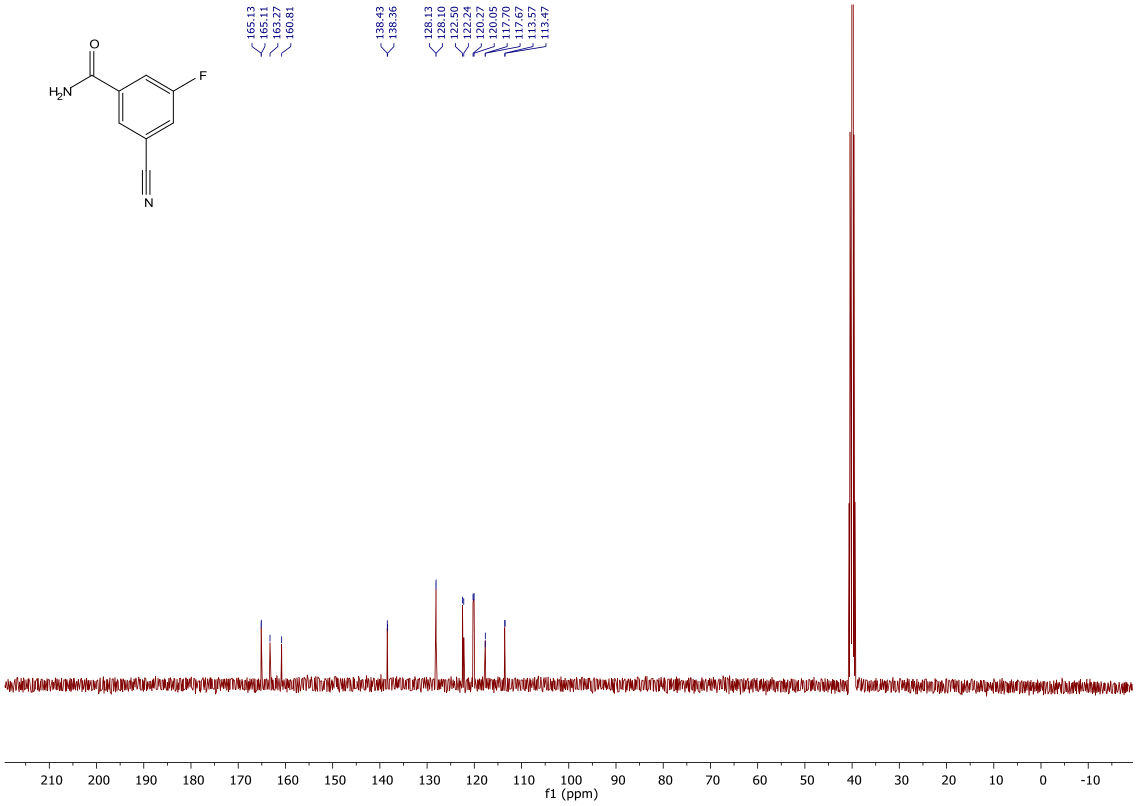

^{1}H NMR spectrum of 3-fluoro-5-(1,2,4,5-tetrazin-3-yl)benzamide (**17-*m***) (400 MHz, DMSO-d_6)

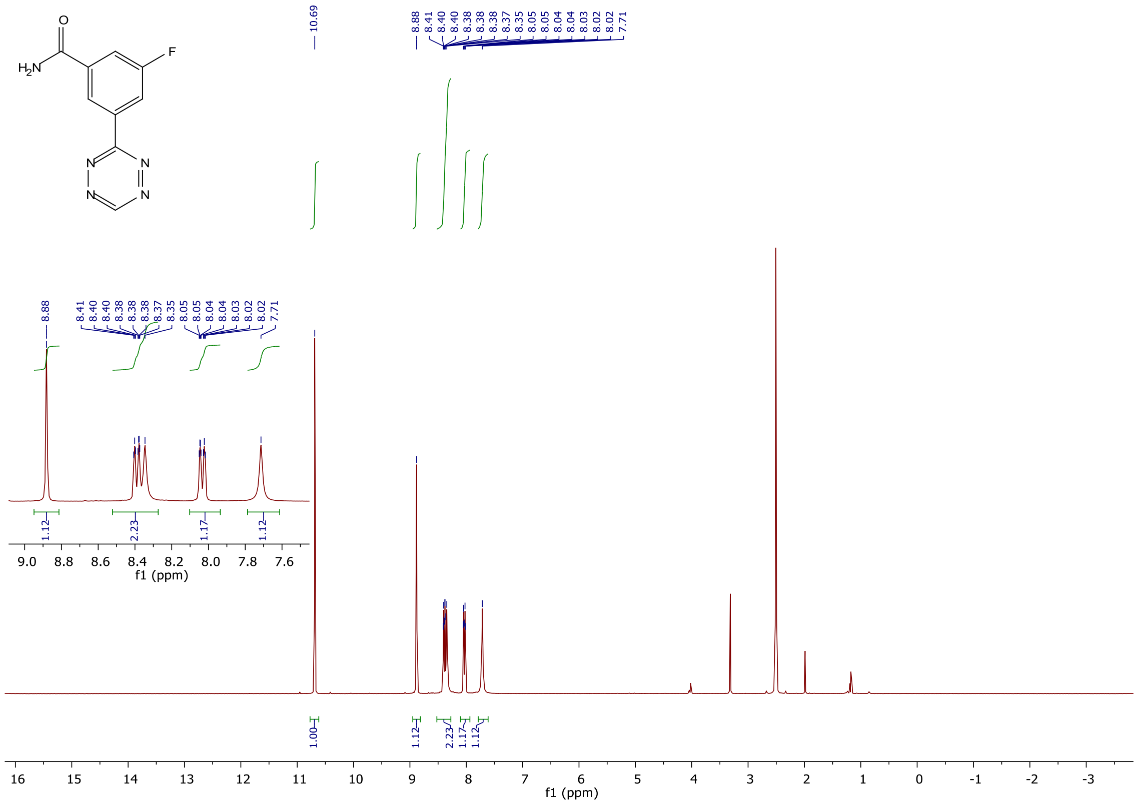

^{13}C NMR spectrum of 3-fluoro-5-(1,2,4,5-tetrazin-3-yl)benzamide (**17-*m***) (101 MHz, DMSO-d_6)

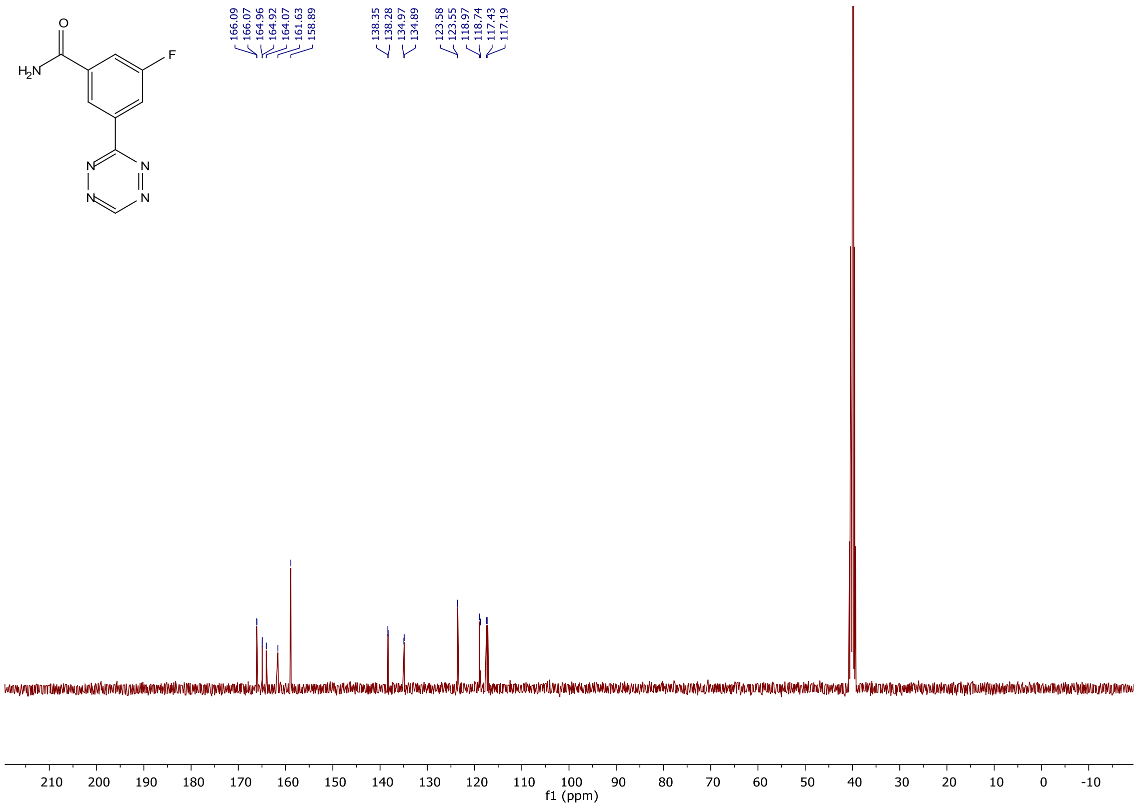

Analytical HPLC of 3-fluoro-5-(1,2,4,5-tetrazin-3-yl)benzamide (**17-*m***)

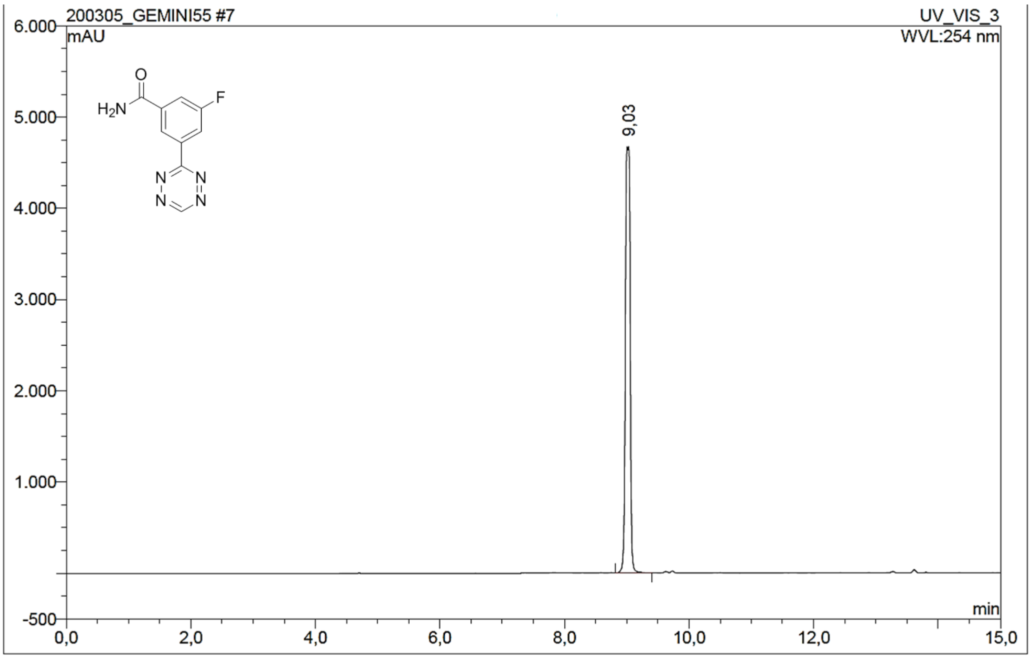

No.	Ret.Time min	Peak Name	Height mAU	Area mAU*min	Rel.Area %	Amount	Resolution(EP)
1	9,03	n.a.	4674,329	415,956	100,00	n.a.	n.a.
Total:			4674,329	415,956	100,00	0,000	

^{1}H NMR spectrum of 3-iodo-5-(methoxycarbonyl)benzoic acid (**37a**) (400 MHz, DMSO-d$_6$)

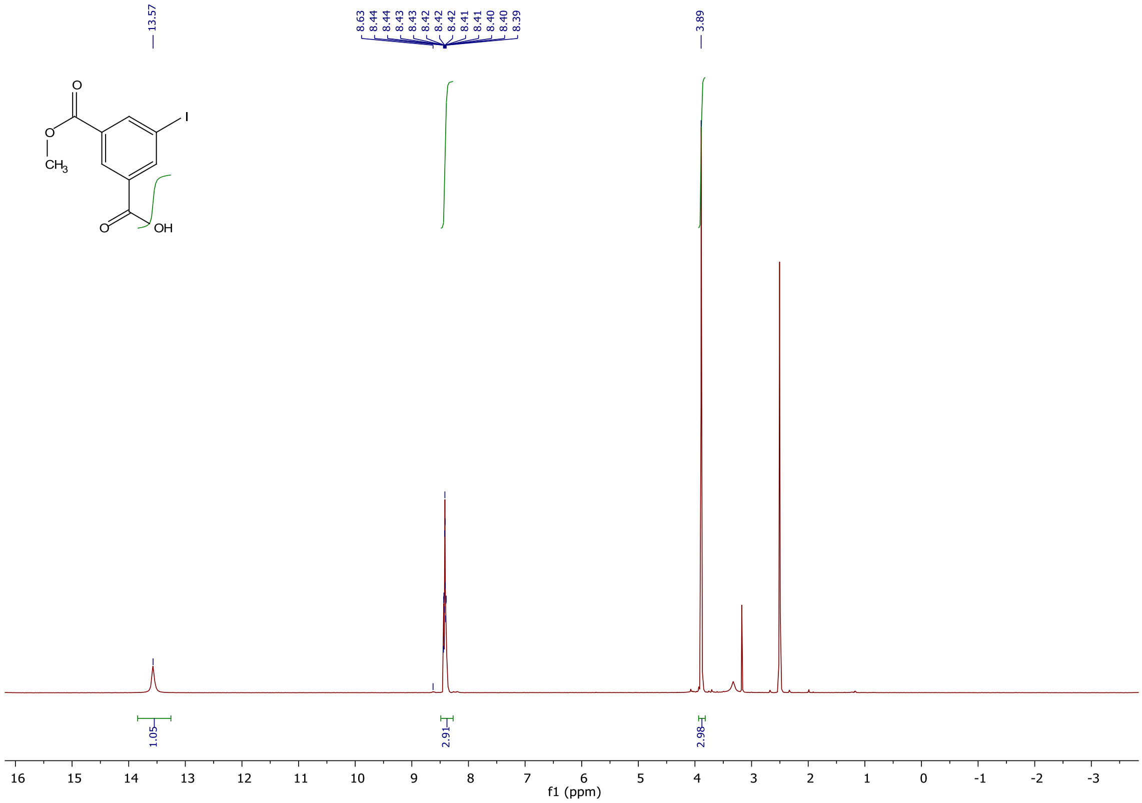

^{13}C NMR spectrum of 3-iodo-5-(methoxycarbonyl)benzoic acid (**37a**) (101 MHz, DMSO-d$_6$)

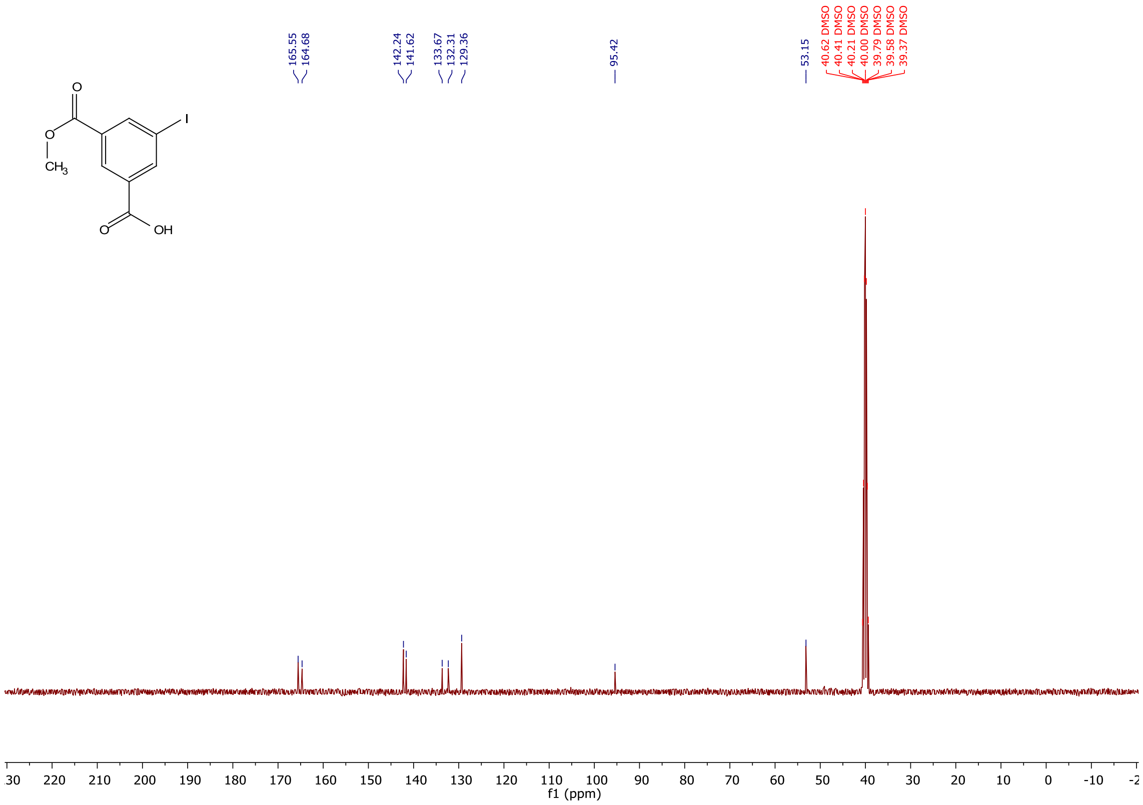

^{1}H NMR spectrum of methyl 3-carbamoyl-5-iodobenzoate (**37b**) (400 MHz, DMSO-d$_6$)

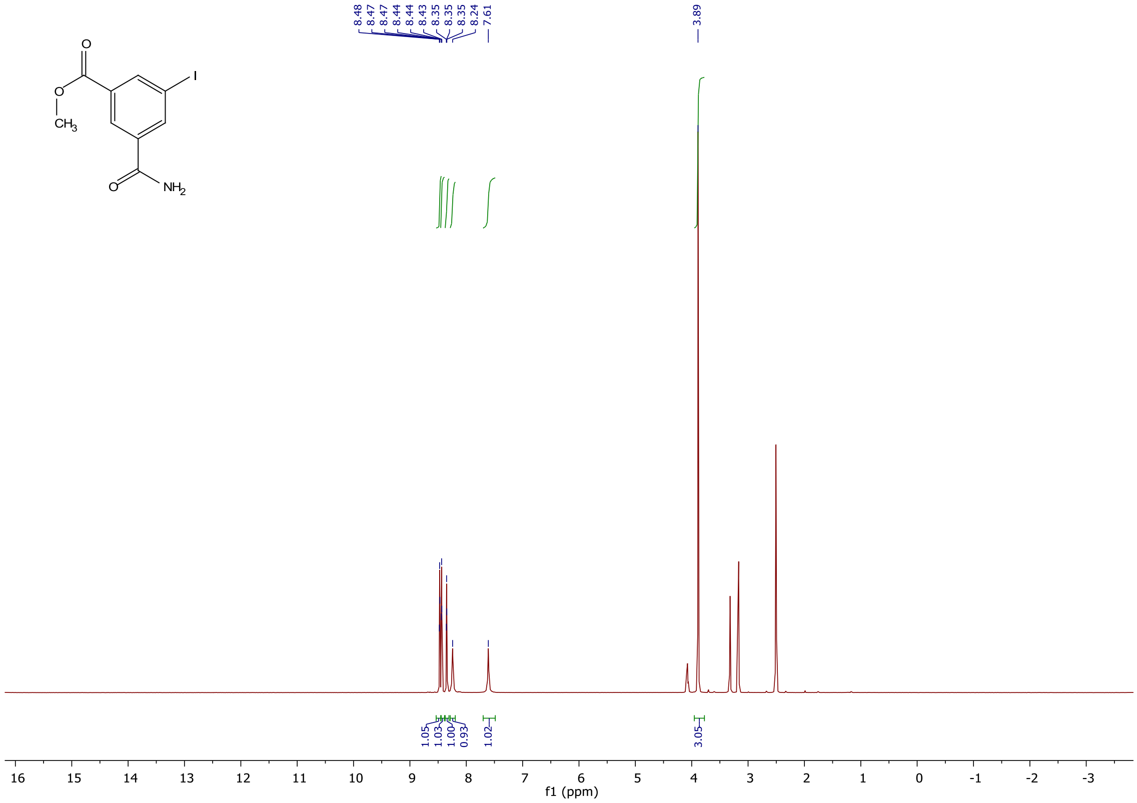

^{13}C NMR spectrum of methyl 3-carbamoyl-5-iodobenzoate (**37b**) (101 MHz, DMSO-d$_6$)

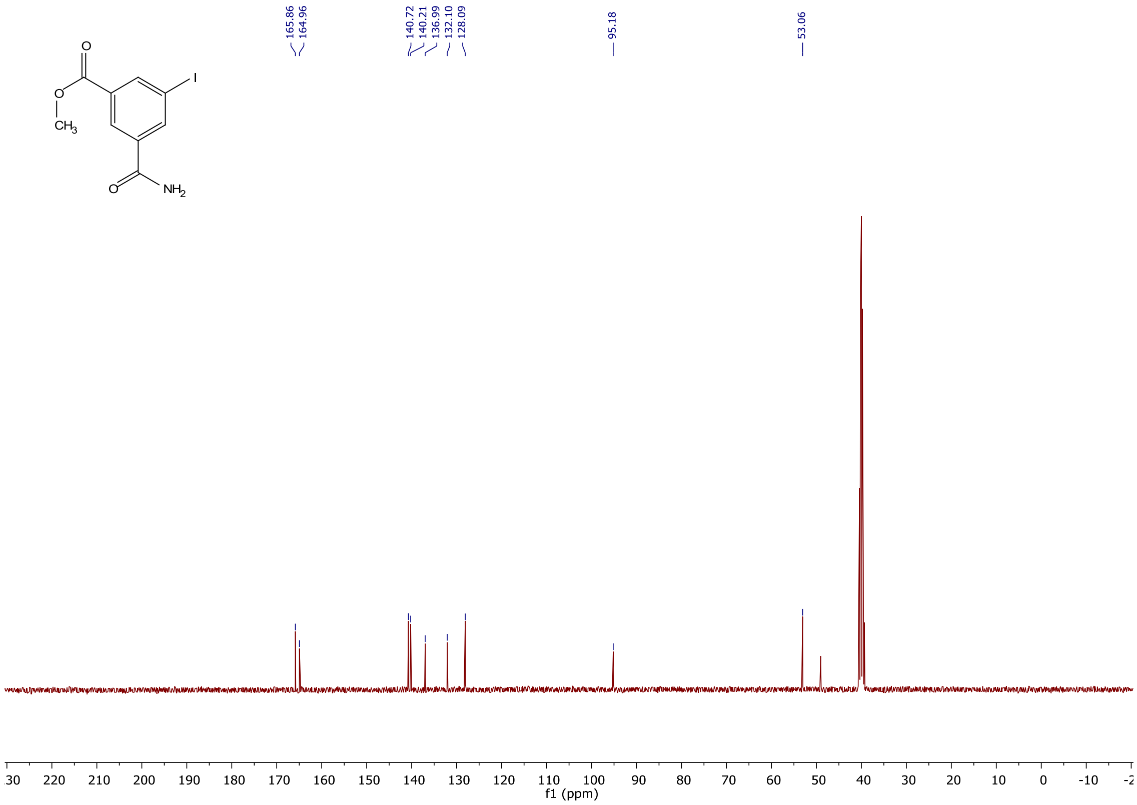

^{1}H NMR spectrum of methyl 3-cyano-5-iodobenzoate (**37c**) (400 MHz, CDCl3)

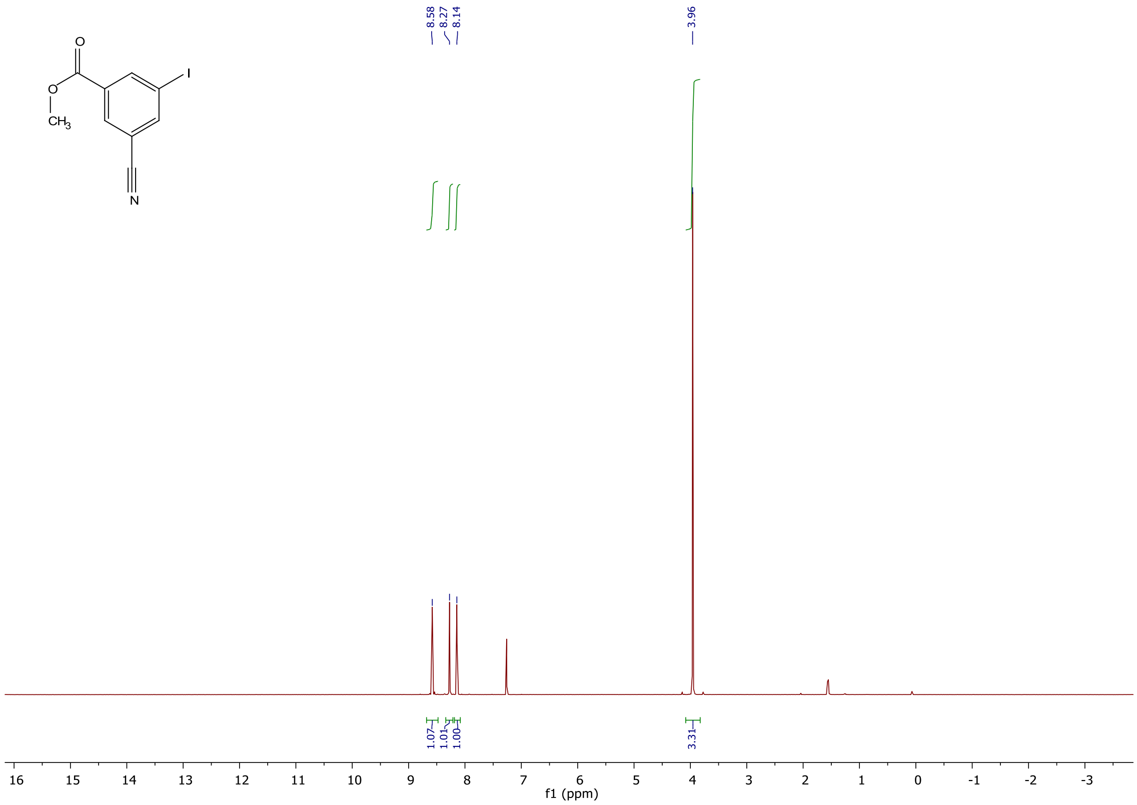

13C NMR spectrum of methyl 3-cyano-5-iodobenzoate (**37c**) (101 MHz, CDCl3)

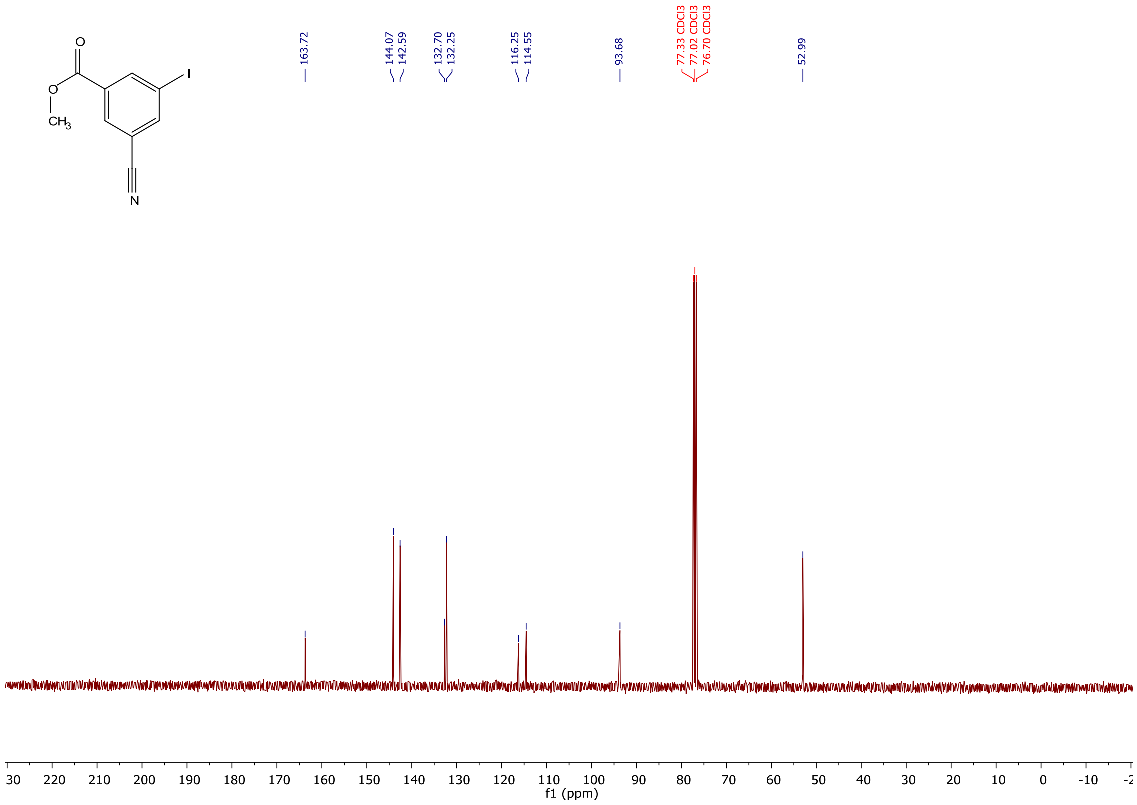

1H NMR spectrum of methyl 3-cyano-5-iodobenzoic acid (**37d**) (400 MHz, CDCl3)

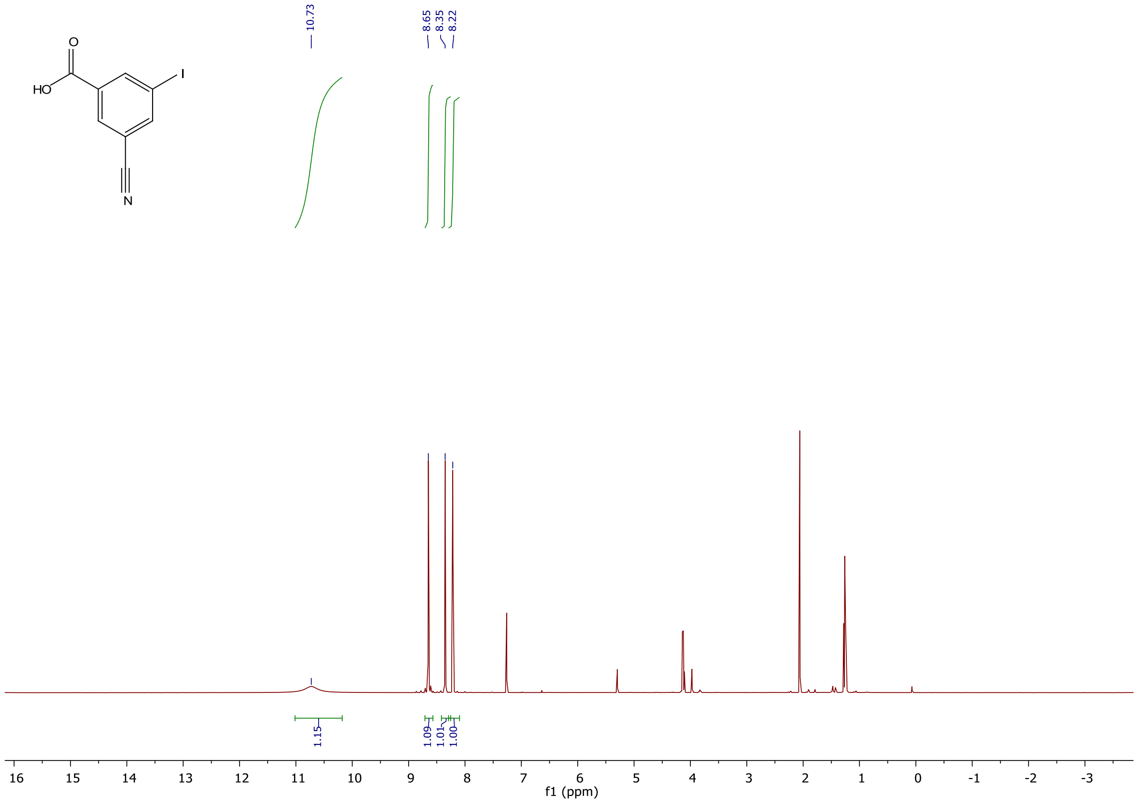

^{13}C NMR spectrum of 3-cyano-5-iodobenzoic acid (**37d**) (101 MHz, CDCl3)

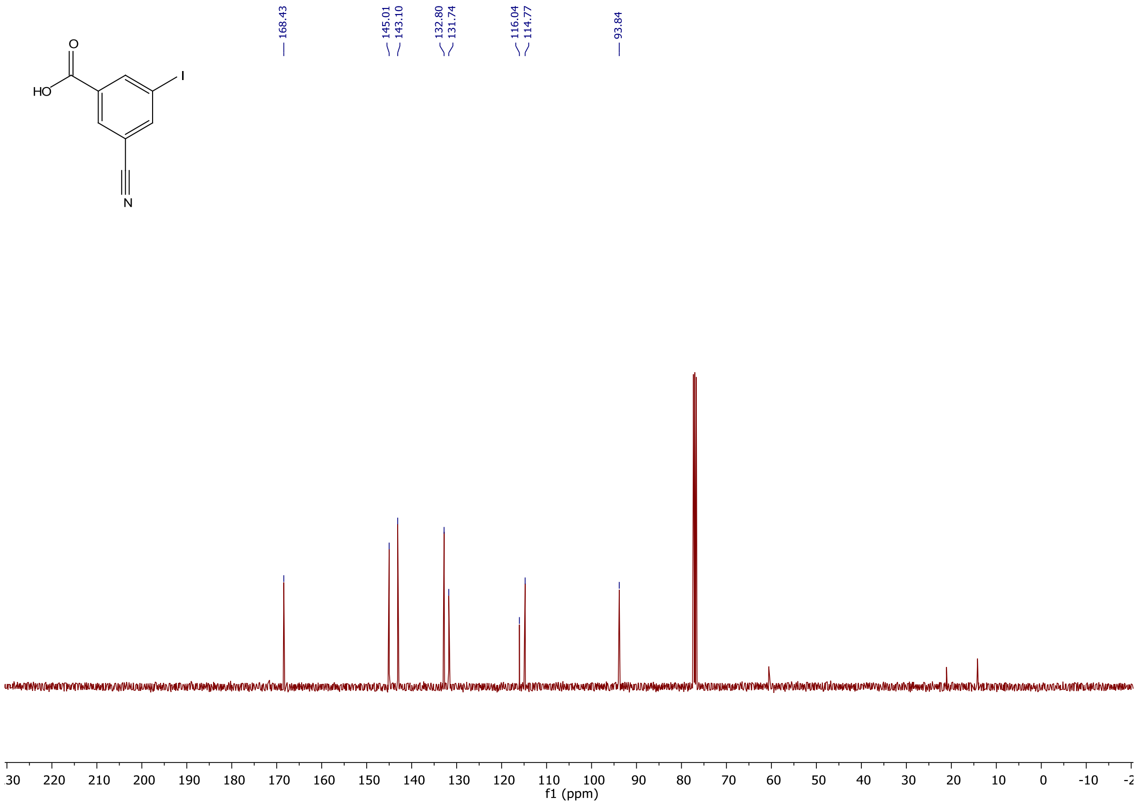

^{1}H NMR spectrum of 3-cyano-5-iodobenzamide (**37e**) (400 MHz, CD_3OD)

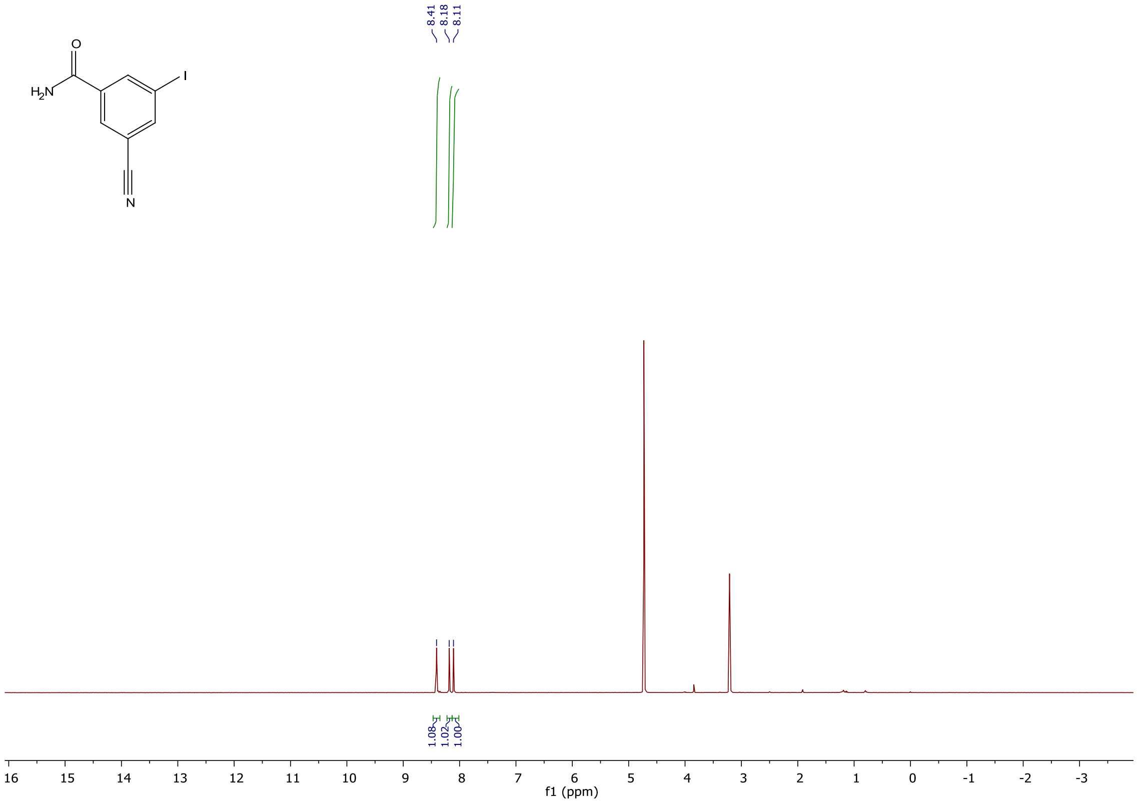

^{13}C NMR spectrum of 3-cyano-5-iodobenzamide (**37e**) (101 MHz, CD_3OD)

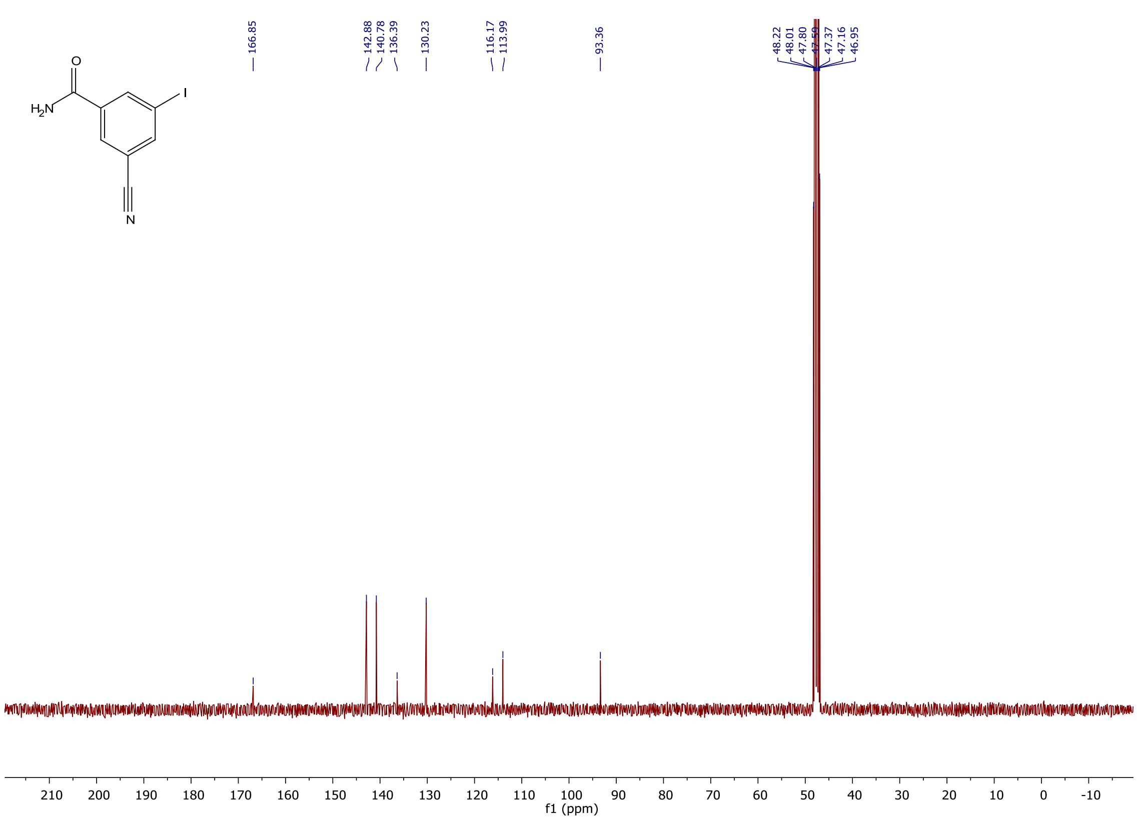

^{1}H NMR spectrum of 3-iodo-5-(1,2,4,5-tetrazin-3-yl)benzamide (**37f**) (400 MHz, DMSO-d_6)

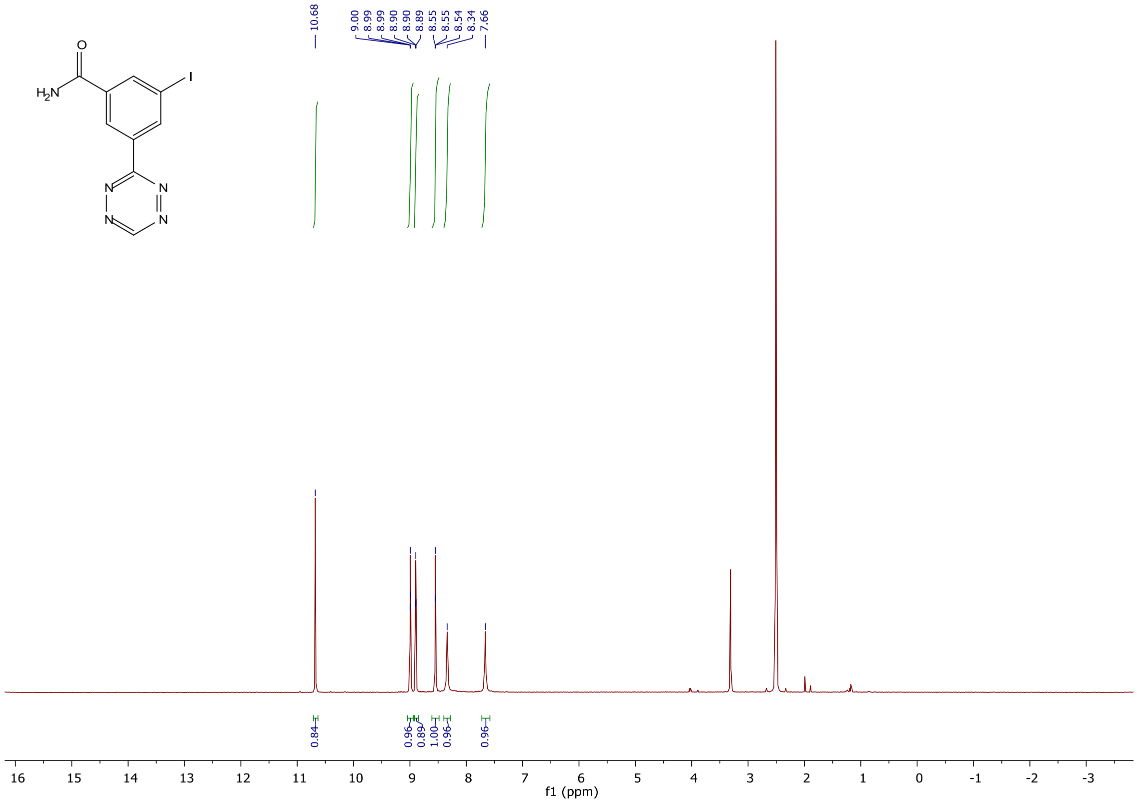

^{13}C NMR spectrum of 3-iodo-5-(1,2,4,5-tetrazin-3-yl)benzamide (**37f**) (101 MHz, DMSO-d$_6$)

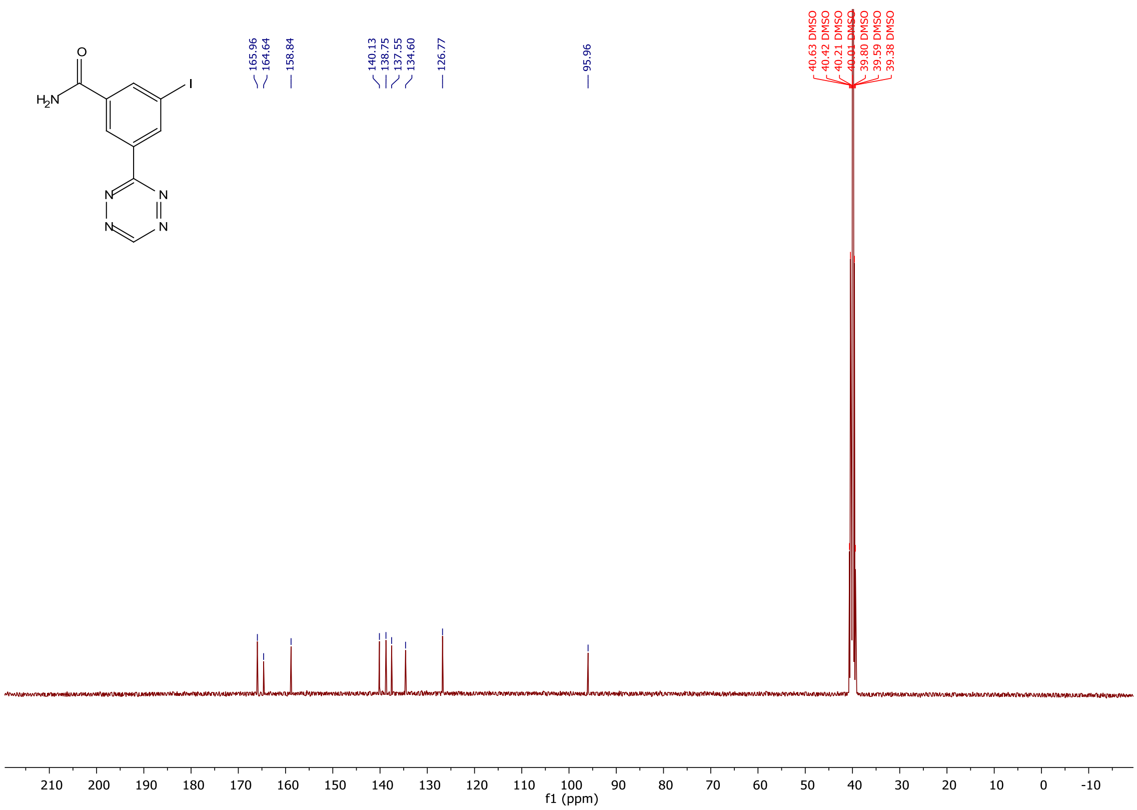

^{1}H NMR spectrum of 5-cyano-3-fluoro-N-methylbenzamide (**18a-*m***) (400 MHz, DMSO-d$_6$)

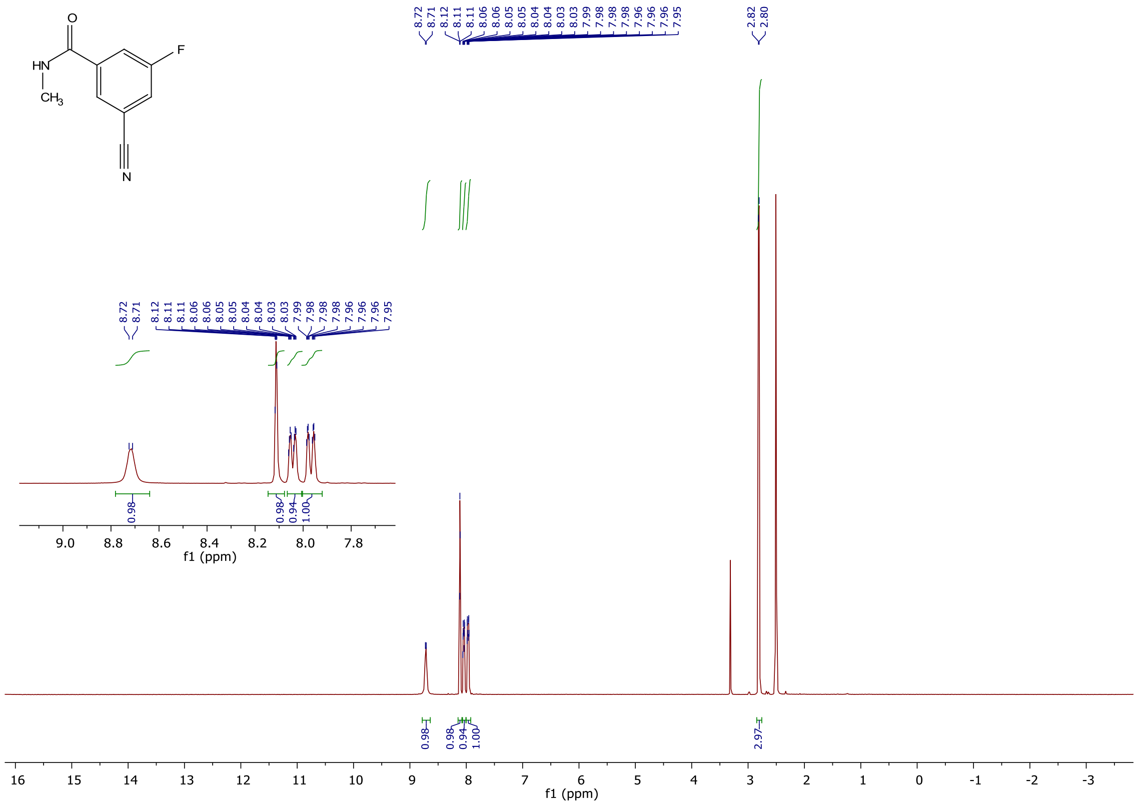

^{13}C NMR spectrum of 5-cyano-3-fluoro-N-methylbenzamide (**18a-*m***) (101 MHz, DMSO-d_6)

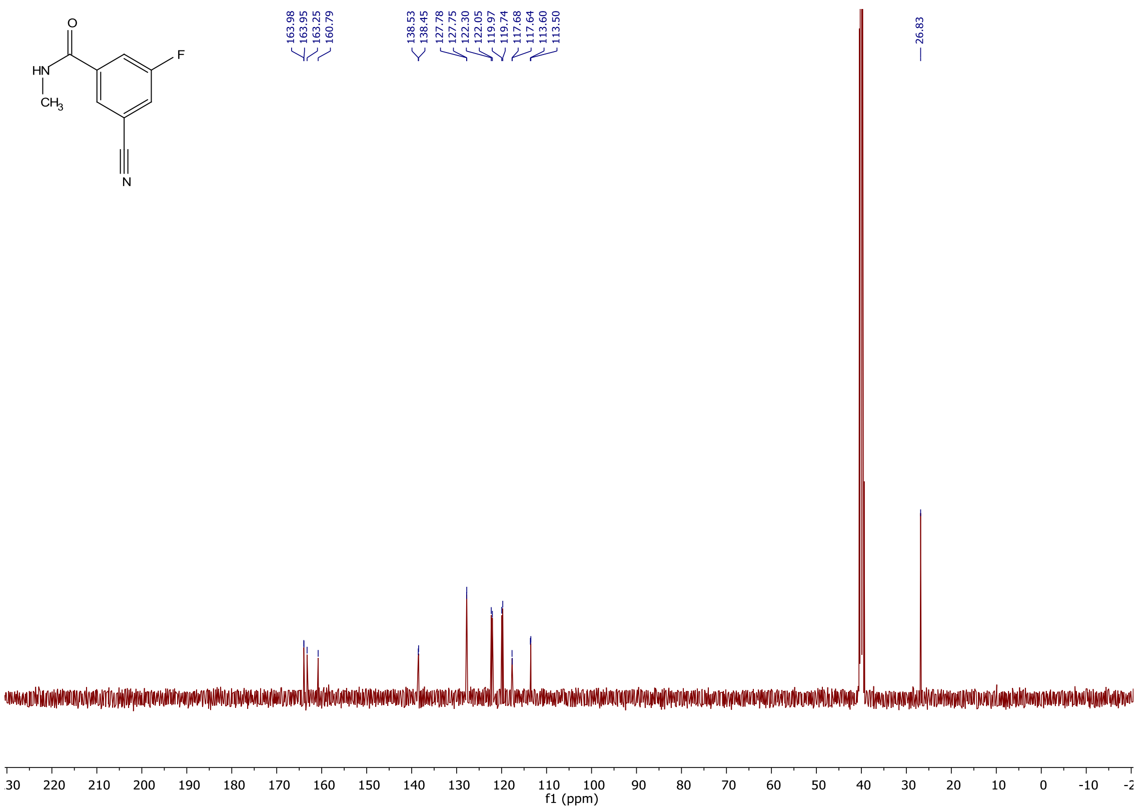

^{1}H NMR spectrum of 3-fluoro-N-methyl-5-(1,2,4,5-tetrazin-3-yl)benzamide (**18-*m***) (400 MHz, DMSO-d_6)

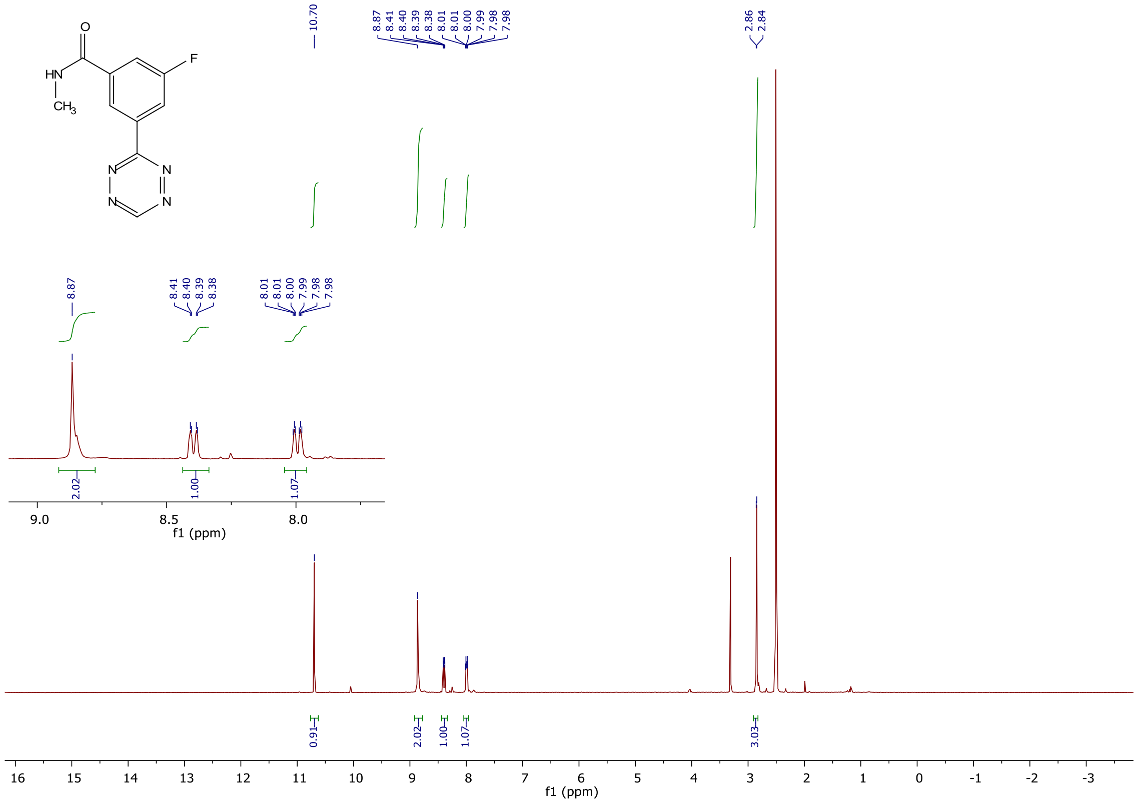

^{13}C NMR spectrum of 3-fluoro-N-methyl-5-(1,2,4,5-tetrazin-3-yl)benzamide (**18-*m***) (101 MHz, DMSO-d_6)

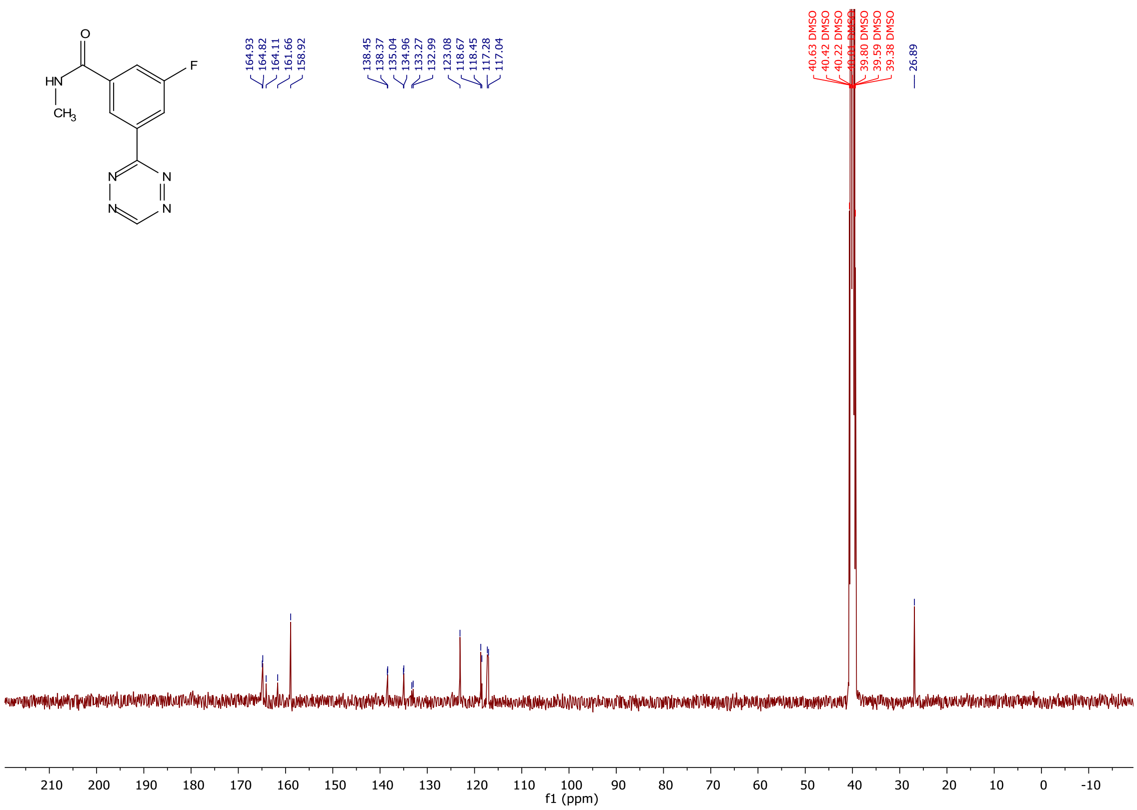

Analytical HPLC of 3-fluoro-N-methyl-5-(1,2,4,5-tetrazin-3-yl)benzamide (**18-*m***)

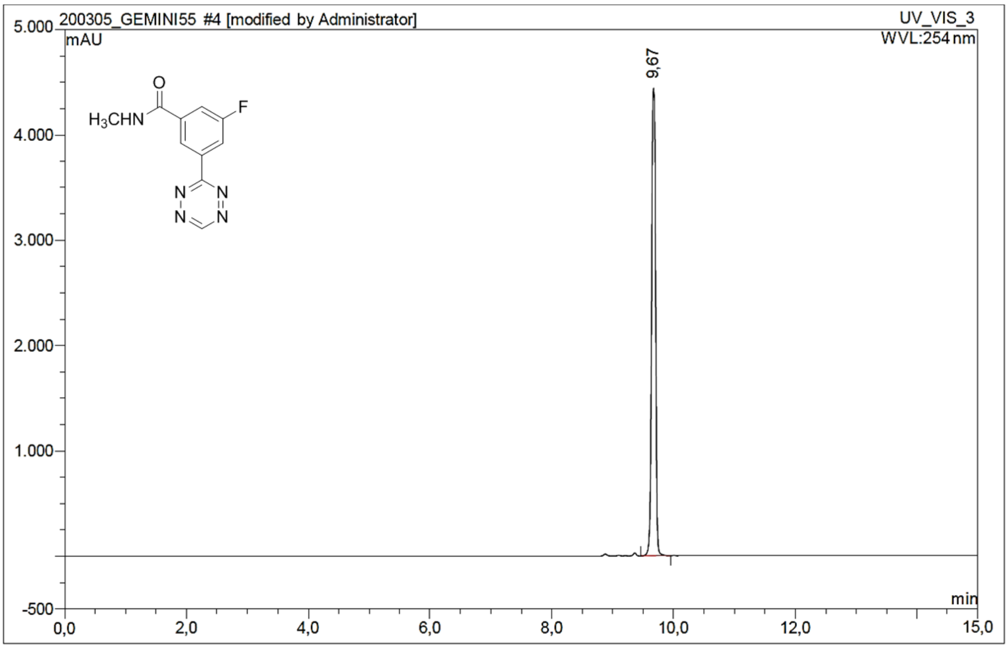

No.	Ret.Time min	Peak Name	Height mAU	Area mAU*min	Rel.Area %	Amount	Resolution(EP)
1	9,67	n.a.	4439,383	329,694	100,00	n.a.	n.a.
Total:			4439,383	329,694	100,00	0,000	

^{1}H NMR spectrum of 5-cyano-3-iodo-N-methylbenzamide (**38a**) (400 MHz, CD_3OD)

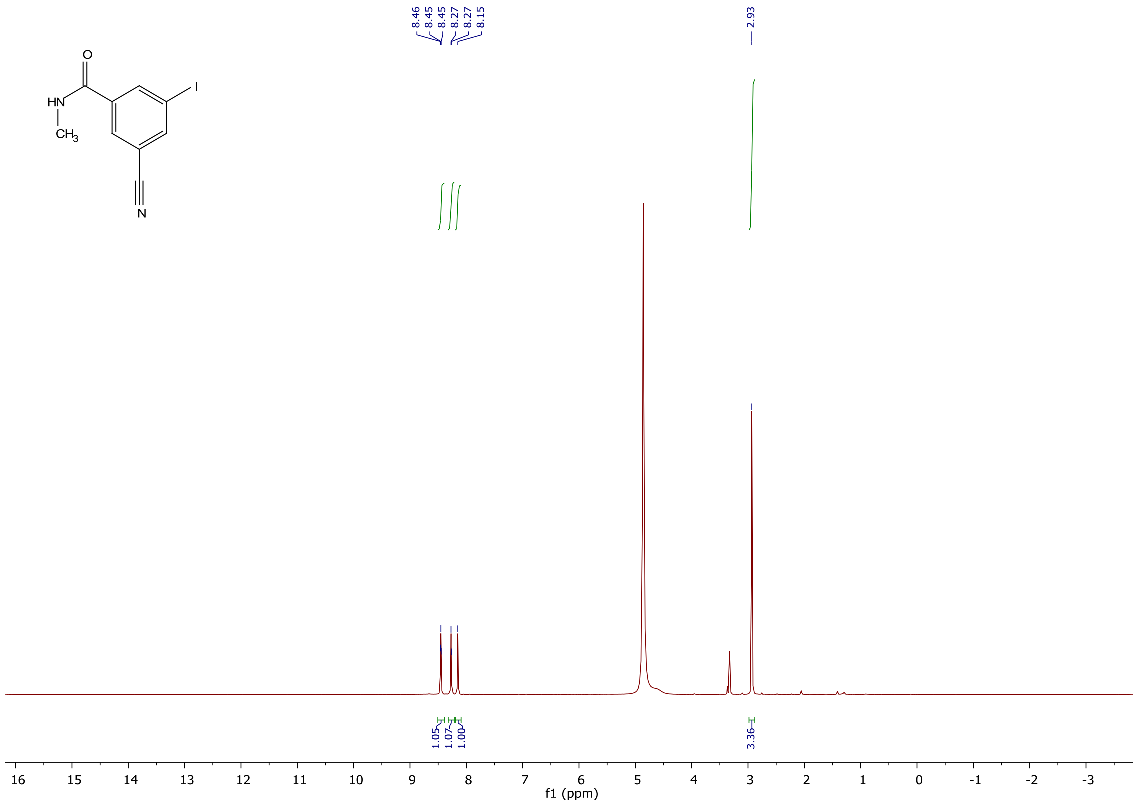

^{13}C NMR spectrum of 5-cyano-3-iodo-N-methylbenzamide (**38a**) (101 MHz, CD_3OD)

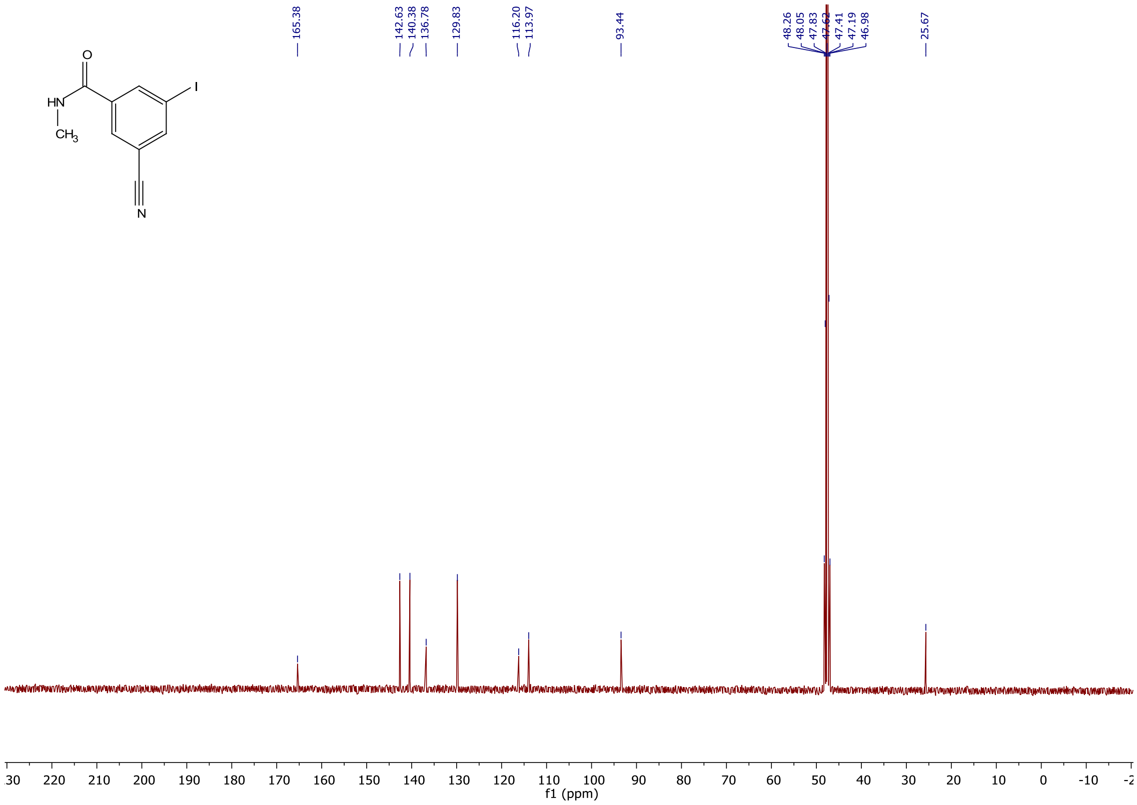

^{1}H NMR spectrum of 3-iodo-N-methyl-5-(1,2,4,5-tetrazin-3-yl)benzamide (**38b**) (400 MHz, DMSO-d$_6$)

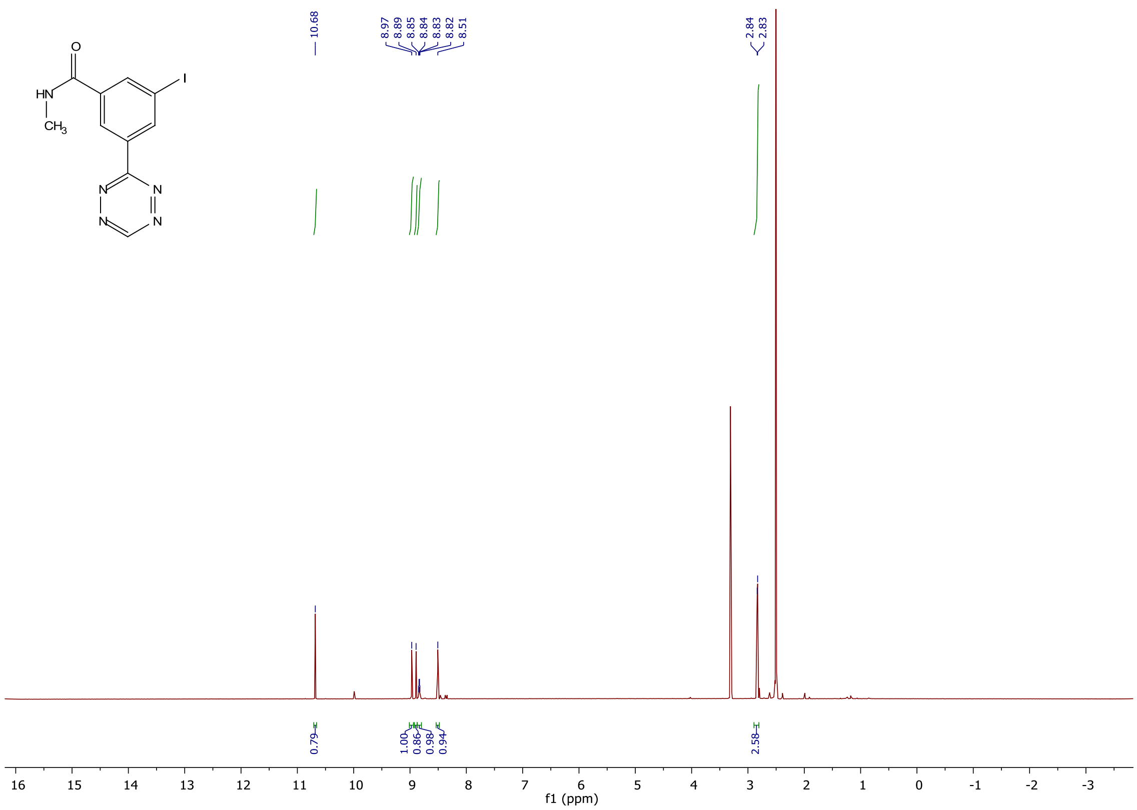

^{13}C NMR spectrum of 3-iodo-N-methyl-5-(1,2,4,5-tetrazin-3-yl)benzamide (**38b**) (101 MHz, DMSO-d$_6$)

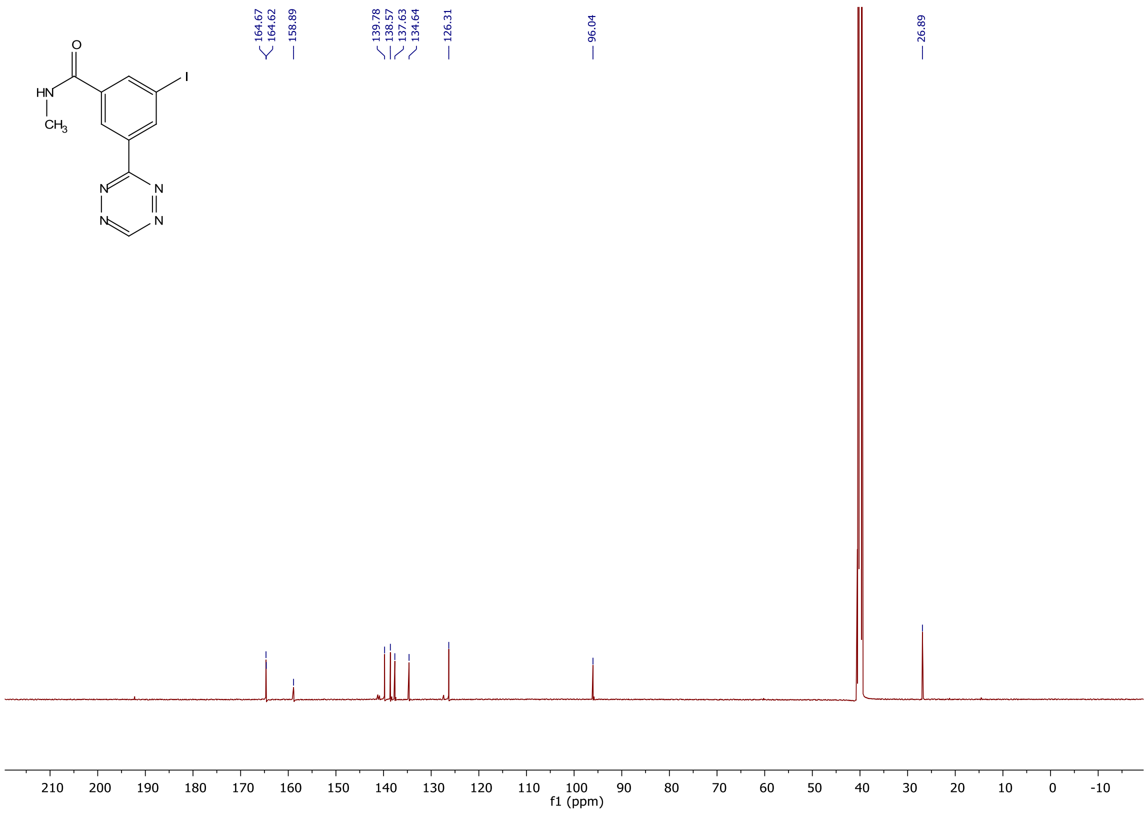

^{1}H NMR spectrum of 3-(3-fluoro-6-methylphenyl)-1,2,4,5-tetrazine (**14-*o***) (600 MHz, CDCl3)

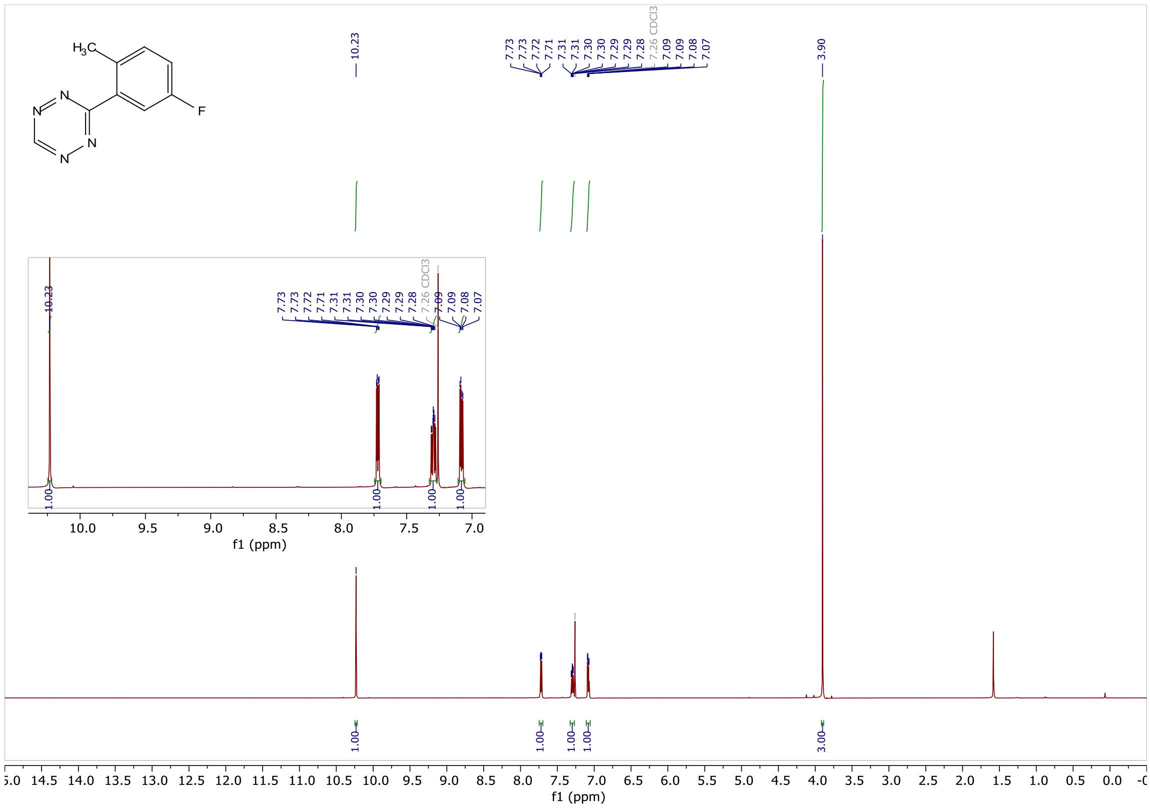

^{13}C NMR spectrum of 3-(3-fluoro-6-methylphenyl)-1,2,4,5-tetrazine (**14-*o***) (151 MHz, CDCl3)

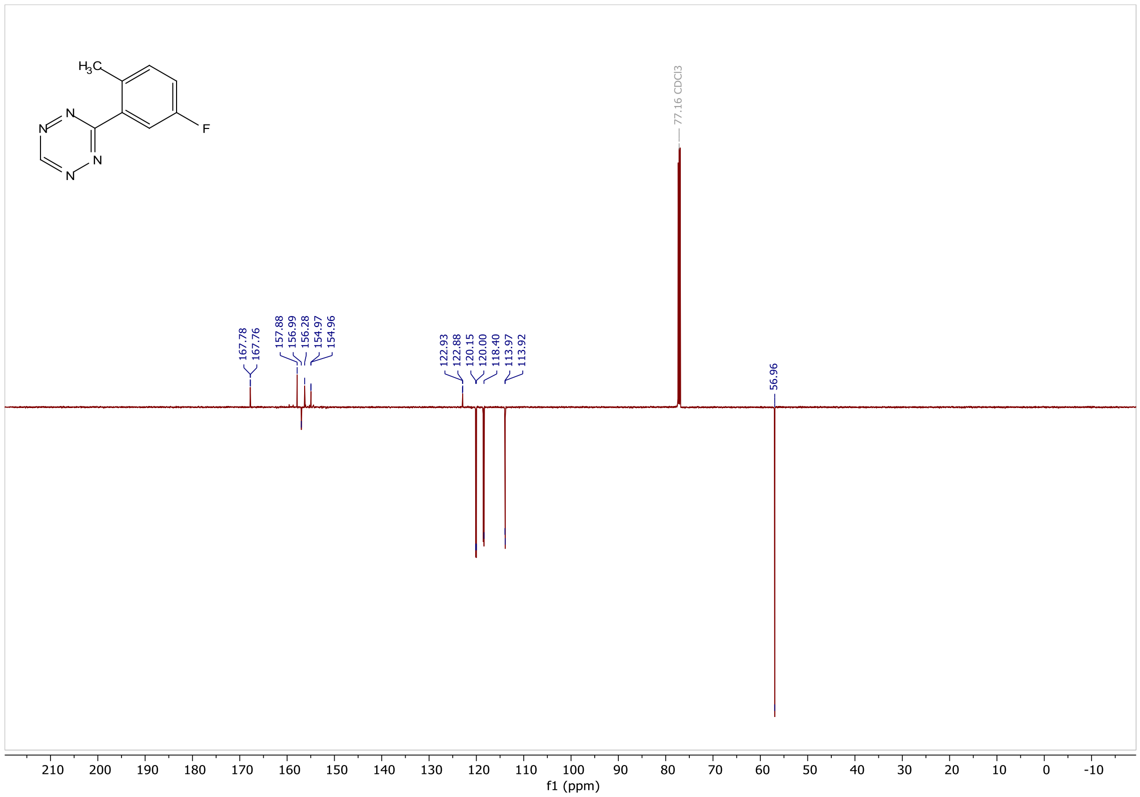

Analytical HPLC of 3-(3-fluoro-6-methylphenyl)-1,2,4,5-tetrazine (**14-*o***)

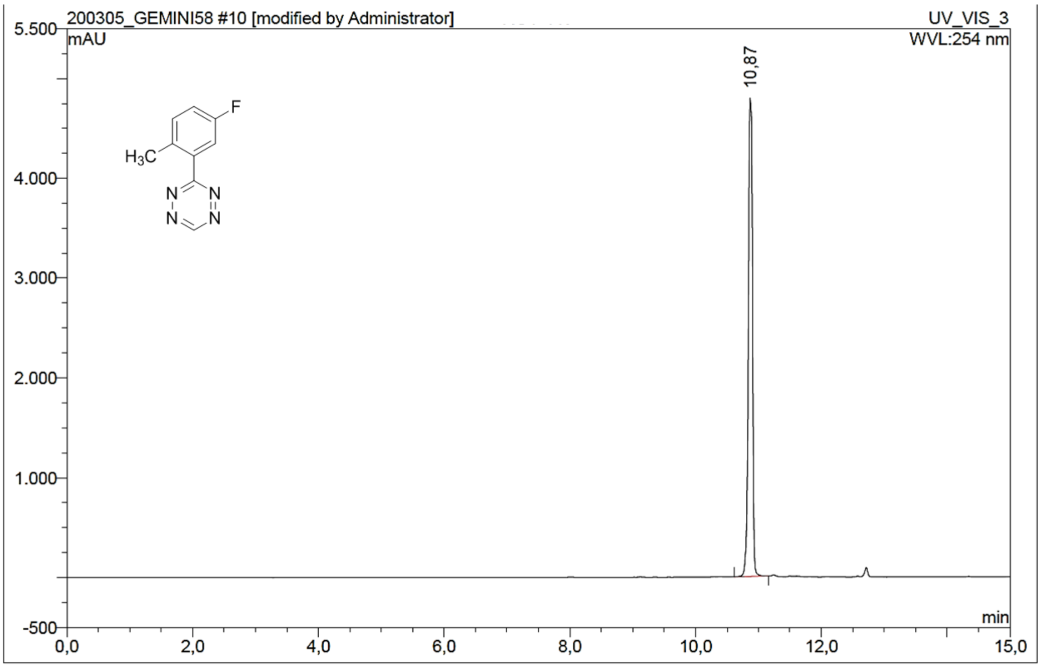

No.	Ret.Time min	Peak Name	Height mAU	Area mAU*min	Rel.Area %	Amount	Resolution(EP)
1	10,87	n.a.	4790,359	370,750	100,00	n.a.	n.a.
**Total:**			4790,359	370,750	100,00	0,000	

^{1}H NMR spectrum of 3-(5-iodo-2-methylphenyl)-1,2,4,5-tetrazine (**39a**) (600 MHz, $CDCl_3$)

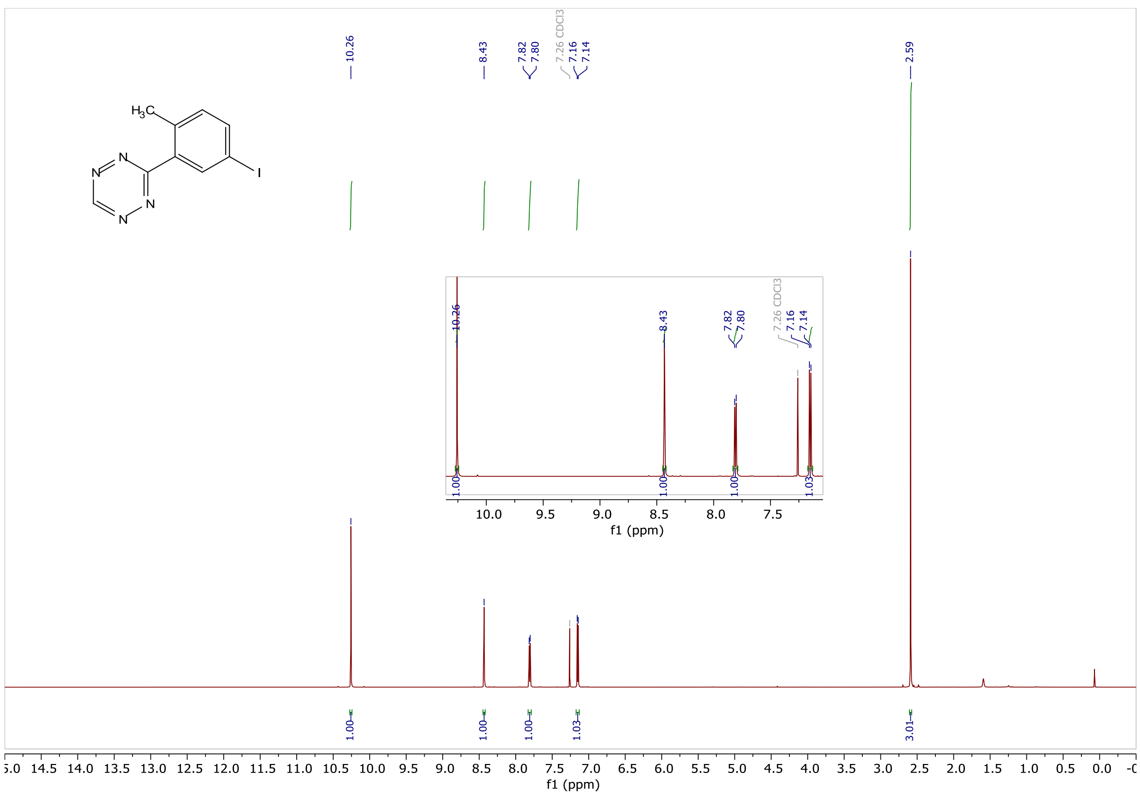

^{13}C NMR spectrum of 3-(5-iodo-2-methylphenyl)-1,2,4,5-tetrazine (**39a**) (151 MHz, CDCl3)

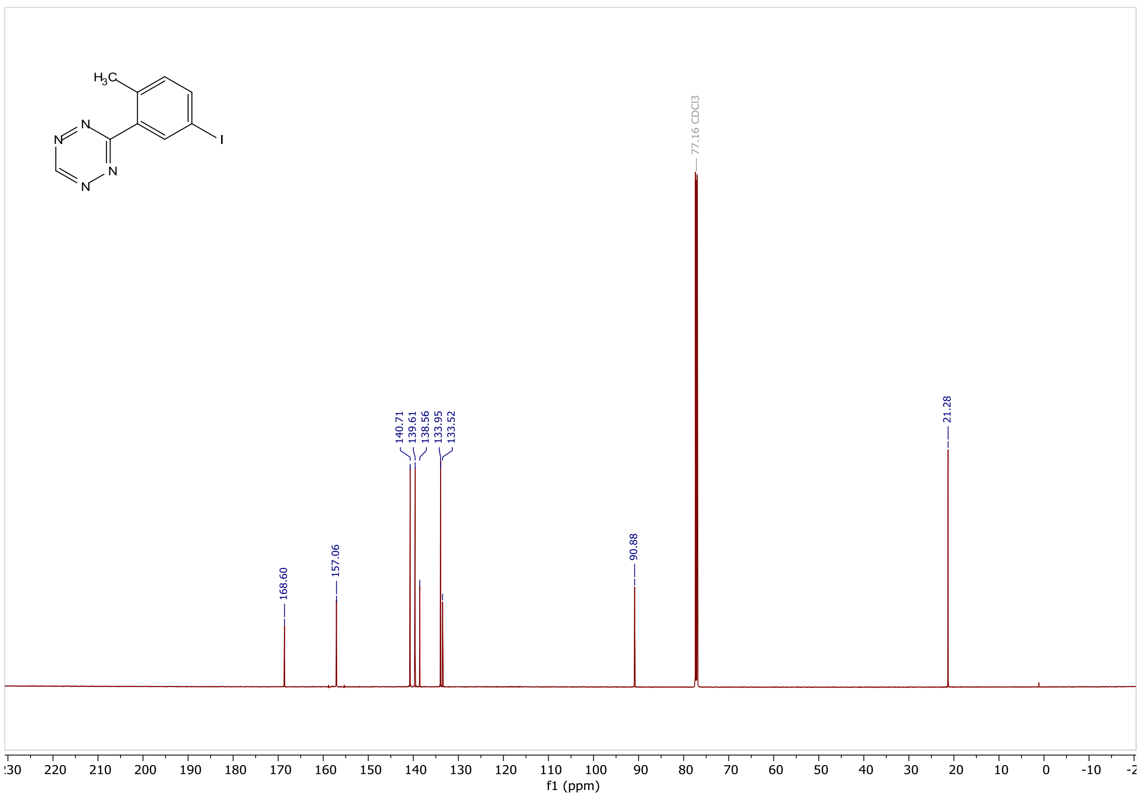

^{1}H NMR spectrum of 3-(3-fluoro-6-methoxyphenyl)-1,2,4,5-tetrazine (**15-*o***) (600 MHz, $CDCl_3$)

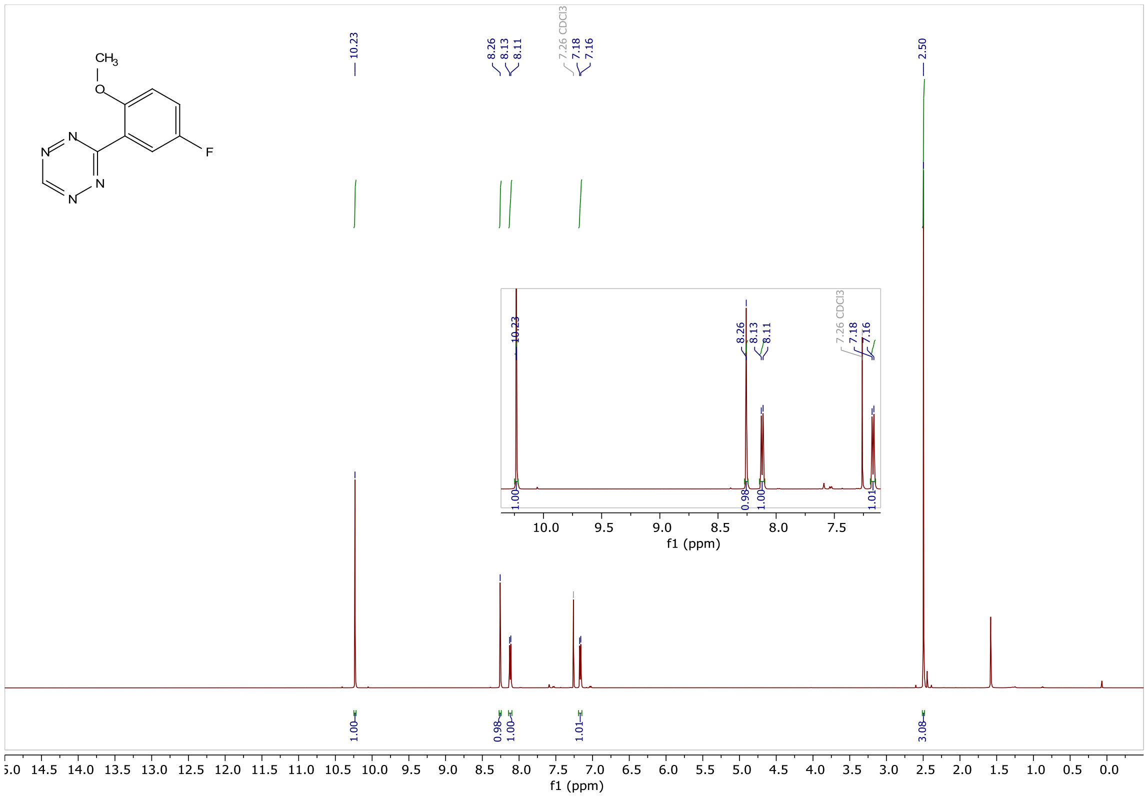

^{13}C NMR spectrum of 3-(3-fluoro-6-methoxyphenyl)-1,2,4,5-tetrazine (**15-*o***) (101 MHz, CDCl3)

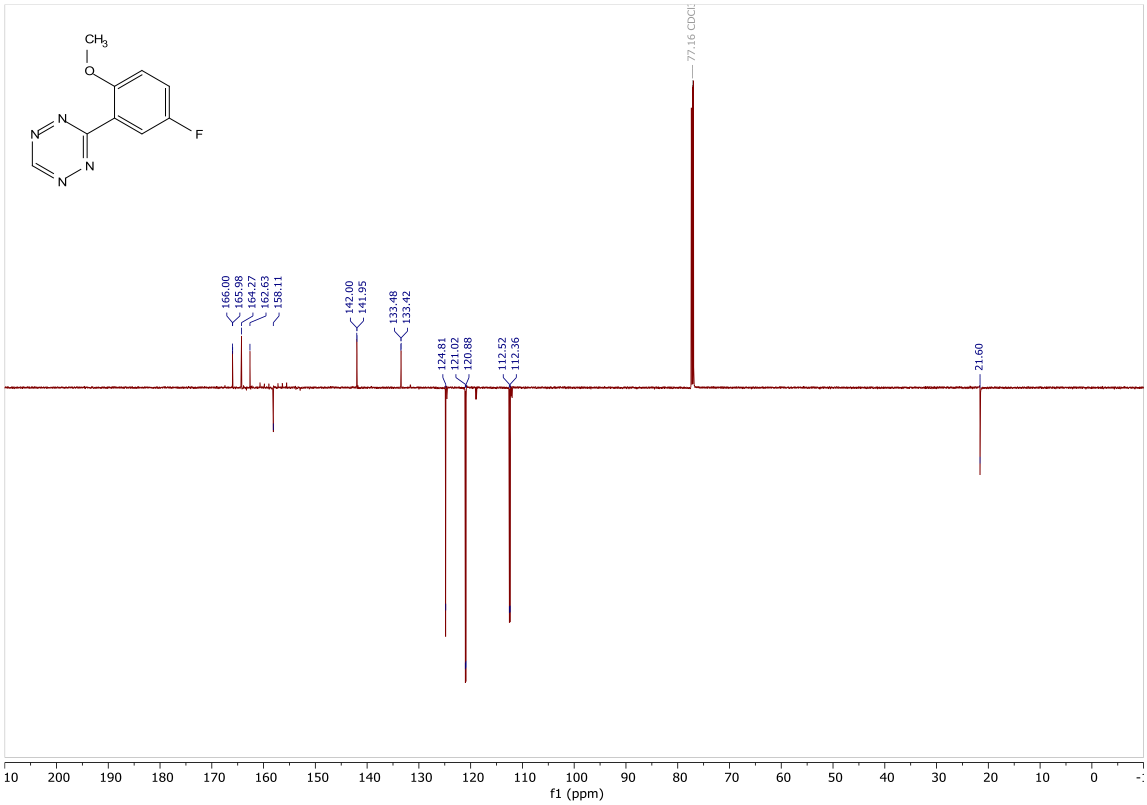

Analytical HPLC of 3-(3-fluoro-6-methoxyphenyl)-1,2,4,5-tetrazine (**15-*o***)

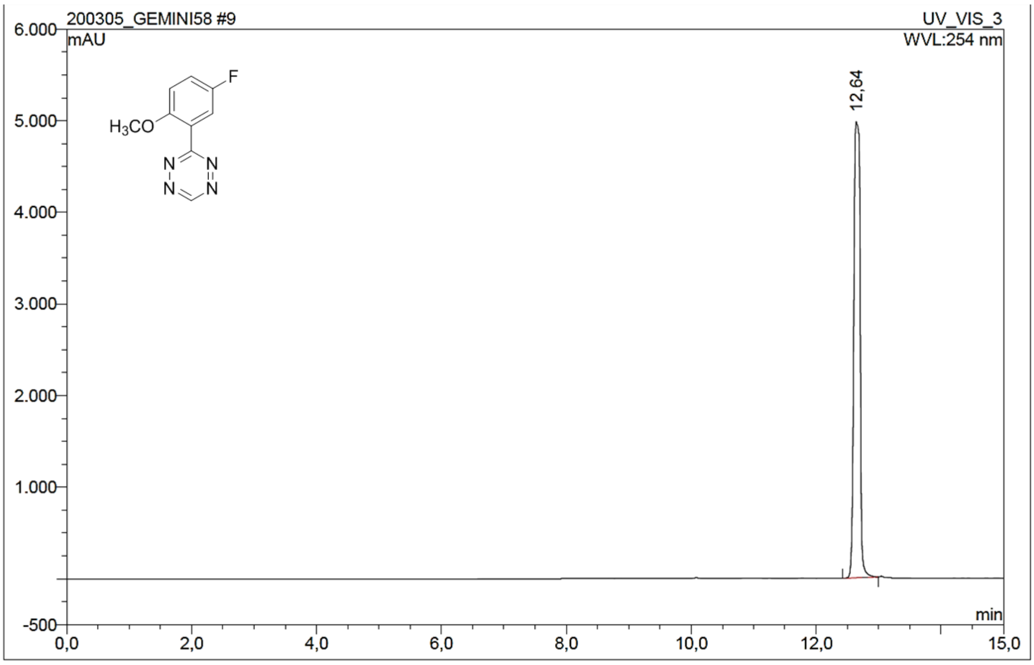

No.	Ret.Time min	Peak Name	Height mAU	Area mAU*min	Rel.Area %	Amount	Resolution(EP)
1	12,64	n.a.	4981,204	567,379	100,00	n.a.	n.a.
**Total:**			4981,204	567,379	100,00	0,000	

^{1}H NMR spectrum of 3-(5-iodo-2-methoxyphenyl)-1,2,4,5-tetrazine (**40a**) (400 MHz, $CDCl_3$)

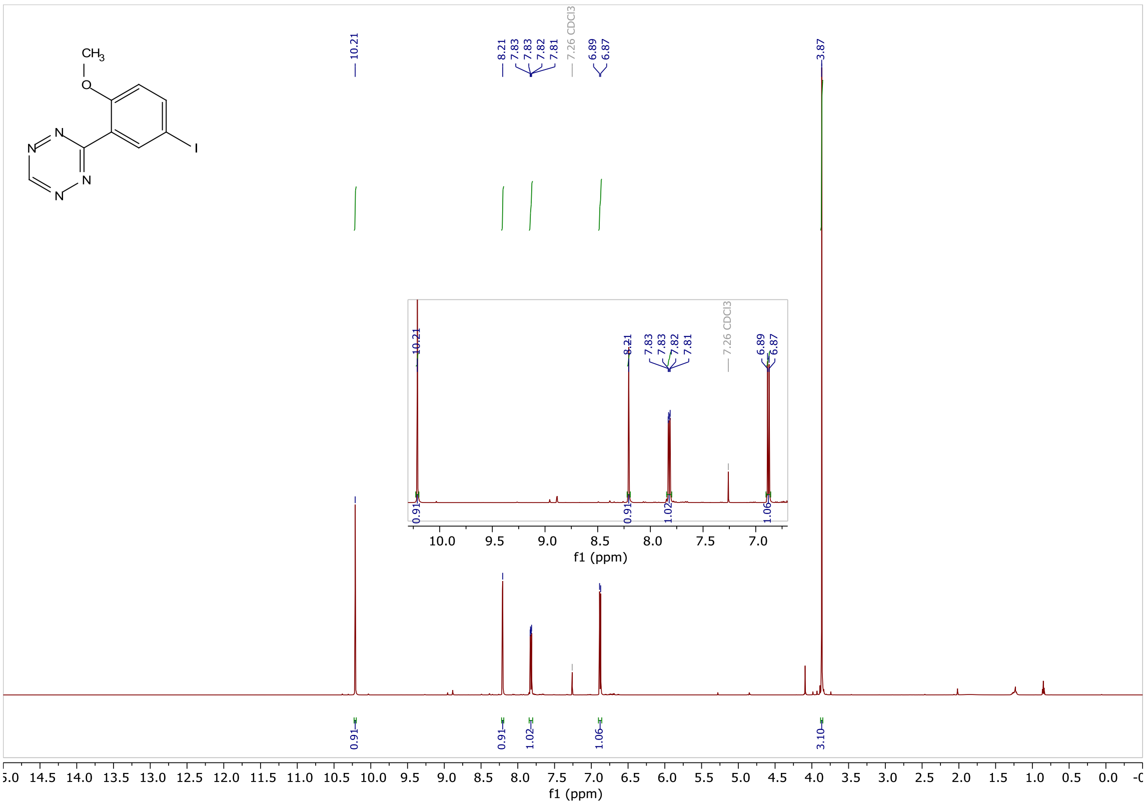

^{13}C NMR spectrum of 3-(5-iodo-2-methoxyphenyl)-1,2,4,5-tetrazine (**40a**) (101 MHz, CDCl3)

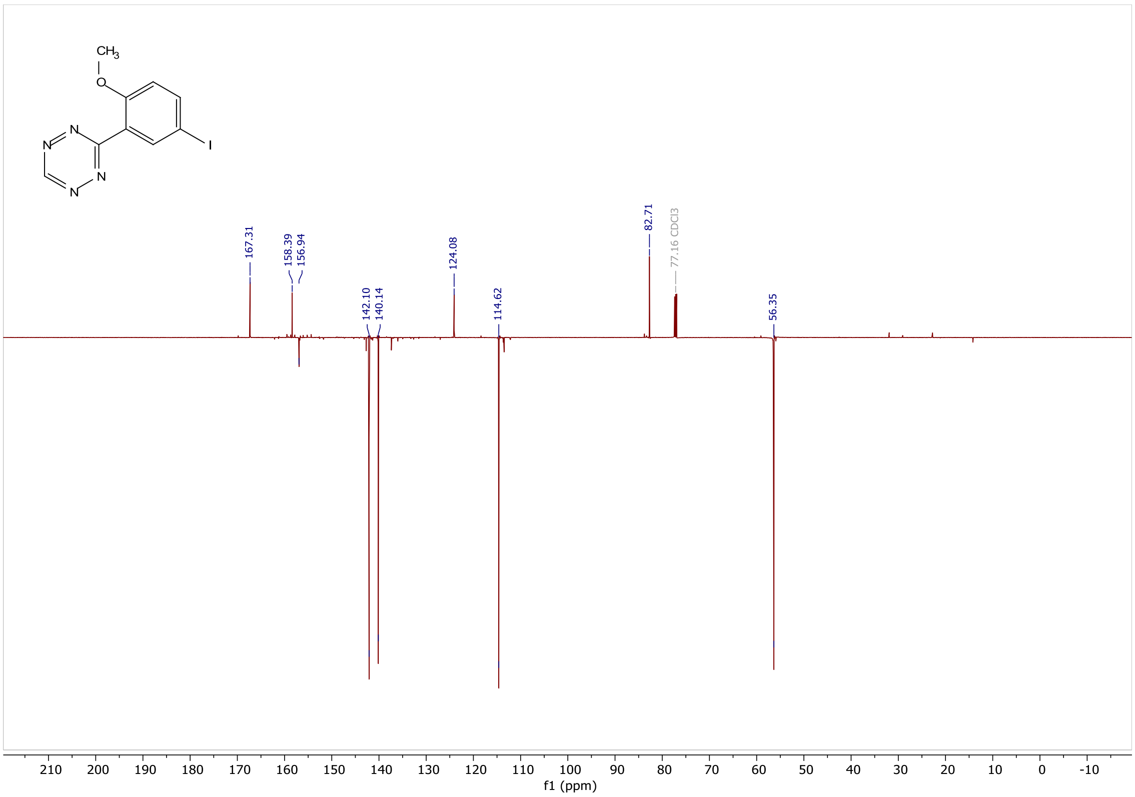

^{1}H NMR spectrum of N-(2-cyano-4-fluorophenyl)acetamide (**16a-*o***) (400 MHz, DMSO-d_6)

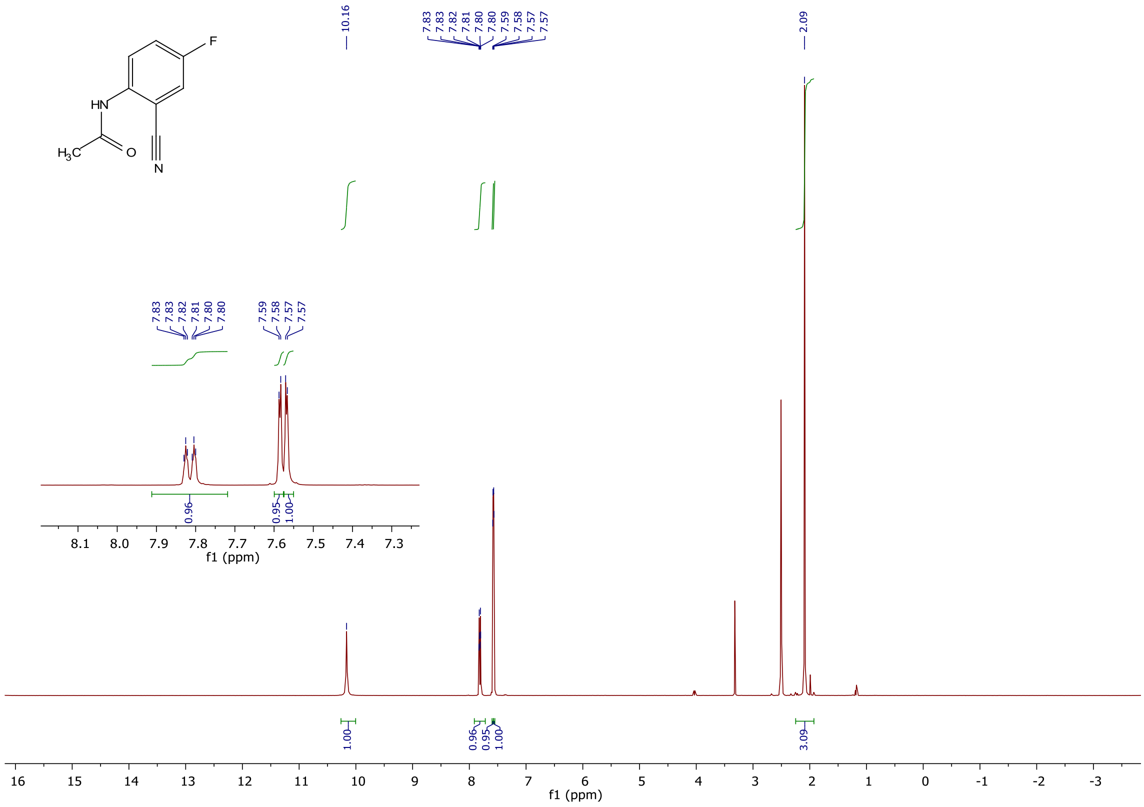

^{13}C NMR spectrum of N-(2-cyano-4-fluorophenyl)acetamide (**16a-*o***) (101 MHz, DMSO-d$_6$)

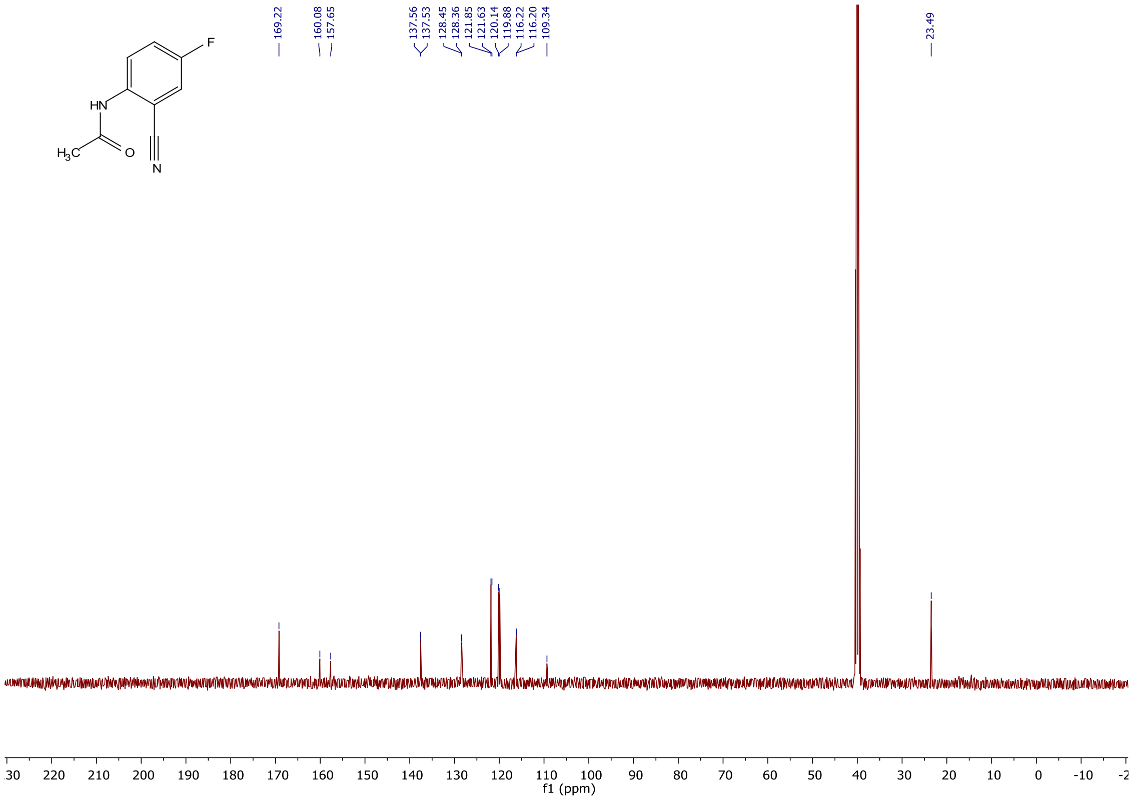

^{1}H NMR spectrum of 2-cyano-4-fluorobenzamide (**17a-*o***) (400 MHz, DMSO-d_6)

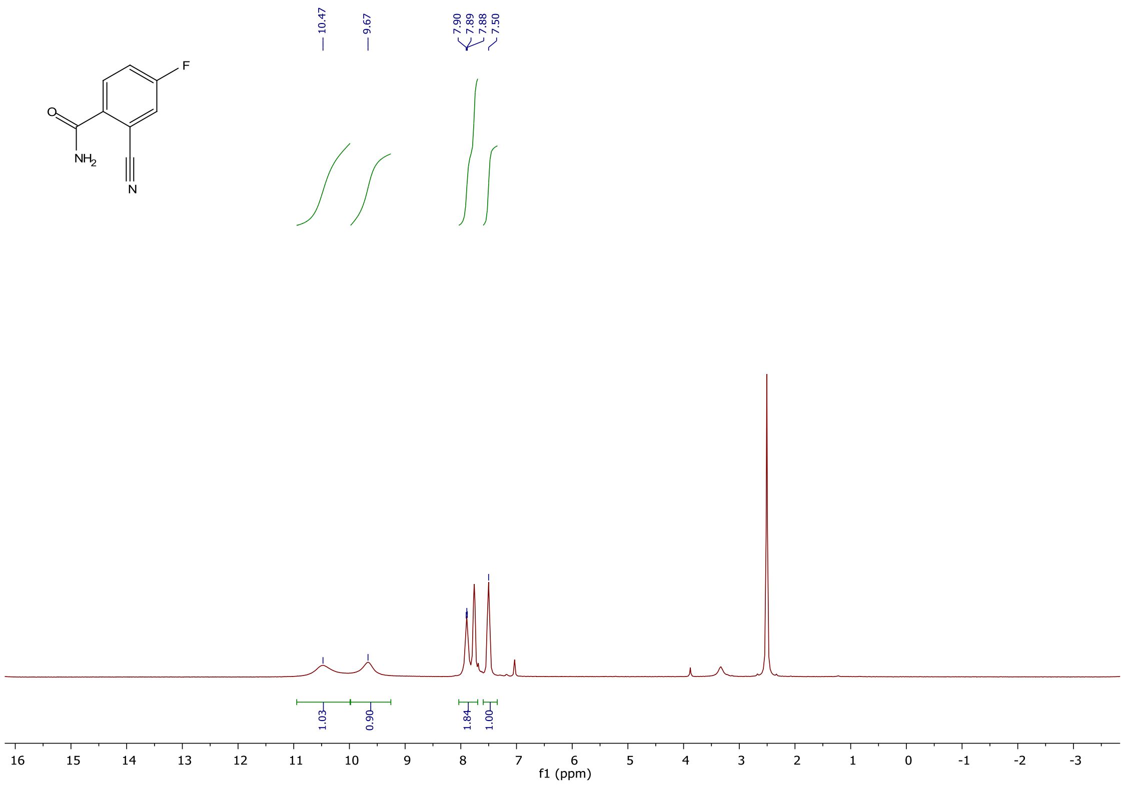

^{13}C NMR spectrum of 2-cyano-4-fluorobenzamide (**17a-*o***) (101 MHz, DMSO-d_6)

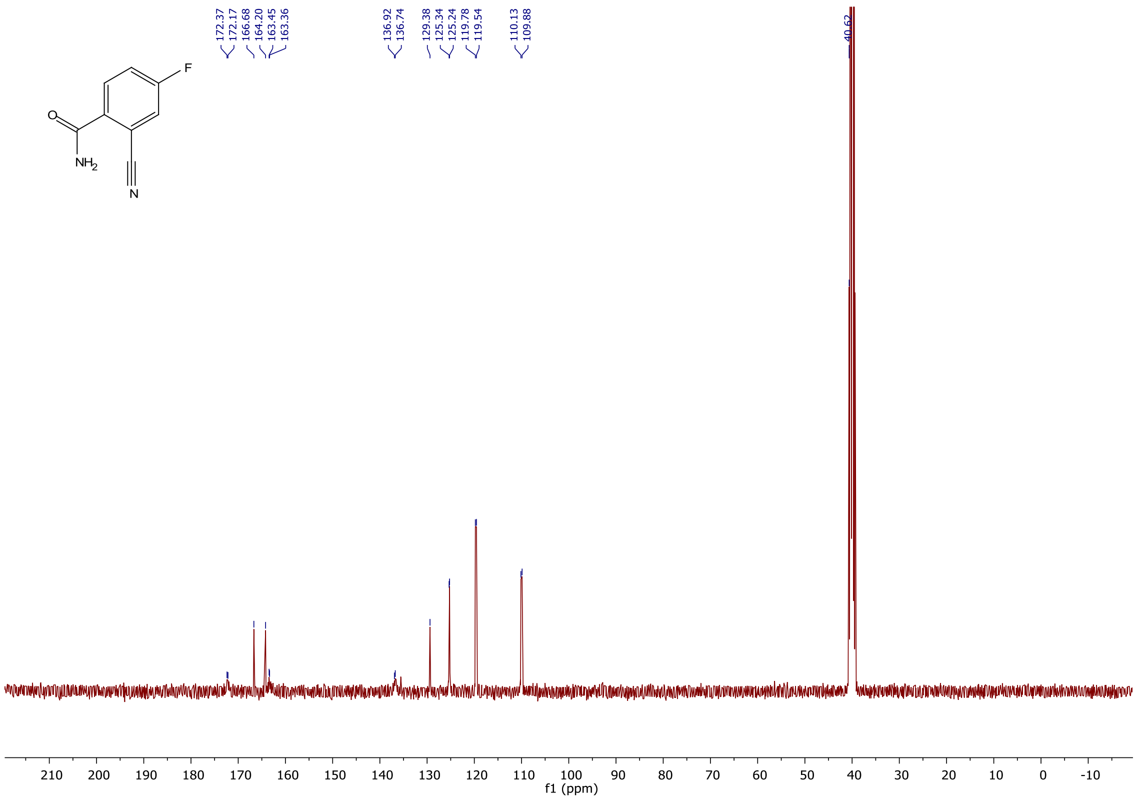

^{1}H NMR spectrum of 2-Cyano-4-fluoro-N-methylbenzamide (**18a-*o***) (400 MHz, DMSO-d_6)

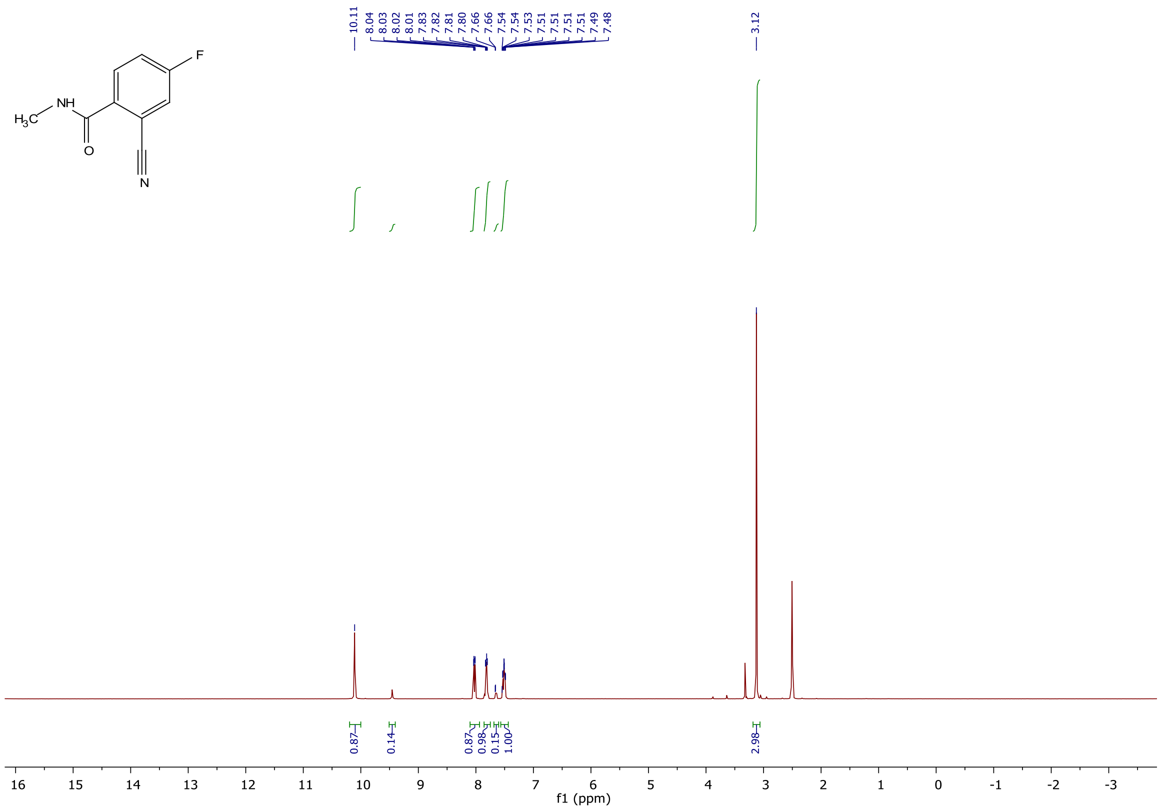

^{13}C NMR spectrum of 2-Cyano-4-fluoro-N-methylbenzamide (**18a-*o***) (101 MHz, DMSO-d_6)

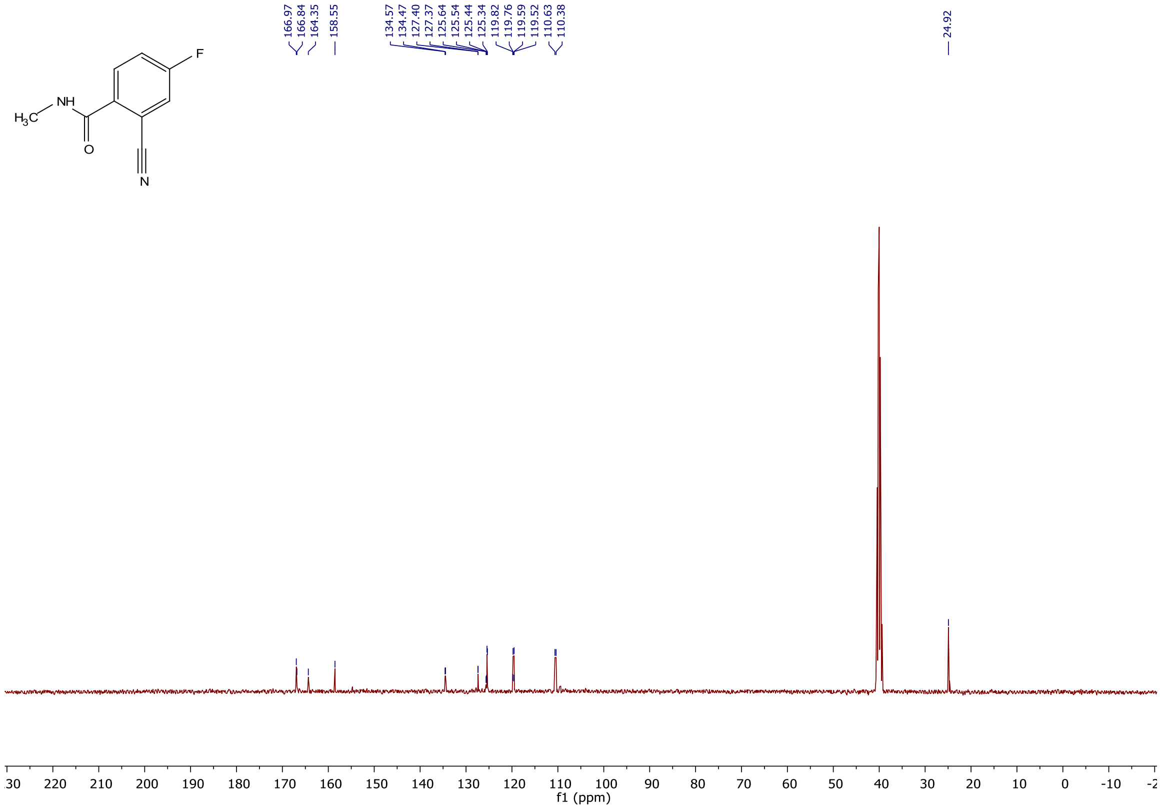

^{1}H NMR spectrum of 8-((4-(6-methyl-1,2,4,5-tetrazin-3-yl)phenyl)-l3-iodaneylidene)-6,10-dioxaspiro[4.5]decane-7,9-dione (**2**) (101 MHz, MeOD)

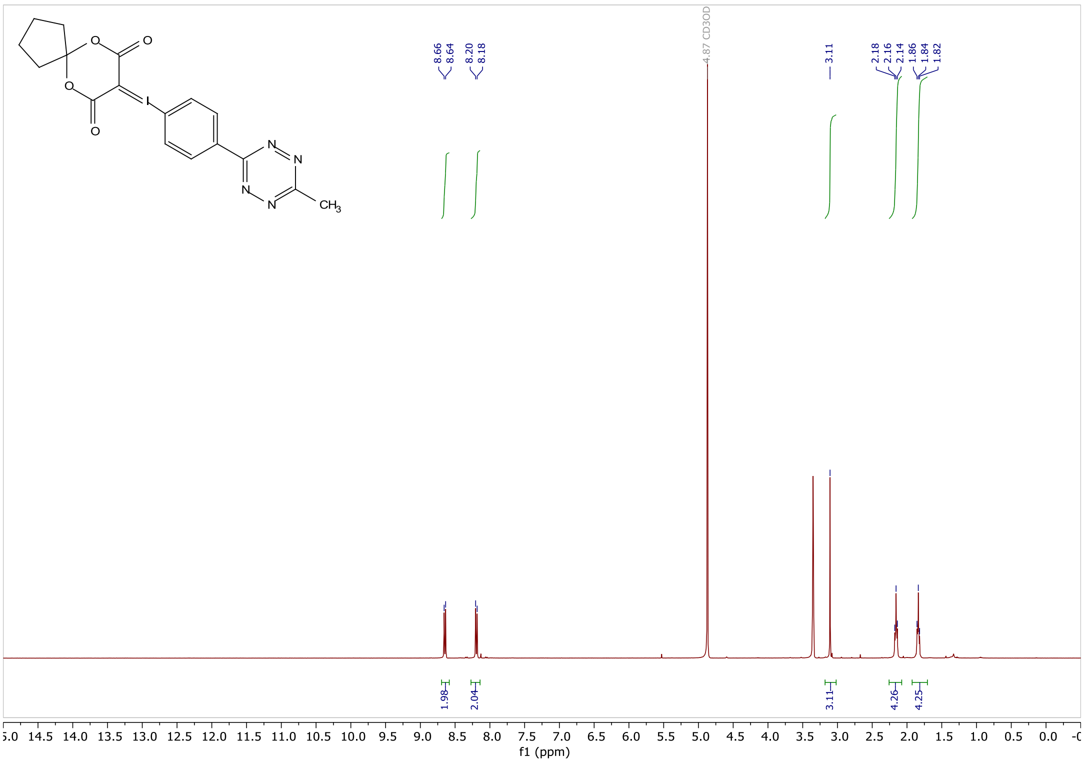

^{1}H NMR spectrum of 8-((4-(6-methyl-1,2,4,5-tetrazin-3-yl)phenyl)-l3-iodaneylidene)-6,10-dioxaspiro[4.5]decane-7,9-dione (**2**) (600 MHz, $CDCl_3$)

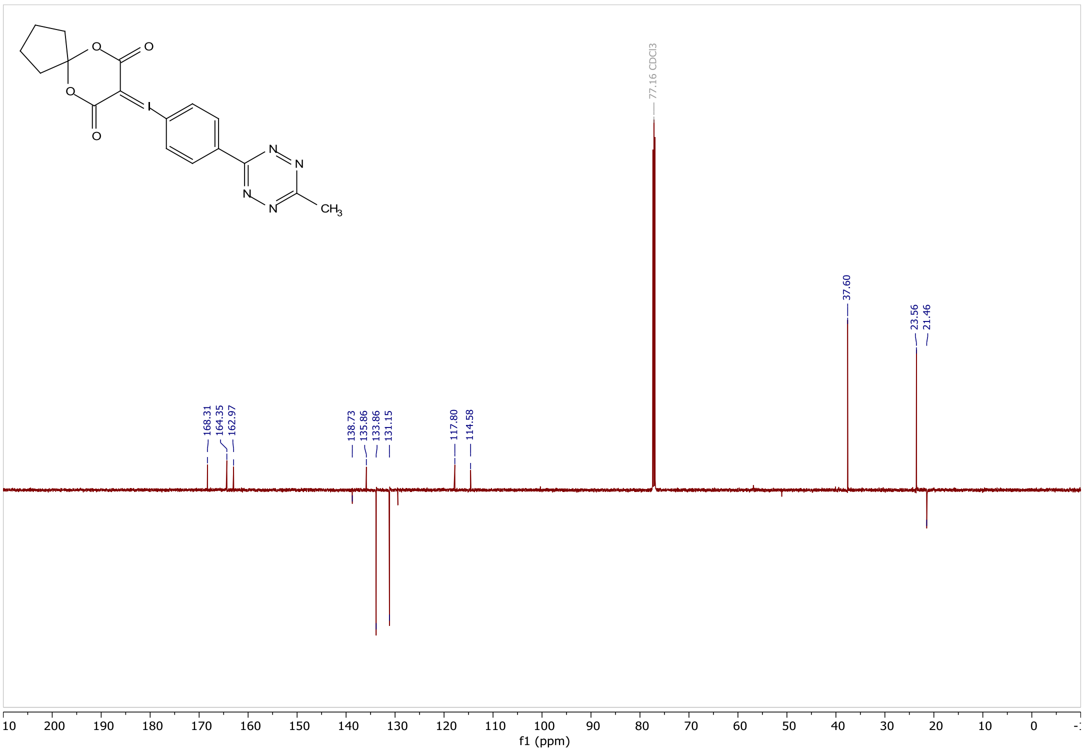

^{1}H NMR spectrum of mesityl(4-(6-methyl-1,2,4,5-tetrazin-3-yl)phenyl)iodonium (**5**) (600 MHz, MeOD)

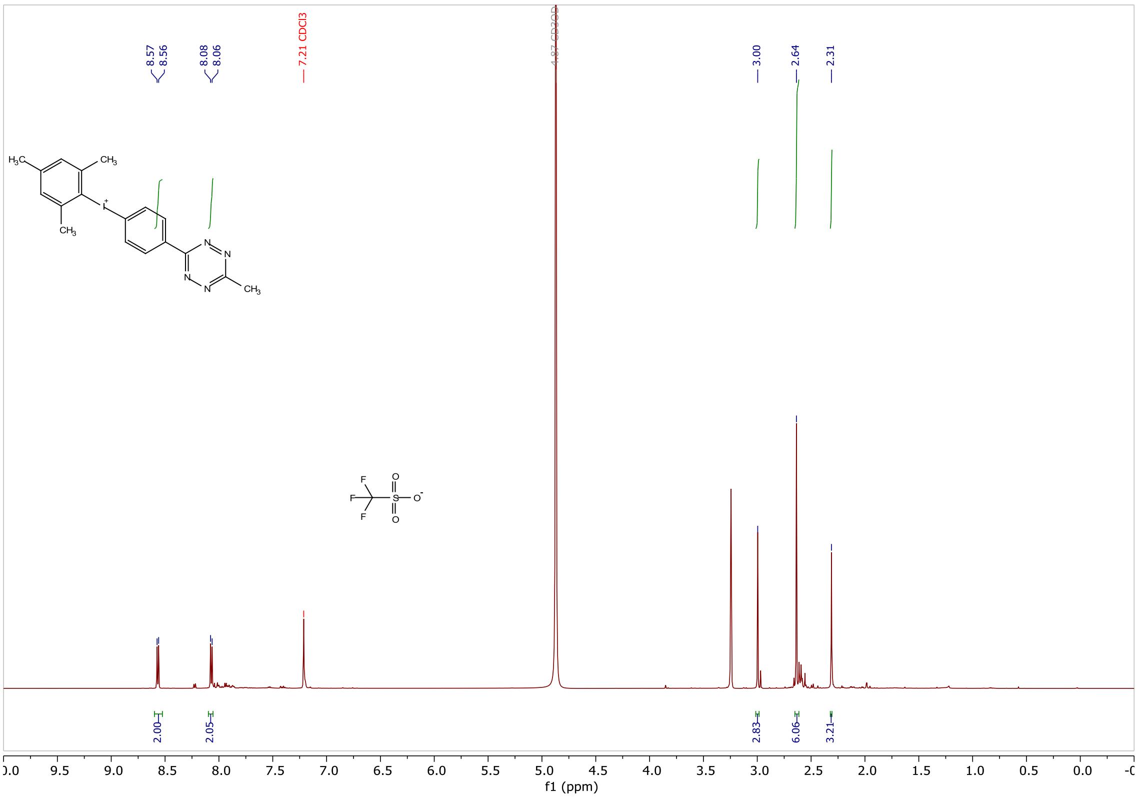

^{1}H NMR spectrum of 3-methyl-6-(4-(4,4,5,5-tetramethyl-1,3,2-dioxaborolan-2-yl)phenyl)-1,2,4,5-tetrazine (**3a**) (400 MHz, CDCl3)

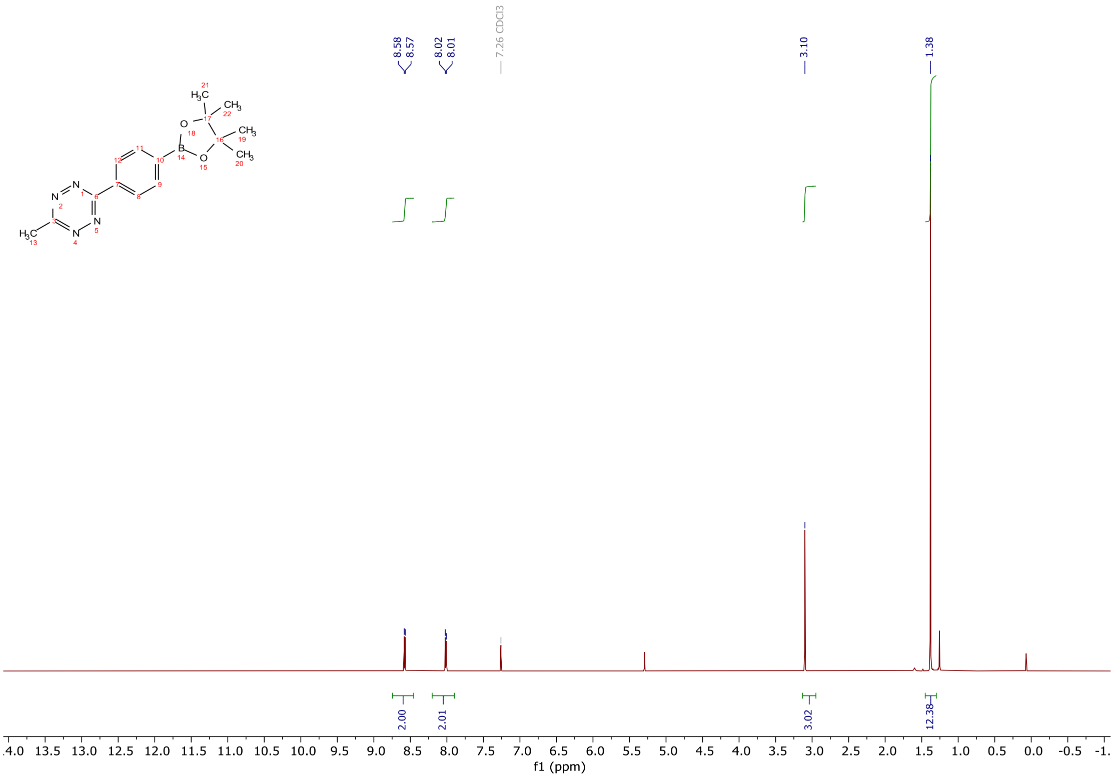

^{13}C NMR spectrum of 3-methyl-6-(4-(4,4,5,5-tetramethyl-1,3,2-dioxaborolan-2-yl)phenyl)-1,2,4,5-tetrazine (**3a**) (400 MHz, CDCl3)

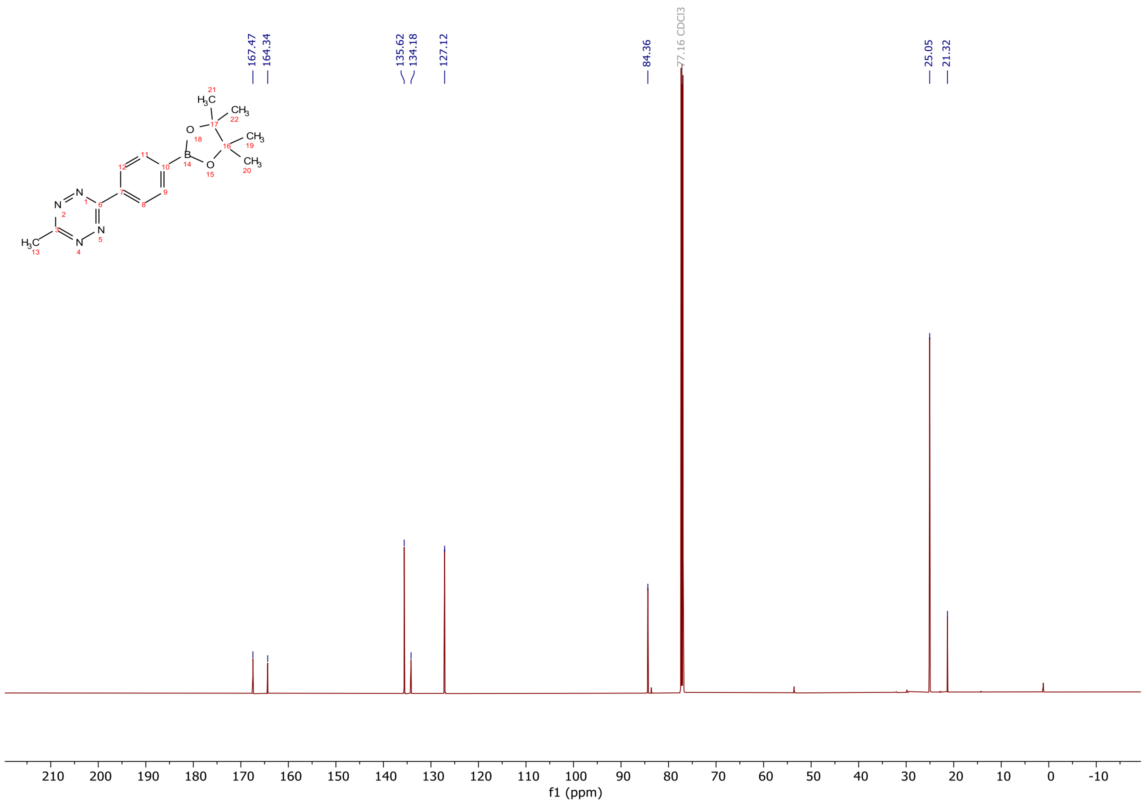

^{1}H NMR spectrum of 3-methyl-6-(4-(trimethylstannyl)phenyl)-1,2,4,5-tetrazine (**3**) (400 MHz, CDCl3)

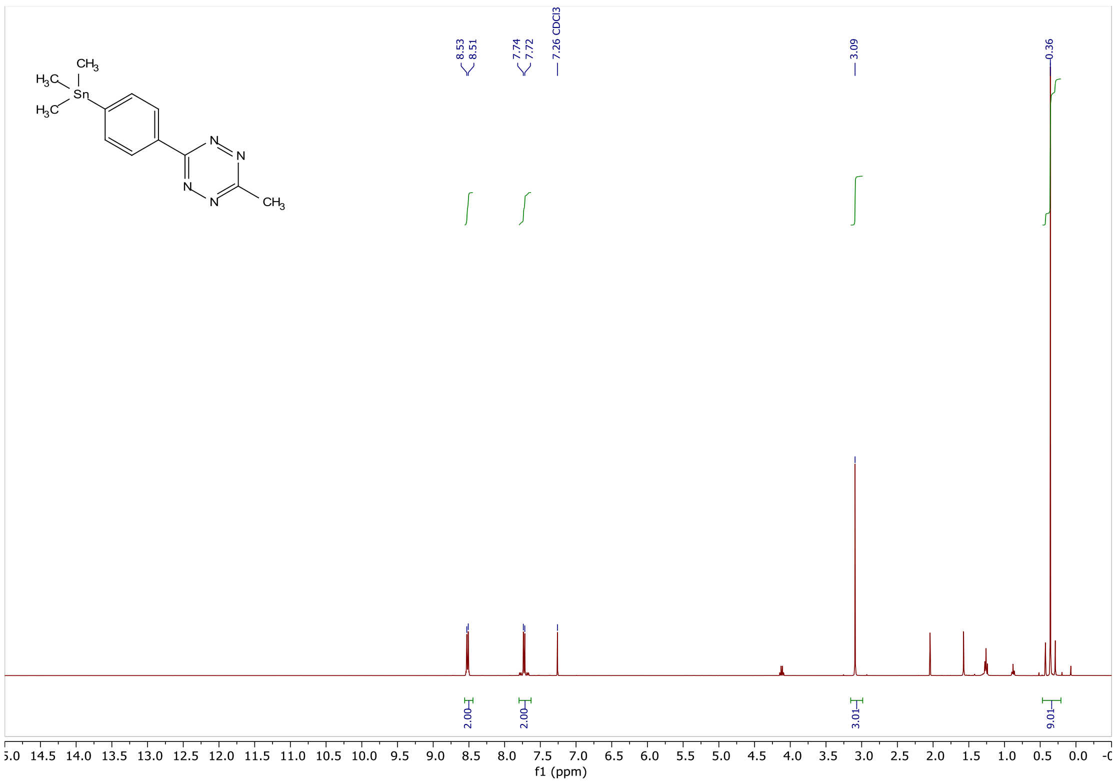

^{13}C NMR spectrum of 3-methyl-6-(4-(trimethylstannyl)phenyl)-1,2,4,5-tetrazine (**3**) (101 MHz, CDCl3)

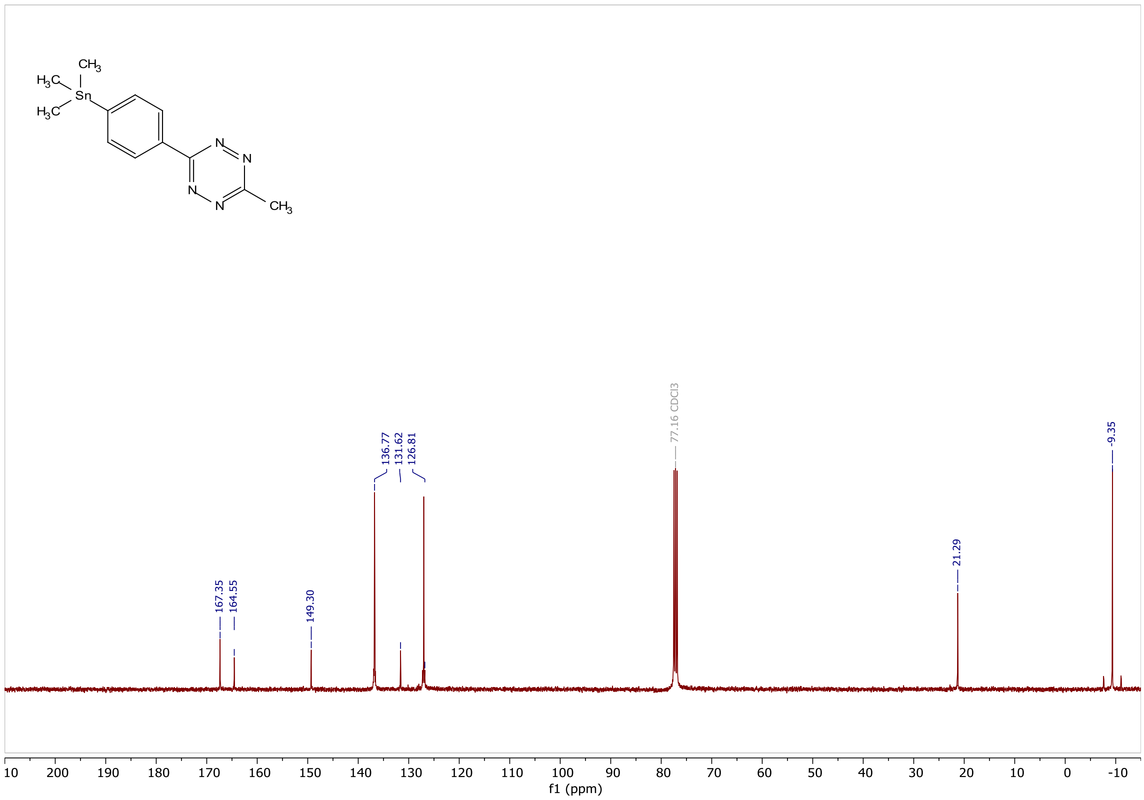

Analytical HPLC of 3-methyl-6-(4-(trimethylstannyl)phenyl)-1,2,4,5-tetrazine (**3**)

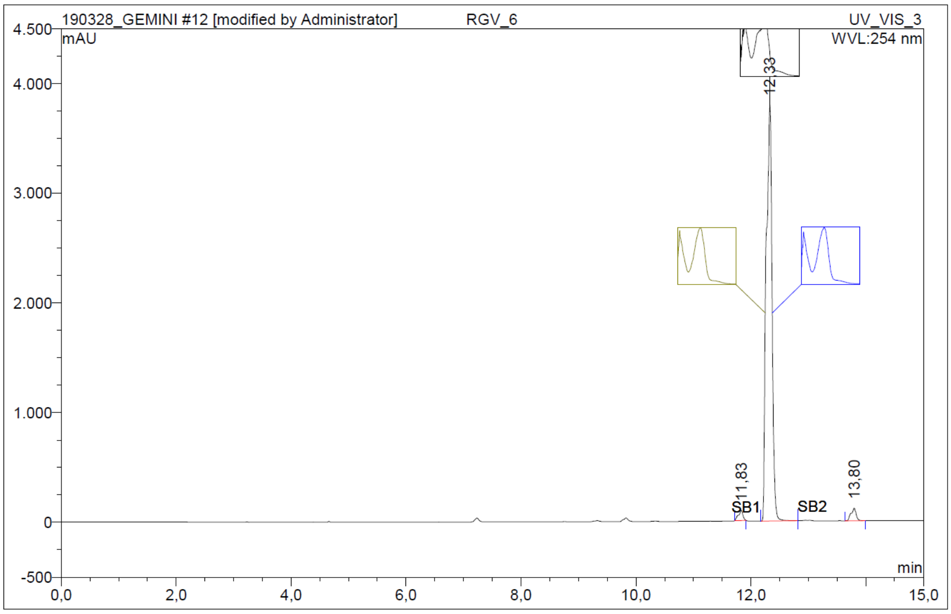

No.	Ret.Time min	Peak Name	Height mAU	Area mAU*min	Rel.Area %	Amount	Resolution(EP)
1	11,83	n.a.	92,280	8,450	1,82	n.a.	2,69
2	12,33	n.a.	3797,252	443,585	95,71	n.a.	7,58
3	13,80	n.a.	115,026	11,416	2,46	n.a.	n.a.
Total:			4004,558	463,450	100,00	0,000	

^{1}H NMR spectrum of 3-(3-trimethyltin)-6-methyl-1,2,4,5-tetrazine (**22**) (400 MHz, $CDCl_3$)

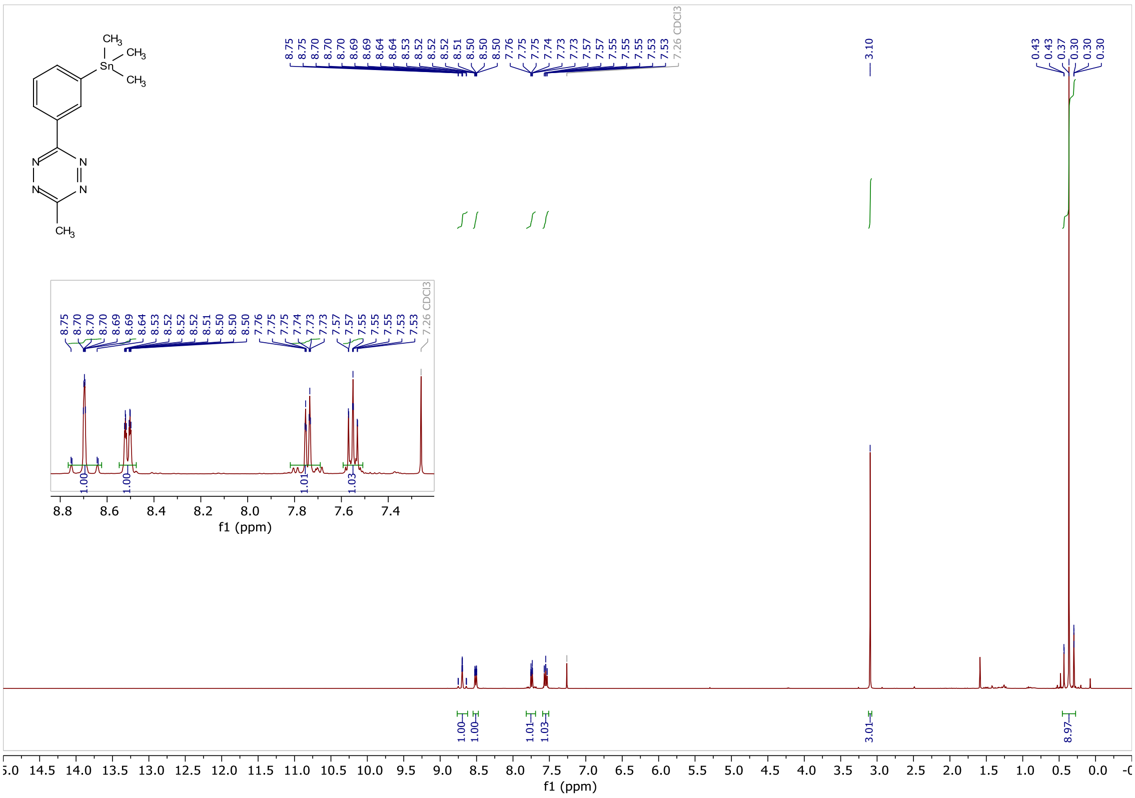

^{13}C NMR spectrum of 3-(3-trimethyltin)-6-methyl-1,2,4,5-tetrazine (**22**) (101 MHz, CDCl3)

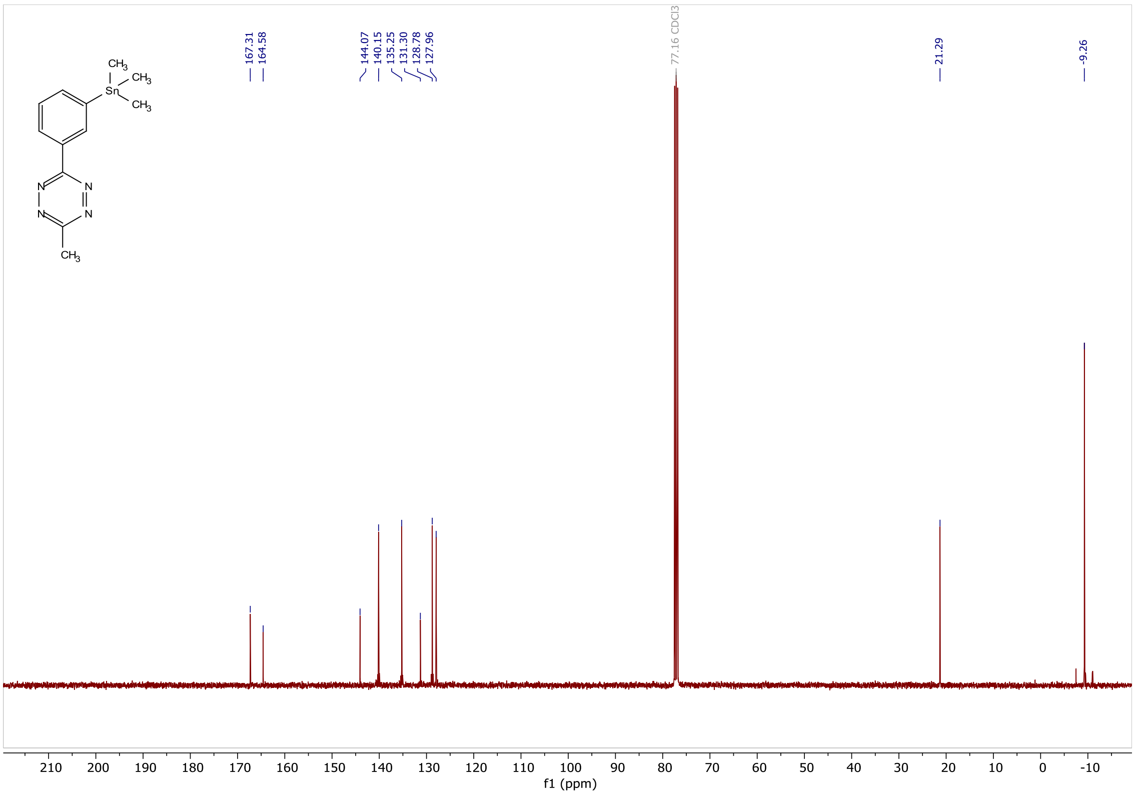

Analytical HPLC of 3-(3-trimethyltin)-6-methyl-1,2,4,5-tetrazine (**22**)

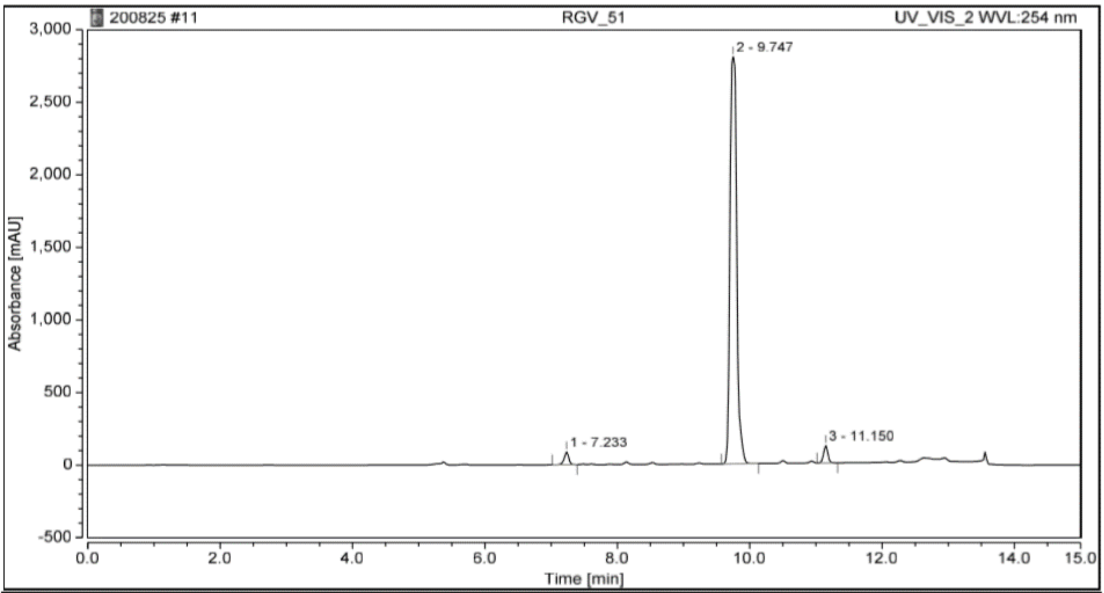

Integration Results

No.	Peak Name	Retention Time min	Area mAU*min	Height mAU	Relative Area %	Relative Height %	Amount
n.a.	Tosylate	n.a.	n.a.	n.a.	n.a.	n.a.	n.a.
n.a.	cold fallyprode	n.a.	n.a.	n.a.	n.a.	n.a.	n.a.
n.a.	18F-fallypride	n.a.	n.a.	n.a.	n.a.	n.a.	n.a.
1		7.233	7.012	89.097	2.04	2.96	n.a.
2		9.747	327.820	2804.417	95.31	93.02	n.a.
3		11.150	9.133	121.327	2.66	4.02	n.a.
**Total:**			**343.964**	**3014.840**	**100.00**	**100.00**	

¹H NMR spectrum of 3-(4-trimethyltin)-6-methyl-1,2,4,5-tetrazine (**27**) (400 MHz, CDCl3)

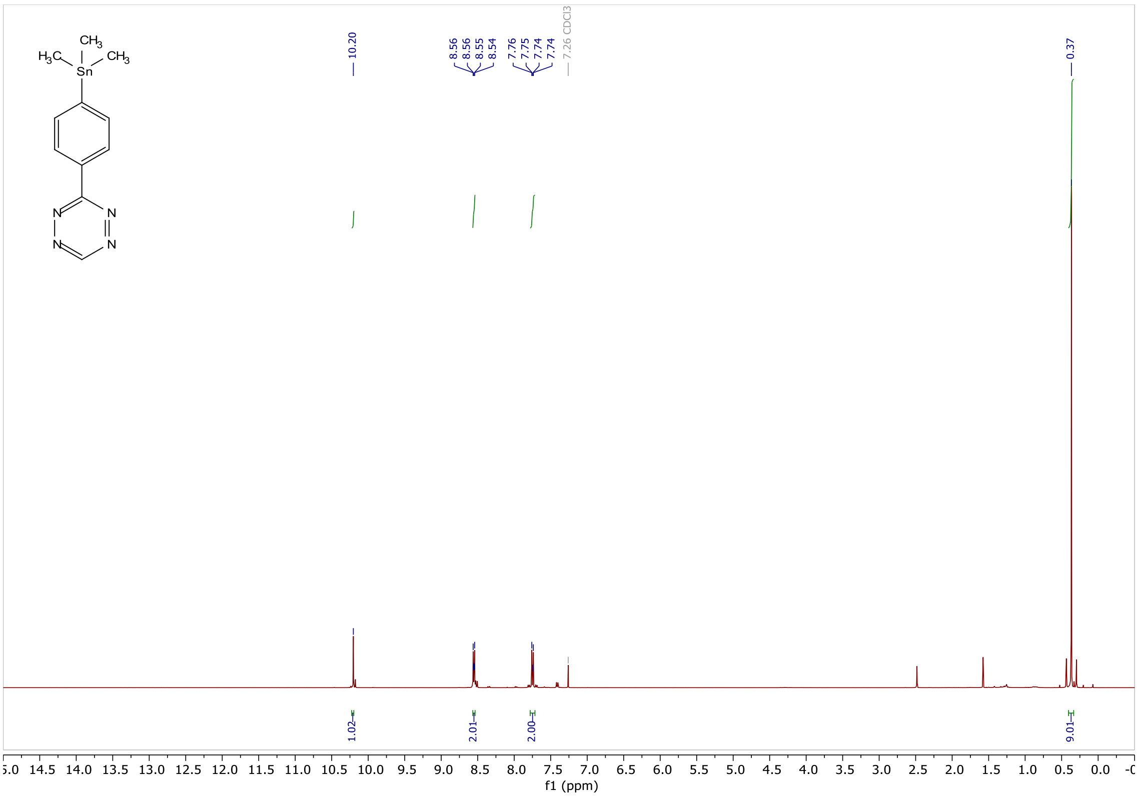

¹³C NMR spectrum of 3-(4-trimethyltin)-6-methyl-1,2,4,5-tetrazine (**27**) (101 MHz, CDCl3)

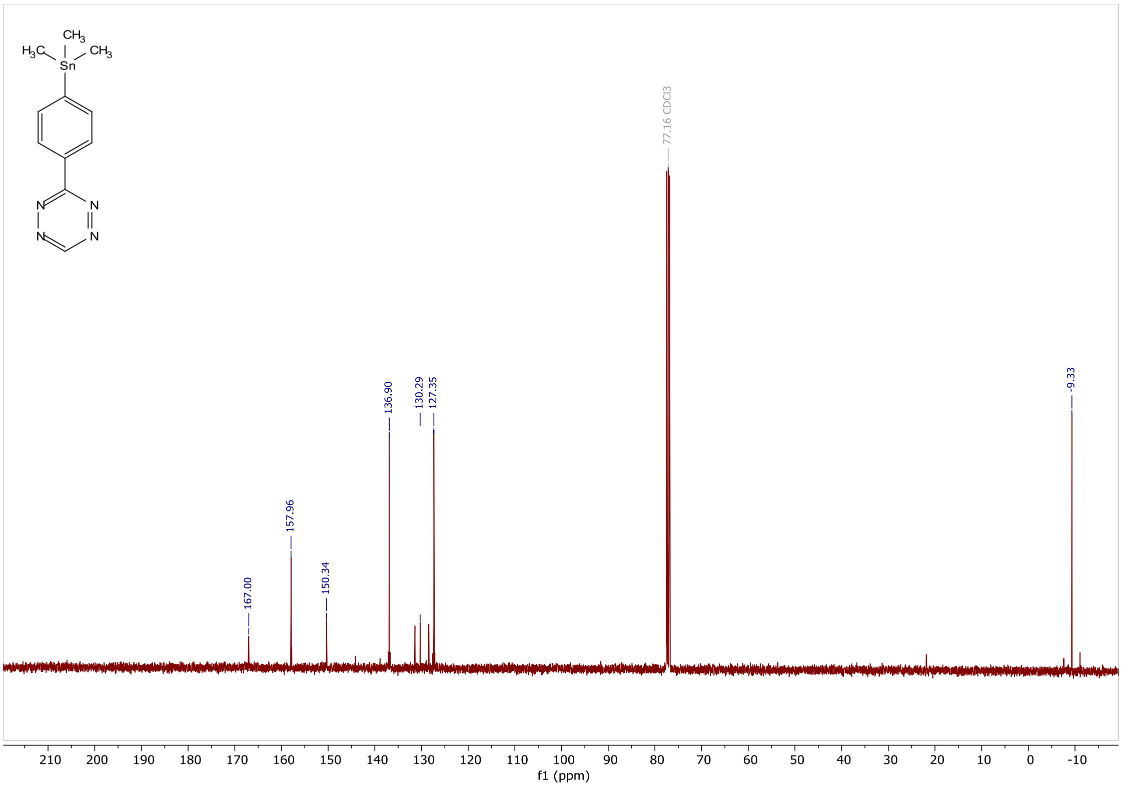

Analytical HPLC of 3-(4-trimethyltin)-6-methyl-1,2,4,5-tetrazine (**27**)

Chromatogram

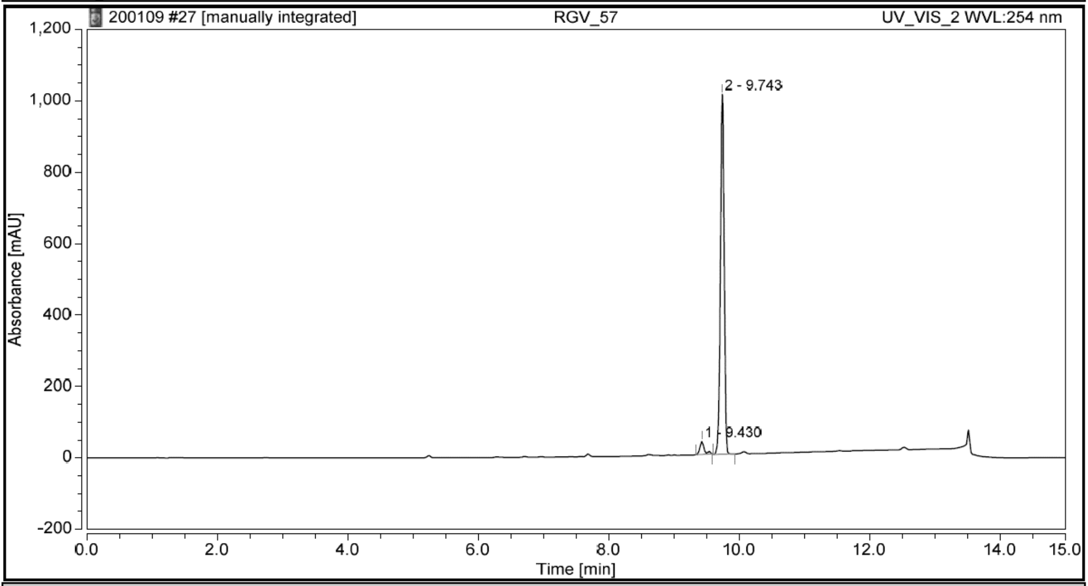

Integration Results

No.	Peak Name	Retention Time min	Area mAU*min	Height mAU	Relative Area %	Relative Height %	Amount
n.a.	Tosylate	n.a.	n.a.	n.a.	n.a.	n.a.	n.a.
n.a.	cold fallyprode	n.a.	n.a.	n.a.	n.a.	n.a.	n.a.
n.a.	18F-fallypride	n.a.	n.a.	n.a.	n.a.	n.a.	n.a.
1		9.430	2.849	36.197	4.12	3.47	n.a.
2		9.743	66.279	1008.018	95.88	96.53	n.a.
**Total:**			**69.128**	**1044.215**	**100.00**	**100.00**	

^{1}H NMR spectrum of 3-(3-trimethyltin)-1,2,4,5-tetrazine (**28**) (400 MHz, $CDCl_3$)

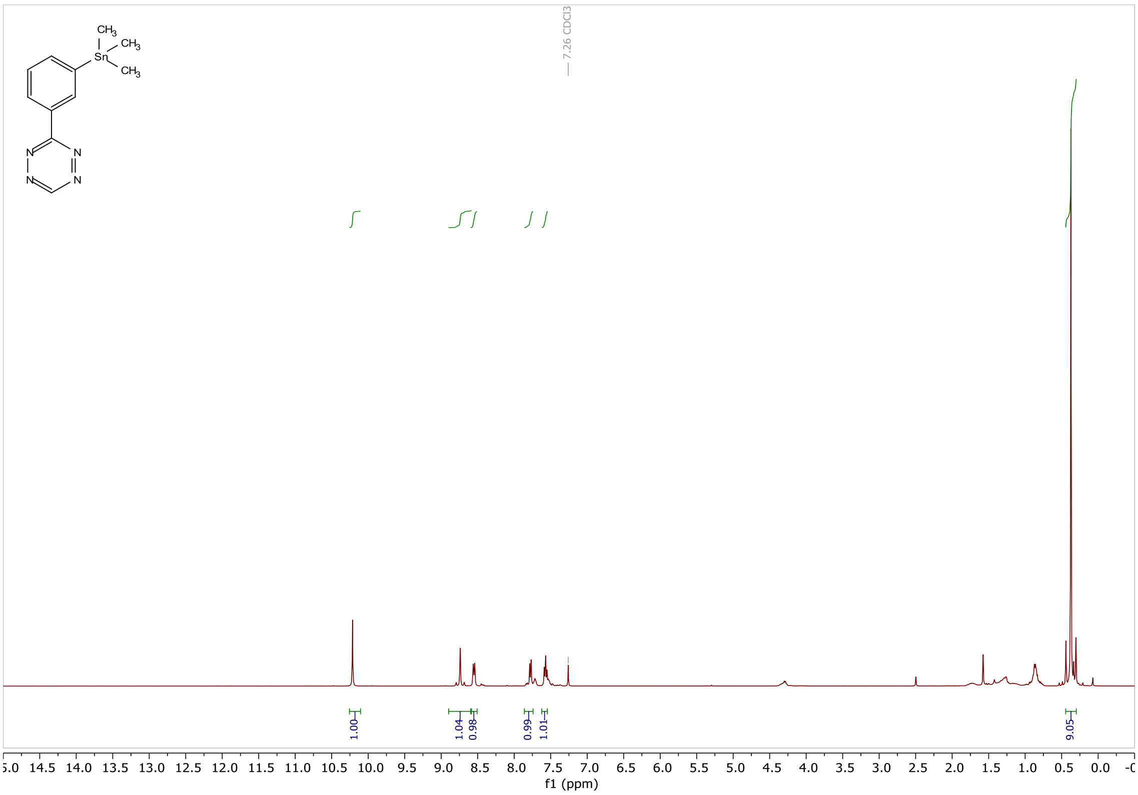

^{13}C NMR spectrum of 3-(3-trimethyltin)-1,2,4,5-tetrazine (**28**) (101 MHz, CDCl3)

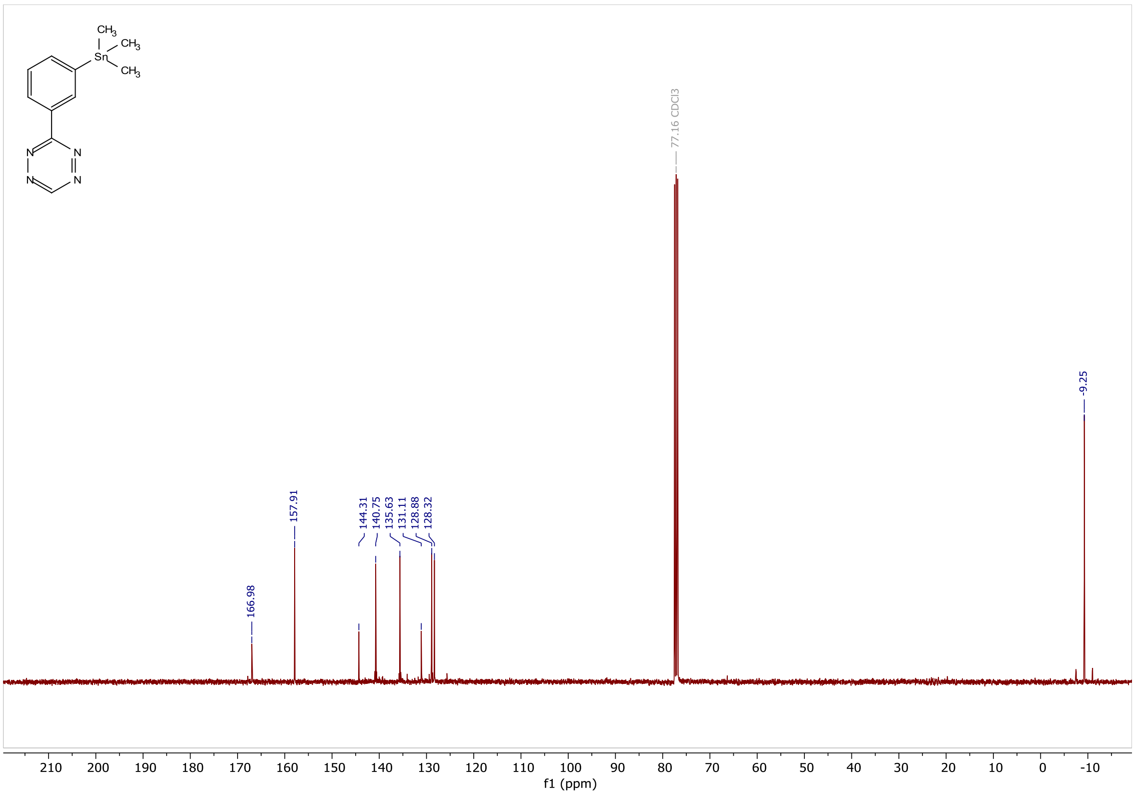

Analytical HPLC of 3-(3-trimethyltin)-1,2,4,5-tetrazine (**28**)

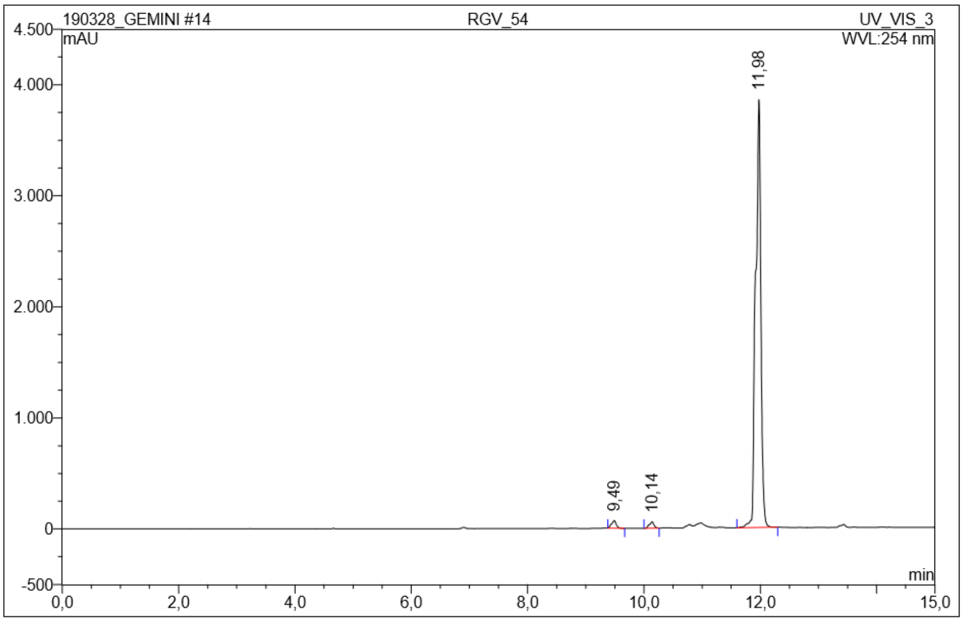

No.	Ret.Time min	Peak Name	Height mAU	Area mAU*min	Rel.Area %	Amount	Resolution(EP)
1	9,49	n.a.	68,894	6,312	1,53	n.a.	4,23
2	10,14	n.a.	57,605	5,308	1,29	n.a.	10,74
3	11,98	n.a.	3854,578	401,400	97,19	n.a.	n.a.
**Total:**			3981,078	413,020	100,00	0,000	

^{1}H NMR spectrum of 3-(4-methoxy-3-(trimethylstannyl)phenyl)-1,2,4,5-tetrazine (**30**) (400 MHz, CDCl3)

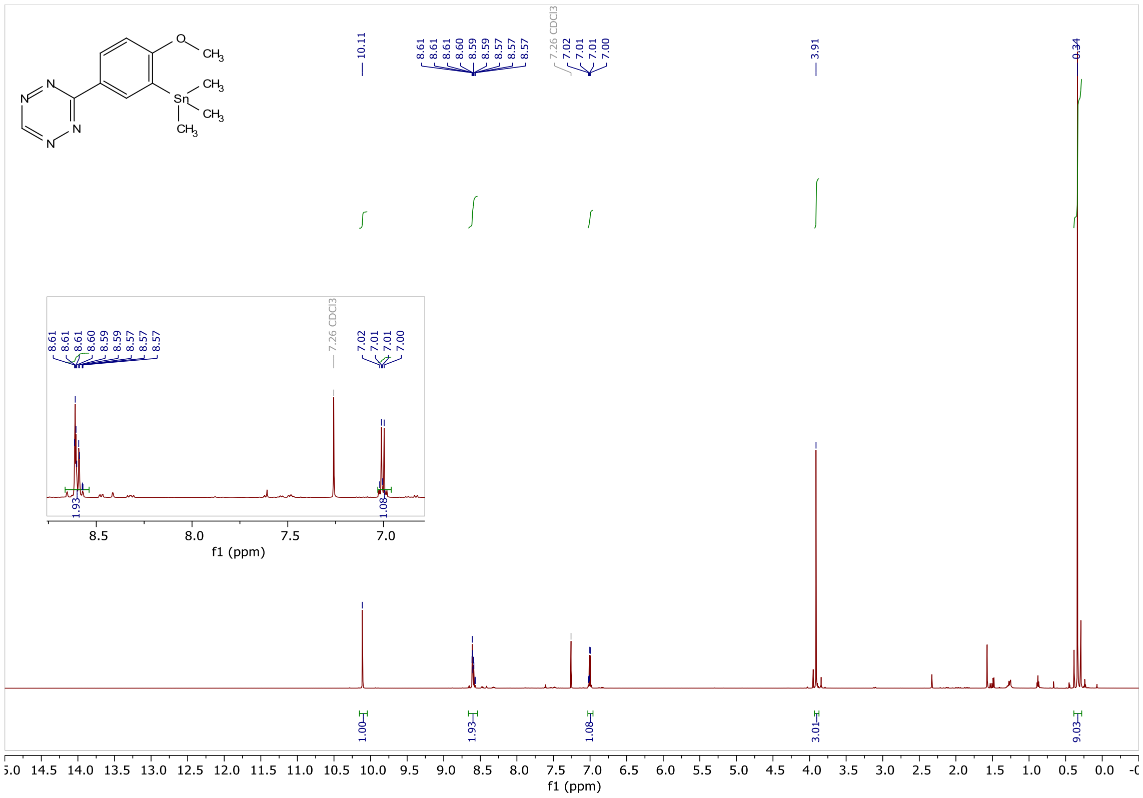

^{13}C NMR spectrum of 3-(4-methoxy-3-(trimethylstannyl)phenyl)-1,2,4,5-tetrazine (**30**) (101 MHz, CDCl3)

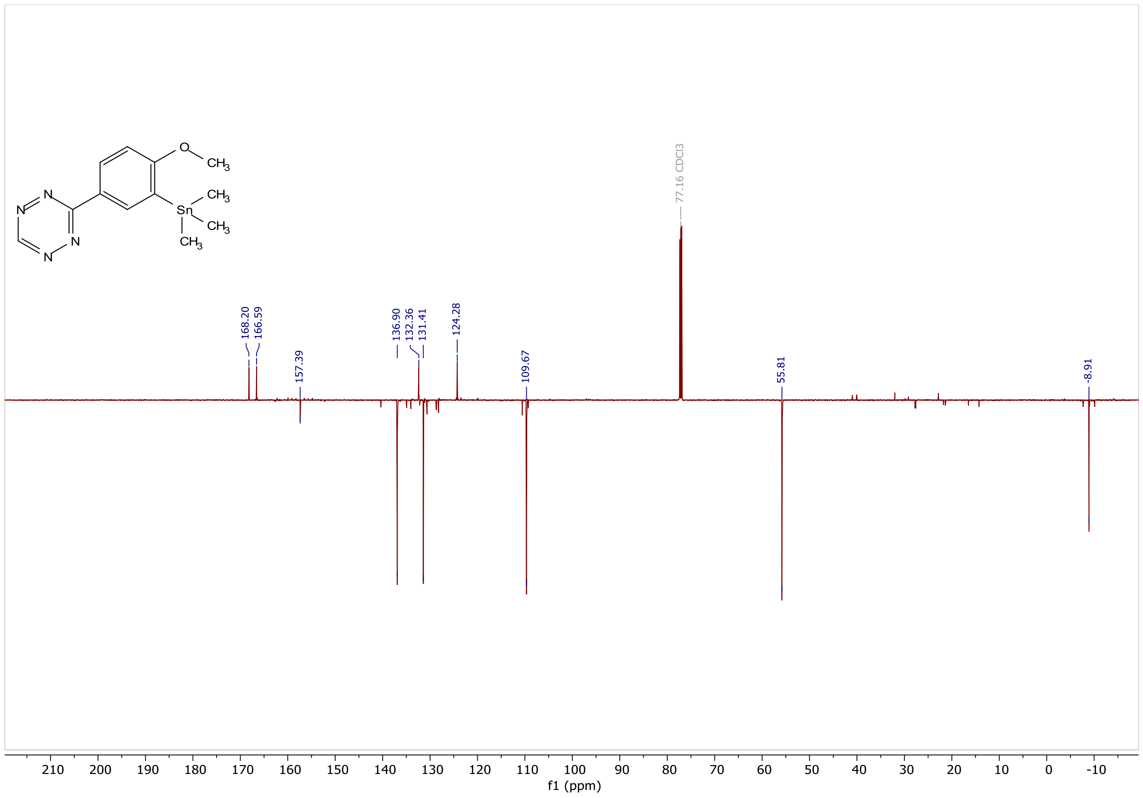

Analytical HPLC of 3-(4-methoxy-3-(trimethylstannyl)phenyl)-1,2,4,5-tetrazine (**30**)

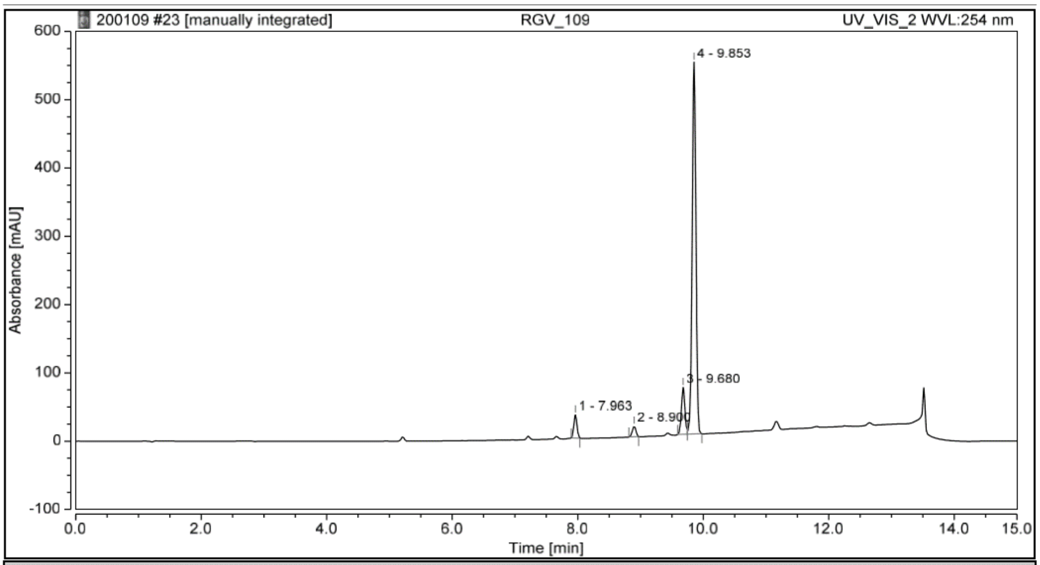

Integration Results							
No.	Peak Name	Retention Time min	Area mAU*min	Height mAU	Relative Area %	Relative Height %	Amount
n.a.	Tosylate	n.a.	n.a.	n.a.	n.a.	n.a.	n.a.
n.a.	cold fallyprode	n.a.	n.a.	n.a.	n.a.	n.a.	n.a.
n.a.	18F-fallypride	n.a.	n.a.	n.a.	n.a.	n.a.	n.a.
1		7.963	1.995	34.093	4.42	5.14	n.a.
2		8.900	1.007	15.749	2.23	2.38	n.a.
3		9.680	4.477	68.444	9.92	10.33	n.a.
4		9.853	37.662	544.488	83.43	82.15	n.a.
**Total:**			**45.140**	**662.774**	**100.00**	**100.00**	

^{1}H NMR spectrum of 3-(2-methoxy-5-(trimethylstannyl)phenyl)-1,2,4,5-tetrazine (**40**) (600 MHz, $CDCl_3$)

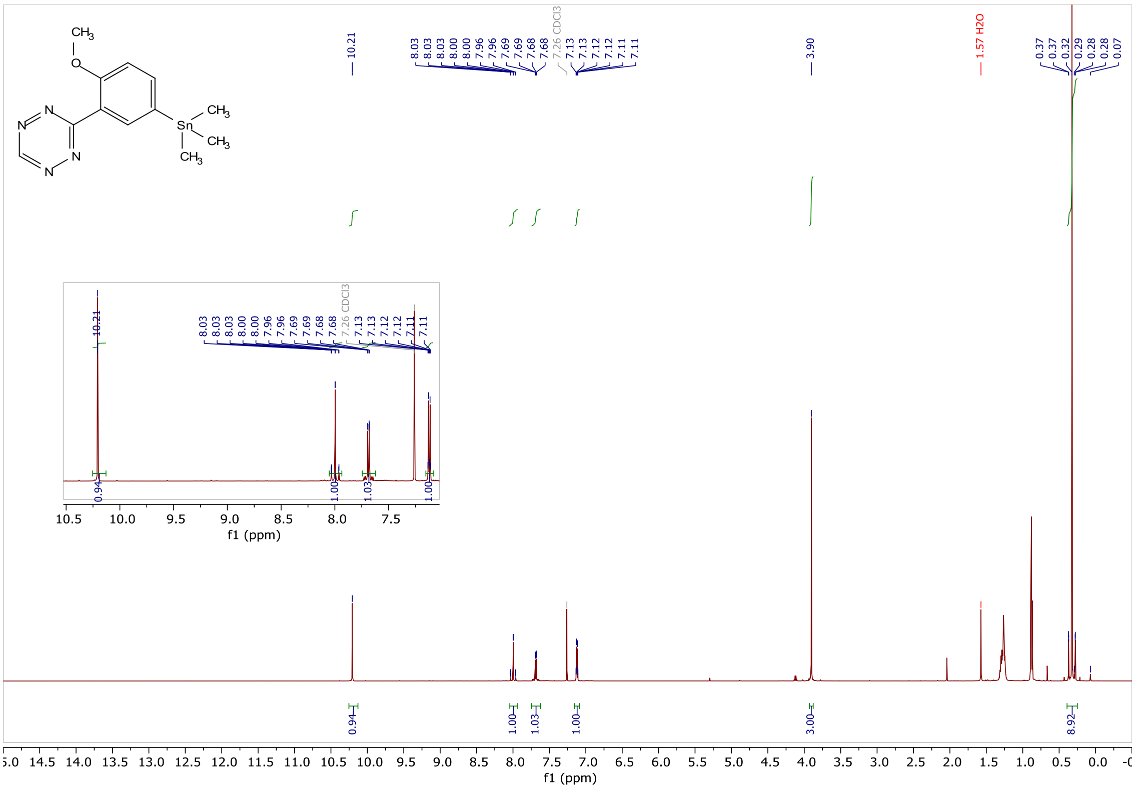

^{13}C NMR spectrum of 3-(2-methoxy-5-(trimethylstannyl)phenyl)-1,2,4,5-tetrazine (**40**) (151 MHz, CDCl3)

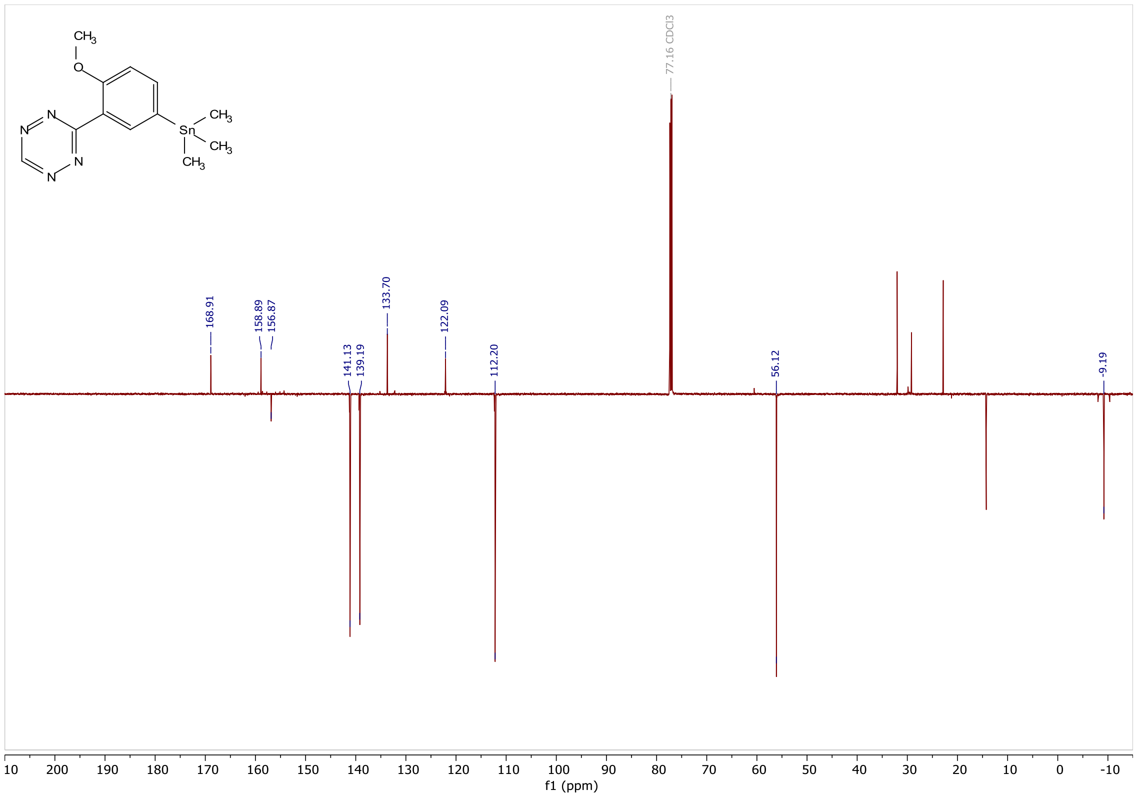

Analytical HPLC of 3-(2-methoxy-5-(trimethylstannyl)phenyl)-1,2,4,5-tetrazine (**40**)

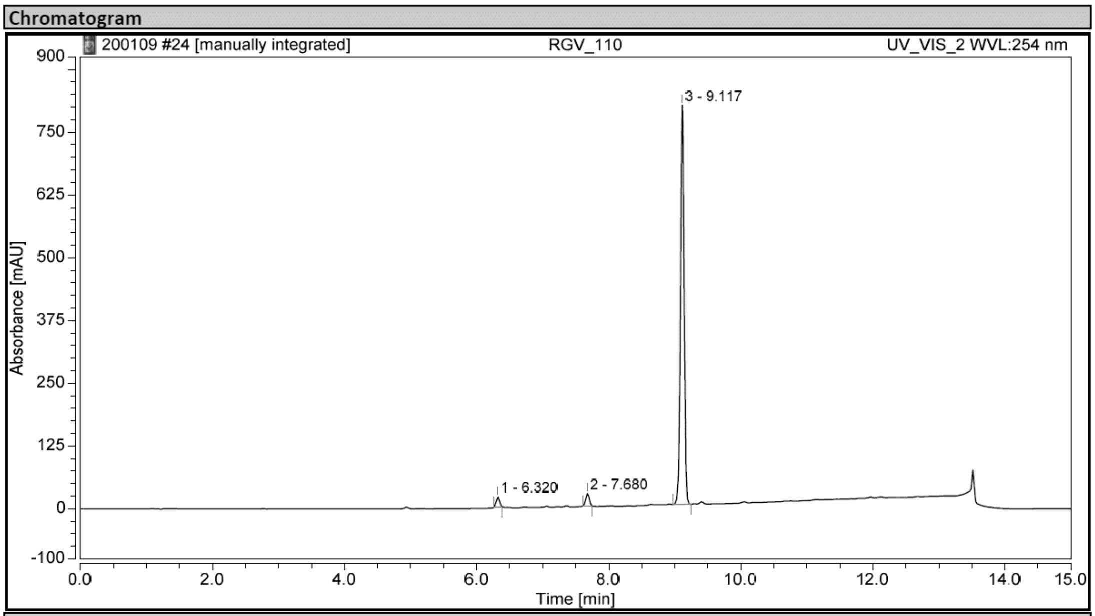

Integration Results

No.	Peak Name	Retention Time min	Area mAU*min	Height mAU	Relative Area %	Relative Height %	Amount
n.a.	Tosylate	n.a.	n.a.	n.a.	n.a.	n.a.	n.a.
n.a.	cold fallyprode	n.a.	n.a.	n.a.	n.a.	n.a.	n.a.
n.a.	18F-fallypride	n.a.	n.a.	n.a.	n.a.	n.a.	n.a.
1		6.320	1.218	20.208	2.22	2.41	n.a.
2		7.680	1.488	24.803	2.71	2.95	n.a.
3		9.117	52.255	795.109	95.08	94.64	n.a.
**Total:**			**54.961**	**840.120**	**100.00**	**100.00**	

^{1}H NMR spectrum of 3-phenyl-6-(4-(trimethylstannyl)phenyl)-1,2,4,5-tetrazine (**23**) (400 MHz, $CDCl_3$)

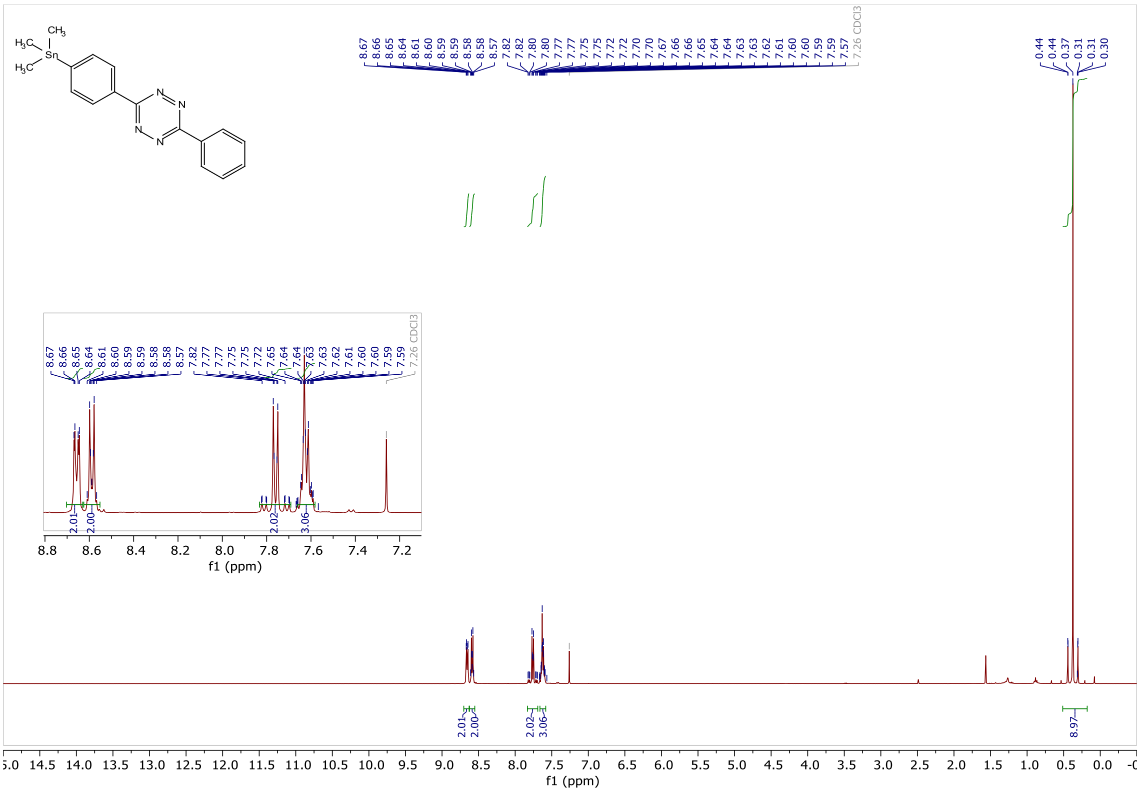

^{13}C NMR spectrum of 3-phenyl-6-(4-(trimethylstannyl)phenyl)-1,2,4,5-tetrazine (**23**) (101 MHz, CDCl3)

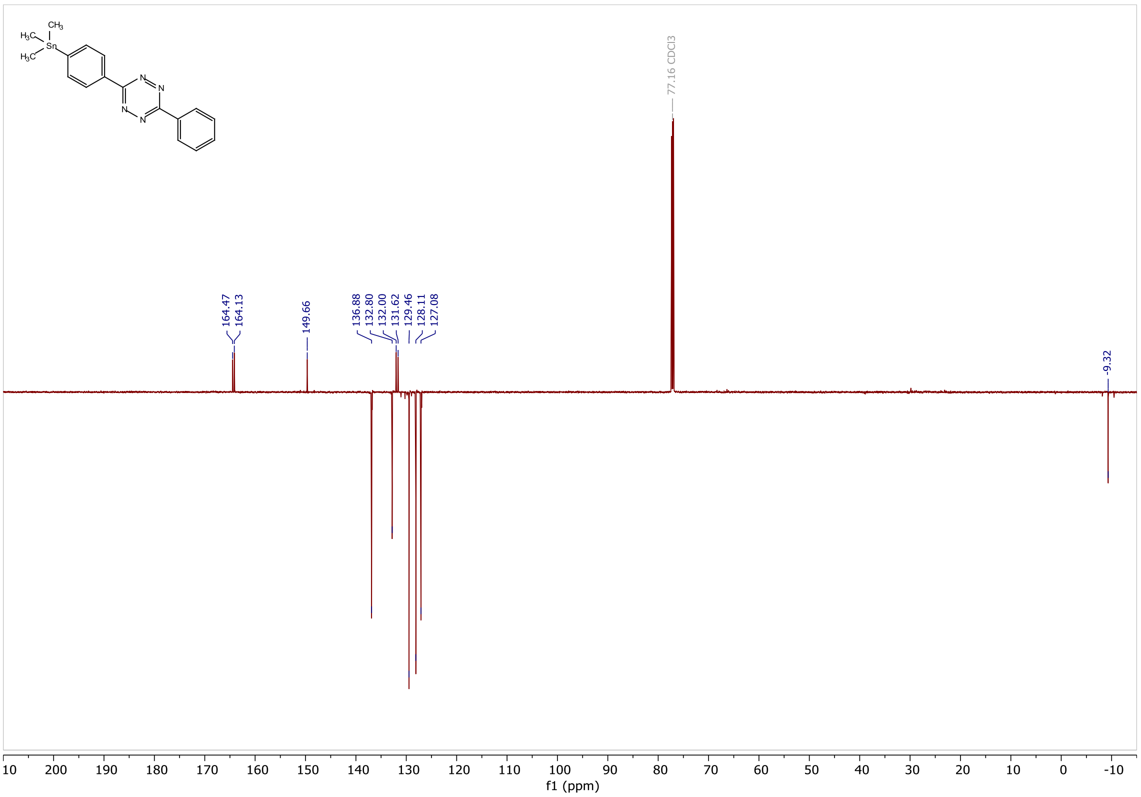

Analytical HPLC of 3-phenyl-6-(4-(trimethylstannyl)phenyl)-1,2,4,5-tetrazine (**23**)

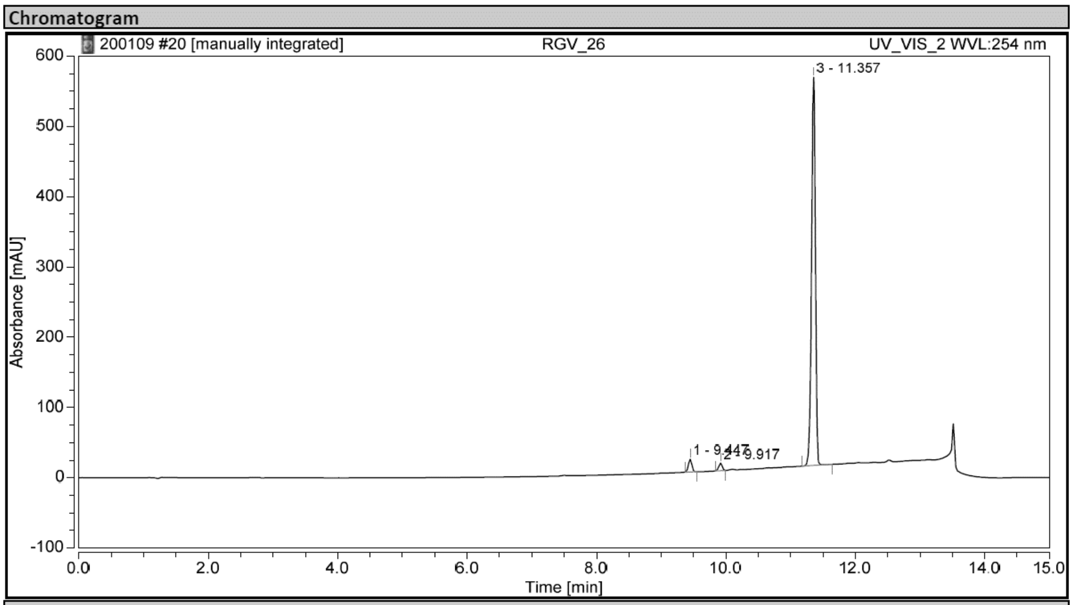

Integration Results

No.	Peak Name	Retention Time min	Area mAU*min	Height mAU	Relative Area %	Relative Height %	Amount
n.a.	Tosylate	n.a.	n.a.	n.a.	n.a.	n.a.	n.a.
n.a.	cold fallyprode	n.a.	n.a.	n.a.	n.a.	n.a.	n.a.
n.a.	18F-fallypride	n.a.	n.a.	n.a.	n.a.	n.a.	n.a.
1		9.447	1.115	18.332	2.81	3.15	n.a.
2		9.917	0.657	10.625	1.65	1.83	n.a.
3		11.357	37.970	552.200	95.54	95.02	n.a.
**Total:**			**39.743**	**581.157**	**100.00**	**100.00**	

^{1}H NMR spectrum of 3-phenyl-6-(3-(trimethylstannyl)phenyl)-1,2,4,5-tetrazine (**24**) (600 MHz, $CDCl_3$)

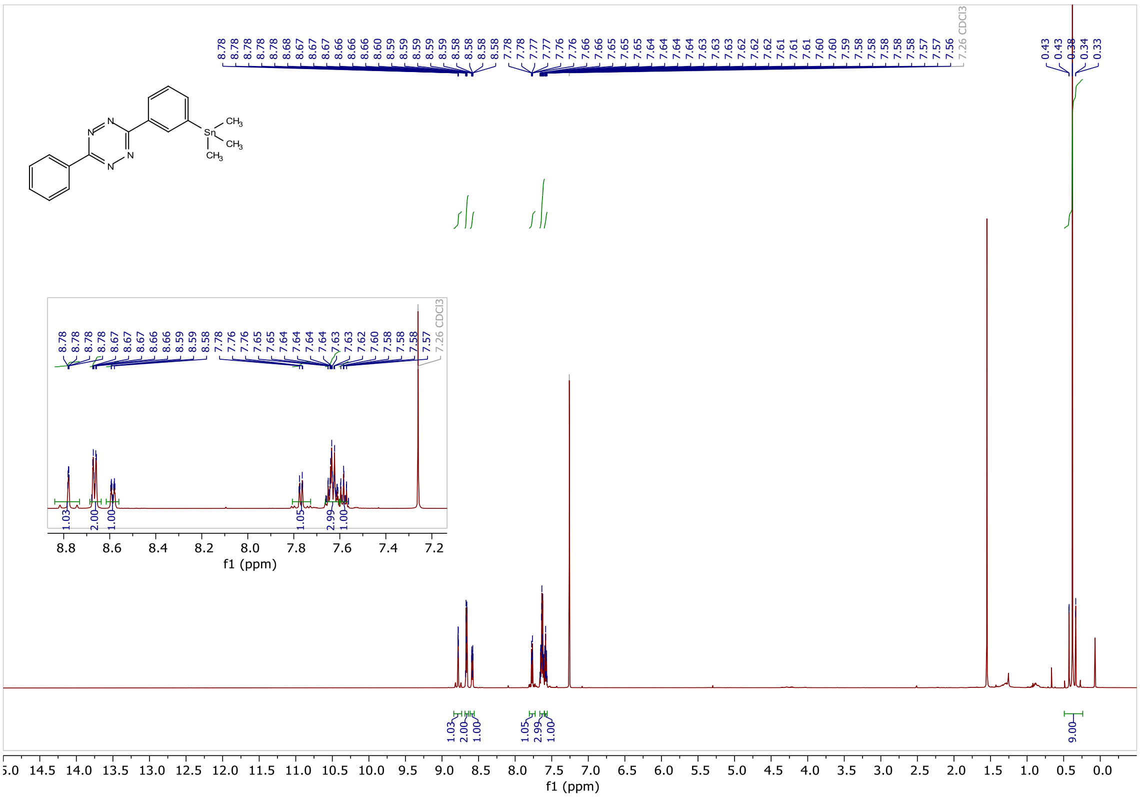

^{13}C NMR spectrum of 3-phenyl-6-(3-(trimethylstannyl)phenyl)-1,2,4,5-tetrazine (**24**) (101 MHz, CDCl3)

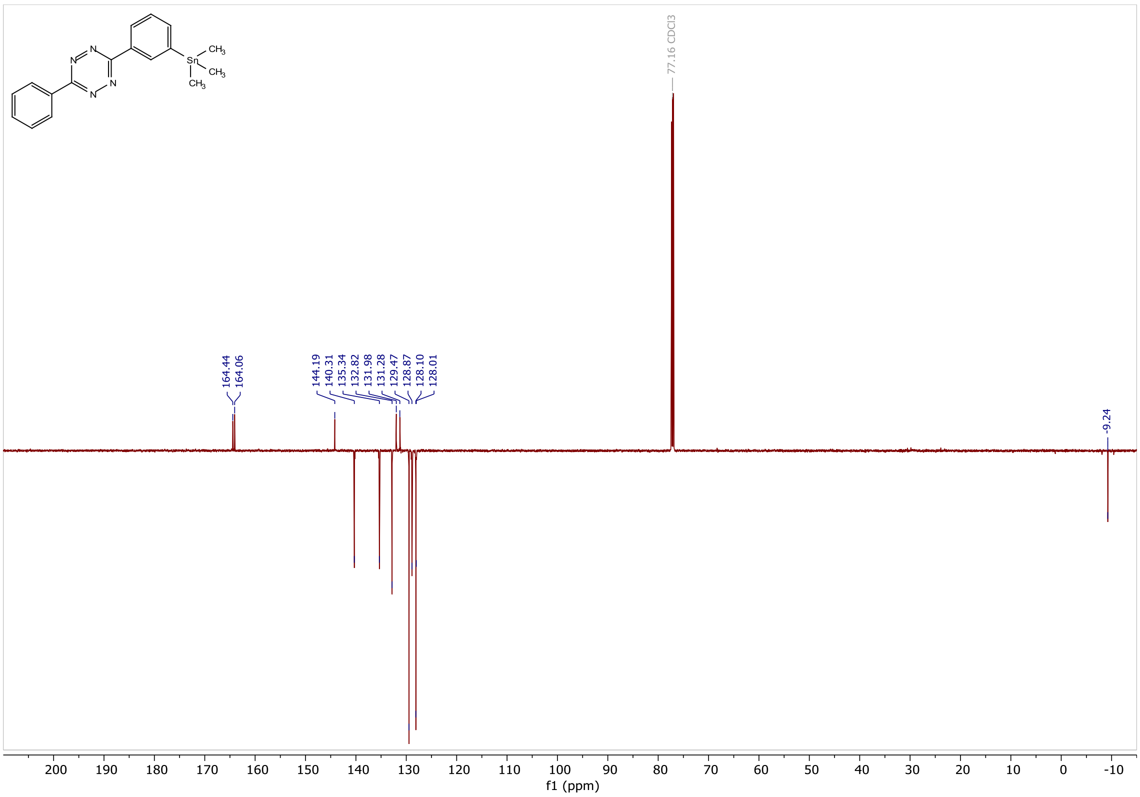

Analytical HPLC of 3-phenyl-6-(3-(trimethylstannyl)phenyl)-1,2,4,5-tetrazine (**24**)

Chromatogram

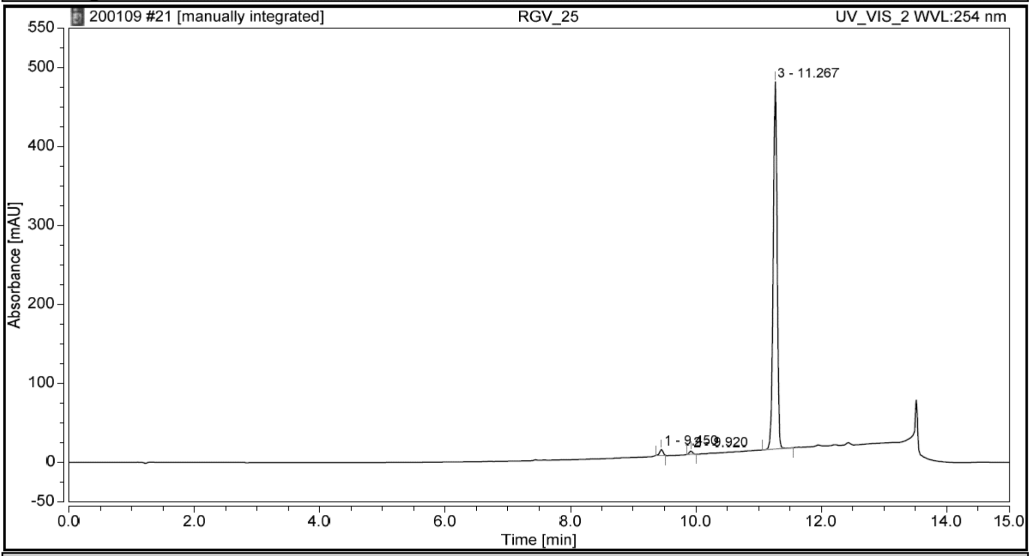

Integration Results

No.	Peak Name	Retention Time min	Area mAU*min	Height mAU	Relative Area %	Relative Height %	Amount
n.a.	Tosylate	n.a.	n.a.	n.a.	n.a.	n.a.	n.a.
n.a.	cold fallyprode	n.a.	n.a.	n.a.	n.a.	n.a.	n.a.
n.a.	18F-fallypride	n.a.	n.a.	n.a.	n.a.	n.a.	n.a.
1		9.450	0.493	7.683	1.43	1.61	n.a.
2		9.920	0.258	4.128	0.75	0.87	n.a.
3		11.267	33.708	464.981	97.82	97.52	n.a.
**Total:**			**34.460**	**476.792**	**100.00**	**100.00**	

^{1}H NMR spectrum of 3-(pyridin-2-yl)-6-(5-(trimethylstannyl)pyridin-2-yl)-1,2-dihydro-1,2,4,5-tetrazine (**26b**) (400 MHz, CDCl3)

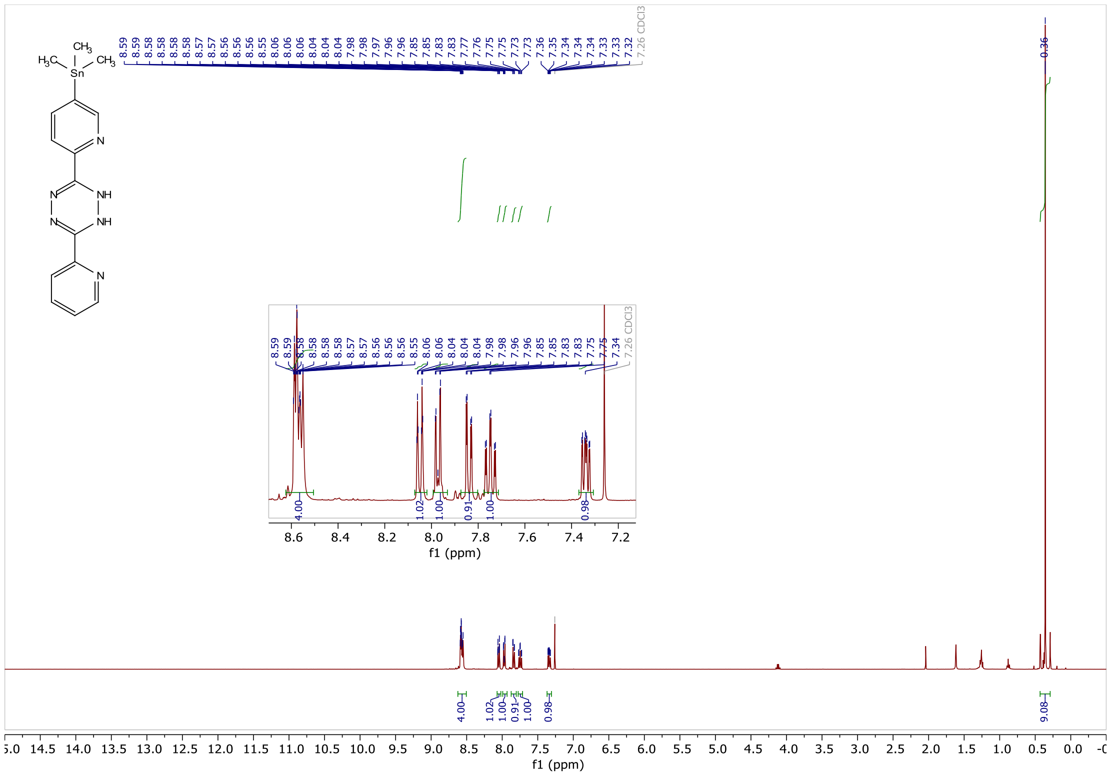

^{13}C NMR spectrum of 3-(pyridin-2-yl)-6-(5-(trimethylstannyl)pyridin-2-yl)-1,2-dihydro-1,2,4,5-tetrazine (**26b**) (101 MHz, CDCl3)

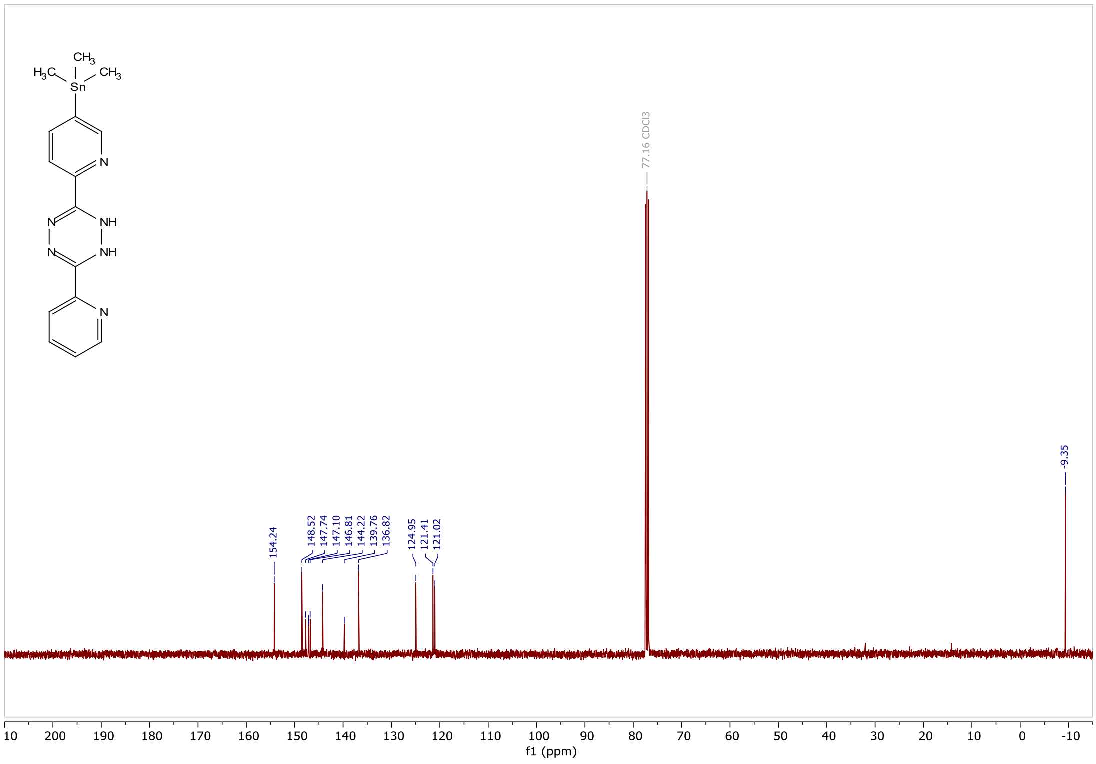

¹H NMR spectrum of 3-(pyridin-2-yl)-6-(5-(trimethylstannyl)pyridin-2-yl)-1,2,4,5-tetrazine (**26**) (400 MHz, CDCl3)

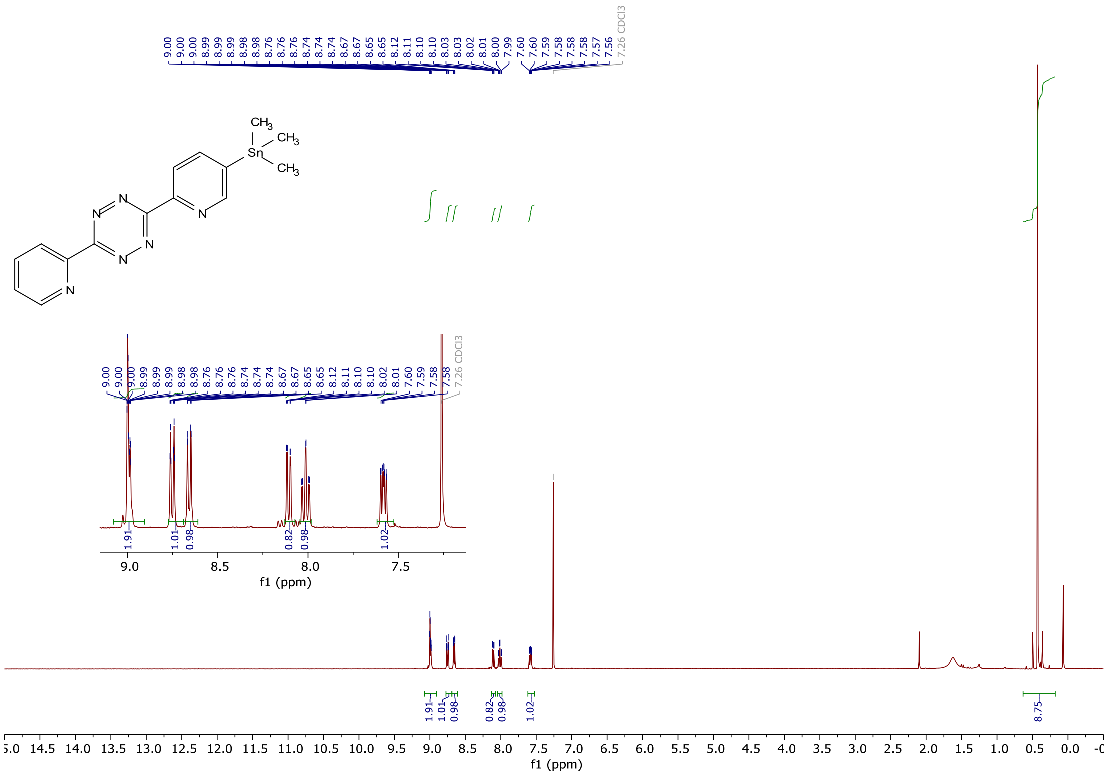

¹³C NMR spectrum of 3-(pyridin-2-yl)-6-(5-(trimethylstannyl)pyridin-2-yl)-1,2,4,5-tetrazine (**26**) (101 MHz, CDCl3)

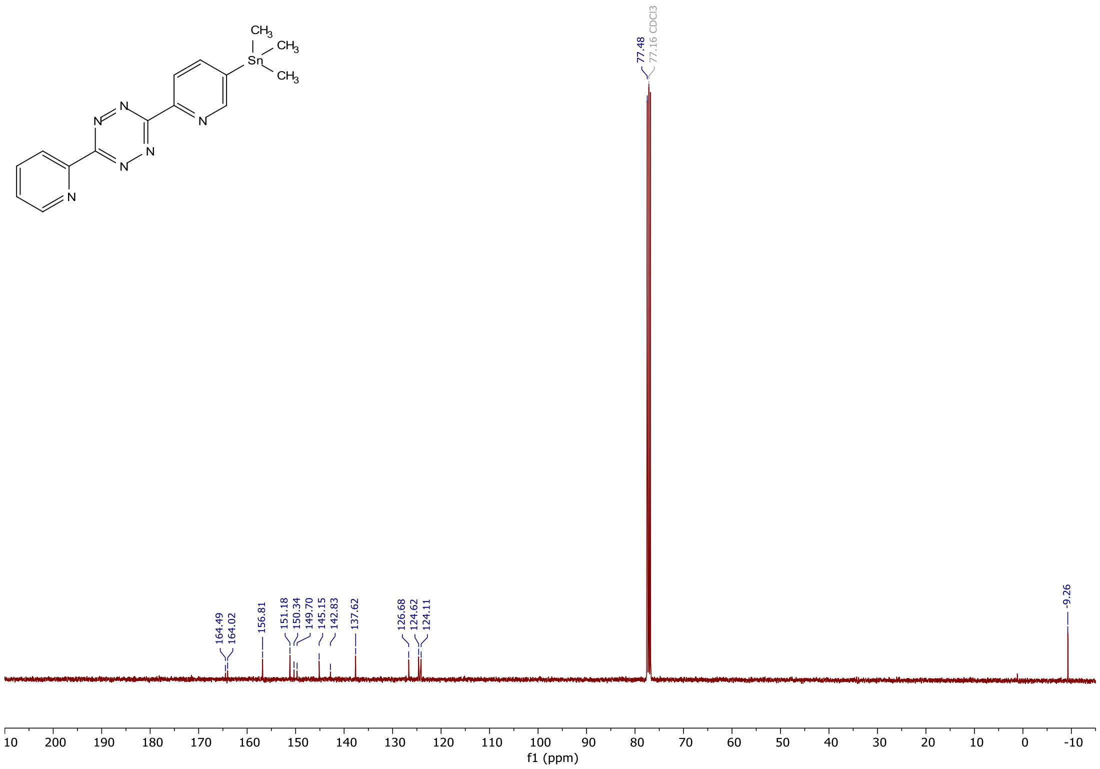

Analytical HPLC of 3-(pyridin-2-yl)-6-(5-(trimethylstannyl)pyridin-2-yl)-1,2,4,5-tetrazine (**26**)

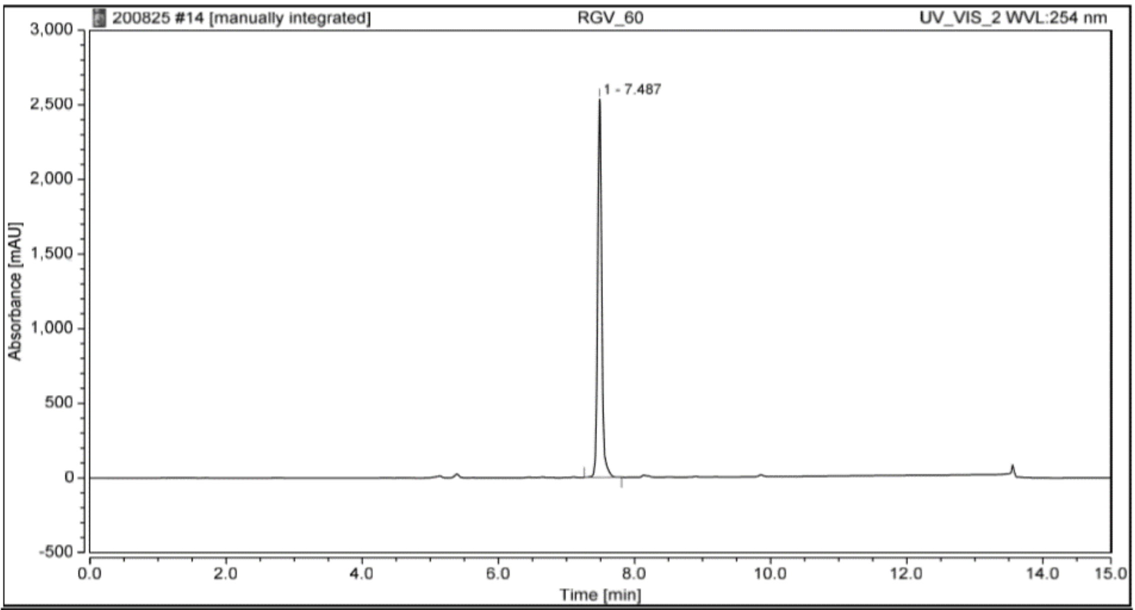

Integration Results

No.	Peak Name	Retention Time min	Area mAU*min	Height mAU	Relative Area %	Relative Height %	Amount
n.a.	Tosylate	n.a.	n.a.	n.a.	n.a.	n.a.	n.a.
n.a.	cold fallyprode	n.a.	n.a.	n.a.	n.a.	n.a.	n.a.
n.a.	18F-fallypride	n.a.	n.a.	n.a.	n.a.	n.a.	n.a.
1		7.487	179.016	2535.339	100.00	100.00	n.a.
**Total:**			**179.016**	**2535.339**	**100.00**	**100.00**	

^{1}H NMR spectrum of 3-(pyridin-2-yl)-6-(4-(trimethylstannyl)phenyl)-1,2,4,5-tetrazine (**25**) (400 MHz, $CDCl_3$)

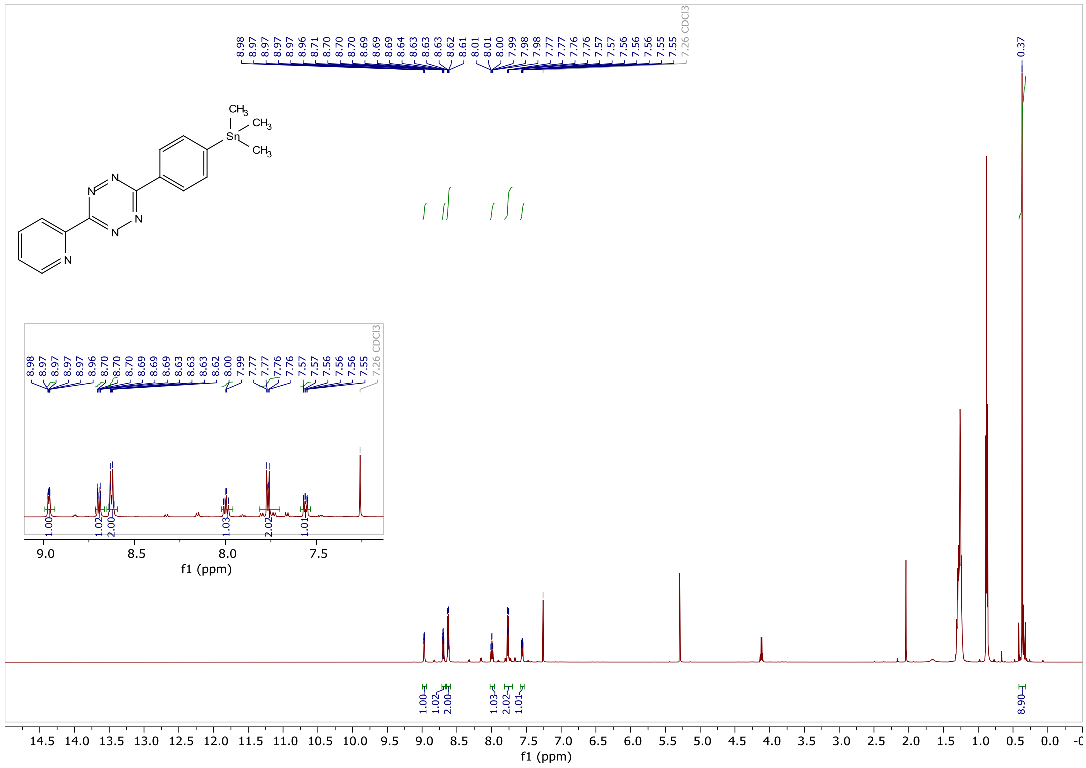

^{13}C NMR spectrum of 3-(pyridin-2-yl)-6-(4-(trimethylstannyl)phenyl)-1,2,4,5-tetrazine (**25**) (151 MHz, CDCl3)

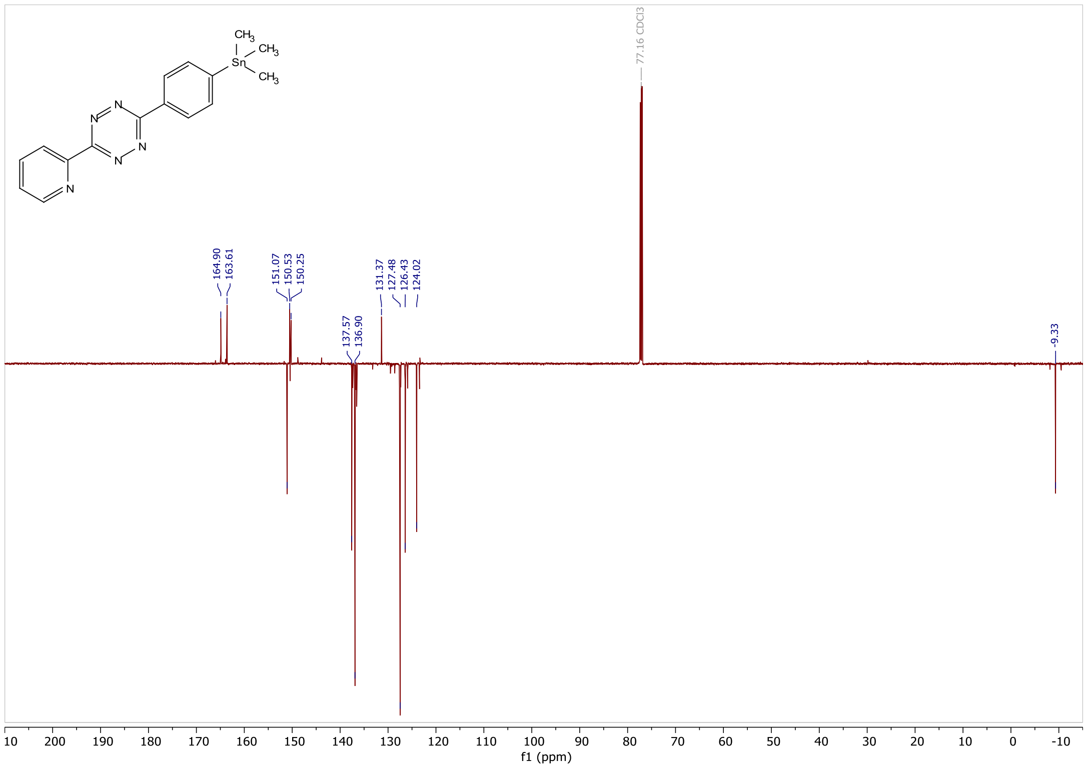

Analytical HPLC of 3-(pyridin-2-yl)-6-(4-(trimethylstannyl)phenyl)-1,2,4,5-tetrazine (**25**)

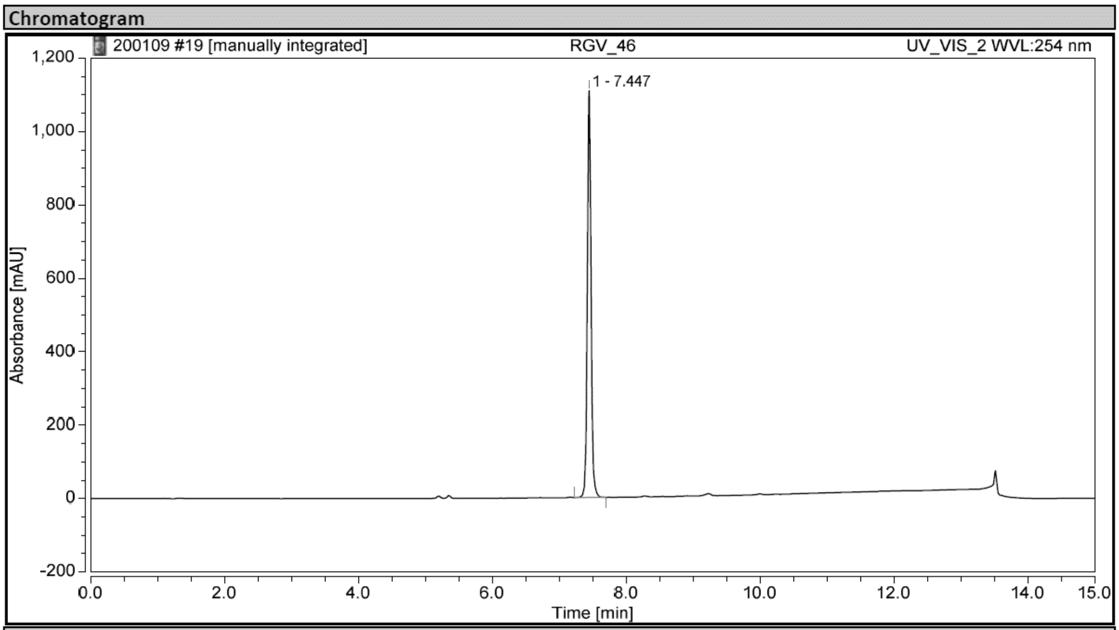

Integration Results

No.	Peak Name	Retention Time min	Area mAU*min	Height mAU	Relative Area %	Relative Height %	Amount
n.a.	Tosylate	n.a.	n.a.	n.a.	n.a.	n.a.	n.a.
n.a.	cold fallyprode	n.a.	n.a.	n.a.	n.a.	n.a.	n.a.
n.a.	18F-fallypride	n.a.	n.a.	n.a.	n.a.	n.a.	n.a.
1		7.447	72.267	1107.708	100.00	100.00	n.a.
**Total:**			**72.267**	**1107.708**	**100.00**	**100.00**	

^{1}H NMR spectrum of 3-(3-methyl-5-(trimethylstannyl)phenyl)-1,2,4,5-tetrazine (**34**) (400 MHz, $CDCl_3$)

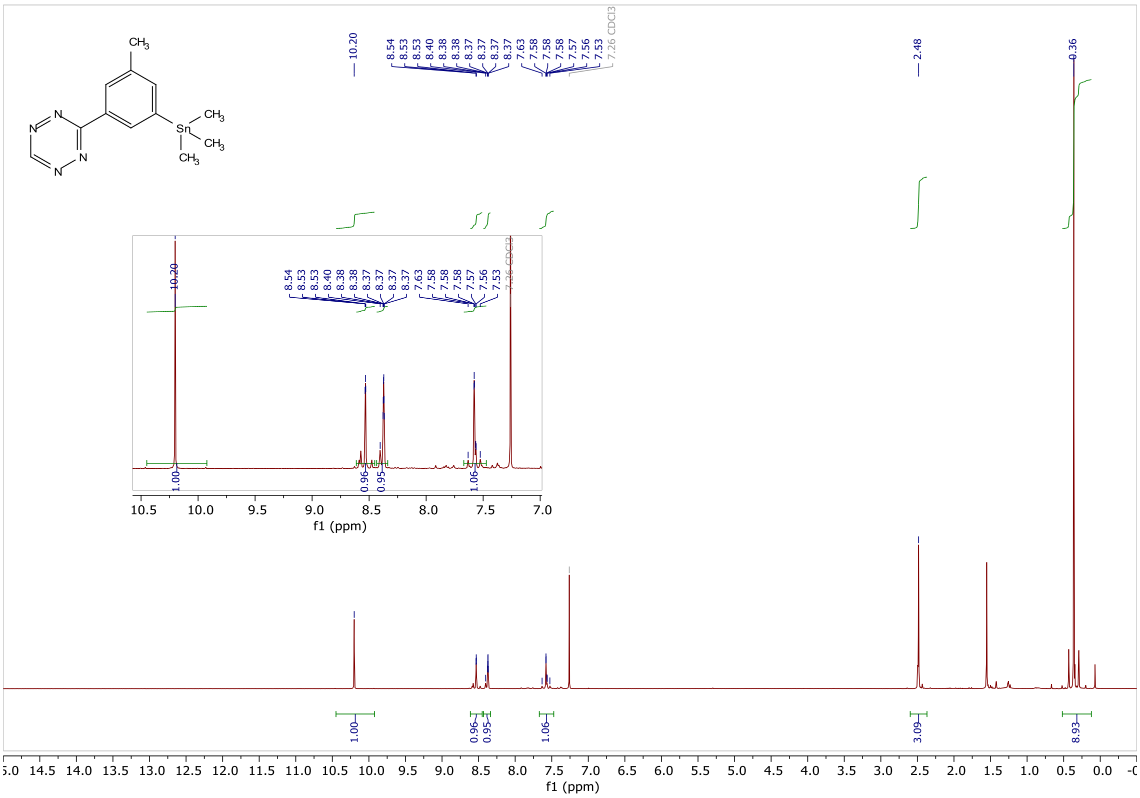

^{13}C NMR spectrum of 3-(3-methyl-5-(trimethylstannyl)phenyl)-1,2,4,5-tetrazine (**34**) (151 MHz, CDCl3)

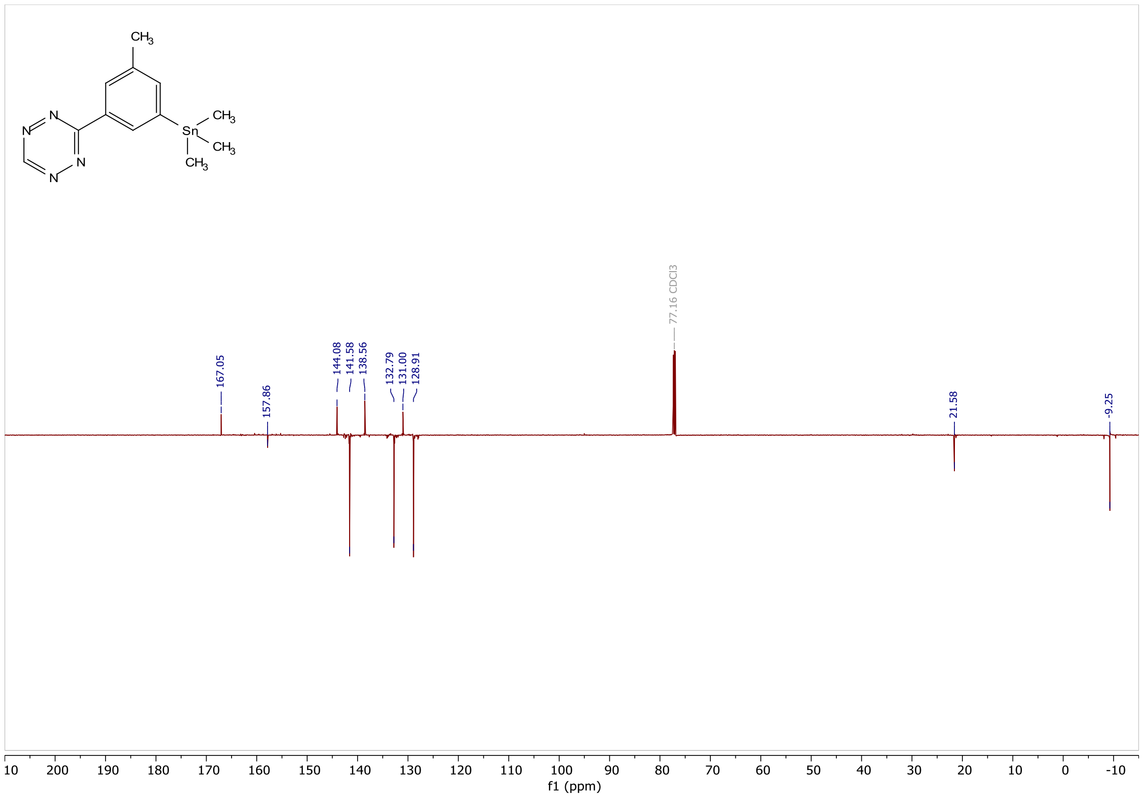

Analytical HPLC 3-(3-methyl-5-(trimethylstannyl)phenyl)-1,2,4,5-tetrazine (**34**)

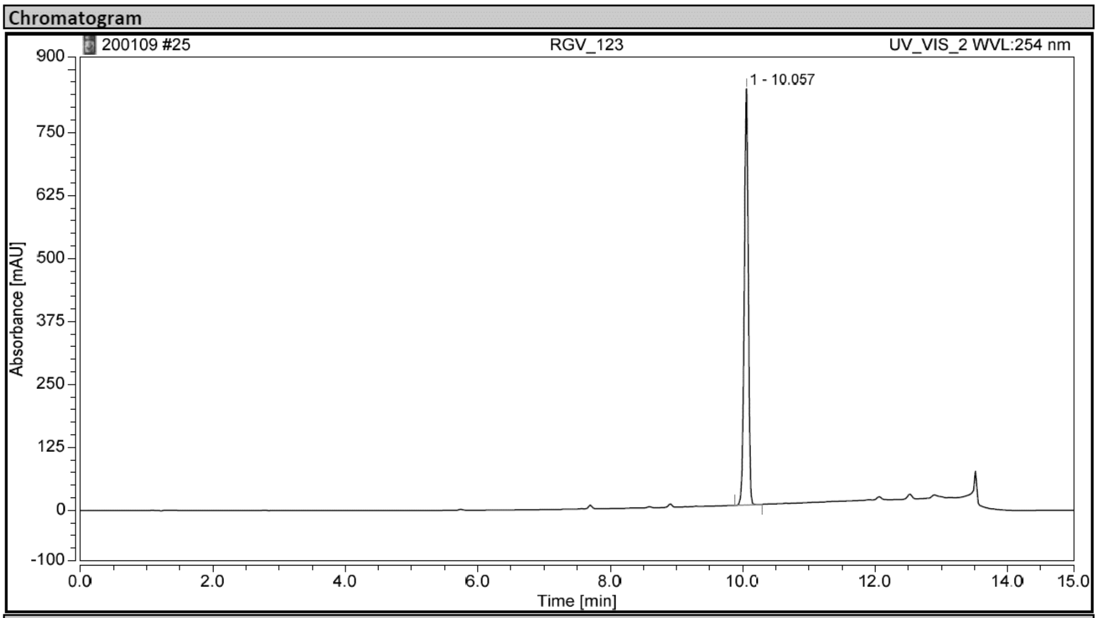

Integration Results

No.	Peak Name	Retention Time min	Area mAU*min	Height mAU	Relative Area %	Relative Height %	Amount
n.a.	Tosylate	n.a.	n.a.	n.a.	n.a.	n.a.	n.a.
n.a.	cold fallyprode	n.a.	n.a.	n.a.	n.a.	n.a.	n.a.
n.a.	18F-fallypride	n.a.	n.a.	n.a.	n.a.	n.a.	n.a.
1		10.057	57.359	825.936	100.00	100.00	n.a.
**Total:**			**57.359**	**825.936**	**100.00**	**100.00**	

^{1}H NMR spectrum of 3-(2-methyl-5-(trimethylstannyl)phenyl)-1,2,4,5-tetrazine (**39**) (600 MHz, $CDCl_3$)

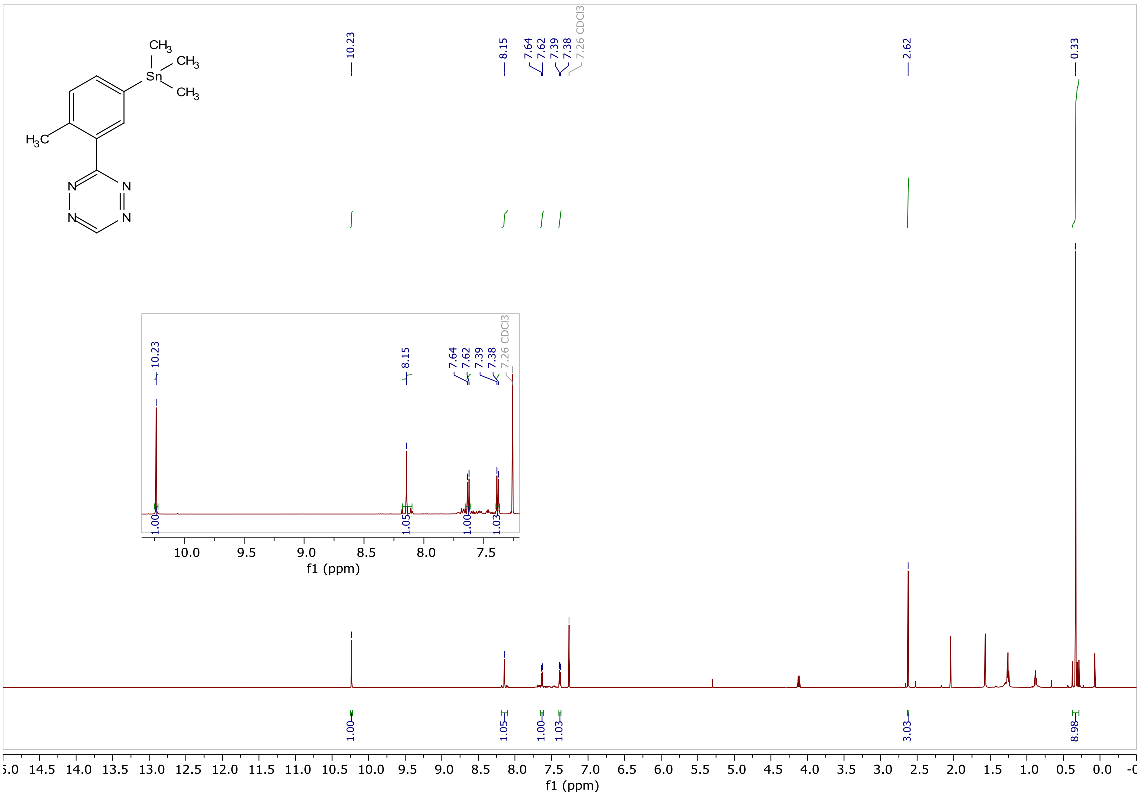

^{13}C NMR spectrum of 3-(2-methyl-5-(trimethylstannyl)phenyl)-1,2,4,5-tetrazine (**39**) (151 MHz, CDCl3)

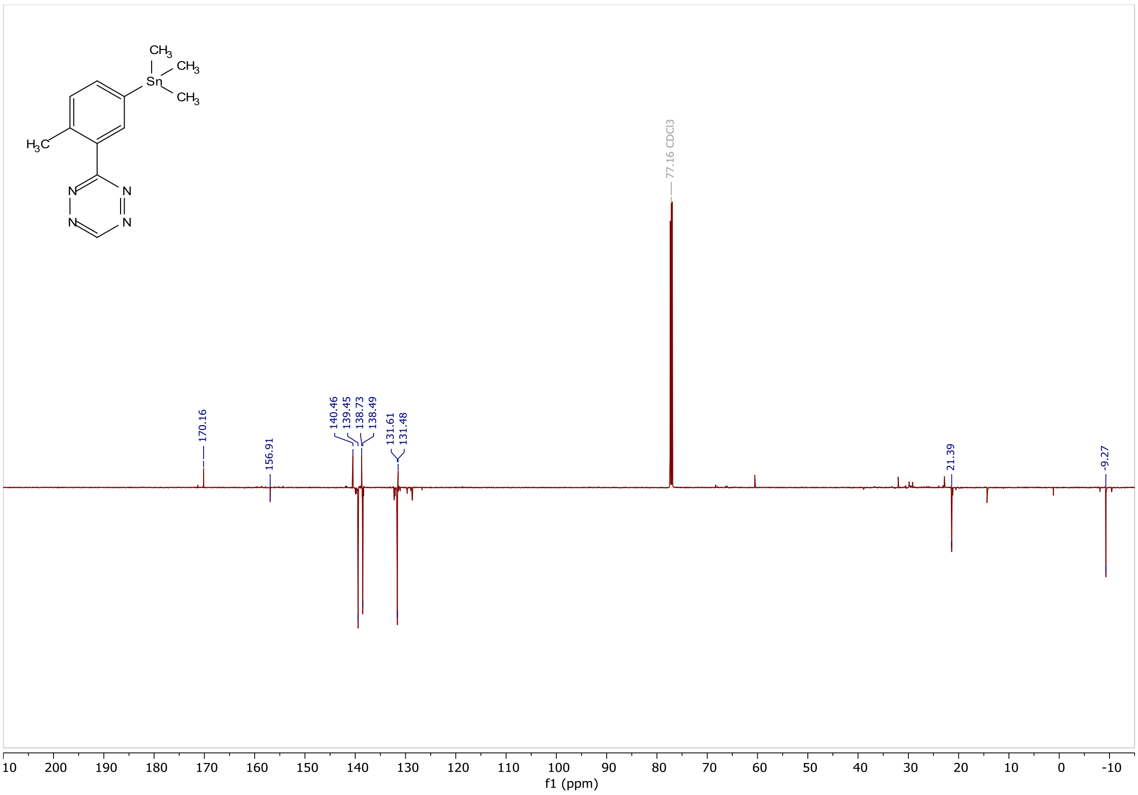

Analytical HPLC of 3-(2-methyl-5-(trimethylstannyl)phenyl)-1,2,4,5-tetrazine (**39**)

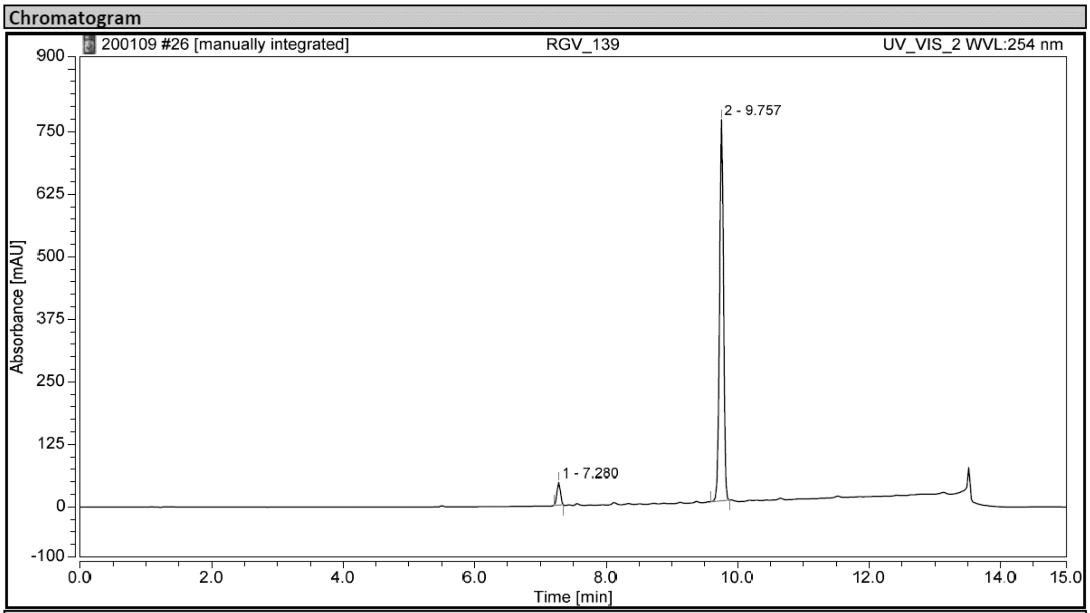

Integration Results

No.	Peak Name	Retention Time min	Area mAU*min	Height mAU	Relative Area %	Relative Height %	Amount
n.a.	Tosylate	n.a.	n.a.	n.a.	n.a.	n.a.	n.a.
n.a.	cold fallyprode	n.a.	n.a.	n.a.	n.a.	n.a.	n.a.
n.a.	18F-fallypride	n.a.	n.a.	n.a.	n.a.	n.a.	n.a.
1		7.280	2.725	45.486	4.91	5.64	n.a.
2		9.757	52.783	761.481	95.09	94.36	n.a.
**Total:**			**55.508**	**806.967**	**100.00**	**100.00**	

^{1}H NMR spectrum of 3-(3-methoxy-5-(trimethylstannyl)phenyl)-1,2,4,5-tetrazine (**35**) (400 MHz, $CDCl_3$)

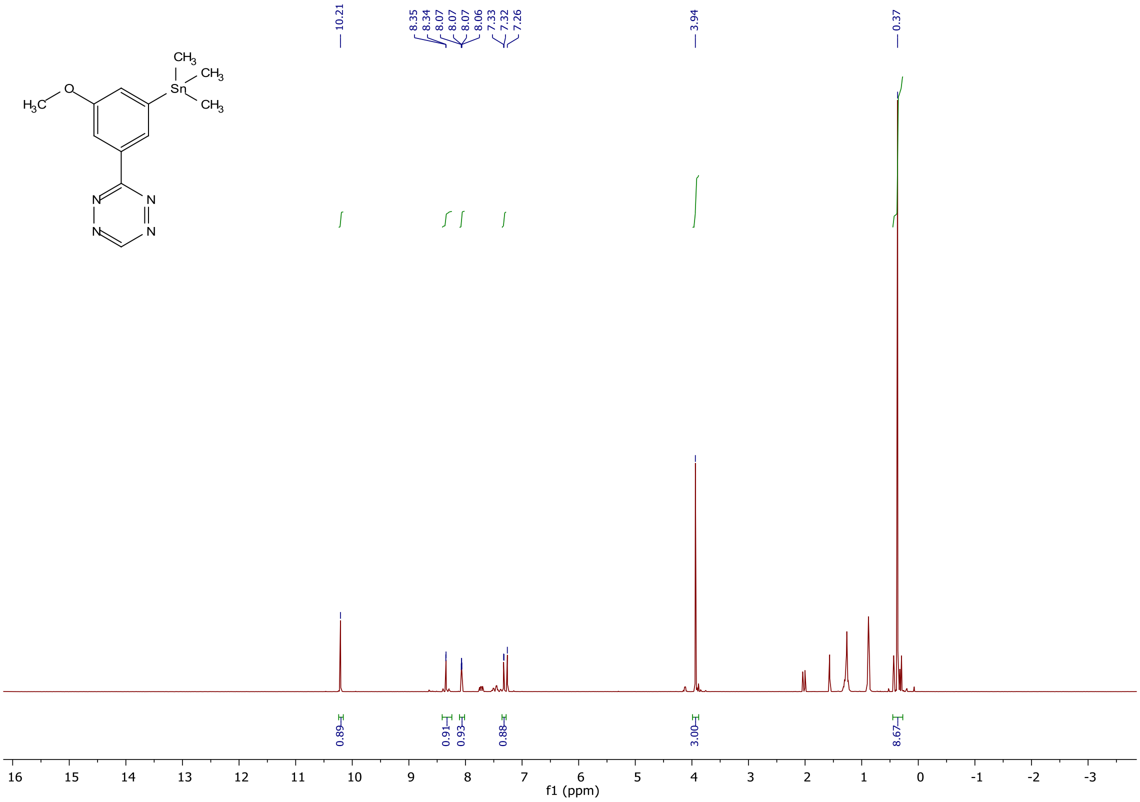

^{13}C NMR spectrum of 3-(3-methoxy-5-(trimethylstannyl)phenyl)-1,2,4,5-tetrazine (**35**) (101 MHz, $CDCl_3$)

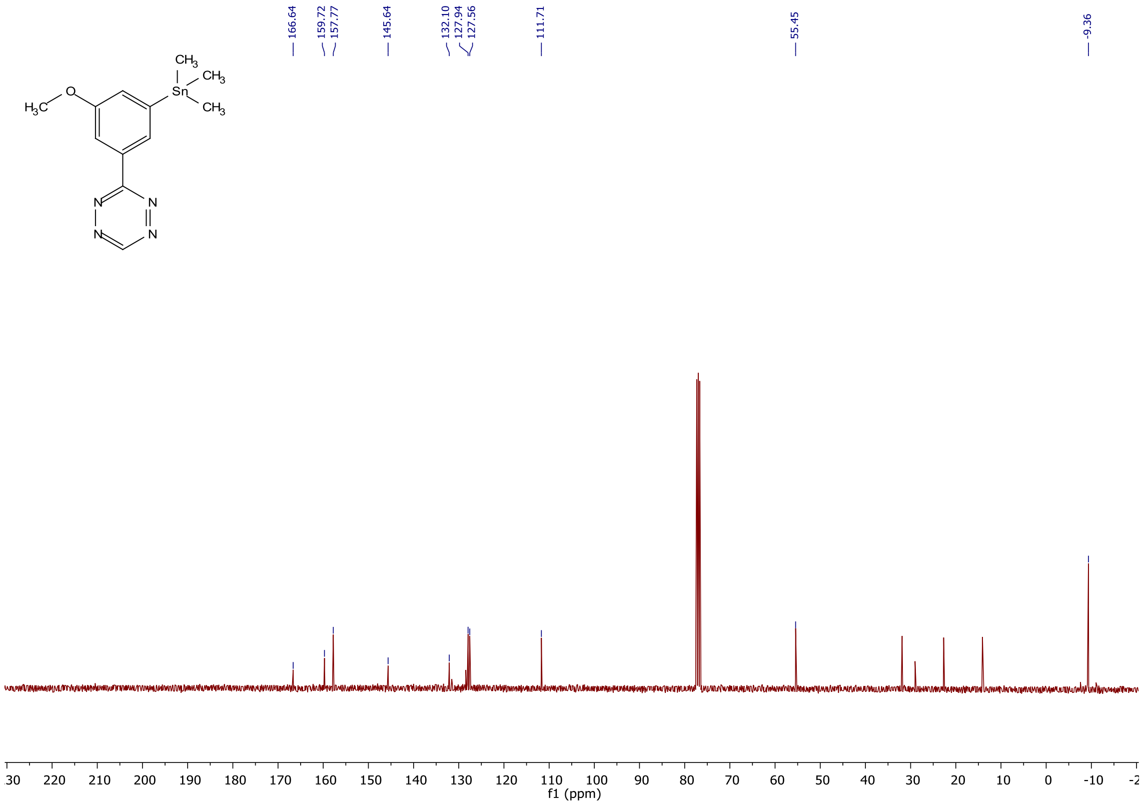

Analytical HPLC of 3-(3-methoxy-5-(trimethylstannyl)phenyl)-1,2,4,5-tetrazine (**35**)

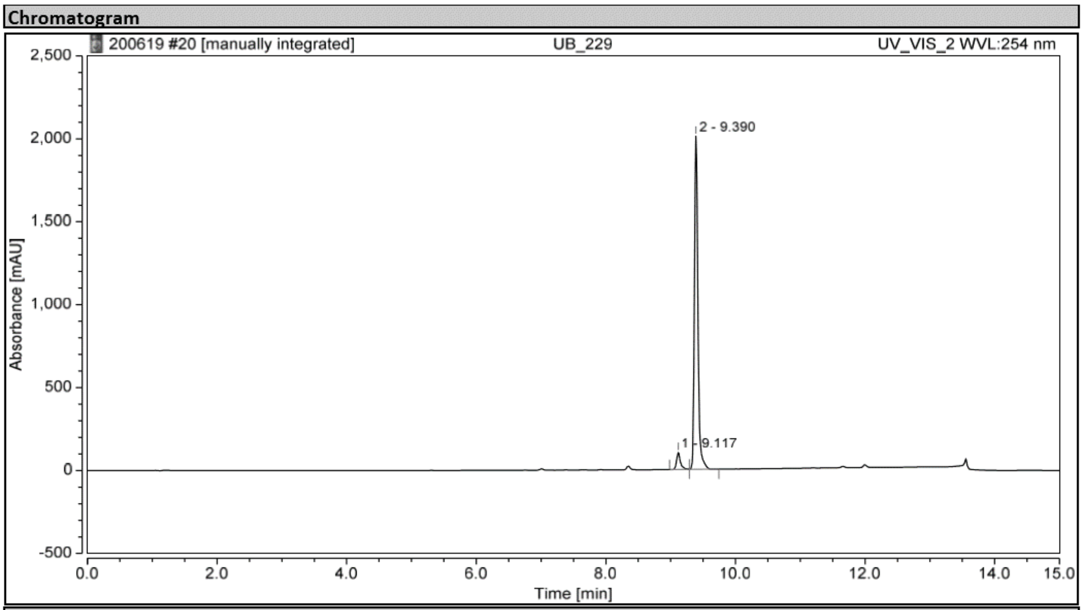

Integration Results

No.	Peak Name	Retention Time<br>min	Area<br>mAU*min	Height<br>mAU	Relative Area<br>%	Relative Height<br>%	Amount
n.a.	Tosylate	n.a.	n.a.	n.a.	n.a.	n.a.	n.a.
n.a.	cold fallyprode	n.a.	n.a.	n.a.	n.a.	n.a.	n.a.
n.a.	18F-fallypride	n.a.	n.a.	n.a.	n.a.	n.a.	n.a.
1		9.117	7.029	103.278	5.24	4.89	n.a.
2		9.390	127.103	2009.312	94.76	95.11	n.a.
**Total:**			**134.132**	**2112.590**	**100.00**	**100.00**	

^{1}H NMR spectrum of N-(3-(1,2,4,5-tetrazin-3-yl)-5-(trimethylstannyl)phenyl)acetamide (**36**) (400 MHz, $CDCl_3$)

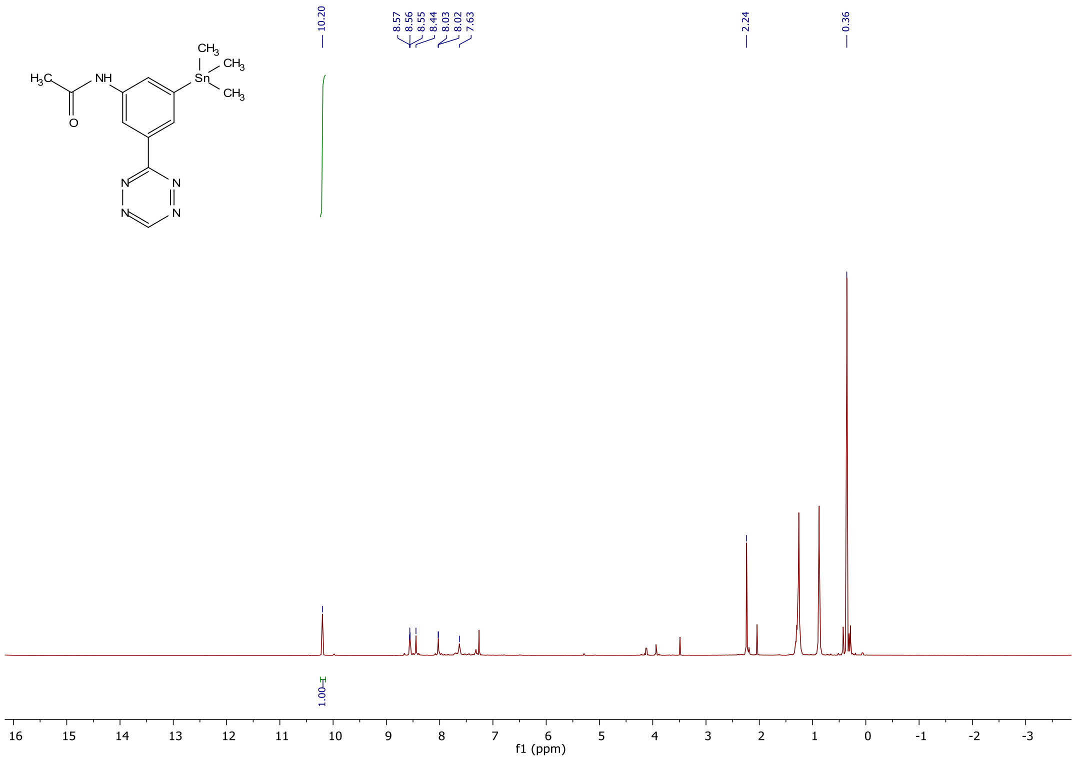

^{13}C NMR spectrum of N-(3-(1,2,4,5-tetrazin-3-yl)-5-(trimethylstannyl)phenyl)acetamide (**36**) (101 MHz, $CDCl_3$)

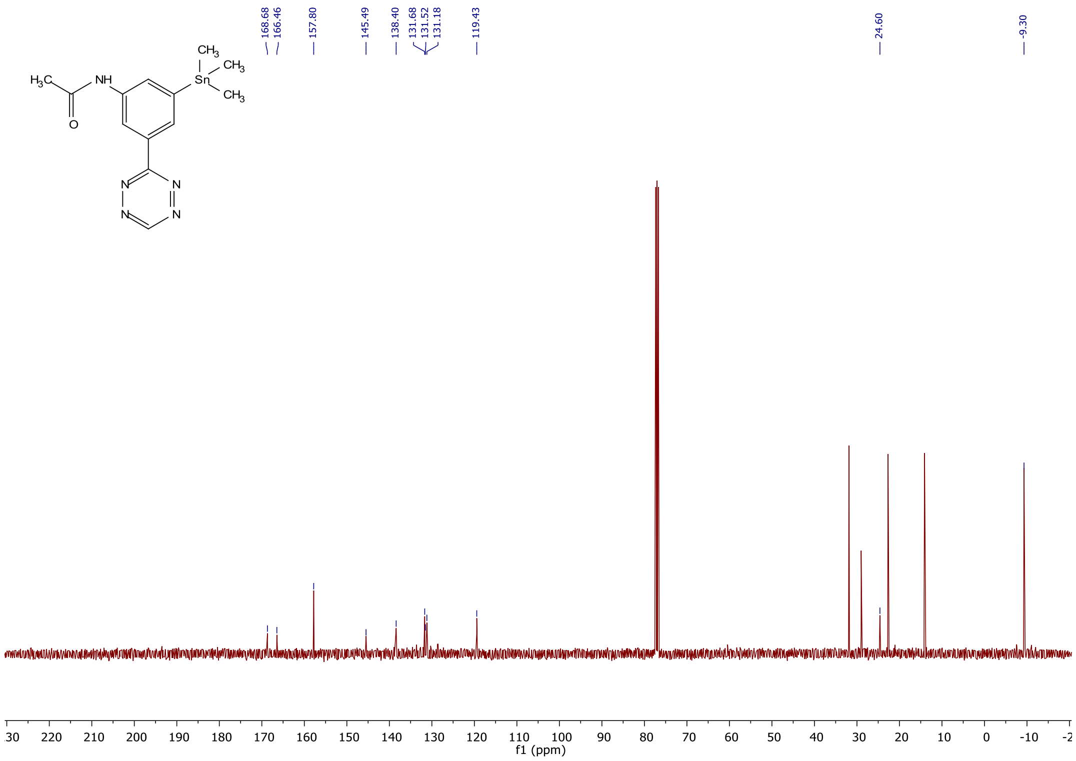

Analytical HPLC of N-(3-(1,2,4,5-tetrazin-3-yl)-5-(trimethylstannyl)phenyl)acetamide (**36**)

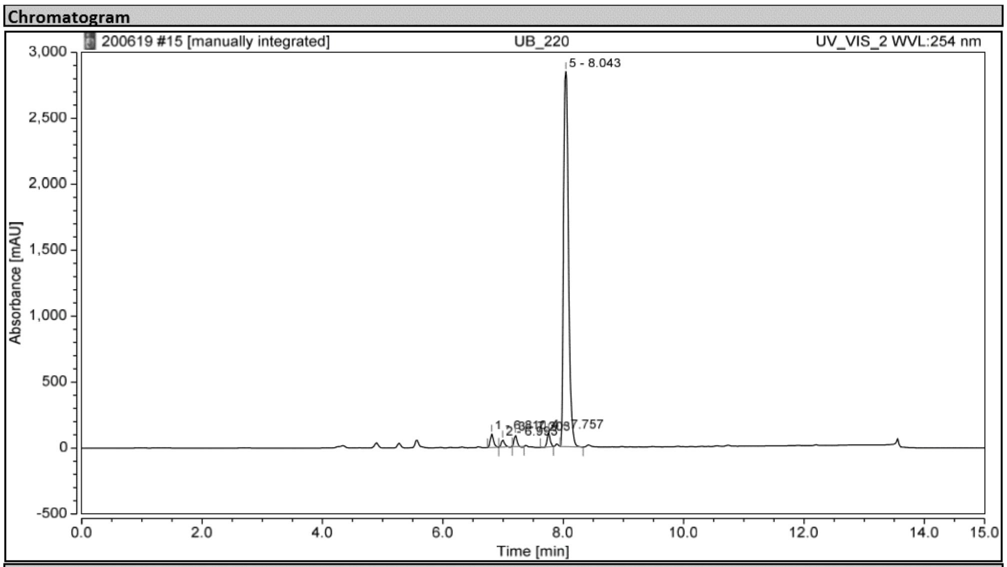

Integration Results

No.	Peak Name	Retention Time min	Area mAU*min	Height mAU	Relative Area %	Relative Height %	Amount
n.a.	Tosylate	n.a.	n.a.	n.a.	n.a.	n.a.	n.a.
n.a.	cold fallyprode	n.a.	n.a.	n.a.	n.a.	n.a.	n.a.
n.a.	18F-fallypride	n.a.	n.a.	n.a.	n.a.	n.a.	n.a.
1		6.810	6.553	102.884	2.27	3.22	n.a.
2		6.993	3.062	54.380	1.06	1.70	n.a.
3		7.203	4.560	87.173	1.58	2.73	n.a.
4		7.757	5.964	106.166	2.07	3.32	n.a.
5		8.043	268.288	2843.690	93.02	89.02	n.a.
**Total:**			**288.427**	**3194.293**	**100.00**	**100.00**	

^{1}H NMR spectrum of 3-(1,2,4,5-tetrazin-3-yl)-5-(trimethylstannyl)benzamide (**37**) (600 MHz, CD_3OD)

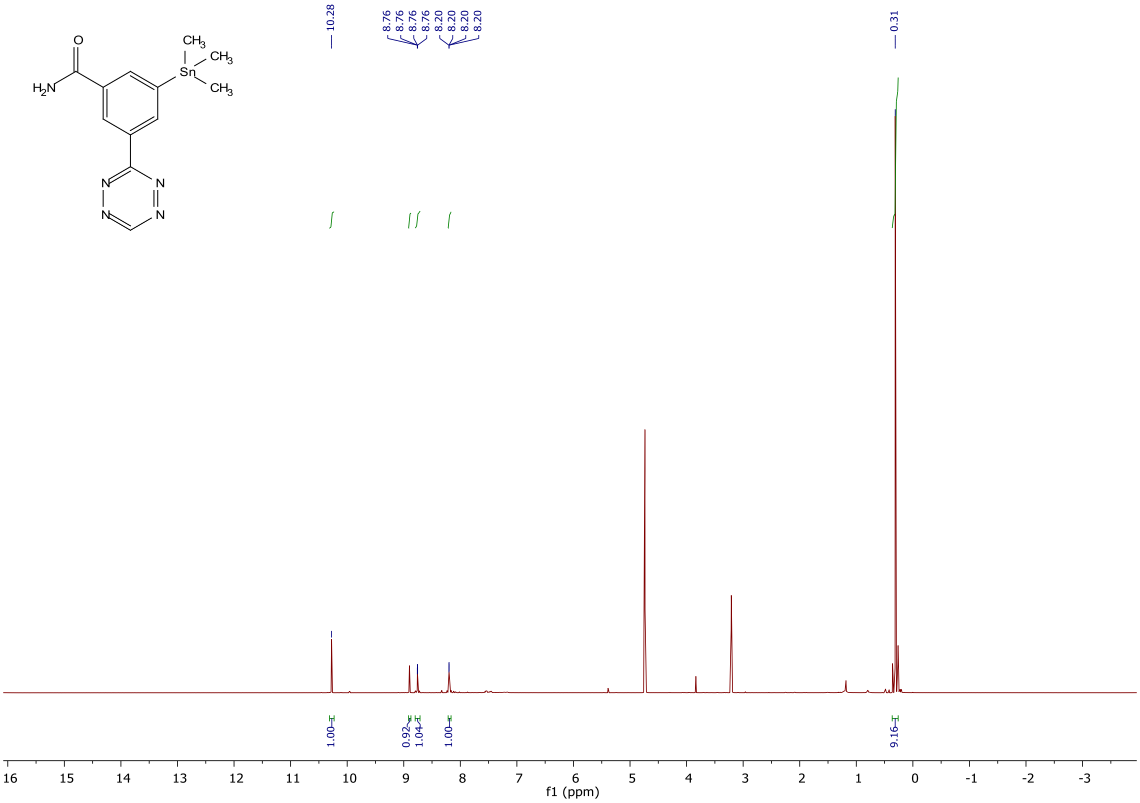

^{13}C NMR spectrum of 3-(1,2,4,5-tetrazin-3-yl)-5-(trimethylstannyl)benzamide (**37**) (151 MHz, CD_3OD)

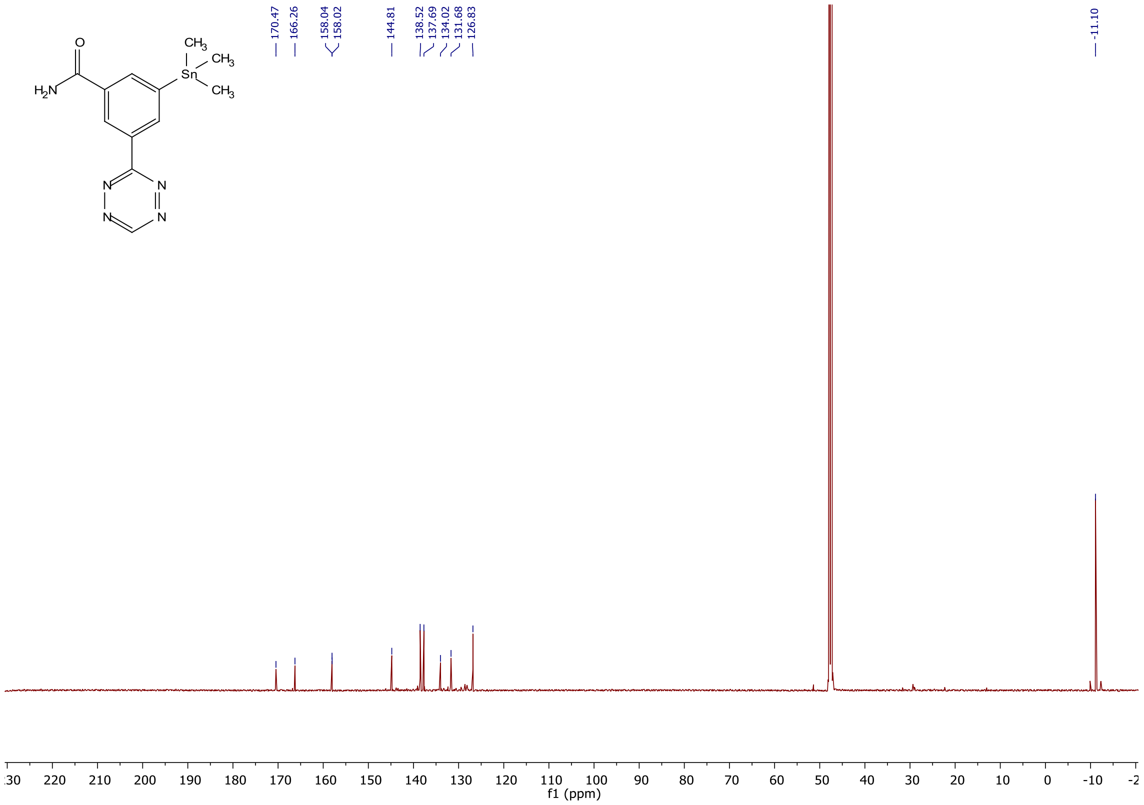

Analytical HPLC of 3-(1,2,4,5-tetrazin-3-yl)-5-(trimethylstannyl)benzamide (**37**)

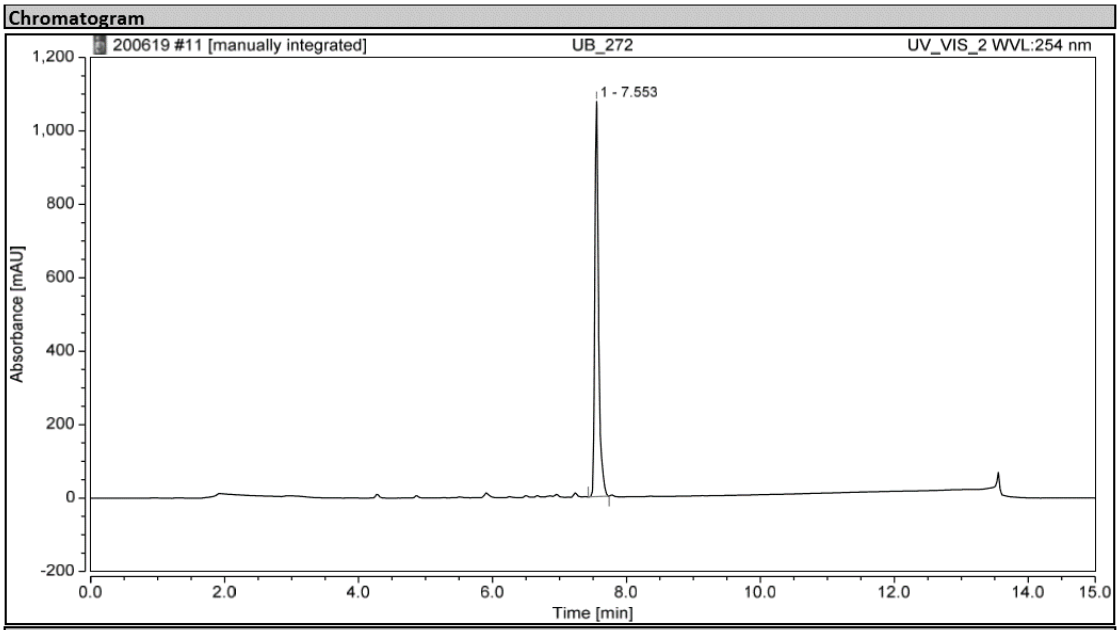

Integration Results

No.	Peak Name	Retention Time min	Area mAU*min	Height mAU	Relative Area %	Relative Height %	Amount
n.a.	Tosylate	n.a.	n.a.	n.a.	n.a.	n.a.	n.a.
n.a.	cold fallyprode	n.a.	n.a.	n.a.	n.a.	n.a.	n.a.
n.a.	18F-fallypride	n.a.	n.a.	n.a.	n.a.	n.a.	n.a.
1		7.553	69.447	1075.856	100.00	100.00	n.a.
**Total:**			**69.447**	**1075.856**	**100.00**	**100.00**	

^{1}H NMR spectrum of N-methyl-3-(1,2,4,5-tetrazin-3-yl)-5-(trimethylstannyl)benzamide (**38**) (400 MHz, $CDCl_3$)

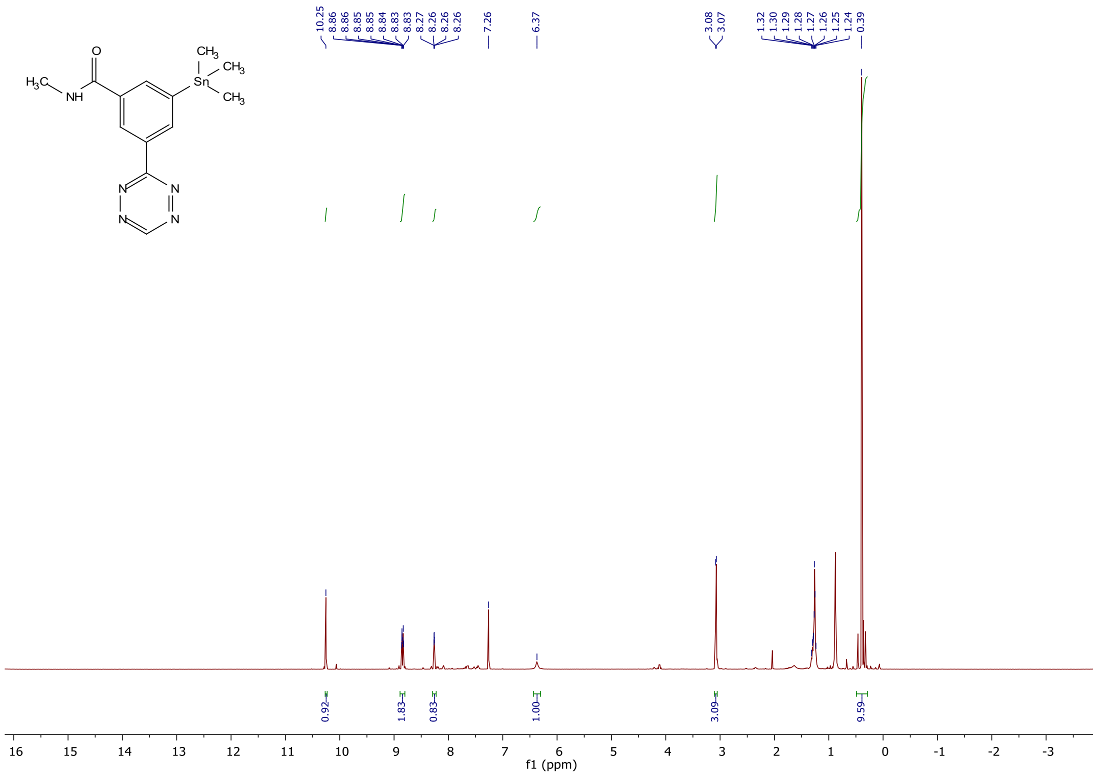

^{13}C NMR spectrum of N-methyl-3-(1,2,4,5-tetrazin-3-yl)-5-(trimethylstannyl)benzamide (**38**) (101 MHz, $CDCl_3$)

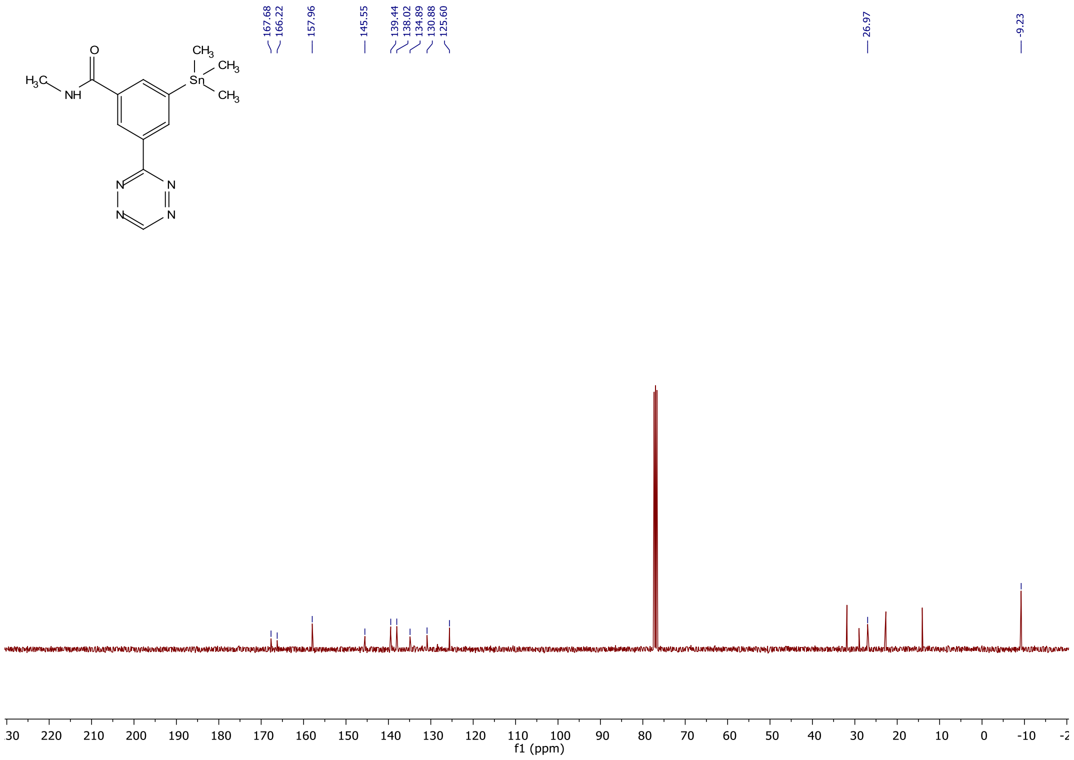

Analytical HPLC of N-methyl-3-(1,2,4,5-tetrazin-3-yl)-5-(trimethylstannyl)benzamide (**38**)

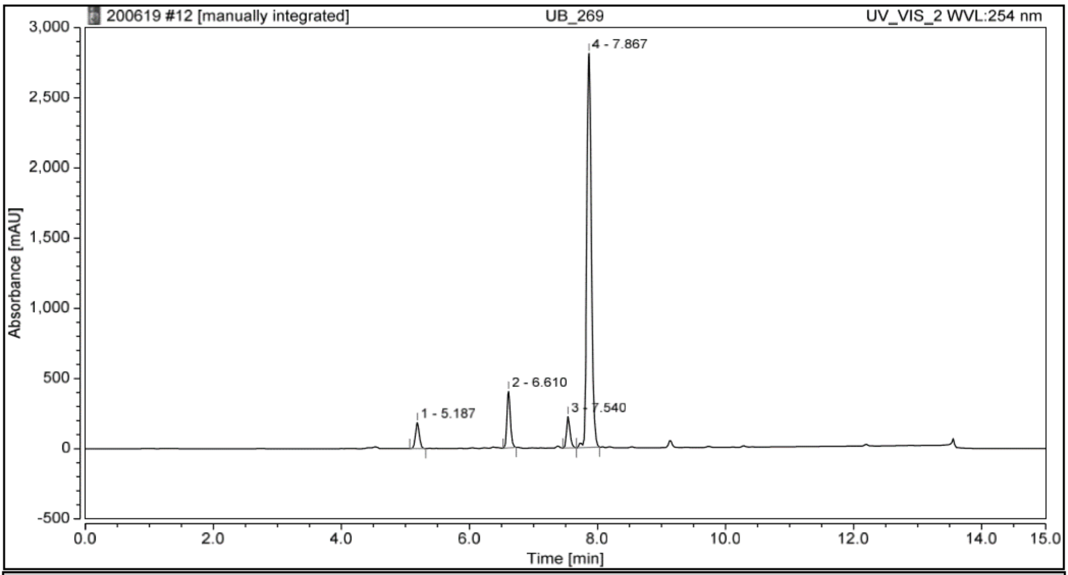

Integration Results							
No.	Peak Name	Retention Time min	Area mAU*min	Height mAU	Relative Area %	Relative Height %	Amount
n.a.	Tosylate	n.a.	n.a.	n.a.	n.a.	n.a.	n.a.
1		5.187	12.487	184.877	4.35	5.11	n.a.
n.a.	cold fallyprode	n.a.	n.a.	n.a.	n.a.	n.a.	n.a.
n.a.	18F-fallypride	n.a.	n.a.	n.a.	n.a.	n.a.	n.a.
2		6.610	25.181	402.124	8.78	11.11	n.a.
3		7.540	13.856	223.196	4.83	6.17	n.a.
4		7.867	235.301	2808.500	82.04	77.61	n.a.
**Total:**			**286.825**	**3618.698**	**100.00**	**100.00**	

^{1}H NMR spectrum of 3-fluoro-5-(1,2,4,5-tetrazin-3-yl)benzoic acid (**19**) (101 MHz, CD_3OD)

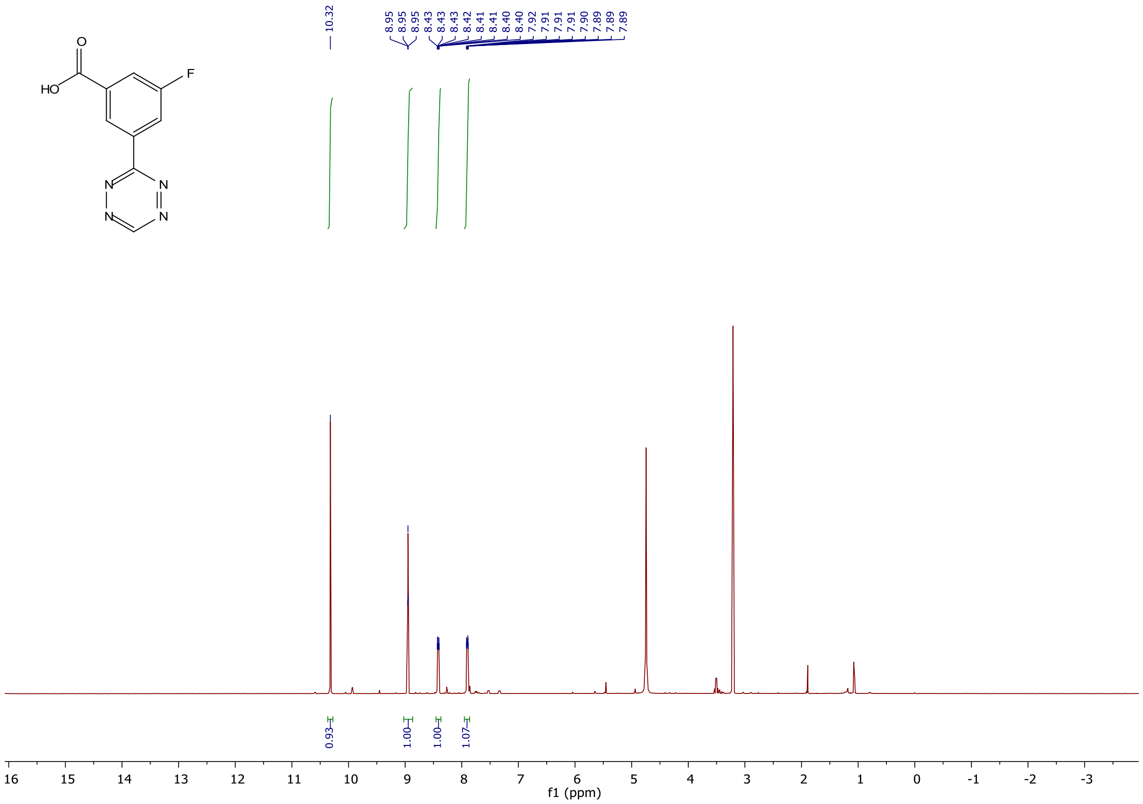

^{13}C NMR spectrum of 3-fluoro-5-(1,2,4,5-tetrazin-3-yl)benzoic acid (**19**) (101 MHz, CD_3OD)

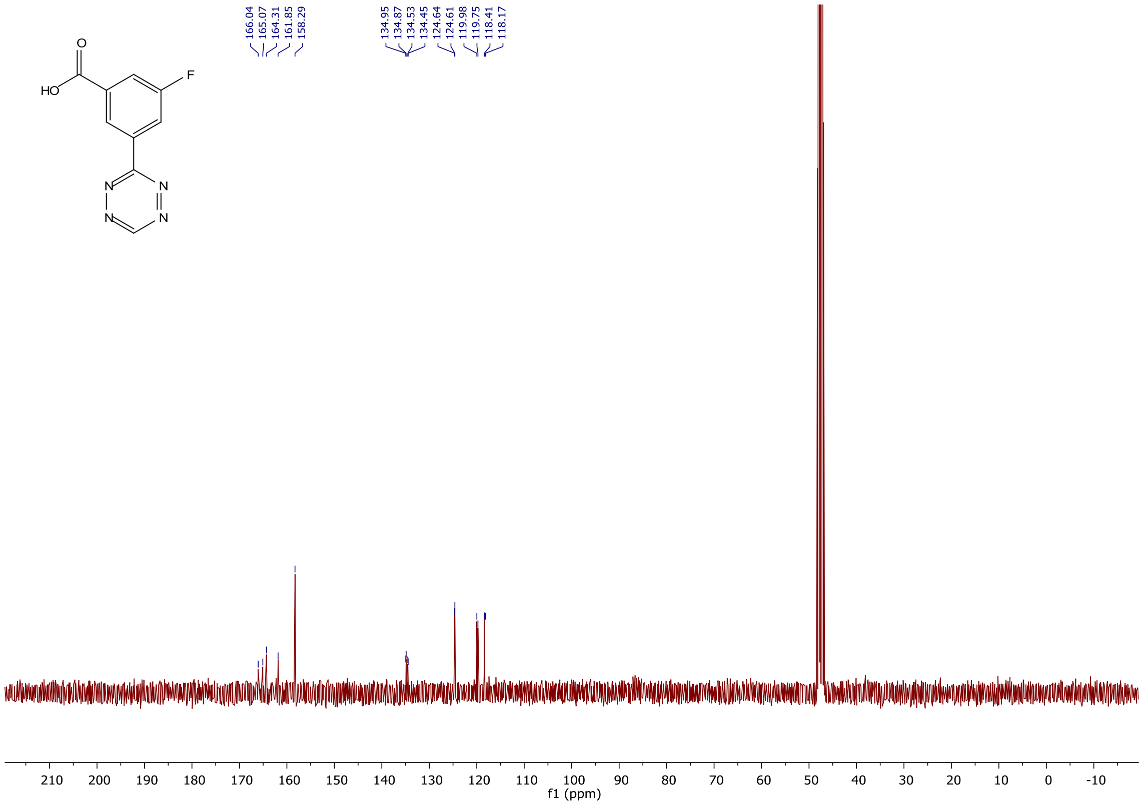

Analytical HPLC of 3-fluoro-5-(1,2,4,5-tetrazin-3-yl)benzoic acid (**19**)

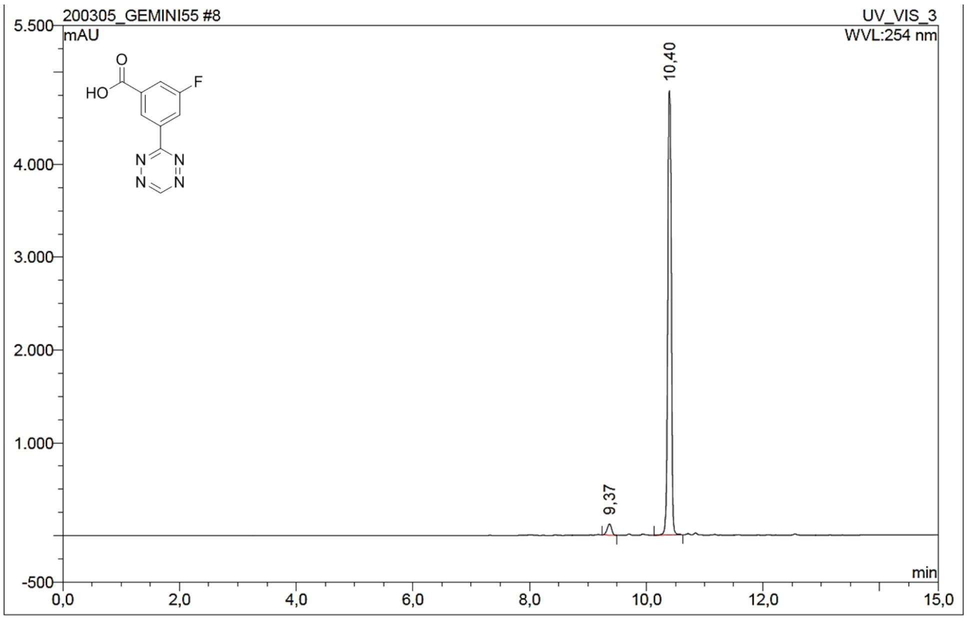

No.	Ret.Time min	Peak Name	Height mAU	Area mAU*min	Rel.Area %	Amount	Resolution(EP)
1	9,37	n.a.	121,321	10,980	3,22	n.a.	8,12
2	10,40	n.a.	4787,636	329,674	96,78	n.a.	n.a.
**Total:**			4908,957	340,653	100,00	0,000	

^{1}H NMR spectrum of 3-(bromomethyl)-5-fluorobenzonitrile (**21a**) (400 MHz, $CDCl_3$)

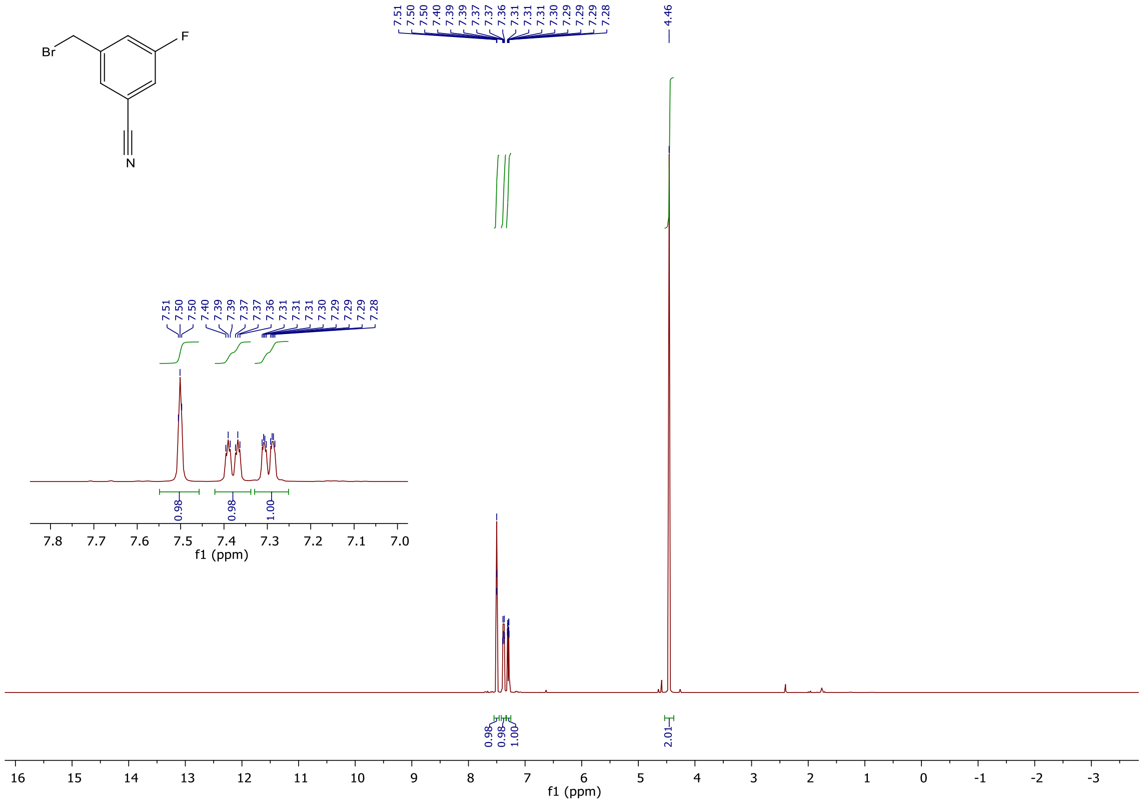

^{13}C NMR spectrum of 3-(bromomethyl)-5-fluorobenzonitrile (**21a**) (101 MHz, $CDCl_3$)

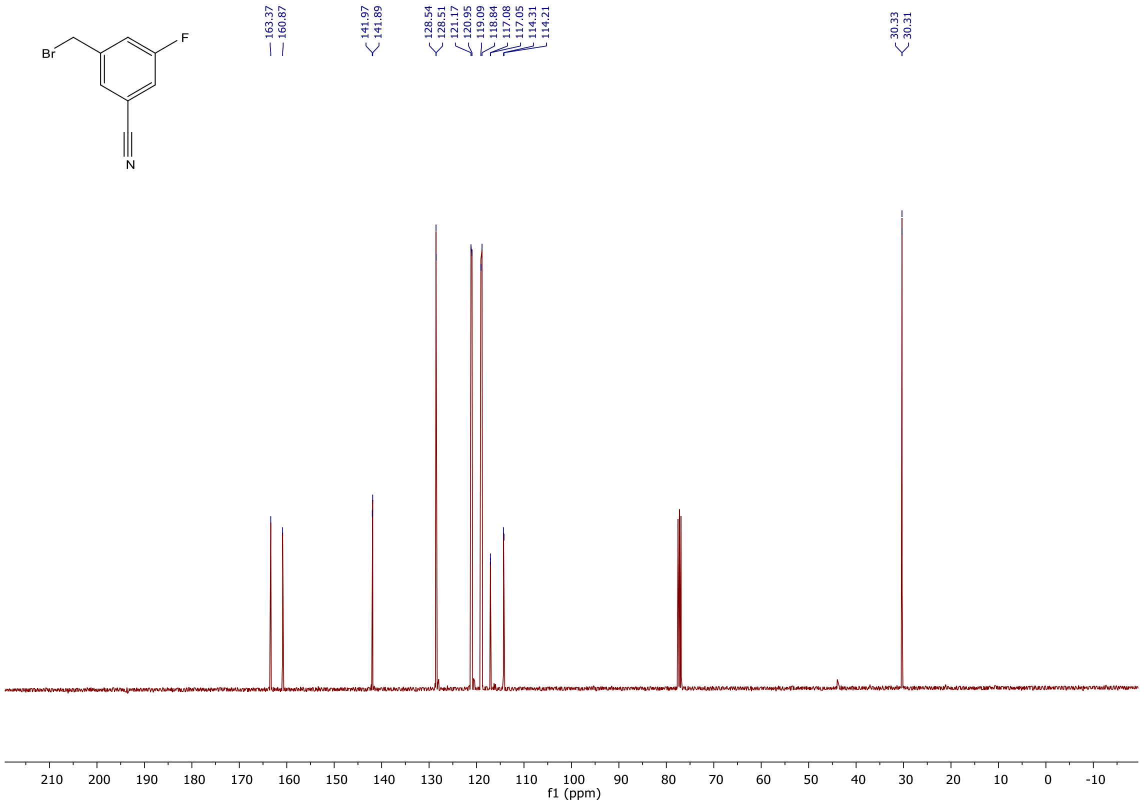

^{1}H NMR spectrum of di-tert-butyl 2,2'-((3-cyano-5-fluorobenzyl)azanediyl)diacetate (**21b**) (400 MHz, $CDCl_3$)

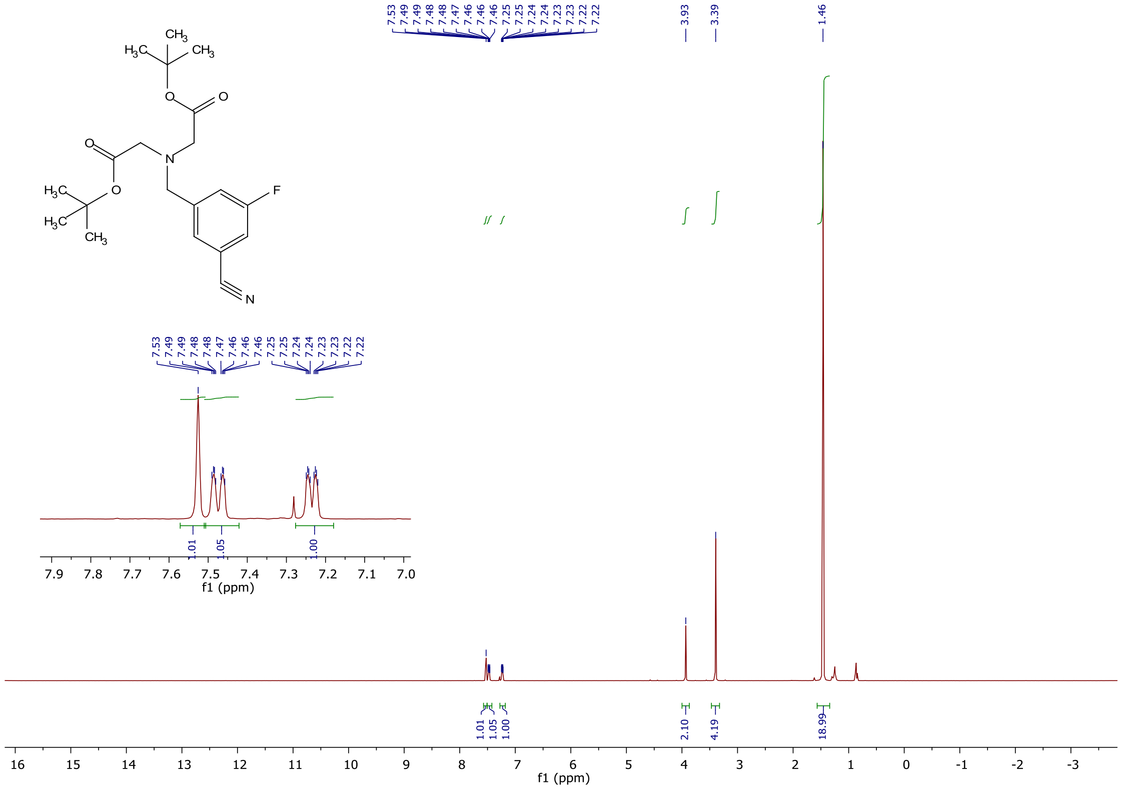

^{13}C NMR spectrum of di-tert-butyl 2,2'-((3-cyano-5-fluorobenzyl)azanediyl)diacetate (**21b**) (101 MHz, $CDCl_3$)

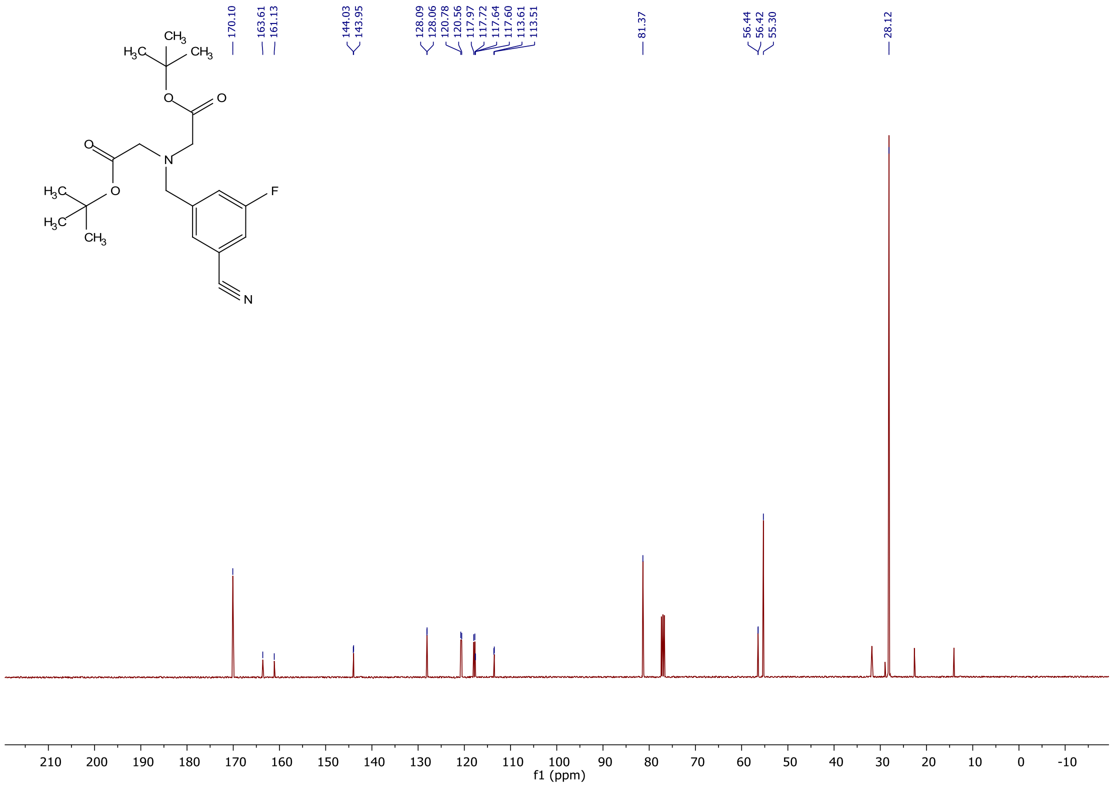

^{1}H NMR spectrum of di-tert-butyl di-tert-butyl 2,2'-((3-fluoro-5-(1,2,4,5-tetrazin-3-yl)benzyl)azanediyl)diacetate (**21c**) (400 MHz, $CDCl_3$)

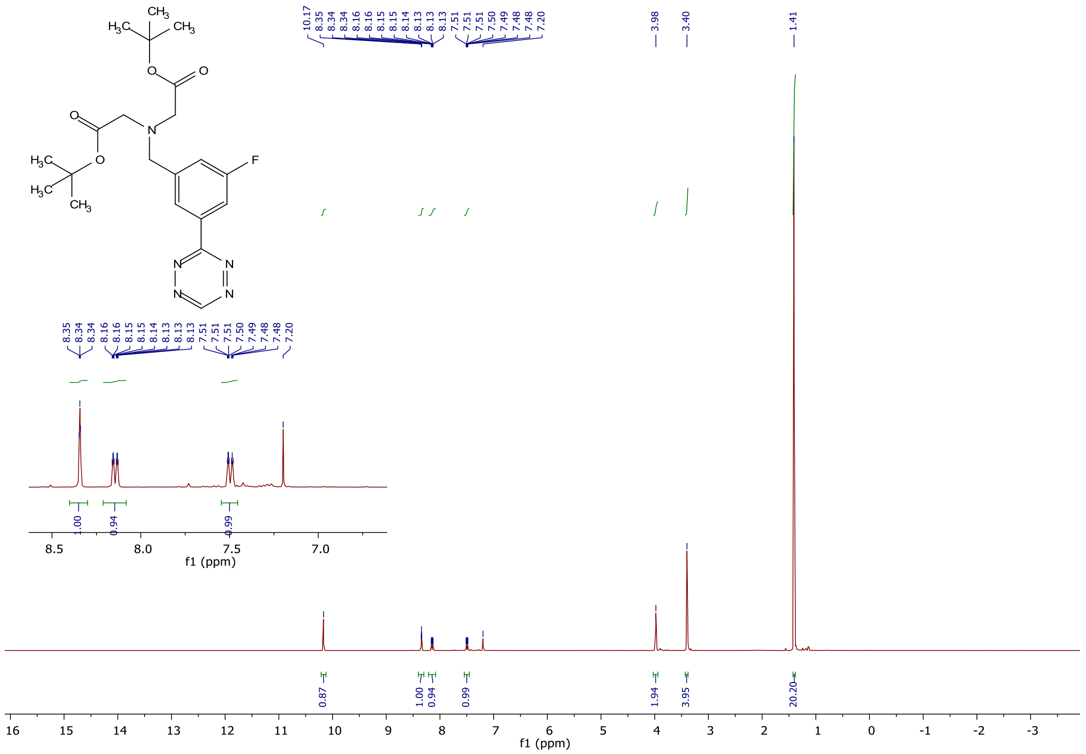

^{13}C NMR spectrum of di-tert-butyl 2,2'-((3-fluoro-5-(1,2,4,5-tetrazin-3-yl)benzyl)azanediyl)diacetate (**21c**) (101 MHz, $CDCl_3$)

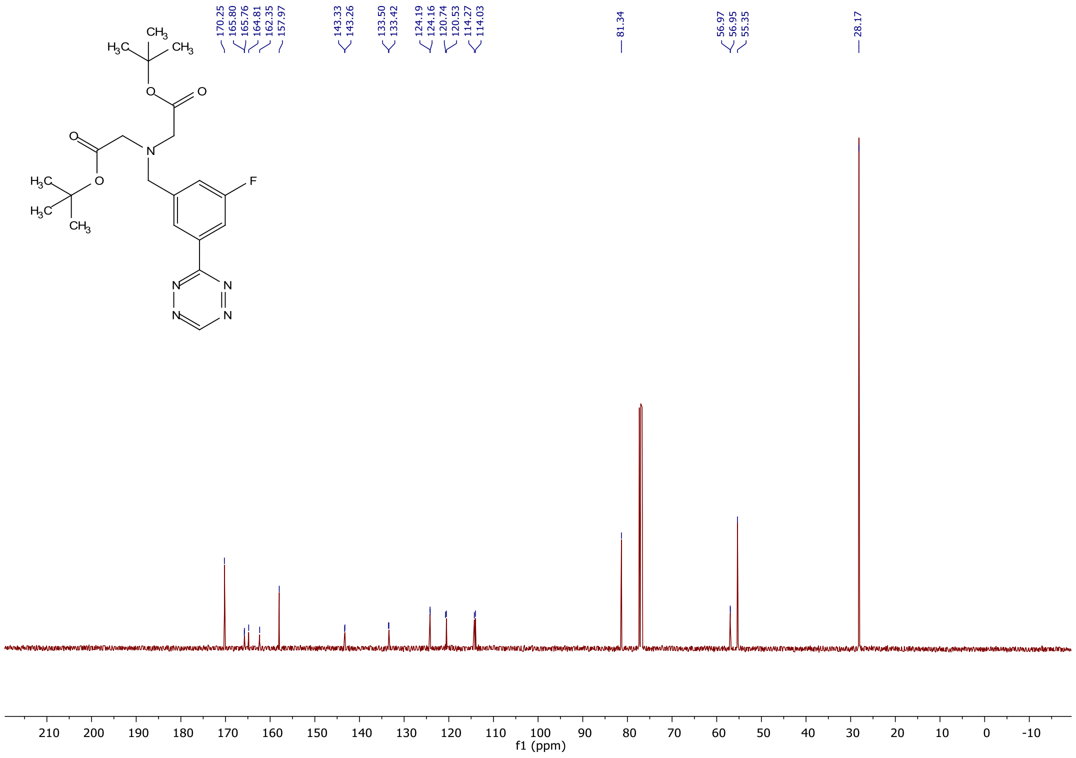

Analytical HPLC of di-tert-butyl 2,2'-((3-fluoro-5-(1,2,4,5-tetrazin-3-yl)benzyl)azanediyl)diacetate (**21c**)

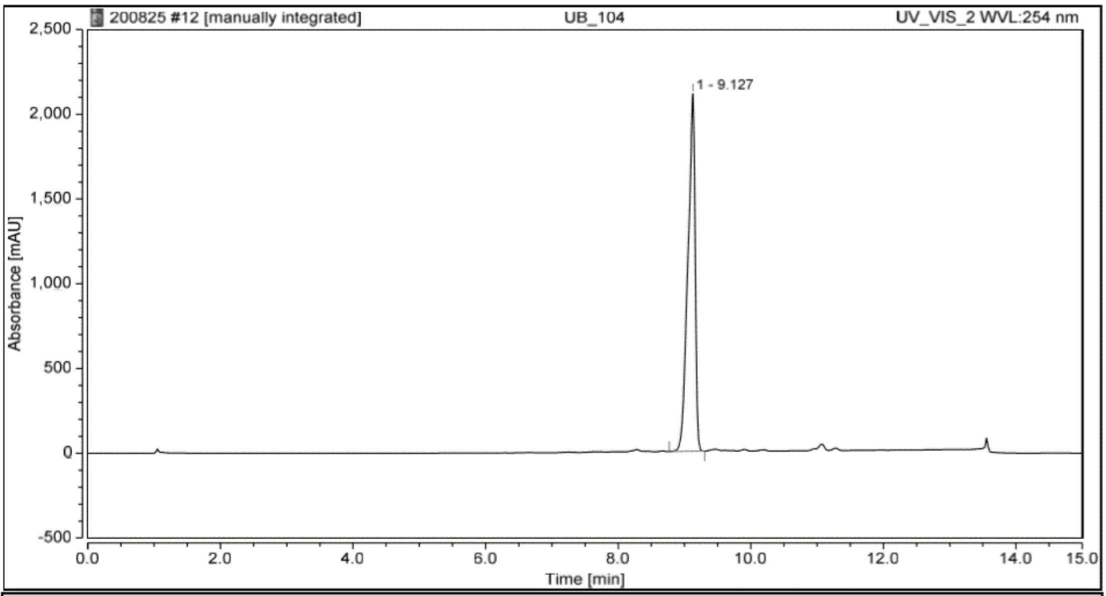

Integration Results

No.	Peak Name	Retention Time min	Area mAU*min	Height mAU	Relative Area %	Relative Height %	Amount
n.a.	Tosylate	n.a.	n.a.	n.a.	n.a.	n.a.	n.a.
n.a.	cold fallyprode	n.a.	n.a.	n.a.	n.a.	n.a.	n.a.
n.a.	18F-fallypride	n.a.	n.a.	n.a.	n.a.	n.a.	n.a.
1		9.127	264.205	2108.152	100.00	100.00	n.a.
**Total:**			**264.205**	**2108.152**	**100.00**	**100.00**	

^{1}H NMR spectrum of 1-Carboxy-N-(carboxymethyl)-N-(3-fluoro-5-(1,2,4,5-tetrazin-3-yl)benzyl)methanaminium 2,2,2-trifluoroacetate (**21**) (400 MHz, CD_3OD)

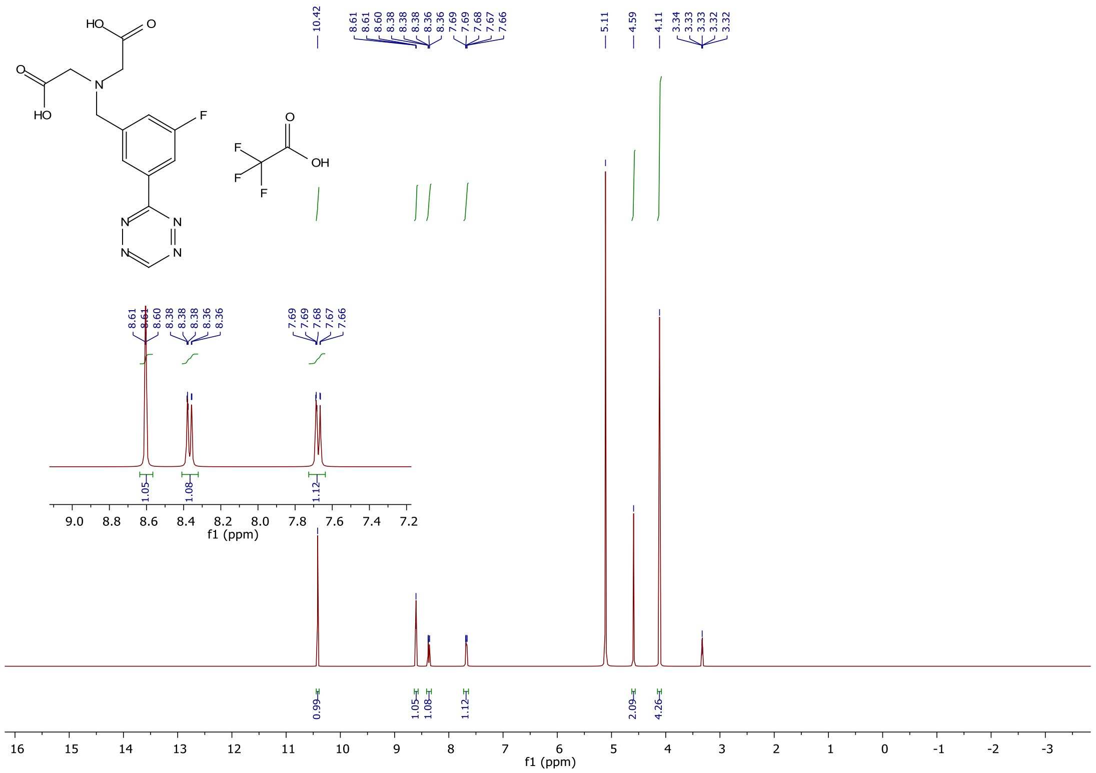

^{13}C NMR spectrum of 1-Carboxy-N-(carboxymethyl)-N-(3-fluoro-5-(1,2,4,5-tetrazin-3-yl)benzyl)methanaminium 2,2,2-trifluoroacetate (**21**) (101 MHz, CD_3OD)

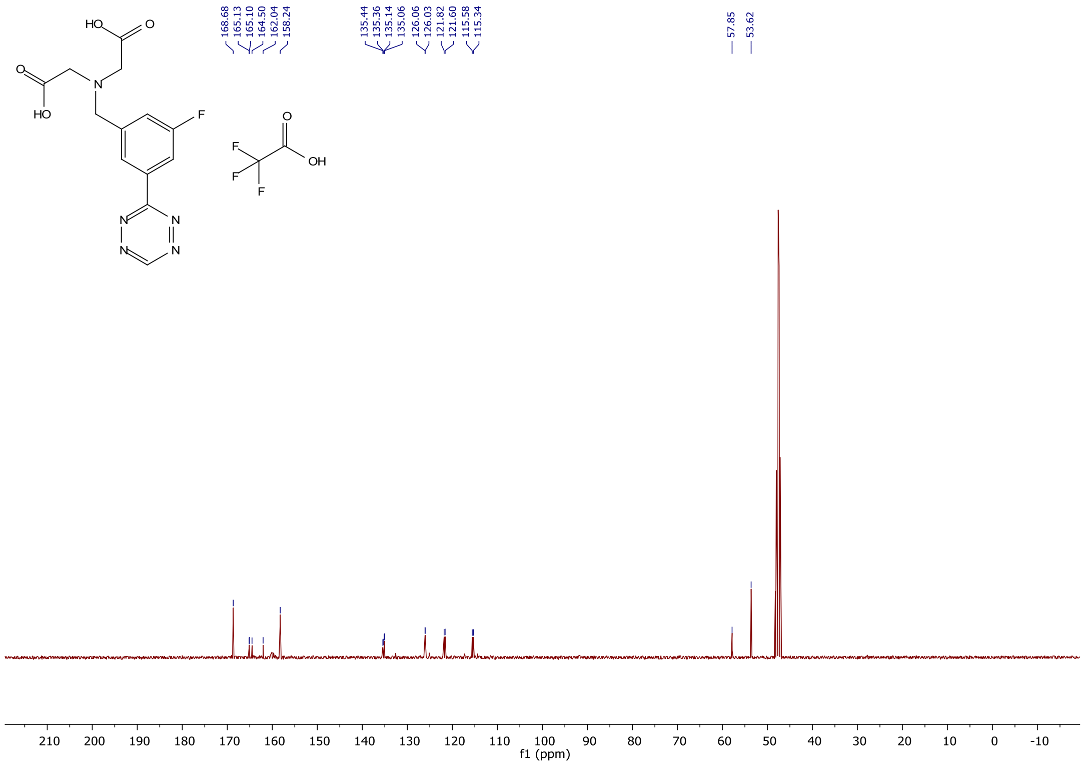

Analytical HPLC of 1-Carboxy-N-(carboxymethyl)-N-(3-fluoro-5-(1,2,4,5-tetrazin-3-yl)benzyl)methanaminium 2,2,2-trifluoroacetate (**21**)

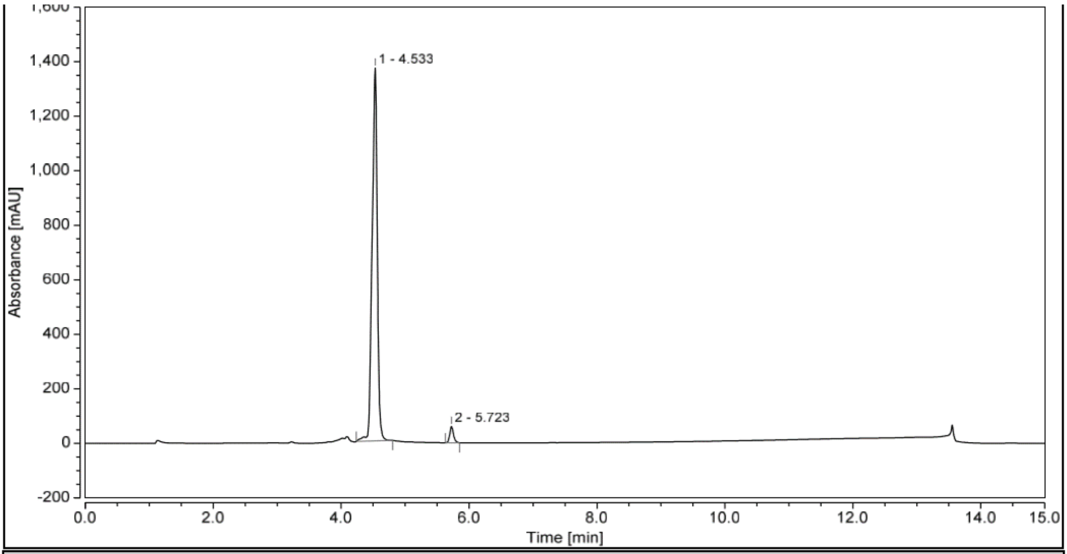

Integration Results

No.	Peak Name	Retention Time min	Area mAU*min	Height mAU	Relative Area %	Relative Height %	Amount
n.a.	Tosylate	n.a.	n.a.	n.a.	n.a.	n.a.	n.a.
1		4.533	126.570	1367.537	96.91	95.81	n.a.
n.a.	cold fallyprode	n.a.	n.a.	n.a.	n.a.	n.a.	n.a.
n.a.	18F-fallypride	n.a.	n.a.	n.a.	n.a.	n.a.	n.a.
2		5.723	4.037	59.862	3.09	4.19	n.a.
**Total:**			**130.607**	**1427.399**	**100.00**	**100.00**	

^{1}H NMR spectrum of 3-(bromomethyl)-5-iodobenzonitrile (**20a**) (400 MHz, $CDCl_3$)

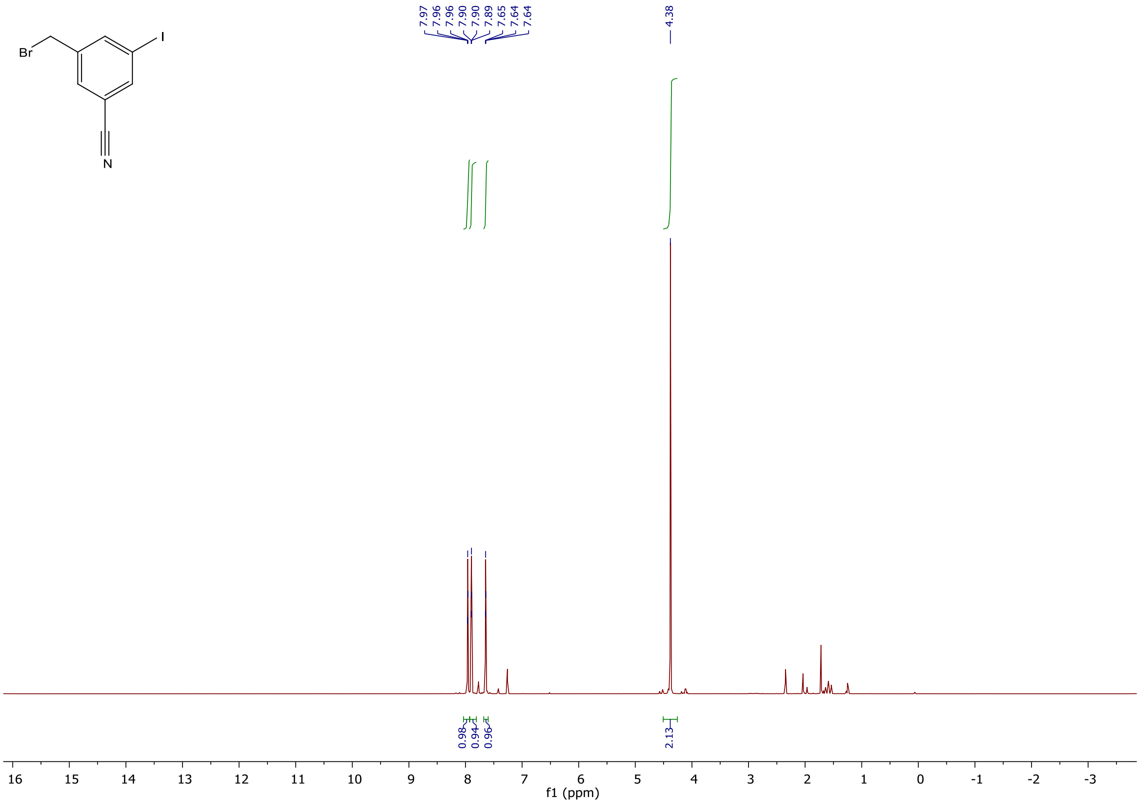

^{13}C NMR spectrum of 3-(bromomethyl)-5-iodobenzonitrile (**20a**) (101 MHz, $CDCl_3$)

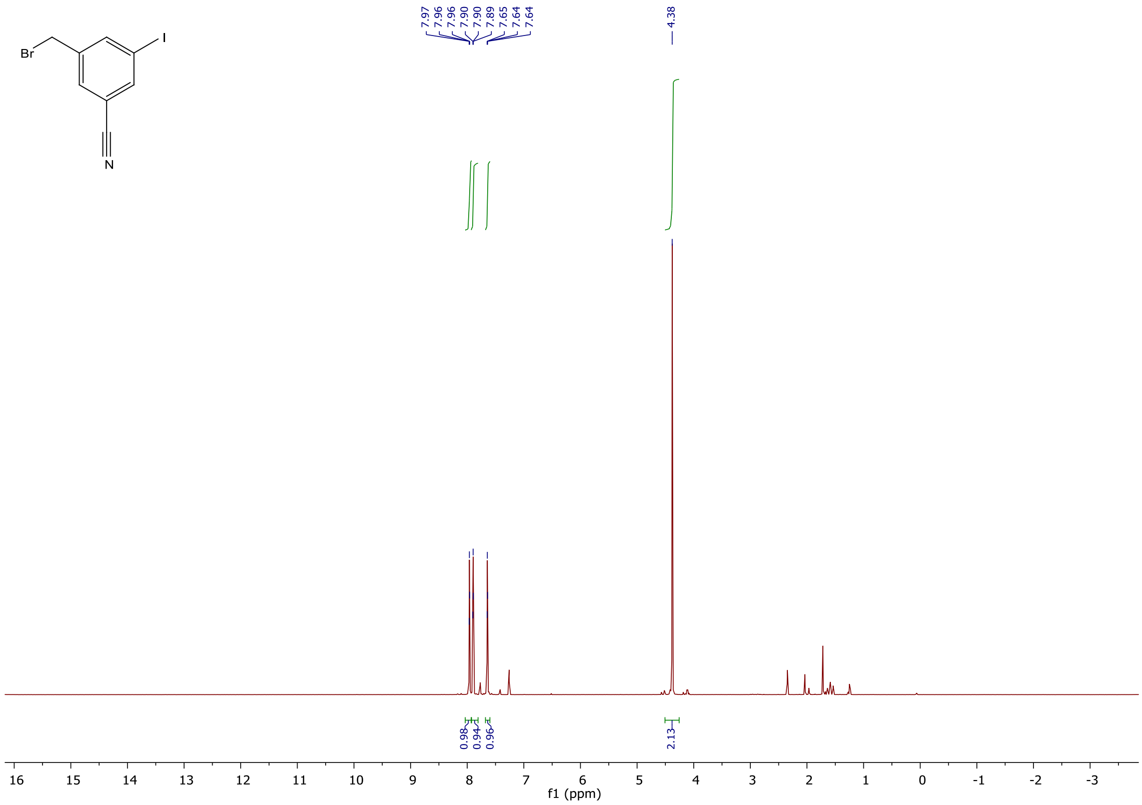

^{1}H NMR spectrum of di-tert-butyl 2,2'-((3-cyano-5-iodobenzyl)azanediyl)diacetate (**20b**) (400 MHz, $CDCl_3$)

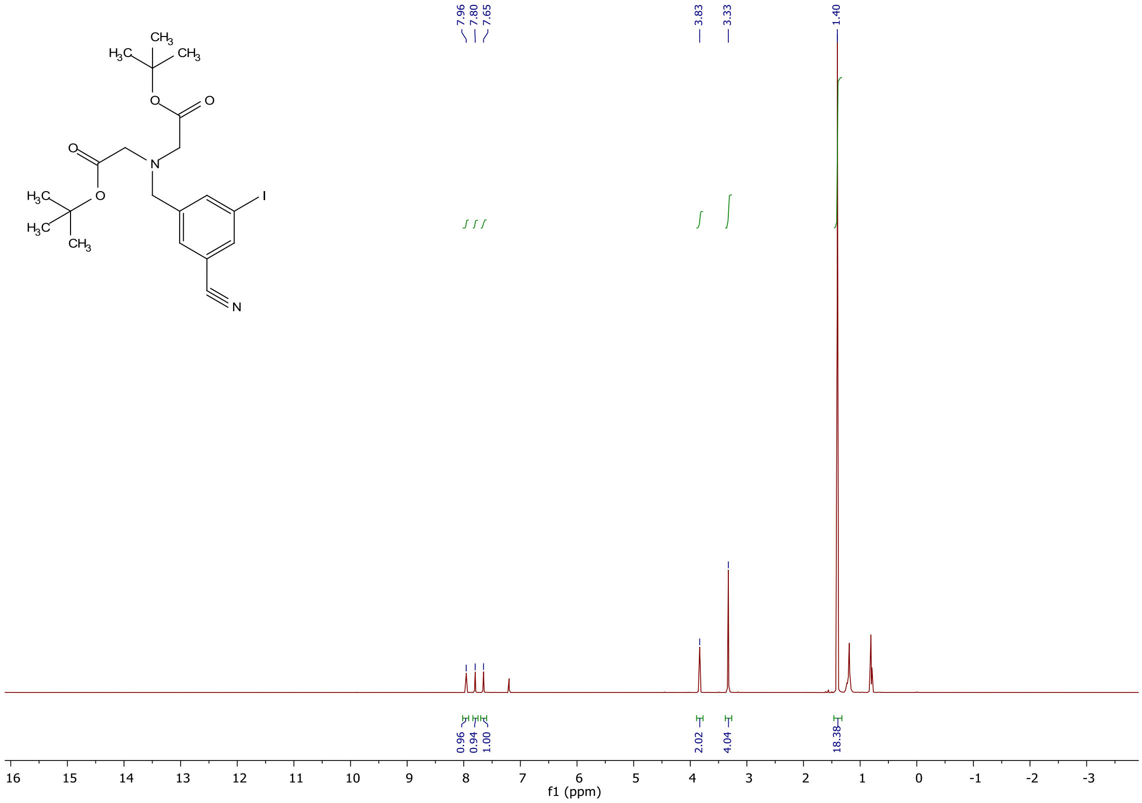

^{13}C NMR spectrum of di-tert-butyl 2,2'-((3-cyano-5-iodobenzyl)azanediyl)diacetate (**20b**) (101 MHz, $CDCl_3$)

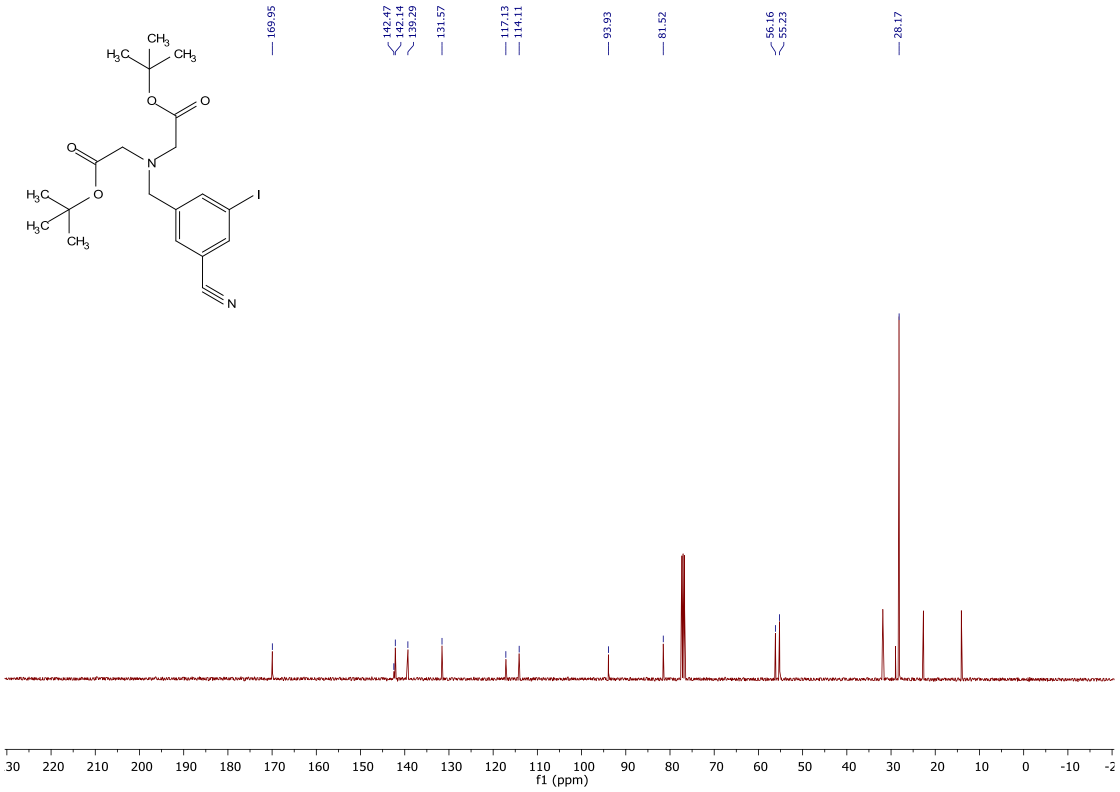

^{1}H NMR spectrum of di-tert-butyl 2,2'-((3-iodo-5-(1,2,4,5-tetrazin-3-yl)benzyl)azanediyl)diacetate (**20c**) (400 MHz, $CDCl_3$)

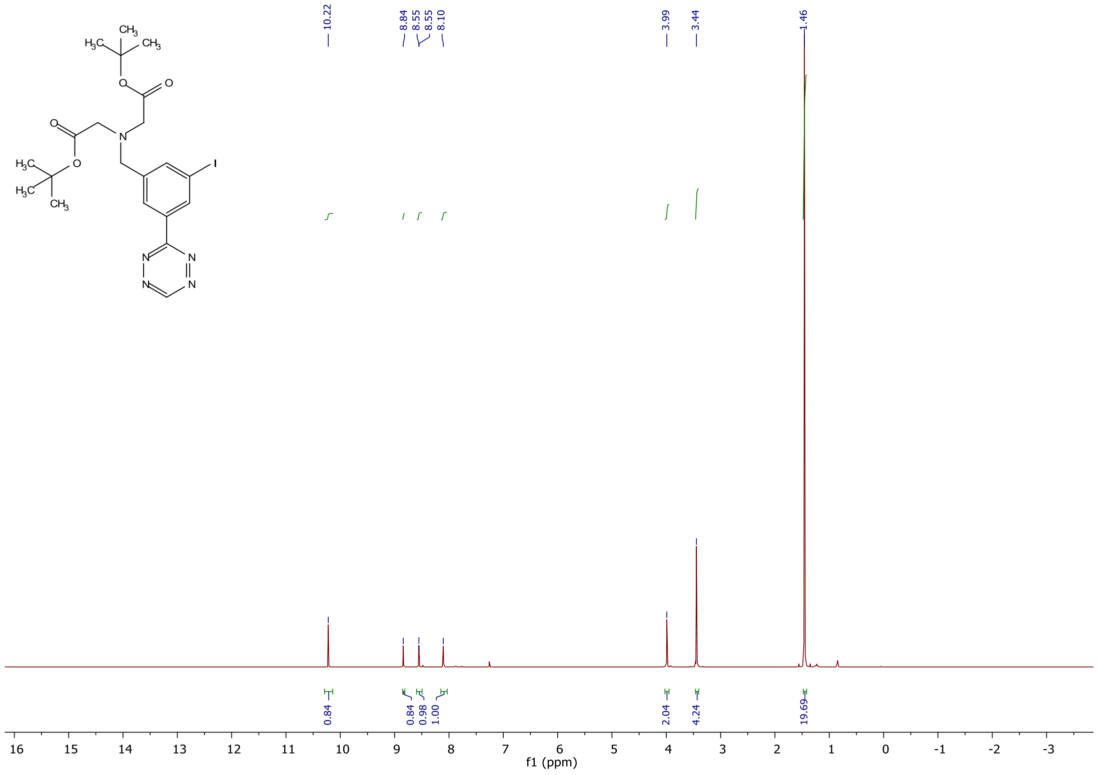

^{13}C NMR spectrum of di-tert-butyl 2,2'-((3-iodo-5-(1,2,4,5-tetrazin-3-yl)benzyl)azanediyl)diacetate (**20c**) (101 MHz, $CDCl_3$)

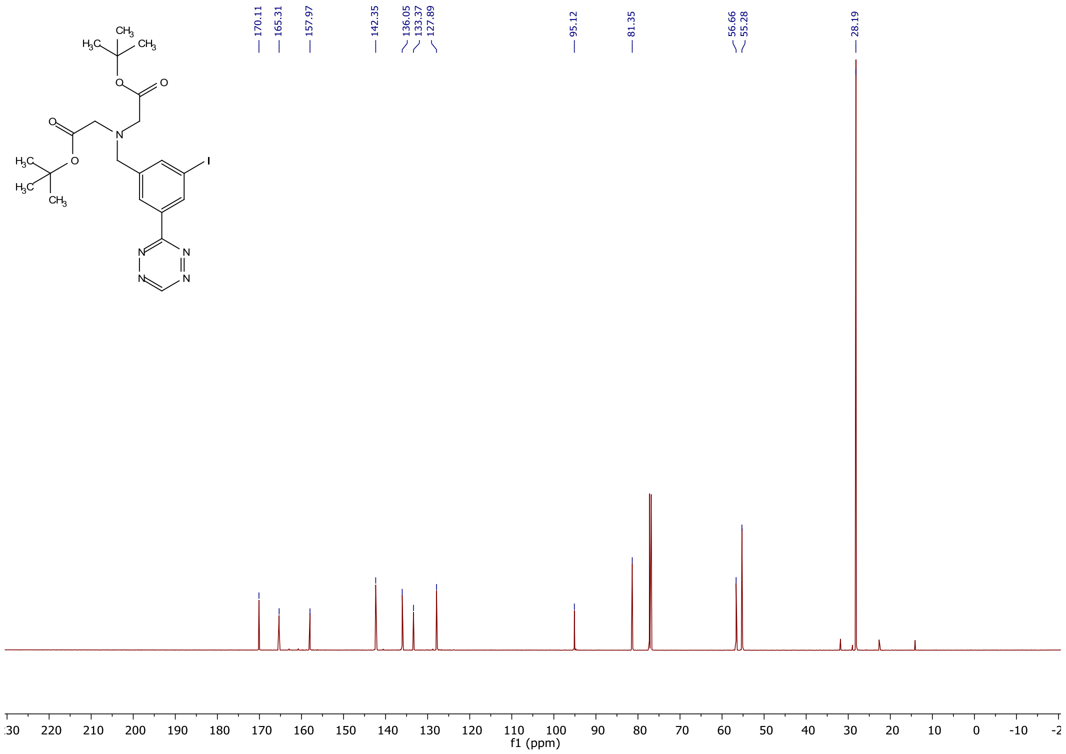

^{1}H NMR spectrum of di-tert-butyl 2,2'-((3-(1,2,4,5-tetrazin-3-yl)-5-(trimethylstannyl)benzyl)azanediyl)diacetate (**20**) (400 MHz, $CDCl_3$)

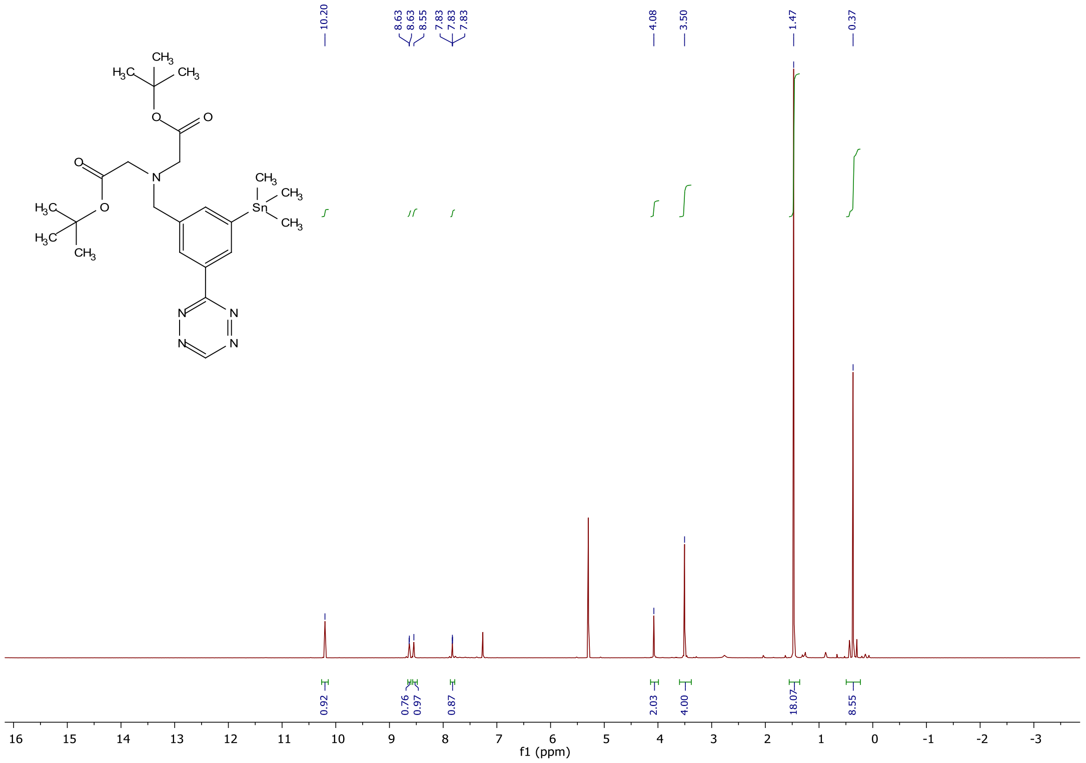

^{13}C NMR spectrum of di-tert-butyl 2,2'-((3-(1,2,4,5-tetrazin-3-yl)-5-(trimethylstannyl)benzyl)azanediyl)diacetate (**20**) (101 MHz, $CDCl_3$)

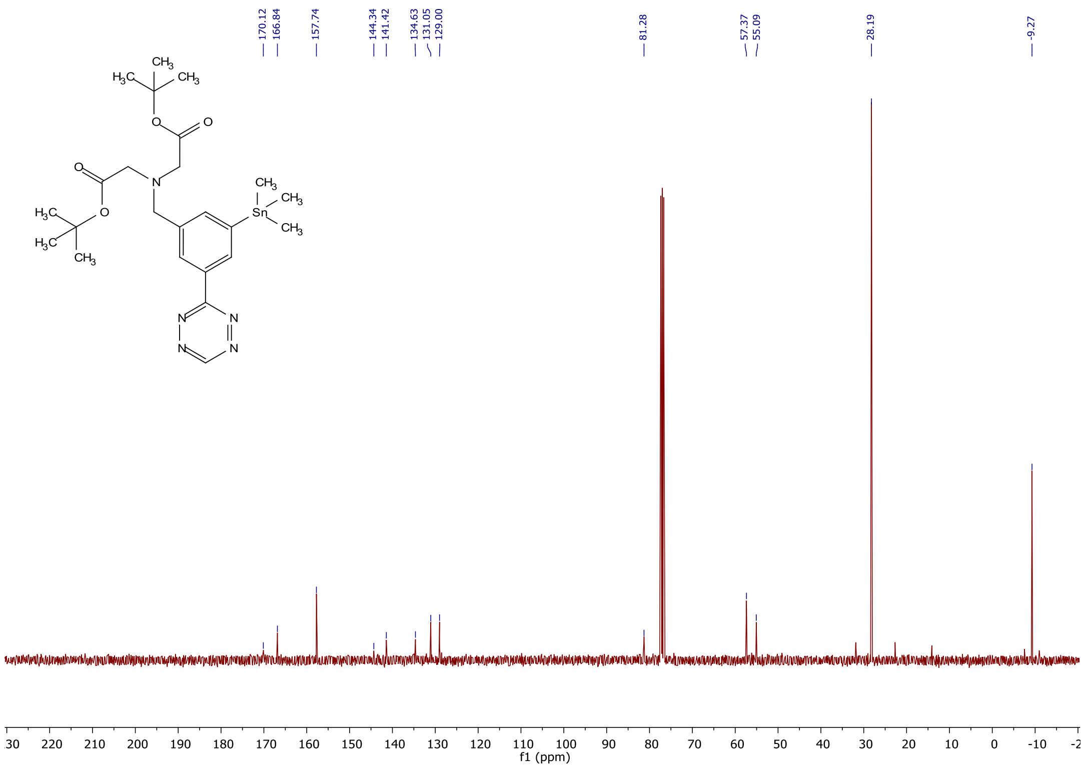

Analytical HPLC of di-tert-butyl 2,2'-((3-(1,2,4,5-tetrazin-3-yl)-5-(trimethylstannyl)benzyl)azanediyl)diacetate (**20**) (101 MHz, $CDCl_3$)

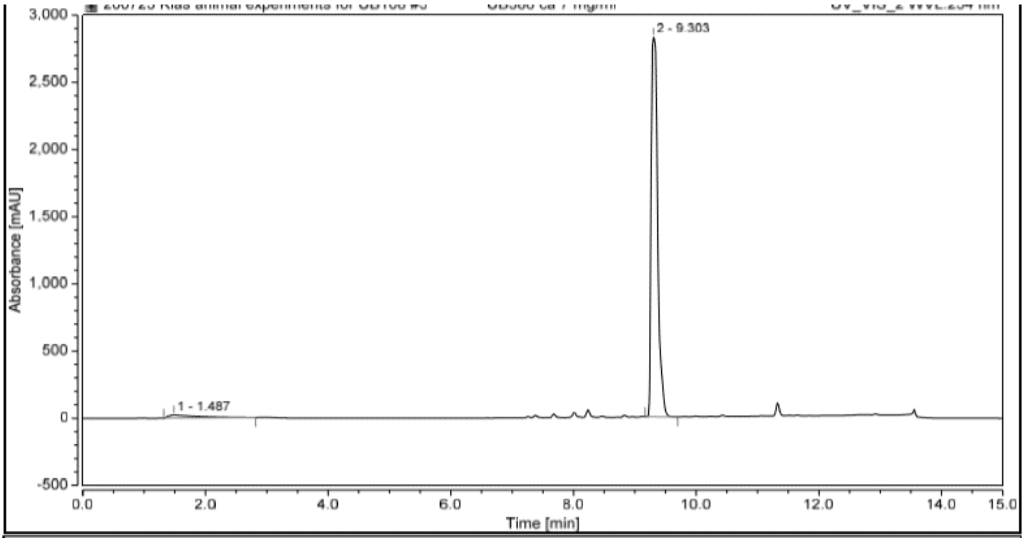

Integration Results

No.	Peak Name	Retention Time min	Area mAU*min	Height mAU	Relative Area %	Relative Height %	Amount
n.a.	Tosylate	n.a.	n.a.	n.a.	n.a.	n.a.	n.a.
1		1.487	13.440	23.499	3.75	0.82	n.a.
n.a.	cold fallyprode	n.a.	n.a.	n.a.	n.a.	n.a.	n.a.
n.a.	18F-fallypride	n.a.	n.a.	n.a.	n.a.	n.a.	n.a.
2		9.303	344.565	2826.206	96.25	99.18	n.a.
**Total:**			**358.005**	**2849.704**	**100.00**	**100.00**	
